# Benchmarking of different molecular docking methods for protein-peptide docking

**DOI:** 10.1186/s12859-018-2449-y

**Published:** 2019-02-04

**Authors:** Piyush Agrawal, Harinder Singh, Hemant Kumar Srivastava, Sandeep Singh, Gaurav Kishore, Gajendra P. S. Raghava

**Affiliations:** 1 Center for Computation Biology, Indraprastha Institute of Information Technology, Okhla Phase III, New Delhi, 110020 India; 2 CSIR-Institute of Microbial Technology, Sector 39A, Chandigarh, India

**Keywords:** Protein-peptide docking, Benchmark, CAPRI, ZDOCK, FRODOCK, Hex, ATTRACT, PatchDock, pepATTRACT

## Abstract

**Background:**

Molecular docking studies on protein-peptide interactions are a challenging and time-consuming task because peptides are generally more flexible than proteins and tend to adopt numerous conformations. There are several benchmarking studies on protein-protein, protein-ligand and nucleic acid-ligand docking interactions. However, a series of docking methods is not rigorously validated for protein-peptide complexes in the literature. Considering the importance and wide application of peptide docking, we describe benchmarking of 6 docking methods on 133 protein-peptide complexes having peptide length between 9 to 15 residues. The performance of docking methods was evaluated using CAPRI parameters like FNAT, I-RMSD, L-RMSD.

**Result:**

Firstly, we performed blind docking and evaluate the performance of the top docking pose of each method. It was observed that FRODOCK performed better than other methods with average L-RMSD of 12.46 Å. The performance of all methods improved significantly for their best docking pose and achieved highest average L-RMSD of 3.72 Å in case of FRODOCK. Similarly, we performed re-docking and evaluated the performance of the top and best docking pose of each method. We achieved the best performance in case of ZDOCK with average L-RMSD 8.60 Å and 2.88 Å for the top and best docking pose respectively. Methods were also evaluated on 40 protein-peptide complexes used in the previous benchmarking study, where peptide have length up to 5 residues. In case of best docking pose, we achieved the highest average L-RMSD of 4.45 Å and 2.09 Å for the blind docking using FRODOCK and re-docking using AutoDock Vina respectively.

**Conclusion:**

The study shows that FRODOCK performed best in case of blind docking and ZDOCK in case of re-docking. There is a need to improve the ranking of docking pose generated by different methods, as the present ranking scheme is not satisfactory. To facilitate the scientific community for calculating CAPRI parameters between native and docked complexes, we developed a web-based service named PPDbench (http://webs.iiitd.edu.in/raghava/ppdbench/).

**Electronic supplementary material:**

The online version of this article (10.1186/s12859-018-2449-y) contains supplementary material, which is available to authorized users.

## Background

Protein-peptide interactions are essential in various biological processes involving signaling, cellular localization, immune system, and apoptotic pathways. Such interactions serve as structural components in approximately 40% of all macromolecular interactions [1, 2]. Peptides can be used to prevent diseases involving malfunctioning of proteins due to undesirable protein-protein interactions [3, 4]. Many databases and algorithms have been developed in the past specifically in the field of peptide-based therapeutics [5–15]. There are more than 200 therapeutics peptides, approved by FDA for the treatment of various diseases [16, 17]. Peptides are more flexible than proteins and tend to adopt numerous conformations. Thus, modeling protein-peptide interactions is a challenging and time-consuming task [18].

Numerous docking methods have been developed in the past for structural determination of protein-peptide complexes. Broadly, these methods can be classified into the following 3 categories; i) protein-peptide docking, ii) protein-protein docking and iii) protein-small molecule docking. Protein-peptide docking methods [19–28] have been specifically developed to dock peptide on protein like pepATTRACT, FlexPepDock, HADDOCK, PEP-SiteFinder, etc. Though protein-protein docking methods [28–39] have been developed for docking two proteins; some of these methods, for example, ZDOCK, Hex can also be used to dock peptide on a protein. Similarly, some of the software developed for docking small-molecules on a protein [40–48] can also be used to dock peptide on a protein, for example, AutoDock and AutoDock Vina. In summary, a wide range of docking methods have been developed in past that can be used directly or indirectly for docking peptide on a protein.

Prediction of peptide interaction with receptor protein is highly desirable to design peptide-based therapeutics. However, utility of any prediction is entirely dependent on the accuracy of the prediction. All the above docking methods can be used to predict the interaction between protein and peptides, thus evaluating the performance of these methods is essential to understand their pros and cons. Also, benchmarking is required to develop highly accurate docking methods that can overcome the limitation of the existing methods. There are a number of benchmarking studies on protein-protein [49, 50], protein-ligand [51, 52] and nucleic acid-ligand [53] docking interactions. Comparatively, limited attempts had been made to benchmark docking method on protein-peptide complexes. Spoel and coworkers evaluated the capability of AutoDock for docking studies of a set of 8 protein-peptide complexes without having the prior knowledge of the binding site [54]. Rentzsch and Renard assessed the performance of AutoDock Vina on a meta-data set of 47 protein-peptide complexes [55]. Recently, Hauser and Windshugel developed a LEADS-PEP dataset to evaluate the performance of peptide docking methods [56]. The major limitation of existing benchmarking studies is that they evaluated only a limited number of docking methods, as well as dataset used for evaluation contain small number of protein-peptide complexes. In addition, there is no platform or web server where users can benchmark or evaluate the performance of their newly developed docking method.

In order to facilitate scientific community and to complement previous benchmarking studies, we made a systematic attempt to benchmark docking methods on a large set of protein-peptide complexes. The main aim of this study is to evaluate the performance of major docking methods, as well as evaluation of scoring function used by docking methods. We also perform a wide range of analysis to understand the impact of the absence of the binding site information, type of secondary structure, and other molecular properties on the performance of docking method. It is not practically possible to evaluate all docking methods. Thus we select those methods, which broadly satisfy following conditions; i) available free for public use, ii) standalone version is available, iii) widely used by scientific community and iv) performed well in the Critical Assessment of PRediction of Interactions (CAPRI) competition. CAPRI is an international competition which provides a framework for evaluating protein-protein interactions/docking and refinement methods by blind testing on the set of unpublished targets [30, 36, 57–61]. Finally we select following 6 software for benchmarking; ZDOCK 3.0.2, FRODOCK 2.0, Hex 8.0.0, PatchDock 1.0, ATTRACT and pepATTRACT. ZDOCK 3.0.2 is a rigid body docking method based on the Fast Fourier Transform algorithm, and its scoring function is a combination of pairwise shape complementarity, desolvation and electrostatic energy [62–64]. ATTRACT is a flexible protein-protein docking method based on randomized search algorithm and employs Lennard-Jones potential and electrostatic energy as a scoring function [30, 39]. Hex 8.0.0 is another popular method which uses Spherical Polar Fourier (SPF) correlations algorithm rather than Fast Fourier Transform (FFT) based search [65, 66]. FRODOCK 2.0 is a rigid body docking algorithm and is based on the principle of 3D grid-based potentials with knowledge-based potential and spherical harmonics (SH) properties which help in improving docking success rate more significantly [35, 36]. pepATTRACT is a flexible protein-peptide docking algorithm which performs a rapid coarse-grained global search on the protein surface and model peptide simultaneously during docking [67]. PatchDock 1.0 is also a rigid body docking software which considers surface variability or flexibility implicitly marked through liberal intermolecular penetration. Its scoring function is based on geometry fit and atomic desolvation energy [47]. The standalone versions of ZDOCK 3.0.2, Hex 8.0.0 and PatchDock 1.0 docking methods are available for use on the local machine. FRODOCK 2.0 provides an online web service where a user can upload their docking partner for docking. ATTRACT, and pepATTRACT provides a ready-to-use script, which can be downloaded from their web-based service for performing docking of each complex. The main dataset created and used in this study comprises of highly annotated 133 protein-peptide complexes; Table 1 shows detailed information about these complexes. Also, methods were evaluated on datasets used in previous studies.

**Table 1:** The list of all the considered complexes along with their PDB-IDs

Sr. No.	PDB-ID	Classification	Protein Chain	Peptide Chain	Peptide Length	PDB Resolution (Å)
1	1CJR	Hydrolase	B	A	15	2.30
2	1CKA	Oncogene protein	A	B	9	1.50
3	1D4T	Signaling protein	A	B	11	1.10
4	1EG4	Structural protein	A	P	13	2.00
5	1H6W	Structural protein	A	B	10	1.90
6	1HC9	Toxin protein	A	C	13	1.80
7	1JBU	Hydrolase	H	X	15	2.00
8	1MFG	Signaling protein	A	B	9	1.25
9	1NLN	Hydrolase	A	B	11	1.60
10	1NQ7	Transcription	A	B	10	1.50
11	1NTV	Signaling protein	A	B	10	1.50
12	1NX1	Hydrolase inhibitor	A	C	11	2.00
13	1OAI	Nuclear transport	A	B	9	1.55
14	1OJ5	Transcriptional activator	A	B	14	2.21
15	1OW6	Transferase	A	D	12	2.35
16	1PZL	Transcription	A	B	14	2.10
17	1QKZ	Immune system	H	P	10	1.95
18	1RXZ	Replication	A	B	11	2.00
19	1SFI	Hydrolase	A	I	14	1.65
20	1SSH	Contractile protein	A	B	11	1.40
21	1 T08	Cell cycle protein	A	C	15	2.10
22	1T4F	Ligase	M	P	9	1.90
23	1T7R	Growth factor protein	A	B	10	1.40
24	1TFC	Transcription	A	C	11	2.40
25	1 U00	Chaperone protein	A	P	9	1.95
26	1UJ0	Signaling protein	A	B	9	1.70
27	1X2R	Transcription	A	B	9	1.70
28	1XOC	Transport protein	A	B	9	1.55
29	1YMT	Transcription	A	B	10	1.20
30	1YUC	Transcriptional Regulation	A	C	14	1.90
31	1YWO	Signaling protein	A	P	10	1.81
32	2A31	Transferase	A	B	12	1.25
33	2AQ9	Transferase	A	X	12	1.80
34	2B9H	Transferase	A	C	12	1.55
35	2BBA	Signaling protein	A	P	14	1.65
36	2CCH	Cell cycle protein	D	F	12	1.70
37	2D0N	Signaling protein	C	D	9	1.57
38	2DRK	Contractile protein	A	B	10	1.42
39	2FFF	Transferase	B	A	15	2.23
40	2FKA	Signaling protein	A	B	10	2.00
41	2FMF	Signaling protein	A	B	13	1.99
42	2FTS	Structural protein	A	P	13	2.41
43	2FVJ	Signaling protein	A	B	10	1.99
44	2HO2	Protein binding	A	B	10	1.33
45	2HT9	Oxidoreductase	A	X	12	1.90
46	2O02	Toxin protein	A	P	14	1.50
47	2O4J	Growth factor protein	A	C	12	1.74
48	2O9V	Signaling protein	A	B	10	1.63
49	2P0W	Transferase	A	P	15	1.90
50	2P1O	Hydrolase	B	C	13	1.90
51	2P1T	Hormone receptor	A	B	10	1.80
52	2P54	Transcription	A	B	12	1.79
53	2PEH	Protein binding	A	C	10	2.11
54	2PUX	Hydrolase	B	C	13	2.00
55	2PUY	Transcription	B	E	10	1.43
56	2QBX	Signaling protein	B	D	11	2.30
57	2QOS	Immune System	C	A	11	1.81
58	2QSE	Transcription	B	D	11	1.85
59	2R7G	Transcription repressor	C	D	10	1.67
60	2V8Y	Translation	A	B	14	2.10
61	2VR3	Cell adhesion protein	B	D	13	1.95
62	2VWF	Signaling	A	B	14	1.58
63	2W2U	Hydrolase	A	C	11	2.20
64	2WHX	Hydrolase	A	C	14	2.20
65	2XRW	Transcription	A	B	12	1.33
66	2XU7	Transcription	B	C	12	1.90
67	2XVC	Cell cycle protein	A	B	13	2.15
68	2ZJD	Apoptosis protein	A	B	10	1.56
69	3AWR	Transport protein	A	C	12	2.00
70	3AYU	Hydrolase	A	B	10	2.00
71	3BFQ	Structural	G	F	15	1.34
72	3C3R	Transport	A	B	13	2.02
73	3D32	Transport	A	C	12	1.30
74	3DS4	Viral protein	A	T	12	1.12
75	3FDO	Cell cycle protein	A	B	12	1.40
76	3G2S	Signaling protein	A	C	11	1.70
77	3GYT	Transcription	A	B	10	2.40
78	3H1Z	Transferase	A	P	15	1.83
79	3KMR	Transcription	A	C	10	1.80
80	3KUJ	Protein binding	A	B	15	1.40
81	3KUS	Protein binding	A	C	11	1.40
82	3L0E	Transcription	A	B	12	2.30
83	3LLZ	Sugar binding	A	B	14	1.55
84	3OLF	Hormone protein	A	B	11	1.90
85	3P72	Clotting protein	A	B	11	1.90
86	3P8F	Hydrolase	A	I	14	2.00
87	3PTL	Hydrolase	A	B	10	1.30
88	3QIS	Hydrolase	A	B	13	2.30
89	3RQG	Protein binding	C	E	12	2.50
90	3SFJ	Signaling protein	A	B	10	1.24
91	3SO6	Protein binding	A	Q	13	1.37
92	3TZY	Transferase	B	C	10	2.20
93	3UP3	Transcription	A	P	13	1.25
94	3V2X	Protein binding	A	B	11	1.85
95	3VTC	Transcription	A	B	11	1.50
96	3W1B	Ligase	A	B	10	2.40
97	3ZQH	Transcription	A	C	12	1.60
98	4B4N	Viral protein	A	B	15	1.81
99	4DCB	Hydrolase	A	F	11	2.03
100	4E34	Transport	B	D	10	1.39
101	4EIK	Protein binding	A	B	11	1.60
102	4ERY	Transcription	A	D	14	1.30
103	4F14	Actin binding	A	B	11	1.20
104	4F1Z	Cell adhesion protein	A	Q	14	2.30
105	4GQ6	Transcription	A	B	12	1.55
106	4GXL	Protein binding	A	B	11	2.02
107	4GYW	Transferase	A	B	14	1.70
108	4H4F	Hydrolase	A	Q	10	1.90
109	4HOM	Hydrolase	A	B	11	1.90
110	4HTP	Hydrolase	A	C	10	2.25
111	4IIM	Endocytosis	A	C	12	1.80
112	4J8S	Protein binding	A	B	12	1.55
113	4K0U	Protein transport	A	B	15	2.15
114	1CVU	Oxidoreductase	B	F	9	2.40
115	1K5N	Immune system	A	C	9	1.09
116	1OU8	Transport	A	C	11	1.60
117	1RST	Signaling	B	P	9	1.70
118	2A25	Ligase	A	B	9	2.20
119	2 CE8	Transcription	A	X	9	2.03
120	2DYP	Immune system	A	C	9	2.50
121	2FFU	Transferase	A	P	9	1.64
122	2OEI	Protein binding	A	B	9	1.35
123	2R9Q	Hydrolase	B	Y	9	2.20
124	2VKN	Membrane protein	A	C	9	2.05
125	3ASL	DNA binding protein	A	B	9	1.41
126	3ERY	Immune system	A	P	9	1.95
127	3I5R	Protein binding	A	B	9	1.70
128	3IVV	Ligase	A	D	10	1.25
129	3LL8	Protein binding	A	E	11	2.00
130	3OBQ	Transport	A	B	9	1.40
131	3RM1	Protein binding	A	B	9	1.24
132	3TJV	Hydrolase	A	B	9	2.40
133	3U9Q	Transcription	A	B	9	1.52

## Results and discussion

**Fig. 1:**
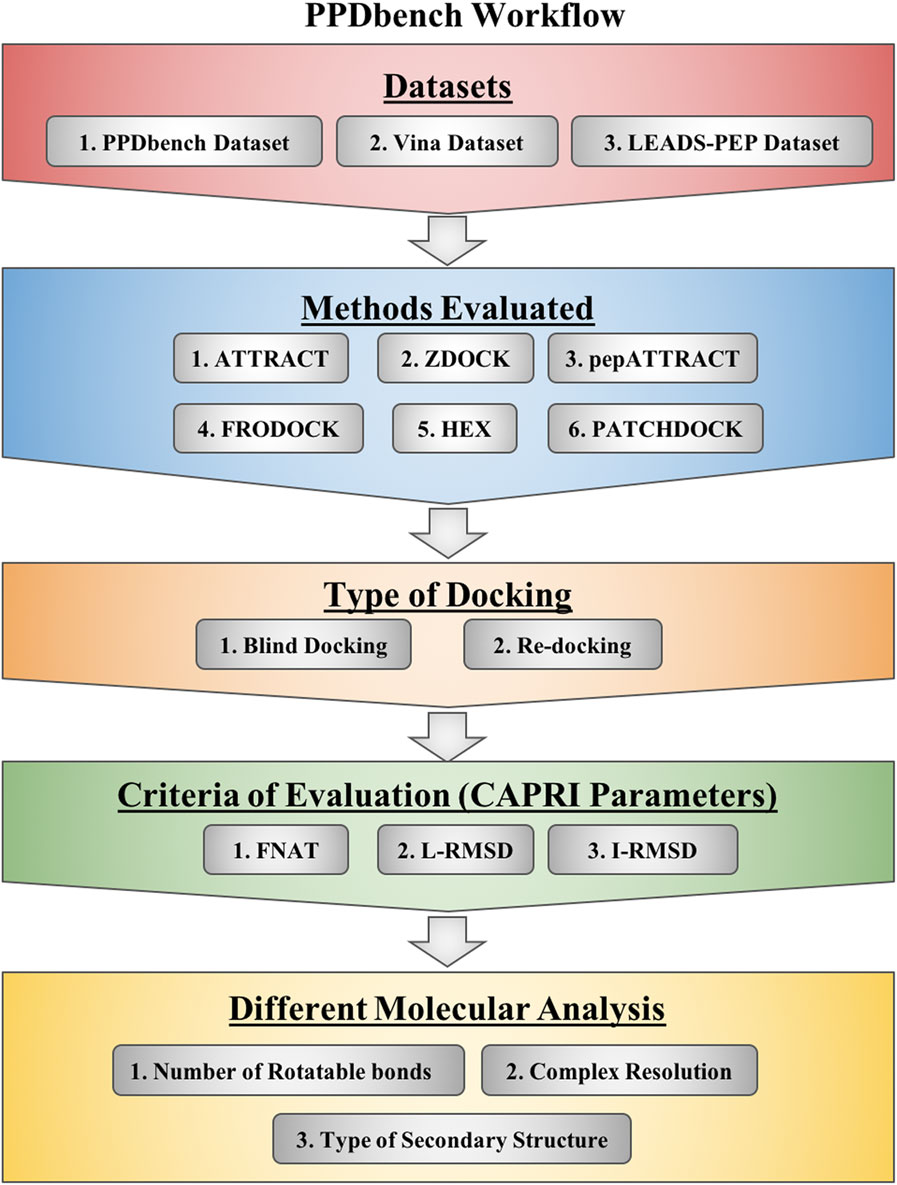
Schematic representation of the workflow of PPDbench algorithm

In this study, a dataset called PPDbench has been used to evaluate the performance of 6 docking methods. This dataset contains 133 non-redundant complexes of protein-peptides at 40%, it means no two proteins have more than 40% sequence similarity. We removed redundancy using commonly used software CD-HIT; detail procedure is given in the Material and Method section. In total, 117 clusters were obtained out of which similarity among the proteins was present only in 12 clusters. Detail information of the clustering result is provided in Additional file 1: S1. We used CAPRI parameters (e.g., FNAT, I-RMSD, and L-RMSD) and the following 3 steps for evaluating the performance of the methods. In the first step, the structure of a protein-peptide complex is obtained from PPDbench. In the second step, docking methods use structures of protein and peptide to predict the structure of the protein-peptide complex. Finally, the performance of docking methods is determined by comparing the predicted and actual structure of a protein-peptide complex. Overview of the PPDbench algorithm has been shown in Figure 1.

### Shifting Cartesian coordinates of peptide structure for blind docking

Docking method requires structural coordinates of the protein and peptide for docking peptide on the protein. As we are providing structure coordinates of both peptide and protein from the original complex, it means we are giving actual docking pose to the docking software. This docking pose information may affect the performance of a docking method. In order to avoid biasness in the evaluation, we shifted Cartesian coordinate of the structure without changing the structure of the peptide, i.e. dihedral angles of both original and modified (shifted Cartesian coordinates) peptide remains the same (see Material and Methods). We compute the backbone RMSD (B-RMSD) between the actual and modified structure to verify that shifting of coordinates does not affect peptide structure too much. It was observed that B-RMSD value ranges from 0.067 to 0.827 for 133 peptides with 123 peptides showing value ≤0.5 Å. In order to understand the shifting of peptide structure from the original position, we compute the distance between actual and modified peptide. As shown in Additional file 1: S2, the modified peptide shifted/moved drastically from its original position. It means modified peptide does not maintain original docking position so it will not affect the performance of docking methods in blind docking.

### Blind docking ability of methods

Blind docking of two structures is one of the major challenges in the field of docking. We used default parameters for blind docking and generated 20 docking poses for each complex. In order to compute the performance of a method on a protein-peptide complex, we compared its top 20 poses one by one with original docking pose. The average performance of different methods on the PPDbench dataset is shown in Table 2. The pose which is ranked first by the respective scoring function of method is termed as “Top pose” while the pose for which we obtain the lowest L-RMSD value among all the generated poses is termed as “Best pose”. In the case of top docking pose, FRODOCK performs better than any other method and achieve average L-RMSD of 12.46 Å. It was noted that the performance of different methods in term of I-RMSD and FNAT also follow the same trend. The performance of different methods on individual complexes is shown in Additional file 1: S3-S5. The performance of best docking poses out of top 20 poses generated by various methods, also shows the similar trend; FRODOCK with average L-RMSD of 3.72 Å performed better than other methods.

**Table 2:** The performance of best docking pose generated by different docking methods using blind docking on the PPDbench dataset

Docking methods	FNAT	L-RMSD	I-RMSD	% Success
ATTRACT-20^a^	66.51	6.16	6.12	54.13
ATTRACT-10	57.44	8.86	8.75	48.87
ATTRACT-5	53.01	10.38	10.23	42.10
ATTRACT-3	48.95	11.74	11.53	39.09
ATTRACT-1	40.86	15.59	15.30	34.58
Hex-20	30.92	25.73	25.64	18.04
Hex-10	26.62	27.85	27.82	17.29
Hex-5	21.33	30.37	30.31	15.03
Hex-3	17.88	31.77	31.70	12.78
Hex-1	13.06	35.64	35.55	10.52
ZDOCK-20	69.67	7.53	7.40	63.90
ZDOCK-10	61.79	9.42	9.27	53.38
ZDOCK-5	57.16	10.87	10.78	48.87
ZDOCK-3	54.14	11.97	11.84	42.85
ZDOCK-1	42.88	15.85	15.74	32.33
PatchDock-20	55.99	7.98	7.79	26.31
PatchDock-10	48.01	9.93	9.78	21.05
PatchDock-5	39.78	12.45	12.30	18.79
PatchDock-3	34.55	14.61	14.39	17.29
PatchDock-1	21.83	19.97	19.71	11.27
pepATTRACT-20	27.25	13.76	13.57	1.50
pepATTRACT-10	22.70	16.04	15.84	1.50
pepATTRACT-5	18.67	18.24	18.07	1.50
pepATTRACT-3	16.22	19.55	19.31	0.75
pepATTRACT-1	12.32	22.12	21.88	0.00
FRODOCK-20	71.44	3.72	3.69	55.63
FRODOCK-10	70.79	4.06	3.94	55.63
FRODOCK-5	64.16	6.04	5.92	50.37
FRODOCK-3	62.92	6.79	6.69	50.37
FRODOCK-1	48.40	12.46	12.21	39.09

^a^Number indicate number of top docking poses generated by method

### Re-docking ability of methods

Re-docking is preferred over blind docking if one knows the binding site of peptide/ligand on protein/receptor. Binding site information reduces searching space drastically; thus, re-docking is fast and more precise. In order to evaluate re-docking ability of methods, we perform re-docking on the PPDbench dataset. In this study, we used default parameters for re-docking using instructions provided by different docking method (See Material and Methods Section for detail). It is important to note that we used the original structure of the peptide in the case of re-docking instead of the modified structure since we are already providing information of binding site in the case of re-docking. We were unable to perform the re-docking experiment using FRODOCK, as there is no provision for re-docking in this software. Similarly, ATTRACT also didn’t perform re-docking, as the server does not take the input of active residue information. Thus, we performed re-docking only using 4 methods (ZDOCK, Hex, PatchDock, and pepATTRACT) and generated top 20 docking poses for each complex and compute the performance of the methods. We observed that the performance of all the docking methods improved during the re-docking study (Table 3). ZDOCK performed better than other docking methods for the top pose as well as for the best pose; followed by Hex. In the case of ZDOCK, L-RMSD improved from 15.85 Å to 8.60 Å for top docking pose and from 7.53 Å to 2.88 Å for the best pose. FNAT, L-RMSD and I-RMD values for all the individual complexes are given separately in Additional file 1: S6-S8.

**Table 3:** The performance best docking pose generated by different docking methods using re-docking on the PPDbench dataset

Docking methods	FNAT	L-RMSD	I-RMSD	% Success
Hex-20^a^	78.66	4.55	4.44	69.17
Hex-10	75.73	5.25	5.18	66.17
Hex-5	71.24	6.50	6.41	65.41
Hex-3	66.76	7.74	7.66	61.65
Hex-1	59.44	10.63	10.49	56.39
ZDOCK-20	89.61	2.88	2.81	85.71
ZDOCK-10	84.55	3.85	3.76	78.95
ZDOCK-5	80.77	5.14	5.04	75.19
ZDOCK-3	79.06	5.67	5.56	69.92
ZDOCK-1	68.44	8.60	8.44	55.64
PatchDock-20	63.14	5.14	5.05	32.33
PatchDock-10	55.64	6.73	6.63	29.32
PatchDock-5	48.30	8.22	8.08	24.81
PatchDock-3	43.25	9.46	9.27	23.31
PatchDock-1	29.03	13.36	13.13	18.80
pepATTRACT-20	39.07	8.21	8.07	3.76
pepATTRACT-10	34.82	9.17	9.02	2.26
pepATTRACT-5	30.32	10.03	9.83	2.26
pepATTRACT-3	27.20	10.87	10.70	2.26
pepATTRACT-1	21.11	12.32	12.07	1.50

^a^Number indicate number of top docking poses generated by method

### Ranking ability of methods

Ideally top pose assigned by a docking method should have the best performance, but in reality, it is not always correct. Thus, the docking method faces two major challenges, (i) how to generate the best docking poses and (ii) ranking these docking poses based on their performance. In order to rank docking poses, the different method uses different scoring functions. Thus, the scoring function plays a crucial role in ranking docking poses particularly in the identification of top/best pose out of many poses generated by a method. We tested the scoring function of all the 6 docking methods on the PPDbench dataset using blind docking. We analysed top 3, 5, 10 and 20 poses generated by the different software and computed performance of best poses (Table 2). The sequential improvement was observed with the increase of the number of selected poses in all the docking methods. However, Hex, ZDOCK, PatchDock and ATTRACT docking methods show a higher deviation in the results as clear from Table 2. The scoring function of FRODOCK seems better for docking studies of peptides compared to other docking methods. Percent of success rate in reproducing the docked poses within 2.0 Å L-RMSD with the original pose is also presented in Table 2. ZDOCK show the success rate of 32.33% on considering the top pose whereas FRODOCK shows a success rate of 39.09% Docking method pepATTRACT performed worst among all the docking methods. It takes the sequence of the peptide instead structure of the peptide. The method itself predicts the structure of the peptide from its sequence, and it is possible that predicted structure of the peptide is not correct.

In order to understand the limits of blind docking, for each complex we analyse top 20 docking poses generated by different methods. We identify best docking pose for a given peptide-complex generated by any method and compute the performance of best pose. This process is repeated for all complexes in PPDbench dataset, and average performance is computed. We achieved an average performance of 92.92% in term of FNAT, which is better than the performance achieved by any individual method. Similarly, we calculated the performance in term of L-RMSD and achieved an average L-RMSD value of 1.55 Å. This analysis shows that the combination of all 6 docking methods can dock almost all the peptides to their proteins with reasonably high accuracy (Additional file 1: S9-S10).

**Fig. 2:**
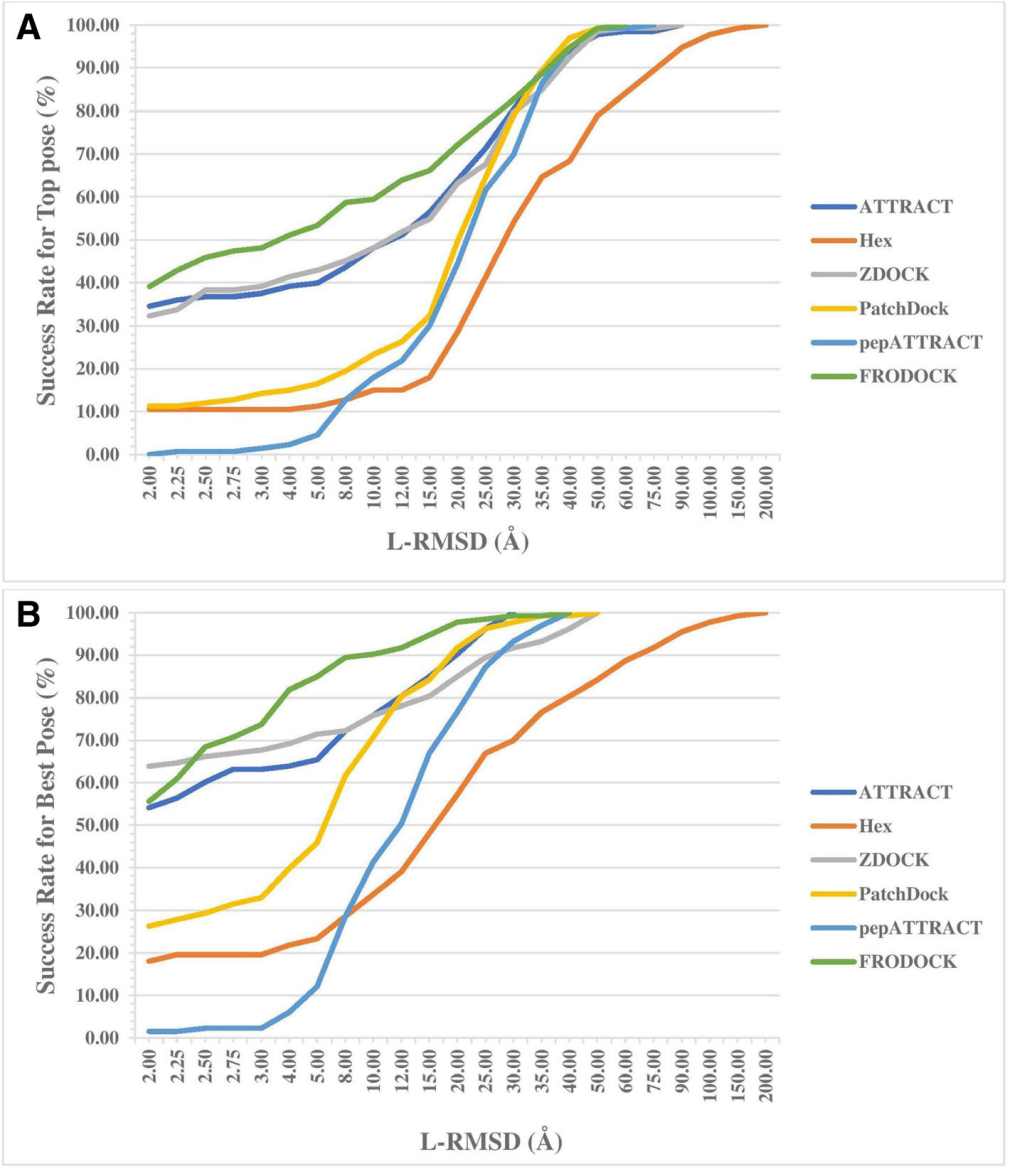
L-RMSD values obtained for all the docking methods on the PPDbench dataset for the (**a**) Top poses and (**b**) Best pose

Figure 2(a-b) shows the deviation in the success rate with the increase in the L-RMSD values. The success rate is calculated as the percentile of the success in obtaining the docked poses within the specified L-RMSD values. In our study, we observed that the success rate is not much affected by a slight increase in the L-RMSD for most of the considered docking methods. Considering the top 20 solutions, FRODOCK reproduced around 82.00% complexes within 4.0 Å L-RMSD. Thus, the performance of FRODOCK is much better as compared to other 5 docking methods. Figure 2(a-b) also shows the complete failure of docking of all the docking methods (more than 30 Å L-RMSD) for some of the complexes. It is clear from Additional file 1 S11, deviation in the success rate with the increase in the I-RMSD values follow the same trend as the deviation in the success rate with the increase in L-RSMD values. Any successful scoring function should either produce the top score pose as the best pose or should show the minimum deviation between these two poses. In order to understand the difference between top pose and best pose generated by each method, we calculate the percent of complexes having L-RMSD (difference in best and top pose) in a different range (Table 4). In most of the cases (~ 56.00%), top and best pose generated by FRODOCK have no difference with L-RMSD 0.00. As shown in Table 4, the scoring function of FRODOCK, ATTRACT and ZDOCK are better than other methods.

**Table 4:** Percentile of success rate where ‘Top pose’ and ‘Best pose’ are same or within the range of some specified differences

(Best – Top) RMSD (Å)	ATTRACT	Hex	ZDOCK	PatchDock	pepATTRACT	FRODOCK
0.00	24.81	14.28	24.81	18.04	12.03	55.63
0.00–1.00	18.79	0.75	14.28	1.50	12.03	0.75
1.00–2.00	0.75	9.77	6.76	3.00	8.27	0.75
2.00–5.00	11.27	15.78	14.28	9.77	16.54	5.26
5.00–10.00	10.52	16.54	10.52	13.53	13.53	5.26
> 10.00	33.83	42.85	29.32	54.13	37.59	32.33

### Reproducibility of docking poses

Ideally, docking pose generated by a method and ranking of docking poses should be same every time for a given protein-peptide complex. In order to check reproducibility, we generate docking pose using blind docking for each complex two times called “First docking” and “Second Docking” for each docking method. Then we compute the performance of each method for “First Docking” and “Second Docking” as well as the difference in the performance. As shown in Table 5, for most of the methods, the difference was either zero or negligible. It means the results of docking methods are reproducible (Table 5).

**Table 5:** The performance of best docking pose generated by different methods and difference in performance of docking poses generated in two events on the PPDbench dataset

Docking Method	First Docking			Second Docking			Difference (First-Second)		
	FNAT	L-RMSD	I-RMSD	FNAT	L-RMSD	I-RMSD	FNAT	L-RMSD	I-RMSD
ATTRACT-20^a^	66.51	6.16	6.12	66.51	6.16	6.12	0.00	0.00	0.00
ATTRACT-1	40.86	15.59	15.30	40.86	15.59	15.30	0.00	0.00	0.00
Hex-20	30.92	25.73	25.64	30.60	25.77	25.72	0.32	0.04	0.08
Hex-1	13.06	35.64	35.55	12.33	35.39	35.38	0.73	0.25	0.17
ZDOCK-20	69.67	7.53	7.40	69.68	7.53	7.40	0.01	0.00	0.00
ZDOCK-1	42.88	15.85	15.74	42.86	15.85	15.74	0.02	0.00	0.00
PatchDock-20	55.99	7.98	7.79	56.00	7.98	7.79	0.01	0.00	0.00
PatchDock-1	21.83	19.97	19.71	21.87	19.97	19.71	0.04	0.00	0.00
pepATTRACT-20	27.25	13.76	13.57	27.26	13.76	13.57	0.01	0.00	0.00
pepATTRACT-1	12.32	22.12	21.88	12.33	22.12	21.88	0.01	0.00	0.00
FRODOCK-20	71.44	3.72	3.69	71.44	3.72	3.69	0.00	0.03	0.00
FRODOCK-1	48.40	12.46	12.21	48.39	12.46	12.21	0.01	0.00	0.00

^a^Number indicate number of top docking poses generated by method

### Molecular analysis

#### Resolution analysis

**Fig. 3:**
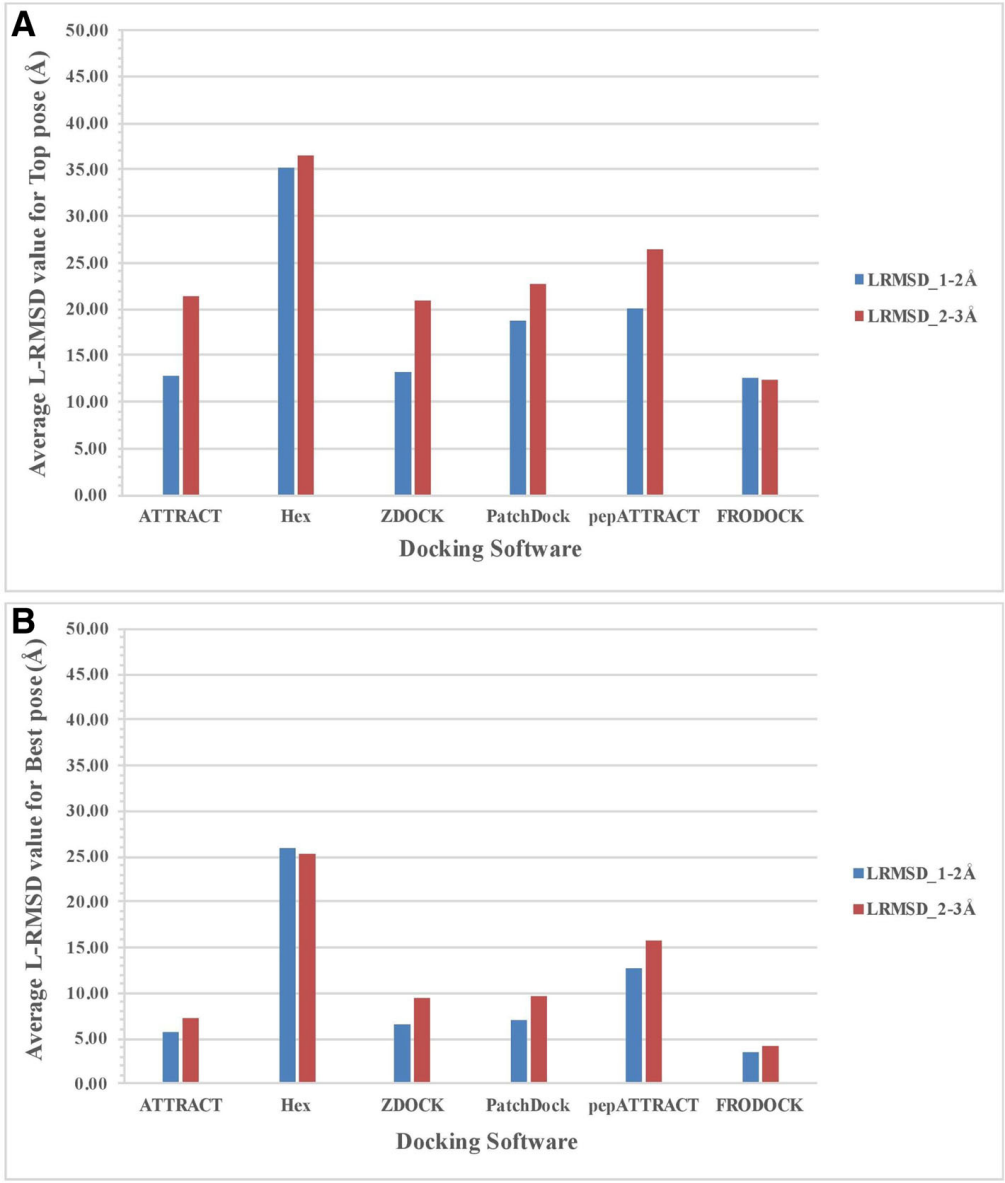
The performance of different docking methods on the PPDbench dataset with resolution 1–2 Å and 2–3 Å for (**a**) Top pose and (**b**) Best pose based on average L-RMSD value

The quality of structure of a complex depends on the resolution of the crystal structure. The quality of benchmarking depends on the quality of the complex structure as it is used as a standard of truth or reference for measuring quality docking pose. Thus, we divide protein-peptide complexes into two groups having the resolution between 1 and 2 Å and between 2 and 3 Å. Then we compute performance in term of L-RMSD of all methods using blind docking for above two group of complexes. In case of top pose, the performance of all method except FRODOCK improves for complexes having resolution 1–2 Å (Figure 3(a)). In case of best pose, the performance of all methods except Hex improves for complexes having resolution 1–2 Å than complexes having resolution 2–3 Å (Figure 3(b)). This is expected as the quality of evaluation also depend on the resolution of complex structures. Average value of FNAT and I-RMSD was also analysed for the top pose (Additional file 2:(a-b)) and best pose (Additional file 3:(a-b)).

#### Rotatable bonds

We group protein-peptide complexes based on rotatable bonds in the peptide to understand the effect of rotatable bonds on quality of docking pose generated by different methods (Tables 6, 7, 8). Considering average L-RMSD, we found the different behaviour of software in docking results with respect to the number of rotatable bonds. When the top pose was considered, FRODOCK and ATTRACT were found to perform better for complexes either having a smaller number of rotatable bonds or larger bonds while other software showed a decline in the performance with the increasing number of rotatable bonds. In case of best pose, the situation was a little different. ZDOCK and FRODOCK were found to perform better than other software (Tables 6, 7, 8).

**Table 6:** The performance of best docking poses having different number of rotatable bonds in term of FNAT; poses generated by different methods on the PPDbench dataset

Docking methods	Rotatable bonds		
	0–40	41–60	> 60
ATTRACT-20^a^	62.90	63.85	79.91
ATTRACT-1	40.93	38.55	48.25
Hex-20	31.96	29.78	33.08
Hex-1	08.12	15.06	13.58
ZDOCK-20	78.51	65.30	71.41
ZDOCK-1	41.15	42.98	44.83
PatchDock-20	55.09	59.25	46.95
PatchDock-1	27.00	22.02	14.37
pepATTRACT-20	43.60	21.69	22.41
pepATTRACT-1	22.03	09.26	08.70
FRODOCK-20	73.42	69.47	75.08
FRODOCK-1	49.78	46.17	53.75

^a^Number indicate number of top docking poses generated by method

**Table 7:** The performance of best docking poses having different number of rotatable bonds in term of L-RMSD; poses generated by different methods on the PPDbench dataset

Docking methods	0–40	41–60	> 60
ATTRACT-20^a^	6.00	6.76	4.49
ATTRACT-1	11.78	17.03	16.21
Hex-20	23.55	26.07	27.63
Hex-1	33.56	35.72	38.21
ZDOCK-20	4.76	8.26	8.98
ZDOCK-1	11.96	16.97	17.62
PatchDock-20	9.16	7.28	8.51
PatchDock-1	16.54	20.16	24.05
pepATTRACT-20	9.44	15.61	13.81
pepATTRACT-1	16.64	22.80	27.48
FRODOCK-20	2.75	4.28	3.25
FRODOCK-1	10.25	13.97	10.70

^a^Number indicate number of top docking poses generated by method

**Table 8:** The performance of best docking poses having different number of rotatable bonds in term of I-RMSD; poses generated by different methods on the PPDbench dataset

Docking methods	0–40	41–60	> 60
ATTRACT-20^a^	5.99	6.73	4.42
ATTRACT-1	11.33	16.77	16.11
Hex-20	23.35	25.99	27.66
Hex-1	33.42	35.65	38.13
ZDOCK-20	4.74	8.09	8.85
ZDOCK-1	11.73	16.92	17.49
PatchDock-20	8.93	7.09	8.39
PatchDock-1	16.32	19.84	23.93
pepATTRACT-20	9.31	15.34	13.79
pepATTRACT-1	16.49	22.47	27.40
FRODOCK-20	2.73	4.22	3.29
FRODOCK-1	9.97	13.70	10.53

^a^Number indicate number of top docking poses generated by method

#### Secondary structure analysis

**Fig. 4:**
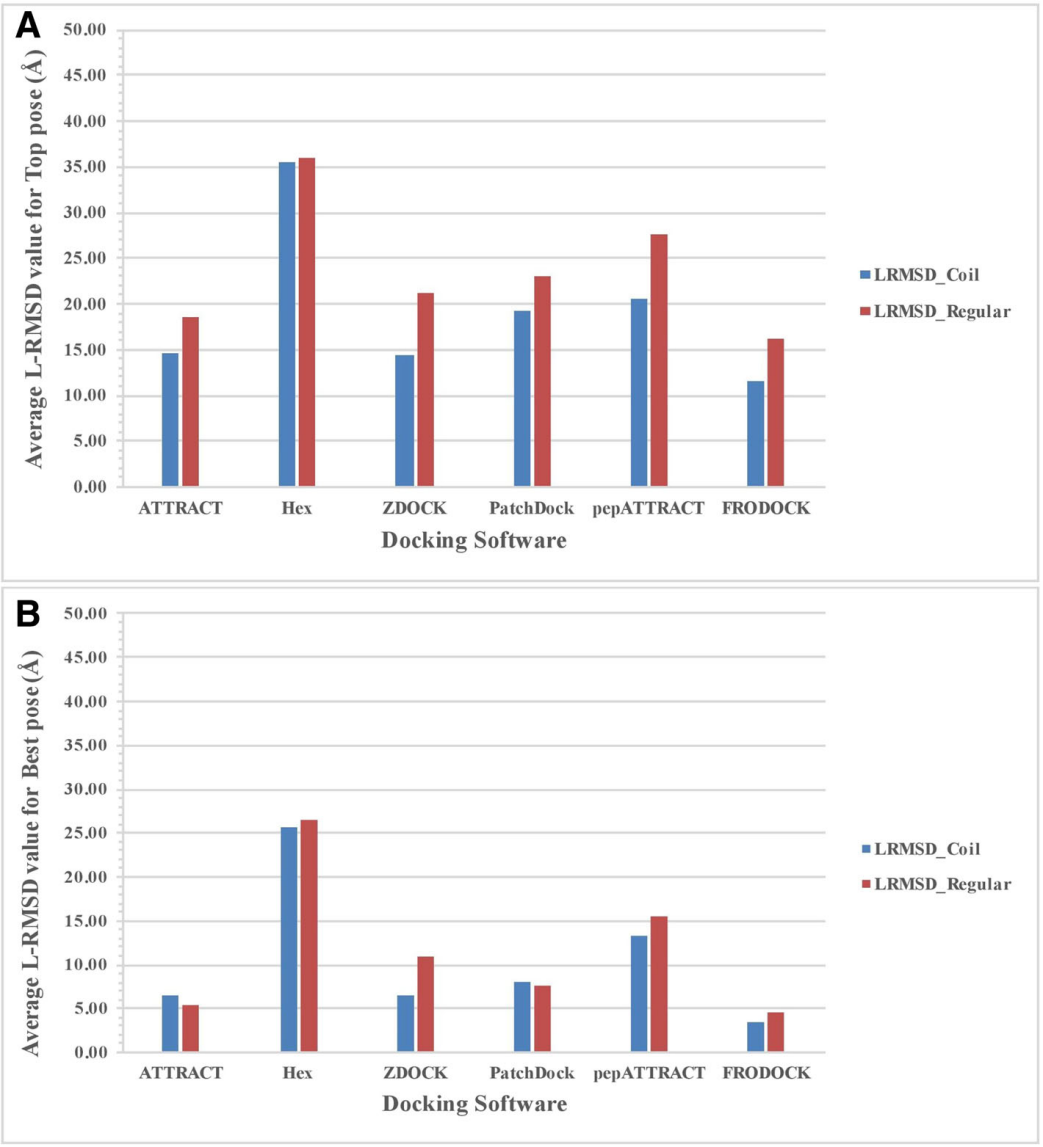
The performance of different docking methods on the PPDbench dataset with different secondary structure for (**a**) Top pose and (**b**) Best pose based on average L-RMSD value

We divide protein-peptide complexes into two groups based on the secondary structure of the peptides. The first group contains peptides dominated by regular secondary structure (e.g., Helix, Sheet). The second group is dominated by the peptide structure comprising coils. We compute performance of the docking methods using blind docking on both groups of complexes separately. The performance in term of L-RMSD for two group is shown in Figure 4. In case of top pose, all methods except Hex performed better on protein-peptide complex dominated by the coil in comparison (Figure 4(a)). A similar trend was observed for best docking pose, where most of the method perform better for peptides dominated by the coils (Figure 4(b)). Average value of FNAT and I-RMSD was also analysed for the top pose (Additional file 4(a-b)) and best pose (Additional file 5(a-b)). This analysis indicates that docking peptides having regular secondary structure is more difficult than peptides having no regular secondary structure. One of the possible explanation for this result could be the flexible nature and high degree of freedom of coiled peptides in comparison to helical peptides which is more rigid and possess a lesser degree of freedom. Coiled peptide might adapt better conformational change during docking to find the near-native pose. Also, in previous studies, it has shown that the formation of coiled-coil in peptide help in better docking with the receptor molecule [68, 69]. Similarly, in another study, it was shown that the docking method performed better on coiled peptides [70].

#### Categorization of PPDbench dataset and the performance of the method

When we analysed the classification of the PPDbench dataset (Table 1), we found that most of the complexes belong to the enzymatic class, for example, hydrolase, ligase, oxidoreductase, transferase (around 27%), transcription (~ 17%) or to signaling proteins (~ 12%). Rest of the complexes were present in other classes like structural proteins, membrane proteins, binding proteins, etc. Overall, a good amount of diversity was present in the dataset. Based on the blind docking result and considering the top pose with lowest L-RMSD value, we analysed the group of complexes preferred by each software (Table 9). FRODOCK performed best on 42 complexes out of 133 which belongs majorly to Transcription and Signaling protein class. ATTRACT achieved 2nd position by performing best on 35 complexes where most of them belong to the Enzymatic class, Immune system class or Signaling protein class. ZDOCK performed best on 30 complexes, and during analysis, we didn’t find any specific class which is preferred over another. Likewise, PatchDock performed best on 15 complexes in total, and the class diversity was a mix. However, it didn’t cover complexes belonging to Signaling proteins or Structural proteins class. Hex showed lowest L-RMSD only for 8 complexes, belonging to class Signaling proteins or Transcription and lastly, pepATTRACT was found best only on 3 complexes.

**Table 9:** Clustering result of docking method showing the PDB IDs for which it performed best during blind docking when top pose was considered

Sr. No.	Docking Method	Number of Complexes^a^	PDB ID
1	ZDOCK	30	1CJR, 1CKA, 1H6W, 1YMT, 2AQ9, 2CCH, 2D0N, 2DRK, 2FTS, 2HO2, 2P1T, 2VWF, 2WHX, 2XU7, 2ZJD, 3G2S, 3H1Z, 3KMR, 3KUS, 3V2X, 3VTC, 4F1Z, 4J8S, 4K0U, 1RST, 2DYP, 3ASL, 3I5R, 3IVV, 3LL8,
2	ATTRACT	35	1D4T, 1JBU, 1NLN, 1PZL, 1QKZ, 1RXZ, 1SFI, 1SSH, 1UJ0, 2A3I, 2B9H, 2BBA, 2FKA, 2HT9, 2O4J, 2PUY, 2V8Y, 3AYU, 3BFQ, 3D32, 3KUJ, 3OLF, 3P8F, 3QIS, 3SFJ, 3SO6, 4E34, 4EIK, 4ERY, 4F14, 4GXL, 4H4F, 2 CE8, 2VKN, 3ERY
3	FRODOCK	42	1MFG, 1NTV, 1NX1, 1OJ5, 1OW6, 1 T08, 1T4F, 1T7R, 1TFC, 1 U00, 1YUC, 1YWO, 2FFF, 2FMF, 2O9V, 2P0W, 2P1O, 2P54, 2PEH, 2PUX, 2QOS, 2QSE, 2R7G, 2W2U, 2XRW, 2XVC, 3C3R, 3FDO, 3GYT, 3L0E, 3TZY, 3UP3, 3W1B, 4GYW, 4HTP, 1K5N, 1OU8, 2FFU, 2R9Q, 3OBQ, 3RM1, 3TJV
4	PatchDock	15	1HC9, 1X2R, 1XOC, 2O02, 2VR3, 3DS4, 3P72, 3PTL, 3RQG, 4B4N, 4DCB, 4GQ6, 4HOM, 1CVU, 2A25
5	Hex	8	1EG4, 1NQ7, 2FVJ, 2QBX, 3AWR, 3LLZ, 4IIM, 3U9Q
6	pepATTRACT	3	1OAI, 3ZQH, 2OEI

^a^Number of complexes for which software performed best

### Performance on benchmarking data used in previous studies

Rentzsch and Renard evaluated the performance of AutoDock Vina on a dataset of 47 protein-peptide complexes, where the length of peptide varies from 2 to 5. Rentzsch and Renard also generated 20 docking poses and compute performance of top pose as well as the performance of best docking pose. We also evaluated our methods on this dataset; unfortunately, few methods fail on certain PDB IDs, or there were certain issues due to which we didn’t involve them in our study. For example, pepATTRACT failed on PDB files 1PAU, 8GCH, 1JQ9 and 5SGA, ATTRACT failed on IDs 1PAU and 1BE9, FRODOCK failed on 1PAU and 5SGA. After analysing these files, we found that one potential reason for the failure of different docking methods is the presence of non-natural residues (example ACE, ACY) in the peptide of these complexes. Likewise, we didn’t get any contacts for the IDs 1BHX and 3TPI as per our criteria (mentioned in Material and Methods) during re-docking studies. Therefore, we excluded these 7 IDs and proceeded with 40 peptide-protein complexes instead of 47 for benchmarking methods used in this study. We referred this dataset (40 protein-peptide complex structures) as Vina dataset. We performed blind docking using 7 docking methods including AutoDock Vina and evaluate the performance of all methods (Table 10). In the case of top docking pose, PatchDock got the average L-RMSD value of 12.21 Å; performing better than other methods. Similarly, FRODOCK achieved an average L-RMSD value of 4.45 Å in case of best docking pose; performing better than other methods. We also evaluate the re-docking ability of four docking methods only as some of the methods do not have provision for re-docking. In the case of re-docking, we have not evaluated the re-docking ability of AutoDock Vina instead we have taken performance reported by authors for these complexes. As shown in Table 10, AutoDock Vina performed better than other methods. The performance of different docking methods in detail is described in Additional file 1: S12-S13.

**Table 10:** The performance of different docking methods on Vina dataset (40 protein-peptide complexes) in terms of average L-RMSD

Docking methods	Blind docking	Re-docking
Autodock Vina-20^a^	9.80	2.09
Autodock Vina-1	17.13	4.25
Hex-20	23.70	3.50
Hex-1	30.16	9.98
ZDOCK-20	11.16	11.48
ZDOCK-1	17.76	18.25
PatchDock-20	4.54	3.49
PatchDock-1	12.21	8.96
pepATTRACT-20	7.28	4.31
pepATTRACT-1	13.57	7.89
FRODOCK-20	4.45	NA^#^
FRODOCK-1	13.77	NA
ATTRACT-20	8.13	NA
ATTRACT-1	14.43	NA

^a^Number indicate number of top docking poses generated by method; #NA re-docking provision is not available

Recently, Hauser and Windshugel used LEADS-PEP dataset for benchmarked re-docking ability of four different protein-ligand docking software AutoDock, AutoDock Vina, Surflex, and GOLD. We compare three datasets (LEADS-PEP dataset, with our dataset of 133 complexes and 40 complexes of dataset created by Rentzsch and Renard) and found 10 common protein-peptide complexes (8 complexes from the PPDbench dataset and 2 complexes from Vina dataset). On these 10 common complexes, we evaluate the performance of top and best docking pose generated by docking methods used in our study. We have taken the performance of other methods as reported by their authors (Table 11). Z-DOCK achieved an average L-RMSD value of 4.87 Å and 2.14 Å for the top and best docking pose respectively, which is better than other methods.

**Table 11:** The performance of different docking methods on common 10-protein-peptide complexes, in term of their re-docking ability

Docking Method	L-RMSD	
	Top pose	Best pose
AutoDock	8.31	5.77
Autodock Vina	8.37	4.57
Surflex-Dock	8.02	4.96
GOLD (ASP)^a^	8.52	4.65
GOLD (CP)^a^	9.59	5.51
GOLD (CS)^a^	9.86	4.99
GOLD (GS)^a^	6.39	3.95
Hex	12.27	9.55
ZDOCK	4.87	2.14
PatchDock	11.98	4.22
pepATTRACT	13.07	7.66

^a^ASP, CP, CS and GS are different scoring functions

### Platform for benchmarking

In order to facilitate the scientific community, we developed a web server or portal called PPDbench for benchmarking docking methods. This web server consists of following modules: (i) Single: This module is developed to provide a tool to calculate FNAT, I-RMSD and L-RMSD values for a single docked protein-peptide complex. (ii) Batch: This module is designed to calculate the above parameters for more than one complex. The user needs to submit the original and their respective docked poses separately in zip format for batch mode. A complete dataset used in this study with all the receptors, ligands and their respective complexes are available in the web server for downloading. PPDbench web service is freely accessible at http://webs.iiitd.edu.in/raghava/ppdbench/.

## Conclusions

In this study, we selected 133 protein-peptide complexes to rigorously validate the applicability of 6 widely used docking methods for studying protein-peptide interactions. We generated 20 docked poses for each protein-peptide complex using different docking methods to evaluate their performance. The study shows that in the case of blind docking FRODOCK performed best among all methods whereas, in the case of re-docking, ZDOCK performed best. One of the possible explanation for this result is that ZDOCK is a FFT based docking algorithm and its scoring functions comprise of pairwise shape complementarity with desolvation and electrostatics. When binding site information is not present the method performs a complete search over the protein surface for locating the ligand binding site. However, when the binding site information is known, it restricts the search to the complementarity region and hence probability of obtaining near-native pose is much higher. This could be one of the reasons why ZDOCK performs better in re-docking in comparison to blind docking. However, FRODOCK is an initial stage rigid body docking algorithm which optimizes different interactions such as van der Waals interactions, electrostatic interaction and desolvation potentials using a new fast rotational algorithm based on spherical harmonics (SH). In the previous studies, SH has been shown to enhance the docking efficiency [65, 71] and inspired by these studies, FRODOCK implemented the same in their algorithm for enhancing the docking efficiency. This approach increased the searching by accelerating the 3 rotational degrees of freedom. This novel approach could be one of the main reason for the better performance of FRODOCK over the other methods in case of blind docking. Also, the procedural differences (such as the use of search constraints, the definition of interface residues in the evaluation, statistical differences in the number of runs and different sampling sizes) and different approximations probably facilitates FRODOCK to provide better results compared to ZDOCK in blind docking [35]. Therefore, based on our study we proposed FRODOCK for performing blind docking and ZDOCK for re-docking.

It was observed that most of the docking method fails to rank their docking pose successfully, as the performance of their best pose is much better than the top pose (Table 2). Thus, there is a need to develop new scoring functions which can rank docking poses with high precision. We combine docking pose generated by different methods for a protein-peptide complex and compute performance of best pose. It has been observed that the performance of best pose obtained from different methods collectively is much better than the performance of pose generated by any individual method. This observation suggests the need for a universal scoring function that can rank pose generated by any method. Our study also suggests the utilization of high-resolution protein-peptide complex structures for benchmarking. In order to facilitate scientific community, we develop a web-based platform that provides benchmarking dataset as well as tools to evaluate the performance of docking poses.

## Material and methods

### Dataset for benchmarking

We created a dataset of 133 protein-peptide complexes by combining peptiDB dataset and ACCLUSTER dataset. The peptiDB [72] and ACCLUSTER [73] datasets consist of 103 and 251 protein-peptide complexes respectively. We removed all those complexes having more than 1 protein chain or where the length of the peptide was less than 9 or more than 15 residues. We also removed complexes containing any modified residue and complexes corresponding to obsolete PDB entry. After applying the above filters, we were left with 44 protein-peptide complexes from the peptiDB dataset and 115 from ACCLUSTER dataset. We combined both the datasets and selected unique protein-peptide complexes since there were some common PDB IDs in both the datasets. Finally, we were left with unique 133 protein-peptide complexes, and this dataset is referred as PPDbench or main dataset. The detail information of all the selected complexes is given in Table 1. We processed the proteins and peptide chains before docking. All heteroatoms (for example metal ions, water molecules) were removed, if an atom has alternative locations (coordinates) then the only ‘A’ coordinates were considered, and rest were removed, missing atoms (if any) in the PDB file were modeled and completed using Modeller software [74]. We also calculated redundancy among the 133 proteins using CD-HIT software [75]. The redundancy was calculated at 40% sequence similarity at default parameters (−c = 0.4, −*n* = 3, and -M = 400) since it is a well-established standard approach [76, 77].

Complexes present in the PPDbench or main dataset contain long peptides with the number of residues between 9 to 15 residues, in contrast to previous studies where small peptides were used. To provide a comprehensive picture, we also evaluated the performance of the above docking methods on protein-peptide complexes used in previous studies. The first dataset is of 47 protein-peptide complexes having peptide length up to 5 residues, used in a study by Rentzsch and Renard. In this study, authors evaluated the performance of AutoDock Vina on a meta-data set of 47 protein-peptide complexes based on existing 11 publications, with peptide length up to 5 residues. Re-docking and Semi-blind docking were performed with the maximum number of poses set to 20, energy range to 10 and exhaustiveness level-up to 1024. At the end of the study, authors concluded that increased sampling made the result more reproducible and improved the primary rigid docking result however no change in the case of flexible docking was observed. Also, there was no correlation present in between the ranked pose and the native pose. The overall performance of AutoDock Vina performance was case dependent and poor for peptides with more than 4 residues [55]. However, in our study, we used only 40 complexes out of 47 which are suitable for docking (reason for excluding 7 complexes is explained in the results section). We referred this dataset as Vina dataset.

The other dataset was the LEADS-PEP dataset, used for benchmarking different docking methods in a study by Hauser and Windshugel [56]. The dataset comprises 53 protein-peptide complexes with peptide length 3–12. Authors evaluated the performance of four docking methods namely AutoDock, AutoDock Vina, Surflex and GOLD with different scoring functions. The result showed that all the software were able to reproduce conformations of the peptides up to 4 residues. However, performance declined with increasing peptide length. Author also concluded that implementing the scoring function, the performance of the methods improved in identifying near-native pose. Overall the performance of GOLD:ASP in combination with CS rescoring was the method of choice in this study.

### Shifting Cartesian coordinates of peptides for blind docking

In order to evaluate blind docking ability of a docking method, one should not provide any information related to peptide binding site. The Cartesian coordinate of peptide structure already has information of binding site since we have taken the peptide from the protein-peptide complex. Thus, it is important to change the Cartesian coordinates of a peptide without changing the structure of the peptides. In this study, we converted the Cartesian coordinates to Internal coordinates and back from Internal to Cartesian coordinates using the following approach. The Cartesian coordinates of 133 peptides were converted to internal coordinates using “dihed.pl”, a perl script of MMTSB toolkit [78]. All dihedral angles of ligand were calculated and using these dihedral angles, the structure of the ligands was reconstructed using “tleap” module of AMBER [79]. In this way, original information of peptide coordinates is lost in the new structure.

### Re-docking on PPDbench dataset

Re-docking is preferred over blind docking if one knows the binding site of peptide/ligand on protein/receptor [80]. We also performed re-docking to generate docking pose using different methods on the PPDbench dataset of 133 protein-peptide complexes. In order to perform re-docking, we obtained information about all interacting residues, which were in contact with any of the peptide heavy atom within range of 5 Å using the script from pdbtools [81]. This binding site information was provided to all docking methods for performing re-docking. In the case of re-docking, we used original peptides instead of a modified structure with shifted coordinates.

### Docking protocol

Detail description of all the 6 docking methods along with the parameters used for running the experiment is given below.

#### Attract

The method is based on the randomized search algorithm. It performs a systematic docking based on energy minimization of the protein in the translational and rotational degree of freedom. This docking approach adopts the protocol where each amino acid of a protein is represented by up to three pseudo-atoms. This reduction of protein helps in faster energy minimization and also helps in finding docking energy minima on the protein surfaces. Scoring function used in this program is Lennard-Jones type effective potentials and electrostatics. It is relatively simple and distinguishes between hydrophobic and hydrophilic side chains. Here, residue-residue potential has been used which ranks amount of surface complementarity, hydrophilic or hydrophobic nature of contracting protein regions during docking experiment. This docking approach was built to provide fast docking method that can account for side-chain conformational flexibility. The program is written in Python and C++ and is part of the object-oriented PTools library. This library consists of several routines to manipulate the structure of proteins, to prepare and perform docking and to analyze the results obtained after docking**.**

For blind docking, we uploaded the receptor and ligand file onto the “Partners” tab of the server with Generate Harmonic Mode and RMSD calculation option off. No files were loaded in the “Sampling” tab. In “Energy and Interaction” tab, grid-accelerated docking option was checked but not the iATTRACT refinement [82]. The number of poses was set to 20 in “Analysis” tab and lastly in computation 1 processor core was used. In ATTRACT we can provide grid size but in our case software itself calculated it. After providing this information, we download the ready-to-use script. The script was further run on the local machinery. ATTRACT performs 1000 minimization steps on each starting structure by default since the grid option was provided. No provision of re-docking is available in the ATTRACT, and hence re-docking study was not performed.

#### Hex 8.0.0

Hex 8.0.0 is another popular and widely used method for protein-protein docking. This program uses Spherical Polar Fourier (SPF) correlations rather than Fast Fourier Transform (FFT) based search. In SPF search, 5 rotational and 1 translational degree of freedom is present which reduces the execution time to few minutes whereas in FFT based search 3 rotational and 3 translational degrees of freedom is there. This SPF algorithm of Hex has been validated in CAPRI blind docking experiment [34]. Hex uses the strategy of densely sampling the search space and then cluster the solutions showing similar orientation. In its scoring scheme, Hex calculates shape complementarity excluded volume with an optimal in vacuo electrostatic contribution. The Hex docking algorithm has also been implemented in the form of a web server known as HexServer. This server takes PDB files as an input and provides high-quality docking predictions for further refinement. In recep.mac file (which is a macros file) we set the docking receptor samples to 492, docking ligand sample to 492, docking alpha samples to 128, receptor range angle to 30, Ligand range angle to 30, twist range angle to 30, R12 range as 31, R12 step to 0.75, grid size to 0.6, docking main scan to 16 and docking main search to 25. These values are macros value, and we have taken it from the Hex manual pdf. These values were common for both blind and re-docking. The only difference was that in blind docking we used ‘nopos’ option in the script file, whereas during re-docking we used ‘pos’ option because this option position ligand near receptor during docking.

#### PatchDock 1.0

PatchDock 1.0 is a molecular docking method, which is based on shape complementarity theory. The algorithm of PatchDock is inspired by the technique of object recognition and image segmentation. Surfaces of the two given molecules are divided into different patches on the basis of their shape. These patches are matched with the corresponding generated patterns. Once the identification of patches is completed, they are mapped using the shape-matching algorithm. The patches identified retained the “hot spot” residues. For surface-patch mapping, PatchDock implies hybrid of the Geometric Hashing and Pose-Clustering machine algorithms. Complexes are ranked according to their geometric shape complementarity score. PatchDock algorithm is available as a web server for docking. Here in this study, option “drug” was given as complex type during blind docking with the default clustering RMSD parameter of 4 Å. The ‘drug’ option is given when docking is performed for the small molecules like peptide, drug, etc. However, in the case of re-docking, we provided additional information about residues involved in receptor active site which was used during docking.

#### ZDOCK3.0.2

ZDOCK3.0.2 is one of the widely used protein-protein docking method developed at Weng lab and is based on rigid-body Fast Fourier Transform docking algorithm. The scoring function of ZDOCK is a combination of pair-wise shape complementarity (PSC) with desolvation (DE) and electrostatics (ELEC) where desolvation is the main component of the ZDOCK’s competitive function. The algorithm performs the global search over the protein rotational and translational space in the absence of binding site information. Chen and Weng benchmarked these scoring functions individually and in combination and showed that the overall combination of PSC + DE + ELEC performed best and is responsible for the better performance of ZDOCK. ZDOCK web server has also been developed for the docking purpose to help researchers.

In case of blind docking, receptor and ligand file were processed using “mark_sur” and “uniCHARMM” binary files as mentioned in the README file of the software. ZDOCK generate the grid size according to the size of the protein. The default spacing between the grid cells was constant of 1.2 Å, and by default, the receptor was fixed during docking with the initial rotation of the ligand with Euler angle 0. However, during the re-docking study, we blocked those residues which were not present in the receptor active site using perl script ‘block.pl’ given in the software along with the above-mentioned default parameters.

#### pepATTRACT

pepATTRACT is a recently developed docking approach specifically for docking peptide and protein. This program is a part of ATTRACT and is quite flexible in nature. pepATTRACT takes the sequence of the peptide as an input and generates its own peptide models. pepATTRACT performs rapid coarse-grained ab-initio global docking onto protein surfaces. This docking method selects top models on the basis of ATTRACT ranking score and performs atomistic refinement using iATTRACT [82]. After the refinement, top 1000 models are selected and refined using molecular dynamics simulations with AMBER14. Finally, models are clustered using the fraction of common residue contacts and are ranked on the basis of the average energy value of the top 4 ranking members.

In case of blind docking, receptor and ligand file were uploaded onto the “Partners” tab of the server with RMSD calculation option off. In the case of pepATTRACT, no “Sampling” tab is there. In “Energy and Interaction” tab, grid-accelerated docking and iATTRACT refinement options were checked. Number of poses were set to 20 in “Analysis” tab and lastly in computation 1 processor core was used. It performs 1000 minimization steps on each starting structure by default since grid option was provided. After providing this information, we download the ready-to-use script with the above-mentioned information. The script was further run on the local machinery. In case of re-docking, in addition to the above-mentioned parameters, we provide the list of active residues directly involved in the interaction in the Protein section of Partner tab.

#### FRODOCK 2.0

FRODOCK is one of the popular and widely used servers for protein-protein docking. It was ranked 4th among the 18 docking/scoring function tested. Earlier version of FRODOCK was based on the principle of 3D grid-based potentials with spherical harmonics (SH) properties. However, recently developed version FRODOCK 2.0 includes an extra knowledge-based potential, which helps in improving docking success rate more significantly. It combines 3 binding energy van der Waals, desolvation, and electrostatics interaction and optimizes it by using new fast rotational docking algorithm based on spherical harmonics which is coupled with systematic translational search. The scoring function was improved by adding complementary coarse-grained knowledge-based protein-protein docking potential [83]. FRODOCK allows the user to predict protein-protein complexes using unbound components in a few minutes. Scoring function in the server has been optimized for 3 different types of interactions, i.e. enzyme-substrate, antigen-antibody and others.

Web server was used in the case of FRODOCK for performing docking studies. Files were uploaded onto the server with the type of interaction “Unknown” which is the default case. Web server methodology section tells that in case of FRODOCK electrostatic contribution is calculated in the range of ±10 Kcal/mol e. buried surface area of receptor and ligand is calculated using a generic probe of radius 1.7 Å at the grid points near to the corresponding surface. In the case of FRODOCK, there is no provision of re-docking. Therefore, only blind docking study was performed using this method.

### Performance evaluation parameters

In order to measure the performance of docking poses generated by the different method, we used parameters namely FNAT, I-RMSD, and L-RMSD adopted in worldwide competition CAPRI (Critical Assessment of PRedicted Interactions). Mendez et al. proposed “A pair of residues on different sides of the interface was considered to be in contact if any of their atoms were within 5Å” [84, 85]. FNAT is the fraction of correct (native) residue-residue contact in predicted complex divided by residue–residue contacts in the original complex. We computed L-RMSD and I-RMSD for measuring the overall geometric fit between the original complex and predicted complex tertiary structure. L-RMSD is the backbone root-mean-square deviation of the ligands in the original and predicted complexes based on superpositioning of backbone atoms. I-RMSD is the root-mean-square deviation of the backbone atoms of the interface (contact) residues in the original and predicted complexes. In our study, Ptools [86] (an open source molecular docking library) is used for calculating FNAT and I-RMSD values and PyMol for calculating L-RMSD values.

### Grouping of complexes for molecular analysis

We divided our dataset into two categories based on the resolution of the structure. In the first group, we put all those structures whose resolution was in between 1 and 2 Å (89 complexes out of 133 complexes), and the other group consists of structures whose resolution was in between 2 and 3 Å (44 complexes out of 133 complexes). We evaluated the performance of docking methods each group of complexes. We calculated the number of rotatable bonds present in the peptides using PADEL software [87]. All 133 complexes were divided into three groups based on the number of rotatable bonds present in the peptides (i) peptides having a number of rotatable bonds in between 0 and 40 (ii) peptides having a number of rotatable bonds in between 41 and 60 and (iii) peptides having a number of rotatable bonds above 60.

Similarly, we group complexes based secondary structure of peptides. We assign secondary structure of all peptides in complexes using DSSP software [88, 89]. We made two groups of protein-peptide complexes (i) regular secondary structure and (ii) coil. Regular secondary structure was assigned to that complex whose sum of helix and sheet content is 60% or more. Rest of the complexes were assigned to coil category. We also analysed the class of 133 complexes present in PPDbench dataset to observe the class preference of different docking methods. For this, we considered blind docking result obtained for the top pose. Method which showed the lowest L-RMSD value for the complex was considered best for that particular complex.

## Additional files

Additional file 1: **S1.** Sequence similarity at 40% between the proteins of PPDbench dataset using CD-HIT software. **S2.** Distance between the original peptides and the peptides with changed coordinates. **S3 (a-f).** FNAT values of 133 complexes obtained for all 6 docking methods by blind docking. **S4 (a-f).** L-RMSD values of 133 complexes obtained for all 6 docking methods by blind docking. **S5 (a-f).** I-RMSD values of 133 complexes obtained for all 6 docking methods by blind docking. **S6 (a-d).** FNAT values of 133 complexes obtained for 4 docking methods by re-docking. **S7 (a-d).** L-RMSD values of 133 complexes obtained for 4 docking methods by re-docking. **S8 (a-d).** I-RMSD values of 133 complexes obtained for 4 docking methods by re-docking. **S9 (a-c).** FNAT, L-RMSD and I-RMSD values for the top poses of all the considered docking methods obtained by blind docking. **S10 (a-c).** FNAT, L-RMSD and I-RMSD values for the best poses of all the considered docking methods obtained by blind docking. **S11.** Deviation in the success rate with the increase in the I-RMSD values obtained by blind docking. **S12. (a-g).** L-RMSD values of 40 complexes obtained for 7 docking methods by blind docking. **S13 (a-d).** L-RMSD values of 40 complexes obtained for docking methods which perform re-docking. (DOCX 1777 kb)

Additional file 2: Performance of different docking method on the PPDbench dataset with resolution 1–2 Å and 2–3 Å for top pose based on average (a) FNAT value and (b) I-RMSD respectively. (JPG 349 kb)

Additional file 3: Performance of different docking method on the PPDbench dataset with resolution 1–2 Å and 2–3 Å for best pose based on average (a) FNAT value and (b) I-RMSD respectively. (JPG 352 kb)

Additional file 4: Performance of different docking methods on the PPDbench dataset with the different secondary structure for top pose based on (a) FNAT and (b) I-RMSD value respectively. (JPG 362 kb)

Additional file 5: Performance of different docking methods on the PPDbench dataset with the different secondary structure for best pose based on (a) FNAT and (b) I-RMSD value respectively. (JPG 274 kb)

## Abbreviations

**CAPRI**: Critical Assessment of PRedicted Interactions
**I-RMSD**: Interface RMSD
**L-RMSD**: Ligand RMSD
**RMSD**: Root Mean Square Deviation
**SH**: Spherical Harmonics
