# Supplementary material for: Benchmarking of different molecular docking methods for protein-peptide docking
Source: BMC Bioinformatics. 2019 Feb 4;19(Suppl 13):426. doi: 10.1186/s12859-018-2449-y (PMC7394329; doi:10.1186/s12859-018-2449-y)
Supplement: Supplementary file 1 — S1. Sequence similarity at 40% between the proteins of PPDbench dataset using CD-HIT software. S2. Distance between the original peptides and the peptides with changed coordinates. S3 (a-f). FNAT values of 133 complexes obtained for all 6 docking methods by blind docking. S4 (a-f). L-RMSD values of 133 complexes obtained for all 6 docking methods by blind docking. S5 (a-f). I-RMSD values of 133 complexes obtained for all 6 docking methods by blind docking. S6 (a-d). FNAT values of 133 complexes obtained for 4 docking methods by re-docking. S7 (a-d). L-RMSD values of 133 complexes obtained for 4 docking methods by re-docking. S8 (a-d). I-RMSD values of 133 complexes obtained for 4 docking methods by re-docking. S9 (a-c). FNAT, L-RMSD and I-RMSD values for the top poses of all the considered docking methods obtained by blind docking. S10 (a-c). FNAT, L-RMSD and I-RMSD values for the best poses of all the considered docking methods obtained by blind docking. S11. Deviation in the success rate with the increase in the I-RMSD values obtained by blind docking. S12. (a-g). L-RMSD values of 40 complexes obtained for 7 docking methods by blind docking. S13 (a-d). L-RMSD values of 40 complexes obtained for docking methods which perform re-docking. (DOCX 1777 kb) [file 12859_2018_2449_MOESM1_ESM.docx]

**Supplementary Information**

**Benchmarking of different molecular docking methods for protein-peptide docking**

**Piyush Agrawal^1#^, Harinder Singh^1#^, Hemant Kumar Srivastava^1^, Sandeep Singh^1^, Gaurav Kishore^1^, Gajendra P. S. Raghava^1,2^***

^1^CSIR-Institute of Microbial Technology, Sector 39A, Chandigarh, India.

^2^Indraprastha Institute of Information Technology, Okhla Phase III, Delhi India

^#^Authors Contributed Equally

**Emails of Authors:**

PA: piyush_11@imtech.res.in

HS: harinder@imtech.res.in

HKS: hemantkrsri@gmail.com

SS: sandybioteck@gmail.com

GK: gauravkishor3@gmail.com

**^*^ Corresponding author**

Professor of Computation Biology, Indraprastha Institute of Information Technology (IIIT Delhi), Okhla Phase III, New Delhi-110020, India. Phone: +91-1126907444

E-mail: [raghava@iiitd.ac.in](mailto:raghava@iiitd.ac.in)

**S1. Sequence similarity at 40% between the proteins of PPDbench dataset using CD-HIT software.**

| **Cluster Number** | **Protein PDB ID with chain** | **PDB ID showing Similarity** | **Percentage of Similarity (%)** |
| --- | --- | --- | --- |
| 1 | 4HOMA | NA | NA |
| 2 | 4GYWA | NA | NA |
| 3 | 3W1BA | 4HTPA | 98 |
| 4 | 2WHXA | NA | NA |
| 5 | 2P1OB | NA | NA |
| 6 | 1CVUB | NA | NA |
| 7 | 1T08A | NA | NA |
| 8 | 1XOCA | NA | NA |
| 9 | 2FFUA | NA | NA |
| 10 | 4GQ6A | NA | NA |
| 11 | 3TZYB | NA | NA |
| 12 | 2FFFB | NA | NA |
| 13 | 2FTSA | NA | NA |
| 14 | 2XU7B | NA | NA |
| 15 | 3C3RA | NA | NA |
| 16 | 3LLBA | NA | NA |
| 17 | 2XRWA | NA | NA |
| 18 | 2R9QB | NA | NA |
| 19 | 2B9HA | NA | NA |
| 20 | 2R7GC | NA | NA |
| 21 | 2CE8A | NA | NA |
| 22 | 4F1ZA | NA | NA |
| 23 | 2P0WA | NA | NA |
| 24 | 2VR3B | NA | NA |
| 25 | 4ERYA | NA | NA |
| 26 | 3QISA | NA | NA |
| 27 | 1X2RA | NA | NA |
| 28 | 3PTLA | NA | NA |
| 29 | 4DCBA | NA | NA |
| 30 | 1K5NA | 2DYPA, 3ERYA | 82, 71 |
| 31 | 3P72A | NA | NA |
| 32 | 2P54A | 2FVJA, 3U9QA | 63, 63 |
| 33 | 2AQ9A | NA | NA |
| 34 | 1EG4A | NA | NA |
| 35 | 2PUXB | NA | NA |
| 36 | 2CCHD | NA | NA |
| 37 | 1T7RA | NA | NA |
| 38 | 1RXZA | NA | NA |
| 39 | 1NQ7A | NA | NA |
| 40 | 3L0EA | NA | NA |
| 41 | 2QSEB | NA | NA |
| 42 | 3GYTA | 3UP3A | 47 |
| 43 | 3P8FA | 1SFIA, 2A31A | 40, 43 |
| 44 | 1YUCA | 1YMTA | 60 |
| 45 | 2O4JA | 3VTCA | 99 |
| 46 | 1JBUH | NA | NA |
| 47 | 4H4FA | NA | NA |
| 48 | 3KMRA | NA | NA |
| 49 | 3H1ZA | NA | NA |
| 50 | 3OLFA | NA | NA |
| 51 | 1PZLA | NA | NA |
| 52 | 1U00A | NA | NA |
| 53 | 1TFCA | NA | NA |
| 54 | 3TJVA | NA | NA |
| 55 | 2O02A | NA | NA |
| 56 | 1QKZH | NA | NA |
| 57 | 3RQGC | NA | NA |
| 58 | 2P1TA | NA | NA |
| 59 | 3ZQHA | NA | NA |
| 60 | 1NLNA | NA | NA |
| 61 | 2BBAA | NA | NA |
| 62 | 2V8YA | NA | NA |
| 63 | 4J8SA | NA | NA |
| 64 | 2QBXB | NA | NA |
| 65 | 1NX1A | NA | NA |
| 66 | 2QOSC | NA | NA |
| 67 | 3AYUA | NA | NA |
| 68 | 3V2XA | NA | NA |
| 69 | 1NTVA | NA | NA |
| 70 | 1H6WA | NA | NA |
| 71 | 3G2SA | NA | NA |
| 72 | 2A25A | NA | NA |
| 73 | 4GXLA | NA | NA |
| 74 | 3OBQA | NA | NA |
| 75 | 3IVVA | NA | NA |
| 76 | 3SO6A | NA | NA |
| 77 | 4B4NA | NA | NA |
| 78 | 1OW6A | NA | NA |
| 79 | 3LLZA | NA | NA |
| 80 | 3BFQG | NA | NA |
| 81 | 2FKAA | 2FMFA | 100 |
| 82 | 1RSTB | NA | NA |
| 83 | 2ZJDA | NA | NA |
| 84 | 3D32A | NA | NA |
| 85 | 1OU8A | NA | NA |
| 86 | 1OJ5A | NA | NA |
| 87 | 1D4TA | NA | NA |
| 88 | 2HT9A | NA | NA |
| 89 | 2PEHA | NA | NA |
| 90 | 3SFJA | NA | NA |
| 91 | 1CJRB | NA | NA |
| 92 | 1MFGA | NA | NA |
| 93 | 4K0UA | NA | NA |
| 94 | 3FDOA | 1T4FM | 53 |
| 95 | 3RM1A | NA | NA |
| 96 | 4E34B | NA | NA |
| 97 | 3KUSA | 3KUJA | 98 |
| 98 | 3I5RA | NA | NA |
| 99 | 3DS4A | NA | NA |
| 100 | 2W2UA | NA | NA |
| 101 | 1HC9A | NA | NA |
| 102 | 3AWRA | NA | NA |
| 103 | 3ASLA | NA | NA |
| 104 | 2O9VA | NA | NA |
| 105 | 2VKNA | NA | NA |
| 106 | 1SSHA | NA | NA |
| 107 | 2PUYB | NA | NA |
| 108 | 1OAIA | NA | NA |
| 109 | 2DRKA | NA | NA |
| 110 | 1UJ0A | 2D0NC, 2VWFA | 50, 50 |
| 111 | 4EIKA | NA | NA |
| 112 | 4IIMA | NA | NA |
| 113 | 1CKAA | NA | NA |
| 114 | 2XVCA | NA | NA |
| 115 | 4F14A | NA | NA |
| 116 | 1YWOA | NA | NA |
| 117 | 2OEIA | 2HO2A | 100 |

* NA = Not Available (it means no protein chain was found to be similar)

**S2. Distance between the original peptides and the peptides with changed coordinates.**

| **Sr. No.** | **PDB_ID** | **Distance (in Å)** |
| --- | --- | --- |
| 1 | 1cjr | 26.37 |
| 2 | 1cka | 75.52 |
| 3 | 1cvu | 75.52 |
| 4 | 1d4t | 17.70 |
| 5 | 1eg4 | 78.20 |
| 6 | 1h6w | 156.50 |
| 7 | 1hc9 | 56.13 |
| 8 | 1jbu | 38.44 |
| 9 | 1k5n | 39.34 |
| 10 | 1mfg | 28.70 |
| 11 | 1nln | 68.41 |
| 12 | 1nq7 | 30.05 |
| 13 | 1ntv | 14.82 |
| 14 | 1nx1 | 87.87 |
| 15 | 1oai | 37.24 |
| 16 | 1oj5 | 41.77 |
| 17 | 1ou8 | 113.00 |
| 18 | 1ow6 | 47.78 |
| 19 | 1pzl | 101.40 |
| 20 | 1qkz | 43.38 |
| 21 | 1rst | 68.97 |
| 22 | 1rxz | 90.03 |
| 23 | 1sfi | 46.46 |
| 24 | 1ssh | 40.96 |
| 25 | 1t08 | 52.47 |
| 26 | 1t4f | 29.57 |
| 27 | 1t7r | 59.55 |
| 28 | 1tfc | 56.76 |
| 29 | 1u00 | 43.11 |
| 30 | 1uj0 | 34.52 |
| 31 | 1x2r | 69.22 |
| 32 | 1xoc | 55.89 |
| 33 | 1ymt | 108.50 |
| 34 | 1yuc | 66.28 |
| 35 | 1ywo | 30.70 |
| 36 | 2a25 | 47.87 |
| 37 | 2a3i | 90.95 |
| 38 | 2aq9 | 111.40 |
| 39 | 2b9h | 26.06 |
| 40 | 2bba | 55.86 |
| 41 | 2cch | 34.70 |
| 42 | 2ce8 | 42.17 |
| 43 | 2d0n | 35.61 |
| 44 | 2drk | 30.51 |
| 45 | 2dyp | 40.60 |
| 46 | 2fff | 56.82 |
| 47 | 2ffu | 20.88 |
| 48 | 2fka | 123.40 |
| 49 | 2fmf | 124.70 |
| 50 | 2fts | 50.23 |
| 51 | 2fvj | 32.35 |
| 52 | 2ho2 | 31.19 |
| 53 | 2ht9 | 61.99 |
| 54 | 2o02 | 18.29 |
| 55 | 2o4j | 69.96 |
| 56 | 2o9v | 36.74 |
| 57 | 2oei | 21.66 |
| 58 | 2p0w | 109.30 |
| 59 | 2p1o | 104.00 |
| 60 | 2p1t | 19.99 |
| 61 | 2p54 | 46.33 |
| 62 | 2peh | 22.68 |
| 63 | 2pux | 44.13 |
| 64 | 2puy | 68.88 |
| 65 | 2qbx | 14.11 |
| 66 | 2qos | 71.45 |
| 67 | 2qse | 35.56 |
| 68 | 2r7g | 26.42 |
| 69 | 2r9q | 70.09 |
| 70 | 2v8y | 53.45 |
| 71 | 2vkn | 15.39 |
| 72 | 2vr3 | 87.12 |
| 73 | 2vwf | 27.89 |
| 74 | 2w2u | 51.26 |
| 75 | 2whx | 32.80 |
| 76 | 2xrw | 32.68 |
| 77 | 2xu7 | 42.02 |
| 78 | 2xvc | 43.74 |
| 79 | 2zjd | 47.85 |
| 80 | 3asl | 30.61 |
| 81 | 3awr | 25.27 |
| 82 | 3ayu | 24.22 |
| 83 | 3bfq | 31.60 |
| 84 | 3c3r | 53.59 |
| 85 | 3d32 | 48.09 |
| 86 | 3ds4 | 14.76 |
| 87 | 3ery | 15.71 |
| 88 | 3fdo | 27.24 |
| 89 | 3g2s | 48.87 |
| 90 | 3gyt | 50.90 |
| 91 | 3h1z | 48.66 |
| 92 | 3i5r | 36.65 |
| 93 | 3ivv | 27.68 |
| 94 | 3kmr | 20.85 |
| 95 | 3kuj | 20.39 |
| 96 | 3kus | 30.36 |
| 97 | 3l0e | 37.89 |
| 98 | 3ll8 | 30.78 |
| 99 | 3llz | 18.64 |
| 100 | 3obq | 12.57 |
| 101 | 3olf | 23.30 |
| 102 | 3p72 | 47.25 |
| 103 | 3p8f | 27.15 |
| 104 | 3ptl | 27.37 |
| 105 | 3qis | 35.03 |
| 106 | 3rm1 | 21.87 |
| 107 | 3rqg | 68.86 |
| 108 | 3sfj | 46.99 |
| 109 | 3so6 | 72.72 |
| 110 | 3tjv | 27.48 |
| 111 | 3tzy | 65.35 |
| 112 | 3u9q | 29.92 |
| 113 | 3up3 | 44.56 |
| 114 | 3v2x | 22.42 |
| 115 | 3vtc | 28.78 |
| 116 | 3w1b | 82.21 |
| 117 | 3zqh | 19.82 |
| 118 | 4b4n | 20.53 |
| 119 | 4dcb | 20.74 |
| 120 | 4e34 | 47.16 |
| 121 | 4eik | 45.56 |
| 122 | 4ery | 23.58 |
| 123 | 4f14 | 30.98 |
| 124 | 4f1z | 18.03 |
| 125 | 4gq6 | 25.80 |
| 126 | 4gxl | 28.25 |
| 127 | 4gyw | 28.45 |
| 128 | 4h4f | 87.26 |
| 129 | 4hom | 66.46 |
| 130 | 4htp | 11.89 |
| 131 | 4iim | 27.38 |
| 132 | 4j8s | 38.40 |
| 133 | 4k0u | 20.38 |

**S3(a). FNAT values of all 20 poses obtained after blind docking by ATTRACT software on 133 protein-peptide complexes.**

| **ID** | **Pose1** | **Pose2** | **Pose3** | **Pose4** | **Pose5** | **Pose6** | **Pose7** | **Pose8** | **Pose9** | **Pose10** | **Pose11** | **Pose12** | **Pose13** | **Pose14** | **Pose15** | **Pose16** | **Pose17** | **Pose18** | **Pose19** | **Pose20** |
| --- | --- | --- | --- | --- | --- | --- | --- | --- | --- | --- | --- | --- | --- | --- | --- | --- | --- | --- | --- | --- |
| 1cjr | 0.00 | 0.00 | 4.08 | 0.00 | 0.00 | 4.08 | 2.04 | 2.04 | 2.04 | 0.00 | 2.04 | 2.04 | 0.00 | 2.04 | 8.16 | 4.08 | 14.29 | 8.16 | 2.04 | 0.00 |
| 1cka | 46.43 | 42.86 | 42.86 | 46.43 | 46.43 | 42.86 | 42.86 | 42.86 | 89.29 | 89.29 | 89.29 | 42.86 | 89.29 | 39.29 | 96.43 | 17.86 | 75.00 | 46.43 | 14.29 | 17.86 |
| 1cvu | 0.00 | 0.00 | 0.00 | 0.00 | 0.00 | 0.00 | 0.00 | 0.00 | 0.00 | 0.00 | 0.00 | 0.00 | 0.00 | 0.00 | 0.00 | 0.00 | 0.00 | 0.00 | 0.00 | 0.00 |
| 1d4t | 96.43 | 0.00 | 0.00 | 5.36 | 0.00 | 1.79 | 0.00 | 0.00 | 0.00 | 1.79 | 0.00 | 3.57 | 5.36 | 0.00 | 0.00 | 0.00 | 0.00 | 0.00 | 0.00 | 0.00 |
| 1eg4 | 0.00 | 0.00 | 6.06 | 21.21 | 18.18 | 0.00 | 0.00 | 9.09 | 15.15 | 0.00 | 0.00 | 0.00 | 12.12 | 15.15 | 0.00 | 0.00 | 27.27 | 0.00 | 27.27 | 21.21 |
| 1h6w | 0.00 | 25.35 | 29.58 | 4.23 | 4.23 | 0.00 | 8.45 | 7.04 | 0.00 | 11.27 | 2.82 | 0.00 | 0.00 | 23.94 | 4.23 | 0.00 | 14.08 | 0.00 | 0.00 | 0.00 |
| 1hc9 | 6.67 | 17.78 | 0.00 | 2.22 | 2.22 | 2.22 | 4.44 | 2.22 | 2.22 | 2.22 | 8.89 | 11.11 | 2.22 | 2.22 | 0.00 | 0.00 | 8.89 | 8.89 | 0.00 | 8.89 |
| 1jbu | 88.52 | 86.89 | 37.70 | 24.59 | 37.70 | 22.95 | 22.95 | 0.00 | 45.90 | 0.00 | 0.00 | 0.00 | 18.03 | 0.00 | 16.39 | 0.00 | 0.00 | 18.03 | 3.28 | 19.67 |
| 1k5n | 9.26 | 3.70 | 0.00 | 0.00 | 0.00 | 0.00 | 0.00 | 0.00 | 0.00 | 5.56 | 0.00 | 0.00 | 3.70 | 0.00 | 0.00 | 11.11 | 0.00 | 0.00 | 5.56 | 3.70 |
| 1mfg | 17.65 | 11.76 | 11.76 | 2.94 | 14.71 | 17.65 | 0.00 | 11.76 | 2.94 | 17.65 | 2.94 | 5.88 | 2.94 | 0.00 | 11.76 | 14.71 | 5.88 | 2.94 | 14.71 | 5.88 |
| 1nln | 96.92 | 49.23 | 40.00 | 40.00 | 49.23 | 21.54 | 44.62 | 23.08 | 23.08 | 24.62 | 24.62 | 24.62 | 23.08 | 23.08 | 23.08 | 49.23 | 20.00 | 35.38 | 35.38 | 21.54 |
| 1nq7 | 0.00 | 0.00 | 0.00 | 0.00 | 0.00 | 0.00 | 0.00 | 0.00 | 0.00 | 0.00 | 0.00 | 0.00 | 0.00 | 0.00 | 0.00 | 0.00 | 0.00 | 0.00 | 96.97 | 0.00 |
| 1ntv | 8.70 | 4.35 | 0.00 | 0.00 | 0.00 | 97.83 | 4.35 | 0.00 | 0.00 | 0.00 | 0.00 | 6.52 | 0.00 | 13.04 | 0.00 | 8.70 | 0.00 | 4.35 | 6.52 | 2.17 |
| 1nx1 | 0.00 | 0.00 | 0.00 | 0.00 | 0.00 | 0.00 | 0.00 | 0.00 | 0.00 | 0.00 | 0.00 | 12.50 | 0.00 | 0.00 | 65.00 | 15.00 | 60.00 | 60.00 | 0.00 | 0.00 |
| 1oai | 3.23 | 0.00 | 0.00 | 0.00 | 58.06 | 58.06 | 0.00 | 45.16 | 38.71 | 32.26 | 45.16 | 35.48 | 77.42 | 58.06 | 6.45 | 54.84 | 3.23 | 54.84 | 16.13 | 3.23 |
| 1oj5 | 8.00 | 0.00 | 0.00 | 100.00 | 0.00 | 0.00 | 0.00 | 0.00 | 0.00 | 0.00 | 0.00 | 12.00 | 0.00 | 0.00 | 12.00 | 0.00 | 0.00 | 0.00 | 0.00 | 0.00 |
| 1ou8 | 6.67 | 13.33 | 8.89 | 0.00 | 15.56 | 20.00 | 20.00 | 20.00 | 15.56 | 15.56 | 17.78 | 22.22 | 0.00 | 15.56 | 2.22 | 13.33 | 0.00 | 0.00 | 8.89 | 40.00 |
| 1ow6 | 0.00 | 0.00 | 0.00 | 0.00 | 0.00 | 0.00 | 50.00 | 50.00 | 50.00 | 50.00 | 50.00 | 0.00 | 50.00 | 0.00 | 41.67 | 50.00 | 0.00 | 50.00 | 41.67 | 0.00 |
| 1pzl | 96.67 | 93.33 | 90.00 | 86.67 | 0.00 | 36.67 | 53.33 | 0.00 | 36.67 | 50.00 | 0.00 | 0.00 | 0.00 | 30.00 | 0.00 | 0.00 | 0.00 | 0.00 | 0.00 | 0.00 |
| 1qkz | 30.77 | 20.51 | 35.90 | 23.08 | 15.38 | 25.64 | 38.46 | 25.64 | 23.08 | 25.64 | 15.38 | 15.38 | 15.38 | 25.64 | 25.64 | 23.08 | 15.38 | 10.26 | 15.38 | 30.77 |
| 1rst | 34.48 | 37.93 | 31.03 | 34.48 | 0.00 | 0.00 | 0.00 | 48.28 | 48.28 | 0.00 | 0.00 | 0.00 | 27.59 | 89.66 | 24.14 | 0.00 | 65.52 | 10.34 | 86.21 | 27.59 |
| 1rxz | 92.59 | 48.15 | 7.41 | 51.85 | 46.30 | 9.26 | 7.41 | 12.96 | 42.59 | 48.15 | 27.78 | 48.15 | 25.93 | 16.67 | 22.22 | 0.00 | 16.67 | 20.37 | 9.26 | 18.52 |
| 1sfi | 82.46 | 0.00 | 1.75 | 1.75 | 7.02 | 14.04 | 15.79 | 0.00 | 5.26 | 0.00 | 1.75 | 10.53 | 5.26 | 1.75 | 21.05 | 0.00 | 12.28 | 0.00 | 0.00 | 0.00 |
| 1ssh | 93.33 | 90.00 | 96.67 | 90.00 | 96.67 | 96.67 | 83.33 | 96.67 | 66.67 | 53.33 | 56.67 | 60.00 | 60.00 | 16.67 | 46.67 | 40.00 | 63.33 | 43.33 | 63.33 | 40.00 |
| 1t08 | 0.00 | 0.00 | 0.00 | 0.00 | 0.00 | 0.00 | 0.00 | 0.00 | 0.00 | 0.00 | 4.48 | 2.99 | 0.00 | 0.00 | 0.00 | 0.00 | 0.00 | 0.00 | 0.00 | 0.00 |
| 1t4f | 100.00 | 100.00 | 100.00 | 100.00 | 100.00 | 100.00 | 25.00 | 100.00 | 89.29 | 28.57 | 32.14 | 42.86 | 21.43 | 64.29 | 50.00 | 42.86 | 53.57 | 42.86 | 28.57 | 42.86 |
| 1t7r | 96.77 | 96.77 | 93.55 | 83.87 | 0.00 | 0.00 | 0.00 | 12.90 | 0.00 | 29.03 | 0.00 | 0.00 | 0.00 | 0.00 | 0.00 | 51.61 | 0.00 | 32.26 | 0.00 | 0.00 |
| 1tfc | 0.00 | 0.00 | 0.00 | 0.00 | 0.00 | 0.00 | 0.00 | 0.00 | 0.00 | 0.00 | 0.00 | 0.00 | 0.00 | 0.00 | 0.00 | 0.00 | 0.00 | 35.48 | 93.55 | 35.48 |
| 1u00 | 10.42 | 2.08 | 0.00 | 0.00 | 0.00 | 0.00 | 4.17 | 2.08 | 0.00 | 0.00 | 20.83 | 0.00 | 0.00 | 0.00 | 0.00 | 0.00 | 0.00 | 0.00 | 0.00 | 0.00 |
| 1uj0 | 100.00 | 100.00 | 96.30 | 51.85 | 55.56 | 48.15 | 92.59 | 44.44 | 11.11 | 25.93 | 18.52 | 18.52 | 25.93 | 25.93 | 77.78 | 51.85 | 77.78 | 11.11 | 77.78 | 77.78 |
| 1x2r | 18.60 | 16.28 | 41.86 | 4.65 | 53.49 | 4.65 | 0.00 | 95.35 | 60.47 | 0.00 | 23.26 | 13.95 | 18.60 | 23.26 | 20.93 | 0.00 | 9.30 | 0.00 | 0.00 | 9.30 |
| 1xoc | 1.18 | 0.00 | 0.00 | 0.00 | 2.35 | 0.00 | 1.18 | 1.18 | 1.18 | 0.00 | 0.00 | 0.00 | 0.00 | 0.00 | 1.18 | 1.18 | 0.00 | 0.00 | 0.00 | 0.00 |
| 1ymt | 67.86 | 32.14 | 28.57 | 32.14 | 85.71 | 28.57 | 96.43 | 96.43 | 78.57 | 96.43 | 0.00 | 92.86 | 39.29 | 32.14 | 35.71 | 42.86 | 32.14 | 0.00 | 0.00 | 0.00 |
| 1yuc | 2.78 | 0.00 | 0.00 | 0.00 | 0.00 | 0.00 | 0.00 | 0.00 | 0.00 | 88.89 | 94.44 | 0.00 | 91.67 | 91.67 | 2.78 | 0.00 | 0.00 | 97.22 | 0.00 | 0.00 |
| 1ywo | 24.00 | 24.00 | 24.00 | 20.00 | 24.00 | 16.00 | 24.00 | 28.00 | 100.00 | 24.00 | 24.00 | 28.00 | 28.00 | 28.00 | 12.00 | 32.00 | 88.00 | 20.00 | 20.00 | 24.00 |
| 2a25 | 0.00 | 0.00 | 0.00 | 0.00 | 0.00 | 0.00 | 0.00 | 0.00 | 0.00 | 0.00 | 75.00 | 0.00 | 0.00 | 0.00 | 0.00 | 0.00 | 17.50 | 0.00 | 0.00 | 20.00 |
| 2a3i | 93.75 | 100.00 | 93.75 | 96.88 | 75.00 | 65.63 | 81.25 | 53.13 | 43.75 | 0.00 | 0.00 | 0.00 | 43.75 | 0.00 | 43.75 | 6.25 | 0.00 | 46.88 | 25.00 | 40.63 |
| 2aq9 | 0.00 | 0.00 | 0.00 | 0.00 | 0.00 | 0.00 | 0.00 | 0.00 | 0.00 | 0.00 | 0.00 | 0.00 | 0.00 | 0.00 | 0.00 | 0.00 | 0.00 | 0.00 | 0.00 | 0.00 |
| 2b9h | 79.59 | 81.63 | 83.67 | 91.84 | 0.00 | 0.00 | 0.00 | 0.00 | 0.00 | 0.00 | 0.00 | 0.00 | 0.00 | 0.00 | 14.29 | 14.29 | 14.29 | 0.00 | 16.33 | 16.33 |
| 2bba | 94.74 | 0.00 | 91.23 | 0.00 | 0.00 | 0.00 | 0.00 | 0.00 | 0.00 | 0.00 | 0.00 | 0.00 | 0.00 | 0.00 | 1.75 | 0.00 | 0.00 | 0.00 | 0.00 | 0.00 |
| 2cch | 100.00 | 13.95 | 76.74 | 13.95 | 46.51 | 13.95 | 0.00 | 0.00 | 0.00 | 46.51 | 0.00 | 13.95 | 0.00 | 0.00 | 0.00 | 0.00 | 0.00 | 74.42 | 0.00 | 0.00 |
| 2ce8 | 22.58 | 19.35 | 16.13 | 0.00 | 16.13 | 19.35 | 0.00 | 0.00 | 16.13 | 16.13 | 0.00 | 0.00 | 0.00 | 0.00 | 0.00 | 0.00 | 0.00 | 19.35 | 19.35 | 0.00 |
| 2d0n | 100.00 | 96.00 | 100.00 | 96.00 | 92.00 | 92.00 | 84.00 | 68.00 | 76.00 | 64.00 | 76.00 | 68.00 | 72.00 | 72.00 | 76.00 | 72.00 | 84.00 | 76.00 | 72.00 | 68.00 |
| 2drk | 20.00 | 20.00 | 93.33 | 20.00 | 80.00 | 16.67 | 100.00 | 20.00 | 100.00 | 13.33 | 20.00 | 13.33 | 100.00 | 100.00 | 16.67 | 100.00 | 13.33 | 90.00 | 20.00 | 16.67 |
| 2dyp | 1.54 | 0.00 | 13.85 | 0.00 | 0.00 | 0.00 | 3.08 | 0.00 | 0.00 | 3.08 | 3.08 | 18.46 | 1.54 | 1.54 | 0.00 | 47.69 | 0.00 | 0.00 | 0.00 | 0.00 |
| 2fff | 0.00 | 95.65 | 0.00 | 0.00 | 6.52 | 15.22 | 6.52 | 0.00 | 13.04 | 13.04 | 0.00 | 2.17 | 4.35 | 4.35 | 0.00 | 0.00 | 0.00 | 0.00 | 4.35 | 4.35 |
| 2ffu | 0.00 | 0.00 | 0.00 | 0.00 | 0.00 | 0.00 | 0.00 | 0.00 | 0.00 | 0.00 | 0.00 | 0.00 | 0.00 | 0.00 | 0.00 | 0.00 | 0.00 | 0.00 | 0.00 | 0.00 |
| 2fka | 47.62 | 66.67 | 61.90 | 66.67 | 76.19 | 66.67 | 66.67 | 47.62 | 47.62 | 23.81 | 23.81 | 19.05 | 23.81 | 19.05 | 47.62 | 47.62 | 66.67 | 28.57 | 47.62 | 19.05 |
| 2fmf | 9.09 | 50.00 | 59.09 | 50.00 | 50.00 | 45.45 | 50.00 | 45.45 | 50.00 | 36.36 | 63.64 | 45.45 | 45.45 | 45.45 | 22.73 | 22.73 | 45.45 | 50.00 | 50.00 | 50.00 |
| 2fts | 96.23 | 94.34 | 94.34 | 64.15 | 60.38 | 0.00 | 0.00 | 22.64 | 18.87 | 0.00 | 0.00 | 0.00 | 0.00 | 0.00 | 0.00 | 0.00 | 0.00 | 0.00 | 22.64 | 1.89 |
| 2fvj | 0.00 | 0.00 | 0.00 | 0.00 | 0.00 | 0.00 | 0.00 | 0.00 | 0.00 | 0.00 | 0.00 | 0.00 | 100.00 | 0.00 | 0.00 | 0.00 | 0.00 | 0.00 | 0.00 | 0.00 |
| 2ho2 | 35.29 | 35.29 | 35.29 | 23.53 | 17.65 | 35.29 | 35.29 | 41.18 | 70.59 | 17.65 | 47.06 | 58.82 | 35.29 | 5.88 | 5.88 | 47.06 | 70.59 | 82.35 | 5.88 | 17.65 |
| 2ht9 | 0.00 | 0.00 | 0.00 | 0.00 | 0.00 | 0.00 | 0.00 | 0.00 | 0.00 | 0.00 | 0.00 | 0.00 | 0.00 | 0.00 | 0.00 | 0.00 | 7.69 | 0.00 | 0.00 | 0.00 |
| 2o02 | 35.21 | 0.00 | 0.00 | 0.00 | 0.00 | 0.00 | 0.00 | 0.00 | 0.00 | 0.00 | 0.00 | 0.00 | 0.00 | 0.00 | 0.00 | 0.00 | 0.00 | 0.00 | 0.00 | 0.00 |
| 2o4j | 91.18 | 76.47 | 88.24 | 0.00 | 0.00 | 0.00 | 0.00 | 0.00 | 0.00 | 20.59 | 0.00 | 0.00 | 20.59 | 0.00 | 11.76 | 0.00 | 0.00 | 20.59 | 0.00 | 23.53 |
| 2o9v | 80.00 | 76.00 | 76.00 | 76.00 | 100.00 | 96.00 | 88.00 | 96.00 | 20.00 | 100.00 | 20.00 | 20.00 | 16.00 | 16.00 | 16.00 | 100.00 | 16.00 | 16.00 | 16.00 | 96.00 |
| 2oei | 31.82 | 31.82 | 31.82 | 31.82 | 31.82 | 31.82 | 31.82 | 27.27 | 95.45 | 95.45 | 95.45 | 18.18 | 95.45 | 100.00 | 13.64 | 18.18 | 31.82 | 31.82 | 31.82 | 31.82 |
| 2p0w | 0.00 | 0.00 | 1.56 | 0.00 | 3.13 | 3.13 | 7.81 | 1.56 | 0.00 | 0.00 | 0.00 | 7.81 | 7.81 | 92.19 | 0.00 | 0.00 | 0.00 | 0.00 | 0.00 | 0.00 |
| 2p1o | 0.00 | 0.00 | 4.44 | 0.00 | 0.00 | 0.00 | 0.00 | 0.00 | 2.22 | 2.22 | 2.22 | 0.00 | 73.33 | 0.00 | 93.33 | 13.33 | 0.00 | 0.00 | 0.00 | 0.00 |
| 2p1t | 0.00 | 0.00 | 0.00 | 0.00 | 0.00 | 0.00 | 0.00 | 0.00 | 0.00 | 0.00 | 0.00 | 0.00 | 0.00 | 0.00 | 0.00 | 0.00 | 34.29 | 0.00 | 0.00 | 0.00 |
| 2p54 | 0.00 | 0.00 | 0.00 | 0.00 | 0.00 | 0.00 | 0.00 | 0.00 | 0.00 | 0.00 | 0.00 | 0.00 | 0.00 | 0.00 | 0.00 | 0.00 | 0.00 | 0.00 | 0.00 | 0.00 |
| 2peh | 85.29 | 85.29 | 0.00 | 2.94 | 0.00 | 2.94 | 32.35 | 0.00 | 29.41 | 11.76 | 32.35 | 14.71 | 11.76 | 11.76 | 11.76 | 14.71 | 14.71 | 11.76 | 2.94 | 14.71 |
| 2pux | 97.30 | 94.59 | 89.19 | 62.16 | 91.89 | 62.16 | 86.49 | 59.46 | 27.03 | 0.00 | 0.00 | 29.73 | 0.00 | 0.00 | 0.00 | 0.00 | 32.43 | 0.00 | 35.14 | 0.00 |
| 2puy | 100.00 | 0.00 | 0.00 | 8.11 | 45.95 | 45.95 | 8.11 | 45.95 | 45.95 | 8.11 | 51.35 | 0.00 | 0.00 | 0.00 | 0.00 | 45.95 | 0.00 | 45.95 | 40.54 | 5.41 |
| 2qbx | 0.00 | 13.56 | 15.25 | 0.00 | 0.00 | 0.00 | 0.00 | 0.00 | 0.00 | 0.00 | 0.00 | 0.00 | 0.00 | 0.00 | 0.00 | 0.00 | 0.00 | 0.00 | 15.25 | 0.00 |
| 2qos | 0.00 | 0.00 | 67.92 | 0.00 | 1.89 | 0.00 | 0.00 | 0.00 | 0.00 | 0.00 | 9.43 | 0.00 | 0.00 | 0.00 | 9.43 | 11.32 | 0.00 | 0.00 | 0.00 | 1.89 |
| 2qse | 96.67 | 96.67 | 96.67 | 96.67 | 96.67 | 90.00 | 90.00 | 0.00 | 50.00 | 50.00 | 50.00 | 0.00 | 50.00 | 0.00 | 50.00 | 66.67 | 0.00 | 0.00 | 50.00 | 0.00 |
| 2r7g | 0.00 | 0.00 | 0.00 | 0.00 | 0.00 | 0.00 | 0.00 | 0.00 | 4.26 | 0.00 | 0.00 | 0.00 | 0.00 | 0.00 | 0.00 | 2.13 | 0.00 | 0.00 | 0.00 | 0.00 |
| 2r9q | 12.50 | 84.38 | 71.88 | 40.63 | 37.50 | 37.50 | 12.50 | 40.63 | 40.63 | 40.63 | 34.38 | 25.00 | 37.50 | 37.50 | 0.00 | 9.38 | 18.75 | 28.13 | 34.38 | 37.50 |
| 2v8y | 97.56 | 92.68 | 90.24 | 9.76 | 9.76 | 0.00 | 9.76 | 9.76 | 0.00 | 75.61 | 7.32 | 0.00 | 0.00 | 0.00 | 0.00 | 0.00 | 0.00 | 0.00 | 0.00 | 0.00 |
| 2vkn | 100.00 | 95.45 | 100.00 | 95.45 | 95.45 | 100.00 | 86.36 | 27.27 | 27.27 | 100.00 | 72.73 | 27.27 | 72.73 | 77.27 | 22.73 | 72.73 | 27.27 | 27.27 | 68.18 | 27.27 |
| 2vr3 | 0.00 | 4.76 | 1.19 | 1.19 | 0.00 | 3.57 | 2.38 | 0.00 | 1.19 | 2.38 | 0.00 | 4.76 | 4.76 | 3.57 | 0.00 | 0.00 | 4.76 | 0.00 | 0.00 | 0.00 |
| 2vwf | 0.00 | 0.00 | 97.14 | 100.00 | 57.14 | 100.00 | 57.14 | 82.86 | 0.00 | 0.00 | 0.00 | 0.00 | 0.00 | 54.29 | 51.43 | 28.57 | 20.00 | 20.00 | 20.00 | 54.29 |
| 2w2u | 0.00 | 0.00 | 0.00 | 0.00 | 0.00 | 0.00 | 0.00 | 0.00 | 0.00 | 0.00 | 0.00 | 0.00 | 25.53 | 10.64 | 8.51 | 0.00 | 0.00 | 0.00 | 0.00 | 89.36 |
| 2whx | 0.00 | 1.23 | 2.47 | 2.47 | 2.47 | 0.00 | 0.00 | 3.70 | 0.00 | 2.47 | 3.70 | 3.70 | 3.70 | 3.70 | 3.70 | 0.00 | 0.00 | 2.47 | 0.00 | 0.00 |
| 2xrw | 92.50 | 0.00 | 35.00 | 0.00 | 0.00 | 2.50 | 0.00 | 2.50 | 0.00 | 0.00 | 0.00 | 2.50 | 22.50 | 10.00 | 0.00 | 0.00 | 2.50 | 2.50 | 0.00 | 10.00 |
| 2xu7 | 95.92 | 95.92 | 93.88 | 55.10 | 95.92 | 97.96 | 81.63 | 83.67 | 2.04 | 67.35 | 71.43 | 77.55 | 85.71 | 61.22 | 59.18 | 0.00 | 0.00 | 0.00 | 0.00 | 0.00 |
| 2xvc | 0.00 | 5.88 | 5.88 | 0.00 | 0.00 | 0.00 | 17.65 | 17.65 | 0.00 | 0.00 | 0.00 | 0.00 | 0.00 | 0.00 | 0.00 | 0.00 | 0.00 | 3.92 | 0.00 | 0.00 |
| 2zjd | 100.00 | 92.31 | 84.62 | 0.00 | 0.00 | 0.00 | 0.00 | 0.00 | 25.64 | 0.00 | 28.21 | 7.69 | 28.21 | 12.82 | 0.00 | 0.00 | 25.64 | 12.82 | 12.82 | 25.64 |
| 3asl | 0.00 | 0.00 | 0.00 | 0.00 | 0.00 | 0.00 | 0.00 | 0.00 | 0.00 | 0.00 | 0.00 | 0.00 | 0.00 | 6.45 | 3.23 | 0.00 | 0.00 | 0.00 | 0.00 | 0.00 |
| 3awr | 0.00 | 12.90 | 16.13 | 16.13 | 0.00 | 0.00 | 0.00 | 0.00 | 0.00 | 0.00 | 0.00 | 0.00 | 22.58 | 0.00 | 0.00 | 0.00 | 0.00 | 0.00 | 0.00 | 0.00 |
| 3ayu | 95.00 | 88.33 | 13.33 | 33.33 | 0.00 | 0.00 | 0.00 | 0.00 | 11.67 | 26.67 | 60.00 | 15.00 | 5.00 | 0.00 | 0.00 | 0.00 | 0.00 | 0.00 | 0.00 | 0.00 |
| 3bfq | 98.96 | 1.04 | 0.00 | 0.00 | 0.00 | 0.00 | 0.00 | 0.00 | 0.00 | 0.00 | 0.00 | 10.42 | 12.50 | 0.00 | 0.00 | 0.00 | 5.21 | 0.00 | 12.50 | 4.17 |
| 3c3r | 17.24 | 86.21 | 20.69 | 17.24 | 0.00 | 86.21 | 0.00 | 34.48 | 37.93 | 0.00 | 37.93 | 37.93 | 37.93 | 0.00 | 37.93 | 0.00 | 34.48 | 0.00 | 34.48 | 0.00 |
| 3d32 | 97.62 | 16.67 | 16.67 | 19.05 | 16.67 | 16.67 | 19.05 | 7.14 | 9.52 | 4.76 | 7.14 | 19.05 | 19.05 | 9.52 | 0.00 | 16.67 | 16.67 | 2.38 | 14.29 | 11.90 |
| 3ds4 | 2.56 | 0.00 | 48.72 | 0.00 | 46.15 | 0.00 | 0.00 | 0.00 | 20.51 | 0.00 | 20.51 | 0.00 | 0.00 | 15.38 | 0.00 | 0.00 | 0.00 | 0.00 | 0.00 | 0.00 |
| 3ery | 85.94 | 62.50 | 45.31 | 48.44 | 17.19 | 39.06 | 35.94 | 21.88 | 31.25 | 12.50 | 21.88 | 12.50 | 12.50 | 39.06 | 35.94 | 26.56 | 34.38 | 32.81 | 28.13 | 35.94 |
| 3fdo | 93.33 | 93.33 | 0.00 | 93.33 | 26.67 | 20.00 | 0.00 | 0.00 | 3.33 | 0.00 | 0.00 | 0.00 | 13.33 | 6.67 | 16.67 | 0.00 | 0.00 | 23.33 | 3.33 | 23.33 |
| 3g2s | 28.95 | 92.11 | 92.11 | 0.00 | 0.00 | 2.63 | 84.21 | 0.00 | 2.63 | 0.00 | 26.32 | 2.63 | 2.63 | 7.89 | 0.00 | 26.32 | 26.32 | 0.00 | 26.32 | 2.63 |
| 3gyt | 93.10 | 0.00 | 0.00 | 0.00 | 0.00 | 0.00 | 0.00 | 0.00 | 0.00 | 0.00 | 0.00 | 0.00 | 0.00 | 0.00 | 0.00 | 0.00 | 0.00 | 0.00 | 0.00 | 0.00 |
| 3h1z | 84.91 | 16.98 | 16.98 | 11.32 | 11.32 | 0.00 | 0.00 | 7.55 | 9.43 | 1.89 | 9.43 | 0.00 | 7.55 | 47.17 | 0.00 | 0.00 | 0.00 | 0.00 | 0.00 | 0.00 |
| 3i5r | 96.15 | 92.31 | 96.15 | 92.31 | 96.15 | 23.08 | 19.23 | 23.08 | 23.08 | 23.08 | 19.23 | 19.23 | 30.77 | 19.23 | 19.23 | 23.08 | 26.92 | 26.92 | 26.92 | 30.77 |
| 3ivv | 8.82 | 14.71 | 0.00 | 0.00 | 0.00 | 0.00 | 0.00 | 0.00 | 0.00 | 2.94 | 0.00 | 14.71 | 32.35 | 0.00 | 2.94 | 11.76 | 0.00 | 0.00 | 0.00 | 0.00 |
| 3kmr | 0.00 | 0.00 | 0.00 | 0.00 | 90.00 | 0.00 | 96.67 | 0.00 | 0.00 | 0.00 | 0.00 | 0.00 | 0.00 | 0.00 | 0.00 | 0.00 | 0.00 | 0.00 | 0.00 | 0.00 |
| 3kuj | 95.45 | 6.82 | 13.64 | 6.82 | 6.82 | 2.27 | 2.27 | 2.27 | 2.27 | 9.09 | 2.27 | 2.27 | 0.00 | 15.91 | 15.91 | 2.27 | 2.27 | 0.00 | 0.00 | 0.00 |
| 3kus | 2.22 | 28.89 | 0.00 | 0.00 | 0.00 | 0.00 | 8.89 | 0.00 | 0.00 | 0.00 | 0.00 | 0.00 | 0.00 | 0.00 | 8.89 | 8.89 | 8.89 | 0.00 | 0.00 | 0.00 |
| 3l0e | 0.00 | 0.00 | 0.00 | 0.00 | 0.00 | 0.00 | 0.00 | 0.00 | 0.00 | 0.00 | 0.00 | 0.00 | 0.00 | 0.00 | 0.00 | 0.00 | 0.00 | 0.00 | 0.00 | 92.68 |
| 3ll8 | 0.00 | 97.67 | 74.42 | 88.37 | 13.95 | 13.95 | 13.95 | 16.28 | 18.60 | 30.23 | 16.28 | 20.93 | 20.93 | 30.23 | 20.93 | 4.65 | 13.95 | 0.00 | 16.28 | 20.93 |
| 3llz | 0.00 | 0.00 | 0.00 | 100.00 | 100.00 | 0.00 | 0.00 | 0.00 | 0.00 | 0.00 | 20.51 | 0.00 | 23.08 | 0.00 | 0.00 | 0.00 | 0.00 | 0.00 | 0.00 | 0.00 |
| 3obq | 22.22 | 19.44 | 22.22 | 0.00 | 0.00 | 22.22 | 0.00 | 22.22 | 22.22 | 19.44 | 0.00 | 0.00 | 22.22 | 0.00 | 0.00 | 0.00 | 0.00 | 0.00 | 0.00 | 0.00 |
| 3olf | 100.00 | 96.88 | 100.00 | 100.00 | 100.00 | 96.88 | 0.00 | 0.00 | 0.00 | 0.00 | 0.00 | 0.00 | 0.00 | 0.00 | 0.00 | 0.00 | 0.00 | 0.00 | 0.00 | 0.00 |
| 3p72 | 36.59 | 0.00 | 0.00 | 0.00 | 0.00 | 0.00 | 0.00 | 0.00 | 0.00 | 29.27 | 0.00 | 0.00 | 0.00 | 0.00 | 0.00 | 0.00 | 0.00 | 0.00 | 0.00 | 0.00 |
| 3p8f | 94.12 | 54.41 | 8.82 | 10.29 | 0.00 | 2.94 | 0.00 | 0.00 | 2.94 | 1.47 | 1.47 | 0.00 | 0.00 | 0.00 | 2.94 | 2.94 | 22.06 | 11.76 | 0.00 | 13.24 |
| 3ptl | 0.00 | 14.71 | 0.00 | 0.00 | 0.00 | 0.00 | 0.00 | 0.00 | 4.41 | 4.41 | 0.00 | 0.00 | 4.41 | 0.00 | 0.00 | 16.18 | 7.35 | 0.00 | 2.94 | 2.94 |
| 3qis | 100.00 | 100.00 | 100.00 | 100.00 | 14.71 | 0.00 | 0.00 | 0.00 | 0.00 | 0.00 | 64.71 | 0.00 | 67.65 | 23.53 | 0.00 | 67.65 | 0.00 | 0.00 | 0.00 | 0.00 |
| 3rm1 | 83.33 | 83.33 | 0.00 | 0.00 | 0.00 | 86.11 | 0.00 | 0.00 | 0.00 | 0.00 | 5.56 | 0.00 | 0.00 | 86.11 | 75.00 | 36.11 | 72.22 | 0.00 | 25.00 | 16.67 |
| 3rqg | 0.00 | 0.00 | 0.00 | 0.00 | 0.00 | 0.00 | 0.00 | 0.00 | 0.00 | 0.00 | 0.00 | 0.00 | 0.00 | 0.00 | 85.19 | 0.00 | 0.00 | 0.00 | 0.00 | 74.07 |
| 3sfj | 97.73 | 97.73 | 11.36 | 0.00 | 25.00 | 6.82 | 0.00 | 0.00 | 0.00 | 0.00 | 0.00 | 4.55 | 6.82 | 18.18 | 0.00 | 22.73 | 0.00 | 6.82 | 0.00 | 0.00 |
| 3so6 | 93.22 | 44.07 | 42.37 | 27.12 | 33.90 | 0.00 | 0.00 | 0.00 | 0.00 | 15.25 | 8.47 | 16.95 | 30.51 | 5.08 | 23.73 | 0.00 | 0.00 | 28.81 | 0.00 | 0.00 |
| 3tjv | 12.50 | 10.94 | 10.94 | 9.38 | 9.38 | 6.25 | 7.81 | 12.50 | 0.00 | 0.00 | 0.00 | 6.25 | 6.25 | 15.63 | 0.00 | 3.13 | 3.13 | 14.06 | 9.38 | 3.13 |
| 3tzy | 0.00 | 0.00 | 0.00 | 0.00 | 0.00 | 0.00 | 0.00 | 0.00 | 0.00 | 0.00 | 0.00 | 0.00 | 0.00 | 0.00 | 0.00 | 0.00 | 0.00 | 0.00 | 0.00 | 0.00 |
| 3u9q | 75.76 | 0.00 | 0.00 | 78.79 | 0.00 | 0.00 | 0.00 | 84.85 | 93.94 | 0.00 | 0.00 | 0.00 | 0.00 | 0.00 | 0.00 | 0.00 | 0.00 | 0.00 | 0.00 | 0.00 |
| 3up3 | 0.00 | 0.00 | 0.00 | 97.22 | 97.22 | 0.00 | 0.00 | 11.11 | 0.00 | 0.00 | 0.00 | 0.00 | 2.78 | 22.22 | 22.22 | 22.22 | 0.00 | 0.00 | 22.22 | 0.00 |
| 3v2x | 42.22 | 42.22 | 93.33 | 93.33 | 95.56 | 44.44 | 46.67 | 37.78 | 0.00 | 0.00 | 6.67 | 0.00 | 15.56 | 31.11 | 17.78 | 15.56 | 28.89 | 13.33 | 37.78 | 15.56 |
| 3vtc | 87.88 | 93.94 | 100.00 | 0.00 | 0.00 | 0.00 | 0.00 | 0.00 | 18.18 | 0.00 | 0.00 | 0.00 | 0.00 | 24.24 | 21.21 | 0.00 | 0.00 | 0.00 | 12.12 | 0.00 |
| 3w1b | 0.00 | 0.00 | 0.00 | 0.00 | 0.00 | 3.70 | 0.00 | 0.00 | 0.00 | 0.00 | 0.00 | 0.00 | 0.00 | 3.70 | 0.00 | 0.00 | 3.70 | 0.00 | 3.70 | 0.00 |
| 3zqh | 0.00 | 0.00 | 0.00 | 0.00 | 0.00 | 0.00 | 0.00 | 0.00 | 0.00 | 0.00 | 0.00 | 0.00 | 3.23 | 3.23 | 0.00 | 0.00 | 0.00 | 3.23 | 3.23 | 3.23 |
| 4b4n | 0.00 | 0.00 | 0.00 | 0.00 | 0.00 | 0.00 | 4.88 | 0.00 | 0.00 | 4.88 | 0.00 | 0.00 | 0.00 | 0.00 | 0.00 | 0.00 | 0.00 | 0.00 | 0.00 | 0.00 |
| 4dcb | 2.44 | 0.00 | 0.00 | 0.00 | 0.00 | 0.00 | 0.00 | 0.00 | 2.44 | 2.44 | 2.44 | 0.00 | 19.51 | 0.00 | 21.95 | 0.00 | 0.00 | 0.00 | 0.00 | 2.44 |
| 4e34 | 97.30 | 10.81 | 0.00 | 16.22 | 0.00 | 16.22 | 16.22 | 0.00 | 0.00 | 0.00 | 18.92 | 0.00 | 0.00 | 0.00 | 29.73 | 0.00 | 0.00 | 37.84 | 0.00 | 37.84 |
| 4eik | 92.31 | 92.31 | 94.87 | 97.44 | 92.31 | 7.69 | 7.69 | 7.69 | 7.69 | 7.69 | 69.23 | 20.51 | 23.08 | 23.08 | 7.69 | 10.26 | 10.26 | 7.69 | 7.69 | 7.69 |
| 4ery | 89.58 | 89.58 | 8.33 | 8.33 | 8.33 | 0.00 | 0.00 | 8.33 | 8.33 | 8.33 | 0.00 | 6.25 | 2.08 | 2.08 | 2.08 | 12.50 | 2.08 | 4.17 | 0.00 | 2.08 |
| 4f14 | 86.21 | 82.76 | 79.31 | 68.97 | 10.34 | 31.03 | 17.24 | 10.34 | 31.03 | 10.34 | 10.34 | 3.45 | 17.24 | 27.59 | 3.45 | 17.24 | 24.14 | 17.24 | 3.45 | 13.79 |
| 4f1z | 0.00 | 0.00 | 2.30 | 0.00 | 0.00 | 0.00 | 0.00 | 3.45 | 0.00 | 9.20 | 0.00 | 0.00 | 0.00 | 2.30 | 1.15 | 0.00 | 0.00 | 0.00 | 0.00 | 0.00 |
| 4gq6 | 12.50 | 17.86 | 17.86 | 12.50 | 0.00 | 0.00 | 0.00 | 0.00 | 10.71 | 19.64 | 0.00 | 0.00 | 21.43 | 14.29 | 0.00 | 3.57 | 0.00 | 0.00 | 0.00 | 0.00 |
| 4gxl | 10.53 | 28.95 | 26.32 | 10.53 | 13.16 | 13.16 | 10.53 | 13.16 | 5.26 | 0.00 | 31.58 | 28.95 | 0.00 | 10.53 | 5.26 | 0.00 | 0.00 | 28.95 | 5.26 | 7.89 |
| 4gyw | 7.69 | 9.62 | 0.00 | 15.38 | 7.69 | 0.00 | 0.00 | 0.00 | 0.00 | 0.00 | 0.00 | 0.00 | 0.00 | 0.00 | 0.00 | 0.00 | 0.00 | 0.00 | 7.69 | 0.00 |
| 4h4f | 80.85 | 14.89 | 91.49 | 93.62 | 17.02 | 8.51 | 4.26 | 72.34 | 8.51 | 0.00 | 8.51 | 12.77 | 10.64 | 0.00 | 23.40 | 0.00 | 0.00 | 0.00 | 23.40 | 19.15 |
| 4hom | 0.00 | 0.00 | 0.00 | 0.00 | 0.00 | 3.61 | 0.00 | 0.00 | 0.00 | 0.00 | 0.00 | 0.00 | 0.00 | 0.00 | 0.00 | 0.00 | 0.00 | 0.00 | 0.00 | 0.00 |
| 4htp | 0.00 | 0.00 | 0.00 | 0.00 | 0.00 | 0.00 | 0.00 | 0.00 | 0.00 | 0.00 | 0.00 | 0.00 | 0.00 | 6.67 | 10.00 | 0.00 | 0.00 | 0.00 | 3.33 | 0.00 |
| 4iim | 55.17 | 55.17 | 62.07 | 0.00 | 44.83 | 41.38 | 44.83 | 0.00 | 13.79 | 6.90 | 13.79 | 17.24 | 6.90 | 13.79 | 13.79 | 17.24 | 13.79 | 17.24 | 17.24 | 100.00 |
| 4j8s | 0.00 | 0.00 | 0.00 | 0.00 | 0.00 | 0.00 | 0.00 | 0.00 | 0.00 | 0.00 | 0.00 | 0.00 | 0.00 | 0.00 | 0.00 | 0.00 | 0.00 | 0.00 | 0.00 | 0.00 |
| 4k0u | 97.78 | 88.89 | 93.33 | 91.11 | 68.89 | 15.56 | 33.33 | 37.78 | 46.67 | 11.11 | 13.33 | 44.44 | 11.11 | 11.11 | 11.11 | 11.11 | 35.56 | 35.56 | 15.56 | 35.56 |

**S3(b). FNAT values of all 20 poses obtained after blind docking by Hex software on 133 protein-peptide complexes.**

| **ID** | **Pose1** | **Pose2** | **Pose3** | **Pose4** | **Pose5** | **Pose6** | **Pose7** | **Pose8** | **Pose9** | **Pose10** | **Pose11** | **Pose12** | **Pose13** | **Pose14** | **Pose15** | **Pose16** | **Pose17** | **Pose18** | **Pose19** | **Pose20** |
| --- | --- | --- | --- | --- | --- | --- | --- | --- | --- | --- | --- | --- | --- | --- | --- | --- | --- | --- | --- | --- |
| 1cjr | 16.33 | 0.00 | 40.82 | 16.33 | 95.92 | 16.33 | 24.49 | 12.24 | 12.24 | 8.16 | 14.29 | 75.51 | 22.45 | 18.37 | 0.00 | 40.82 | 12.24 | 32.65 | 14.29 | 0.00 |
| 1cka | 0.00 | NA | NA | NA | NA | NA | NA | NA | NA | NA | NA | NA | NA | NA | NA | NA | NA | NA | NA | NA |
| 1cvu | 0.00 | 0.00 | 0.00 | 0.00 | 0.00 | 0.00 | 0.00 | 0.00 | 0.00 | 0.00 | 0.00 | 0.00 | 0.00 | 0.00 | 0.00 | 0.00 | 0.00 | 0.00 | 0.00 | 0.00 |
| 1d4t | 10.71 | 71.43 | 12.50 | 12.50 | 12.50 | 3.57 | 0.00 | 10.71 | 39.29 | 0.00 | 8.93 | 0.00 | 1.79 | 25.00 | 0.00 | 55.36 | 0.00 | 35.71 | 3.57 | 10.71 |
| 1eg4 | 3.03 | 0.00 | 0.00 | 0.00 | 0.00 | 3.03 | 3.03 | 0.00 | 0.00 | 0.00 | 0.00 | 18.18 | 3.03 | 3.03 | 0.00 | 3.03 | 3.03 | 0.00 | 0.00 | 0.00 |
| 1h6w | 0.00 | 0.00 | 0.00 | 0.00 | 0.00 | 0.00 | 0.00 | 0.00 | 0.00 | 0.00 | 0.00 | 0.00 | 0.00 | 0.00 | 0.00 | 0.00 | 0.00 | 0.00 | 0.00 | 0.00 |
| 1hc9 | 0.00 | 0.00 | 0.00 | 0.00 | 0.00 | 0.00 | 0.00 | 0.00 | 0.00 | 0.00 | 0.00 | 0.00 | 0.00 | 0.00 | 0.00 | 0.00 | 0.00 | 0.00 | 0.00 | 0.00 |
| 1jbu | 22.95 | 0.00 | 1.64 | 0.00 | 0.00 | 1.64 | 0.00 | 0.00 | 0.00 | 0.00 | 0.00 | 0.00 | 6.56 | 0.00 | 21.31 | 0.00 | 6.56 | 0.00 | 1.64 | 13.11 |
| 1k5n | 18.52 | 3.70 | 0.00 | 11.11 | 11.11 | 0.00 | 3.70 | 1.85 | 5.56 | 11.11 | 5.56 | 0.00 | 16.67 | 11.11 | 3.70 | 5.56 | 0.00 | 0.00 | 0.00 | 0.00 |
| 1mfg | 0.00 | 0.00 | 8.82 | 0.00 | 2.94 | 0.00 | 0.00 | 2.94 | 0.00 | 5.88 | 0.00 | 35.29 | 0.00 | 0.00 | 0.00 | 5.88 | 5.88 | 0.00 | 0.00 | 0.00 |
| 1nln | 0.00 | 0.00 | 0.00 | 0.00 | 0.00 | 0.00 | 0.00 | 0.00 | 0.00 | 0.00 | 0.00 | 0.00 | 0.00 | 0.00 | 0.00 | 0.00 | 0.00 | 0.00 | 0.00 | 0.00 |
| 1nq7 | 3.03 | 0.00 | 0.00 | 0.00 | 18.18 | 0.00 | 12.12 | 0.00 | 0.00 | 0.00 | 0.00 | 0.00 | 15.15 | 0.00 | 0.00 | 0.00 | 21.21 | 0.00 | 0.00 | 33.33 |
| 1ntv | 0.00 | 4.35 | 13.04 | 21.74 | 30.43 | 4.35 | 0.00 | 15.22 | 19.57 | 23.91 | 8.70 | 10.87 | 4.35 | 0.00 | 4.35 | 0.00 | 0.00 | 0.00 | 0.00 | 0.00 |
| 1nx1 | 0.00 | 0.00 | 0.00 | 0.00 | 0.00 | 0.00 | 0.00 | 0.00 | 0.00 | 0.00 | 0.00 | 0.00 | 0.00 | 0.00 | 0.00 | 0.00 | 0.00 | 0.00 | 0.00 | 0.00 |
| 1oai | 0.00 | 0.00 | 0.00 | 6.45 | 9.68 | 0.00 | 0.00 | 3.23 | 0.00 | 0.00 | 16.13 | 3.23 | 0.00 | 0.00 | 0.00 | 6.45 | 0.00 | 0.00 | 0.00 | 0.00 |
| 1oj5 | 0.00 | 0.00 | 0.00 | 0.00 | 0.00 | 0.00 | 0.00 | 0.00 | 0.00 | 0.00 | 0.00 | 0.00 | 0.00 | 0.00 | 0.00 | 0.00 | 0.00 | 0.00 | 0.00 | 0.00 |
| 1ou8 | 0.00 | 0.00 | 0.00 | 0.00 | 0.00 | 0.00 | 0.00 | 0.00 | 0.00 | 0.00 | 0.00 | 0.00 | 0.00 | 0.00 | 0.00 | 0.00 | 0.00 | 0.00 | 0.00 | 0.00 |
| 1ow6 | 0.00 | 0.00 | 0.00 | 0.00 | 0.00 | 0.00 | 0.00 | 0.00 | 0.00 | 0.00 | 0.00 | 0.00 | 0.00 | 0.00 | 0.00 | 0.00 | 0.00 | 0.00 | 0.00 | 0.00 |
| 1pzl | 0.00 | 0.00 | 0.00 | 0.00 | 0.00 | 0.00 | 0.00 | 0.00 | 0.00 | 0.00 | 0.00 | 0.00 | 0.00 | 0.00 | 0.00 | 0.00 | 0.00 | 0.00 | 0.00 | 0.00 |
| 1qkz | 0.00 | 0.00 | 0.00 | 0.00 | 0.00 | 0.00 | 0.00 | 0.00 | 0.00 | 0.00 | 0.00 | 0.00 | 0.00 | 0.00 | 0.00 | 0.00 | 0.00 | 0.00 | 0.00 | 0.00 |
| 1rst | 0.00 | 0.00 | 0.00 | 0.00 | 0.00 | 0.00 | 0.00 | 0.00 | 0.00 | 0.00 | 0.00 | 0.00 | 0.00 | 0.00 | 0.00 | 0.00 | 0.00 | 0.00 | 0.00 | 0.00 |
| 1rxz | 0.00 | 0.00 | 0.00 | 0.00 | 0.00 | 0.00 | 0.00 | 0.00 | 0.00 | 0.00 | 0.00 | 0.00 | 0.00 | 0.00 | 0.00 | 0.00 | 0.00 | 0.00 | 0.00 | 0.00 |
| 1sfi | 0.00 | 0.00 | 0.00 | 0.00 | 0.00 | 0.00 | 0.00 | 0.00 | 0.00 | 0.00 | 0.00 | 0.00 | 0.00 | 0.00 | 0.00 | 0.00 | 0.00 | 0.00 | 0.00 | 0.00 |
| 1ssh | 3.33 | 6.67 | 6.67 | 0.00 | 20.00 | 6.67 | 10.00 | 6.67 | 16.67 | 0.00 | 0.00 | 20.00 | 20.00 | 6.67 | 20.00 | 3.33 | 0.00 | 10.00 | 0.00 | 0.00 |
| 1t08 | 0.00 | 0.00 | 0.00 | 10.45 | 0.00 | 10.45 | 0.00 | 0.00 | 0.00 | 0.00 | 0.00 | 0.00 | 1.49 | 0.00 | 0.00 | 11.94 | 0.00 | 0.00 | 0.00 | 0.00 |
| 1t4f | 25.00 | 100.00 | 25.00 | 17.86 | 21.43 | 21.43 | 21.43 | 28.57 | 17.86 | 32.14 | 10.71 | 21.43 | 67.86 | 7.14 | 35.71 | 10.71 | 14.29 | 35.71 | 25.00 | 7.14 |
| 1t7r | 0.00 | 0.00 | 0.00 | 0.00 | 0.00 | 0.00 | 0.00 | 0.00 | 0.00 | 0.00 | 0.00 | 0.00 | 0.00 | 0.00 | 0.00 | 0.00 | 0.00 | 0.00 | 0.00 | 0.00 |
| 1tfc | 6.45 | 0.00 | 3.23 | 0.00 | 0.00 | 0.00 | 0.00 | 0.00 | 0.00 | 0.00 | 0.00 | 0.00 | 0.00 | 0.00 | 0.00 | 0.00 | 0.00 | 0.00 | 0.00 | 0.00 |
| 1u00 | 0.00 | 0.00 | 0.00 | 0.00 | 0.00 | 0.00 | 2.08 | 0.00 | 0.00 | 0.00 | 0.00 | 0.00 | 0.00 | 2.08 | 6.25 | 4.17 | 0.00 | 0.00 | 4.17 | 0.00 |
| 1uj0 | 0.00 | 0.00 | 0.00 | 7.41 | 0.00 | 0.00 | 0.00 | 0.00 | 3.70 | 0.00 | 7.41 | 0.00 | 0.00 | 0.00 | 7.41 | 22.22 | 18.52 | 0.00 | 3.70 | 0.00 |
| 1x2r | 0.00 | 0.00 | 0.00 | 0.00 | 0.00 | 0.00 | 0.00 | 0.00 | 0.00 | 0.00 | 0.00 | 0.00 | 0.00 | 0.00 | 0.00 | 0.00 | 0.00 | 0.00 | 0.00 | 0.00 |
| 1xoc | 0.00 | 0.00 | 0.00 | 0.00 | 0.00 | 0.00 | 0.00 | 0.00 | 0.00 | 0.00 | 0.00 | 0.00 | 0.00 | 0.00 | 0.00 | 0.00 | 0.00 | 0.00 | 0.00 | 0.00 |
| 1ymt | 0.00 | 0.00 | 0.00 | 0.00 | 0.00 | 0.00 | 0.00 | 0.00 | 0.00 | 0.00 | 0.00 | 0.00 | 0.00 | 0.00 | 0.00 | 0.00 | 0.00 | 0.00 | 0.00 | 0.00 |
| 1yuc | 0.00 | 0.00 | 0.00 | 0.00 | 0.00 | 0.00 | 0.00 | 0.00 | 0.00 | 0.00 | 0.00 | 0.00 | 0.00 | 0.00 | 0.00 | 0.00 | 0.00 | 0.00 | 0.00 | 0.00 |
| 1ywo | 0.00 | 4.00 | 0.00 | 16.00 | 0.00 | 0.00 | 12.00 | 12.00 | 4.00 | 0.00 | 4.00 | 16.00 | 4.00 | 0.00 | 0.00 | 4.00 | 0.00 | 12.00 | 0.00 | 4.00 |
| 2a25 | 0.00 | 0.00 | 0.00 | 0.00 | 0.00 | 0.00 | 0.00 | 0.00 | 0.00 | 0.00 | 0.00 | 0.00 | 0.00 | 0.00 | 0.00 | 0.00 | 0.00 | 0.00 | 0.00 | 0.00 |
| 2a3i | 0.00 | 0.00 | 0.00 | 0.00 | 0.00 | 0.00 | 0.00 | 0.00 | 0.00 | 0.00 | 0.00 | 0.00 | 0.00 | 0.00 | 0.00 | 0.00 | 0.00 | 0.00 | 0.00 | 0.00 |
| 2aq9 | 0.00 | 0.00 | 0.00 | 0.00 | 0.00 | 0.00 | 0.00 | 0.00 | 0.00 | 0.00 | 0.00 | 0.00 | 0.00 | 0.00 | 0.00 | 0.00 | 0.00 | 0.00 | 0.00 | 0.00 |
| 2b9h | 0.00 | 2.04 | 0.00 | 40.82 | 12.24 | 0.00 | 2.04 | 2.04 | 6.12 | 0.00 | 0.00 | 0.00 | 0.00 | 0.00 | 6.12 | 0.00 | 14.29 | 0.00 | 6.12 | 0.00 |
| 2bba | 0.00 | 0.00 | 0.00 | 0.00 | 0.00 | 0.00 | 0.00 | 0.00 | 0.00 | 0.00 | 0.00 | 0.00 | 0.00 | 0.00 | 0.00 | 0.00 | 0.00 | 0.00 | 0.00 | 0.00 |
| 2cch | 0.00 | 0.00 | 0.00 | 0.00 | 0.00 | 4.65 | 27.91 | 6.98 | 46.51 | 0.00 | 0.00 | 0.00 | 0.00 | 2.33 | 0.00 | 0.00 | 0.00 | 20.93 | 0.00 | 0.00 |
| 2ce8 | 0.00 | 0.00 | 0.00 | 0.00 | 0.00 | 0.00 | 0.00 | 0.00 | 0.00 | 0.00 | 0.00 | 0.00 | 0.00 | 0.00 | 0.00 | 0.00 | 0.00 | 0.00 | 0.00 | 0.00 |
| 2d0n | 16.00 | 4.00 | 12.00 | 16.00 | 4.00 | 40.00 | 0.00 | 16.00 | 4.00 | 0.00 | 0.00 | 0.00 | 0.00 | 20.00 | 0.00 | 32.00 | 20.00 | 28.00 | 0.00 | 36.00 |
| 2drk | 3.33 | 0.00 | 100.00 | 16.67 | 0.00 | 13.33 | 0.00 | 3.33 | 3.33 | 93.33 | 0.00 | 16.67 | 93.33 | 0.00 | 3.33 | 30.00 | 23.33 | 3.33 | 0.00 | 20.00 |
| 2dyp | 1.54 | 6.15 | 16.92 | 0.00 | 0.00 | 3.08 | 6.15 | 9.23 | 1.54 | 6.15 | 0.00 | 20.00 | 1.54 | 0.00 | 1.54 | 0.00 | 3.08 | 0.00 | 0.00 | 7.69 |
| 2fff | 0.00 | 0.00 | 0.00 | 0.00 | 0.00 | 0.00 | 0.00 | 0.00 | 0.00 | 0.00 | 0.00 | 0.00 | 0.00 | 0.00 | 0.00 | 0.00 | 0.00 | 0.00 | 0.00 | 0.00 |
| 2ffu | 0.00 | 2.56 | 7.69 | 10.26 | 17.95 | 17.95 | 20.51 | 17.95 | 15.38 | 5.13 | 23.08 | 25.64 | 0.00 | 10.26 | 12.82 | 10.26 | 0.00 | 2.56 | 2.56 | 0.00 |
| 2fka | 0.00 | 0.00 | 0.00 | 0.00 | 0.00 | 0.00 | 0.00 | 0.00 | 0.00 | 0.00 | 0.00 | 0.00 | 0.00 | 0.00 | 0.00 | 0.00 | 0.00 | 0.00 | 0.00 | 0.00 |
| 2fmf | 0.00 | 0.00 | 0.00 | 0.00 | 0.00 | 0.00 | 0.00 | 0.00 | 0.00 | 0.00 | 0.00 | 0.00 | 0.00 | 0.00 | 0.00 | 0.00 | 0.00 | 0.00 | 0.00 | 0.00 |
| 2fts | 0.00 | 0.00 | 0.00 | 0.00 | 0.00 | 0.00 | 0.00 | 0.00 | 5.66 | 0.00 | 0.00 | 0.00 | 0.00 | 0.00 | 0.00 | 0.00 | 0.00 | 0.00 | 0.00 | 0.00 |
| 2fvj | 21.88 | 0.00 | 0.00 | 0.00 | 0.00 | 0.00 | 0.00 | 3.13 | 0.00 | 0.00 | 21.88 | 0.00 | 34.38 | 0.00 | 0.00 | 9.38 | 0.00 | 0.00 | 0.00 | 0.00 |
| 2ho2 | 5.88 | 0.00 | 0.00 | 0.00 | 0.00 | 0.00 | 5.88 | 0.00 | 0.00 | 23.53 | 0.00 | 0.00 | 23.53 | 23.53 | 47.06 | 5.88 | 0.00 | 0.00 | 5.88 | 0.00 |
| 2ht9 | 0.00 | 0.00 | 0.00 | 0.00 | 0.00 | 0.00 | 0.00 | 0.00 | 0.00 | 0.00 | 0.00 | 0.00 | 0.00 | 0.00 | 0.00 | 0.00 | 0.00 | 0.00 | 0.00 | 0.00 |
| 2o02 | 95.77 | 12.68 | 8.45 | 12.68 | 74.65 | 0.00 | 9.86 | 19.72 | 2.82 | 38.03 | 23.94 | 0.00 | 35.21 | 76.06 | 8.45 | 12.68 | 2.82 | 21.13 | 14.08 | 11.27 |
| 2o4j | 0.00 | 0.00 | 0.00 | 0.00 | 0.00 | 0.00 | 0.00 | 0.00 | 0.00 | 0.00 | 0.00 | 0.00 | 0.00 | 0.00 | 0.00 | 0.00 | 0.00 | 0.00 | 0.00 | 0.00 |
| 2o9v | 0.00 | 0.00 | 0.00 | 0.00 | 0.00 | 4.00 | 20.00 | 0.00 | 0.00 | 0.00 | 4.00 | 4.00 | 0.00 | 0.00 | 0.00 | 0.00 | 20.00 | 0.00 | 0.00 | 0.00 |
| 2oei | 0.00 | 0.00 | 0.00 | 0.00 | 0.00 | 0.00 | 0.00 | 0.00 | 0.00 | 27.27 | 27.27 | 36.36 | 0.00 | 4.55 | 4.55 | 22.73 | 0.00 | 0.00 | 18.18 | 72.73 |
| 2p0w | 0.00 | 0.00 | 0.00 | 0.00 | 0.00 | 0.00 | 0.00 | 0.00 | 0.00 | 0.00 | 0.00 | 0.00 | 0.00 | 0.00 | 0.00 | 0.00 | 0.00 | 0.00 | 0.00 | 0.00 |
| 2p1o | 0.00 | 0.00 | 0.00 | 0.00 | 0.00 | 0.00 | 0.00 | 0.00 | 0.00 | 0.00 | 0.00 | 0.00 | 0.00 | 0.00 | 0.00 | 0.00 | 0.00 | 0.00 | 0.00 | 0.00 |
| 2p1t | 5.71 | 5.71 | 0.00 | 0.00 | 5.71 | 2.86 | 0.00 | 0.00 | 0.00 | 0.00 | 8.57 | 2.86 | 17.14 | 0.00 | 2.86 | 14.29 | 8.57 | 5.71 | 0.00 | 0.00 |
| 2p54 | 0.00 | 0.00 | 5.26 | 0.00 | 0.00 | 0.00 | 10.53 | 0.00 | 0.00 | 0.00 | 0.00 | 0.00 | 2.63 | 0.00 | 7.89 | 0.00 | 2.63 | 10.53 | 0.00 | 0.00 |
| 2peh | 47.06 | 0.00 | 5.88 | 0.00 | 2.94 | 0.00 | 0.00 | 5.88 | 8.82 | 0.00 | 0.00 | 5.88 | 8.82 | 0.00 | 2.94 | 0.00 | 8.82 | 2.94 | 0.00 | 8.82 |
| 2pux | 0.00 | 21.62 | 0.00 | 0.00 | 5.41 | 0.00 | 0.00 | 0.00 | 0.00 | 2.70 | 8.11 | 0.00 | 0.00 | 0.00 | 0.00 | 0.00 | 0.00 | 8.11 | 91.89 | 0.00 |
| 2puy | 0.00 | 0.00 | 0.00 | 0.00 | 0.00 | 0.00 | 0.00 | 0.00 | 0.00 | 0.00 | 0.00 | 0.00 | 0.00 | 0.00 | 0.00 | 0.00 | 0.00 | 0.00 | 0.00 | 0.00 |
| 2qbx | 100.00 | 33.90 | 0.00 | 74.58 | 16.95 | 11.86 | 0.00 | 0.00 | 18.64 | 0.00 | 0.00 | 25.42 | 0.00 | 0.00 | 10.17 | 0.00 | 81.36 | 0.00 | 3.39 | 1.69 |
| 2qos | 0.00 | 0.00 | 0.00 | 0.00 | 0.00 | 0.00 | 0.00 | 0.00 | 0.00 | 0.00 | 0.00 | 0.00 | 0.00 | 0.00 | 0.00 | 0.00 | 0.00 | 0.00 | 0.00 | 0.00 |
| 2qse | 0.00 | 0.00 | 0.00 | 0.00 | 0.00 | 0.00 | 0.00 | 0.00 | 0.00 | 0.00 | 0.00 | 0.00 | 0.00 | 0.00 | 0.00 | 0.00 | 0.00 | 0.00 | 0.00 | 0.00 |
| 2r7g | 10.64 | 6.38 | 2.13 | 0.00 | 6.38 | 4.26 | 2.13 | 2.13 | 8.51 | 19.15 | 2.13 | 0.00 | 6.38 | 2.13 | 0.00 | 0.00 | 0.00 | 0.00 | 0.00 | 0.00 |
| 2r9q | 0.00 | 0.00 | 0.00 | 0.00 | 0.00 | 0.00 | 0.00 | 0.00 | 0.00 | 0.00 | 0.00 | 0.00 | 0.00 | 0.00 | 0.00 | 0.00 | 0.00 | 0.00 | 0.00 | 0.00 |
| 2v8y | 0.00 | 14.63 | 0.00 | 0.00 | 0.00 | 0.00 | 0.00 | 0.00 | 0.00 | 0.00 | 0.00 | 12.20 | 0.00 | 0.00 | 0.00 | 0.00 | 0.00 | 0.00 | 2.44 | 2.44 |
| 2vkn | 0.00 | 0.00 | 0.00 | 0.00 | 0.00 | 0.00 | 0.00 | 27.27 | 31.82 | 18.18 | 9.09 | 0.00 | 0.00 | 0.00 | 4.55 | 0.00 | 0.00 | 0.00 | 0.00 | 0.00 |
| 2vr3 | 0.00 | 0.00 | 0.00 | 0.00 | 0.00 | 0.00 | 0.00 | 0.00 | 0.00 | 0.00 | 0.00 | 0.00 | 0.00 | 0.00 | 0.00 | 0.00 | 0.00 | 0.00 | 0.00 | 0.00 |
| 2vwf | 2.86 | 11.43 | 0.00 | 8.57 | 0.00 | 0.00 | 0.00 | 0.00 | 0.00 | 0.00 | 0.00 | 0.00 | 8.57 | 8.57 | 0.00 | 8.57 | 5.71 | 20.00 | 25.71 | 0.00 |
| 2w2u | 0.00 | 0.00 | 8.51 | 0.00 | 0.00 | 0.00 | 0.00 | 6.38 | 0.00 | 17.02 | 0.00 | 0.00 | 0.00 | 0.00 | 4.26 | 0.00 | 4.26 | 6.38 | 8.51 | 0.00 |
| 2whx | 0.00 | 0.00 | 1.23 | 1.23 | 0.00 | 0.00 | 0.00 | 0.00 | 0.00 | 0.00 | 0.00 | 0.00 | 0.00 | 0.00 | 1.23 | 0.00 | 0.00 | 0.00 | 0.00 | 0.00 |
| 2xrw | 0.00 | 0.00 | 0.00 | 0.00 | 0.00 | 5.00 | 0.00 | 0.00 | 7.50 | 0.00 | 0.00 | 0.00 | 0.00 | 0.00 | 10.00 | 0.00 | 5.00 | 0.00 | 0.00 | 5.00 |
| 2xu7 | 95.92 | 0.00 | 0.00 | 8.16 | 0.00 | 0.00 | 0.00 | 2.04 | 0.00 | 0.00 | 0.00 | 0.00 | 0.00 | 2.04 | 0.00 | 0.00 | 0.00 | 0.00 | 40.82 | 0.00 |
| 2xvc | 9.80 | 5.88 | 21.57 | 17.65 | 0.00 | 3.92 | 13.73 | 21.57 | 15.69 | 11.76 | 11.76 | 21.57 | 15.69 | 0.00 | 5.88 | 15.69 | 15.69 | 11.76 | 17.65 | 7.84 |
| 2zjd | 0.00 | 0.00 | 0.00 | 0.00 | 0.00 | 0.00 | 0.00 | 0.00 | 0.00 | 0.00 | 0.00 | 0.00 | 0.00 | 0.00 | 0.00 | 0.00 | 0.00 | 0.00 | 0.00 | 0.00 |
| 3asl | 0.00 | 9.68 | 0.00 | 3.23 | 0.00 | 16.13 | 0.00 | 0.00 | 0.00 | 19.35 | 0.00 | 0.00 | 45.16 | 19.35 | 0.00 | 3.23 | 35.48 | 0.00 | 0.00 | 12.90 |
| 3awr | 22.58 | 0.00 | 6.45 | 16.13 | 0.00 | 0.00 | 96.77 | 19.35 | 6.45 | 0.00 | 3.23 | 0.00 | 9.68 | 38.71 | 0.00 | 16.13 | 6.45 | 0.00 | 0.00 | 19.35 |
| 3ayu | 96.67 | 51.67 | 68.33 | 10.00 | 0.00 | 13.33 | 0.00 | 10.00 | 0.00 | 16.67 | 46.67 | 11.67 | 10.00 | 0.00 | 11.67 | 5.00 | 0.00 | 15.00 | 10.00 | 23.33 |
| 3bfq | 92.71 | 9.38 | 96.88 | 77.08 | 44.79 | 8.33 | 17.71 | 12.50 | 10.42 | 15.63 | 8.33 | 10.42 | 79.17 | 14.58 | 3.13 | 14.58 | 8.33 | 15.63 | 20.83 | 11.46 |
| 3c3r | 0.00 | 0.00 | 0.00 | 0.00 | 0.00 | 0.00 | 0.00 | 0.00 | 0.00 | 0.00 | 0.00 | 0.00 | 0.00 | 0.00 | 0.00 | 0.00 | 0.00 | 0.00 | 0.00 | 0.00 |
| 3d32 | 0.00 | 0.00 | 0.00 | 0.00 | 0.00 | 0.00 | 0.00 | 0.00 | 0.00 | 0.00 | 0.00 | 0.00 | 0.00 | 0.00 | 0.00 | 0.00 | 0.00 | 0.00 | 0.00 | 0.00 |
| 3ds4 | 100.00 | 28.21 | 20.51 | 58.97 | 7.69 | 7.69 | 15.38 | 10.26 | 15.38 | 17.95 | 20.51 | 2.56 | 17.95 | 25.64 | 17.95 | 17.95 | 20.51 | 20.51 | 87.18 | 17.95 |
| 3ery | 18.75 | 89.06 | 20.31 | 17.19 | 15.63 | 68.75 | 4.69 | 65.63 | 85.94 | 12.50 | 67.19 | 17.19 | 26.56 | 9.38 | 10.94 | 17.19 | 17.19 | 10.94 | 34.38 | 1.56 |
| 3fdo | 0.00 | 6.67 | 26.67 | 20.00 | 6.67 | 10.00 | 100.00 | 13.33 | 0.00 | 0.00 | 0.00 | 0.00 | 20.00 | 10.00 | 3.33 | 13.33 | 83.33 | 30.00 | 10.00 | 6.67 |
| 3g2s | 100.00 | 0.00 | 0.00 | 18.42 | 0.00 | 18.42 | 0.00 | 0.00 | 2.63 | 10.53 | 13.16 | 2.63 | 2.63 | 0.00 | 0.00 | 0.00 | 0.00 | 0.00 | 13.16 | 0.00 |
| 3gyt | 0.00 | 0.00 | 0.00 | 0.00 | 0.00 | 0.00 | 0.00 | 0.00 | 6.90 | 0.00 | 0.00 | 0.00 | 0.00 | 0.00 | 0.00 | 0.00 | 0.00 | 0.00 | 3.45 | 0.00 |
| 3h1z | 0.00 | 1.89 | 0.00 | 0.00 | 0.00 | 0.00 | 16.98 | 0.00 | 0.00 | 0.00 | 0.00 | 0.00 | 0.00 | 0.00 | 0.00 | 0.00 | 0.00 | 0.00 | 0.00 | 0.00 |
| 3i5r | 3.85 | 7.69 | 0.00 | 0.00 | 3.85 | 0.00 | 0.00 | 0.00 | 88.46 | 0.00 | 15.38 | 19.23 | 15.38 | 61.54 | 0.00 | 0.00 | 30.77 | 0.00 | 0.00 | 3.85 |
| 3ivv | 0.00 | 0.00 | 0.00 | 58.82 | 0.00 | 41.18 | 0.00 | 11.76 | 0.00 | 41.18 | 0.00 | 0.00 | 0.00 | 0.00 | 32.35 | 17.65 | 0.00 | 0.00 | 23.53 | 0.00 |
| 3kmr | 0.00 | 10.00 | 0.00 | 0.00 | 3.33 | 3.33 | 3.33 | 13.33 | 26.67 | 3.33 | 0.00 | 0.00 | 0.00 | 0.00 | 0.00 | 0.00 | 16.67 | 0.00 | 0.00 | 6.67 |
| 3kuj | 100.00 | 93.18 | 0.00 | 4.55 | 6.82 | 0.00 | 25.00 | 4.55 | 2.27 | 4.55 | 0.00 | 11.36 | 13.64 | 22.73 | 45.45 | 0.00 | 20.45 | 22.73 | 88.64 | 4.55 |
| 3kus | 0.00 | 0.00 | 22.22 | 0.00 | 0.00 | 22.22 | 15.56 | 28.89 | 28.89 | 28.89 | 11.11 | 22.22 | 2.22 | 0.00 | 0.00 | 42.22 | 8.89 | 28.89 | 28.89 | 0.00 |
| 3l0e | 0.00 | 0.00 | 0.00 | 0.00 | 4.88 | 0.00 | 17.07 | 0.00 | 0.00 | 0.00 | 0.00 | 0.00 | 0.00 | 0.00 | 0.00 | 0.00 | 2.44 | 0.00 | 0.00 | 0.00 |
| 3ll8 | 0.00 | 0.00 | 0.00 | 0.00 | 2.33 | 0.00 | 0.00 | 0.00 | 0.00 | 0.00 | 0.00 | 0.00 | 0.00 | 0.00 | 0.00 | 0.00 | 0.00 | 0.00 | 0.00 | 0.00 |
| 3llz | 100.00 | 0.00 | 0.00 | 0.00 | 0.00 | 25.64 | 0.00 | 0.00 | 0.00 | 0.00 | 0.00 | 71.79 | 0.00 | 0.00 | 15.38 | 41.03 | 0.00 | 10.26 | 0.00 | 0.00 |
| 3obq | 0.00 | 0.00 | 0.00 | 0.00 | 16.67 | 2.78 | 0.00 | 16.67 | 16.67 | 0.00 | 0.00 | 0.00 | 0.00 | 25.00 | 0.00 | 2.78 | 0.00 | 0.00 | 25.00 | 0.00 |
| 3olf | 0.00 | 0.00 | 21.88 | 96.88 | 0.00 | 3.13 | 0.00 | 0.00 | 0.00 | 0.00 | 6.25 | 28.13 | 0.00 | 0.00 | 0.00 | 0.00 | 0.00 | 0.00 | 9.38 | 3.13 |
| 3p72 | 0.00 | 0.00 | 0.00 | 0.00 | 0.00 | 0.00 | 0.00 | 0.00 | 0.00 | 0.00 | 0.00 | 0.00 | 0.00 | 0.00 | 0.00 | 0.00 | 0.00 | 0.00 | 0.00 | 0.00 |
| 3p8f | 5.88 | 2.94 | 17.65 | 0.00 | 2.94 | 0.00 | 13.24 | 0.00 | 0.00 | 0.00 | 1.47 | 0.00 | 5.88 | 0.00 | 0.00 | 0.00 | 22.06 | 5.88 | 1.47 | 29.41 |
| 3ptl | 0.00 | 0.00 | 0.00 | 0.00 | 0.00 | 13.24 | 0.00 | 0.00 | 0.00 | 2.94 | 13.24 | 0.00 | 1.47 | 0.00 | 0.00 | 0.00 | 0.00 | 0.00 | 0.00 | 0.00 |
| 3qis | 14.71 | 20.59 | 2.94 | 8.82 | 26.47 | 11.76 | 8.82 | 14.71 | 20.59 | 8.82 | 0.00 | 2.94 | 11.76 | 2.94 | 0.00 | 2.94 | 20.59 | 5.88 | 0.00 | 0.00 |
| 3rm1 | 94.44 | 11.11 | 13.89 | 33.33 | 27.78 | 0.00 | 13.89 | 16.67 | 22.22 | 0.00 | 2.78 | 16.67 | 38.89 | 11.11 | 0.00 | 25.00 | 0.00 | 0.00 | 11.11 | 2.78 |
| 3rqg | 0.00 | 0.00 | 0.00 | 0.00 | 0.00 | 0.00 | 0.00 | 0.00 | 0.00 | 0.00 | 0.00 | 0.00 | 0.00 | 0.00 | 0.00 | 0.00 | 0.00 | 0.00 | 0.00 | 0.00 |
| 3sfj | 0.00 | 0.00 | 0.00 | 0.00 | 2.27 | 6.82 | 0.00 | 0.00 | 2.27 | 0.00 | 0.00 | 0.00 | 0.00 | 0.00 | 13.64 | 0.00 | 0.00 | 2.27 | 0.00 | 0.00 |
| 3so6 | 0.00 | 0.00 | 0.00 | 0.00 | 0.00 | 0.00 | 0.00 | 0.00 | 0.00 | 0.00 | 0.00 | 0.00 | 0.00 | 0.00 | 0.00 | 0.00 | 0.00 | 0.00 | 0.00 | 0.00 |
| 3tjv | 95.31 | 0.00 | 0.00 | 0.00 | 0.00 | 0.00 | 10.94 | 32.81 | 0.00 | 12.50 | 0.00 | 10.94 | 20.31 | 53.13 | 6.25 | 0.00 | 0.00 | 7.81 | 0.00 | 1.56 |
| 3tzy | 0.00 | 0.00 | 0.00 | 0.00 | 0.00 | 0.00 | 0.00 | 0.00 | 0.00 | 0.00 | 0.00 | 0.00 | 0.00 | 0.00 | 0.00 | 0.00 | 0.00 | 0.00 | 0.00 | 0.00 |
| 3u9q | 100.00 | 0.00 | 39.39 | 0.00 | 0.00 | 0.00 | 30.30 | 0.00 | 18.18 | 39.39 | 0.00 | 0.00 | 0.00 | 0.00 | 21.21 | 0.00 | 0.00 | 0.00 | 0.00 | 0.00 |
| 3up3 | 2.78 | 0.00 | 0.00 | 0.00 | 0.00 | 91.67 | 0.00 | 0.00 | 0.00 | 13.89 | 11.11 | 0.00 | 0.00 | 100.00 | 0.00 | 8.33 | 0.00 | 0.00 | 0.00 | 13.89 |
| 3v2x | 28.89 | 13.33 | 2.22 | 0.00 | 97.78 | 0.00 | 2.22 | 17.78 | 0.00 | 22.22 | 17.78 | 8.89 | 44.44 | 8.89 | 55.56 | 2.22 | 20.00 | 0.00 | 20.00 | 0.00 |
| 3vtc | 0.00 | 9.09 | 0.00 | 0.00 | 0.00 | 0.00 | 0.00 | 0.00 | 0.00 | 0.00 | 0.00 | 0.00 | 0.00 | 3.03 | 0.00 | 0.00 | 0.00 | 6.06 | 0.00 | 0.00 |
| 3w1b | 0.00 | 0.00 | 0.00 | 0.00 | 0.00 | 0.00 | 0.00 | 0.00 | 0.00 | 0.00 | 0.00 | 0.00 | 0.00 | 0.00 | 0.00 | 0.00 | 0.00 | 0.00 | 0.00 | 0.00 |
| 3zqh | 0.00 | 0.00 | 0.00 | 0.00 | 3.23 | 0.00 | 0.00 | 0.00 | 3.23 | 6.45 | 3.23 | 0.00 | 0.00 | 0.00 | 0.00 | 0.00 | 12.90 | 19.35 | 41.94 | 0.00 |
| 4b4n | 0.00 | 0.00 | 17.07 | 0.00 | 24.39 | 0.00 | 0.00 | 24.39 | 19.51 | 2.44 | 26.83 | 7.32 | 0.00 | 0.00 | 82.93 | 0.00 | 0.00 | 0.00 | 24.39 | 12.20 |
| 4dcb | 0.00 | 0.00 | 0.00 | 2.44 | 0.00 | 0.00 | 0.00 | 2.44 | 0.00 | 0.00 | 2.44 | 0.00 | 7.32 | 0.00 | 0.00 | 0.00 | 0.00 | 0.00 | 0.00 | 0.00 |
| 4e34 | 2.70 | 0.00 | 0.00 | 0.00 | 0.00 | 0.00 | 0.00 | 0.00 | 0.00 | 0.00 | 0.00 | 2.70 | 0.00 | 0.00 | 0.00 | 0.00 | 0.00 | 0.00 | 0.00 | 0.00 |
| 4eik | 0.00 | 0.00 | 0.00 | 0.00 | 0.00 | 0.00 | 0.00 | 0.00 | 0.00 | 0.00 | 0.00 | 0.00 | 0.00 | 0.00 | 0.00 | 0.00 | 0.00 | 0.00 | 0.00 | 0.00 |
| 4ery | 0.00 | 0.00 | 0.00 | 0.00 | 0.00 | 0.00 | 0.00 | 0.00 | 0.00 | 0.00 | 0.00 | 8.33 | 0.00 | 0.00 | 0.00 | 0.00 | 8.33 | 0.00 | 87.50 | 0.00 |
| 4f14 | 3.45 | 0.00 | 10.34 | 3.45 | 10.34 | 3.45 | 20.69 | 6.90 | 37.93 | 44.83 | 3.45 | 3.45 | 10.34 | 3.45 | 0.00 | 0.00 | 17.24 | 0.00 | 3.45 | 20.69 |
| 4f1z | 11.49 | 0.00 | 14.94 | 9.20 | 16.09 | 0.00 | 10.34 | 0.00 | 0.00 | 5.75 | 9.20 | 17.24 | 2.30 | 5.75 | 5.75 | 0.00 | 3.45 | 0.00 | 0.00 | 0.00 |
| 4gq6 | 16.07 | 28.57 | 10.71 | 5.36 | 23.21 | 28.57 | 21.43 | 10.71 | 5.36 | 17.86 | 42.86 | 23.21 | 10.71 | 5.36 | 25.00 | 1.79 | 16.07 | 8.93 | 26.79 | 14.29 |
| 4gxl | 0.00 | 0.00 | 0.00 | 21.05 | 0.00 | 0.00 | 73.68 | 0.00 | 0.00 | 15.79 | 26.32 | 0.00 | 50.00 | 0.00 | 0.00 | 0.00 | 5.26 | 0.00 | 0.00 | 0.00 |
| 4gyw | 3.85 | 25.00 | 23.08 | 5.77 | 9.62 | 9.62 | 23.08 | 0.00 | 7.69 | 0.00 | 5.77 | 1.92 | 0.00 | 0.00 | 3.85 | 7.69 | 0.00 | 0.00 | 11.54 | 0.00 |
| 4h4f | 0.00 | 0.00 | 0.00 | 0.00 | 0.00 | 0.00 | 0.00 | 0.00 | 0.00 | 0.00 | 0.00 | 0.00 | 0.00 | 0.00 | 0.00 | 0.00 | 0.00 | 0.00 | 0.00 | 0.00 |
| 4hom | 0.00 | 0.00 | 0.00 | 0.00 | 0.00 | 0.00 | 0.00 | 0.00 | 0.00 | 0.00 | 0.00 | 0.00 | 0.00 | 0.00 | 0.00 | 0.00 | 0.00 | 0.00 | 0.00 | 0.00 |
| 4htp | 0.00 | 0.00 | 0.00 | 0.00 | 0.00 | 0.00 | 3.33 | 0.00 | 0.00 | 0.00 | 0.00 | 0.00 | 26.67 | 0.00 | 0.00 | 0.00 | 0.00 | 0.00 | 0.00 | 0.00 |
| 4iim | 96.55 | 0.00 | 10.34 | 55.17 | 10.34 | 93.10 | 0.00 | 3.45 | 0.00 | 27.59 | 0.00 | 17.24 | 3.45 | 13.79 | 17.24 | 0.00 | 0.00 | 24.14 | 0.00 | 72.41 |
| 4j8s | 0.00 | 0.00 | 0.00 | 0.00 | 0.00 | 0.00 | 0.00 | 0.00 | 0.00 | 0.00 | 0.00 | 0.00 | 0.00 | 0.00 | 0.00 | 0.00 | 0.00 | 0.00 | 0.00 | 0.00 |
| 4k0u | 100.00 | 2.22 | 22.22 | 37.78 | 13.33 | 13.33 | 11.11 | 8.89 | 4.44 | 8.89 | 26.67 | 11.11 | 40.00 | 13.33 | 22.22 | 13.33 | 13.33 | 8.89 | 15.56 | 11.11 |

**S3(c) FNAT values of all 20 poses obtained after blind docking by ZDOCK on 133 protein-peptide complexes.**

| **ID** | **Pose1** | **Pose2** | **Pose3** | **Pose4** | **Pose5** | **Pose6** | **Pose7** | **Pose8** | **Pose9** | **Pose10** | **Pose11** | **Pose12** | **Pose13** | **Pose14** | **Pose15** | **Pose16** | **Pose17** | **Pose18** | **Pose19** | **Pose20** |
| --- | --- | --- | --- | --- | --- | --- | --- | --- | --- | --- | --- | --- | --- | --- | --- | --- | --- | --- | --- | --- |
| 1cjr | 81.63 | 83.67 | 89.80 | 95.92 | 0.00 | 85.71 | 0.00 | 0.00 | 0.00 | 0.00 | 0.00 | 0.00 | 65.31 | 0.00 | 0.00 | 14.29 | 0.00 | 0.00 | 0.00 | 0.00 |
| 1cka | 96.43 | 100.00 | 85.71 | 21.43 | 17.86 | 89.29 | 17.86 | 89.29 | 14.29 | 14.29 | 14.29 | 21.43 | 75.00 | 82.14 | 17.86 | 82.14 | 17.86 | 14.29 | 50.00 | 75.00 |
| 1cvu | 0.00 | 0.00 | 0.00 | 0.00 | 0.00 | 0.00 | 0.00 | 0.00 | 0.00 | 0.00 | 0.00 | 0.00 | 0.00 | 0.00 | 0.00 | 0.00 | 0.00 | 0.00 | 0.00 | 0.00 |
| 1d4t | 82.14 | 94.64 | 16.07 | 51.79 | 50.00 | 5.36 | 64.29 | 8.93 | 21.43 | 21.43 | 3.57 | 14.29 | 55.36 | 35.71 | 53.57 | 78.57 | 62.50 | 25.00 | 17.86 | 17.86 |
| 1eg4 | 0.00 | 0.00 | 0.00 | 0.00 | 0.00 | 0.00 | 0.00 | 0.00 | 0.00 | 0.00 | 0.00 | 0.00 | 0.00 | 0.00 | 0.00 | 0.00 | 0.00 | 0.00 | 0.00 | 0.00 |
| 1h6w | 100.00 | 98.59 | 90.14 | 85.92 | 90.14 | 88.73 | 95.77 | 19.72 | 19.72 | 9.86 | 80.28 | 22.54 | 19.72 | 14.08 | 18.31 | 12.68 | 14.08 | 12.68 | 26.76 | 22.54 |
| 1hc9 | 0.00 | 97.78 | 2.22 | 77.78 | 0.00 | 6.67 | 4.44 | 2.22 | 6.67 | 2.22 | 0.00 | 0.00 | 2.22 | 2.22 | 0.00 | 0.00 | 2.22 | 0.00 | 0.00 | 0.00 |
| 1jbu | 83.61 | 85.25 | 0.00 | 0.00 | 90.16 | 0.00 | 0.00 | 0.00 | 0.00 | 0.00 | 0.00 | 0.00 | 0.00 | 0.00 | 0.00 | 0.00 | 0.00 | 0.00 | 0.00 | 0.00 |
| 1k5n | 92.59 | 79.63 | 1.85 | 1.85 | 66.67 | 0.00 | 0.00 | 0.00 | 1.85 | 1.85 | 7.41 | 0.00 | 0.00 | 57.41 | 0.00 | 1.85 | 1.85 | 0.00 | 20.37 | 0.00 |
| 1mfg | 0.00 | 0.00 | 0.00 | 0.00 | 0.00 | 0.00 | 0.00 | 0.00 | 0.00 | 0.00 | 0.00 | 0.00 | 0.00 | 0.00 | 0.00 | 0.00 | 0.00 | 0.00 | 85.29 | 0.00 |
| 1nln | 0.00 | 0.00 | 0.00 | 0.00 | 0.00 | 0.00 | 0.00 | 0.00 | 0.00 | 0.00 | 0.00 | 0.00 | 0.00 | 0.00 | 0.00 | 0.00 | 0.00 | 0.00 | 0.00 | 0.00 |
| 1nq7 | 0.00 | 0.00 | 0.00 | 0.00 | 3.03 | 0.00 | 15.15 | 3.03 | 0.00 | 0.00 | 0.00 | 0.00 | 0.00 | 0.00 | 3.03 | 0.00 | 0.00 | 0.00 | 0.00 | 0.00 |
| 1ntv | 69.57 | 4.35 | 10.87 | 13.04 | 8.70 | 50.00 | 95.65 | 10.87 | 8.70 | 6.52 | 2.17 | 13.04 | 6.52 | 10.87 | 54.35 | 4.35 | 2.17 | 10.87 | 93.48 | 8.70 |
| 1nx1 | 0.00 | 0.00 | 0.00 | 0.00 | 0.00 | 0.00 | 0.00 | 0.00 | 0.00 | 0.00 | 0.00 | 0.00 | 87.50 | 0.00 | 0.00 | 0.00 | 0.00 | 0.00 | 0.00 | 0.00 |
| 1oai | 3.23 | 3.23 | 80.65 | 96.77 | 3.23 | 6.45 | 3.23 | 3.23 | 83.87 | 6.45 | 0.00 | 3.23 | 3.23 | 3.23 | 3.23 | 3.23 | 0.00 | 9.68 | 0.00 | 0.00 |
| 1oj5 | 20.00 | 28.00 | 16.00 | 28.00 | 28.00 | 64.00 | 8.00 | 44.00 | 28.00 | 32.00 | 68.00 | 20.00 | 20.00 | 36.00 | 24.00 | 12.00 | 16.00 | 32.00 | 4.00 | 4.00 |
| 1ou8 | 20.00 | 15.56 | 13.33 | 13.33 | 11.11 | 11.11 | 6.67 | 31.11 | 13.33 | 2.22 | 15.56 | 17.78 | 11.11 | 4.44 | 0.00 | 6.67 | 4.44 | 20.00 | 2.22 | 2.22 |
| 1ow6 | 0.00 | 0.00 | 0.00 | 0.00 | 0.00 | 0.00 | 0.00 | 0.00 | 0.00 | 0.00 | 0.00 | 0.00 | 0.00 | 0.00 | 0.00 | 0.00 | 0.00 | 0.00 | 0.00 | 0.00 |
| 1pzl | 0.00 | 0.00 | 0.00 | 0.00 | 0.00 | 0.00 | 0.00 | 0.00 | 0.00 | 0.00 | 0.00 | 0.00 | 0.00 | 0.00 | 0.00 | 0.00 | 0.00 | 0.00 | 0.00 | 0.00 |
| 1qkz | 20.51 | 20.51 | 17.95 | 17.95 | 17.95 | 38.46 | 20.51 | 17.95 | 17.95 | 20.51 | 17.95 | 15.38 | 17.95 | 48.72 | 51.28 | 20.51 | 20.51 | 17.95 | 15.38 | 23.08 |
| 1rst | 20.69 | 86.21 | 17.24 | 82.76 | 37.93 | 6.90 | 34.48 | 17.24 | 34.48 | 89.66 | 31.03 | 93.10 | 37.93 | 79.31 | 82.76 | 17.24 | 41.38 | 24.14 | 62.07 | 10.34 |
| 1rxz | 16.67 | 20.37 | 77.78 | 62.96 | 18.52 | 0.00 | 53.70 | 88.89 | 46.30 | 18.52 | 62.96 | 0.00 | 18.52 | 7.41 | 64.81 | 18.52 | 5.56 | 40.74 | 0.00 | 0.00 |
| 1sfi | 8.77 | 5.26 | 8.77 | 0.00 | 0.00 | 0.00 | 0.00 | 10.53 | 0.00 | 5.26 | 12.28 | 0.00 | 7.02 | 8.77 | 0.00 | 8.77 | 0.00 | 0.00 | 0.00 | 7.02 |
| 1ssh | 90.00 | 86.67 | 83.33 | 20.00 | 46.67 | 20.00 | 26.67 | 23.33 | 20.00 | 16.67 | 86.67 | 73.33 | 50.00 | 50.00 | 33.33 | 86.67 | 66.67 | 40.00 | 86.67 | 23.33 |
| 1t08 | 0.00 | 0.00 | 0.00 | 0.00 | 1.49 | 0.00 | 0.00 | 0.00 | 1.49 | 7.46 | 7.46 | 7.46 | 1.49 | 0.00 | 2.99 | 0.00 | 0.00 | 1.49 | 0.00 | 8.96 |
| 1t4f | 96.43 | 96.43 | 96.43 | 100.00 | 96.43 | 21.43 | 32.14 | 32.14 | 78.57 | 100.00 | 0.00 | 7.14 | 57.14 | 75.00 | 92.86 | 32.14 | 3.57 | 17.86 | 50.00 | 3.57 |
| 1t7r | 0.00 | 0.00 | 80.65 | 90.32 | 0.00 | 0.00 | 0.00 | 38.71 | 0.00 | 0.00 | 96.77 | 80.65 | 0.00 | 0.00 | 0.00 | 0.00 | 0.00 | 0.00 | 0.00 | 0.00 |
| 1tfc | 100.00 | 87.10 | 0.00 | 0.00 | 0.00 | 0.00 | 77.42 | 0.00 | 0.00 | 0.00 | 0.00 | 3.23 | 0.00 | 0.00 | 77.42 | 0.00 | 96.77 | 96.77 | 0.00 | 0.00 |
| 1u00 | 64.58 | 91.67 | 70.83 | 91.67 | 66.67 | 29.17 | 60.42 | 64.58 | 31.25 | 64.58 | 75.00 | 81.25 | 43.75 | 29.17 | 45.83 | 56.25 | 81.25 | 0.00 | 41.67 | 0.00 |
| 1uj0 | 96.30 | 100.00 | 92.59 | 22.22 | 88.89 | 70.37 | 70.37 | 18.52 | 22.22 | 22.22 | 22.22 | 18.52 | 14.81 | 37.04 | 14.81 | 14.81 | 14.81 | 66.67 | 74.07 | 18.52 |
| 1x2r | 23.26 | 65.12 | 37.21 | 30.23 | 39.53 | 58.14 | 6.98 | 23.26 | 62.79 | 11.63 | 20.93 | 20.93 | 13.95 | 20.93 | 16.28 | 27.91 | 76.74 | 34.88 | 4.65 | 37.21 |
| 1xoc | 1.18 | 96.47 | 0.00 | 95.29 | 0.00 | 1.18 | 0.00 | 0.00 | 2.35 | 1.18 | 0.00 | 0.00 | 0.00 | 4.71 | 0.00 | 0.00 | 0.00 | 0.00 | 0.00 | 2.35 |
| 1ymt | 100.00 | 100.00 | 25.00 | 85.71 | 96.43 | 67.86 | 96.43 | 14.29 | 78.57 | 3.57 | 7.14 | 0.00 | 7.14 | 32.14 | 0.00 | 0.00 | 96.43 | 92.86 | 7.14 | 78.57 |
| 1yuc | 94.44 | 19.44 | 8.33 | 0.00 | 0.00 | 91.67 | 2.78 | 16.67 | 0.00 | 100.00 | 2.78 | 0.00 | 11.11 | 2.78 | 0.00 | 0.00 | 13.89 | 0.00 | 2.78 | 19.44 |
| 1ywo | 36.00 | 60.00 | 60.00 | 52.00 | 24.00 | 24.00 | 32.00 | 12.00 | 44.00 | 8.00 | 44.00 | 36.00 | 60.00 | 24.00 | 52.00 | 48.00 | 24.00 | 100.00 | 32.00 | 24.00 |
| 2a25 | 0.00 | 0.00 | 0.00 | 0.00 | 0.00 | 0.00 | 0.00 | 0.00 | 0.00 | 0.00 | 0.00 | 0.00 | 0.00 | 0.00 | 60.00 | 0.00 | 0.00 | 0.00 | 0.00 | 0.00 |
| 2a3i | 0.00 | 0.00 | 0.00 | 0.00 | 0.00 | 0.00 | 0.00 | 0.00 | 0.00 | 0.00 | 0.00 | 0.00 | 0.00 | 0.00 | 0.00 | 0.00 | 0.00 | 0.00 | 0.00 | 0.00 |
| 2aq9 | 5.00 | 10.00 | 5.00 | 15.00 | 0.00 | 5.00 | 0.00 | 5.00 | 5.00 | 5.00 | 5.00 | 10.00 | 5.00 | 15.00 | 5.00 | 0.00 | 0.00 | 0.00 | 0.00 | 0.00 |
| 2b9h | 0.00 | 87.76 | 73.47 | 75.51 | 0.00 | 0.00 | 0.00 | 0.00 | 0.00 | 0.00 | 0.00 | 0.00 | 0.00 | 0.00 | 0.00 | 0.00 | 0.00 | 0.00 | 0.00 | 0.00 |
| 2bba | 0.00 | 0.00 | 0.00 | 0.00 | 0.00 | 0.00 | 0.00 | 0.00 | 0.00 | 0.00 | 0.00 | 0.00 | 0.00 | 0.00 | 0.00 | 0.00 | 0.00 | 0.00 | 0.00 | 0.00 |
| 2cch | 97.67 | 9.30 | 0.00 | 0.00 | 0.00 | 9.30 | 0.00 | 6.98 | 9.30 | 0.00 | 0.00 | 11.63 | 9.30 | 0.00 | 0.00 | 0.00 | 0.00 | 0.00 | 13.95 | 0.00 |
| 2ce8 | 0.00 | 0.00 | 0.00 | 0.00 | 0.00 | 0.00 | 0.00 | 0.00 | 0.00 | 0.00 | 0.00 | 0.00 | 0.00 | 0.00 | 0.00 | 0.00 | 0.00 | 0.00 | 0.00 | 0.00 |
| 2d0n | 100.00 | 92.00 | 100.00 | 80.00 | 88.00 | 72.00 | 72.00 | 84.00 | 76.00 | 80.00 | 20.00 | 96.00 | 24.00 | 84.00 | 96.00 | 24.00 | 92.00 | 8.00 | 0.00 | 84.00 |
| 2drk | 86.67 | 96.67 | 90.00 | 96.67 | 20.00 | 33.33 | 96.67 | 13.33 | 20.00 | 33.33 | 36.67 | 26.67 | 33.33 | 23.33 | 10.00 | 96.67 | 30.00 | 20.00 | 30.00 | 33.33 |
| 2dyp | 90.77 | 87.69 | 92.31 | 93.85 | 1.54 | 0.00 | 0.00 | 81.54 | 87.69 | 1.54 | 83.08 | 0.00 | 0.00 | 1.54 | 0.00 | 1.54 | 3.08 | 0.00 | 0.00 | 0.00 |
| 2fff | 0.00 | 0.00 | 0.00 | 0.00 | 0.00 | 0.00 | 0.00 | 0.00 | 0.00 | 0.00 | 0.00 | 0.00 | 0.00 | 0.00 | 0.00 | 0.00 | 0.00 | 0.00 | 0.00 | 0.00 |
| 2ffu | 20.51 | 87.18 | 87.18 | 17.95 | 12.82 | 17.95 | 15.38 | 92.31 | 0.00 | 17.95 | 43.59 | 87.18 | 17.95 | 71.79 | 51.28 | 41.03 | 17.95 | 74.36 | 30.77 | 69.23 |
| 2fka | 0.00 | 57.14 | 0.00 | 52.38 | 0.00 | 0.00 | 0.00 | 0.00 | 0.00 | 0.00 | 52.38 | 0.00 | 0.00 | 0.00 | 52.38 | 0.00 | 100.00 | 0.00 | 0.00 | 57.14 |
| 2fmf | 0.00 | 0.00 | 54.55 | 0.00 | 86.36 | 0.00 | 0.00 | 0.00 | 77.27 | 0.00 | 0.00 | 0.00 | 0.00 | 0.00 | 0.00 | 0.00 | 0.00 | 0.00 | 0.00 | 0.00 |
| 2fts | 100.00 | 90.57 | 84.91 | 83.02 | 86.79 | 83.02 | 75.47 | 66.04 | 66.04 | 64.15 | 9.43 | 26.42 | 26.42 | 28.30 | 77.36 | 20.75 | 26.42 | 0.00 | 0.00 | 11.32 |
| 2fvj | 0.00 | 0.00 | 0.00 | 0.00 | 0.00 | 0.00 | 0.00 | 0.00 | 0.00 | 0.00 | 0.00 | 0.00 | 0.00 | 0.00 | 0.00 | 0.00 | 0.00 | 0.00 | 0.00 | 0.00 |
| 2ho2 | 47.06 | 41.18 | 47.06 | 58.82 | 41.18 | 35.29 | 47.06 | 47.06 | 29.41 | 29.41 | 29.41 | 47.06 | 41.18 | 17.65 | 35.29 | 11.76 | 35.29 | 29.41 | 41.18 | 35.29 |
| 2ht9 | 0.00 | 0.00 | 0.00 | 0.00 | 0.00 | 0.00 | 0.00 | 0.00 | 0.00 | 0.00 | 0.00 | 0.00 | 0.00 | 0.00 | 0.00 | 0.00 | 0.00 | 0.00 | 0.00 | 0.00 |
| 2o02 | 0.00 | 0.00 | 0.00 | 0.00 | 7.04 | 0.00 | 1.41 | 0.00 | 0.00 | 0.00 | 0.00 | 0.00 | 0.00 | 0.00 | 0.00 | 0.00 | 4.23 | 0.00 | 0.00 | 0.00 |
| 2o4j | 0.00 | 0.00 | 0.00 | 0.00 | 0.00 | 0.00 | 0.00 | 0.00 | 0.00 | 0.00 | 0.00 | 0.00 | 0.00 | 0.00 | 0.00 | 0.00 | 0.00 | 0.00 | 0.00 | 0.00 |
| 2o9v | 56.00 | 24.00 | 56.00 | 56.00 | 20.00 | 56.00 | 24.00 | 48.00 | 56.00 | 24.00 | 100.00 | 52.00 | 12.00 | 20.00 | 52.00 | 20.00 | 40.00 | 24.00 | 24.00 | 88.00 |
| 2oei | 31.82 | 22.73 | 54.55 | 27.27 | 27.27 | 27.27 | 31.82 | 72.73 | 50.00 | 27.27 | 63.64 | 50.00 | 31.82 | 22.73 | 22.73 | 22.73 | 0.00 | 68.18 | 50.00 | 86.36 |
| 2p0w | 0.00 | 0.00 | 0.00 | 1.56 | 0.00 | 0.00 | 0.00 | 0.00 | 0.00 | 1.56 | 0.00 | 7.81 | 0.00 | 3.13 | 0.00 | 0.00 | 1.56 | 0.00 | 0.00 | 0.00 |
| 2p1o | 20.00 | 17.78 | 95.56 | 31.11 | 88.89 | 24.44 | 93.33 | 20.00 | 20.00 | 24.44 | 22.22 | 15.56 | 57.78 | 11.11 | 15.56 | 20.00 | 31.11 | 15.56 | 17.78 | 20.00 |
| 2p1t | 100.00 | 37.14 | 85.71 | 0.00 | 0.00 | 37.14 | 0.00 | 40.00 | 25.71 | 0.00 | 0.00 | 0.00 | 85.71 | 0.00 | 37.14 | 0.00 | 0.00 | 0.00 | 20.00 | 0.00 |
| 2p54 | 0.00 | 0.00 | 0.00 | 0.00 | 0.00 | 0.00 | 0.00 | 0.00 | 2.63 | 0.00 | 0.00 | 0.00 | 0.00 | 0.00 | 0.00 | 0.00 | 0.00 | 0.00 | 0.00 | 5.26 |
| 2peh | 100.00 | 91.18 | 91.18 | 79.41 | 79.41 | 82.35 | 79.41 | 94.12 | 94.12 | 64.71 | 76.47 | 79.41 | 38.24 | 58.82 | 64.71 | 91.18 | 73.53 | 76.47 | 79.41 | 41.18 |
| 2pux | 0.00 | 0.00 | 0.00 | 0.00 | 0.00 | 0.00 | 0.00 | 0.00 | 0.00 | 0.00 | 0.00 | 0.00 | 0.00 | 97.30 | 0.00 | 0.00 | 0.00 | 0.00 | 0.00 | 0.00 |
| 2puy | 97.30 | 16.22 | 0.00 | 10.81 | 78.38 | 0.00 | 5.41 | 10.81 | 21.62 | 0.00 | 8.11 | 2.70 | 0.00 | 2.70 | 94.59 | 75.68 | 8.11 | 27.03 | 2.70 | 89.19 |
| 2qbx | 0.00 | 0.00 | 0.00 | 0.00 | 0.00 | 0.00 | 0.00 | 0.00 | 0.00 | 0.00 | 0.00 | 0.00 | 0.00 | 0.00 | 0.00 | 0.00 | 0.00 | 0.00 | 0.00 | 0.00 |
| 2qos | 11.32 | 73.58 | 9.43 | 13.21 | 81.13 | 43.40 | 79.25 | 7.55 | 81.13 | 56.60 | 13.21 | 39.62 | 5.66 | 7.55 | 18.87 | 3.77 | 7.55 | 7.55 | 13.21 | 13.21 |
| 2qse | 0.00 | 0.00 | 0.00 | 0.00 | 0.00 | 0.00 | 0.00 | 0.00 | 0.00 | 0.00 | 0.00 | 100.00 | 0.00 | 0.00 | 0.00 | 0.00 | 0.00 | 0.00 | 0.00 | 0.00 |
| 2r7g | 0.00 | 0.00 | 0.00 | 0.00 | 0.00 | 0.00 | 0.00 | 0.00 | 0.00 | 91.49 | 82.98 | 91.49 | 0.00 | 0.00 | 0.00 | 0.00 | 0.00 | 91.49 | 0.00 | 93.62 |
| 2r9q | 25.00 | 31.25 | 21.88 | 31.25 | 31.25 | 25.00 | 28.13 | 25.00 | 31.25 | 31.25 | 43.75 | 15.63 | 40.63 | 62.50 | 12.50 | 37.50 | 25.00 | 28.13 | 96.88 | 28.13 |
| 2v8y | 90.24 | 0.00 | 0.00 | 0.00 | 0.00 | 0.00 | 0.00 | 0.00 | 0.00 | 9.76 | 0.00 | 0.00 | 97.56 | 0.00 | 0.00 | 0.00 | 7.32 | 4.88 | 0.00 | 0.00 |
| 2vkn | 95.45 | 100.00 | 90.91 | 31.82 | 31.82 | 100.00 | 31.82 | 31.82 | 90.91 | 40.91 | 27.27 | 31.82 | 95.45 | 31.82 | 36.36 | 81.82 | 31.82 | 90.91 | 27.27 | 81.82 |
| 2vr3 | 95.24 | 1.19 | 0.00 | 0.00 | 90.48 | 0.00 | 0.00 | 0.00 | 0.00 | 1.19 | 0.00 | 0.00 | 0.00 | 0.00 | 1.19 | 5.95 | 0.00 | 0.00 | 0.00 | 2.38 |
| 2vwf | 100.00 | 88.57 | 65.71 | 8.57 | 85.71 | 11.43 | 22.86 | 8.57 | 0.00 | 11.43 | 11.43 | 14.29 | 22.86 | 2.86 | 22.86 | 20.00 | 85.71 | 8.57 | 57.14 | 8.57 |
| 2w2u | 0.00 | 14.89 | 0.00 | 0.00 | 0.00 | 2.13 | 0.00 | 2.13 | 2.13 | 0.00 | 0.00 | 2.13 | 2.13 | 0.00 | 14.89 | 0.00 | 8.51 | 4.26 | 0.00 | 0.00 |
| 2whx | 91.36 | 60.49 | 43.21 | 72.84 | 19.75 | 39.51 | 51.85 | 39.51 | 11.11 | 28.40 | 9.88 | 1.23 | 14.81 | 16.05 | 18.52 | 79.01 | 7.41 | 12.35 | 6.17 | 9.88 |
| 2xrw | 97.50 | 92.50 | 97.50 | 65.00 | 0.00 | 0.00 | 7.50 | 95.00 | 0.00 | 0.00 | 0.00 | 0.00 | 7.50 | 0.00 | 0.00 | 0.00 | 0.00 | 7.50 | 0.00 | 0.00 |
| 2xu7 | 93.88 | 81.63 | 53.06 | 0.00 | 73.47 | 59.18 | 55.10 | 40.82 | 40.82 | 51.02 | 14.29 | 4.08 | 0.00 | 48.98 | 0.00 | 12.24 | 14.29 | 46.94 | 34.69 | 38.78 |
| 2xvc | 76.47 | 76.47 | 21.57 | 3.92 | 1.96 | 0.00 | 0.00 | 3.92 | 47.06 | 0.00 | 0.00 | 0.00 | 1.96 | 0.00 | 0.00 | 1.96 | 0.00 | 1.96 | 0.00 | 3.92 |
| 2zjd | 97.44 | 0.00 | 0.00 | 0.00 | 0.00 | 0.00 | 0.00 | 0.00 | 0.00 | 0.00 | 0.00 | 0.00 | 0.00 | 0.00 | 0.00 | 0.00 | 82.05 | 2.56 | 0.00 | 0.00 |
| 3asl | 32.26 | 19.35 | 19.35 | 16.13 | 3.23 | 16.13 | 16.13 | 0.00 | 0.00 | 19.35 | 0.00 | 6.45 | 19.35 | 0.00 | 0.00 | 0.00 | 3.23 | 0.00 | 29.03 | 22.58 |
| 3awr | 6.45 | 3.23 | 6.45 | 9.68 | 0.00 | 0.00 | 6.45 | 0.00 | 0.00 | 12.90 | 3.23 | 0.00 | 6.45 | 0.00 | 6.45 | 6.45 | 6.45 | 9.68 | 0.00 | 0.00 |
| 3ayu | 95.00 | 88.33 | 90.00 | 78.33 | 61.67 | 68.33 | 11.67 | 15.00 | 85.00 | 30.00 | 23.33 | 75.00 | 26.67 | 81.67 | 35.00 | 25.00 | 28.33 | 83.33 | 48.33 | 11.67 |
| 3bfq | 100.00 | 100.00 | 10.42 | 4.17 | 4.17 | 4.17 | 21.88 | 95.83 | 5.21 | 6.25 | 38.54 | 3.13 | 9.38 | 55.21 | 8.33 | 0.00 | 2.08 | 4.17 | 46.88 | 9.38 |
| 3c3r | 0.00 | 0.00 | 0.00 | 100.00 | 0.00 | 0.00 | 0.00 | 0.00 | 0.00 | 0.00 | 0.00 | 0.00 | 0.00 | 0.00 | 0.00 | 0.00 | 0.00 | 0.00 | 0.00 | 0.00 |
| 3d32 | 88.10 | 88.10 | 11.90 | 14.29 | 2.38 | 0.00 | 0.00 | 97.62 | 11.90 | 21.43 | 19.05 | 4.76 | 14.29 | 7.14 | 19.05 | 0.00 | 2.38 | 16.67 | 0.00 | 9.52 |
| 3ds4 | 100.00 | 92.31 | 0.00 | 97.44 | 84.62 | 0.00 | 92.31 | 84.62 | 12.82 | 10.26 | 76.92 | 0.00 | 0.00 | 66.67 | 0.00 | 0.00 | 0.00 | 0.00 | 92.31 | 0.00 |
| 3ery | 81.25 | 73.44 | 92.19 | 92.19 | 73.44 | 70.31 | 68.75 | 46.88 | 75.00 | 64.06 | 43.75 | 50.00 | 68.75 | 45.31 | 46.88 | 43.75 | 6.25 | 51.56 | 82.81 | 35.94 |
| 3fdo | 100.00 | 93.33 | 20.00 | 13.33 | 10.00 | 20.00 | 13.33 | 96.67 | 13.33 | 86.67 | 23.33 | 10.00 | 60.00 | 16.67 | 10.00 | 10.00 | 23.33 | 70.00 | 63.33 | 56.67 |
| 3g2s | 97.37 | 86.84 | 84.21 | 13.16 | 15.79 | 0.00 | 94.74 | 89.47 | 15.79 | 89.47 | 89.47 | 47.37 | 52.63 | 31.58 | 94.74 | 84.21 | 71.05 | 5.26 | 15.79 | 0.00 |
| 3gyt | 100.00 | 100.00 | 96.55 | 96.55 | 13.79 | 0.00 | 0.00 | 0.00 | 96.55 | 55.17 | 0.00 | 0.00 | 0.00 | 0.00 | 0.00 | 0.00 | 0.00 | 0.00 | 96.55 | 0.00 |
| 3h1z | 90.57 | 13.21 | 11.32 | 9.43 | 45.28 | 9.43 | 7.55 | 0.00 | 18.87 | 58.49 | 13.21 | 47.17 | 26.42 | 22.64 | 18.87 | 7.55 | 0.00 | 0.00 | 0.00 | 7.55 |
| 3i5r | 100.00 | 92.31 | 23.08 | 23.08 | 100.00 | 23.08 | 19.23 | 19.23 | 100.00 | 38.46 | 23.08 | 15.38 | 96.15 | 26.92 | 26.92 | 23.08 | 23.08 | 26.92 | 23.08 | 26.92 |
| 3ivv | 8.82 | 17.65 | 94.12 | 14.71 | 8.82 | 8.82 | 17.65 | 8.82 | 20.59 | 17.65 | 11.76 | 23.53 | 14.71 | 26.47 | 17.65 | 94.12 | 20.59 | 11.76 | 11.76 | 8.82 |
| 3kmr | 100.00 | 0.00 | 0.00 | 0.00 | 96.67 | 100.00 | 0.00 | 0.00 | 0.00 | 0.00 | 100.00 | 0.00 | 0.00 | 0.00 | 0.00 | 0.00 | 0.00 | 93.33 | 0.00 | 0.00 |
| 3kuj | 13.64 | 0.00 | 2.27 | 15.91 | 20.45 | 4.55 | 2.27 | 11.36 | 2.27 | 2.27 | 0.00 | 0.00 | 0.00 | 11.36 | 15.91 | 0.00 | 0.00 | 4.55 | 4.55 | 0.00 |
| 3kus | 95.56 | 11.11 | 0.00 | 0.00 | 0.00 | 66.67 | 80.00 | 0.00 | 20.00 | 0.00 | 22.22 | 60.00 | 0.00 | 15.56 | 20.00 | 15.56 | 2.22 | 31.11 | 0.00 | 17.78 |
| 3l0e | 0.00 | 0.00 | 85.37 | 87.80 | 0.00 | 92.68 | 0.00 | 0.00 | 0.00 | 0.00 | 2.44 | 0.00 | 0.00 | 0.00 | 0.00 | 0.00 | 0.00 | 0.00 | 0.00 | 0.00 |
| 3ll8 | 95.35 | 83.72 | 76.74 | 0.00 | 0.00 | 0.00 | 0.00 | 0.00 | 0.00 | 0.00 | 0.00 | 0.00 | 0.00 | 0.00 | 0.00 | 0.00 | 0.00 | 0.00 | 0.00 | 16.28 |
| 3llz | 0.00 | 5.13 | 7.69 | 7.69 | 7.69 | 5.13 | 0.00 | 0.00 | 0.00 | 2.56 | 12.82 | 2.56 | 5.13 | 7.69 | 0.00 | 5.13 | 0.00 | 0.00 | 7.69 | 0.00 |
| 3obq | 11.11 | 25.00 | 22.22 | 22.22 | 94.44 | 38.89 | 33.33 | 22.22 | 13.89 | 19.44 | 22.22 | 25.00 | 30.56 | 27.78 | 22.22 | 33.33 | 25.00 | 19.44 | 30.56 | 75.00 |
| 3olf | 0.00 | 3.13 | 3.13 | 3.13 | 0.00 | 0.00 | 3.13 | 6.25 | 3.13 | 3.13 | 0.00 | 3.13 | 96.88 | 0.00 | 0.00 | 0.00 | 0.00 | 0.00 | 3.13 | 0.00 |
| 3p72 | 19.51 | 58.54 | 39.02 | 0.00 | 95.12 | 14.63 | 0.00 | 0.00 | 0.00 | 60.98 | 14.63 | 0.00 | 0.00 | 53.66 | 0.00 | 0.00 | 29.27 | 51.22 | 0.00 | 9.76 |
| 3p8f | 35.29 | 16.18 | 14.71 | 19.12 | 8.82 | 14.71 | 17.65 | 13.24 | 83.82 | 13.24 | 16.18 | 5.88 | 7.35 | 92.65 | 95.59 | 22.06 | 83.82 | 7.35 | 10.29 | 13.24 |
| 3ptl | 1.47 | 1.47 | 17.65 | 13.24 | 0.00 | 0.00 | 14.71 | 0.00 | 8.82 | 0.00 | 10.29 | 5.88 | 8.82 | 13.24 | 0.00 | 11.76 | 0.00 | 0.00 | 0.00 | 13.24 |
| 3qis | 100.00 | 85.29 | 82.35 | 14.71 | 94.12 | 14.71 | 32.35 | 11.76 | 35.29 | 5.88 | 14.71 | 14.71 | 11.76 | 11.76 | 76.47 | 94.12 | 14.71 | 32.35 | 23.53 | 11.76 |
| 3rm1 | 91.67 | 13.89 | 91.67 | 94.44 | 22.22 | 13.89 | 27.78 | 22.22 | 19.44 | 16.67 | 16.67 | 83.33 | 25.00 | 13.89 | 19.44 | 8.33 | 83.33 | 11.11 | 25.00 | 22.22 |
| 3rqg | 0.00 | 0.00 | 0.00 | 0.00 | 0.00 | 0.00 | 0.00 | 0.00 | 0.00 | 0.00 | 0.00 | 0.00 | 0.00 | 0.00 | 0.00 | 0.00 | 0.00 | 3.70 | 0.00 | 0.00 |
| 3sfj | 79.55 | 61.36 | 6.82 | 84.09 | 61.36 | 84.09 | 18.18 | 72.73 | 97.73 | 81.82 | 15.91 | 6.82 | 6.82 | 0.00 | 0.00 | 0.00 | 0.00 | 0.00 | 0.00 | 0.00 |
| 3so6 | 96.61 | 89.83 | 77.97 | 77.97 | 84.75 | 52.54 | 79.66 | 89.83 | 3.39 | 71.19 | 3.39 | 15.25 | 49.15 | 3.39 | 57.63 | 57.63 | 44.07 | 62.71 | 10.17 | 8.47 |
| 3tjv | 98.44 | 90.63 | 76.56 | 81.25 | 82.81 | 7.81 | 18.75 | 12.50 | 28.13 | 15.63 | 15.63 | 4.69 | 60.94 | 78.13 | 14.06 | 17.19 | 67.19 | 18.75 | 12.50 | 7.81 |
| 3tzy | 0.00 | 6.25 | 0.00 | 0.00 | 0.00 | 0.00 | 0.00 | 0.00 | 0.00 | 0.00 | 0.00 | 0.00 | 0.00 | 0.00 | 0.00 | 0.00 | 3.13 | 0.00 | 0.00 | 0.00 |
| 3u9q | 0.00 | 0.00 | 0.00 | 0.00 | 0.00 | 0.00 | 0.00 | 0.00 | 0.00 | 0.00 | 0.00 | 0.00 | 0.00 | 0.00 | 0.00 | 0.00 | 0.00 | 0.00 | 0.00 | 0.00 |
| 3up3 | 97.22 | 100.00 | 80.56 | 100.00 | 30.56 | 97.22 | 27.78 | 30.56 | 88.89 | 77.78 | 63.89 | 13.89 | 61.11 | 22.22 | 22.22 | 13.89 | 33.33 | 36.11 | 97.22 | 30.56 |
| 3v2x | 95.56 | 97.78 | 91.11 | 0.00 | 22.22 | 2.22 | 0.00 | 4.44 | 60.00 | 4.44 | 6.67 | 0.00 | 2.22 | 6.67 | 2.22 | 0.00 | 2.22 | 86.67 | 2.22 | 15.56 |
| 3vtc | 0.00 | 0.00 | 0.00 | 0.00 | 0.00 | 3.03 | 0.00 | 0.00 | 0.00 | 3.03 | 0.00 | 0.00 | 0.00 | 3.03 | 0.00 | 0.00 | 0.00 | 0.00 | 0.00 | 0.00 |
| 3w1b | 0.00 | 0.00 | 0.00 | 0.00 | 0.00 | 0.00 | 0.00 | 0.00 | 0.00 | 0.00 | 0.00 | 0.00 | 0.00 | 0.00 | 0.00 | 0.00 | 0.00 | 0.00 | 92.59 | 92.59 |
| 3zqh | 0.00 | 0.00 | 0.00 | 0.00 | 3.23 | 0.00 | 0.00 | 6.45 | 6.45 | 0.00 | 6.45 | 0.00 | 0.00 | 0.00 | 0.00 | 12.90 | 9.68 | 6.45 | 0.00 | 0.00 |
| 4b4n | 0.00 | 0.00 | 0.00 | 0.00 | 0.00 | 0.00 | 0.00 | 0.00 | 0.00 | 95.12 | 0.00 | 0.00 | 0.00 | 0.00 | 2.44 | 0.00 | 2.44 | 0.00 | 0.00 | 0.00 |
| 4dcb | 0.00 | 0.00 | 0.00 | 0.00 | 0.00 | 0.00 | 0.00 | 0.00 | 0.00 | 0.00 | 0.00 | 0.00 | 0.00 | 0.00 | 0.00 | 0.00 | 0.00 | 0.00 | 0.00 | 0.00 |
| 4e34 | 5.41 | 0.00 | 0.00 | 5.41 | 0.00 | 16.22 | 2.70 | 5.41 | 0.00 | 5.41 | 0.00 | 0.00 | 0.00 | 5.41 | 2.70 | 10.81 | 8.11 | 0.00 | 0.00 | 0.00 |
| 4eik | 97.44 | 97.44 | 23.08 | 17.95 | 23.08 | 20.51 | 25.64 | 20.51 | 15.38 | 87.18 | 20.51 | 23.08 | 23.08 | 17.95 | 17.95 | 38.46 | 15.38 | 64.10 | 15.38 | 66.67 |
| 4ery | 4.17 | 75.00 | 6.25 | 89.58 | 97.92 | 2.08 | 4.17 | 12.50 | 25.00 | 0.00 | 29.17 | 2.08 | 4.17 | 91.67 | 12.50 | 6.25 | 2.08 | 2.08 | 10.42 | 0.00 |
| 4f14 | 13.79 | 13.79 | 10.34 | 20.69 | 13.79 | 10.34 | 17.24 | 20.69 | 0.00 | 17.24 | 20.69 | 3.45 | 20.69 | 3.45 | 96.55 | 24.14 | 24.14 | 20.69 | 17.24 | 10.34 |
| 4f1z | 80.46 | 85.06 | 13.79 | 95.40 | 83.91 | 0.00 | 36.78 | 0.00 | 8.05 | 6.90 | 0.00 | 6.90 | 79.31 | 6.90 | 0.00 | 34.48 | 9.20 | 0.00 | 6.90 | 6.90 |
| 4gq6 | 5.36 | 5.36 | 42.86 | 5.36 | 5.36 | 19.64 | 3.57 | 3.57 | 73.21 | 66.07 | 3.57 | 5.36 | 17.86 | 44.64 | 5.36 | 50.00 | 30.36 | 26.79 | 17.86 | 57.14 |
| 4gxl | 0.00 | 0.00 | 0.00 | 0.00 | 0.00 | 0.00 | 0.00 | 0.00 | 7.89 | 5.26 | 0.00 | 0.00 | 0.00 | 0.00 | 0.00 | 0.00 | 0.00 | 0.00 | 0.00 | 0.00 |
| 4gyw | 5.77 | 0.00 | 0.00 | 5.77 | 7.69 | 0.00 | 11.54 | 36.54 | 0.00 | 0.00 | 0.00 | 3.85 | 0.00 | 0.00 | 0.00 | 0.00 | 0.00 | 0.00 | 0.00 | 3.85 |
| 4h4f | 0.00 | 8.51 | 14.89 | 23.40 | 19.15 | 23.40 | 17.02 | 0.00 | 14.89 | 19.15 | 19.15 | 8.51 | 0.00 | 21.28 | 8.51 | 2.13 | 19.15 | 0.00 | 17.02 | 10.64 |
| 4hom | 31.33 | 22.89 | 4.82 | 0.00 | 0.00 | 4.82 | 0.00 | 18.07 | 25.30 | 18.07 | 0.00 | 0.00 | 0.00 | 4.82 | 0.00 | 25.30 | 0.00 | 0.00 | 0.00 | 0.00 |
| 4htp | 0.00 | 0.00 | 0.00 | 0.00 | 0.00 | 100.00 | 0.00 | 0.00 | 0.00 | 96.67 | 0.00 | 0.00 | 0.00 | 0.00 | 0.00 | 0.00 | 0.00 | 0.00 | 0.00 | 93.33 |
| 4iim | 0.00 | 10.34 | 10.34 | 10.34 | 20.69 | 0.00 | 24.14 | 17.24 | 0.00 | 0.00 | 0.00 | 0.00 | 17.24 | 10.34 | 0.00 | 89.66 | 17.24 | 10.34 | 0.00 | 3.45 |
| 4j8s | 90.00 | 90.00 | 100.00 | 92.50 | 90.00 | 15.00 | 85.00 | 5.00 | 15.00 | 0.00 | 15.00 | 0.00 | 15.00 | 0.00 | 5.00 | 90.00 | 45.00 | 10.00 | 0.00 | 5.00 |
| 4k0u | 93.33 | 80.00 | 93.33 | 80.00 | 93.33 | 95.56 | 64.44 | 0.00 | 0.00 | 31.11 | 42.22 | 4.44 | 31.11 | 35.56 | 20.00 | 17.78 | 2.22 | 2.22 | 0.00 | 17.78 |

**S3(d) FNAT values of all 20 poses obtained after blind docking by PatchDock on 133 protein-peptide complexes.**

| **ID** | **Pose1** | **Pose2** | **Pose3** | **Pose4** | **Pose5** | **Pose6** | **Pose7** | **Pose8** | **Pose9** | **Pose10** | **Pose11** | **Pose12** | **Pose13** | **Pose14** | **Pose15** | **Pose16** | **Pose17** | **Pose18** | **Pose19** | **Pose20** |
| --- | --- | --- | --- | --- | --- | --- | --- | --- | --- | --- | --- | --- | --- | --- | --- | --- | --- | --- | --- | --- |
| 1cjr | 83.67 | 4.08 | 32.65 | 30.61 | 24.49 | 28.57 | 6.12 | 26.53 | 0.00 | 4.08 | 12.24 | 30.61 | 8.16 | 22.45 | 12.24 | 16.33 | 2.04 | 14.29 | 12.24 | 12.24 |
| 1cka | 28.57 | 42.86 | 0.00 | 3.57 | 10.71 | 21.43 | 46.43 | 10.71 | 3.57 | 0.00 | 3.57 | 14.29 | 0.00 | 17.86 | 14.29 | 7.14 | 0.00 | 10.71 | 3.57 | 32.14 |
| 1cvu | 0.00 | 0.00 | 0.00 | 0.00 | 0.00 | 0.00 | 0.00 | 0.00 | 0.00 | 0.00 | 0.00 | 0.00 | 0.00 | 0.00 | 0.00 | 0.00 | 0.00 | 0.00 | 0.00 | 0.00 |
| 1d4t | 92.86 | 66.07 | 60.71 | 32.14 | 12.50 | 14.29 | 7.14 | 39.29 | 12.50 | 41.07 | 0.00 | 19.64 | 0.00 | 0.00 | 50.00 | 42.86 | 39.29 | 21.43 | 5.36 | 71.43 |
| 1eg4 | 0.00 | 0.00 | 0.00 | 0.00 | 0.00 | 0.00 | 0.00 | 0.00 | 0.00 | 0.00 | 0.00 | 0.00 | 0.00 | 0.00 | 0.00 | 0.00 | 6.06 | 0.00 | 0.00 | 0.00 |
| 1h6w | 100.00 | 0.00 | 26.76 | 26.76 | 25.35 | 30.99 | 21.13 | 81.69 | 12.68 | 74.65 | 26.76 | 81.69 | 63.38 | 26.76 | 45.07 | 21.13 | 23.94 | 23.94 | 33.80 | 21.13 |
| 1hc9 | 95.56 | 75.56 | 2.22 | 37.78 | 0.00 | 15.56 | 15.56 | 6.67 | 60.00 | 26.67 | 17.78 | 28.89 | 24.44 | 55.56 | 24.44 | 6.67 | 20.00 | 15.56 | 22.22 | 4.44 |
| 1jbu | 0.00 | 9.84 | 16.39 | 0.00 | 0.00 | 0.00 | 0.00 | 0.00 | 24.59 | 0.00 | 13.11 | 4.92 | 3.28 | 0.00 | 0.00 | 0.00 | 0.00 | 0.00 | 0.00 | 6.56 |
| 1k5n | 3.70 | 24.07 | 0.00 | 0.00 | 1.85 | 40.74 | 5.56 | 0.00 | 9.26 | 0.00 | 0.00 | 7.41 | 27.78 | 27.78 | 0.00 | 0.00 | 33.33 | 3.70 | 25.93 | 0.00 |
| 1mfg | 14.71 | 17.65 | 55.88 | 5.88 | 58.82 | 50.00 | 0.00 | 14.71 | 61.76 | 32.35 | 20.59 | 23.53 | 0.00 | 32.35 | 0.00 | 20.59 | 2.94 | 14.71 | 5.88 | 17.65 |
| 1nln | 9.23 | 12.31 | 0.00 | 12.31 | 0.00 | 0.00 | 3.08 | 0.00 | 0.00 | 0.00 | 0.00 | 0.00 | 0.00 | 0.00 | 0.00 | 0.00 | 0.00 | 0.00 | 6.15 | 0.00 |
| 1nq7 | 0.00 | 9.09 | 0.00 | 6.06 | 0.00 | 0.00 | 0.00 | 0.00 | 15.15 | 0.00 | 3.03 | 0.00 | 0.00 | 0.00 | 0.00 | 0.00 | 0.00 | 6.06 | 0.00 | 3.03 |
| 1ntv | 0.00 | 93.48 | 0.00 | 4.35 | 0.00 | 17.39 | 0.00 | 0.00 | 0.00 | 0.00 | 2.17 | 0.00 | 0.00 | 0.00 | 0.00 | 0.00 | 6.52 | 4.35 | 0.00 | 0.00 |
| 1nx1 | 0.00 | 0.00 | 0.00 | 0.00 | 0.00 | 0.00 | 0.00 | 0.00 | 0.00 | 0.00 | 0.00 | 0.00 | 0.00 | 0.00 | 17.50 | 0.00 | 0.00 | 0.00 | 0.00 | 0.00 |
| 1oai | 22.58 | 0.00 | 0.00 | 16.13 | 0.00 | 9.68 | 3.23 | 6.45 | 0.00 | 25.81 | 0.00 | 0.00 | 0.00 | 48.39 | 9.68 | 0.00 | 12.90 | 0.00 | 3.23 | 22.58 |
| 1oj5 | 56.00 | 0.00 | 12.00 | 24.00 | 0.00 | 0.00 | 16.00 | 8.00 | 4.00 | 4.00 | 0.00 | 8.00 | 4.00 | 56.00 | 20.00 | 8.00 | 4.00 | 0.00 | 0.00 | 28.00 |
| 1ou8 | 24.44 | 24.44 | 33.33 | 0.00 | 95.56 | 28.89 | 0.00 | 0.00 | 24.44 | 0.00 | 0.00 | 0.00 | 0.00 | 0.00 | 0.00 | 0.00 | 0.00 | 0.00 | 31.11 | 0.00 |
| 1ow6 | 0.00 | 0.00 | 0.00 | 0.00 | 0.00 | 0.00 | 0.00 | 16.67 | 8.33 | 0.00 | 0.00 | 0.00 | 0.00 | 0.00 | 0.00 | 8.33 | 25.00 | 0.00 | 0.00 | 8.33 |
| 1pzl | 13.33 | 0.00 | 0.00 | 13.33 | 0.00 | 6.67 | 0.00 | 0.00 | 0.00 | 0.00 | 0.00 | 0.00 | 30.00 | 10.00 | 0.00 | 0.00 | 0.00 | 10.00 | 3.33 | 0.00 |
| 1qkz | 0.00 | 0.00 | 0.00 | 0.00 | 0.00 | 0.00 | 0.00 | 0.00 | 0.00 | 0.00 | 0.00 | 0.00 | 0.00 | 0.00 | 0.00 | 0.00 | 0.00 | 0.00 | 0.00 | 0.00 |
| 1rst | 31.03 | 37.93 | 24.14 | 24.14 | 48.28 | 13.79 | 10.34 | 79.31 | 0.00 | 34.48 | 34.48 | 34.48 | 20.69 | 37.93 | 55.17 | 27.59 | 58.62 | 0.00 | 41.38 | 41.38 |
| 1rxz | 7.41 | 1.85 | 0.00 | 9.26 | 0.00 | 42.59 | 11.11 | 0.00 | 0.00 | 0.00 | 3.70 | 1.85 | 1.85 | 0.00 | 1.85 | 0.00 | 37.04 | 0.00 | 96.30 | 0.00 |
| 1sfi | 87.72 | 12.28 | 10.53 | 36.84 | 35.09 | 0.00 | 17.54 | 0.00 | 21.05 | 14.04 | 15.79 | 40.35 | 8.77 | 17.54 | 24.56 | 0.00 | 31.58 | 50.88 | 26.32 | 21.05 |
| 1ssh | 13.33 | 53.33 | 0.00 | 50.00 | 20.00 | 0.00 | 0.00 | 13.33 | 0.00 | 30.00 | 26.67 | 53.33 | 20.00 | 16.67 | 26.67 | 0.00 | 23.33 | 20.00 | 30.00 | 23.33 |
| 1t08 | 0.00 | 8.96 | 8.96 | 0.00 | 7.46 | 0.00 | 8.96 | 0.00 | 1.49 | 0.00 | 1.49 | 2.99 | 0.00 | 11.94 | 0.00 | 11.94 | 11.94 | 10.45 | 13.43 | 0.00 |
| 1t4f | 25.00 | 21.43 | 71.43 | 21.43 | 17.86 | 17.86 | 42.86 | 0.00 | 32.14 | 28.57 | 32.14 | 21.43 | 21.43 | 17.86 | 25.00 | 7.14 | 17.86 | 0.00 | 32.14 | 32.14 |
| 1t7r | 0.00 | 0.00 | 0.00 | 0.00 | 0.00 | 0.00 | 19.35 | 0.00 | 3.23 | 0.00 | 9.68 | 0.00 | 6.45 | 96.77 | 0.00 | 0.00 | 0.00 | 0.00 | 3.23 | 0.00 |
| 1tfc | 3.23 | 0.00 | 0.00 | 16.13 | 0.00 | 0.00 | 0.00 | 0.00 | 0.00 | 3.23 | 0.00 | 0.00 | 0.00 | 0.00 | 0.00 | 3.23 | 19.35 | 0.00 | 0.00 | 0.00 |
| 1u00 | 0.00 | 0.00 | 37.50 | 12.50 | 35.42 | 8.33 | 0.00 | 10.42 | 0.00 | 0.00 | 0.00 | 20.83 | 0.00 | 12.50 | 0.00 | 18.75 | 37.50 | 60.42 | 0.00 | 0.00 |
| 1uj0 | 14.81 | 14.81 | 18.52 | 29.63 | 44.44 | 33.33 | 0.00 | 11.11 | 14.81 | 29.63 | 11.11 | 3.70 | 22.22 | 11.11 | 0.00 | 22.22 | 22.22 | 29.63 | 40.74 | 18.52 |
| 1x2r | 86.05 | 34.88 | 0.00 | 6.98 | 27.91 | 0.00 | 30.23 | 11.63 | 0.00 | 25.58 | 0.00 | 25.58 | 0.00 | 25.58 | 0.00 | 0.00 | 32.56 | 34.88 | 30.23 | 0.00 |
| 1xoc | 92.94 | 24.71 | 0.00 | 45.88 | 0.00 | 0.00 | 0.00 | 0.00 | 0.00 | 0.00 | 0.00 | 16.47 | 62.35 | 0.00 | 4.71 | 0.00 | 1.18 | 0.00 | 1.18 | 1.18 |
| 1ymt | 0.00 | 0.00 | 32.14 | 21.43 | 0.00 | 0.00 | 0.00 | 3.57 | 0.00 | 0.00 | 17.86 | 0.00 | 0.00 | 0.00 | 42.86 | 0.00 | 0.00 | 0.00 | 0.00 | 0.00 |
| 1yuc | 5.56 | 11.11 | 13.89 | 0.00 | 0.00 | 19.44 | 0.00 | 0.00 | 2.78 | 0.00 | 11.11 | 22.22 | 0.00 | 0.00 | 0.00 | 5.56 | 5.56 | 16.67 | 16.67 | 0.00 |
| 1ywo | 8.00 | 0.00 | 72.00 | 12.00 | 28.00 | 60.00 | 12.00 | 32.00 | 0.00 | 0.00 | 4.00 | 32.00 | 8.00 | 0.00 | 32.00 | 16.00 | 0.00 | 16.00 | 28.00 | 16.00 |
| 2a25 | 100.00 | 27.50 | 25.00 | 2.50 | 7.50 | 27.50 | 15.00 | 10.00 | 32.50 | 20.00 | 5.00 | 12.50 | 0.00 | 22.50 | 0.00 | 20.00 | 2.50 | 0.00 | 17.50 | 0.00 |
| 2a3i | 0.00 | 28.13 | 0.00 | 15.63 | 12.50 | 0.00 | 0.00 | 31.25 | 0.00 | 0.00 | 0.00 | 28.13 | 21.88 | 12.50 | 21.88 | 0.00 | 25.00 | 0.00 | 0.00 | 0.00 |
| 2aq9 | 15.00 | 0.00 | 0.00 | 0.00 | 0.00 | 0.00 | 0.00 | 0.00 | 0.00 | 0.00 | 0.00 | 10.00 | 0.00 | 5.00 | 0.00 | 0.00 | 0.00 | 0.00 | 0.00 | 5.00 |
| 2b9h | 0.00 | 0.00 | 4.08 | 22.45 | 0.00 | 2.04 | 0.00 | 0.00 | 0.00 | 0.00 | 2.04 | 0.00 | 0.00 | 0.00 | 0.00 | 0.00 | 22.45 | 0.00 | 0.00 | 0.00 |
| 2bba | 12.28 | 96.49 | 15.79 | 8.77 | 15.79 | 0.00 | 0.00 | 22.81 | 15.79 | 21.05 | 0.00 | 49.12 | 12.28 | 8.77 | 5.26 | 45.61 | 21.05 | 7.02 | 0.00 | 8.77 |
| 2cch | 0.00 | 0.00 | 9.30 | 0.00 | 0.00 | 0.00 | 0.00 | 0.00 | 0.00 | 2.33 | 0.00 | 0.00 | 0.00 | 0.00 | 0.00 | 0.00 | 0.00 | 0.00 | 0.00 | 0.00 |
| 2ce8 | 0.00 | 0.00 | 0.00 | 0.00 | 3.23 | 0.00 | 0.00 | 0.00 | 0.00 | 0.00 | 0.00 | 0.00 | 0.00 | 0.00 | 0.00 | 0.00 | 0.00 | 0.00 | 0.00 | 0.00 |
| 2d0n | 8.00 | 28.00 | 0.00 | 20.00 | 32.00 | 28.00 | 24.00 | 16.00 | 8.00 | 32.00 | 4.00 | 12.00 | 20.00 | 8.00 | 0.00 | 40.00 | 28.00 | 8.00 | 4.00 | 24.00 |
| 2drk | 0.00 | 13.33 | 0.00 | 33.33 | 36.67 | 26.67 | 16.67 | 0.00 | 0.00 | 16.67 | 26.67 | 3.33 | 3.33 | 10.00 | 13.33 | 46.67 | 3.33 | 26.67 | 10.00 | 3.33 |
| 2dyp | 4.62 | 10.77 | 1.54 | 9.23 | 21.54 | 13.85 | 0.00 | 66.15 | 26.15 | 6.15 | 0.00 | 0.00 | 18.46 | 16.92 | 47.69 | 0.00 | 0.00 | 0.00 | 0.00 | 0.00 |
| 2fff | 0.00 | 0.00 | 0.00 | 0.00 | 0.00 | 0.00 | 0.00 | 0.00 | 0.00 | 0.00 | 0.00 | 0.00 | 0.00 | 0.00 | 0.00 | 0.00 | 0.00 | 0.00 | 0.00 | 0.00 |
| 2ffu | 17.95 | 0.00 | 53.85 | 10.26 | 28.21 | 25.64 | 15.38 | 30.77 | 0.00 | 0.00 | 0.00 | 12.82 | 0.00 | 0.00 | 0.00 | 15.38 | 0.00 | 15.38 | 0.00 | 0.00 |
| 2fka | 0.00 | 52.38 | 33.33 | 52.38 | 0.00 | 28.57 | 19.05 | 0.00 | 52.38 | 19.05 | 0.00 | 4.76 | 23.81 | 23.81 | 23.81 | 0.00 | 0.00 | 23.81 | 23.81 | 38.10 |
| 2fmf | 22.73 | 54.55 | 31.82 | 0.00 | 0.00 | 0.00 | 9.09 | 22.73 | 0.00 | 18.18 | 0.00 | 0.00 | 13.64 | 0.00 | 0.00 | 0.00 | 9.09 | 27.27 | 36.36 | 0.00 |
| 2fts | 3.77 | 100.00 | 3.77 | 0.00 | 0.00 | 0.00 | 13.21 | 0.00 | 0.00 | 0.00 | 0.00 | 0.00 | 0.00 | 0.00 | 0.00 | 0.00 | 11.32 | 0.00 | 18.87 | 1.89 |
| 2fvj | 0.00 | 0.00 | 0.00 | 0.00 | 6.25 | 0.00 | 0.00 | 28.13 | 0.00 | 0.00 | 0.00 | 46.88 | 0.00 | 21.88 | 0.00 | 0.00 | 12.50 | 0.00 | 0.00 | 0.00 |
| 2ho2 | 0.00 | 0.00 | 0.00 | 0.00 | 0.00 | 0.00 | 0.00 | 0.00 | 0.00 | 11.76 | 17.65 | 0.00 | 0.00 | 0.00 | 5.88 | 23.53 | 5.88 | 17.65 | 5.88 | 35.29 |
| 2ht9 | 0.00 | 0.00 | 0.00 | 7.69 | 0.00 | 0.00 | 0.00 | 15.38 | 0.00 | 0.00 | 0.00 | 0.00 | 7.69 | 7.69 | 0.00 | 0.00 | 38.46 | 0.00 | 0.00 | 15.38 |
| 2o02 | 95.77 | 12.68 | 38.03 | 19.72 | 74.65 | 8.45 | 59.15 | 36.62 | 14.08 | 47.89 | 46.48 | 16.90 | 4.23 | 11.27 | 0.00 | 12.68 | 12.68 | 0.00 | 11.27 | 43.66 |
| 2o4j | 29.41 | 0.00 | 8.82 | 5.88 | 8.82 | 0.00 | 23.53 | 14.71 | 23.53 | 0.00 | 0.00 | 0.00 | 23.53 | 20.59 | 0.00 | 2.94 | 0.00 | 0.00 | 0.00 | 0.00 |
| 2o9v | 0.00 | 4.00 | 0.00 | 0.00 | 8.00 | 0.00 | 16.00 | 0.00 | 0.00 | 0.00 | 12.00 | 0.00 | 4.00 | 0.00 | 8.00 | 0.00 | 12.00 | 0.00 | 0.00 | 0.00 |
| 2oei | 0.00 | 0.00 | 0.00 | 0.00 | 0.00 | 18.18 | 0.00 | 0.00 | 0.00 | 13.64 | 0.00 | 0.00 | 0.00 | 0.00 | 0.00 | 0.00 | 31.82 | 13.64 | 13.64 | 0.00 |
| 2p0w | 17.19 | 18.75 | 0.00 | 1.56 | 3.13 | 12.50 | 10.94 | 35.94 | 40.63 | 0.00 | 7.81 | 3.13 | 12.50 | 25.00 | 1.56 | 0.00 | 0.00 | 6.25 | 0.00 | 3.13 |
| 2p1o | 0.00 | 8.89 | 64.44 | 2.22 | 0.00 | 0.00 | 2.22 | 17.78 | 13.33 | 0.00 | 17.78 | 6.67 | 75.56 | 15.56 | 0.00 | 20.00 | 26.67 | 2.22 | 13.33 | 2.22 |
| 2p1t | 17.14 | 8.57 | 5.71 | 8.57 | 20.00 | 20.00 | 2.86 | 11.43 | 0.00 | 5.71 | 0.00 | 14.29 | 20.00 | 25.71 | 14.29 | 0.00 | 2.86 | 8.57 | 0.00 | 0.00 |
| 2p54 | 0.00 | 0.00 | 97.37 | 2.63 | 0.00 | 0.00 | 0.00 | 0.00 | 0.00 | 0.00 | 0.00 | 0.00 | 0.00 | 0.00 | 2.63 | 0.00 | 0.00 | 0.00 | 13.16 | 0.00 |
| 2peh | 0.00 | 0.00 | 2.94 | 2.94 | 0.00 | 0.00 | 0.00 | 0.00 | 0.00 | 0.00 | 0.00 | 5.88 | 0.00 | 2.94 | 23.53 | 2.94 | 0.00 | 0.00 | 0.00 | 0.00 |
| 2pux | 0.00 | 0.00 | 2.70 | 16.22 | 0.00 | 0.00 | 5.41 | 0.00 | 8.11 | 5.41 | 18.92 | 0.00 | 0.00 | 5.41 | 16.22 | 2.70 | 0.00 | 0.00 | 0.00 | 8.11 |
| 2puy | 13.51 | 8.11 | 8.11 | 43.24 | 0.00 | 21.62 | 24.32 | 29.73 | 0.00 | 27.03 | 29.73 | 2.70 | 13.51 | 8.11 | 13.51 | 29.73 | 18.92 | 21.62 | 29.73 | 0.00 |
| 2qbx | 91.53 | 35.59 | 0.00 | 18.64 | 33.90 | 5.08 | 0.00 | 0.00 | 33.90 | 27.12 | 5.08 | 91.53 | 8.47 | 11.86 | 0.00 | 30.51 | 5.08 | 22.03 | 0.00 | 0.00 |
| 2qos | 13.21 | 32.08 | 28.30 | 16.98 | 96.23 | 13.21 | 39.62 | 13.21 | 35.85 | 18.87 | 11.32 | 50.94 | 20.75 | 52.83 | 37.74 | 30.19 | 18.87 | 18.87 | 13.21 | 16.98 |
| 2qse | 36.67 | 20.00 | 0.00 | 16.67 | 0.00 | 0.00 | 13.33 | 0.00 | 3.33 | 0.00 | 0.00 | 20.00 | 0.00 | 0.00 | 0.00 | 0.00 | 0.00 | 3.33 | 0.00 | 0.00 |
| 2r7g | 27.66 | 25.53 | 36.17 | 21.28 | 17.02 | 14.89 | 76.60 | 21.28 | 12.77 | 17.02 | 14.89 | 19.15 | 57.45 | 14.89 | 42.55 | 27.66 | 0.00 | 23.40 | 0.00 | 12.77 |
| 2r9q | 28.13 | 3.13 | 6.25 | 0.00 | 0.00 | 9.38 | 0.00 | 93.75 | 12.50 | 34.38 | 0.00 | 12.50 | 3.13 | 3.13 | 28.13 | 0.00 | 31.25 | 0.00 | 0.00 | 28.13 |
| 2v8y | 0.00 | 0.00 | 100.00 | 0.00 | 0.00 | 0.00 | 0.00 | 9.76 | 0.00 | 0.00 | 0.00 | 0.00 | 0.00 | 0.00 | 19.51 | 0.00 | 9.76 | 0.00 | 0.00 | 0.00 |
| 2vkn | 0.00 | 9.09 | 4.55 | 13.64 | 9.09 | 0.00 | 0.00 | 45.45 | 22.73 | 0.00 | 0.00 | 0.00 | 0.00 | 0.00 | 0.00 | 18.18 | 0.00 | 0.00 | 0.00 | 13.64 |
| 2vr3 | 100.00 | 0.00 | 9.52 | 5.95 | 19.05 | 13.10 | 17.86 | 0.00 | 0.00 | 0.00 | 22.62 | 9.52 | 29.76 | 0.00 | 0.00 | 1.19 | 36.90 | 0.00 | 0.00 | 25.00 |
| 2vwf | 17.14 | 5.71 | 2.86 | 8.57 | 14.29 | 14.29 | 22.86 | 22.86 | 11.43 | 2.86 | 2.86 | 2.86 | 11.43 | 0.00 | 20.00 | 31.43 | 2.86 | 31.43 | 0.00 | 0.00 |
| 2w2u | 19.15 | 6.38 | 12.77 | 8.51 | 36.17 | 2.13 | 29.79 | 27.66 | 19.15 | 0.00 | 0.00 | 25.53 | 6.38 | 27.66 | 0.00 | 17.02 | 0.00 | 23.40 | 12.77 | 29.79 |
| 2whx | 1.23 | 0.00 | 0.00 | 0.00 | 0.00 | 0.00 | 0.00 | 0.00 | 0.00 | 0.00 | 0.00 | 0.00 | 0.00 | 0.00 | 1.23 | 0.00 | 0.00 | 0.00 | 0.00 | 0.00 |
| 2xrw | 0.00 | 0.00 | 0.00 | 0.00 | 0.00 | 0.00 | 30.00 | 5.00 | 0.00 | 0.00 | 0.00 | 0.00 | 0.00 | 0.00 | 0.00 | 0.00 | 0.00 | 0.00 | 0.00 | 0.00 |
| 2xu7 | 0.00 | 0.00 | 8.16 | 4.08 | 0.00 | 0.00 | 14.29 | 0.00 | 0.00 | 2.04 | 0.00 | 0.00 | 0.00 | 12.24 | 16.33 | 44.90 | 0.00 | 0.00 | 8.16 | 46.94 |
| 2xvc | 21.57 | 25.49 | 25.49 | 15.69 | 25.49 | 27.45 | 23.53 | 98.04 | 56.86 | 23.53 | 70.59 | 13.73 | 21.57 | 19.61 | 21.57 | 27.45 | 52.94 | 23.53 | 29.41 | 21.57 |
| 2zjd | 2.56 | 0.00 | 0.00 | 7.69 | 5.13 | 0.00 | 0.00 | 0.00 | 0.00 | 0.00 | 0.00 | 0.00 | 0.00 | 0.00 | 92.31 | 2.56 | 0.00 | 2.56 | 0.00 | 10.26 |
| 3asl | 12.90 | 0.00 | 0.00 | 0.00 | 16.13 | 25.81 | 0.00 | 0.00 | 0.00 | 38.71 | 0.00 | 3.23 | 22.58 | 3.23 | 19.35 | 12.90 | 22.58 | 0.00 | 32.26 | 3.23 |
| 3awr | 9.68 | 0.00 | 16.13 | 3.23 | 12.90 | 19.35 | 12.90 | 12.90 | 6.45 | 0.00 | 3.23 | 16.13 | 19.35 | 22.58 | 19.35 | 12.90 | 0.00 | 9.68 | 19.35 | 0.00 |
| 3ayu | 10.00 | 96.67 | 33.33 | 26.67 | 0.00 | 20.00 | 16.67 | 0.00 | 35.00 | 13.33 | 1.67 | 73.33 | 0.00 | 0.00 | 0.00 | 45.00 | 0.00 | 10.00 | 0.00 | 0.00 |
| 3bfq | 96.88 | 11.46 | 11.46 | 11.46 | 19.79 | 14.58 | 67.71 | 0.00 | 13.54 | 13.54 | 11.46 | 9.38 | 44.79 | 17.71 | 13.54 | 9.38 | 12.50 | 13.54 | 9.38 | 14.58 |
| 3c3r | 0.00 | 0.00 | 0.00 | 0.00 | 0.00 | 0.00 | 0.00 | 0.00 | 0.00 | 0.00 | 24.14 | 0.00 | 0.00 | 0.00 | 0.00 | 0.00 | 0.00 | 3.45 | 0.00 | 0.00 |
| 3d32 | 100.00 | 16.67 | 26.19 | 33.33 | 0.00 | 0.00 | 16.67 | 7.14 | 4.76 | 0.00 | 0.00 | 2.38 | 4.76 | 2.38 | 9.52 | 2.38 | 0.00 | 4.76 | 0.00 | 4.76 |
| 3ds4 | 100.00 | 17.95 | 15.38 | 12.82 | 12.82 | 48.72 | 20.51 | 17.95 | 20.51 | 33.33 | 20.51 | 7.69 | 25.64 | 30.77 | 12.82 | 30.77 | 15.38 | 10.26 | 7.69 | 12.82 |
| 3ery | 65.63 | 12.50 | 37.50 | 42.19 | 0.00 | 46.88 | 34.38 | 25.00 | 12.50 | 14.06 | 14.06 | 10.94 | 15.63 | 3.13 | 0.00 | 0.00 | 0.00 | 20.31 | 1.56 | 6.25 |
| 3fdo | 6.67 | 23.33 | 10.00 | 20.00 | 43.33 | 16.67 | 83.33 | 10.00 | 3.33 | 3.33 | 26.67 | 26.67 | 56.67 | 0.00 | 93.33 | 16.67 | 26.67 | 23.33 | 16.67 | 30.00 |
| 3g2s | 0.00 | 0.00 | 0.00 | 0.00 | 44.74 | 47.37 | 2.63 | 13.16 | 0.00 | 0.00 | 0.00 | 0.00 | 13.16 | 0.00 | 0.00 | 5.26 | 13.16 | 0.00 | 0.00 | 0.00 |
| 3gyt | 0.00 | 0.00 | 0.00 | 0.00 | 0.00 | 20.69 | 6.90 | 0.00 | 0.00 | 0.00 | 0.00 | 0.00 | 3.45 | 0.00 | 31.03 | 0.00 | 0.00 | 6.90 | 3.45 | 0.00 |
| 3h1z | 0.00 | 0.00 | 0.00 | 0.00 | 0.00 | 0.00 | 0.00 | 43.40 | 0.00 | 0.00 | 0.00 | 0.00 | 1.89 | 3.77 | 0.00 | 0.00 | 5.66 | 0.00 | 0.00 | 0.00 |
| 3i5r | 23.08 | 7.69 | 0.00 | 23.08 | 38.46 | 46.15 | 15.38 | 0.00 | 38.46 | 19.23 | 11.54 | 11.54 | 7.69 | 0.00 | 7.69 | 38.46 | 0.00 | 26.92 | 53.85 | 0.00 |
| 3ivv | 0.00 | 0.00 | 58.82 | 0.00 | 0.00 | 0.00 | 0.00 | 0.00 | 0.00 | 20.59 | 0.00 | 0.00 | 0.00 | 0.00 | 0.00 | 8.82 | 0.00 | 0.00 | 0.00 | 0.00 |
| 3kmr | 16.67 | 0.00 | 3.33 | 0.00 | 3.33 | 6.67 | 0.00 | 0.00 | 0.00 | 6.67 | 0.00 | 0.00 | 10.00 | 0.00 | 13.33 | 0.00 | 10.00 | 0.00 | 6.67 | 3.33 |
| 3kuj | 0.00 | 15.91 | 40.91 | 0.00 | 0.00 | 13.64 | 4.55 | 0.00 | 0.00 | 15.91 | 0.00 | 0.00 | 2.27 | 4.55 | 0.00 | 4.55 | 0.00 | 13.64 | 0.00 | 0.00 |
| 3kus | 0.00 | 0.00 | 13.33 | 0.00 | 20.00 | 17.78 | 42.22 | 13.33 | 35.56 | 17.78 | 4.44 | 20.00 | 17.78 | 0.00 | 4.44 | 20.00 | 8.89 | 11.11 | 35.56 | 55.56 |
| 3l0e | 0.00 | 17.07 | 0.00 | 2.44 | 21.95 | 0.00 | 0.00 | 19.51 | 2.44 | 0.00 | 14.63 | 0.00 | 0.00 | 0.00 | 0.00 | 0.00 | 0.00 | 14.63 | 0.00 | 17.07 |
| 3ll8 | 0.00 | 0.00 | 0.00 | 0.00 | 0.00 | 0.00 | 2.33 | 100.00 | 0.00 | 0.00 | 0.00 | 0.00 | 0.00 | 0.00 | 0.00 | 2.33 | 0.00 | 0.00 | 0.00 | 0.00 |
| 3llz | 0.00 | 0.00 | 10.26 | 2.56 | 0.00 | 0.00 | 0.00 | 0.00 | 2.56 | 17.95 | 0.00 | 0.00 | 0.00 | 5.13 | 12.82 | 0.00 | 0.00 | 0.00 | 5.13 | 0.00 |
| 3obq | 52.78 | 0.00 | 0.00 | 5.56 | 0.00 | 0.00 | 75.00 | 0.00 | 0.00 | 0.00 | 0.00 | 2.78 | 0.00 | 0.00 | 2.78 | 0.00 | 0.00 | 0.00 | 0.00 | 0.00 |
| 3olf | 0.00 | 0.00 | 0.00 | 0.00 | 0.00 | 9.38 | 0.00 | 0.00 | 0.00 | 25.00 | 0.00 | 0.00 | 0.00 | 0.00 | 0.00 | 0.00 | 0.00 | 0.00 | 0.00 | 0.00 |
| 3p72 | 60.98 | 29.27 | 17.07 | 9.76 | 17.07 | 14.63 | 0.00 | 19.51 | 7.32 | 17.07 | 19.51 | 12.20 | 17.07 | 7.32 | 17.07 | 21.95 | 17.07 | 85.37 | 7.32 | 17.07 |
| 3p8f | 94.12 | 13.24 | 4.41 | 76.47 | 10.29 | 19.12 | 10.29 | 5.88 | 14.71 | 42.65 | 7.35 | 13.24 | 23.53 | 17.65 | 7.35 | 11.76 | 45.59 | 8.82 | 16.18 | 5.88 |
| 3ptl | 8.82 | 23.53 | 13.24 | 0.00 | 77.94 | 0.00 | 2.94 | 4.41 | 0.00 | 0.00 | 0.00 | 17.65 | 30.88 | 0.00 | 0.00 | 0.00 | 0.00 | 14.71 | 0.00 | 0.00 |
| 3qis | 0.00 | 0.00 | 0.00 | 0.00 | 0.00 | 0.00 | 0.00 | 0.00 | 0.00 | 0.00 | 0.00 | 0.00 | 0.00 | 23.53 | 0.00 | 5.88 | 11.76 | 0.00 | 8.82 | 0.00 |
| 3rm1 | 11.11 | 5.56 | 22.22 | 75.00 | 11.11 | 16.67 | 13.89 | 0.00 | 5.56 | 0.00 | 47.22 | 25.00 | 50.00 | 22.22 | 0.00 | 8.33 | 30.56 | 5.56 | 75.00 | 33.33 |
| 3rqg | 0.00 | 0.00 | 0.00 | 0.00 | 33.33 | 14.81 | 22.22 | 55.56 | 0.00 | 0.00 | 0.00 | 0.00 | 18.52 | 7.41 | 100.00 | 0.00 | 37.04 | 14.81 | 0.00 | 0.00 |
| 3sfj | 18.18 | 0.00 | 22.73 | 11.36 | 0.00 | 0.00 | 11.36 | 93.18 | 20.45 | 15.91 | 0.00 | 11.36 | 0.00 | 11.36 | 36.36 | 45.45 | 0.00 | 11.36 | 2.27 | 0.00 |
| 3so6 | 96.61 | 15.25 | 20.34 | 11.86 | 57.63 | 0.00 | 0.00 | 15.25 | 1.69 | 1.69 | 3.39 | 66.10 | 0.00 | 1.69 | 0.00 | 0.00 | 0.00 | 0.00 | 10.17 | 0.00 |
| 3tjv | 76.56 | 25.00 | 25.00 | 17.19 | 17.19 | 12.50 | 0.00 | 56.25 | 0.00 | 0.00 | 0.00 | 7.81 | 31.25 | 18.75 | 76.56 | 0.00 | 12.50 | 0.00 | 0.00 | 0.00 |
| 3tzy | 3.13 | 3.13 | 0.00 | 3.13 | 0.00 | 3.13 | 0.00 | 15.63 | 6.25 | 9.38 | 0.00 | 9.38 | 28.13 | 3.13 | 3.13 | 12.50 | 6.25 | 15.63 | 0.00 | 0.00 |
| 3u9q | 0.00 | 0.00 | 21.21 | 0.00 | 78.79 | 0.00 | 12.12 | 0.00 | 0.00 | 0.00 | 36.36 | 0.00 | 0.00 | 0.00 | 0.00 | 0.00 | 0.00 | 3.03 | 0.00 | 0.00 |
| 3up3 | 0.00 | 22.22 | 0.00 | 19.44 | 11.11 | 13.89 | 5.56 | 16.67 | 0.00 | 0.00 | 0.00 | 91.67 | 0.00 | 0.00 | 0.00 | 8.33 | 16.67 | 38.89 | 0.00 | 2.78 |
| 3v2x | 0.00 | 4.44 | 22.22 | 6.67 | 0.00 | 26.67 | 0.00 | 0.00 | 0.00 | 0.00 | 0.00 | 0.00 | 26.67 | 15.56 | 0.00 | 0.00 | 55.56 | 2.22 | 0.00 | 0.00 |
| 3vtc | 12.12 | 0.00 | 15.15 | 6.06 | 18.18 | 12.12 | 24.24 | 0.00 | 0.00 | 24.24 | 3.03 | 27.27 | 6.06 | 0.00 | 6.06 | 96.97 | 0.00 | 0.00 | 0.00 | 3.03 |
| 3w1b | 0.00 | 0.00 | 0.00 | 0.00 | 0.00 | 0.00 | 0.00 | 0.00 | 0.00 | 0.00 | 0.00 | 0.00 | 0.00 | 0.00 | 0.00 | 0.00 | 0.00 | 0.00 | 0.00 | 0.00 |
| 3zqh | 0.00 | 0.00 | 6.45 | 0.00 | 0.00 | 0.00 | 0.00 | 0.00 | 3.23 | 0.00 | 0.00 | 0.00 | 0.00 | 0.00 | 25.81 | 0.00 | 16.13 | 12.90 | 0.00 | 0.00 |
| 4b4n | 19.51 | 0.00 | 0.00 | 24.39 | 26.83 | 26.83 | 12.20 | 0.00 | 0.00 | 0.00 | 0.00 | 0.00 | 0.00 | 0.00 | 0.00 | 19.51 | 0.00 | 0.00 | 0.00 | 9.76 |
| 4dcb | 26.83 | 26.83 | 14.63 | 56.10 | 12.20 | 9.76 | 31.71 | 26.83 | 51.22 | 39.02 | 31.71 | 31.71 | 17.07 | 51.22 | 58.54 | 0.00 | 14.63 | 100.00 | 0.00 | 0.00 |
| 4e34 | 21.62 | 0.00 | 100.00 | 2.70 | 5.41 | 5.41 | 16.22 | 2.70 | 27.03 | 5.41 | 0.00 | 2.70 | 0.00 | 24.32 | 2.70 | 8.11 | 29.73 | 0.00 | 24.32 | 16.22 |
| 4eik | 20.51 | 5.13 | 2.56 | 0.00 | 0.00 | 0.00 | 0.00 | 0.00 | 5.13 | 0.00 | 2.56 | 92.31 | 0.00 | 5.13 | 7.69 | 25.64 | 43.59 | 30.77 | 20.51 | 10.26 |
| 4ery | 0.00 | 0.00 | 0.00 | 0.00 | 29.17 | 0.00 | 0.00 | 0.00 | 35.42 | 0.00 | 0.00 | 0.00 | 29.17 | 8.33 | 0.00 | 0.00 | 64.58 | 4.17 | 0.00 | 0.00 |
| 4f14 | 20.69 | 17.24 | 10.34 | 3.45 | 3.45 | 13.79 | 10.34 | 20.69 | 6.90 | 3.45 | 13.79 | 0.00 | 20.69 | 0.00 | 20.69 | 3.45 | 3.45 | 27.59 | 0.00 | 10.34 |
| 4f1z | 86.21 | 82.76 | 13.79 | 8.05 | 0.00 | 0.00 | 0.00 | 0.00 | 5.75 | 9.20 | 1.15 | 0.00 | 64.37 | 12.64 | 0.00 | 9.20 | 24.14 | 17.24 | 0.00 | 0.00 |
| 4gq6 | 62.50 | 44.64 | 48.21 | 23.21 | 12.50 | 5.36 | 28.57 | 25.00 | 21.43 | 16.07 | 3.57 | 28.57 | 23.21 | 32.14 | 30.36 | 16.07 | 23.21 | 16.07 | 16.07 | 17.86 |
| 4gxl | 0.00 | 0.00 | 0.00 | 0.00 | 0.00 | 0.00 | 0.00 | 0.00 | 0.00 | 0.00 | 0.00 | 0.00 | 0.00 | 0.00 | 0.00 | 0.00 | 0.00 | 0.00 | 0.00 | 0.00 |
| 4gyw | 0.00 | 0.00 | 0.00 | 0.00 | 0.00 | 0.00 | 19.23 | 0.00 | 0.00 | 0.00 | 9.62 | 0.00 | 0.00 | 7.69 | 0.00 | 48.08 | 9.62 | 0.00 | 0.00 | 0.00 |
| 4h4f | 0.00 | 0.00 | 0.00 | 0.00 | 0.00 | 0.00 | 0.00 | 0.00 | 0.00 | 0.00 | 0.00 | 0.00 | 0.00 | 0.00 | 0.00 | 0.00 | 0.00 | 0.00 | 0.00 | 0.00 |
| 4hom | 87.95 | 7.23 | 0.00 | 0.00 | 0.00 | 0.00 | 0.00 | 4.82 | 10.84 | 0.00 | 9.64 | 0.00 | 2.41 | 0.00 | 0.00 | 0.00 | 0.00 | 0.00 | 0.00 | 0.00 |
| 4htp | 3.33 | 0.00 | 0.00 | 0.00 | 6.67 | 0.00 | 30.00 | 0.00 | 0.00 | 0.00 | 0.00 | 3.33 | 0.00 | 0.00 | 0.00 | 0.00 | 0.00 | 0.00 | 0.00 | 0.00 |
| 4iim | 17.24 | 0.00 | 6.90 | 0.00 | 0.00 | 24.14 | 93.10 | 0.00 | 55.17 | 13.79 | 0.00 | 3.45 | 24.14 | 0.00 | 0.00 | 17.24 | 17.24 | 3.45 | 17.24 | 20.69 |
| 4j8s | 25.00 | 12.50 | 0.00 | 10.00 | 5.00 | 2.50 | 22.50 | 0.00 | 0.00 | 15.00 | 25.00 | 0.00 | 17.50 | 15.00 | 22.50 | 5.00 | 5.00 | 32.50 | 7.50 | 17.50 |
| 4k0u | 0.00 | 100.00 | 0.00 | 22.22 | 28.89 | 13.33 | 6.67 | 15.56 | 15.56 | 13.33 | 24.44 | 11.11 | 13.33 | 17.78 | 4.44 | 42.22 | 0.00 | 8.89 | 24.44 | 13.33 |

**S3(e). FNAT values of all 20 poses obtained after blind docking by pepATTRACT on 133 protein-peptide complexes.**

| **ID** | **Pose1** | **Pose2** | **Pose3** | **Pose4** | **Pose5** | **Pose6** | **Pose7** | **Pose8** | **Pose9** | **Pose10** | **Pose11** | **Pose12** | **Pose13** | **Pose14** | **Pose15** | **Pose16** | **Pose17** | **Pose18** | **Pose19** | **Pose20** |
| --- | --- | --- | --- | --- | --- | --- | --- | --- | --- | --- | --- | --- | --- | --- | --- | --- | --- | --- | --- | --- |
| 1cjr | 14.29 | 14.29 | 8.16 | 10.20 | 6.12 | 14.29 | 18.37 | 10.20 | 18.37 | 12.24 | 12.24 | 12.24 | 12.24 | 0.00 | 10.20 | 18.37 | 6.12 | 8.16 | 0.00 | 4.08 |
| 1cka | 82.14 | 92.86 | 96.43 | 17.86 | 89.29 | 96.43 | 46.43 | 100.00 | 50.00 | 50.00 | 14.29 | 64.29 | 46.43 | 42.86 | 35.71 | 60.71 | 17.86 | 14.29 | 42.86 | 42.86 |
| 1cvu | 0.00 | 0.00 | 0.00 | 0.00 | 0.00 | 0.00 | 0.00 | 0.00 | 0.00 | 0.00 | 0.00 | 0.00 | 0.00 | 0.00 | 0.00 | 0.00 | 0.00 | 0.00 | 0.00 | 0.00 |
| 1d4t | 0.00 | 0.00 | 7.14 | 0.00 | 0.00 | 0.00 | 0.00 | 0.00 | 0.00 | 0.00 | 0.00 | 0.00 | 0.00 | 0.00 | 0.00 | 0.00 | 0.00 | 0.00 | 0.00 | 0.00 |
| 1eg4 | 0.00 | 0.00 | 0.00 | 0.00 | 0.00 | 0.00 | 0.00 | 0.00 | 0.00 | 0.00 | 21.21 | 0.00 | 0.00 | 0.00 | 0.00 | 0.00 | 6.06 | 0.00 | 0.00 | 0.00 |
| 1h6w | 0.00 | 0.00 | 0.00 | 0.00 | 0.00 | 0.00 | 0.00 | 0.00 | 0.00 | 0.00 | 0.00 | 0.00 | 0.00 | 0.00 | 0.00 | 0.00 | 0.00 | 0.00 | 0.00 | 0.00 |
| 1hc9 | 2.22 | 2.22 | 15.56 | 2.22 | 0.00 | 26.67 | 2.22 | 2.22 | 6.67 | 4.44 | 2.22 | 11.11 | 4.44 | 15.56 | 4.44 | 8.89 | 4.44 | 2.22 | 8.89 | 6.67 |
| 1jbu | 1.64 | 0.00 | 1.64 | 1.64 | 1.64 | 21.31 | 34.43 | 18.03 | 11.48 | 19.67 | 0.00 | 29.51 | 1.64 | 13.11 | 3.28 | 9.84 | 6.56 | 11.48 | 0.00 | 32.79 |
| 1k5n | 0.00 | 0.00 | 0.00 | 0.00 | 0.00 | 0.00 | 0.00 | 9.26 | 0.00 | 0.00 | 0.00 | 0.00 | 0.00 | 0.00 | 0.00 | 0.00 | 0.00 | 0.00 | 0.00 | 0.00 |
| 1mfg | 0.00 | 2.94 | 8.82 | 2.94 | 23.53 | 20.59 | 2.94 | 17.65 | 0.00 | 20.59 | 0.00 | 23.53 | 17.65 | 20.59 | 17.65 | 0.00 | 23.53 | 8.82 | 0.00 | 0.00 |
| 1nln | 0.00 | 0.00 | 0.00 | 0.00 | 0.00 | 0.00 | 0.00 | 0.00 | 0.00 | 0.00 | 0.00 | 0.00 | 0.00 | 0.00 | 0.00 | 0.00 | 0.00 | 0.00 | 0.00 | 0.00 |
| 1nq7 | 0.00 | 0.00 | 0.00 | 0.00 | 0.00 | 0.00 | 0.00 | 0.00 | 0.00 | 0.00 | 0.00 | 0.00 | 0.00 | 0.00 | 0.00 | 0.00 | 0.00 | 0.00 | 0.00 | 0.00 |
| 1ntv | 21.74 | 8.70 | 23.91 | 4.35 | 0.00 | 2.17 | 8.70 | 6.52 | 13.04 | 0.00 | 6.52 | 17.39 | 19.57 | 17.39 | 13.04 | 0.00 | 0.00 | 13.04 | 0.00 | 17.39 |
| 1nx1 | 0.00 | 0.00 | 0.00 | 0.00 | 0.00 | 0.00 | 0.00 | 0.00 | 0.00 | 0.00 | 0.00 | 0.00 | 0.00 | 0.00 | 0.00 | 0.00 | 0.00 | 0.00 | 0.00 | 0.00 |
| 1oai | 38.71 | 38.71 | 38.71 | 32.26 | 41.94 | 41.94 | 38.71 | 38.71 | 32.26 | 41.94 | 32.26 | 41.94 | 41.94 | 35.48 | 35.48 | 35.48 | 19.35 | 22.58 | 35.48 | 45.16 |
| 1oj5 | 0.00 | 0.00 | 0.00 | 0.00 | 0.00 | 0.00 | 0.00 | 4.00 | 0.00 | 0.00 | 0.00 | 4.00 | 0.00 | 16.00 | 0.00 | 8.00 | 4.00 | 0.00 | 0.00 | 4.00 |
| 1ou8 | 13.33 | 17.78 | 15.56 | 13.33 | 4.44 | 15.56 | 6.67 | 11.11 | 2.22 | 2.22 | 15.56 | 55.56 | 8.89 | 11.11 | 13.33 | 15.56 | 13.33 | 0.00 | 0.00 | 22.22 |
| 1ow6 | 16.67 | 0.00 | 16.67 | 8.33 | 25.00 | 16.67 | 16.67 | 0.00 | 16.67 | 16.67 | 25.00 | 16.67 | 25.00 | 16.67 | 0.00 | 0.00 | 8.33 | 0.00 | 0.00 | 0.00 |
| 1pzl | 0.00 | 0.00 | 0.00 | 0.00 | 0.00 | 0.00 | 0.00 | 0.00 | 0.00 | 0.00 | 0.00 | 0.00 | 0.00 | 0.00 | 0.00 | 0.00 | 0.00 | 0.00 | 0.00 | 0.00 |
| 1qkz | 17.95 | 0.00 | 0.00 | 0.00 | 0.00 | 0.00 | 0.00 | 0.00 | 0.00 | 0.00 | 0.00 | 0.00 | 0.00 | 0.00 | 0.00 | 12.82 | 0.00 | 0.00 | 25.64 | 0.00 |
| 1rst | 24.14 | 31.03 | 31.03 | 27.59 | 31.03 | 24.14 | 31.03 | 24.14 | 27.59 | 31.03 | 24.14 | 6.90 | 17.24 | 20.69 | 34.48 | 55.17 | 37.93 | 24.14 | 20.69 | 24.14 |
| 1rxz | 11.11 | 12.96 | 12.96 | 12.96 | 11.11 | 11.11 | 12.96 | 9.26 | 0.00 | 12.96 | 12.96 | 11.11 | 18.52 | 12.96 | 11.11 | 16.67 | 0.00 | 9.26 | 0.00 | 9.26 |
| 1sfi | 5.26 | 8.77 | 10.53 | 10.53 | 5.26 | 7.02 | 10.53 | 7.02 | 12.28 | 7.02 | 5.26 | 7.02 | 7.02 | 3.51 | 10.53 | 15.79 | 5.26 | 10.53 | 7.02 | 7.02 |
| 1ssh | 80.00 | 90.00 | 76.67 | 90.00 | 86.67 | 83.33 | 83.33 | 66.67 | 80.00 | 86.67 | 63.33 | 83.33 | 86.67 | 76.67 | 80.00 | 63.33 | 86.67 | 76.67 | 86.67 | 80.00 |
| 1t08 | 5.97 | 1.49 | 0.00 | 0.00 | 1.49 | 0.00 | 1.49 | 4.48 | 1.49 | 2.99 | 5.97 | 0.00 | 7.46 | 5.97 | 2.99 | 2.99 | 1.49 | 4.48 | 0.00 | 2.99 |
| 1t4f | 25.00 | 35.71 | 0.00 | 28.57 | 10.71 | 0.00 | 35.71 | 7.14 | 21.43 | 3.57 | 46.43 | 35.71 | 46.43 | 42.86 | 39.29 | 0.00 | 21.43 | 3.57 | 3.57 | 3.57 |
| 1t7r | 0.00 | 0.00 | 19.35 | 19.35 | 0.00 | 22.58 | 29.03 | 0.00 | 22.58 | 16.13 | 25.81 | 25.81 | 0.00 | 0.00 | 19.35 | 25.81 | 22.58 | 19.35 | 0.00 | 0.00 |
| 1tfc | 12.90 | 25.81 | 25.81 | 41.94 | 12.90 | 38.71 | 22.58 | 25.81 | 41.94 | 22.58 | 25.81 | 38.71 | 22.58 | 19.35 | 29.03 | 29.03 | 32.26 | 29.03 | 32.26 | 29.03 |
| 1u00 | 22.92 | 16.67 | 12.50 | 2.08 | 25.00 | 22.92 | 20.83 | 10.42 | 20.83 | 4.17 | 10.42 | 8.33 | 6.25 | 14.58 | 27.08 | 14.58 | 6.25 | 10.42 | 6.25 | 14.58 |
| 1uj0 | 59.26 | 40.74 | 51.85 | 66.67 | 55.56 | 40.74 | 74.07 | 51.85 | 55.56 | 7.41 | 25.93 | 25.93 | 70.37 | 25.93 | 59.26 | 44.44 | 7.41 | 37.04 | 14.81 | 18.52 |
| 1x2r | 20.93 | 16.28 | 16.28 | 11.63 | 9.30 | 11.63 | 11.63 | 11.63 | 9.30 | 20.93 | 6.98 | 0.00 | 0.00 | 11.63 | 0.00 | 6.98 | 16.28 | 18.60 | 9.30 | 0.00 |
| 1xoc | 0.00 | 0.00 | 0.00 | 0.00 | 0.00 | 0.00 | 0.00 | 0.00 | 2.35 | 0.00 | 0.00 | 0.00 | 0.00 | 0.00 | 0.00 | 1.18 | 0.00 | 0.00 | 1.18 | 1.18 |
| 1ymt | 32.14 | 17.86 | 0.00 | 0.00 | 35.71 | 21.43 | 10.71 | 0.00 | 0.00 | 10.71 | 0.00 | 10.71 | 0.00 | 0.00 | 0.00 | 7.14 | 0.00 | 0.00 | 3.57 | 0.00 |
| 1yuc | 8.33 | 0.00 | 0.00 | 0.00 | 0.00 | 0.00 | 0.00 | 2.78 | 0.00 | 0.00 | 0.00 | 0.00 | 0.00 | 0.00 | 0.00 | 0.00 | 0.00 | 0.00 | 19.44 | 0.00 |
| 1ywo | 32.00 | 24.00 | 12.00 | 44.00 | 12.00 | 4.00 | 12.00 | 0.00 | 60.00 | 24.00 | 16.00 | 28.00 | 32.00 | 4.00 | 4.00 | 4.00 | 36.00 | 48.00 | 52.00 | 40.00 |
| 2a25 | 0.00 | 0.00 | 0.00 | 0.00 | 0.00 | 10.00 | 0.00 | 0.00 | 0.00 | 17.50 | 0.00 | 12.50 | 0.00 | 0.00 | 2.50 | 22.50 | 0.00 | 60.00 | 0.00 | 0.00 |
| 2a3i | 0.00 | 6.25 | 0.00 | 0.00 | 34.38 | 0.00 | 28.13 | 6.25 | 0.00 | 0.00 | 0.00 | 0.00 | 0.00 | 40.63 | 0.00 | 0.00 | 0.00 | 28.13 | 40.63 | 0.00 |
| 2aq9 | 0.00 | 0.00 | 0.00 | 0.00 | 0.00 | 0.00 | 0.00 | 0.00 | 0.00 | 0.00 | 0.00 | 0.00 | 0.00 | 0.00 | 0.00 | 0.00 | 0.00 | 0.00 | 0.00 | 0.00 |
| 2b9h | 36.73 | 34.69 | 6.12 | 0.00 | 0.00 | 0.00 | 8.16 | 8.16 | 4.08 | 0.00 | 12.24 | 6.12 | 44.90 | 8.16 | 6.12 | 16.33 | 0.00 | 0.00 | 0.00 | 0.00 |
| 2bba | 24.56 | 29.82 | 0.00 | 0.00 | 0.00 | 26.32 | 0.00 | 22.81 | 0.00 | 0.00 | 12.28 | 21.05 | 28.07 | 28.07 | 26.32 | 22.81 | 0.00 | 22.81 | 0.00 | 0.00 |
| 2cch | 0.00 | 0.00 | 0.00 | 0.00 | 0.00 | 0.00 | 0.00 | 0.00 | 41.86 | 0.00 | 0.00 | 0.00 | 0.00 | 0.00 | 0.00 | 0.00 | 0.00 | 0.00 | 0.00 | 0.00 |
| 2ce8 | 0.00 | 0.00 | 0.00 | 0.00 | 0.00 | 0.00 | 0.00 | 0.00 | 0.00 | 0.00 | 0.00 | 0.00 | 0.00 | 0.00 | 0.00 | 0.00 | 0.00 | 0.00 | 0.00 | 0.00 |
| 2d0n | 72.00 | 68.00 | 72.00 | 36.00 | 64.00 | 80.00 | 68.00 | 60.00 | 68.00 | 68.00 | 76.00 | 12.00 | 68.00 | 20.00 | 28.00 | 64.00 | 32.00 | 72.00 | 20.00 | 16.00 |
| 2drk | 20.00 | 23.33 | 16.67 | 23.33 | 16.67 | 13.33 | 20.00 | 30.00 | 16.67 | 20.00 | 26.67 | 20.00 | 20.00 | 16.67 | 70.00 | 16.67 | 33.33 | 20.00 | 16.67 | 20.00 |
| 2dyp | 0.00 | 3.08 | 0.00 | 1.54 | 3.08 | 0.00 | 0.00 | 3.08 | 3.08 | 1.54 | 3.08 | 0.00 | 3.08 | 3.08 | 3.08 | 0.00 | 3.08 | 3.08 | 0.00 | 3.08 |
| 2fff | 0.00 | 0.00 | 6.52 | 0.00 | 0.00 | 0.00 | 0.00 | 0.00 | 0.00 | 0.00 | 0.00 | 0.00 | 0.00 | 0.00 | 0.00 | 0.00 | 0.00 | 0.00 | 0.00 | 0.00 |
| 2ffu | 12.82 | 12.82 | 0.00 | 20.51 | 15.38 | 20.51 | 43.59 | 0.00 | 20.51 | 20.51 | 2.56 | 0.00 | 17.95 | 10.26 | 7.69 | 5.13 | 7.69 | 41.03 | 17.95 | 17.95 |
| 2fka | 38.10 | 38.10 | 38.10 | 42.86 | 52.38 | 57.14 | 47.62 | 42.86 | 47.62 | 33.33 | 42.86 | 38.10 | 47.62 | 42.86 | 42.86 | 52.38 | 38.10 | 47.62 | 33.33 | 42.86 |
| 2fmf | 31.82 | 45.45 | 36.36 | 13.64 | 13.64 | 13.64 | 13.64 | 22.73 | 18.18 | 13.64 | 18.18 | 9.09 | 13.64 | 9.09 | 18.18 | 18.18 | 13.64 | 18.18 | 13.64 | 18.18 |
| 2fts | 0.00 | 0.00 | 0.00 | 0.00 | 0.00 | 0.00 | 0.00 | 0.00 | 0.00 | 0.00 | 0.00 | 0.00 | 0.00 | 0.00 | 0.00 | 0.00 | 0.00 | 0.00 | 0.00 | 0.00 |
| 2fvj | 0.00 | 3.13 | 12.50 | 3.13 | 3.13 | 9.38 | 3.13 | 3.13 | 0.00 | 9.38 | 6.25 | 3.13 | 3.13 | 9.38 | 0.00 | 3.13 | 0.00 | 6.25 | 0.00 | 3.13 |
| 2ho2 | 41.18 | 17.65 | 35.29 | 29.41 | 35.29 | 64.71 | 35.29 | 52.94 | 29.41 | 29.41 | 11.76 | 29.41 | 35.29 | 64.71 | 17.65 | 35.29 | 29.41 | 23.53 | 35.29 | 5.88 |
| 2ht9 | 0.00 | 0.00 | 0.00 | 0.00 | 0.00 | 0.00 | 0.00 | 0.00 | 0.00 | 0.00 | 0.00 | 0.00 | 0.00 | 0.00 | 0.00 | 0.00 | 0.00 | 0.00 | 0.00 | 0.00 |
| 2o02 | 7.04 | 9.86 | 4.23 | 8.45 | 4.23 | 5.63 | 5.63 | 25.35 | 4.23 | 5.63 | 21.13 | 4.23 | 18.31 | 1.41 | 2.82 | 2.82 | 18.31 | 0.00 | 12.68 | 0.00 |
| 2o4j | 0.00 | 0.00 | 0.00 | 0.00 | 0.00 | 0.00 | 0.00 | 0.00 | 0.00 | 0.00 | 0.00 | 0.00 | 0.00 | 0.00 | 0.00 | 0.00 | 0.00 | 0.00 | 0.00 | 0.00 |
| 2o9v | 12.00 | 20.00 | 16.00 | 28.00 | 20.00 | 16.00 | 16.00 | 20.00 | 12.00 | 28.00 | 24.00 | 20.00 | 28.00 | 16.00 | 16.00 | 12.00 | 24.00 | 28.00 | 12.00 | 20.00 |
| 2oei | 59.09 | 22.73 | 22.73 | 31.82 | 27.27 | 22.73 | 50.00 | 22.73 | 54.55 | 36.36 | 22.73 | 18.18 | 18.18 | 36.36 | 18.18 | 18.18 | 27.27 | 13.64 | 31.82 | 22.73 |
| 2p0w | 10.94 | 12.50 | 7.81 | 9.38 | 10.94 | 12.50 | 10.94 | 4.69 | 4.69 | 7.81 | 4.69 | 15.63 | 12.50 | 0.00 | 0.00 | 6.25 | 0.00 | 0.00 | 14.06 | 0.00 |
| 2p1o | 0.00 | 0.00 | 0.00 | 0.00 | 0.00 | 0.00 | 0.00 | 0.00 | 2.22 | 2.22 | 0.00 | 0.00 | 17.78 | 0.00 | 15.56 | 0.00 | 22.22 | 11.11 | 0.00 | 20.00 |
| 2p1t | 0.00 | 0.00 | 0.00 | 0.00 | 0.00 | 0.00 | 0.00 | 0.00 | 0.00 | 0.00 | 5.71 | 0.00 | 0.00 | 0.00 | 0.00 | 0.00 | 0.00 | 0.00 | 0.00 | 0.00 |
| 2p54 | 0.00 | 0.00 | 0.00 | 0.00 | 0.00 | 0.00 | 0.00 | 0.00 | 0.00 | 0.00 | 0.00 | 0.00 | 0.00 | 0.00 | 0.00 | 0.00 | 0.00 | 0.00 | 0.00 | 7.89 |
| 2peh | 0.00 | 0.00 | 0.00 | 2.94 | 0.00 | 0.00 | 0.00 | 2.94 | 0.00 | 0.00 | 0.00 | 0.00 | 20.59 | 0.00 | 20.59 | 23.53 | 20.59 | 0.00 | 20.59 | 0.00 |
| 2pux | 10.81 | 10.81 | 13.51 | 16.22 | 13.51 | 8.11 | 13.51 | 16.22 | 10.81 | 10.81 | 10.81 | 8.11 | 8.11 | 16.22 | 13.51 | 10.81 | 13.51 | 8.11 | 16.22 | 0.00 |
| 2puy | 10.81 | 16.22 | 16.22 | 13.51 | 13.51 | 13.51 | 16.22 | 13.51 | 13.51 | 10.81 | 16.22 | 10.81 | 13.51 | 10.81 | 5.41 | 13.51 | 13.51 | 35.14 | 13.51 | 35.14 |
| 2qbx | 0.00 | 0.00 | 0.00 | 0.00 | 0.00 | 0.00 | 10.17 | 0.00 | 1.69 | 0.00 | 0.00 | 18.64 | 0.00 | 0.00 | 18.64 | 0.00 | 0.00 | 0.00 | 0.00 | 0.00 |
| 2qos | 0.00 | 1.89 | 1.89 | 1.89 | 1.89 | 1.89 | 11.32 | 5.66 | 0.00 | 9.43 | 13.21 | 16.98 | 1.89 | 11.32 | 0.00 | 1.89 | 7.55 | 11.32 | 13.21 | 13.21 |
| 2qse | 0.00 | 0.00 | 0.00 | 0.00 | 0.00 | 0.00 | 0.00 | 0.00 | 0.00 | 0.00 | 0.00 | 0.00 | 0.00 | 0.00 | 0.00 | 23.33 | 0.00 | 0.00 | 0.00 | 0.00 |
| 2r7g | 0.00 | 0.00 | 0.00 | 0.00 | 0.00 | 0.00 | 0.00 | 6.38 | 0.00 | 0.00 | 0.00 | 0.00 | 0.00 | 0.00 | 0.00 | 0.00 | 0.00 | 0.00 | 0.00 | 0.00 |
| 2r9q | 12.50 | 34.38 | 21.88 | 31.25 | 3.13 | 21.88 | 21.88 | 6.25 | 15.63 | 21.88 | 18.75 | 21.88 | 15.63 | 21.88 | 3.13 | 18.75 | 28.13 | 21.88 | 25.00 | 21.88 |
| 2v8y | 9.76 | 0.00 | 0.00 | 9.76 | 7.32 | 0.00 | 0.00 | 0.00 | 2.44 | 0.00 | 7.32 | 0.00 | 0.00 | 0.00 | 9.76 | 0.00 | 12.20 | 9.76 | 0.00 | 9.76 |
| 2vkn | 40.91 | 36.36 | 40.91 | 31.82 | 36.36 | 31.82 | 40.91 | 36.36 | 36.36 | 36.36 | 18.18 | 36.36 | 50.00 | 31.82 | 36.36 | 45.45 | 18.18 | 36.36 | 40.91 | 36.36 |
| 2vr3 | 0.00 | 5.95 | 3.57 | 5.95 | 3.57 | 1.19 | 0.00 | 0.00 | 2.38 | 1.19 | 0.00 | 11.90 | 0.00 | 0.00 | 0.00 | 2.38 | 23.81 | 15.48 | 22.62 | 0.00 |
| 2vwf | 22.86 | 14.29 | 0.00 | 37.14 | 20.00 | 14.29 | 20.00 | 28.57 | 14.29 | 17.14 | 5.71 | 28.57 | 2.86 | 31.43 | 28.57 | 20.00 | 37.14 | 11.43 | 37.14 | 20.00 |
| 2w2u | 34.04 | 48.94 | 51.06 | 44.68 | 48.94 | 48.94 | 40.43 | 27.66 | 40.43 | 0.00 | 0.00 | 0.00 | 48.94 | 51.06 | 0.00 | 0.00 | 23.40 | 44.68 | 21.28 | 53.19 |
| 2whx | 0.00 | 0.00 | 0.00 | 0.00 | 0.00 | 0.00 | 0.00 | 0.00 | 0.00 | 0.00 | 0.00 | 0.00 | 0.00 | 0.00 | 0.00 | 0.00 | 0.00 | 0.00 | 0.00 | 0.00 |
| 2xrw | 0.00 | 0.00 | 0.00 | 0.00 | 0.00 | 0.00 | 0.00 | 0.00 | 0.00 | 0.00 | 0.00 | 0.00 | 0.00 | 0.00 | 0.00 | 2.50 | 0.00 | 0.00 | 0.00 | 0.00 |
| 2xu7 | 42.86 | 44.90 | 36.73 | 0.00 | 0.00 | 8.16 | 42.86 | 0.00 | 0.00 | 40.82 | 4.08 | 0.00 | 4.08 | 0.00 | 0.00 | 34.69 | 0.00 | 0.00 | 10.20 | 0.00 |
| 2xvc | 9.80 | 9.80 | 9.80 | 17.65 | 9.80 | 7.84 | 13.73 | 11.76 | 3.92 | 9.80 | 7.84 | 9.80 | 0.00 | 7.84 | 0.00 | 21.57 | 0.00 | 7.84 | 11.76 | 7.84 |
| 2zjd | 41.03 | 56.41 | 48.72 | 56.41 | 58.97 | 56.41 | 53.85 | 15.38 | 53.85 | 58.97 | 58.97 | 51.28 | 0.00 | 20.51 | 10.26 | 12.82 | 23.08 | 25.64 | 10.26 | 0.00 |
| 3asl | 0.00 | 6.45 | 0.00 | 25.81 | 0.00 | 22.58 | 3.23 | 0.00 | 16.13 | 0.00 | 0.00 | 0.00 | 0.00 | 3.23 | 16.13 | 0.00 | 12.90 | 3.23 | 3.23 | 0.00 |
| 3awr | 0.00 | 0.00 | 0.00 | 0.00 | 3.23 | 6.45 | 0.00 | 19.35 | 0.00 | 0.00 | 0.00 | 32.26 | 29.03 | 38.71 | 29.03 | 29.03 | 3.23 | 0.00 | 35.48 | 6.45 |
| 3ayu | 11.67 | 11.67 | 33.33 | 30.00 | 25.00 | 20.00 | 28.33 | 0.00 | 16.67 | 21.67 | 23.33 | 21.67 | 23.33 | 23.33 | 15.00 | 15.00 | 23.33 | 28.33 | 33.33 | 36.67 |
| 3bfq | 0.00 | 0.00 | 0.00 | 0.00 | 0.00 | 28.13 | 4.17 | 0.00 | 32.29 | 5.21 | 37.50 | 6.25 | 38.54 | 0.00 | 0.00 | 0.00 | 0.00 | 3.13 | 11.46 | 10.42 |
| 3c3r | 0.00 | 0.00 | 0.00 | 0.00 | 0.00 | 0.00 | 0.00 | 0.00 | 0.00 | 20.69 | 0.00 | 0.00 | 0.00 | 0.00 | 0.00 | 0.00 | 0.00 | 0.00 | 0.00 | 0.00 |
| 3d32 | 0.00 | 0.00 | 14.29 | 0.00 | 0.00 | 0.00 | 2.38 | 0.00 | 0.00 | 9.52 | 23.81 | 7.14 | 16.67 | 0.00 | 11.90 | 0.00 | 9.52 | 9.52 | 0.00 | 4.76 |
| 3ds4 | 0.00 | 0.00 | 0.00 | 0.00 | 0.00 | 0.00 | 0.00 | 0.00 | 0.00 | 0.00 | 0.00 | 0.00 | 0.00 | 0.00 | 0.00 | 0.00 | 0.00 | 0.00 | 0.00 | 0.00 |
| 3ery | 35.94 | 57.81 | 53.13 | 59.38 | 54.69 | 62.50 | 60.94 | 54.69 | 18.75 | 64.06 | 25.00 | 64.06 | 60.94 | 57.81 | 65.63 | 48.44 | 7.81 | 9.38 | 42.19 | 62.50 |
| 3fdo | 0.00 | 0.00 | 0.00 | 0.00 | 0.00 | 36.67 | 30.00 | 0.00 | 23.33 | 33.33 | 36.67 | 36.67 | 36.67 | 43.33 | 36.67 | 33.33 | 30.00 | 43.33 | 33.33 | 33.33 |
| 3g2s | 5.26 | 2.63 | 15.79 | 5.26 | 21.05 | 18.42 | 26.32 | 31.58 | 23.68 | 13.16 | 13.16 | 0.00 | 0.00 | 34.21 | 0.00 | 18.42 | 26.32 | 10.53 | 0.00 | 15.79 |
| 3gyt | 0.00 | 0.00 | 0.00 | 0.00 | 0.00 | 0.00 | 0.00 | 0.00 | 0.00 | 0.00 | 0.00 | 20.69 | 0.00 | 0.00 | 0.00 | 0.00 | 0.00 | 0.00 | 0.00 | 0.00 |
| 3h1z | 5.66 | 1.89 | 0.00 | 0.00 | 0.00 | 0.00 | 0.00 | 0.00 | 0.00 | 0.00 | 0.00 | 0.00 | 0.00 | 0.00 | 3.77 | 0.00 | 0.00 | 0.00 | 0.00 | 0.00 |
| 3i5r | 3.85 | 11.54 | 3.85 | 23.08 | 23.08 | 23.08 | 3.85 | 26.92 | 11.54 | 30.77 | 19.23 | 3.85 | 19.23 | 26.92 | 19.23 | 7.69 | 15.38 | 15.38 | 3.85 | 53.85 |
| 3ivv | 20.59 | 20.59 | 20.59 | 20.59 | 17.65 | 20.59 | 23.53 | 23.53 | 20.59 | 5.88 | 20.59 | 17.65 | 23.53 | 23.53 | 20.59 | 0.00 | 20.59 | 23.53 | 20.59 | 26.47 |
| 3kmr | 0.00 | 0.00 | 0.00 | 0.00 | 0.00 | 0.00 | 0.00 | 0.00 | 0.00 | 0.00 | 0.00 | 0.00 | 0.00 | 0.00 | 0.00 | 0.00 | 0.00 | 0.00 | 0.00 | 0.00 |
| 3kuj | 11.36 | 18.18 | 15.91 | 6.82 | 15.91 | 20.45 | 6.82 | 9.09 | 13.64 | 9.09 | 9.09 | 11.36 | 13.64 | 9.09 | 0.00 | 11.36 | 0.00 | 0.00 | 4.55 | 11.36 |
| 3kus | 2.22 | 2.22 | 2.22 | 13.33 | 11.11 | 8.89 | 17.78 | 13.33 | 8.89 | 8.89 | 2.22 | 20.00 | 4.44 | 17.78 | 4.44 | 2.22 | 2.22 | 4.44 | 13.33 | 2.22 |
| 3l0e | 0.00 | 0.00 | 0.00 | 4.88 | 0.00 | 0.00 | 0.00 | 0.00 | 0.00 | 0.00 | 0.00 | 0.00 | 12.20 | 2.44 | 0.00 | 0.00 | 0.00 | 0.00 | 0.00 | 0.00 |
| 3ll8 | 0.00 | 0.00 | 0.00 | 0.00 | 0.00 | 0.00 | 0.00 | 0.00 | 0.00 | 0.00 | 0.00 | 0.00 | 0.00 | 0.00 | 0.00 | 0.00 | 0.00 | 0.00 | 0.00 | 0.00 |
| 3llz | 23.08 | 17.95 | 12.82 | 12.82 | 20.51 | 20.51 | 7.69 | 17.95 | 17.95 | 20.51 | 17.95 | 15.38 | 20.51 | 20.51 | 15.38 | 17.95 | 17.95 | 0.00 | 17.95 | 20.51 |
| 3obq | 33.33 | 27.78 | 33.33 | 33.33 | 36.11 | 30.56 | 0.00 | 0.00 | 58.33 | 0.00 | 0.00 | 0.00 | 33.33 | 0.00 | 0.00 | 0.00 | 0.00 | 41.67 | 33.33 | 0.00 |
| 3olf | 34.38 | 0.00 | 31.25 | 31.25 | 0.00 | 37.50 | 18.75 | 0.00 | 0.00 | 0.00 | 0.00 | 0.00 | 40.63 | 0.00 | 0.00 | 0.00 | 0.00 | 0.00 | 34.38 | 0.00 |
| 3p72 | 12.20 | 4.88 | 2.44 | 9.76 | 14.63 | 7.32 | 19.51 | 14.63 | 29.27 | 31.71 | 19.51 | 24.39 | 12.20 | 17.07 | 12.20 | 9.76 | 19.51 | 24.39 | 36.59 | 0.00 |
| 3p8f | 0.00 | 0.00 | 14.71 | 14.71 | 16.18 | 19.12 | 0.00 | 32.35 | 10.29 | 0.00 | 0.00 | 2.94 | 7.35 | 1.47 | 23.53 | 29.41 | 11.76 | 2.94 | 10.29 | 1.47 |
| 3ptl | 0.00 | 0.00 | 0.00 | 1.47 | 0.00 | 1.47 | 0.00 | 1.47 | 2.94 | 2.94 | 2.94 | 1.47 | 0.00 | 2.94 | 2.94 | 1.47 | 0.00 | 0.00 | 1.47 | 2.94 |
| 3qis | 0.00 | 0.00 | 23.53 | 26.47 | 47.06 | 0.00 | 35.29 | 26.47 | 29.41 | 23.53 | 32.35 | 29.41 | 20.59 | 20.59 | 26.47 | 35.29 | 38.24 | 0.00 | 0.00 | 23.53 |
| 3rm1 | 13.89 | 8.33 | 16.67 | 0.00 | 2.78 | 2.78 | 2.78 | 8.33 | 2.78 | 2.78 | 2.78 | 13.89 | 0.00 | 25.00 | 0.00 | 2.78 | 2.78 | 0.00 | 0.00 | 22.22 |
| 3rqg | 0.00 | 0.00 | 0.00 | 0.00 | 0.00 | 0.00 | 0.00 | 0.00 | 0.00 | 0.00 | 0.00 | 0.00 | 0.00 | 0.00 | 0.00 | 0.00 | 0.00 | 0.00 | 0.00 | 0.00 |
| 3sfj | 18.18 | 18.18 | 20.45 | 20.45 | 20.45 | 20.45 | 18.18 | 15.91 | 20.45 | 18.18 | 18.18 | 20.45 | 20.45 | 15.91 | 20.45 | 22.73 | 20.45 | 18.18 | 27.27 | 18.18 |
| 3so6 | 40.68 | 42.37 | 40.68 | 15.25 | 1.69 | 13.56 | 0.00 | 0.00 | 0.00 | 0.00 | 0.00 | 1.69 | 0.00 | 1.69 | 8.47 | 0.00 | 6.78 | 0.00 | 0.00 | 13.56 |
| 3tjv | 9.38 | 9.38 | 70.31 | 56.25 | 9.38 | 46.88 | 18.75 | 48.44 | 9.38 | 9.38 | 0.00 | 10.94 | 10.94 | 0.00 | 6.25 | 0.00 | 0.00 | 10.94 | 0.00 | 7.81 |
| 3tzy | 0.00 | 0.00 | 0.00 | 46.88 | 31.25 | 6.25 | 0.00 | 6.25 | 3.13 | 6.25 | 3.13 | 0.00 | 0.00 | 9.38 | 9.38 | 0.00 | 3.13 | 3.13 | 3.13 | 28.13 |
| 3u9q | 0.00 | 0.00 | 0.00 | 0.00 | 3.03 | 0.00 | 0.00 | 21.21 | 21.21 | 0.00 | 27.27 | 9.09 | 21.21 | 9.09 | 15.15 | 21.21 | 0.00 | 0.00 | 3.03 | 24.24 |
| 3up3 | 0.00 | 0.00 | 0.00 | 0.00 | 0.00 | 0.00 | 0.00 | 0.00 | 0.00 | 0.00 | 13.89 | 0.00 | 0.00 | 13.89 | 0.00 | 13.89 | 13.89 | 0.00 | 13.89 | 13.89 |
| 3v2x | 6.67 | 4.44 | 4.44 | 6.67 | 8.89 | 4.44 | 4.44 | 6.67 | 4.44 | 6.67 | 6.67 | 4.44 | 6.67 | 6.67 | 0.00 | 2.22 | 4.44 | 17.78 | 20.00 | 15.56 |
| 3vtc | 0.00 | 6.06 | 0.00 | 0.00 | 0.00 | 0.00 | 0.00 | 0.00 | 12.12 | 0.00 | 0.00 | 0.00 | 9.09 | 18.18 | 0.00 | 9.09 | 9.09 | 0.00 | 18.18 | 0.00 |
| 3w1b | 0.00 | 0.00 | 0.00 | 0.00 | 0.00 | 0.00 | 0.00 | 0.00 | 0.00 | 0.00 | 0.00 | 0.00 | 0.00 | 0.00 | 0.00 | 0.00 | 0.00 | 0.00 | 0.00 | 0.00 |
| 3zqh | 19.35 | 12.90 | 19.35 | 0.00 | 12.90 | 12.90 | 19.35 | 12.90 | 9.68 | 12.90 | 0.00 | 38.71 | 3.23 | 6.45 | 12.90 | 19.35 | 6.45 | 16.13 | 9.68 | 0.00 |
| 4b4n | 0.00 | 0.00 | 0.00 | 0.00 | 0.00 | 0.00 | 2.44 | 0.00 | 0.00 | 0.00 | 0.00 | 0.00 | 0.00 | 0.00 | 0.00 | 0.00 | 0.00 | 0.00 | 0.00 | 4.88 |
| 4dcb | 0.00 | 0.00 | 0.00 | 0.00 | 0.00 | 0.00 | 0.00 | 0.00 | 0.00 | 0.00 | 0.00 | 0.00 | 0.00 | 0.00 | 0.00 | 0.00 | 0.00 | 0.00 | 0.00 | 0.00 |
| 4e34 | 5.41 | 2.70 | 2.70 | 2.70 | 5.41 | 5.41 | 2.70 | 5.41 | 2.70 | 2.70 | 2.70 | 5.41 | 5.41 | 5.41 | 5.41 | 5.41 | 5.41 | 2.70 | 8.11 | 5.41 |
| 4eik | 66.67 | 61.54 | 53.85 | 28.21 | 25.64 | 20.51 | 20.51 | 35.90 | 28.21 | 23.08 | 74.36 | 64.10 | 28.21 | 7.69 | 61.54 | 20.51 | 41.03 | 15.38 | 46.15 | 38.46 |
| 4ery | 20.83 | 14.58 | 14.58 | 18.75 | 14.58 | 14.58 | 18.75 | 2.08 | 8.33 | 14.58 | 18.75 | 12.50 | 0.00 | 0.00 | 18.75 | 20.83 | 0.00 | 4.17 | 16.67 | 2.08 |
| 4f14 | 24.14 | 34.48 | 48.28 | 27.59 | 27.59 | 34.48 | 31.03 | 31.03 | 27.59 | 79.31 | 27.59 | 31.03 | 31.03 | 75.86 | 51.72 | 27.59 | 34.48 | 34.48 | 58.62 | 31.03 |
| 4f1z | 2.30 | 0.00 | 10.34 | 1.15 | 1.15 | 4.60 | 0.00 | 0.00 | 5.75 | 0.00 | 0.00 | 3.45 | 2.30 | 4.60 | 0.00 | 0.00 | 0.00 | 1.15 | 4.60 | 0.00 |
| 4gq6 | 5.36 | 0.00 | 5.36 | 7.14 | 7.14 | 8.93 | 7.14 | 7.14 | 7.14 | 7.14 | 7.14 | 7.14 | 7.14 | 7.14 | 7.14 | 7.14 | 7.14 | 7.14 | 7.14 | 7.14 |
| 4gxl | 0.00 | 0.00 | 0.00 | 0.00 | 21.05 | 0.00 | 0.00 | 0.00 | 0.00 | 0.00 | 0.00 | 15.79 | 2.63 | 0.00 | 0.00 | 23.68 | 7.89 | 13.16 | 0.00 | 28.95 |
| 4gyw | 7.69 | 0.00 | 5.77 | 9.62 | 5.77 | 0.00 | 0.00 | 0.00 | 0.00 | 0.00 | 0.00 | 0.00 | 0.00 | 1.92 | 0.00 | 0.00 | 0.00 | 0.00 | 0.00 | 9.62 |
| 4h4f | 0.00 | 10.64 | 0.00 | 8.51 | 14.89 | 10.64 | 0.00 | 10.64 | 10.64 | 17.02 | 8.51 | 12.77 | 12.77 | 14.89 | 8.51 | 19.15 | 17.02 | 12.77 | 17.02 | 10.64 |
| 4hom | 15.66 | 15.66 | 0.00 | 0.00 | 0.00 | 0.00 | 16.87 | 0.00 | 0.00 | 0.00 | 0.00 | 0.00 | 9.64 | 6.02 | 0.00 | 0.00 | 0.00 | 0.00 | 0.00 | 0.00 |
| 4htp | 10.00 | 13.33 | 10.00 | 6.67 | 3.33 | 6.67 | 3.33 | 3.33 | 6.67 | 6.67 | 3.33 | 6.67 | 6.67 | 6.67 | 10.00 | 10.00 | 3.33 | 0.00 | 6.67 | 6.67 |
| 4iim | 6.90 | 20.69 | 20.69 | 20.69 | 0.00 | 17.24 | 13.79 | 0.00 | 6.90 | 17.24 | 20.69 | 10.34 | 6.90 | 10.34 | 17.24 | 17.24 | 0.00 | 17.24 | 6.90 | 17.24 |
| 4j8s | 0.00 | 2.50 | 5.00 | 0.00 | 0.00 | 0.00 | 0.00 | 0.00 | 0.00 | 0.00 | 0.00 | 0.00 | 0.00 | 0.00 | 0.00 | 0.00 | 12.50 | 7.50 | 0.00 | 0.00 |
| 4k0u | 15.56 | 11.11 | 2.22 | 4.44 | 2.22 | 20.00 | 15.56 | 8.89 | 11.11 | 15.56 | 11.11 | 2.22 | 15.56 | 13.33 | 4.44 | 15.56 | 11.11 | 2.22 | 2.22 | 4.44 |

**S3(f). FNAT values of all 20 poses obtained after blind docking by FRODOCK on 133 protein-peptide complexes.**

| **ID** | **Pose1** | **Pose2** | **Pose3** | **Pose4** | **Pose5** | **Pose6** | **Pose7** | **Pose8** | **Pose9** | **Pose10** | **Pose11** | **Pose12** | **Pose13** | **Pose14** | **Pose15** | **Pose16** | **Pose17** | **Pose18** | **Pose19** | **Pose20** |
| --- | --- | --- | --- | --- | --- | --- | --- | --- | --- | --- | --- | --- | --- | --- | --- | --- | --- | --- | --- | --- |
| 1cjr | 83.67 | 12.24 | 44.90 | 42.86 | 6.12 | 0.00 | 0.00 | 0.00 | 0.00 | 2.04 | 0.00 | 0.00 | 0.00 | 0.00 | 14.29 | 0.00 | 12.24 | 8.16 | 0.00 | 12.24 |
| 1cka | 64.29 | 42.86 | 3.57 | 21.43 | 10.71 | 7.14 | 3.57 | 28.57 | 17.86 | 14.29 | 17.86 | 17.86 | 35.71 | 17.86 | 21.43 | 0.00 | 14.29 | 14.29 | 14.29 | 10.71 |
| 1cvu | 0.00 | 0.00 | 0.00 | 0.00 | 0.00 | 0.00 | 0.00 | 0.00 | 0.00 | 0.00 | 0.00 | 0.00 | 0.00 | 0.00 | 0.00 | 0.00 | 0.00 | 0.00 | 0.00 | 0.00 |
| 1d4t | 92.86 | 7.14 | 0.00 | 5.36 | 0.00 | 5.36 | 5.36 | 16.07 | 8.93 | 19.64 | 10.71 | 3.57 | 1.79 | 14.29 | 3.57 | 37.50 | 41.07 | 10.71 | 7.14 | 3.57 |
| 1eg4 | 0.00 | 42.42 | 9.09 | 0.00 | 9.09 | 18.18 | 87.88 | 0.00 | 6.06 | 0.00 | 6.06 | 21.21 | 0.00 | 0.00 | 0.00 | 18.18 | 6.06 | 9.09 | 0.00 | 0.00 |
| 1h6w | 92.96 | 15.49 | 12.68 | 8.45 | 9.86 | 14.08 | 16.90 | 1.41 | 0.00 | 15.49 | 5.63 | 5.63 | 23.94 | 11.27 | 26.76 | 7.04 | 0.00 | 1.41 | 12.68 | 23.94 |
| 1hc9 | 95.56 | 4.44 | 15.56 | 0.00 | 6.67 | 0.00 | 11.11 | 0.00 | 0.00 | 0.00 | 2.22 | 0.00 | 0.00 | 13.33 | 0.00 | 0.00 | 0.00 | 0.00 | 0.00 | 2.22 |
| 1jbu | 81.97 | 0.00 | 0.00 | 0.00 | 57.38 | 0.00 | 0.00 | 0.00 | 0.00 | 0.00 | 0.00 | 0.00 | 0.00 | 0.00 | 0.00 | 0.00 | 0.00 | 0.00 | 0.00 | 27.87 |
| 1k5n | 88.89 | 0.00 | 0.00 | 7.41 | 0.00 | 0.00 | 0.00 | 25.93 | 0.00 | 0.00 | 7.41 | 0.00 | 0.00 | 11.11 | 0.00 | 0.00 | 0.00 | 0.00 | 0.00 | 0.00 |
| 1mfg | 11.76 | 23.53 | 20.59 | 5.88 | 2.94 | 79.41 | 2.94 | 23.53 | 8.82 | 0.00 | 5.88 | 0.00 | 11.76 | 11.76 | 0.00 | 17.65 | 0.00 | 5.88 | 5.88 | 0.00 |
| 1nln | 0.00 | 72.31 | 41.54 | 0.00 | 4.62 | 0.00 | 0.00 | 0.00 | 0.00 | 0.00 | 3.08 | 0.00 | 0.00 | 0.00 | 0.00 | 0.00 | 0.00 | 4.62 | 0.00 | 0.00 |
| 1nq7 | 0.00 | 0.00 | 0.00 | 0.00 | 0.00 | 0.00 | 0.00 | 96.97 | 6.06 | 0.00 | 3.03 | 0.00 | 0.00 | 0.00 | 0.00 | 6.06 | 0.00 | 0.00 | 3.03 | 0.00 |
| 1ntv | 84.78 | 10.87 | 4.35 | 41.30 | 6.52 | 13.04 | 6.52 | 10.87 | 32.61 | 8.70 | 15.22 | 0.00 | 15.22 | 19.57 | 4.35 | 10.87 | 2.17 | 10.87 | 0.00 | 2.17 |
| 1nx1 | 77.50 | 0.00 | 0.00 | 0.00 | 0.00 | 0.00 | 20.00 | 25.00 | 0.00 | 0.00 | 0.00 | 0.00 | 0.00 | 0.00 | 12.50 | 0.00 | 0.00 | 0.00 | 22.50 | 5.00 |
| 1oai | 41.94 | 0.00 | 0.00 | 3.23 | 0.00 | 0.00 | 0.00 | 12.90 | 70.97 | 3.23 | 6.45 | 0.00 | 61.29 | 0.00 | 12.90 | 3.23 | 19.35 | 25.81 | 0.00 | 3.23 |
| 1oj5 | 44.00 | 64.00 | 20.00 | 8.00 | 32.00 | 0.00 | 16.00 | 12.00 | 0.00 | 0.00 | 16.00 | 0.00 | 16.00 | 12.00 | 0.00 | 12.00 | 8.00 | 12.00 | 20.00 | 0.00 |
| 1ou8 | 82.22 | 42.22 | 0.00 | 2.22 | 0.00 | 0.00 | 0.00 | 0.00 | 20.00 | 17.78 | 0.00 | 6.67 | 13.33 | 22.22 | 0.00 | 0.00 | 0.00 | 0.00 | 0.00 | 4.44 |
| 1ow6 | 25.00 | 0.00 | 0.00 | 16.67 | 0.00 | 0.00 | 8.33 | 0.00 | 0.00 | 0.00 | 33.33 | 8.33 | 33.33 | 0.00 | 16.67 | 0.00 | 0.00 | 0.00 | 0.00 | 0.00 |
| 1pzl | 0.00 | 0.00 | 0.00 | 0.00 | 0.00 | 76.67 | 40.00 | 0.00 | 0.00 | 0.00 | 0.00 | 0.00 | 0.00 | 0.00 | 0.00 | 0.00 | 0.00 | 0.00 | 0.00 | 0.00 |
| 1qkz | 15.38 | 10.26 | 20.51 | 15.38 | 5.13 | 15.38 | 12.82 | 12.82 | 7.69 | 12.82 | 12.82 | 7.69 | 20.51 | 15.38 | 15.38 | 7.69 | 0.00 | 12.82 | 5.13 | 25.64 |
| 1rst | 24.14 | 20.69 | 6.90 | 3.45 | 37.93 | 31.03 | 6.90 | 34.48 | 3.45 | 75.86 | 6.90 | 6.90 | 0.00 | 27.59 | 13.79 | 6.90 | 3.45 | 31.03 | 55.17 | 3.45 |
| 1rxz | 81.48 | 9.26 | 7.41 | 25.93 | 0.00 | 18.52 | 3.70 | 0.00 | 0.00 | 5.56 | 3.70 | 9.26 | 16.67 | 0.00 | 11.11 | 9.26 | 0.00 | 18.52 | 3.70 | 5.56 |
| 1sfi | 15.79 | 14.04 | 5.26 | 68.42 | 10.53 | 0.00 | 1.75 | 5.26 | 5.26 | 0.00 | 8.77 | 0.00 | 10.53 | 21.05 | 0.00 | 0.00 | 7.02 | 1.75 | 0.00 | 12.28 |
| 1ssh | 100.00 | 10.00 | 16.67 | 23.33 | 13.33 | 26.67 | 10.00 | 56.67 | 40.00 | 16.67 | 16.67 | 20.00 | 46.67 | 16.67 | 0.00 | 30.00 | 6.67 | 23.33 | 16.67 | 13.33 |
| 1t08 | 74.63 | 1.49 | 0.00 | 0.00 | 7.46 | 0.00 | 0.00 | 4.48 | 0.00 | 17.91 | 2.99 | 0.00 | 4.48 | 1.49 | 0.00 | 0.00 | 0.00 | 5.97 | 0.00 | 0.00 |
| 1t4f | 100.00 | 0.00 | 25.00 | 3.57 | 7.14 | 46.43 | 10.71 | 0.00 | 10.71 | 3.57 | 17.86 | 3.57 | 3.57 | 14.29 | 0.00 | 3.57 | 17.86 | 7.14 | 0.00 | 10.71 |
| 1t7r | 90.32 | 0.00 | 0.00 | 0.00 | 0.00 | 0.00 | 0.00 | 0.00 | 0.00 | 0.00 | 0.00 | 0.00 | 0.00 | 0.00 | 0.00 | 0.00 | 0.00 | 61.29 | 0.00 | 0.00 |
| 1tfc | 83.87 | 0.00 | 3.23 | 3.23 | 0.00 | 0.00 | 3.23 | 0.00 | 0.00 | 0.00 | 0.00 | 0.00 | 0.00 | 6.45 | 0.00 | 0.00 | 0.00 | 41.94 | 0.00 | 0.00 |
| 1u00 | 77.08 | 16.67 | 29.17 | 37.50 | 29.17 | 18.75 | 12.50 | 6.25 | 12.50 | 10.42 | 2.08 | 39.58 | 4.17 | 18.75 | 0.00 | 0.00 | 29.17 | 0.00 | 0.00 | 6.25 |
| 1uj0 | 88.89 | 25.93 | 11.11 | 14.81 | 33.33 | 11.11 | 51.85 | 14.81 | 11.11 | 44.44 | 29.63 | 14.81 | 14.81 | 11.11 | 0.00 | 7.41 | 18.52 | 22.22 | 33.33 | 18.52 |
| 1x2r | 39.53 | 4.65 | 13.95 | 25.58 | 4.65 | 0.00 | 13.95 | 0.00 | 18.60 | 9.30 | 13.95 | 0.00 | 4.65 | 6.98 | 4.65 | 6.98 | 9.30 | 4.65 | 0.00 | 0.00 |
| 1xoc | 84.71 | 0.00 | 0.00 | 0.00 | 0.00 | 0.00 | 0.00 | 22.35 | 0.00 | 0.00 | 0.00 | 0.00 | 0.00 | 1.18 | 0.00 | 0.00 | 0.00 | 0.00 | 0.00 | 0.00 |
| 1ymt | 82.14 | 21.43 | 0.00 | 0.00 | 0.00 | 0.00 | 0.00 | 0.00 | 0.00 | 0.00 | 0.00 | 0.00 | 0.00 | 0.00 | 0.00 | 0.00 | 0.00 | 28.57 | 0.00 | 0.00 |
| 1yuc | 97.22 | 5.56 | 0.00 | 0.00 | 19.44 | 0.00 | 0.00 | 0.00 | 2.78 | 0.00 | 0.00 | 0.00 | 0.00 | 2.78 | 0.00 | 0.00 | 0.00 | 8.33 | 0.00 | 27.78 |
| 1ywo | 84.00 | 28.00 | 20.00 | 20.00 | 20.00 | 48.00 | 16.00 | 16.00 | 28.00 | 24.00 | 24.00 | 40.00 | 0.00 | 36.00 | 20.00 | 16.00 | 0.00 | 0.00 | 16.00 | 20.00 |
| 2a25 | 80.00 | 0.00 | 17.50 | 20.00 | 0.00 | 0.00 | 20.00 | 12.50 | 0.00 | 0.00 | 0.00 | 25.00 | 37.50 | 0.00 | 10.00 | 0.00 | 27.50 | 2.50 | 0.00 | 22.50 |
| 2a3i | 0.00 | 0.00 | 0.00 | 0.00 | 0.00 | 93.75 | 0.00 | 0.00 | 0.00 | 0.00 | 0.00 | 0.00 | 0.00 | 0.00 | 0.00 | 0.00 | 0.00 | 0.00 | 0.00 | 0.00 |
| 2aq9 | 0.00 | 0.00 | 0.00 | 0.00 | 0.00 | 0.00 | 0.00 | 5.00 | 0.00 | 0.00 | 0.00 | 0.00 | 0.00 | 0.00 | 0.00 | 0.00 | 0.00 | 0.00 | 0.00 | 0.00 |
| 2b9h | 0.00 | 77.55 | 0.00 | 0.00 | 20.41 | 0.00 | 0.00 | 0.00 | 0.00 | 0.00 | 0.00 | 0.00 | 0.00 | 0.00 | 18.37 | 0.00 | 0.00 | 0.00 | 14.29 | 22.45 |
| 2bba | 50.88 | 0.00 | 0.00 | 54.39 | 0.00 | 0.00 | 0.00 | 0.00 | 15.79 | 0.00 | 0.00 | 0.00 | 0.00 | 0.00 | 0.00 | 0.00 | 0.00 | 26.32 | 0.00 | 7.02 |
| 2cch | 0.00 | 0.00 | 0.00 | 0.00 | 0.00 | 90.70 | 0.00 | 0.00 | 0.00 | 0.00 | 0.00 | 0.00 | 0.00 | 0.00 | 0.00 | 0.00 | 0.00 | 0.00 | 0.00 | 0.00 |
| 2ce8 | 0.00 | 83.87 | 0.00 | 0.00 | 0.00 | 0.00 | 0.00 | 0.00 | 0.00 | 0.00 | 0.00 | 0.00 | 0.00 | 0.00 | 0.00 | 0.00 | 0.00 | 0.00 | 0.00 | 0.00 |
| 2d0n | 76.00 | 40.00 | 24.00 | 8.00 | 52.00 | 20.00 | 0.00 | 0.00 | 16.00 | 12.00 | 36.00 | 20.00 | 40.00 | 4.00 | 44.00 | 8.00 | 4.00 | 32.00 | 4.00 | 8.00 |
| 2drk | 6.67 | 70.00 | 10.00 | 6.67 | 10.00 | 16.67 | 13.33 | 10.00 | 13.33 | 13.33 | 10.00 | 0.00 | 16.67 | 23.33 | 16.67 | 13.33 | 13.33 | 20.00 | 20.00 | 3.33 |
| 2dyp | 3.08 | 16.92 | 1.54 | 3.08 | 0.00 | 4.62 | 0.00 | 23.08 | 0.00 | 0.00 | 15.38 | 0.00 | 0.00 | 1.54 | 0.00 | 0.00 | 0.00 | 0.00 | 0.00 | 1.54 |
| 2fff | 73.91 | 0.00 | 0.00 | 0.00 | 0.00 | 0.00 | 6.52 | 10.87 | 0.00 | 13.04 | 0.00 | 0.00 | 6.52 | 0.00 | 0.00 | 19.57 | 0.00 | 0.00 | 8.70 | 0.00 |
| 2ffu | 84.62 | 0.00 | 43.59 | 15.38 | 10.26 | 15.38 | 0.00 | 12.82 | 7.69 | 0.00 | 15.38 | 23.08 | 38.46 | 0.00 | 15.38 | 0.00 | 17.95 | 33.33 | 0.00 | 10.26 |
| 2fka | 47.62 | 0.00 | 0.00 | 0.00 | 0.00 | 19.05 | 0.00 | 9.52 | 0.00 | 0.00 | 0.00 | 0.00 | 4.76 | 0.00 | 0.00 | 23.81 | 19.05 | 0.00 | 0.00 | 0.00 |
| 2fmf | 40.91 | 18.18 | 95.45 | 13.64 | 0.00 | 0.00 | 36.36 | 13.64 | 18.18 | 22.73 | 0.00 | 18.18 | 0.00 | 0.00 | 0.00 | 0.00 | 0.00 | 0.00 | 18.18 | 0.00 |
| 2fts | 0.00 | 92.45 | 0.00 | 5.66 | 0.00 | 69.81 | 0.00 | 0.00 | 7.55 | 20.75 | 33.96 | 0.00 | 1.89 | 0.00 | 1.89 | 0.00 | 33.96 | 0.00 | 11.32 | 1.89 |
| 2fvj | 0.00 | 93.75 | 0.00 | 0.00 | 0.00 | 0.00 | 0.00 | 0.00 | 0.00 | 0.00 | 0.00 | 0.00 | 0.00 | 0.00 | 0.00 | 0.00 | 0.00 | 0.00 | 0.00 | 0.00 |
| 2ho2 | 35.29 | 23.53 | 11.76 | 17.65 | 29.41 | 35.29 | 23.53 | 35.29 | 35.29 | 29.41 | 5.88 | 5.88 | 5.88 | 11.76 | 35.29 | 0.00 | 0.00 | 5.88 | 41.18 | 29.41 |
| 2ht9 | 0.00 | 7.69 | 0.00 | 0.00 | 0.00 | 0.00 | 0.00 | 7.69 | 38.46 | 0.00 | 0.00 | 0.00 | 46.15 | 0.00 | 38.46 | 0.00 | 0.00 | 0.00 | 0.00 | 0.00 |
| 2o02 | 2.82 | 0.00 | 0.00 | 56.34 | 14.08 | 0.00 | 0.00 | 0.00 | 0.00 | 0.00 | 0.00 | 1.41 | 0.00 | 0.00 | 0.00 | 0.00 | 12.68 | 1.41 | 0.00 | 2.82 |
| 2o4j | 0.00 | 0.00 | 0.00 | 5.88 | 0.00 | 2.94 | 0.00 | 0.00 | 0.00 | 0.00 | 0.00 | 0.00 | 0.00 | 0.00 | 0.00 | 0.00 | 0.00 | 0.00 | 0.00 | 0.00 |
| 2o9v | 84.00 | 12.00 | 20.00 | 8.00 | 36.00 | 8.00 | 24.00 | 0.00 | 20.00 | 4.00 | 20.00 | 8.00 | 16.00 | 0.00 | 20.00 | 12.00 | 24.00 | 24.00 | 16.00 | 44.00 |
| 2oei | 31.82 | 13.64 | 31.82 | 18.18 | 31.82 | 68.18 | 13.64 | 72.73 | 18.18 | 13.64 | 45.45 | 9.09 | 18.18 | 0.00 | 22.73 | 9.09 | 27.27 | 27.27 | 27.27 | 9.09 |
| 2p0w | 87.50 | 0.00 | 0.00 | 10.94 | 0.00 | 0.00 | 0.00 | 0.00 | 0.00 | 3.13 | 0.00 | 6.25 | 0.00 | 6.25 | 0.00 | 0.00 | 0.00 | 0.00 | 6.25 | 1.56 |
| 2p1o | 31.11 | 4.44 | 57.78 | 15.56 | 11.11 | 15.56 | 11.11 | 8.89 | 20.00 | 75.56 | 17.78 | 6.67 | 2.22 | 20.00 | 17.78 | 6.67 | 13.33 | 17.78 | 2.22 | 0.00 |
| 2p1t | 0.00 | 0.00 | 5.71 | 0.00 | 0.00 | 0.00 | 0.00 | 14.29 | 0.00 | 0.00 | 0.00 | 0.00 | 0.00 | 0.00 | 0.00 | 0.00 | 0.00 | 2.86 | 0.00 | 0.00 |
| 2p54 | 92.11 | 0.00 | 0.00 | 0.00 | 0.00 | 0.00 | 0.00 | 0.00 | 0.00 | 0.00 | 2.63 | 0.00 | 0.00 | 0.00 | 0.00 | 0.00 | 0.00 | 0.00 | 0.00 | 2.63 |
| 2peh | 94.12 | 50.00 | 0.00 | 0.00 | 50.00 | 11.76 | 0.00 | 0.00 | 2.94 | 26.47 | 2.94 | 0.00 | 38.24 | 0.00 | 8.82 | 8.82 | 2.94 | 0.00 | 0.00 | 2.94 |
| 2pux | 97.30 | 0.00 | 0.00 | 0.00 | 0.00 | 0.00 | 0.00 | 0.00 | 2.70 | 0.00 | 0.00 | 0.00 | 0.00 | 0.00 | 0.00 | 0.00 | 0.00 | 0.00 | 2.70 | 0.00 |
| 2puy | 83.78 | 8.11 | 8.11 | 0.00 | 5.41 | 13.51 | 0.00 | 2.70 | 2.70 | 5.41 | 5.41 | 0.00 | 0.00 | 29.73 | 10.81 | 2.70 | 10.81 | 0.00 | 0.00 | 5.41 |
| 2qbx | 11.86 | 50.85 | 0.00 | 18.64 | 0.00 | 1.69 | 20.34 | 0.00 | 23.73 | 0.00 | 0.00 | 0.00 | 0.00 | 0.00 | 0.00 | 0.00 | 0.00 | 0.00 | 0.00 | 32.20 |
| 2qos | 88.68 | 24.53 | 9.43 | 7.55 | 7.55 | 0.00 | 0.00 | 24.53 | 16.98 | 0.00 | 7.55 | 0.00 | 20.75 | 0.00 | 11.32 | 0.00 | 0.00 | 13.21 | 16.98 | 18.87 |
| 2qse | 90.00 | 0.00 | 0.00 | 0.00 | 0.00 | 0.00 | 0.00 | 16.67 | 0.00 | 0.00 | 0.00 | 0.00 | 0.00 | 0.00 | 0.00 | 13.33 | 0.00 | 0.00 | 0.00 | 0.00 |
| 2r7g | 91.49 | 0.00 | 0.00 | 0.00 | 2.13 | 0.00 | 2.13 | 0.00 | 0.00 | 0.00 | 0.00 | 0.00 | 0.00 | 0.00 | 0.00 | 0.00 | 0.00 | 0.00 | 10.64 | 0.00 |
| 2r9q | 62.50 | 12.50 | 28.13 | 43.75 | 25.00 | 15.63 | 0.00 | 3.13 | 0.00 | 9.38 | 0.00 | 18.75 | 0.00 | 25.00 | 31.25 | 28.13 | 34.38 | 0.00 | 0.00 | 9.38 |
| 2v8y | 58.54 | 19.51 | 4.88 | 17.07 | 4.88 | 7.32 | 7.32 | 31.71 | 70.73 | 0.00 | 2.44 | 14.63 | 14.63 | 2.44 | 9.76 | 14.63 | 29.27 | 0.00 | 17.07 | 19.51 |
| 2vkn | 77.27 | 0.00 | 22.73 | 27.27 | 0.00 | 18.18 | 31.82 | 18.18 | 4.55 | 0.00 | 13.64 | 13.64 | 45.45 | 13.64 | 4.55 | 0.00 | 9.09 | 22.73 | 9.09 | 9.09 |
| 2vr3 | 5.95 | 73.81 | 9.52 | 3.57 | 0.00 | 2.38 | 7.14 | 0.00 | 0.00 | 5.95 | 0.00 | 5.95 | 0.00 | 3.57 | 3.57 | 11.90 | 0.00 | 1.19 | 4.76 | 3.57 |
| 2vwf | 82.86 | 0.00 | 0.00 | 0.00 | 0.00 | 0.00 | 0.00 | 0.00 | 0.00 | 8.57 | 22.86 | 0.00 | 0.00 | 14.29 | 14.29 | 0.00 | 0.00 | 0.00 | 11.43 | 0.00 |
| 2w2u | 93.62 | 29.79 | 19.15 | 12.77 | 0.00 | 12.77 | 6.38 | 6.38 | 0.00 | 23.40 | 8.51 | 34.04 | 12.77 | 2.13 | 2.13 | 0.00 | 25.53 | 14.89 | 4.26 | 12.77 |
| 2whx | 74.07 | 2.47 | 1.23 | 0.00 | 0.00 | 0.00 | 0.00 | 1.23 | 0.00 | 0.00 | 16.05 | 0.00 | 0.00 | 1.23 | 0.00 | 34.57 | 0.00 | 0.00 | 0.00 | 0.00 |
| 2xrw | 97.50 | 0.00 | 0.00 | 0.00 | 0.00 | 0.00 | 0.00 | 7.50 | 0.00 | 0.00 | 0.00 | 37.50 | 0.00 | 0.00 | 5.00 | 0.00 | 7.50 | 2.50 | 5.00 | 0.00 |
| 2xu7 | 77.55 | 44.90 | 34.69 | 32.65 | 0.00 | 24.49 | 14.29 | 0.00 | 0.00 | 12.24 | 46.94 | 28.57 | 0.00 | 10.20 | 0.00 | 0.00 | 0.00 | 0.00 | 14.29 | 8.16 |
| 2xvc | 82.35 | 31.37 | 25.49 | 0.00 | 25.49 | 0.00 | 25.49 | 21.57 | 19.61 | 0.00 | 0.00 | 0.00 | 0.00 | 0.00 | 0.00 | 0.00 | 7.84 | 31.37 | 50.98 | 0.00 |
| 2zjd | 0.00 | 0.00 | 100.00 | 0.00 | 0.00 | 0.00 | 20.51 | 0.00 | 0.00 | 10.26 | 0.00 | 0.00 | 0.00 | 0.00 | 0.00 | 0.00 | 0.00 | 0.00 | 0.00 | 0.00 |
| 3asl | 0.00 | 6.45 | 90.32 | 0.00 | 0.00 | 0.00 | 0.00 | 3.23 | 0.00 | 6.45 | 0.00 | 12.90 | 0.00 | 0.00 | 6.45 | 3.23 | 0.00 | 3.23 | 0.00 | 0.00 |
| 3awr | 0.00 | 0.00 | 0.00 | 3.23 | 0.00 | 3.23 | 0.00 | 0.00 | 0.00 | 0.00 | 0.00 | 0.00 | 0.00 | 0.00 | 9.68 | 9.68 | 58.06 | 9.68 | 0.00 | 9.68 |
| 3ayu | 80.00 | 18.33 | 10.00 | 8.33 | 15.00 | 8.33 | 1.67 | 0.00 | 3.33 | 8.33 | 31.67 | 10.00 | 6.67 | 0.00 | 16.67 | 20.00 | 10.00 | 0.00 | 20.00 | 1.67 |
| 3bfq | 92.71 | 6.25 | 8.33 | 4.17 | 11.46 | 14.58 | 3.13 | 17.71 | 4.17 | 0.00 | 0.00 | 9.38 | 0.00 | 28.13 | 2.08 | 46.88 | 0.00 | 1.04 | 13.54 | 2.08 |
| 3c3r | 93.10 | 0.00 | 0.00 | 0.00 | 0.00 | 0.00 | 0.00 | 0.00 | 0.00 | 17.24 | 0.00 | 37.93 | 0.00 | 27.59 | 0.00 | 0.00 | 6.90 | 0.00 | 13.79 | 0.00 |
| 3d32 | 0.00 | 83.33 | 38.10 | 9.52 | 2.38 | 0.00 | 14.29 | 11.90 | 0.00 | 0.00 | 0.00 | 0.00 | 11.90 | 0.00 | 0.00 | 0.00 | 11.90 | 0.00 | 0.00 | 0.00 |
| 3ds4 | 84.62 | 15.38 | 0.00 | 7.69 | 0.00 | 0.00 | 0.00 | 2.56 | 0.00 | 0.00 | 0.00 | 0.00 | 0.00 | 5.13 | 5.13 | 0.00 | 5.13 | 0.00 | 5.13 | 0.00 |
| 3ery | 85.94 | 14.06 | 10.94 | 17.19 | 40.63 | 9.38 | 14.06 | 29.69 | 10.94 | 15.63 | 9.38 | 25.00 | 10.94 | 3.13 | 10.94 | 10.94 | 10.94 | 1.56 | 3.13 | 7.81 |
| 3fdo | 100.00 | 6.67 | 16.67 | 3.33 | 40.00 | 20.00 | 20.00 | 3.33 | 10.00 | 0.00 | 10.00 | 3.33 | 13.33 | 0.00 | 0.00 | 10.00 | 0.00 | 0.00 | 10.00 | 36.67 |
| 3g2s | 97.37 | 0.00 | 0.00 | 10.53 | 15.79 | 13.16 | 0.00 | 2.63 | 0.00 | 0.00 | 0.00 | 0.00 | 0.00 | 2.63 | 0.00 | 0.00 | 23.68 | 2.63 | 2.63 | 0.00 |
| 3gyt | 96.55 | 0.00 | 0.00 | 0.00 | 0.00 | 0.00 | 6.90 | 34.48 | 0.00 | 0.00 | 27.59 | 17.24 | 6.90 | 3.45 | 17.24 | 6.90 | 3.45 | 13.79 | 31.03 | 6.90 |
| 3h1z | 0.00 | 7.55 | 5.66 | 1.89 | 0.00 | 0.00 | 0.00 | 0.00 | 5.66 | 5.66 | 11.32 | 0.00 | 1.89 | 1.89 | 0.00 | 7.55 | 0.00 | 0.00 | 11.32 | 3.77 |
| 3i5r | 19.23 | 96.15 | 19.23 | 19.23 | 0.00 | 3.85 | 23.08 | 19.23 | 30.77 | 26.92 | 19.23 | 26.92 | 26.92 | 15.38 | 7.69 | 19.23 | 15.38 | 15.38 | 15.38 | 7.69 |
| 3ivv | 35.29 | 88.24 | 32.35 | 23.53 | 23.53 | 20.59 | 14.71 | 0.00 | 20.59 | 14.71 | 20.59 | 8.82 | 11.76 | 5.88 | 35.29 | 20.59 | 17.65 | 8.82 | 26.47 | 14.71 |
| 3kmr | 0.00 | 0.00 | 0.00 | 0.00 | 0.00 | 76.67 | 0.00 | 0.00 | 0.00 | 0.00 | 0.00 | 0.00 | 0.00 | 0.00 | 0.00 | 0.00 | 0.00 | 0.00 | 0.00 | 0.00 |
| 3kuj | 0.00 | 36.36 | 20.45 | 2.27 | 4.55 | 0.00 | 18.18 | 18.18 | 0.00 | 0.00 | 29.55 | 2.27 | 13.64 | 0.00 | 0.00 | 11.36 | 0.00 | 0.00 | 2.27 | 2.27 |
| 3kus | 77.78 | 0.00 | 4.44 | 0.00 | 0.00 | 0.00 | 0.00 | 60.00 | 0.00 | 0.00 | 4.44 | 0.00 | 22.22 | 0.00 | 26.67 | 0.00 | 4.44 | 2.22 | 4.44 | 0.00 |
| 3l0e | 0.00 | 0.00 | 9.76 | 0.00 | 0.00 | 4.88 | 0.00 | 0.00 | 0.00 | 0.00 | 2.44 | 0.00 | 0.00 | 0.00 | 0.00 | 0.00 | 0.00 | 0.00 | 0.00 | 0.00 |
| 3ll8 | 0.00 | 0.00 | 0.00 | 0.00 | 0.00 | 0.00 | 0.00 | 0.00 | 0.00 | 0.00 | 0.00 | 0.00 | 0.00 | 0.00 | 0.00 | 0.00 | 0.00 | 0.00 | 0.00 | 0.00 |
| 3llz | 84.62 | 2.56 | 15.38 | 35.90 | 58.97 | 7.69 | 15.38 | 58.97 | 15.38 | 20.51 | 25.64 | 2.56 | 0.00 | 15.38 | 2.56 | 0.00 | 10.26 | 5.13 | 17.95 | 7.69 |
| 3obq | 72.22 | 19.44 | 22.22 | 33.33 | 0.00 | 36.11 | 2.78 | 13.89 | 0.00 | 33.33 | 36.11 | 0.00 | 5.56 | 16.67 | 11.11 | 0.00 | 0.00 | 0.00 | 25.00 | 2.78 |
| 3olf | 0.00 | 0.00 | 0.00 | 0.00 | 0.00 | 0.00 | 0.00 | 0.00 | 0.00 | 6.25 | 0.00 | 0.00 | 0.00 | 0.00 | 0.00 | 0.00 | 0.00 | 0.00 | 0.00 | 0.00 |
| 3p72 | 26.83 | 14.63 | 0.00 | 0.00 | 0.00 | 9.76 | 0.00 | 0.00 | 0.00 | 0.00 | 0.00 | 0.00 | 0.00 | 0.00 | 9.76 | 0.00 | 0.00 | 0.00 | 0.00 | 0.00 |
| 3p8f | 83.82 | 2.94 | 35.29 | 5.88 | 11.76 | 10.29 | 5.88 | 10.29 | 1.47 | 0.00 | 2.94 | 11.76 | 4.41 | 7.35 | 1.47 | 10.29 | 5.88 | 1.47 | 0.00 | 2.94 |
| 3ptl | 0.00 | 0.00 | 2.94 | 0.00 | 5.88 | 0.00 | 2.94 | 0.00 | 13.24 | 0.00 | 2.94 | 0.00 | 0.00 | 7.35 | 0.00 | 0.00 | 0.00 | 0.00 | 8.82 | 0.00 |
| 3qis | 20.59 | 14.71 | 41.18 | 5.88 | 14.71 | 26.47 | 38.24 | 11.76 | 0.00 | 0.00 | 8.82 | 38.24 | 0.00 | 0.00 | 0.00 | 17.65 | 5.88 | 14.71 | 0.00 | 14.71 |
| 3rm1 | 72.22 | 13.89 | 0.00 | 2.78 | 8.33 | 5.56 | 0.00 | 5.56 | 0.00 | 27.78 | 0.00 | 0.00 | 0.00 | 13.89 | 0.00 | 0.00 | 0.00 | 2.78 | 0.00 | 8.33 |
| 3rqg | 0.00 | 0.00 | 11.11 | 0.00 | 0.00 | 0.00 | 0.00 | 0.00 | 77.78 | 0.00 | 0.00 | 7.41 | 0.00 | 0.00 | 11.11 | 0.00 | 0.00 | 0.00 | 0.00 | 0.00 |
| 3sfj | 11.36 | 68.18 | 0.00 | 0.00 | 0.00 | 0.00 | 0.00 | 6.82 | 0.00 | 0.00 | 0.00 | 18.18 | 13.64 | 2.27 | 59.09 | 0.00 | 0.00 | 0.00 | 11.36 | 0.00 |
| 3so6 | 72.88 | 15.25 | 3.39 | 5.08 | 18.64 | 18.64 | 1.69 | 5.08 | 28.81 | 1.69 | 8.47 | 11.86 | 0.00 | 11.86 | 3.39 | 0.00 | 3.39 | 1.69 | 5.08 | 0.00 |
| 3tjv | 87.50 | 4.69 | 7.81 | 1.56 | 10.94 | 20.31 | 15.63 | 6.25 | 1.56 | 0.00 | 4.69 | 46.88 | 4.69 | 0.00 | 20.31 | 14.06 | 14.06 | 0.00 | 12.50 | 1.56 |
| 3tzy | 46.88 | 12.50 | 25.00 | 15.63 | 0.00 | 6.25 | 9.38 | 6.25 | 0.00 | 12.50 | 0.00 | 0.00 | 6.25 | 0.00 | 6.25 | 3.13 | 0.00 | 6.25 | 0.00 | 0.00 |
| 3u9q | 96.97 | 0.00 | 0.00 | 30.30 | 0.00 | 21.21 | 6.06 | 0.00 | 15.15 | 0.00 | 0.00 | 0.00 | 0.00 | 0.00 | 0.00 | 12.12 | 0.00 | 9.09 | 3.03 | 3.03 |
| 3up3 | 97.22 | 0.00 | 22.22 | 0.00 | 16.67 | 5.56 | 16.67 | 0.00 | 0.00 | 25.00 | 2.78 | 19.44 | 5.56 | 11.11 | 0.00 | 11.11 | 0.00 | 0.00 | 0.00 | 0.00 |
| 3v2x | 6.67 | 95.56 | 42.22 | 15.56 | 13.33 | 0.00 | 0.00 | 0.00 | 6.67 | 4.44 | 11.11 | 2.22 | 0.00 | 0.00 | 4.44 | 2.22 | 0.00 | 0.00 | 0.00 | 0.00 |
| 3vtc | 0.00 | 0.00 | 0.00 | 0.00 | 30.30 | 0.00 | 0.00 | 15.15 | 0.00 | 0.00 | 0.00 | 0.00 | 0.00 | 0.00 | 0.00 | 0.00 | 0.00 | 0.00 | 0.00 | 3.03 |
| 3w1b | 88.89 | 0.00 | 0.00 | 0.00 | 0.00 | 0.00 | 25.93 | 0.00 | 0.00 | 0.00 | 0.00 | 0.00 | 0.00 | 0.00 | 0.00 | 0.00 | 0.00 | 0.00 | 0.00 | 0.00 |
| 3zqh | 6.45 | 0.00 | 0.00 | 6.45 | 0.00 | 0.00 | 0.00 | 3.23 | 0.00 | 0.00 | 0.00 | 0.00 | 0.00 | 0.00 | 6.45 | 3.23 | 0.00 | 0.00 | 0.00 | 0.00 |
| 4b4n | 0.00 | 0.00 | 0.00 | 0.00 | 0.00 | 0.00 | 0.00 | 0.00 | 0.00 | 0.00 | 0.00 | 0.00 | 0.00 | 0.00 | 0.00 | 0.00 | 0.00 | 0.00 | 0.00 | 0.00 |
| 4dcb | 0.00 | 0.00 | 100.00 | 0.00 | 0.00 | 0.00 | 2.44 | 12.20 | 0.00 | 0.00 | 0.00 | 0.00 | 0.00 | 0.00 | 0.00 | 0.00 | 2.44 | 0.00 | 0.00 | 0.00 |
| 4e34 | 18.92 | 81.08 | 0.00 | 2.70 | 18.92 | 5.41 | 10.81 | 0.00 | 16.22 | 0.00 | 5.41 | 0.00 | 0.00 | 27.03 | 0.00 | 0.00 | 16.22 | 0.00 | 0.00 | 10.81 |
| 4eik | 92.31 | 12.82 | 28.21 | 5.13 | 5.13 | 12.82 | 15.38 | 17.95 | 17.95 | 7.69 | 17.95 | 7.69 | 15.38 | 10.26 | 7.69 | 2.56 | 15.38 | 20.51 | 0.00 | 10.26 |
| 4ery | 75.00 | 0.00 | 4.17 | 25.00 | 2.08 | 10.42 | 6.25 | 8.33 | 14.58 | 12.50 | 4.17 | 8.33 | 33.33 | 2.08 | 2.08 | 8.33 | 8.33 | 2.08 | 8.33 | 25.00 |
| 4f14 | 10.34 | 72.41 | 3.45 | 6.90 | 0.00 | 17.24 | 6.90 | 6.90 | 34.48 | 10.34 | 41.38 | 0.00 | 17.24 | 20.69 | 0.00 | 13.79 | 34.48 | 13.79 | 44.83 | 17.24 |
| 4f1z | 4.60 | 0.00 | 3.45 | 1.15 | 2.30 | 9.20 | 4.60 | 6.90 | 0.00 | 11.49 | 0.00 | 4.60 | 3.45 | 12.64 | 1.15 | 6.90 | 0.00 | 8.05 | 0.00 | 0.00 |
| 4gq6 | 14.29 | 1.79 | 73.21 | 23.21 | 0.00 | 17.86 | 10.71 | 1.79 | 1.79 | 0.00 | 12.50 | 0.00 | 25.00 | 8.93 | 8.93 | 0.00 | 3.57 | 0.00 | 0.00 | 0.00 |
| 4gxl | 2.63 | 0.00 | 86.84 | 0.00 | 0.00 | 18.42 | 0.00 | 0.00 | 21.05 | 0.00 | 0.00 | 0.00 | 0.00 | 0.00 | 2.63 | 0.00 | 21.05 | 2.63 | 2.63 | 0.00 |
| 4gyw | 25.00 | 80.77 | 3.85 | 0.00 | 0.00 | 3.85 | 0.00 | 23.08 | 7.69 | 1.92 | 1.92 | 3.85 | 3.85 | 0.00 | 5.77 | 1.92 | 13.46 | 5.77 | 0.00 | 0.00 |
| 4h4f | 0.00 | 12.77 | 0.00 | 0.00 | 0.00 | 12.77 | 82.98 | 17.02 | 25.53 | 0.00 | 4.26 | 17.02 | 0.00 | 0.00 | 0.00 | 2.13 | 0.00 | 12.77 | 4.26 | 0.00 |
| 4hom | 30.12 | 0.00 | 2.41 | 0.00 | 7.23 | 27.71 | 2.41 | 24.10 | 0.00 | 0.00 | 0.00 | 3.61 | 0.00 | 7.23 | 8.43 | 14.46 | 0.00 | 21.69 | 0.00 | 3.61 |
| 4htp | 80.00 | 0.00 | 0.00 | 0.00 | 0.00 | 0.00 | 0.00 | 0.00 | 23.33 | 3.33 | 0.00 | 0.00 | 0.00 | 0.00 | 0.00 | 20.00 | 0.00 | 0.00 | 0.00 | 0.00 |
| 4iim | 72.41 | 17.24 | 24.14 | 10.34 | 0.00 | 37.93 | 17.24 | 0.00 | 0.00 | 10.34 | 6.90 | 0.00 | 24.14 | 13.79 | 0.00 | 0.00 | 13.79 | 0.00 | 0.00 | 10.34 |
| 4j8s | 0.00 | 80.00 | 0.00 | 0.00 | 0.00 | 0.00 | 0.00 | 12.50 | 0.00 | 0.00 | 0.00 | 0.00 | 0.00 | 0.00 | 0.00 | 0.00 | 0.00 | 0.00 | 0.00 | 0.00 |
| 4k0u | 68.89 | 15.56 | 15.56 | 24.44 | 11.11 | 13.33 | 8.89 | 0.00 | 4.44 | 4.44 | 2.22 | 17.78 | 8.89 | 0.00 | 11.11 | 0.00 | 8.89 | 17.78 | 0.00 | 11.11 |

**S4(a). L-RMSD values of all 20 poses obtained after blind docking by ATTRACT on 133 protein-peptide complexes.**

| **ID** | **Pose1** | **Pose2** | **Pose3** | **Pose4** | **Pose5** | **Pose6** | **Pose7** | **Pose8** | **Pose9** | **Pose10** | **Pose11** | **Pose12** | **Pose13** | **Pose14** | **Pose15** | **Pose16** | **Pose17** | **Pose18** | **Pose19** | **Pose20** |
| --- | --- | --- | --- | --- | --- | --- | --- | --- | --- | --- | --- | --- | --- | --- | --- | --- | --- | --- | --- | --- |
| 1cjr | 21.51 | 25.69 | 21.28 | 29.32 | 25.69 | 21.36 | 21.25 | 21.87 | 21.78 | 26.86 | 22.50 | 29.23 | 19.66 | 29.23 | 15.77 | 21.98 | 17.92 | 15.60 | 29.09 | 28.75 |
| 1cka | 3.35 | 3.35 | 3.49 | 3.24 | 3.40 | 3.55 | 3.70 | 3.47 | 1.43 | 1.35 | 1.23 | 3.54 | 1.43 | 3.83 | 0.82 | 13.11 | 2.69 | 7.64 | 13.32 | 12.96 |
| 1cvu | 53.94 | 33.60 | 33.47 | 53.56 | 53.78 | 42.82 | 35.39 | 33.35 | 29.37 | 55.01 | 33.32 | 35.57 | 48.50 | 39.96 | 53.84 | 56.91 | 49.86 | 54.06 | 46.25 | 53.52 |
| 1d4t | 0.63 | 31.10 | 23.63 | 19.32 | 24.40 | 19.72 | 26.60 | 31.03 | 22.37 | 20.01 | 22.23 | 17.94 | 18.85 | 21.53 | 25.25 | 22.13 | 23.99 | 21.87 | 27.33 | 23.73 |
| 1eg4 | 40.86 | 35.18 | 15.77 | 12.14 | 16.88 | 30.40 | 34.19 | 17.51 | 15.84 | 38.62 | 25.47 | 41.81 | 24.61 | 19.64 | 30.17 | 29.39 | 7.89 | 40.26 | 14.34 | 12.74 |
| 1h6w | 40.66 | 15.34 | 12.33 | 20.00 | 20.05 | 46.96 | 16.29 | 19.95 | 46.41 | 13.11 | 13.99 | 46.47 | 46.02 | 18.09 | 13.95 | 40.06 | 13.19 | 32.81 | 36.49 | 36.57 |
| 1hc9 | 11.75 | 12.18 | 9.57 | 7.74 | 18.09 | 10.16 | 12.00 | 9.93 | 20.23 | 9.82 | 19.09 | 19.74 | 18.38 | 20.33 | 22.71 | 25.70 | 18.37 | 6.78 | 21.41 | 18.43 |
| 1jbu | 1.02 | 1.03 | 4.70 | 10.69 | 4.71 | 10.61 | 10.40 | 44.46 | 4.82 | 44.45 | 43.24 | 45.05 | 17.33 | 44.52 | 17.47 | 45.66 | 45.16 | 16.93 | 11.72 | 16.40 |
| 1k5n | 10.76 | 13.46 | 21.57 | 24.39 | 13.99 | 23.39 | 23.22 | 12.51 | 24.81 | 15.15 | 60.43 | 60.45 | 13.89 | 20.83 | 60.53 | 14.04 | 25.40 | 13.72 | 13.97 | 14.79 |
| 1mfg | 13.32 | 12.38 | 12.51 | 20.46 | 11.23 | 12.84 | 18.86 | 11.26 | 20.58 | 14.00 | 19.12 | 18.81 | 19.29 | 19.06 | 11.74 | 14.57 | 19.01 | 19.39 | 19.38 | 18.76 |
| 1nln | 0.92 | 3.34 | 6.67 | 6.56 | 3.24 | 9.12 | 5.07 | 15.24 | 15.04 | 15.04 | 15.08 | 15.07 | 15.39 | 15.58 | 15.86 | 3.58 | 9.95 | 9.03 | 9.99 | 18.53 |
| 1nq7 | 34.00 | 33.80 | 34.07 | 33.18 | 33.15 | 33.99 | 33.83 | 33.76 | 33.25 | 32.77 | 32.74 | 35.36 | 33.26 | 26.36 | 26.52 | 34.89 | 21.80 | 26.64 | 1.41 | 38.98 |
| 1ntv | 13.44 | 13.51 | 21.23 | 30.26 | 28.80 | 1.49 | 19.31 | 31.93 | 31.89 | 31.87 | 25.72 | 13.52 | 26.83 | 15.07 | 29.12 | 16.97 | 31.73 | 19.85 | 20.42 | 20.14 |
| 1nx1 | 34.46 | 34.57 | 34.79 | 34.99 | 34.79 | 35.46 | 36.93 | 36.37 | 35.94 | 37.16 | 35.41 | 7.88 | 36.76 | 38.55 | 2.54 | 6.75 | 3.00 | 3.03 | 36.72 | 37.61 |
| 1oai | 25.87 | 23.49 | 26.80 | 26.72 | 5.93 | 6.08 | 26.78 | 9.99 | 10.33 | 14.68 | 11.33 | 14.50 | 2.62 | 5.88 | 14.93 | 5.14 | 21.72 | 5.24 | 18.32 | 21.86 |
| 1oj5 | 13.14 | 25.97 | 25.92 | 1.02 | 26.15 | 26.02 | 24.39 | 25.24 | 24.21 | 24.09 | 26.29 | 16.56 | 29.72 | 16.72 | 16.44 | 24.85 | 25.46 | 29.77 | 29.75 | 25.17 |
| 1ou8 | 12.15 | 15.77 | 11.03 | 29.45 | 13.98 | 12.36 | 13.21 | 27.38 | 13.37 | 13.37 | 27.34 | 11.67 | 22.18 | 13.70 | 19.27 | 13.81 | 23.30 | 30.33 | 10.36 | 6.52 |
| 1ow6 | 28.09 | 28.15 | 28.50 | 28.20 | 28.41 | 29.51 | 11.20 | 11.47 | 12.08 | 11.59 | 11.97 | 29.50 | 11.16 | 29.52 | 12.45 | 11.77 | 29.55 | 12.11 | 12.54 | 17.16 |
| 1pzl | 2.18 | 2.53 | 3.56 | 3.89 | 35.91 | 9.87 | 3.99 | 33.97 | 9.79 | 6.86 | 33.46 | 33.16 | 32.11 | 8.14 | 30.30 | 33.09 | 31.80 | 38.16 | 33.45 | 38.19 |
| 1qkz | 9.02 | 10.08 | 6.69 | 10.22 | 9.18 | 9.25 | 8.30 | 10.03 | 6.67 | 10.11 | 9.16 | 11.92 | 11.82 | 9.57 | 10.25 | 10.09 | 11.76 | 10.99 | 11.62 | 12.07 |
| 1rst | 8.20 | 7.55 | 7.17 | 7.36 | 36.47 | 36.45 | 36.76 | 8.26 | 8.28 | 35.59 | 35.66 | 35.60 | 4.78 | 2.65 | 7.58 | 35.09 | 3.35 | 10.17 | 2.64 | 7.37 |
| 1rxz | 1.25 | 5.31 | 16.56 | 4.66 | 6.07 | 18.04 | 16.61 | 17.20 | 6.24 | 6.56 | 16.04 | 6.28 | 7.93 | 10.37 | 9.07 | 24.28 | 10.38 | 10.14 | 16.45 | 8.91 |
| 1sfi | 1.76 | 21.00 | 18.06 | 18.22 | 15.29 | 13.13 | 12.56 | 33.50 | 13.16 | 21.16 | 25.78 | 12.64 | 14.70 | 25.81 | 7.40 | 33.86 | 14.53 | 19.14 | 24.67 | 37.56 |
| 1ssh | 1.23 | 1.36 | 1.10 | 1.61 | 1.12 | 1.11 | 1.90 | 1.39 | 3.09 | 5.92 | 5.60 | 3.87 | 5.06 | 20.35 | 5.21 | 6.51 | 4.08 | 6.31 | 4.87 | 6.65 |
| 1t08 | 36.23 | 27.74 | 27.83 | 23.54 | 23.55 | 37.24 | 34.49 | 48.02 | 37.17 | 57.51 | 27.93 | 47.19 | 48.00 | 57.40 | 34.15 | 35.80 | 37.75 | 43.01 | 57.26 | 41.76 |
| 1t4f | 1.01 | 1.66 | 1.03 | 1.24 | 1.64 | 0.79 | 8.10 | 1.92 | 2.65 | 8.24 | 4.99 | 5.78 | 11.81 | 3.19 | 5.16 | 3.82 | 4.25 | 4.14 | 9.41 | 6.80 |
| 1t7r | 1.75 | 1.64 | 2.50 | 3.04 | 29.20 | 29.20 | 29.26 | 10.05 | 26.97 | 6.77 | 33.77 | 32.28 | 21.24 | 40.96 | 38.92 | 3.77 | 38.98 | 5.77 | 40.80 | 33.21 |
| 1tfc | 32.73 | 34.77 | 33.42 | 32.20 | 32.26 | 32.45 | 36.33 | 32.35 | 32.02 | 32.00 | 34.59 | 34.60 | 32.08 | 35.94 | 31.85 | 35.81 | 34.18 | 4.24 | 2.31 | 4.21 |
| 1u00 | 18.93 | 16.01 | 25.07 | 19.87 | 25.20 | 16.97 | 20.69 | 17.14 | 25.31 | 29.18 | 14.66 | 25.05 | 29.38 | 29.07 | 29.25 | 29.02 | 29.14 | 28.93 | 28.99 | 28.22 |
| 1uj0 | 1.33 | 1.14 | 1.11 | 7.42 | 7.36 | 7.51 | 1.55 | 7.77 | 13.66 | 14.72 | 18.23 | 18.35 | 14.56 | 14.36 | 2.93 | 8.86 | 3.49 | 22.37 | 3.98 | 1.65 |
| 1x2r | 7.36 | 7.33 | 4.47 | 7.32 | 3.58 | 7.40 | 32.16 | 1.59 | 3.00 | 31.53 | 6.46 | 8.59 | 6.61 | 6.10 | 6.51 | 37.72 | 7.58 | 32.98 | 31.99 | 7.58 |
| 1xoc | 25.10 | 26.00 | 22.82 | 34.15 | 19.54 | 34.23 | 25.16 | 25.04 | 25.13 | 24.79 | 25.66 | 25.60 | 24.80 | 36.92 | 20.45 | 25.79 | 25.71 | 28.61 | 40.36 | 28.13 |
| 1ymt | 3.53 | 6.85 | 6.36 | 7.16 | 1.98 | 6.23 | 1.51 | 1.37 | 1.98 | 1.41 | 31.76 | 1.28 | 6.11 | 6.85 | 6.67 | 4.70 | 5.26 | 38.56 | 30.08 | 30.03 |
| 1yuc | 20.09 | 27.70 | 27.82 | 35.56 | 26.04 | 27.97 | 37.00 | 33.02 | 26.33 | 1.49 | 1.13 | 15.30 | 0.89 | 1.32 | 20.49 | 27.04 | 27.93 | 1.48 | 33.34 | 34.05 |
| 1ywo | 17.10 | 16.47 | 16.47 | 17.25 | 16.46 | 17.24 | 16.47 | 17.11 | 1.65 | 16.58 | 17.40 | 17.05 | 17.55 | 17.52 | 12.17 | 17.19 | 1.88 | 12.17 | 12.08 | 12.10 |
| 2a25 | 24.93 | 17.18 | 35.18 | 31.13 | 31.31 | 26.39 | 31.08 | 25.24 | 26.03 | 25.08 | 2.07 | 23.62 | 34.21 | 24.57 | 27.94 | 25.04 | 9.42 | 31.05 | 24.44 | 16.33 |
| 2a3i | 1.71 | 1.46 | 1.36 | 1.37 | 2.66 | 2.58 | 2.25 | 2.90 | 4.68 | 38.57 | 38.52 | 38.52 | 4.88 | 38.09 | 4.83 | 20.95 | 38.19 | 4.96 | 10.12 | 6.23 |
| 2aq9 | 31.49 | 31.58 | 31.63 | 31.65 | 31.46 | 30.94 | 31.75 | 31.78 | 31.34 | 31.03 | 29.14 | 31.66 | 30.94 | 30.78 | 32.07 | 31.17 | 31.30 | 31.28 | 32.11 | 32.10 |
| 2b9h | 1.68 | 1.74 | 1.54 | 1.35 | 44.63 | 40.12 | 37.56 | 44.58 | 49.95 | 33.92 | 52.01 | 43.24 | 52.22 | 49.77 | 19.41 | 19.25 | 19.18 | 25.07 | 18.90 | 19.07 |
| 2bba | 1.86 | 29.13 | 2.00 | 35.57 | 28.66 | 29.02 | 28.81 | 29.28 | 33.30 | 35.26 | 30.02 | 37.35 | 30.25 | 24.14 | 22.04 | 37.29 | 27.70 | 30.29 | 30.09 | 31.45 |
| 2cch | 1.26 | 25.33 | 1.58 | 25.28 | 6.75 | 19.26 | 32.01 | 32.19 | 39.49 | 6.83 | 41.79 | 19.22 | 44.58 | 37.05 | 44.31 | 41.65 | 36.15 | 2.28 | 36.14 | 33.33 |
| 2ce8 | 8.06 | 8.09 | 8.02 | 38.71 | 9.36 | 8.33 | 38.55 | 38.33 | 8.56 | 8.41 | 39.16 | 33.96 | 29.14 | 29.28 | 39.43 | 32.84 | 41.38 | 8.68 | 8.84 | 41.44 |
| 2d0n | 1.56 | 1.62 | 1.44 | 1.50 | 1.76 | 1.56 | 2.65 | 4.46 | 3.03 | 4.67 | 3.75 | 4.21 | 3.84 | 4.19 | 3.37 | 4.48 | 2.01 | 3.13 | 3.33 | 4.95 |
| 2drk | 17.59 | 17.63 | 1.75 | 17.57 | 3.55 | 17.59 | 1.59 | 17.62 | 1.30 | 17.58 | 17.55 | 17.59 | 1.43 | 1.32 | 17.59 | 1.49 | 17.62 | 2.46 | 17.54 | 17.79 |
| 2dyp | 24.63 | 58.23 | 16.71 | 58.36 | 58.04 | 58.15 | 24.95 | 58.23 | 58.30 | 24.90 | 15.44 | 16.14 | 21.01 | 21.33 | 23.74 | 5.08 | 41.15 | 47.69 | 33.74 | 58.73 |
| 2fff | 41.18 | 1.37 | 27.48 | 28.13 | 21.76 | 11.99 | 21.85 | 28.13 | 21.14 | 21.34 | 27.69 | 27.54 | 19.34 | 26.11 | 28.03 | 40.02 | 43.25 | 42.83 | 22.29 | 21.62 |
| 2ffu | 25.93 | 33.18 | 51.44 | 36.96 | 29.57 | 52.01 | 51.52 | 37.02 | 37.28 | 32.86 | 32.98 | 46.84 | 46.76 | 29.32 | 44.57 | 33.63 | 48.16 | 33.41 | 31.70 | 52.55 |
| 2fka | 4.32 | 4.28 | 4.33 | 3.35 | 3.67 | 3.36 | 4.61 | 7.01 | 4.69 | 9.30 | 8.47 | 8.52 | 8.50 | 8.53 | 6.15 | 5.95 | 3.82 | 8.36 | 6.23 | 8.50 |
| 2fmf | 11.65 | 6.48 | 6.62 | 6.86 | 6.65 | 6.49 | 6.68 | 6.46 | 6.60 | 6.64 | 6.48 | 6.36 | 6.38 | 6.37 | 11.24 | 11.29 | 6.17 | 8.57 | 6.13 | 8.79 |
| 2fts | 0.80 | 0.79 | 0.84 | 5.21 | 5.04 | 63.11 | 60.99 | 19.19 | 18.70 | 37.52 | 18.82 | 45.89 | 18.90 | 18.73 | 61.69 | 53.38 | 47.35 | 53.26 | 19.82 | 16.82 |
| 2fvj | 32.40 | 32.45 | 33.80 | 24.98 | 33.85 | 32.79 | 33.72 | 32.73 | 26.74 | 33.28 | 36.68 | 36.76 | 1.42 | 36.83 | 36.84 | 29.94 | 28.31 | 43.59 | 21.77 | 28.21 |
| 2ho2 | 17.35 | 17.47 | 17.50 | 6.52 | 8.94 | 17.62 | 16.93 | 7.77 | 3.53 | 7.09 | 7.54 | 4.19 | 17.82 | 12.33 | 12.28 | 7.82 | 3.56 | 2.23 | 12.23 | 9.41 |
| 2ht9 | 23.44 | 23.33 | 23.24 | 21.47 | 23.96 | 23.76 | 21.32 | 20.57 | 22.36 | 22.36 | 22.51 | 22.36 | 19.25 | 22.27 | 33.31 | 21.27 | 22.42 | 25.27 | 27.40 | 27.34 |
| 2o02 | 6.28 | 33.20 | 33.04 | 34.29 | 21.39 | 21.30 | 32.54 | 32.56 | 25.72 | 32.57 | 32.60 | 32.64 | 32.72 | 32.94 | 31.87 | 33.63 | 33.36 | 33.49 | 32.14 | 33.79 |
| 2o4j | 1.96 | 2.44 | 2.22 | 34.99 | 35.01 | 34.90 | 42.00 | 41.97 | 42.26 | 6.94 | 35.24 | 42.21 | 6.53 | 43.10 | 9.41 | 42.91 | 43.03 | 7.07 | 30.76 | 7.07 |
| 2o9v | 2.40 | 2.52 | 2.62 | 2.66 | 1.91 | 1.81 | 2.08 | 1.27 | 16.79 | 1.06 | 16.76 | 16.75 | 16.98 | 17.00 | 16.95 | 1.03 | 16.95 | 17.00 | 17.16 | 1.23 |
| 2oei | 17.51 | 17.58 | 17.46 | 17.44 | 17.57 | 17.92 | 18.15 | 17.77 | 1.60 | 1.71 | 1.02 | 16.72 | 1.83 | 1.63 | 10.22 | 10.19 | 19.03 | 18.90 | 18.74 | 18.79 |
| 2p0w | 18.26 | 31.58 | 24.87 | 21.24 | 17.33 | 26.63 | 25.77 | 25.42 | 24.66 | 36.11 | 20.58 | 26.20 | 25.34 | 1.41 | 14.17 | 22.70 | 28.30 | 30.24 | 26.37 | 13.72 |
| 2p1o | 34.31 | 32.99 | 12.42 | 33.58 | 32.51 | 53.12 | 37.91 | 37.74 | 22.70 | 22.53 | 22.55 | 38.71 | 2.74 | 29.57 | 1.62 | 10.67 | 35.35 | 35.79 | 34.42 | 38.95 |
| 2p1t | 33.09 | 33.56 | 33.19 | 36.07 | 34.33 | 35.46 | 36.07 | 36.14 | 35.43 | 31.84 | 36.01 | 31.65 | 32.08 | 35.46 | 36.14 | 34.25 | 4.25 | 37.64 | 35.53 | 33.71 |
| 2p54 | 26.36 | 26.41 | 26.90 | 23.97 | 25.94 | 44.53 | 27.17 | 27.48 | 25.69 | 25.84 | 26.77 | 26.63 | 31.05 | 26.89 | 26.86 | 32.29 | 27.90 | 31.98 | 42.94 | 27.79 |
| 2peh | 2.87 | 2.49 | 28.51 | 28.45 | 29.56 | 27.94 | 9.02 | 27.66 | 9.46 | 11.50 | 8.80 | 10.13 | 11.53 | 11.46 | 13.20 | 9.72 | 9.68 | 11.56 | 27.35 | 9.43 |
| 2pux | 1.46 | 1.57 | 1.94 | 3.81 | 1.91 | 3.72 | 2.22 | 4.26 | 7.91 | 43.77 | 38.42 | 8.87 | 38.35 | 37.80 | 35.45 | 40.87 | 10.29 | 40.42 | 10.85 | 40.96 |
| 2puy | 1.20 | 24.17 | 23.97 | 34.27 | 5.76 | 6.16 | 34.40 | 5.53 | 6.64 | 34.29 | 5.08 | 25.95 | 25.12 | 23.80 | 24.02 | 6.42 | 26.25 | 6.92 | 8.62 | 23.65 |
| 2qbx | 27.31 | 11.16 | 11.14 | 25.13 | 27.25 | 41.11 | 25.09 | 41.11 | 41.39 | 27.87 | 27.44 | 41.21 | 32.22 | 41.29 | 27.18 | 21.67 | 22.57 | 25.59 | 10.57 | 25.08 |
| 2qos | 22.14 | 22.11 | 2.26 | 24.49 | 22.17 | 28.68 | 22.68 | 23.49 | 26.62 | 28.71 | 16.89 | 28.77 | 25.09 | 25.73 | 17.04 | 13.58 | 26.10 | 26.58 | 25.02 | 22.72 |
| 2qse | 1.53 | 1.64 | 1.69 | 1.41 | 1.82 | 1.69 | 1.86 | 32.95 | 6.06 | 6.09 | 6.28 | 40.59 | 6.40 | 40.56 | 6.46 | 3.32 | 40.33 | 34.18 | 6.52 | 40.48 |
| 2r7g | 36.81 | 20.31 | 20.15 | 36.95 | 36.83 | 37.17 | 36.64 | 37.43 | 19.69 | 21.29 | 20.57 | 43.86 | 20.84 | 21.12 | 36.44 | 19.71 | 48.70 | 37.99 | 37.73 | 45.53 |
| 2r9q | 9.08 | 1.86 | 1.65 | 6.09 | 6.15 | 6.10 | 10.14 | 6.55 | 5.99 | 6.28 | 7.74 | 8.18 | 5.84 | 7.34 | 33.78 | 8.30 | 10.44 | 7.05 | 5.69 | 5.80 |
| 2v8y | 0.77 | 0.88 | 1.18 | 18.94 | 18.91 | 34.70 | 18.68 | 19.59 | 36.49 | 2.66 | 14.99 | 38.96 | 38.25 | 38.92 | 36.62 | 35.53 | 28.25 | 36.53 | 36.51 | 38.22 |
| 2vkn | 0.85 | 1.08 | 0.72 | 1.36 | 1.59 | 0.65 | 2.49 | 17.11 | 15.55 | 1.55 | 6.28 | 15.56 | 6.19 | 2.50 | 10.25 | 5.41 | 15.53 | 15.46 | 6.32 | 19.35 |
| 2vr3 | 23.80 | 25.53 | 38.24 | 13.78 | 33.64 | 26.71 | 16.24 | 37.25 | 13.53 | 32.94 | 28.85 | 20.48 | 20.47 | 24.33 | 37.46 | 35.11 | 20.76 | 36.52 | 37.77 | 36.70 |
| 2vwf | 22.19 | 22.17 | 0.63 | 0.62 | 6.84 | 0.67 | 7.30 | 1.58 | 30.83 | 26.12 | 26.08 | 26.12 | 30.73 | 3.28 | 5.44 | 15.09 | 12.13 | 12.32 | 11.86 | 3.70 |
| 2w2u | 26.38 | 24.01 | 23.98 | 24.28 | 24.08 | 24.13 | 25.02 | 25.03 | 25.10 | 25.37 | 25.26 | 23.87 | 11.19 | 21.72 | 21.82 | 23.86 | 25.70 | 24.32 | 23.86 | 1.27 |
| 2whx | 87.31 | 28.04 | 24.57 | 28.07 | 24.59 | 34.33 | 22.84 | 15.50 | 77.40 | 28.78 | 15.39 | 15.48 | 15.56 | 15.38 | 15.57 | 78.82 | 78.52 | 29.53 | 43.50 | 26.89 |
| 2xrw | 2.00 | 24.52 | 6.08 | 31.75 | 46.36 | 23.58 | 25.17 | 23.78 | 25.02 | 28.71 | 24.96 | 25.01 | 16.27 | 20.61 | 26.87 | 37.21 | 25.31 | 26.08 | 33.05 | 22.50 |
| 2xu7 | 1.21 | 0.95 | 1.03 | 4.28 | 0.99 | 1.30 | 2.92 | 2.49 | 14.09 | 4.14 | 3.84 | 3.39 | 2.01 | 4.51 | 5.45 | 43.67 | 43.15 | 42.19 | 43.30 | 41.00 |
| 2xvc | 25.82 | 22.93 | 22.72 | 26.58 | 27.84 | 26.74 | 26.79 | 26.71 | 33.26 | 33.22 | 33.26 | 33.04 | 26.59 | 28.02 | 23.54 | 27.92 | 32.84 | 25.95 | 28.08 | 26.89 |
| 2zjd | 0.84 | 1.33 | 1.71 | 30.78 | 30.78 | 30.21 | 30.22 | 28.91 | 8.15 | 29.36 | 7.70 | 18.35 | 7.99 | 18.63 | 33.12 | 28.77 | 8.23 | 19.30 | 19.19 | 8.22 |
| 3asl | 27.18 | 25.37 | 25.81 | 27.84 | 25.17 | 25.29 | 26.83 | 24.95 | 25.01 | 25.71 | 25.17 | 27.09 | 28.08 | 25.08 | 25.01 | 34.16 | 34.14 | 28.25 | 25.33 | 28.14 |
| 3awr | 20.70 | 11.44 | 12.47 | 12.49 | 22.97 | 23.14 | 23.50 | 22.93 | 22.89 | 19.32 | 23.00 | 24.17 | 11.09 | 19.64 | 19.53 | 24.28 | 23.14 | 23.86 | 19.93 | 23.08 |
| 3ayu | 0.73 | 1.08 | 17.69 | 4.76 | 24.25 | 15.82 | 17.26 | 25.07 | 16.07 | 8.56 | 2.45 | 20.68 | 18.87 | 31.88 | 32.03 | 31.76 | 31.74 | 31.52 | 31.76 | 31.69 |
| 3bfq | 0.36 | 23.94 | 33.46 | 33.43 | 33.84 | 33.82 | 33.97 | 29.51 | 33.13 | 33.26 | 33.05 | 11.75 | 13.46 | 31.52 | 33.94 | 31.52 | 23.70 | 32.63 | 12.85 | 23.85 |
| 3c3r | 12.09 | 2.55 | 12.06 | 12.06 | 39.50 | 2.38 | 39.71 | 5.90 | 5.93 | 36.74 | 6.04 | 5.92 | 5.71 | 40.60 | 5.71 | 40.70 | 5.57 | 40.57 | 5.61 | 40.53 |
| 3d32 | 0.64 | 18.52 | 19.52 | 19.50 | 19.33 | 11.28 | 11.19 | 15.67 | 18.16 | 22.02 | 15.77 | 11.25 | 8.33 | 18.00 | 24.95 | 15.64 | 15.50 | 14.68 | 11.88 | 16.48 |
| 3ds4 | 23.74 | 23.76 | 4.96 | 24.17 | 4.89 | 22.95 | 23.80 | 23.60 | 9.34 | 23.83 | 9.31 | 24.35 | 22.90 | 10.41 | 22.78 | 22.88 | 22.81 | 23.35 | 23.37 | 22.95 |
| 3ery | 1.00 | 2.55 | 5.93 | 5.45 | 7.78 | 7.75 | 8.77 | 7.28 | 9.97 | 8.07 | 6.61 | 15.39 | 16.45 | 4.34 | 12.71 | 10.60 | 12.20 | 5.40 | 10.48 | 12.52 |
| 3fdo | 1.79 | 2.14 | 17.14 | 2.48 | 11.86 | 11.55 | 25.97 | 25.87 | 17.76 | 25.91 | 25.79 | 25.99 | 17.99 | 19.43 | 8.19 | 24.57 | 15.97 | 11.64 | 17.96 | 11.57 |
| 3g2s | 13.78 | 2.61 | 2.35 | 30.30 | 30.31 | 30.34 | 2.00 | 30.23 | 30.63 | 23.86 | 12.92 | 30.42 | 30.24 | 30.24 | 32.81 | 12.78 | 12.37 | 17.91 | 12.29 | 30.52 |
| 3gyt | 1.96 | 38.36 | 38.40 | 37.95 | 38.62 | 37.71 | 41.38 | 41.15 | 43.38 | 38.26 | 41.02 | 41.18 | 39.52 | 45.94 | 41.29 | 39.50 | 41.33 | 39.46 | 41.52 | 39.42 |
| 3h1z | 1.47 | 10.16 | 9.55 | 21.21 | 21.21 | 31.30 | 26.66 | 21.15 | 21.23 | 25.37 | 24.18 | 26.44 | 19.60 | 4.58 | 30.15 | 37.81 | 57.82 | 37.76 | 31.40 | 31.10 |
| 3i5r | 1.23 | 1.11 | 1.33 | 1.07 | 1.47 | 15.58 | 15.49 | 15.56 | 15.62 | 15.53 | 10.46 | 10.24 | 10.13 | 15.46 | 10.28 | 15.50 | 11.55 | 16.66 | 11.21 | 6.33 |
| 3ivv | 17.22 | 11.54 | 32.80 | 32.70 | 35.15 | 35.08 | 35.07 | 28.74 | 26.65 | 18.91 | 26.01 | 19.06 | 20.35 | 35.09 | 18.50 | 18.08 | 32.03 | 29.31 | 27.09 | 27.11 |
| 3kmr | 32.80 | 34.79 | 34.91 | 34.99 | 2.04 | 35.49 | 1.13 | 35.41 | 35.88 | 35.53 | 35.34 | 34.47 | 35.45 | 34.37 | 35.57 | 34.75 | 34.01 | 33.92 | 33.90 | 33.93 |
| 3kuj | 1.03 | 19.46 | 20.99 | 19.69 | 15.11 | 17.80 | 18.73 | 17.86 | 21.80 | 15.28 | 20.96 | 20.85 | 25.27 | 21.85 | 21.10 | 21.16 | 24.37 | 21.95 | 25.85 | 21.91 |
| 3kus | 18.01 | 15.95 | 18.42 | 24.28 | 24.85 | 25.09 | 16.64 | 18.19 | 26.66 | 26.23 | 27.05 | 26.57 | 26.46 | 25.31 | 23.37 | 23.46 | 23.36 | 27.18 | 26.79 | 17.81 |
| 3l0e | 28.76 | 28.67 | 29.30 | 28.12 | 32.11 | 28.38 | 28.38 | 29.41 | 39.25 | 32.57 | 39.18 | 28.91 | 26.63 | 32.00 | 25.86 | 26.44 | 26.43 | 29.32 | 26.77 | 1.27 |
| 3ll8 | 41.91 | 0.84 | 3.38 | 1.66 | 24.87 | 24.84 | 24.87 | 24.99 | 27.94 | 12.81 | 25.04 | 12.36 | 22.10 | 10.27 | 22.21 | 20.19 | 20.42 | 48.65 | 22.26 | 22.35 |
| 3llz | 36.69 | 29.75 | 36.84 | 2.65 | 2.50 | 36.93 | 25.87 | 26.06 | 26.16 | 24.07 | 22.77 | 25.67 | 22.59 | 25.85 | 30.57 | 24.00 | 23.96 | 30.47 | 30.41 | 25.93 |
| 3obq | 17.80 | 17.87 | 17.65 | 25.58 | 23.55 | 7.64 | 26.30 | 7.60 | 7.63 | 7.80 | 26.77 | 32.77 | 7.47 | 32.84 | 26.63 | 27.44 | 26.00 | 32.84 | 42.51 | 28.15 |
| 3olf | 1.52 | 1.73 | 1.48 | 1.31 | 1.17 | 2.13 | 37.37 | 37.29 | 37.36 | 37.91 | 27.11 | 27.23 | 37.70 | 37.84 | 26.95 | 37.16 | 37.88 | 37.63 | 32.01 | 39.66 |
| 3p72 | 5.55 | 35.30 | 35.31 | 21.45 | 35.31 | 35.45 | 35.19 | 35.43 | 41.33 | 6.94 | 41.33 | 41.28 | 35.17 | 41.18 | 21.82 | 39.53 | 23.85 | 23.72 | 23.45 | 39.55 |
| 3p8f | 1.13 | 5.63 | 14.44 | 14.29 | 26.59 | 16.54 | 26.59 | 26.59 | 15.75 | 16.48 | 16.39 | 22.64 | 22.51 | 22.12 | 15.57 | 15.62 | 6.15 | 12.46 | 22.46 | 12.14 |
| 3ptl | 33.75 | 17.15 | 28.19 | 20.19 | 19.99 | 29.52 | 29.58 | 28.55 | 13.77 | 13.81 | 19.03 | 19.05 | 13.76 | 18.70 | 29.41 | 16.68 | 19.95 | 27.01 | 16.29 | 16.34 |
| 3qis | 0.90 | 0.81 | 0.77 | 0.69 | 13.58 | 30.68 | 30.57 | 41.47 | 34.57 | 41.44 | 2.59 | 34.13 | 2.02 | 12.81 | 31.01 | 2.77 | 34.61 | 31.70 | 31.47 | 28.71 |
| 3rm1 | 1.42 | 1.63 | 20.03 | 27.33 | 20.10 | 0.70 | 27.21 | 27.38 | 27.32 | 27.36 | 18.46 | 20.16 | 27.41 | 1.00 | 2.75 | 8.79 | 2.99 | 27.39 | 7.55 | 9.44 |
| 3rqg | 40.98 | 41.05 | 41.13 | 40.80 | 41.33 | 41.29 | 41.31 | 40.56 | 37.50 | 51.55 | 31.49 | 42.02 | 37.01 | 42.14 | 2.72 | 43.00 | 42.42 | 42.59 | 35.38 | 3.36 |
| 3sfj | 0.57 | 0.47 | 19.22 | 24.76 | 21.60 | 19.48 | 25.90 | 25.89 | 25.97 | 26.38 | 26.62 | 17.97 | 19.48 | 18.37 | 26.70 | 17.47 | 26.05 | 16.94 | 23.79 | 25.78 |
| 3so6 | 0.89 | 4.72 | 4.88 | 7.37 | 4.89 | 27.03 | 26.86 | 26.88 | 26.91 | 21.30 | 17.82 | 19.11 | 6.03 | 14.28 | 8.25 | 29.11 | 24.14 | 6.30 | 23.12 | 24.04 |
| 3tjv | 8.40 | 9.31 | 9.51 | 11.97 | 11.89 | 13.44 | 13.42 | 10.49 | 32.10 | 29.92 | 17.44 | 16.78 | 12.84 | 11.06 | 31.59 | 29.67 | 29.64 | 10.53 | 11.95 | 29.70 |
| 3tzy | 35.87 | 35.75 | 35.80 | 39.95 | 40.11 | 39.70 | 39.55 | 40.03 | 39.87 | 40.20 | 37.67 | 35.04 | 40.20 | 39.37 | 35.01 | 31.95 | 31.83 | 40.09 | 20.79 | 35.99 |
| 3u9q | 2.24 | 30.84 | 31.44 | 2.08 | 30.53 | 31.63 | 31.08 | 1.80 | 1.41 | 31.98 | 31.79 | 31.94 | 32.20 | 30.51 | 41.16 | 30.75 | 30.65 | 30.56 | 29.34 | 30.69 |
| 3up3 | 32.22 | 32.15 | 32.35 | 1.16 | 1.21 | 31.26 | 46.11 | 24.68 | 46.12 | 31.15 | 25.90 | 46.17 | 30.10 | 5.41 | 5.62 | 5.73 | 46.03 | 28.45 | 5.61 | 32.29 |
| 3v2x | 7.00 | 6.86 | 1.29 | 1.32 | 1.53 | 6.30 | 6.49 | 6.80 | 18.02 | 18.09 | 18.05 | 24.19 | 19.86 | 5.45 | 20.67 | 19.84 | 5.90 | 17.68 | 4.54 | 19.88 |
| 3vtc | 1.30 | 1.00 | 0.98 | 31.60 | 32.84 | 41.47 | 31.44 | 41.39 | 5.76 | 33.01 | 31.51 | 31.20 | 40.22 | 10.86 | 10.91 | 32.73 | 32.94 | 40.65 | 9.00 | 31.02 |
| 3w1b | 83.31 | 83.19 | 83.42 | 83.67 | 83.54 | 23.29 | 42.35 | 86.95 | 42.62 | 21.53 | 52.63 | 22.74 | 23.10 | 27.21 | 39.57 | 52.99 | 27.28 | 83.93 | 27.03 | 46.41 |
| 3zqh | 35.95 | 38.23 | 29.42 | 38.01 | 37.04 | 36.81 | 41.36 | 41.29 | 41.08 | 40.97 | 40.09 | 40.08 | 17.64 | 18.19 | 37.37 | 29.45 | 40.01 | 15.94 | 13.18 | 15.83 |
| 4b4n | 29.46 | 28.63 | 30.86 | 26.29 | 31.15 | 27.51 | 22.32 | 26.47 | 28.03 | 22.23 | 29.94 | 27.86 | 27.79 | 27.89 | 29.81 | 25.56 | 26.00 | 25.76 | 28.73 | 25.30 |
| 4dcb | 14.72 | 44.10 | 45.29 | 43.65 | 43.89 | 45.48 | 51.57 | 51.56 | 11.96 | 11.99 | 12.30 | 52.11 | 9.55 | 51.65 | 9.51 | 47.39 | 52.34 | 51.32 | 53.29 | 14.41 |
| 4e34 | 1.33 | 14.72 | 21.05 | 14.59 | 28.30 | 14.50 | 14.59 | 21.42 | 21.09 | 21.02 | 14.45 | 28.22 | 28.33 | 21.06 | 10.05 | 22.28 | 22.12 | 9.15 | 22.34 | 9.79 |
| 4eik | 0.67 | 0.66 | 0.95 | 1.13 | 0.71 | 23.00 | 23.05 | 24.97 | 24.93 | 24.94 | 1.83 | 11.89 | 11.78 | 10.52 | 26.23 | 26.35 | 26.43 | 27.02 | 26.95 | 26.97 |
| 4ery | 1.44 | 1.60 | 12.71 | 12.22 | 12.58 | 12.60 | 11.94 | 13.03 | 13.12 | 12.09 | 11.60 | 11.88 | 11.37 | 14.49 | 12.06 | 7.70 | 11.94 | 7.61 | 15.33 | 14.53 |
| 4f14 | 1.88 | 1.85 | 1.81 | 2.27 | 19.22 | 8.40 | 10.84 | 19.13 | 7.75 | 19.00 | 19.05 | 11.43 | 18.77 | 7.71 | 11.09 | 18.37 | 7.84 | 10.69 | 11.61 | 21.41 |
| 4f1z | 30.87 | 28.76 | 15.26 | 30.24 | 24.10 | 24.08 | 32.11 | 14.92 | 33.80 | 31.09 | 32.13 | 34.94 | 32.37 | 24.16 | 24.03 | 25.39 | 49.90 | 31.55 | 28.81 | 49.75 |
| 4gq6 | 9.27 | 10.05 | 10.08 | 9.58 | 33.17 | 33.26 | 32.71 | 33.31 | 9.77 | 10.30 | 32.90 | 34.73 | 10.35 | 12.03 | 39.61 | 13.42 | 31.90 | 30.95 | 25.81 | 34.70 |
| 4gxl | 11.10 | 9.98 | 10.04 | 11.31 | 8.88 | 8.77 | 9.31 | 8.91 | 23.74 | 29.58 | 10.20 | 10.31 | 27.75 | 12.58 | 12.11 | 32.64 | 27.66 | 10.72 | 12.07 | 14.49 |
| 4gyw | 15.33 | 15.92 | 39.49 | 25.76 | 26.04 | 45.46 | 38.96 | 27.91 | 47.85 | 38.15 | 38.07 | 42.04 | 47.71 | 40.62 | 41.03 | 24.90 | 49.55 | 38.70 | 25.67 | 38.59 |
| 4h4f | 1.52 | 14.44 | 1.04 | 1.31 | 14.52 | 14.83 | 15.78 | 2.14 | 14.91 | 34.41 | 15.15 | 14.92 | 15.09 | 34.46 | 9.82 | 27.84 | 36.41 | 33.77 | 9.50 | 10.21 |
| 4hom | 32.20 | 36.79 | 32.43 | 33.15 | 37.00 | 24.45 | 33.33 | 36.16 | 40.93 | 33.73 | 40.66 | 34.37 | 38.17 | 37.30 | 31.25 | 35.53 | 37.45 | 37.11 | 36.05 | 36.86 |
| 4htp | 38.72 | 38.37 | 38.83 | 44.65 | 34.99 | 35.13 | 38.92 | 33.29 | 33.56 | 34.86 | 38.87 | 34.43 | 38.83 | 26.83 | 26.49 | 27.87 | 46.31 | 30.36 | 25.34 | 34.85 |
| 4iim | 5.20 | 5.17 | 3.31 | 17.24 | 8.42 | 8.56 | 8.76 | 17.25 | 22.96 | 17.12 | 23.11 | 11.12 | 25.91 | 23.27 | 23.22 | 10.77 | 23.24 | 10.94 | 11.14 | 0.82 |
| 4j8s | 29.49 | 26.53 | 26.63 | 26.54 | 34.93 | 30.01 | 28.14 | 26.78 | 44.04 | 44.02 | 30.72 | 29.98 | 26.79 | 26.66 | 31.36 | 26.92 | 44.04 | 26.74 | 26.85 | 31.30 |
| 4k0u | 1.61 | 1.77 | 1.49 | 1.50 | 1.88 | 11.45 | 6.45 | 3.39 | 2.32 | 11.31 | 10.88 | 2.57 | 10.97 | 11.11 | 11.01 | 11.14 | 9.02 | 8.76 | 13.62 | 2.79 |

**S4(b). L-RMSD values of all 20 poses obtained after blind docking by Hex on 133 protein-peptide complexes.**

| **ID** | **Pose1** | **Pose2** | **Pose3** | **Pose4** | **Pose5** | **Pose6** | **Pose7** | **Pose8** | **Pose9** | **Pose10** | **Pose11** | **Pose12** | **Pose13** | **Pose14** | **Pose15** | **Pose16** | **Pose17** | **Pose18** | **Pose19** | **Pose20** |
| --- | --- | --- | --- | --- | --- | --- | --- | --- | --- | --- | --- | --- | --- | --- | --- | --- | --- | --- | --- | --- |
| 1cjr | 13.05 | 22.65 | 6.95 | 9.56 | 1.86 | 8.73 | 12.35 | 10.75 | 13.40 | 12.93 | 11.13 | 4.62 | 12.48 | 10.13 | 22.70 | 4.66 | 19.67 | 11.08 | 10.78 | 18.86 |
| 1cka | 182.04 | NA | NA | NA | NA | NA | NA | NA | NA | NA | NA | NA | NA | NA | NA | NA | NA | NA | NA | NA |
| 1cvu | 56.68 | 44.12 | 47.71 | 46.94 | 56.68 | 46.52 | 55.96 | 45.63 | 54.76 | 55.55 | 46.70 | 48.57 | 54.68 | 45.56 | 49.31 | 48.32 | 43.28 | 45.68 | 59.70 | 55.67 |
| 1d4t | 17.51 | 3.66 | 18.15 | 21.08 | 15.25 | 27.17 | 25.26 | 15.98 | 10.04 | 24.80 | 19.75 | 26.33 | 20.01 | 10.05 | 19.69 | 7.05 | 26.43 | 8.73 | 18.00 | 16.79 |
| 1eg4 | 34.72 | 64.62 | 64.71 | 62.89 | 65.90 | 41.86 | 35.49 | 54.63 | 32.03 | 63.61 | 28.81 | 33.40 | 40.31 | 37.06 | 24.79 | 42.36 | 34.49 | 53.54 | 61.56 | 63.57 |
| 1h6w | 143.01 | 142.70 | 142.73 | 142.80 | 141.96 | 143.34 | 141.57 | 142.98 | 142.78 | 142.78 | 142.89 | 142.14 | 142.17 | 142.73 | 142.91 | 141.91 | 143.03 | 142.55 | 142.72 | 141.84 |
| 1hc9 | 45.42 | 42.50 | 28.10 | 48.96 | 41.66 | 46.21 | 46.70 | 48.98 | 49.16 | 29.25 | 25.62 | 40.22 | 45.18 | 26.81 | 27.30 | 40.50 | 44.25 | 37.99 | 42.09 | 48.43 |
| 1jbu | 15.66 | 40.88 | 34.25 | 23.73 | 30.72 | 26.85 | 40.63 | 42.69 | 20.31 | 37.41 | 37.90 | 32.77 | 17.10 | 38.42 | 11.58 | 22.66 | 22.64 | 30.95 | 30.63 | 20.77 |
| 1k5n | 16.15 | 13.14 | 26.53 | 18.83 | 15.11 | 46.57 | 22.90 | 16.58 | 17.60 | 18.39 | 11.44 | 29.12 | 17.60 | 16.81 | 14.88 | 13.74 | 30.02 | 45.59 | 46.93 | 26.85 |
| 1mfg | 24.06 | 21.96 | 27.68 | 26.45 | 30.15 | 29.02 | 18.94 | 27.18 | 21.07 | 13.88 | 22.63 | 16.23 | 27.72 | 24.70 | 28.63 | 15.23 | 26.94 | 28.08 | 30.76 | 22.36 |
| 1nln | 51.50 | 51.12 | 51.12 | 54.34 | 54.97 | 55.95 | 52.66 | 51.71 | 53.24 | 56.55 | 53.85 | 55.54 | 49.86 | 48.45 | 54.86 | 54.34 | 48.34 | 52.59 | 57.95 | 51.36 |
| 1nq7 | 20.58 | 39.93 | 36.14 | 37.76 | 5.59 | 38.30 | 15.70 | 33.31 | 34.18 | 35.92 | 37.10 | 38.42 | 11.17 | 33.50 | 37.64 | 30.10 | 8.04 | 30.76 | 34.17 | 5.80 |
| 1ntv | 28.66 | 18.07 | 14.04 | 10.19 | 15.67 | 20.63 | 27.43 | 16.17 | 19.41 | 16.64 | 23.24 | 20.86 | 14.16 | 25.22 | 19.32 | 29.35 | 28.23 | 27.85 | 27.04 | 26.99 |
| 1nx1 | 73.84 | 74.07 | 72.98 | 74.99 | 72.35 | 74.20 | 72.77 | 76.00 | 73.11 | 73.93 | 74.05 | 71.65 | 71.72 | 74.61 | 73.44 | 74.16 | 75.33 | 50.11 | 77.00 | 78.25 |
| 1oai | 22.25 | 27.43 | 17.68 | 18.15 | 20.40 | 28.44 | 19.82 | 18.95 | 25.43 | 27.96 | 17.69 | 23.47 | 27.68 | 18.89 | 20.85 | 23.25 | 24.29 | 25.17 | 27.03 | 28.60 |
| 1oj5 | 30.39 | 21.83 | 25.34 | 28.22 | 30.83 | 31.83 | 30.74 | 28.49 | 34.05 | 33.72 | 29.09 | 27.89 | 33.11 | 28.42 | 32.02 | 33.70 | 27.03 | 34.28 | 31.58 | 32.38 |
| 1ou8 | 95.25 | 94.17 | 95.18 | 94.50 | 94.19 | 94.81 | 94.55 | 94.85 | 94.47 | 94.10 | 94.81 | 94.97 | 93.67 | 95.33 | 94.13 | 95.64 | 94.76 | 94.23 | 95.53 | 93.44 |
| 1ow6 | 53.97 | 55.01 | 37.42 | 47.27 | 50.56 | 39.80 | 45.69 | 52.11 | 43.56 | 45.20 | 53.77 | 46.07 | 38.21 | 51.58 | 51.53 | 52.07 | 34.85 | 36.94 | 36.23 | 52.41 |
| 1pzl | 89.20 | 89.40 | 88.89 | 89.77 | 88.43 | 88.39 | 88.95 | 87.77 | 88.33 | 94.17 | 88.64 | 88.83 | 88.51 | 90.33 | 87.93 | 89.52 | 88.91 | 89.08 | 94.71 | 90.32 |
| 1qkz | 33.54 | 35.67 | 34.17 | 34.43 | 35.07 | 37.84 | 34.43 | 33.68 | 34.26 | 35.18 | 33.84 | 37.15 | 33.10 | 34.07 | 35.58 | 30.07 | 37.53 | 38.30 | 34.50 | 36.91 |
| 1rst | 59.13 | 58.99 | 57.12 | 59.00 | 61.71 | 60.64 | 60.77 | 60.80 | 61.03 | 59.39 | 61.88 | 58.29 | 58.88 | 60.76 | 61.76 | 58.32 | 60.43 | 60.52 | 57.73 | 58.08 |
| 1rxz | 84.54 | 83.91 | 85.36 | 85.83 | 83.67 | 84.40 | 83.63 | 84.36 | 84.86 | 84.33 | 83.19 | 85.21 | 84.62 | 86.28 | 84.66 | 85.36 | 85.38 | 82.71 | 84.54 | 82.62 |
| 1sfi | 26.24 | 26.45 | 40.52 | 37.44 | 40.57 | 38.44 | 25.84 | 41.27 | 39.27 | 26.54 | 23.26 | 26.14 | 39.99 | 31.11 | 28.49 | 38.37 | 39.66 | 26.31 | 37.32 | 40.15 |
| 1ssh | 23.29 | 21.97 | 22.53 | 31.47 | 23.41 | 27.62 | 24.61 | 17.28 | 16.18 | 32.30 | 31.47 | 21.87 | 20.83 | 19.19 | 16.19 | 28.36 | 24.67 | 21.77 | 24.74 | 25.65 |
| 1t08 | 41.83 | 40.35 | 46.12 | 40.55 | 43.27 | 40.82 | 38.57 | 39.78 | 43.33 | 36.85 | 46.59 | 42.72 | 40.47 | 39.31 | 39.20 | 42.24 | 40.13 | 32.40 | 39.56 | 44.70 |
| 1t4f | 8.93 | 1.66 | 8.06 | 6.55 | 8.25 | 8.72 | 6.25 | 9.50 | 5.65 | 7.59 | 9.50 | 8.15 | 4.08 | 11.80 | 6.30 | 7.53 | 9.16 | 5.12 | 10.04 | 8.54 |
| 1t7r | 37.56 | 43.22 | 36.31 | 41.33 | 38.94 | 45.46 | 43.65 | 37.18 | 37.28 | 45.72 | 40.12 | 47.30 | 45.73 | 42.71 | 39.54 | 44.53 | 46.42 | 43.79 | 38.52 | 47.29 |
| 1tfc | 13.84 | 37.53 | 27.18 | 25.60 | 29.13 | 24.71 | 21.76 | 47.16 | 26.07 | 31.63 | 26.80 | 39.58 | 38.37 | 32.09 | 38.79 | 29.92 | 35.77 | 35.92 | 39.21 | 26.45 |
| 1u00 | 27.36 | 29.97 | 26.30 | 27.47 | 26.89 | 23.85 | 25.98 | 25.87 | 24.92 | 29.78 | 24.85 | 23.68 | 29.21 | 22.46 | 24.37 | 22.70 | 24.29 | 28.37 | 23.32 | 29.41 |
| 1uj0 | 30.13 | 28.42 | 32.38 | 19.30 | 30.06 | 31.04 | 32.55 | 31.94 | 28.82 | 29.85 | 24.79 | 28.69 | 25.85 | 27.07 | 19.31 | 24.34 | 18.62 | 30.84 | 16.57 | 29.24 |
| 1x2r | 63.38 | 61.22 | 66.38 | 67.55 | 64.71 | 63.44 | 65.63 | 55.17 | 62.94 | 63.82 | 64.85 | 60.43 | 60.33 | 65.01 | 65.86 | 65.29 | 63.62 | 63.55 | 61.82 | 63.34 |
| 1xoc | 41.38 | 45.40 | 44.47 | 43.10 | 43.07 | 42.48 | 43.95 | 43.28 | 42.43 | 40.33 | 43.52 | 41.20 | 41.46 | 41.79 | 45.56 | 41.96 | 42.06 | 44.81 | 44.47 | 43.08 |
| 1ymt | 84.33 | 85.01 | 84.28 | 78.61 | 80.65 | 89.95 | 79.71 | 80.28 | 84.26 | 77.99 | 78.07 | 79.13 | 71.62 | 79.91 | 76.83 | 84.91 | 78.19 | 77.85 | 79.74 | 84.08 |
| 1yuc | 77.23 | 72.82 | 53.44 | 75.93 | 52.42 | 53.17 | 76.35 | 56.80 | 55.01 | 55.46 | 56.00 | 50.11 | 50.87 | 59.91 | 54.09 | 76.82 | 56.58 | 54.56 | 75.83 | 50.70 |
| 1ywo | 17.03 | 17.78 | 24.50 | 21.53 | 27.69 | 25.58 | 19.33 | 22.50 | 24.51 | 23.15 | 22.01 | 19.85 | 19.61 | 24.82 | 27.58 | 24.19 | 27.48 | 22.65 | 24.04 | 30.11 |
| 2a25 | 33.90 | 33.65 | 32.91 | 34.96 | 29.97 | 31.00 | 29.86 | 33.09 | 33.72 | 30.90 | 34.53 | 29.73 | 34.86 | 30.28 | 29.20 | 31.70 | 29.88 | 30.78 | 32.58 | 30.78 |
| 2a3i | 65.02 | 66.44 | 68.24 | 65.07 | 63.99 | 67.16 | 61.86 | 66.92 | 63.65 | 66.17 | 70.04 | 69.54 | 70.21 | 77.79 | 66.98 | 66.95 | 60.65 | 63.93 | 63.70 | 68.41 |
| 2aq9 | 95.65 | 95.88 | 91.15 | 96.63 | 95.77 | 90.86 | 90.54 | 96.66 | 97.00 | 95.93 | 96.59 | 96.53 | 95.50 | 90.53 | 96.81 | 90.76 | 96.59 | 96.42 | 91.90 | 91.65 |
| 2b9h | 39.72 | 22.76 | 19.03 | 8.29 | 15.73 | 47.20 | 21.55 | 34.24 | 22.74 | 50.34 | 48.16 | 31.66 | 48.26 | 48.57 | 22.27 | 44.37 | 17.07 | 30.29 | 21.22 | 34.65 |
| 2bba | 66.59 | 67.01 | 67.46 | 64.04 | 65.84 | 67.57 | 69.16 | 66.05 | 66.75 | 66.70 | 46.40 | 65.18 | 33.07 | 49.50 | 65.21 | 64.66 | 48.24 | 67.74 | 42.24 | 48.48 |
| 2cch | 35.09 | 31.68 | 37.55 | 34.40 | 31.54 | 29.66 | 15.89 | 19.85 | 4.16 | 36.20 | 29.01 | 29.92 | 36.52 | 18.44 | 40.20 | 29.75 | 42.45 | 9.66 | 31.66 | 33.94 |
| 2ce8 | 49.15 | 33.76 | 46.75 | 47.01 | 46.44 | 34.43 | 35.46 | 40.69 | 36.52 | 36.41 | 41.74 | 38.82 | 42.82 | 44.63 | 40.98 | 37.56 | 41.28 | 40.03 | 38.26 | 40.64 |
| 2d0n | 23.35 | 22.77 | 21.26 | 20.88 | 17.33 | 18.07 | 26.24 | 15.90 | 22.48 | 30.15 | 21.50 | 19.55 | 28.86 | 21.97 | 31.72 | 17.13 | 17.96 | 19.82 | 29.17 | 21.28 |
| 2drk | 21.98 | 27.70 | 1.65 | 22.68 | 20.20 | 19.90 | 26.89 | 25.62 | 21.53 | 2.10 | 22.46 | 19.93 | 3.76 | 24.79 | 22.34 | 11.02 | 17.56 | 24.87 | 24.47 | 20.06 |
| 2dyp | 16.36 | 20.34 | 16.59 | 26.83 | 16.70 | 20.97 | 20.49 | 20.14 | 20.69 | 20.09 | 18.16 | 19.18 | 22.11 | 26.68 | 21.48 | 17.30 | 18.53 | 17.59 | 18.30 | 20.92 |
| 2fff | 29.23 | 28.52 | 53.72 | 40.34 | 53.07 | 34.99 | 45.53 | 29.79 | 45.06 | 28.41 | 40.97 | 40.94 | 43.87 | 28.90 | 31.38 | 26.39 | 32.73 | 44.23 | 23.90 | 30.86 |
| 2ffu | 15.66 | 22.02 | 21.57 | 15.48 | 15.56 | 19.67 | 15.42 | 15.66 | 14.60 | 16.11 | 12.52 | 14.86 | 14.20 | 21.01 | 14.74 | 19.08 | 14.33 | 14.14 | 22.40 | 14.14 |
| 2fka | 91.35 | 91.26 | 91.38 | 91.18 | 94.00 | 91.37 | 91.40 | 94.02 | 93.99 | 93.96 | 93.93 | 93.97 | 94.07 | 96.67 | 88.36 | 96.69 | 93.92 | 96.68 | 96.63 | 96.65 |
| 2fmf | 106.47 | 104.96 | 105.14 | 103.57 | 103.83 | 108.17 | 104.85 | 107.51 | 103.48 | 103.65 | 102.14 | 105.05 | 106.79 | 102.38 | 103.53 | 109.20 | 101.93 | 105.27 | 101.85 | 102.00 |
| 2fts | 47.60 | 34.74 | 37.17 | 27.68 | 45.73 | 46.86 | 28.49 | 46.28 | 13.42 | 32.71 | 45.10 | 45.39 | 24.35 | 45.61 | 45.18 | 46.79 | 40.35 | 44.96 | 16.72 | 32.12 |
| 2fvj | 8.72 | 30.76 | 40.89 | 41.24 | 35.81 | 32.87 | 30.66 | 17.76 | 33.22 | 42.13 | 9.10 | 38.60 | 4.54 | 40.32 | 42.24 | 16.51 | 32.24 | 42.84 | 42.32 | 36.17 |
| 2ho2 | 17.40 | 21.18 | 15.98 | 25.21 | 21.95 | 22.66 | 21.13 | 20.60 | 18.54 | 20.93 | 21.12 | 20.40 | 23.09 | 21.30 | 9.99 | 22.81 | 23.92 | 21.44 | 22.99 | 19.32 |
| 2ht9 | 47.94 | 48.42 | 41.32 | 47.17 | 47.16 | 48.45 | 49.31 | 47.95 | 43.09 | 49.83 | 47.32 | 51.37 | 38.05 | 47.32 | 48.52 | 47.49 | 48.78 | 48.10 | 46.56 | 42.95 |
| 2o02 | 1.04 | 14.33 | 14.82 | 14.71 | 2.72 | 29.37 | 14.09 | 15.58 | 11.63 | 5.90 | 7.47 | 23.60 | 7.54 | 2.92 | 14.44 | 7.59 | 16.37 | 8.81 | 14.88 | 14.22 |
| 2o4j | 47.82 | 48.40 | 54.37 | 40.61 | 37.78 | 52.47 | 41.92 | 38.25 | 52.17 | 41.37 | 53.22 | 44.10 | 50.76 | 50.39 | 29.85 | 43.55 | 53.07 | 59.40 | 38.57 | 36.37 |
| 2o9v | 28.69 | 28.06 | 29.06 | 26.00 | 28.09 | 21.19 | 15.11 | 23.88 | 25.78 | 29.91 | 19.29 | 22.69 | 24.41 | 27.93 | 20.08 | 26.28 | 25.75 | 26.91 | 22.53 | 26.37 |
| 2oei | 22.94 | 20.67 | 19.79 | 20.91 | 20.90 | 20.92 | 18.68 | 20.43 | 21.99 | 8.68 | 13.73 | 17.78 | 25.70 | 22.77 | 24.90 | 18.53 | 21.60 | 21.73 | 19.11 | 6.75 |
| 2p0w | 82.81 | 84.10 | 83.29 | 82.63 | 84.28 | 83.08 | 82.00 | 83.25 | 83.49 | 81.87 | 82.78 | 82.67 | 84.81 | 83.57 | 85.88 | 85.43 | 82.48 | 85.14 | 84.78 | 75.03 |
| 2p1o | 96.81 | 99.09 | 99.17 | 99.07 | 96.88 | 99.05 | 99.28 | 101.53 | 95.68 | 96.05 | 101.61 | 101.53 | 101.50 | 104.07 | 101.69 | 95.66 | 104.13 | 96.29 | 107.02 | 107.08 |
| 2p1t | 21.58 | 21.22 | 32.76 | 23.41 | 22.11 | 21.21 | 23.61 | 22.12 | 20.38 | 31.42 | 22.51 | 22.89 | 23.28 | 30.87 | 24.96 | 23.94 | 23.63 | 25.44 | 21.78 | 23.08 |
| 2p54 | 32.52 | 43.62 | 22.40 | 43.64 | 43.59 | 43.67 | 14.38 | 34.55 | 43.58 | 42.36 | 43.20 | 43.93 | 19.98 | 44.06 | 23.16 | 19.96 | 25.34 | 14.60 | 43.07 | 42.26 |
| 2peh | 5.30 | 29.45 | 23.10 | 23.75 | 27.42 | 30.05 | 26.03 | 25.20 | 25.64 | 31.13 | 25.18 | 24.04 | 22.14 | 22.29 | 25.85 | 36.45 | 21.53 | 24.21 | 23.80 | 21.54 |
| 2pux | 36.10 | 18.01 | 39.92 | 33.26 | 26.57 | 25.72 | 30.74 | 24.75 | 27.19 | 19.16 | 18.59 | 28.47 | 29.47 | 35.88 | 42.76 | 37.59 | 34.28 | 20.65 | 1.69 | 30.11 |
| 2puy | 69.97 | 70.77 | 68.87 | 70.70 | 68.98 | 67.57 | 67.32 | 67.37 | 68.71 | 70.27 | 68.99 | 68.32 | 69.01 | 70.29 | 67.72 | 68.25 | 68.38 | 70.06 | 70.13 | 69.51 |
| 2qbx | 1.32 | 6.63 | 31.91 | 4.25 | 16.59 | 16.72 | 24.79 | 30.53 | 13.21 | 30.77 | 24.17 | 6.77 | 25.59 | 36.95 | 16.54 | 28.10 | 2.52 | 25.36 | 25.45 | 25.97 |
| 2qos | 54.44 | 54.32 | 55.56 | 55.73 | 55.23 | 53.70 | 55.82 | 53.57 | 54.38 | 56.84 | 45.08 | 44.44 | 53.32 | 56.06 | 53.66 | 47.60 | 53.26 | 58.17 | 54.73 | 53.80 |
| 2qse | 32.70 | 29.69 | 32.33 | 33.08 | 32.19 | 30.68 | 29.89 | 27.22 | 37.71 | 33.34 | 38.35 | 29.44 | 32.58 | 26.07 | 22.34 | 30.23 | 29.47 | 24.97 | 34.29 | 37.31 |
| 2r7g | 16.87 | 16.12 | 15.57 | 18.74 | 16.85 | 21.75 | 11.89 | 16.40 | 15.64 | 11.02 | 19.83 | 32.42 | 21.90 | 18.60 | 16.81 | 21.80 | 20.29 | 18.86 | 28.32 | 32.13 |
| 2r9q | 77.21 | 81.25 | 82.27 | 78.71 | 81.65 | 77.05 | 81.72 | 78.59 | 77.41 | 75.43 | 75.97 | 54.62 | 82.13 | 75.81 | 56.14 | 81.53 | 56.31 | 79.14 | 76.91 | 80.11 |
| 2v8y | 27.24 | 14.87 | 26.60 | 26.56 | 37.74 | 38.46 | 23.63 | 30.27 | 36.70 | 28.38 | 40.34 | 19.55 | 37.52 | 39.70 | 40.33 | 27.33 | 31.81 | 38.17 | 24.46 | 17.43 |
| 2vkn | 30.06 | 27.06 | 28.27 | 24.47 | 27.21 | 24.93 | 25.81 | 11.84 | 15.36 | 20.71 | 27.19 | 25.01 | 23.41 | 24.11 | 25.67 | 25.26 | 32.45 | 34.26 | 20.49 | 18.29 |
| 2vr3 | 85.71 | 84.72 | 85.76 | 86.67 | 86.93 | 86.47 | 86.83 | 85.65 | 85.78 | 84.71 | 87.29 | 86.72 | 85.87 | 86.41 | 87.19 | 84.41 | 86.36 | 85.62 | 86.61 | 85.24 |
| 2vwf | 22.28 | 26.81 | 23.67 | 21.62 | 20.66 | 29.67 | 20.02 | 18.33 | 30.46 | 29.22 | 32.99 | 24.44 | 23.21 | 21.81 | 30.65 | 26.84 | 24.11 | 11.73 | 22.46 | 19.80 |
| 2w2u | 28.91 | 33.41 | 26.88 | 23.23 | 28.52 | 25.82 | 22.44 | 28.51 | 24.83 | 30.27 | 28.12 | 26.43 | 26.23 | 35.29 | 26.44 | 27.47 | 25.75 | 28.18 | 25.42 | 28.45 |
| 2whx | 42.78 | 23.14 | 27.87 | 28.13 | 30.54 | 37.66 | 30.50 | 38.87 | 55.34 | 32.79 | 34.78 | 31.82 | 55.70 | 42.29 | 34.84 | 40.38 | 32.44 | 30.25 | 22.81 | 25.36 |
| 2xrw | 27.85 | 32.48 | 37.31 | 31.40 | 33.08 | 30.38 | 33.85 | 35.80 | 24.37 | 35.39 | 39.50 | 37.18 | 37.58 | 39.02 | 25.46 | 27.24 | 25.25 | 25.71 | 39.68 | 21.90 |
| 2xu7 | 1.18 | 30.06 | 31.65 | 27.59 | 33.04 | 36.89 | 33.96 | 14.51 | 32.39 | 38.09 | 20.85 | 21.27 | 31.36 | 38.31 | 34.79 | 35.13 | 38.47 | 39.04 | 8.00 | 32.29 |
| 2xvc | 14.36 | 17.81 | 22.56 | 22.28 | 34.08 | 16.08 | 12.68 | 23.31 | 22.40 | 21.89 | 18.03 | 21.67 | 20.43 | 15.26 | 22.33 | 23.21 | 21.71 | 22.28 | 21.94 | 22.46 |
| 2zjd | 49.74 | 37.39 | 34.01 | 46.63 | 38.12 | 42.06 | 44.36 | 46.62 | 50.33 | 48.35 | 47.28 | 49.50 | 49.11 | 42.42 | 46.03 | 39.32 | 50.48 | 49.31 | 48.03 | 46.53 |
| 3asl | 20.00 | 22.61 | 24.50 | 21.24 | 25.61 | 14.72 | 25.26 | 26.98 | 24.85 | 21.45 | 19.89 | 22.79 | 8.66 | 11.65 | 22.47 | 15.34 | 10.83 | 22.51 | 18.61 | 22.94 |
| 3awr | 4.30 | 22.19 | 12.96 | 12.05 | 13.42 | 23.63 | 1.54 | 14.56 | 10.15 | 22.48 | 21.45 | 22.09 | 11.63 | 5.90 | 25.37 | 11.80 | 9.90 | 21.09 | 22.11 | 10.34 |
| 3ayu | 1.43 | 4.40 | 4.02 | 17.18 | 20.92 | 16.50 | 28.06 | 16.37 | 27.04 | 16.39 | 4.37 | 16.74 | 16.67 | 30.09 | 16.22 | 20.26 | 27.32 | 18.54 | 11.12 | 7.44 |
| 3bfq | 1.54 | 27.02 | 2.38 | 2.70 | 7.01 | 14.57 | 10.65 | 27.55 | 32.73 | 34.08 | 29.26 | 35.74 | 3.75 | 30.25 | 25.57 | 24.89 | 13.91 | 33.95 | 11.52 | 10.78 |
| 3c3r | 51.23 | 40.35 | 51.72 | 51.56 | 50.98 | 52.08 | 48.77 | 50.96 | 52.37 | 50.01 | 49.17 | 52.83 | 52.15 | 49.88 | 50.61 | 50.47 | 53.33 | 43.06 | 32.78 | 53.26 |
| 3d32 | 34.94 | 37.11 | 40.23 | 39.82 | 35.95 | 39.28 | 35.50 | 34.76 | 40.62 | 35.79 | 39.72 | 34.99 | 41.02 | 45.01 | 39.33 | 36.75 | 39.66 | 36.76 | 41.10 | 34.56 |
| 3ds4 | 1.61 | 5.71 | 11.97 | 4.88 | 9.92 | 9.20 | 11.25 | 7.94 | 10.49 | 9.19 | 10.72 | 11.20 | 7.47 | 8.84 | 10.92 | 11.99 | 6.62 | 10.83 | 2.69 | 10.53 |
| 3ery | 12.88 | 1.47 | 7.69 | 15.81 | 14.70 | 3.25 | 15.40 | 4.34 | 2.16 | 14.66 | 3.17 | 14.56 | 11.87 | 15.58 | 15.20 | 14.35 | 11.73 | 15.45 | 5.68 | 13.53 |
| 3fdo | 28.05 | 7.48 | 12.13 | 9.05 | 11.84 | 17.04 | 1.34 | 7.22 | 17.98 | 28.67 | 18.10 | 16.26 | 7.29 | 11.32 | 15.03 | 11.91 | 4.46 | 11.46 | 10.46 | 11.41 |
| 3g2s | 1.29 | 37.54 | 35.64 | 17.91 | 33.93 | 18.66 | 34.36 | 24.17 | 20.07 | 20.31 | 18.42 | 36.07 | 16.34 | 35.41 | 37.84 | 16.94 | 37.13 | 22.03 | 15.01 | 35.70 |
| 3gyt | 26.25 | 26.14 | 29.81 | 39.19 | 38.97 | 12.48 | 38.39 | 41.53 | 18.29 | 38.21 | 39.61 | 41.69 | 35.87 | 19.29 | 28.34 | 37.62 | 41.12 | 29.78 | 15.51 | 24.12 |
| 3h1z | 28.88 | 27.29 | 25.64 | 36.40 | 41.73 | 30.82 | 28.76 | 31.10 | 37.38 | 33.77 | 34.23 | 32.95 | 37.48 | 32.35 | 34.42 | 35.33 | 33.58 | 36.95 | 33.83 | 34.07 |
| 3i5r | 18.91 | 19.86 | 25.37 | 27.10 | 22.61 | 30.44 | 28.17 | 29.86 | 1.82 | 26.91 | 16.22 | 21.91 | 12.61 | 3.73 | 29.50 | 26.64 | 17.20 | 31.52 | 28.04 | 21.94 |
| 3ivv | 27.52 | 26.89 | 30.39 | 11.31 | 21.91 | 12.47 | 30.43 | 23.12 | 26.99 | 10.38 | 27.57 | 28.16 | 31.67 | 27.72 | 10.21 | 25.07 | 26.19 | 23.50 | 14.50 | 21.30 |
| 3kmr | 34.59 | 21.97 | 31.12 | 23.23 | 22.08 | 21.88 | 23.78 | 24.58 | 7.86 | 23.36 | 29.82 | 34.67 | 20.28 | 22.07 | 22.30 | 35.05 | 7.33 | 21.45 | 34.72 | 22.11 |
| 3kuj | 1.66 | 3.55 | 25.09 | 21.92 | 21.75 | 23.48 | 21.99 | 22.04 | 22.03 | 21.88 | 23.47 | 19.21 | 19.16 | 14.47 | 10.97 | 26.96 | 15.41 | 20.82 | 2.30 | 21.29 |
| 3kus | 25.83 | 24.20 | 15.90 | 22.41 | 23.53 | 16.24 | 11.49 | 13.40 | 11.28 | 10.81 | 15.27 | 17.04 | 19.65 | 22.58 | 22.07 | 7.03 | 19.04 | 8.64 | 11.88 | 25.08 |
| 3l0e | 23.65 | 34.43 | 34.13 | 33.66 | 26.18 | 21.32 | 20.71 | 21.64 | 28.05 | 26.97 | 29.98 | 35.30 | 38.09 | 27.94 | 30.96 | 33.79 | 22.84 | 40.97 | 23.49 | 34.40 |
| 3ll8 | 28.69 | 31.98 | 28.57 | 35.35 | 15.90 | 39.94 | 30.94 | 28.44 | 29.07 | 36.47 | 33.75 | 40.24 | 40.73 | 27.41 | 25.95 | 41.90 | 29.42 | 33.50 | 28.00 | 33.52 |
| 3llz | 1.56 | 22.15 | 30.39 | 31.07 | 30.64 | 23.34 | 29.27 | 36.22 | 30.68 | 31.41 | 28.75 | 3.52 | 35.15 | 36.89 | 27.03 | 8.73 | 26.26 | 20.19 | 30.64 | 32.33 |
| 3obq | 24.10 | 24.42 | 21.51 | 28.41 | 22.81 | 23.15 | 26.52 | 19.40 | 11.70 | 21.38 | 16.84 | 32.12 | 28.93 | 8.37 | 21.00 | 14.64 | 30.00 | 34.17 | 18.81 | 32.25 |
| 3olf | 22.76 | 21.18 | 15.20 | 1.38 | 35.69 | 21.18 | 32.43 | 25.01 | 36.51 | 32.90 | 19.14 | 9.97 | 22.66 | 23.67 | 35.13 | 42.86 | 33.47 | 38.18 | 14.78 | 25.69 |
| 3p72 | 35.91 | 28.23 | 34.00 | 35.92 | 26.65 | 34.08 | 35.60 | 24.89 | 26.70 | 34.83 | 27.27 | 35.56 | 35.02 | 26.94 | 27.33 | 26.91 | 28.90 | 34.77 | 27.47 | 38.14 |
| 3p8f | 19.05 | 15.77 | 7.50 | 37.39 | 12.05 | 27.72 | 17.23 | 32.16 | 38.42 | 36.37 | 15.33 | 17.77 | 18.68 | 36.20 | 37.38 | 34.96 | 18.68 | 13.11 | 14.87 | 6.73 |
| 3ptl | 27.01 | 24.77 | 26.38 | 26.82 | 25.15 | 18.47 | 30.16 | 34.99 | 34.23 | 18.38 | 19.59 | 29.46 | 33.90 | 28.94 | 25.10 | 37.42 | 24.83 | 39.42 | 41.36 | 30.52 |
| 3qis | 15.36 | 9.48 | 22.76 | 14.88 | 13.22 | 21.56 | 16.98 | 15.86 | 12.17 | 12.87 | 15.44 | 13.17 | 14.02 | 11.00 | 26.97 | 16.82 | 9.73 | 11.88 | 15.94 | 15.87 |
| 3rm1 | 1.45 | 16.97 | 18.83 | 5.90 | 10.40 | 19.78 | 21.09 | 8.86 | 8.19 | 18.86 | 18.94 | 8.15 | 8.31 | 18.89 | 19.11 | 18.61 | 19.16 | 17.06 | 11.09 | 19.07 |
| 3rqg | 40.31 | 43.14 | 40.88 | 41.05 | 41.50 | 38.89 | 41.26 | 43.51 | 43.42 | 42.22 | 30.79 | 43.37 | 41.25 | 48.92 | 44.61 | 43.52 | 30.73 | 43.47 | 43.98 | 39.46 |
| 3sfj | 30.62 | 32.38 | 32.50 | 24.01 | 25.62 | 26.12 | 24.91 | 28.78 | 30.01 | 23.64 | 22.75 | 25.05 | 32.52 | 31.60 | 17.32 | 31.28 | 35.35 | 27.83 | 30.43 | 29.87 |
| 3so6 | 51.18 | 52.52 | 51.30 | 50.11 | 52.35 | 51.16 | 53.41 | 53.43 | 54.71 | 52.18 | 52.27 | 52.83 | 52.74 | 51.74 | 51.35 | 52.23 | 54.46 | 53.13 | 54.89 | 54.16 |
| 3tjv | 1.18 | 32.14 | 30.31 | 32.02 | 29.42 | 21.99 | 16.60 | 10.70 | 24.63 | 10.84 | 31.06 | 16.41 | 17.61 | 3.82 | 19.33 | 31.37 | 30.25 | 13.27 | 29.97 | 17.54 |
| 3tzy | 45.84 | 46.14 | 46.78 | 45.71 | 47.98 | 42.06 | 44.86 | 35.33 | 47.00 | 48.86 | 49.47 | 45.05 | 48.09 | 48.26 | 47.00 | 48.26 | 49.50 | 48.81 | 47.77 | 46.60 |
| 3u9q | 0.88 | 25.82 | 3.26 | 29.34 | 28.77 | 34.05 | 4.84 | 27.39 | 8.76 | 5.97 | 27.51 | 37.89 | 34.12 | 28.81 | 6.10 | 40.21 | 33.94 | 22.18 | 26.06 | 34.79 |
| 3up3 | 21.95 | 29.60 | 31.20 | 28.76 | 20.63 | 2.64 | 27.99 | 28.17 | 21.30 | 24.82 | 16.17 | 34.49 | 29.77 | 2.11 | 28.43 | 25.79 | 30.85 | 28.43 | 29.44 | 21.50 |
| 3v2x | 7.71 | 12.52 | 20.78 | 24.80 | 1.54 | 25.19 | 23.62 | 9.73 | 26.22 | 7.60 | 8.51 | 16.12 | 7.51 | 28.94 | 7.43 | 26.99 | 14.36 | 23.79 | 8.86 | 13.32 |
| 3vtc | 22.20 | 18.38 | 36.38 | 37.64 | 36.53 | 33.79 | 26.08 | 23.88 | 33.62 | 25.01 | 40.60 | 20.98 | 34.36 | 24.45 | 39.44 | 44.32 | 27.92 | 19.60 | 41.69 | 34.55 |
| 3w1b | 65.15 | 65.89 | 65.95 | 65.12 | 64.93 | 65.17 | 65.55 | 36.67 | 66.61 | 66.99 | 67.30 | 65.51 | 37.14 | 66.09 | 66.28 | 65.98 | 66.46 | 64.91 | 66.76 | 65.69 |
| 3zqh | 24.20 | 36.81 | 31.83 | 34.06 | 17.17 | 25.76 | 33.77 | 23.35 | 32.28 | 14.78 | 24.07 | 34.42 | 24.65 | 31.70 | 30.15 | 29.01 | 26.07 | 13.94 | 15.10 | 32.83 |
| 4b4n | 26.05 | 33.49 | 11.18 | 30.69 | 13.29 | 30.99 | 30.48 | 10.77 | 12.35 | 31.56 | 12.84 | 12.38 | 26.33 | 31.65 | 2.21 | 18.43 | 32.08 | 31.94 | 10.60 | 18.24 |
| 4dcb | 49.74 | 23.92 | 23.11 | 27.83 | 48.36 | 48.92 | 15.91 | 14.32 | 50.46 | 49.00 | 27.82 | 47.79 | 14.34 | 27.34 | 50.90 | 25.78 | 48.01 | 50.19 | 22.86 | 50.30 |
| 4e34 | 16.99 | 38.06 | 23.92 | 35.47 | 24.99 | 20.39 | 27.11 | 28.64 | 37.58 | 21.04 | 25.64 | 14.63 | 26.59 | 25.24 | 27.21 | 24.91 | 28.13 | 35.31 | 17.38 | 25.91 |
| 4eik | 30.31 | 30.08 | 29.40 | 25.75 | 28.75 | 27.31 | 26.88 | 24.38 | 26.57 | 23.95 | 26.98 | 26.47 | 26.95 | 26.73 | 26.48 | 28.00 | 27.10 | 28.60 | 23.31 | 29.37 |
| 4ery | 28.47 | 32.90 | 31.03 | 32.35 | 29.77 | 31.98 | 32.69 | 32.44 | 29.58 | 33.57 | 34.84 | 11.55 | 34.19 | 35.13 | 32.59 | 33.58 | 16.11 | 32.85 | 3.65 | 37.72 |
| 4f14 | 19.69 | 18.67 | 18.96 | 22.76 | 21.15 | 22.53 | 15.77 | 18.58 | 15.81 | 8.51 | 22.75 | 20.23 | 21.47 | 20.74 | 30.00 | 22.64 | 19.06 | 24.03 | 20.14 | 20.52 |
| 4f1z | 20.97 | 26.46 | 9.31 | 20.74 | 19.94 | 27.64 | 25.45 | 25.42 | 24.54 | 26.32 | 24.81 | 23.82 | 18.81 | 20.83 | 22.86 | 25.50 | 17.12 | 31.78 | 29.76 | 28.26 |
| 4gq6 | 9.79 | 7.75 | 10.79 | 11.27 | 9.47 | 9.21 | 9.79 | 10.85 | 12.64 | 16.55 | 6.09 | 7.42 | 12.56 | 14.17 | 11.72 | 14.27 | 10.90 | 11.95 | 8.69 | 10.70 |
| 4gxl | 31.19 | 28.33 | 31.58 | 13.62 | 28.26 | 26.63 | 3.15 | 30.95 | 29.87 | 11.77 | 23.37 | 28.23 | 7.60 | 30.88 | 30.02 | 31.84 | 13.58 | 31.20 | 29.70 | 31.30 |
| 4gyw | 24.64 | 24.05 | 18.30 | 19.23 | 26.13 | 24.05 | 27.37 | 15.12 | 25.42 | 21.16 | 15.43 | 14.47 | 18.08 | 24.84 | 22.67 | 25.14 | 32.77 | 17.23 | 18.01 | 16.39 |
| 4h4f | 65.52 | 64.38 | 63.22 | 63.51 | 64.35 | 68.21 | 66.84 | 63.10 | 62.79 | 64.27 | 67.21 | 67.54 | 64.53 | 62.88 | 65.52 | 63.58 | 68.10 | 64.39 | 65.44 | 70.49 |
| 4hom | 48.43 | 48.21 | 50.12 | 48.46 | 48.10 | 47.88 | 48.29 | 47.22 | 49.07 | 49.10 | 54.53 | 49.95 | 49.94 | 47.95 | 50.09 | 49.01 | 48.40 | 55.19 | 47.96 | 47.67 |
| 4htp | 34.53 | 38.18 | 27.50 | 44.07 | 26.65 | 35.14 | 28.58 | 32.89 | 36.24 | 17.85 | 38.16 | 23.82 | 18.35 | 22.33 | 16.62 | 25.25 | 24.01 | 30.58 | 36.61 | 36.67 |
| 4iim | 1.50 | 19.61 | 14.80 | 6.48 | 21.39 | 3.09 | 25.56 | 22.75 | 23.97 | 11.69 | 22.85 | 21.72 | 23.43 | 14.47 | 24.43 | 24.80 | 28.85 | 10.35 | 23.76 | 4.69 |
| 4j8s | 45.68 | 49.99 | 48.54 | 36.75 | 49.57 | 49.49 | 50.78 | 50.23 | 47.22 | 47.61 | 41.95 | 38.97 | 47.28 | 48.64 | 49.71 | 39.49 | 41.49 | 40.94 | 44.01 | 49.58 |
| 4k0u | 1.39 | 7.57 | 4.83 | 4.55 | 13.68 | 14.23 | 14.41 | 14.61 | 5.62 | 13.76 | 4.31 | 11.03 | 4.09 | 5.93 | 9.34 | 13.07 | 12.30 | 13.83 | 13.90 | 14.24 |

**S4(c). L-RMSD values of all 20 poses obtained after blind docking by ZDOCK on 133 protein-peptide complexes.**

| **ID** | **Pose1** | **Pose2** | **Pose3** | **Pose4** | **Pose5** | **Pose6** | **Pose7** | **Pose8** | **Pose9** | **Pose10** | **Pose11** | **Pose12** | **Pose13** | **Pose14** | **Pose15** | **Pose16** | **Pose17** | **Pose18** | **Pose19** | **Pose20** |
| --- | --- | --- | --- | --- | --- | --- | --- | --- | --- | --- | --- | --- | --- | --- | --- | --- | --- | --- | --- | --- |
| 1cjr | 1.43 | 3.17 | 1.77 | 1.85 | 24.39 | 1.96 | 26.91 | 27.76 | 24.12 | 27.26 | 24.75 | 27.27 | 2.79 | 25.45 | 23.98 | 13.94 | 26.76 | 27.63 | 25.86 | 24.15 |
| 1cka | 0.60 | 1.88 | 2.28 | 15.55 | 15.48 | 1.73 | 15.78 | 2.87 | 17.13 | 16.95 | 17.23 | 15.33 | 4.55 | 2.39 | 15.35 | 4.10 | 8.54 | 8.93 | 5.27 | 2.51 |
| 1cvu | 37.16 | 39.67 | 37.00 | 38.19 | 37.28 | 38.49 | 37.27 | 37.43 | 36.91 | 38.77 | 37.38 | 38.03 | 38.72 | 37.41 | 37.36 | 62.31 | 38.80 | 36.61 | 37.44 | 37.22 |
| 1d4t | 2.42 | 1.71 | 12.72 | 4.01 | 9.66 | 18.56 | 6.38 | 11.83 | 12.41 | 8.77 | 20.72 | 11.09 | 4.16 | 7.20 | 7.60 | 2.81 | 6.23 | 9.51 | 14.50 | 11.14 |
| 1eg4 | 45.30 | 45.40 | 40.99 | 43.22 | 42.45 | 47.17 | 42.00 | 43.59 | 44.57 | 43.30 | 42.48 | 41.61 | 46.58 | 46.58 | 42.29 | 44.08 | 45.45 | 45.80 | 45.77 | 44.17 |
| 1h6w | 0.95 | 1.20 | 1.98 | 1.80 | 1.88 | 2.09 | 1.44 | 16.70 | 16.50 | 12.36 | 2.84 | 18.33 | 16.16 | 18.54 | 16.75 | 12.80 | 18.67 | 19.52 | 18.10 | 16.06 |
| 1hc9 | 22.05 | 0.98 | 20.41 | 3.04 | 19.16 | 21.14 | 17.41 | 16.53 | 18.66 | 16.23 | 19.15 | 21.00 | 21.65 | 16.41 | 18.23 | 21.61 | 15.49 | 22.40 | 21.77 | 19.62 |
| 1jbu | 1.90 | 1.88 | 41.38 | 45.38 | 2.51 | 45.73 | 46.02 | 45.84 | 46.01 | 47.19 | 45.26 | 44.62 | 46.10 | 45.48 | 45.57 | 45.70 | 45.14 | 46.65 | 46.50 | 46.32 |
| 1k5n | 1.77 | 2.28 | 25.65 | 23.16 | 2.96 | 22.29 | 21.35 | 23.10 | 22.42 | 22.52 | 13.90 | 23.51 | 22.96 | 5.60 | 22.87 | 21.82 | 24.95 | 26.24 | 23.28 | 23.43 |
| 1mfg | 27.27 | 26.61 | 23.80 | 26.43 | 28.53 | 23.82 | 26.24 | 22.72 | 25.03 | 26.56 | 26.96 | 24.67 | 28.34 | 22.96 | 27.48 | 29.33 | 26.16 | 23.11 | 1.90 | 29.00 |
| 1nln | 29.69 | 30.54 | 28.50 | 33.21 | 31.09 | 31.20 | 32.79 | 34.92 | 33.14 | 33.46 | 35.98 | 29.78 | 31.00 | 30.06 | 33.40 | 30.84 | 33.63 | 30.31 | 38.45 | 29.52 |
| 1nq7 | 36.04 | 38.46 | 36.50 | 40.03 | 23.45 | 36.67 | 22.38 | 23.86 | 37.65 | 35.29 | 44.04 | 36.92 | 36.49 | 36.02 | 22.01 | 35.29 | 43.97 | 36.68 | 43.13 | 43.40 |
| 1ntv | 3.55 | 16.28 | 15.13 | 9.91 | 10.29 | 5.75 | 1.05 | 14.78 | 15.06 | 14.64 | 15.05 | 9.44 | 11.65 | 15.20 | 3.58 | 16.11 | 14.69 | 15.81 | 1.73 | 16.67 |
| 1nx1 | 34.73 | 35.51 | 33.94 | 33.05 | 35.44 | 35.34 | 33.60 | 34.31 | 36.27 | 39.98 | 32.30 | 35.46 | 0.93 | 34.82 | 36.78 | 36.63 | 35.67 | 35.55 | 38.90 | 33.77 |
| 1oai | 25.25 | 22.50 | 4.26 | 1.61 | 21.66 | 25.55 | 25.13 | 24.61 | 3.14 | 17.98 | 25.63 | 25.28 | 21.55 | 22.19 | 21.88 | 22.16 | 21.71 | 15.16 | 25.28 | 25.30 |
| 1oj5 | 16.07 | 14.58 | 13.53 | 15.14 | 14.56 | 5.63 | 13.18 | 7.47 | 16.31 | 9.03 | 4.76 | 16.28 | 13.23 | 13.99 | 15.84 | 13.52 | 17.06 | 9.92 | 16.11 | 12.07 |
| 1ou8 | 8.49 | 9.94 | 9.96 | 9.91 | 9.95 | 13.98 | 10.94 | 8.52 | 10.78 | 15.42 | 8.36 | 10.05 | 10.11 | 15.16 | 29.80 | 13.88 | 15.62 | 10.63 | 15.61 | 12.19 |
| 1ow6 | 37.97 | 39.55 | 17.77 | 34.69 | 38.10 | 39.52 | 38.50 | 39.34 | 16.42 | 38.03 | 39.90 | 36.15 | 38.11 | 39.25 | 37.59 | 40.19 | 39.84 | 34.94 | 40.14 | 17.54 |
| 1pzl | 42.44 | 39.19 | 41.98 | 39.33 | 41.49 | 42.11 | 40.05 | 39.99 | 44.12 | 42.57 | 43.12 | 39.72 | 43.61 | 39.14 | 44.05 | 41.61 | 44.84 | 43.82 | 44.45 | 41.35 |
| 1qkz | 9.13 | 8.91 | 9.41 | 8.55 | 9.04 | 8.52 | 8.81 | 9.35 | 9.41 | 9.31 | 9.36 | 9.29 | 9.42 | 4.57 | 4.86 | 9.00 | 9.17 | 8.60 | 9.51 | 10.00 |
| 1rst | 7.69 | 3.15 | 10.48 | 2.22 | 7.89 | 10.65 | 9.83 | 10.37 | 8.74 | 2.64 | 7.98 | 3.26 | 6.95 | 2.79 | 3.51 | 7.38 | 9.83 | 6.55 | 3.94 | 7.52 |
| 1rxz | 13.24 | 12.30 | 1.89 | 3.94 | 13.63 | 41.13 | 4.46 | 1.55 | 6.39 | 13.15 | 3.44 | 38.97 | 13.14 | 20.50 | 2.48 | 11.96 | 17.36 | 9.42 | 41.79 | 31.43 |
| 1sfi | 15.18 | 11.86 | 14.31 | 15.21 | 21.12 | 17.06 | 18.15 | 17.11 | 21.84 | 23.87 | 13.77 | 22.30 | 23.99 | 15.58 | 20.81 | 12.31 | 14.89 | 19.23 | 17.57 | 11.11 |
| 1ssh | 1.85 | 1.86 | 1.48 | 18.78 | 4.93 | 18.76 | 9.08 | 18.60 | 20.47 | 19.76 | 2.51 | 3.35 | 5.48 | 5.28 | 6.69 | 1.30 | 3.68 | 4.00 | 3.06 | 18.58 |
| 1t08 | 24.90 | 26.11 | 37.57 | 35.16 | 26.08 | 24.60 | 19.78 | 24.30 | 25.18 | 24.04 | 15.27 | 30.22 | 26.82 | 25.92 | 28.21 | 20.11 | 40.85 | 26.09 | 25.22 | 24.89 |
| 1t4f | 1.05 | 1.69 | 1.87 | 0.72 | 2.04 | 8.55 | 9.10 | 10.94 | 2.94 | 1.37 | 21.19 | 19.01 | 5.59 | 4.48 | 1.66 | 12.58 | 12.12 | 19.29 | 6.79 | 12.11 |
| 1t7r | 29.38 | 40.37 | 2.94 | 1.68 | 35.77 | 38.28 | 38.17 | 5.50 | 42.99 | 37.72 | 1.22 | 2.61 | 38.86 | 38.60 | 39.41 | 39.01 | 27.76 | 39.20 | 27.61 | 40.36 |
| 1tfc | 1.97 | 2.32 | 26.99 | 23.44 | 25.56 | 26.13 | 2.72 | 24.88 | 26.65 | 23.82 | 26.79 | 18.24 | 25.32 | 36.49 | 3.33 | 24.83 | 1.70 | 1.07 | 27.52 | 25.61 |
| 1u00 | 2.87 | 1.42 | 2.77 | 1.49 | 3.13 | 7.44 | 2.55 | 2.73 | 6.53 | 2.31 | 3.31 | 2.97 | 5.85 | 17.35 | 5.68 | 5.10 | 2.44 | 33.26 | 7.00 | 33.24 |
| 1uj0 | 1.84 | 0.76 | 2.83 | 18.07 | 2.34 | 2.82 | 2.27 | 17.99 | 18.33 | 17.80 | 18.25 | 17.82 | 17.62 | 9.03 | 13.76 | 17.66 | 14.03 | 5.42 | 4.72 | 17.79 |
| 1x2r | 8.57 | 2.33 | 3.89 | 7.98 | 4.35 | 2.89 | 7.97 | 9.38 | 2.70 | 7.74 | 8.64 | 6.34 | 7.39 | 8.06 | 7.53 | 6.14 | 2.35 | 4.99 | 8.19 | 4.11 |
| 1xoc | 24.97 | 1.10 | 33.48 | 1.17 | 32.76 | 24.76 | 28.40 | 25.58 | 22.90 | 23.22 | 25.50 | 25.28 | 21.06 | 29.32 | 31.71 | 24.67 | 27.47 | 24.41 | 25.90 | 32.80 |
| 1ymt | 1.29 | 1.40 | 7.90 | 2.79 | 1.46 | 3.58 | 1.97 | 8.05 | 1.99 | 24.09 | 24.22 | 23.16 | 23.47 | 5.81 | 23.24 | 22.54 | 1.79 | 1.50 | 23.40 | 3.28 |
| 1yuc | 1.94 | 13.25 | 16.36 | 23.66 | 23.87 | 2.07 | 24.90 | 16.79 | 35.03 | 1.33 | 29.27 | 32.73 | 25.21 | 23.73 | 27.13 | 26.72 | 22.81 | 33.55 | 24.40 | 14.70 |
| 1ywo | 17.21 | 4.81 | 6.13 | 4.91 | 17.77 | 21.16 | 17.40 | 14.21 | 5.79 | 13.93 | 4.63 | 16.93 | 5.85 | 18.29 | 5.15 | 5.34 | 16.77 | 1.89 | 17.18 | 17.30 |
| 2a25 | 26.27 | 27.93 | 26.96 | 30.91 | 25.04 | 29.27 | 27.24 | 27.39 | 25.68 | 27.31 | 25.88 | 27.77 | 26.10 | 27.55 | 3.85 | 25.21 | 29.16 | 25.81 | 27.39 | 27.27 |
| 2a3i | 37.36 | 38.11 | 38.06 | 39.26 | 38.22 | 38.40 | 38.27 | 40.22 | 37.84 | 38.35 | 37.86 | 38.41 | 38.36 | 38.14 | 39.36 | 37.59 | 38.66 | 38.17 | 38.46 | 37.43 |
| 2aq9 | 16.36 | 19.06 | 14.06 | 19.30 | 32.63 | 12.63 | 30.23 | 14.63 | 19.08 | 16.49 | 18.25 | 13.02 | 14.03 | 19.42 | 19.17 | 30.42 | 32.38 | 32.74 | 31.85 | 35.16 |
| 2b9h | 29.47 | 1.10 | 2.65 | 1.75 | 44.21 | 27.56 | 44.43 | 41.87 | 46.32 | 40.86 | 25.42 | 41.43 | 28.49 | 31.15 | 49.14 | 33.98 | 47.43 | 33.49 | 27.01 | 44.45 |
| 2bba | 29.86 | 30.33 | 37.33 | 30.68 | 30.10 | 29.64 | 28.03 | 29.47 | 31.39 | 30.26 | 29.73 | 29.33 | 29.76 | 29.02 | 30.65 | 29.34 | 38.84 | 27.80 | 30.19 | 30.42 |
| 2cch | 0.86 | 27.78 | 36.33 | 35.93 | 35.14 | 27.88 | 35.66 | 20.54 | 27.42 | 37.02 | 35.91 | 29.05 | 27.94 | 36.70 | 34.47 | 41.26 | 38.34 | 36.38 | 30.04 | 36.06 |
| 2ce8 | 36.16 | 34.52 | 34.20 | 33.21 | 35.68 | 34.06 | 36.38 | 36.64 | 37.25 | 35.24 | 35.15 | 35.66 | 38.34 | 37.35 | 33.38 | 37.13 | 36.76 | 39.57 | 38.79 | 38.16 |
| 2d0n | 1.30 | 2.43 | 1.63 | 2.71 | 2.31 | 3.34 | 3.62 | 2.94 | 3.70 | 4.40 | 16.97 | 1.71 | 16.25 | 2.97 | 2.30 | 11.66 | 2.53 | 20.05 | 29.06 | 1.97 |
| 2drk | 1.54 | 2.23 | 3.55 | 1.18 | 17.53 | 9.83 | 1.89 | 17.46 | 17.51 | 17.25 | 20.16 | 17.63 | 17.18 | 18.05 | 13.76 | 1.58 | 19.25 | 17.83 | 17.21 | 16.98 |
| 2dyp | 2.31 | 1.88 | 1.43 | 1.43 | 19.71 | 24.08 | 24.00 | 2.78 | 1.91 | 19.95 | 1.93 | 20.51 | 21.05 | 22.73 | 62.04 | 21.12 | 18.90 | 20.22 | 20.87 | 61.71 |
| 2fff | 51.16 | 51.27 | 52.74 | 50.32 | 52.65 | 49.12 | 51.48 | 49.73 | 49.81 | 51.82 | 53.02 | 48.82 | 51.39 | 52.47 | 52.98 | 51.03 | 51.97 | 51.88 | 50.76 | 51.08 |
| 2ffu | 15.79 | 1.84 | 1.92 | 15.98 | 15.55 | 15.59 | 15.63 | 1.49 | 15.89 | 15.69 | 4.30 | 1.72 | 16.22 | 3.00 | 4.42 | 4.50 | 15.76 | 2.16 | 9.80 | 3.50 |
| 2fka | 29.19 | 7.10 | 29.43 | 5.46 | 27.28 | 25.28 | 25.32 | 26.26 | 25.68 | 26.82 | 6.98 | 25.05 | 26.35 | 28.59 | 4.80 | 25.81 | 0.86 | 26.01 | 29.74 | 7.00 |
| 2fmf | 26.52 | 30.66 | 7.75 | 31.99 | 4.34 | 31.26 | 30.03 | 31.74 | 4.66 | 30.13 | 30.93 | 29.86 | 28.87 | 28.09 | 27.91 | 26.73 | 28.34 | 32.39 | 28.92 | 30.07 |
| 2fts | 0.49 | 1.77 | 3.55 | 3.65 | 2.31 | 1.96 | 5.42 | 3.19 | 6.79 | 5.33 | 13.09 | 16.15 | 17.19 | 16.46 | 3.42 | 18.42 | 11.30 | 29.75 | 29.16 | 13.35 |
| 2fvj | 42.99 | 46.00 | 43.23 | 42.45 | 42.33 | 43.53 | 45.54 | 43.40 | 42.35 | 42.03 | 43.86 | 47.28 | 44.34 | 43.67 | 42.90 | 44.93 | 42.92 | 42.17 | 45.57 | 45.26 |
| 2ho2 | 7.36 | 8.22 | 7.01 | 3.97 | 17.24 | 20.94 | 7.72 | 7.23 | 19.76 | 17.06 | 17.06 | 6.66 | 17.63 | 26.15 | 20.93 | 14.07 | 20.87 | 9.70 | 5.81 | 9.75 |
| 2ht9 | 28.97 | 28.50 | 27.31 | 26.80 | 30.75 | 28.00 | 27.13 | 27.63 | 25.62 | 26.11 | 23.03 | 24.24 | 30.52 | 33.96 | 25.79 | 25.35 | 29.93 | 25.20 | 28.61 | 29.44 |
| 2o02 | 32.93 | 32.52 | 33.36 | 32.33 | 18.40 | 31.38 | 23.15 | 32.47 | 34.03 | 31.11 | 34.96 | 30.54 | 29.74 | 30.18 | 33.11 | 35.22 | 22.80 | 32.06 | 36.83 | 36.62 |
| 2o4j | 34.74 | 33.99 | 34.41 | 34.90 | 34.25 | 34.61 | 35.94 | 33.94 | 33.07 | 34.17 | 34.82 | 34.84 | 32.25 | 34.72 | 35.18 | 33.81 | 33.19 | 33.22 | 35.25 | 33.75 |
| 2o9v | 4.07 | 17.42 | 4.16 | 4.16 | 17.41 | 5.24 | 21.01 | 5.53 | 4.08 | 21.04 | 1.37 | 4.85 | 18.94 | 17.43 | 4.12 | 22.43 | 5.47 | 17.03 | 17.69 | 1.41 |
| 2oei | 9.10 | 15.58 | 3.79 | 9.86 | 9.52 | 17.81 | 9.30 | 3.80 | 6.68 | 15.12 | 3.54 | 3.79 | 17.80 | 15.54 | 15.32 | 15.91 | 17.02 | 4.80 | 3.50 | 2.75 |
| 2p0w | 36.46 | 26.17 | 22.92 | 30.65 | 38.24 | 22.10 | 25.24 | 21.92 | 32.14 | 31.27 | 28.05 | 25.66 | 39.73 | 30.32 | 32.81 | 26.68 | 30.37 | 29.23 | 29.56 | 25.96 |
| 2p1o | 11.63 | 11.42 | 1.20 | 9.74 | 2.80 | 10.03 | 1.86 | 12.16 | 11.10 | 9.44 | 8.71 | 12.43 | 5.19 | 12.00 | 11.64 | 9.30 | 10.01 | 9.11 | 11.37 | 8.71 |
| 2p1t | 0.95 | 7.59 | 2.35 | 35.39 | 37.10 | 5.74 | 36.03 | 4.58 | 6.13 | 35.37 | 25.49 | 35.70 | 1.93 | 35.79 | 5.25 | 36.75 | 35.72 | 36.08 | 9.98 | 35.94 |
| 2p54 | 42.95 | 43.96 | 42.72 | 39.92 | 39.98 | 40.96 | 44.85 | 43.66 | 28.05 | 32.03 | 41.88 | 42.02 | 31.61 | 43.94 | 43.82 | 45.18 | 44.05 | 26.92 | 45.41 | 28.69 |
| 2peh | 1.06 | 1.80 | 2.54 | 3.70 | 2.76 | 2.18 | 2.43 | 3.33 | 2.90 | 3.90 | 3.71 | 2.15 | 9.36 | 4.58 | 3.41 | 3.66 | 4.27 | 3.74 | 4.25 | 13.27 |
| 2pux | 32.43 | 31.65 | 33.30 | 30.50 | 35.49 | 33.29 | 37.77 | 40.76 | 35.61 | 32.61 | 32.18 | 34.16 | 34.02 | 1.20 | 32.48 | 46.17 | 33.11 | 32.64 | 32.51 | 34.30 |
| 2puy | 2.32 | 18.49 | 15.77 | 13.62 | 2.84 | 20.11 | 14.70 | 17.43 | 18.55 | 31.35 | 23.37 | 16.58 | 16.04 | 27.68 | 1.61 | 4.13 | 14.79 | 10.77 | 15.99 | 1.44 |
| 2qbx | 38.85 | 38.14 | 35.38 | 35.64 | 39.11 | 40.68 | 37.34 | 37.89 | 38.42 | 39.47 | 38.01 | 37.20 | 35.85 | 38.52 | 39.44 | 37.81 | 39.50 | 39.40 | 38.39 | 35.23 |
| 2qos | 11.10 | 1.97 | 10.90 | 10.67 | 2.06 | 4.89 | 1.97 | 10.51 | 1.59 | 4.48 | 7.37 | 5.63 | 11.55 | 11.23 | 8.77 | 11.86 | 9.83 | 10.65 | 11.02 | 10.63 |
| 2qse | 32.42 | 31.03 | 31.94 | 30.80 | 31.49 | 32.90 | 29.59 | 29.58 | 31.70 | 34.31 | 31.99 | 0.69 | 33.72 | 31.31 | 33.26 | 30.83 | 31.02 | 33.45 | 31.20 | 31.68 |
| 2r7g | 38.80 | 37.20 | 37.70 | 37.61 | 39.23 | 37.87 | 40.19 | 39.18 | 38.06 | 1.02 | 1.88 | 1.83 | 37.29 | 39.50 | 37.46 | 37.72 | 37.42 | 2.12 | 37.15 | 1.63 |
| 2r9q | 10.48 | 7.05 | 8.36 | 7.25 | 9.38 | 10.60 | 7.38 | 9.71 | 9.39 | 7.77 | 4.57 | 9.80 | 7.28 | 2.90 | 10.90 | 7.48 | 8.00 | 8.51 | 1.01 | 8.69 |
| 2v8y | 2.20 | 42.10 | 42.44 | 43.89 | 43.54 | 42.44 | 43.60 | 42.84 | 42.47 | 17.95 | 39.33 | 42.73 | 0.69 | 42.42 | 44.04 | 43.26 | 18.18 | 15.22 | 42.90 | 39.01 |
| 2vkn | 1.73 | 1.34 | 2.25 | 15.75 | 15.43 | 1.58 | 15.40 | 15.45 | 3.22 | 5.62 | 15.59 | 15.37 | 1.65 | 15.23 | 15.63 | 3.19 | 15.78 | 2.36 | 15.70 | 3.99 |
| 2vr3 | 1.37 | 30.45 | 25.32 | 28.10 | 1.61 | 39.51 | 23.69 | 32.46 | 23.79 | 30.38 | 32.05 | 30.25 | 36.75 | 35.47 | 39.24 | 32.29 | 33.86 | 27.25 | 31.46 | 39.42 |
| 2vwf | 0.93 | 1.93 | 3.26 | 21.85 | 2.68 | 22.68 | 20.10 | 21.75 | 17.13 | 22.06 | 21.57 | 22.52 | 11.34 | 24.67 | 20.57 | 11.24 | 2.23 | 21.43 | 4.90 | 21.61 |
| 2w2u | 26.60 | 10.03 | 27.32 | 26.26 | 26.62 | 25.66 | 26.95 | 22.96 | 22.80 | 27.73 | 26.32 | 21.77 | 24.88 | 26.30 | 15.39 | 27.03 | 15.29 | 21.03 | 22.03 | 26.85 |
| 2whx | 1.10 | 3.85 | 6.95 | 2.81 | 26.57 | 7.03 | 4.27 | 6.91 | 24.61 | 7.41 | 22.79 | 25.44 | 26.20 | 26.00 | 29.20 | 2.31 | 21.11 | 25.87 | 23.42 | 23.13 |
| 2xrw | 1.21 | 2.63 | 1.22 | 4.24 | 40.73 | 38.47 | 24.14 | 1.76 | 38.21 | 32.37 | 23.47 | 47.09 | 23.79 | 39.11 | 49.37 | 31.21 | 39.73 | 23.15 | 32.43 | 46.37 |
| 2xu7 | 1.15 | 2.66 | 6.23 | 37.89 | 2.66 | 4.13 | 4.72 | 7.36 | 5.09 | 6.23 | 16.96 | 20.01 | 39.06 | 4.58 | 38.47 | 16.87 | 28.12 | 6.06 | 11.18 | 4.99 |
| 2xvc | 3.75 | 2.37 | 21.17 | 25.18 | 20.86 | 18.85 | 24.09 | 21.43 | 6.63 | 24.74 | 23.83 | 24.61 | 19.85 | 28.26 | 28.79 | 21.00 | 34.49 | 23.10 | 28.98 | 21.19 |
| 2zjd | 0.64 | 28.03 | 29.32 | 28.24 | 31.69 | 29.20 | 32.24 | 33.87 | 27.49 | 32.74 | 33.19 | 31.17 | 28.03 | 32.30 | 29.41 | 34.00 | 2.32 | 30.67 | 34.45 | 33.08 |
| 3asl | 17.88 | 9.01 | 9.81 | 13.76 | 13.56 | 10.29 | 9.36 | 22.02 | 14.99 | 14.19 | 17.53 | 13.28 | 8.72 | 12.74 | 14.69 | 23.35 | 27.00 | 21.13 | 9.29 | 14.88 |
| 3awr | 15.25 | 15.73 | 15.60 | 14.85 | 25.11 | 24.23 | 15.45 | 18.24 | 24.68 | 11.17 | 14.99 | 25.68 | 14.27 | 24.46 | 15.76 | 15.79 | 15.47 | 14.82 | 15.76 | 23.88 |
| 3ayu | 1.20 | 1.38 | 1.09 | 2.54 | 4.38 | 3.61 | 17.41 | 23.34 | 2.44 | 8.70 | 8.44 | 2.76 | 8.26 | 1.78 | 6.79 | 10.43 | 6.38 | 1.66 | 4.12 | 20.99 |
| 3bfq | 0.88 | 0.95 | 27.98 | 15.79 | 15.89 | 14.24 | 9.26 | 0.88 | 15.08 | 27.54 | 5.74 | 15.28 | 27.97 | 5.24 | 40.28 | 33.00 | 27.61 | 17.69 | 5.19 | 20.17 |
| 3c3r | 38.63 | 37.67 | 37.57 | 0.80 | 42.64 | 38.11 | 38.29 | 38.96 | 38.18 | 39.49 | 39.85 | 39.84 | 39.74 | 38.38 | 36.69 | 36.48 | 38.14 | 38.54 | 37.69 | 40.09 |
| 3d32 | 1.19 | 2.75 | 17.58 | 18.62 | 19.29 | 25.66 | 28.62 | 1.71 | 17.00 | 19.35 | 19.35 | 23.54 | 16.31 | 21.91 | 18.06 | 24.66 | 21.88 | 16.63 | 32.56 | 17.28 |
| 3ds4 | 1.73 | 2.29 | 25.08 | 1.08 | 2.21 | 25.20 | 1.40 | 2.07 | 11.06 | 10.94 | 3.20 | 24.59 | 24.80 | 3.49 | 23.25 | 25.50 | 25.45 | 24.39 | 1.41 | 25.81 |
| 3ery | 2.36 | 2.85 | 1.26 | 1.15 | 2.38 | 2.63 | 3.36 | 5.72 | 3.33 | 3.96 | 6.87 | 5.20 | 2.39 | 8.56 | 5.78 | 9.00 | 15.73 | 6.85 | 1.82 | 11.84 |
| 3fdo | 0.75 | 1.98 | 10.43 | 14.91 | 15.30 | 10.59 | 13.10 | 1.69 | 10.17 | 2.07 | 11.37 | 11.68 | 6.85 | 10.93 | 11.45 | 12.41 | 12.00 | 4.35 | 4.80 | 6.07 |
| 3g2s | 0.45 | 1.96 | 3.29 | 18.75 | 19.78 | 30.93 | 2.04 | 2.30 | 19.41 | 2.11 | 1.38 | 4.76 | 4.53 | 21.40 | 3.14 | 2.51 | 2.31 | 12.48 | 17.90 | 32.03 |
| 3gyt | 1.23 | 1.32 | 1.20 | 1.52 | 9.30 | 45.80 | 44.88 | 45.18 | 1.18 | 4.46 | 45.36 | 43.68 | 44.34 | 42.34 | 45.66 | 44.78 | 45.14 | 43.34 | 1.53 | 45.63 |
| 3h1z | 1.19 | 17.71 | 20.01 | 17.13 | 6.64 | 22.59 | 21.34 | 27.51 | 17.17 | 3.26 | 20.03 | 5.69 | 9.68 | 9.98 | 20.87 | 21.49 | 55.56 | 38.50 | 55.59 | 22.12 |
| 3i5r | 0.56 | 2.19 | 16.24 | 15.69 | 2.64 | 15.69 | 16.14 | 16.46 | 2.55 | 3.69 | 15.51 | 15.72 | 1.46 | 15.67 | 10.85 | 15.63 | 16.19 | 10.49 | 9.94 | 15.48 |
| 3ivv | 15.24 | 17.40 | 1.09 | 16.87 | 13.26 | 16.26 | 17.93 | 15.64 | 16.48 | 13.10 | 13.45 | 16.24 | 13.76 | 11.42 | 16.12 | 2.71 | 17.35 | 15.31 | 18.69 | 15.40 |
| 3kmr | 0.94 | 35.47 | 33.76 | 34.98 | 1.99 | 1.70 | 37.24 | 36.14 | 35.60 | 36.98 | 2.03 | 37.28 | 37.37 | 35.91 | 27.30 | 35.75 | 35.93 | 1.65 | 36.44 | 36.05 |
| 3kuj | 22.14 | 22.17 | 19.92 | 21.96 | 16.96 | 19.78 | 21.14 | 19.04 | 20.34 | 20.19 | 19.85 | 23.79 | 22.80 | 16.65 | 18.03 | 18.90 | 21.70 | 17.90 | 27.59 | 23.72 |
| 3kus | 1.57 | 11.72 | 20.36 | 20.03 | 20.53 | 3.57 | 2.48 | 21.07 | 20.58 | 20.13 | 9.90 | 3.31 | 20.82 | 21.16 | 19.98 | 20.43 | 20.59 | 7.76 | 20.09 | 10.36 |
| 3l0e | 26.24 | 26.06 | 2.55 | 1.56 | 39.88 | 1.63 | 27.19 | 26.10 | 25.94 | 26.20 | 26.47 | 32.45 | 33.33 | 26.44 | 37.74 | 32.83 | 30.08 | 33.29 | 29.43 | 28.08 |
| 3ll8 | 1.28 | 1.67 | 3.96 | 39.63 | 40.90 | 39.28 | 48.15 | 34.12 | 38.14 | 36.57 | 34.47 | 38.37 | 38.28 | 48.25 | 35.51 | 34.74 | 39.74 | 40.74 | 35.52 | 13.22 |
| 3llz | 32.54 | 33.79 | 28.06 | 33.84 | 25.91 | 24.50 | 33.18 | 33.76 | 32.58 | 28.20 | 27.21 | 35.00 | 33.54 | 35.85 | 32.96 | 35.09 | 31.39 | 24.98 | 34.26 | 35.49 |
| 3obq | 16.31 | 21.73 | 21.85 | 22.03 | 1.03 | 6.41 | 17.26 | 21.89 | 15.27 | 20.89 | 21.91 | 19.07 | 17.81 | 17.18 | 22.49 | 17.38 | 21.34 | 21.53 | 17.30 | 2.62 |
| 3olf | 25.53 | 25.14 | 23.69 | 22.92 | 23.20 | 23.40 | 24.58 | 24.21 | 23.38 | 26.15 | 24.18 | 23.85 | 1.25 | 16.69 | 24.19 | 24.20 | 22.85 | 23.22 | 22.93 | 23.67 |
| 3p72 | 5.77 | 5.59 | 5.85 | 41.71 | 1.41 | 7.08 | 41.24 | 40.80 | 39.95 | 5.77 | 7.52 | 40.80 | 39.58 | 6.08 | 41.80 | 39.97 | 8.44 | 6.45 | 40.30 | 9.23 |
| 3p8f | 4.53 | 8.39 | 9.04 | 6.85 | 13.10 | 9.81 | 8.00 | 9.09 | 1.54 | 9.71 | 8.35 | 12.85 | 11.58 | 1.50 | 1.44 | 7.12 | 2.15 | 13.22 | 11.84 | 9.08 |
| 3ptl | 12.28 | 13.18 | 17.72 | 18.40 | 27.75 | 30.17 | 12.88 | 27.46 | 16.30 | 32.21 | 17.94 | 17.07 | 17.20 | 17.90 | 27.50 | 18.02 | 28.07 | 48.32 | 27.51 | 17.90 |
| 3qis | 0.97 | 1.65 | 1.58 | 13.90 | 1.59 | 14.24 | 5.55 | 13.95 | 4.26 | 14.62 | 13.32 | 14.45 | 14.08 | 13.95 | 2.21 | 1.02 | 13.51 | 5.26 | 14.12 | 14.68 |
| 3rm1 | 2.10 | 18.02 | 1.47 | 1.06 | 20.20 | 17.45 | 18.28 | 14.21 | 17.04 | 19.58 | 15.55 | 3.16 | 13.84 | 17.85 | 16.43 | 16.54 | 3.20 | 18.40 | 20.24 | 15.83 |
| 3rqg | 41.22 | 23.94 | 23.95 | 44.14 | 45.46 | 40.26 | 36.75 | 39.42 | 27.92 | 31.95 | 28.46 | 44.74 | 24.58 | 30.90 | 40.46 | 23.37 | 29.16 | 22.98 | 37.84 | 48.18 |
| 3sfj | 3.54 | 3.27 | 20.48 | 4.04 | 5.25 | 1.29 | 14.87 | 5.10 | 1.29 | 1.59 | 14.65 | 18.97 | 19.77 | 30.54 | 25.35 | 23.91 | 24.42 | 28.39 | 30.57 | 31.51 |
| 3so6 | 1.10 | 1.99 | 2.67 | 2.18 | 2.02 | 4.07 | 2.66 | 2.16 | 14.69 | 2.97 | 15.66 | 10.59 | 5.29 | 15.27 | 3.74 | 4.31 | 5.52 | 3.73 | 13.73 | 12.88 |
| 3tjv | 1.23 | 1.52 | 2.35 | 2.13 | 1.98 | 19.74 | 11.61 | 12.21 | 7.51 | 25.66 | 25.34 | 15.22 | 2.88 | 2.75 | 25.82 | 23.83 | 3.33 | 22.83 | 24.02 | 12.88 |
| 3tzy | 47.28 | 9.68 | 47.12 | 47.05 | 47.25 | 47.63 | 47.80 | 46.34 | 48.29 | 47.98 | 47.21 | 13.39 | 47.18 | 46.93 | 46.61 | 46.58 | 9.31 | 47.16 | 47.32 | 10.36 |
| 3u9q | 43.32 | 42.74 | 44.10 | 45.97 | 44.71 | 43.76 | 42.41 | 42.58 | 42.66 | 42.66 | 46.82 | 44.35 | 44.19 | 44.55 | 41.92 | 44.75 | 42.43 | 45.38 | 42.02 | 44.62 |
| 3up3 | 1.01 | 1.32 | 3.42 | 1.62 | 5.41 | 1.87 | 5.55 | 4.72 | 2.92 | 3.18 | 5.23 | 16.86 | 5.24 | 15.11 | 6.41 | 15.19 | 9.75 | 4.65 | 1.69 | 6.30 |
| 3v2x | 1.26 | 0.87 | 2.93 | 30.63 | 8.93 | 30.94 | 25.60 | 17.71 | 4.44 | 28.52 | 22.84 | 16.90 | 30.92 | 25.86 | 31.22 | 25.57 | 31.65 | 1.82 | 11.73 | 9.61 |
| 3vtc | 22.72 | 23.26 | 25.02 | 22.90 | 24.17 | 25.78 | 24.29 | 23.75 | 22.04 | 25.90 | 23.44 | 23.71 | 23.96 | 26.36 | 23.32 | 23.08 | 22.75 | 23.05 | 27.08 | 23.30 |
| 3w1b | 83.70 | 41.44 | 40.31 | 38.59 | 79.46 | 40.01 | 41.59 | 40.24 | 40.84 | 41.71 | 80.65 | 39.84 | 79.74 | 39.77 | 80.70 | 80.50 | 40.51 | 41.68 | 1.07 | 2.03 |
| 3zqh | 33.13 | 31.07 | 37.63 | 18.97 | 36.50 | 33.62 | 19.72 | 20.82 | 20.38 | 29.98 | 25.18 | 27.57 | 35.16 | 30.37 | 32.85 | 22.81 | 20.81 | 32.98 | 28.62 | 27.88 |
| 4b4n | 26.19 | 31.81 | 30.42 | 30.13 | 27.62 | 28.46 | 27.69 | 27.41 | 31.33 | 1.41 | 28.22 | 30.48 | 24.10 | 27.26 | 26.09 | 26.90 | 28.77 | 30.34 | 26.38 | 29.66 |
| 4dcb | 42.77 | 44.35 | 43.29 | 40.20 | 44.30 | 47.63 | 43.19 | 44.04 | 40.73 | 45.36 | 43.78 | 44.20 | 40.65 | 40.45 | 42.32 | 41.27 | 47.44 | 57.31 | 43.17 | 45.57 |
| 4e34 | 18.33 | 21.40 | 21.88 | 19.46 | 20.64 | 15.12 | 18.19 | 19.69 | 19.20 | 17.15 | 27.32 | 24.96 | 21.80 | 22.86 | 21.46 | 16.61 | 17.41 | 22.70 | 24.95 | 21.12 |
| 4eik | 1.13 | 2.05 | 16.39 | 17.22 | 16.37 | 16.78 | 10.45 | 16.34 | 16.36 | 1.53 | 16.64 | 16.64 | 16.48 | 16.33 | 17.08 | 3.93 | 16.45 | 3.78 | 16.40 | 2.43 |
| 4ery | 10.27 | 2.92 | 9.56 | 1.75 | 1.27 | 12.03 | 10.27 | 12.22 | 7.31 | 10.79 | 7.76 | 10.75 | 10.63 | 1.68 | 12.61 | 12.11 | 8.49 | 11.15 | 10.08 | 11.06 |
| 4f14 | 11.01 | 20.17 | 10.98 | 21.45 | 18.57 | 20.38 | 20.73 | 9.85 | 18.47 | 18.39 | 22.35 | 11.73 | 24.59 | 12.74 | 1.13 | 10.45 | 23.14 | 22.38 | 18.42 | 9.88 |
| 4f1z | 2.46 | 2.06 | 27.50 | 1.73 | 2.58 | 51.63 | 9.03 | 29.38 | 40.61 | 33.79 | 36.62 | 39.19 | 3.40 | 40.51 | 28.99 | 9.42 | 29.59 | 26.64 | 40.10 | 28.08 |
| 4gq6 | 13.42 | 13.96 | 5.99 | 13.62 | 13.45 | 9.66 | 13.36 | 13.12 | 2.80 | 4.82 | 12.47 | 13.22 | 8.99 | 5.98 | 12.29 | 4.57 | 8.84 | 8.30 | 10.99 | 4.42 |
| 4gxl | 27.92 | 29.40 | 28.96 | 29.44 | 27.99 | 28.38 | 29.26 | 29.51 | 13.88 | 15.22 | 27.32 | 29.90 | 29.03 | 28.49 | 28.27 | 27.67 | 28.93 | 29.45 | 25.49 | 27.56 |
| 4gyw | 22.40 | 47.81 | 44.26 | 22.77 | 22.31 | 44.20 | 22.19 | 5.52 | 45.32 | 31.26 | 43.60 | 21.37 | 42.15 | 42.18 | 42.46 | 43.69 | 42.59 | 51.50 | 44.99 | 21.56 |
| 4h4f | 16.55 | 12.31 | 11.99 | 12.16 | 12.55 | 8.79 | 8.84 | 33.26 | 9.84 | 12.53 | 8.90 | 14.19 | 30.37 | 12.87 | 13.30 | 13.56 | 12.54 | 30.47 | 10.67 | 13.24 |
| 4hom | 15.00 | 11.29 | 13.31 | 42.85 | 42.82 | 15.77 | 42.65 | 11.15 | 8.91 | 11.43 | 23.82 | 33.12 | 33.49 | 14.89 | 36.10 | 8.68 | 43.23 | 42.62 | 22.11 | 32.67 |
| 4htp | 36.43 | 37.55 | 36.97 | 36.99 | 36.00 | 0.88 | 34.59 | 33.61 | 37.31 | 1.65 | 35.02 | 34.69 | 40.32 | 33.76 | 35.67 | 38.46 | 34.73 | 36.54 | 34.77 | 1.92 |
| 4iim | 27.26 | 25.99 | 25.20 | 25.46 | 12.16 | 20.57 | 21.34 | 20.99 | 25.76 | 24.63 | 25.32 | 27.00 | 20.61 | 25.17 | 26.80 | 1.19 | 12.24 | 26.51 | 21.54 | 22.00 |
| 4j8s | 2.31 | 1.96 | 1.28 | 2.41 | 1.73 | 8.88 | 3.33 | 11.44 | 12.52 | 30.73 | 11.08 | 27.78 | 9.30 | 31.27 | 11.57 | 1.99 | 4.84 | 12.57 | 30.76 | 12.10 |
| 4k0u | 1.21 | 2.44 | 1.94 | 2.44 | 1.47 | 1.77 | 3.74 | 24.53 | 26.13 | 7.90 | 5.18 | 22.67 | 5.18 | 6.71 | 6.70 | 16.25 | 22.42 | 23.33 | 28.65 | 13.68 |

**S4(d). L-RMSD values of all 20 poses obtained after blind docking by PatchDock on 133 protein-peptide complexes.**

| **ID** | **Pose1** | **Pose2** | **Pose3** | **Pose4** | **Pose5** | **Pose6** | **Pose7** | **Pose8** | **Pose9** | **Pose10** | **Pose11** | **Pose12** | **Pose13** | **Pose14** | **Pose15** | **Pose16** | **Pose17** | **Pose18** | **Pose19** | **Pose20** |
| --- | --- | --- | --- | --- | --- | --- | --- | --- | --- | --- | --- | --- | --- | --- | --- | --- | --- | --- | --- | --- |
| 1cjr | 2.44 | 18.81 | 6.97 | 7.38 | 12.95 | 7.77 | 16.56 | 10.18 | 19.85 | 16.16 | 10.14 | 9.36 | 17.63 | 10.59 | 13.03 | 10.87 | 16.33 | 12.14 | 13.33 | 13.38 |
| 1cka | 14.43 | 10.71 | 22.12 | 23.35 | 17.61 | 16.34 | 10.52 | 18.23 | 19.53 | 19.39 | 20.85 | 19.20 | 24.66 | 14.07 | 12.10 | 21.63 | 24.36 | 21.97 | 22.95 | 13.25 |
| 1cvu | 36.37 | 35.87 | 35.84 | 37.20 | 35.89 | 35.69 | 34.47 | 48.64 | 46.78 | 37.12 | 48.45 | 50.40 | 48.75 | 35.67 | 35.23 | 36.14 | 35.19 | 50.00 | 45.31 | 48.76 |
| 1d4t | 1.52 | 6.15 | 4.63 | 8.49 | 18.24 | 20.81 | 19.24 | 5.98 | 18.89 | 8.71 | 22.14 | 10.88 | 25.98 | 23.72 | 8.75 | 12.95 | 8.36 | 16.36 | 17.30 | 4.09 |
| 1eg4 | 43.37 | 40.63 | 43.97 | 31.28 | 47.02 | 37.34 | 26.83 | 29.30 | 28.16 | 38.91 | 39.77 | 25.92 | 37.76 | 38.92 | 41.20 | 47.47 | 33.71 | 44.27 | 36.14 | 39.76 |
| 1h6w | 0.98 | 36.48 | 17.22 | 18.33 | 17.65 | 8.94 | 17.53 | 3.57 | 11.78 | 5.09 | 11.09 | 4.48 | 4.46 | 21.59 | 11.96 | 11.05 | 17.50 | 19.99 | 8.63 | 18.87 |
| 1hc9 | 1.65 | 4.43 | 20.95 | 6.30 | 22.41 | 11.23 | 11.59 | 19.09 | 4.88 | 10.16 | 10.66 | 8.90 | 11.16 | 6.50 | 10.54 | 22.25 | 11.16 | 11.42 | 10.44 | 20.23 |
| 1jbu | 34.78 | 16.24 | 21.54 | 39.86 | 23.53 | 34.40 | 38.28 | 34.39 | 15.74 | 32.87 | 20.87 | 18.52 | 24.66 | 40.30 | 41.45 | 32.71 | 40.91 | 31.02 | 41.98 | 17.68 |
| 1k5n | 14.92 | 6.58 | 24.57 | 26.46 | 14.65 | 4.79 | 14.73 | 27.04 | 15.98 | 28.45 | 44.12 | 16.08 | 21.50 | 7.06 | 25.18 | 29.86 | 5.07 | 14.23 | 17.41 | 30.75 |
| 1mfg | 15.78 | 17.18 | 9.17 | 14.75 | 6.14 | 6.41 | 20.57 | 16.52 | 6.62 | 12.88 | 16.46 | 15.50 | 22.53 | 19.87 | 24.29 | 16.72 | 21.43 | 15.39 | 16.18 | 11.39 |
| 1nln | 15.68 | 20.32 | 31.24 | 20.65 | 30.17 | 30.65 | 25.59 | 24.91 | 35.72 | 30.56 | 28.85 | 24.27 | 33.71 | 28.04 | 29.80 | 23.82 | 32.67 | 32.21 | 26.46 | 31.51 |
| 1nq7 | 35.43 | 21.64 | 34.84 | 21.45 | 34.76 | 22.28 | 35.29 | 23.04 | 22.70 | 34.55 | 21.24 | 36.03 | 35.05 | 35.60 | 32.82 | 21.16 | 35.46 | 21.33 | 33.48 | 19.89 |
| 1ntv | 27.61 | 1.78 | 32.20 | 29.29 | 28.07 | 13.82 | 27.94 | 32.91 | 26.88 | 25.09 | 28.92 | 30.23 | 28.73 | 25.31 | 25.81 | 28.28 | 16.19 | 19.40 | 27.47 | 32.32 |
| 1nx1 | 27.29 | 32.65 | 34.64 | 28.74 | 35.22 | 25.78 | 31.06 | 34.47 | 27.97 | 32.29 | 30.97 | 28.59 | 33.38 | 33.33 | 5.04 | 32.84 | 25.21 | 33.68 | 26.71 | 35.29 |
| 1oai | 17.86 | 23.55 | 21.43 | 19.38 | 18.70 | 15.24 | 24.95 | 23.60 | 23.06 | 11.47 | 23.79 | 22.63 | 25.68 | 12.28 | 17.94 | 22.15 | 11.09 | 24.36 | 25.43 | 12.30 |
| 1oj5 | 5.17 | 23.31 | 10.45 | 16.16 | 21.34 | 26.02 | 13.43 | 20.93 | 24.96 | 27.34 | 23.30 | 13.54 | 25.79 | 4.53 | 15.60 | 23.87 | 16.58 | 16.41 | 29.04 | 11.74 |
| 1ou8 | 14.09 | 12.24 | 10.86 | 31.27 | 1.08 | 7.70 | 31.14 | 27.20 | 12.35 | 33.93 | 23.62 | 32.47 | 30.77 | 24.04 | 30.69 | 24.72 | 28.88 | 31.17 | 10.39 | 32.18 |
| 1ow6 | 17.75 | 17.34 | 18.44 | 20.12 | 18.70 | 17.94 | 16.89 | 16.42 | 15.18 | 11.83 | 16.07 | 13.78 | 20.20 | 21.02 | 15.73 | 14.69 | 15.44 | 15.01 | 27.47 | 14.78 |
| 1pzl | 24.85 | 21.06 | 28.37 | 25.86 | 21.27 | 27.24 | 28.02 | 24.11 | 30.53 | 26.07 | 30.30 | 25.00 | 6.12 | 19.16 | 32.93 | 28.25 | 24.33 | 27.47 | 26.30 | 45.15 |
| 1qkz | 33.69 | 33.17 | 33.14 | 68.02 | 33.62 | 35.31 | 33.62 | 33.29 | 32.22 | 61.66 | 69.51 | 35.58 | 33.19 | 34.57 | 34.39 | 35.19 | 32.02 | 32.28 | 34.50 | 33.84 |
| 1rst | 8.78 | 8.48 | 9.45 | 7.08 | 7.38 | 8.45 | 8.48 | 3.87 | 35.27 | 10.38 | 8.94 | 6.27 | 8.42 | 7.41 | 5.90 | 8.24 | 5.29 | 35.48 | 7.60 | 6.09 |
| 1rxz | 19.43 | 28.39 | 23.17 | 14.54 | 22.08 | 4.66 | 19.36 | 31.60 | 23.46 | 21.95 | 31.80 | 25.39 | 24.92 | 26.26 | 25.48 | 26.74 | 6.53 | 23.44 | 1.60 | 21.44 |
| 1sfi | 2.52 | 17.12 | 12.56 | 12.26 | 5.73 | 34.61 | 14.31 | 15.07 | 6.54 | 11.40 | 13.97 | 13.30 | 23.07 | 14.86 | 6.78 | 32.41 | 10.39 | 4.25 | 11.64 | 10.33 |
| 1ssh | 19.70 | 8.60 | 25.82 | 11.62 | 17.79 | 25.92 | 23.45 | 18.46 | 28.05 | 16.61 | 13.02 | 12.92 | 18.29 | 20.10 | 11.50 | 25.91 | 8.91 | 15.72 | 17.82 | 12.26 |
| 1t08 | 31.75 | 25.95 | 11.95 | 21.16 | 24.22 | 30.74 | 30.73 | 19.13 | 13.48 | 30.03 | 25.40 | 13.40 | 16.43 | 40.15 | 52.00 | 25.59 | 30.90 | 10.26 | 25.17 | 26.93 |
| 1t4f | 8.23 | 7.75 | 3.07 | 9.31 | 9.67 | 15.90 | 4.39 | 18.83 | 5.82 | 8.13 | 9.14 | 5.66 | 7.69 | 11.31 | 10.53 | 16.90 | 9.10 | 16.00 | 8.94 | 9.33 |
| 1t7r | 27.16 | 38.75 | 35.52 | 34.35 | 27.09 | 38.65 | 8.98 | 25.54 | 15.71 | 29.51 | 15.15 | 39.26 | 18.48 | 1.20 | 28.42 | 37.50 | 35.54 | 28.31 | 19.47 | 35.55 |
| 1tfc | 24.75 | 24.75 | 23.51 | 11.01 | 24.88 | 26.18 | 24.75 | 28.05 | 28.20 | 28.58 | 24.89 | 26.71 | 27.31 | 26.90 | 27.62 | 25.32 | 11.95 | 25.15 | 31.00 | 24.76 |
| 1u00 | 27.18 | 29.55 | 15.84 | 13.83 | 15.15 | 15.40 | 21.82 | 15.46 | 33.67 | 18.75 | 22.89 | 14.21 | 31.02 | 13.18 | 27.62 | 14.53 | 15.29 | 5.48 | 25.26 | 28.88 |
| 1uj0 | 21.48 | 17.42 | 20.26 | 14.55 | 9.88 | 12.86 | 28.01 | 22.23 | 16.46 | 14.28 | 21.84 | 20.82 | 14.30 | 22.38 | 27.47 | 15.49 | 13.42 | 13.99 | 11.12 | 12.50 |
| 1x2r | 1.73 | 5.54 | 30.95 | 8.54 | 6.51 | 31.12 | 7.68 | 9.58 | 31.13 | 7.49 | 32.84 | 6.97 | 30.84 | 7.09 | 32.84 | 29.82 | 6.91 | 5.44 | 8.18 | 31.26 |
| 1xoc | 0.92 | 13.99 | 19.22 | 5.97 | 27.47 | 26.06 | 23.34 | 23.89 | 20.76 | 23.61 | 24.61 | 14.09 | 4.01 | 23.27 | 22.17 | 29.01 | 23.54 | 28.89 | 22.17 | 22.88 |
| 1ymt | 30.34 | 30.53 | 5.98 | 9.90 | 36.54 | 23.17 | 32.26 | 22.58 | 33.49 | 34.04 | 10.80 | 26.79 | 35.38 | 34.79 | 7.65 | 37.61 | 38.85 | 29.31 | 33.99 | 35.45 |
| 1yuc | 21.36 | 20.55 | 19.83 | 29.23 | 17.48 | 14.36 | 30.18 | 28.97 | 23.81 | 32.69 | 19.05 | 7.76 | 29.25 | 17.94 | 32.13 | 21.53 | 22.88 | 18.33 | 19.72 | 20.72 |
| 1ywo | 16.63 | 22.97 | 6.85 | 16.58 | 17.48 | 7.30 | 17.07 | 11.74 | 28.05 | 28.13 | 19.85 | 14.20 | 19.70 | 23.44 | 8.56 | 13.24 | 28.40 | 11.77 | 17.32 | 14.93 |
| 2a25 | 0.46 | 16.36 | 20.24 | 18.98 | 21.78 | 8.73 | 19.43 | 12.21 | 8.65 | 17.14 | 18.40 | 23.07 | 23.24 | 16.83 | 19.20 | 19.36 | 15.59 | 17.04 | 18.97 | 21.88 |
| 2a3i | 28.58 | 10.54 | 31.79 | 13.29 | 21.06 | 39.97 | 25.30 | 10.62 | 28.58 | 35.80 | 34.52 | 10.50 | 7.82 | 9.75 | 8.85 | 29.40 | 6.90 | 38.01 | 37.56 | 33.19 |
| 2aq9 | 19.70 | 31.53 | 29.41 | 20.59 | 25.66 | 31.50 | 30.45 | 34.52 | 35.81 | 27.95 | 32.70 | 19.78 | 35.48 | 18.45 | 35.25 | 35.09 | 37.31 | 34.55 | 19.09 | 13.77 |
| 2b9h | 34.74 | 32.37 | 21.20 | 22.22 | 31.33 | 29.77 | 37.93 | 28.62 | 30.80 | 19.91 | 26.99 | 38.08 | 24.87 | 18.49 | 42.03 | 41.48 | 19.83 | 46.54 | 42.90 | 33.32 |
| 2bba | 17.56 | 1.57 | 12.57 | 13.62 | 13.20 | 28.57 | 30.55 | 11.06 | 14.17 | 14.94 | 27.60 | 5.53 | 13.62 | 15.04 | 28.25 | 6.31 | 15.11 | 15.70 | 28.71 | 12.96 |
| 2cch | 30.36 | 35.00 | 28.72 | 31.34 | 31.33 | 26.16 | 26.42 | 33.91 | 21.81 | 29.13 | 26.59 | 40.05 | 28.42 | 31.09 | 27.86 | 25.36 | 41.62 | 32.89 | 29.00 | 24.61 |
| 2ce8 | 28.67 | 27.89 | 27.54 | 33.78 | 25.96 | 27.44 | 32.79 | 27.73 | 32.69 | 32.18 | 27.83 | 33.90 | 24.49 | 28.98 | 31.37 | 28.18 | 34.19 | 32.37 | 26.65 | 34.56 |
| 2d0n | 18.66 | 18.49 | 22.98 | 20.16 | 14.37 | 19.27 | 17.23 | 21.10 | 24.76 | 17.86 | 24.59 | 20.65 | 16.25 | 22.60 | 20.96 | 10.29 | 15.47 | 18.01 | 19.21 | 22.63 |
| 2drk | 22.72 | 18.31 | 27.62 | 11.83 | 15.94 | 12.37 | 19.12 | 25.98 | 25.62 | 19.50 | 15.87 | 20.73 | 23.26 | 19.53 | 18.35 | 12.23 | 26.20 | 16.56 | 21.94 | 21.34 |
| 2dyp | 19.72 | 15.15 | 30.33 | 16.12 | 15.46 | 19.52 | 30.23 | 3.09 | 7.81 | 10.66 | 29.29 | 23.66 | 16.87 | 17.08 | 6.56 | 23.14 | 21.85 | 34.07 | 27.61 | 29.20 |
| 2fff | 20.98 | 24.93 | 25.69 | 24.29 | 25.08 | 26.40 | 26.01 | 22.52 | 23.91 | 23.79 | 24.12 | 25.06 | 26.12 | 28.27 | 42.75 | 22.54 | 41.72 | 26.17 | 23.55 | 54.78 |
| 2ffu | 15.44 | 29.69 | 6.59 | 14.65 | 10.77 | 11.52 | 14.79 | 9.92 | 32.85 | 26.81 | 32.15 | 19.15 | 34.28 | 47.16 | 32.45 | 21.15 | 36.84 | 15.13 | 50.45 | 33.56 |
| 2fka | 25.06 | 5.17 | 8.62 | 10.00 | 29.25 | 6.47 | 8.33 | 24.11 | 7.61 | 6.94 | 27.37 | 7.99 | 9.76 | 7.54 | 8.39 | 28.94 | 28.04 | 9.77 | 7.50 | 5.34 |
| 2fmf | 11.11 | 7.75 | 12.50 | 29.31 | 19.73 | 20.77 | 6.68 | 6.26 | 29.58 | 11.73 | 25.23 | 27.56 | 12.56 | 31.35 | 22.55 | 24.64 | 10.78 | 13.16 | 13.00 | 20.24 |
| 2fts | 25.07 | 1.41 | 35.26 | 28.47 | 28.77 | 31.00 | 18.62 | 28.34 | 26.82 | 29.49 | 26.58 | 32.23 | 26.88 | 24.26 | 57.93 | 31.50 | 24.26 | 29.51 | 14.47 | 23.42 |
| 2fvj | 27.20 | 29.70 | 27.84 | 25.62 | 21.83 | 26.85 | 25.62 | 8.54 | 28.38 | 27.26 | 22.41 | 5.80 | 28.39 | 10.67 | 18.84 | 37.02 | 17.14 | 29.38 | 37.01 | 25.21 |
| 2ho2 | 30.23 | 23.09 | 29.58 | 21.44 | 18.42 | 23.03 | 20.56 | 24.27 | 27.67 | 14.94 | 19.02 | 18.07 | 20.54 | 20.74 | 18.60 | 17.93 | 15.40 | 18.40 | 27.66 | 15.91 |
| 2ht9 | 28.34 | 23.85 | 21.01 | 22.96 | 29.84 | 23.86 | 30.47 | 19.07 | 30.30 | 21.96 | 32.13 | 22.69 | 11.16 | 23.99 | 28.49 | 28.08 | 9.59 | 22.20 | 28.88 | 21.91 |
| 2o02 | 0.92 | 14.11 | 7.15 | 9.64 | 4.10 | 13.92 | 4.63 | 5.73 | 14.67 | 5.63 | 6.50 | 12.56 | 14.90 | 17.32 | 26.10 | 12.90 | 14.28 | 18.25 | 13.76 | 6.03 |
| 2o4j | 20.90 | 19.76 | 19.84 | 20.54 | 20.65 | 33.71 | 9.32 | 18.56 | 8.68 | 24.68 | 34.29 | 35.30 | 18.23 | 21.14 | 32.87 | 22.73 | 29.57 | 19.86 | 37.79 | 33.53 |
| 2o9v | 30.96 | 21.07 | 27.87 | 28.17 | 18.41 | 28.50 | 16.70 | 22.42 | 27.27 | 23.74 | 17.42 | 23.42 | 19.65 | 23.90 | 25.66 | 28.10 | 18.11 | 20.33 | 27.08 | 21.47 |
| 2oei | 22.61 | 20.92 | 19.31 | 25.04 | 21.38 | 11.07 | 23.33 | 24.74 | 25.86 | 17.65 | 26.70 | 22.96 | 20.96 | 26.03 | 21.26 | 20.09 | 14.22 | 19.79 | 14.50 | 19.24 |
| 2p0w | 17.69 | 14.03 | 24.13 | 26.28 | 17.03 | 19.93 | 14.62 | 8.08 | 11.96 | 33.11 | 21.14 | 15.72 | 13.94 | 11.90 | 18.06 | 29.96 | 34.32 | 15.45 | 32.04 | 22.71 |
| 2p1o | 39.18 | 11.57 | 7.28 | 18.71 | 35.59 | 36.31 | 31.77 | 9.49 | 11.49 | 29.98 | 12.02 | 12.77 | 3.11 | 13.24 | 35.89 | 8.46 | 9.95 | 22.98 | 13.57 | 16.69 |
| 2p1t | 20.71 | 23.01 | 21.41 | 24.35 | 24.34 | 10.70 | 21.52 | 22.99 | 18.66 | 19.21 | 28.20 | 23.72 | 9.15 | 20.10 | 13.63 | 30.32 | 21.47 | 23.06 | 22.38 | 23.67 |
| 2p54 | 21.88 | 26.19 | 1.87 | 24.00 | 25.18 | 23.31 | 23.90 | 25.77 | 32.24 | 26.17 | 21.81 | 30.31 | 42.18 | 21.61 | 22.80 | 20.98 | 37.24 | 21.04 | 12.06 | 23.62 |
| 2peh | 25.97 | 28.08 | 24.64 | 24.79 | 29.07 | 31.21 | 26.25 | 33.12 | 32.32 | 26.43 | 31.25 | 22.71 | 25.62 | 23.42 | 8.74 | 24.63 | 27.32 | 27.19 | 29.53 | 27.00 |
| 2pux | 44.32 | 33.58 | 21.91 | 20.21 | 28.75 | 29.66 | 25.72 | 29.63 | 24.87 | 21.53 | 14.43 | 33.50 | 27.96 | 28.43 | 24.91 | 28.05 | 31.25 | 39.46 | 39.80 | 30.19 |
| 2puy | 24.62 | 21.37 | 21.52 | 7.48 | 26.77 | 21.02 | 12.25 | 13.61 | 24.83 | 9.24 | 16.81 | 26.93 | 23.87 | 15.03 | 16.16 | 20.15 | 20.30 | 22.00 | 20.29 | 24.42 |
| 2qbx | 1.48 | 6.43 | 35.03 | 16.01 | 17.30 | 17.55 | 28.61 | 30.09 | 8.28 | 21.14 | 14.79 | 3.34 | 15.01 | 19.61 | 36.25 | 10.81 | 17.40 | 16.08 | 31.29 | 31.29 |
| 2qos | 9.84 | 9.16 | 5.53 | 6.99 | 1.41 | 10.38 | 4.12 | 8.34 | 6.97 | 8.49 | 10.44 | 5.92 | 9.35 | 3.88 | 5.10 | 7.03 | 9.32 | 11.24 | 9.47 | 11.42 |
| 2qse | 4.58 | 11.58 | 32.53 | 11.32 | 30.19 | 31.83 | 11.52 | 18.18 | 25.93 | 24.53 | 24.89 | 11.43 | 23.86 | 31.10 | 27.67 | 44.27 | 28.20 | 27.71 | 35.20 | 25.91 |
| 2r7g | 4.80 | 9.43 | 4.78 | 11.35 | 10.47 | 6.02 | 2.89 | 11.09 | 9.34 | 10.65 | 6.79 | 5.36 | 4.23 | 11.17 | 5.19 | 7.33 | 31.14 | 10.30 | 30.02 | 10.54 |
| 2r9q | 9.06 | 15.00 | 11.82 | 26.62 | 27.11 | 13.93 | 29.06 | 2.23 | 14.08 | 6.30 | 25.91 | 13.58 | 23.10 | 12.97 | 11.17 | 19.17 | 8.10 | 26.49 | 28.22 | 9.57 |
| 2v8y | 37.13 | 36.49 | 1.11 | 36.85 | 34.36 | 38.45 | 35.01 | 13.42 | 36.49 | 36.50 | 39.32 | 38.62 | 38.43 | 27.26 | 25.81 | 35.27 | 23.36 | 38.99 | 36.46 | 32.17 |
| 2vkn | 25.61 | 23.04 | 23.57 | 21.49 | 20.80 | 24.11 | 26.59 | 11.18 | 23.94 | 21.68 | 25.93 | 27.62 | 28.64 | 22.20 | 30.29 | 21.74 | 24.85 | 28.50 | 25.89 | 19.75 |
| 2vr3 | 0.82 | 23.34 | 27.25 | 24.20 | 23.62 | 24.15 | 23.46 | 22.89 | 32.77 | 19.00 | 19.82 | 27.03 | 10.60 | 17.88 | 14.62 | 23.47 | 11.35 | 17.19 | 30.49 | 19.05 |
| 2vwf | 14.67 | 26.70 | 19.11 | 24.46 | 16.69 | 15.94 | 14.84 | 18.51 | 25.16 | 26.55 | 26.97 | 24.80 | 20.70 | 28.93 | 17.86 | 18.00 | 26.21 | 10.65 | 23.66 | 26.92 |
| 2w2u | 18.64 | 15.84 | 15.08 | 17.61 | 6.30 | 23.14 | 8.22 | 7.41 | 17.26 | 22.42 | 22.16 | 9.98 | 15.26 | 8.08 | 22.01 | 8.80 | 25.92 | 9.43 | 16.79 | 7.26 |
| 2whx | 24.07 | 27.69 | 31.17 | 63.02 | 29.13 | 36.88 | 62.42 | 24.08 | 37.18 | 39.62 | 22.41 | 34.20 | 62.77 | 31.37 | 24.23 | 64.94 | 69.72 | 61.90 | 33.20 | 56.02 |
| 2xrw | 34.68 | 36.40 | 23.51 | 28.34 | 31.65 | 34.23 | 19.58 | 26.52 | 37.29 | 36.24 | 29.19 | 35.74 | 38.71 | 29.30 | 40.01 | 38.56 | 25.97 | 22.30 | 29.33 | 35.85 |
| 2xu7 | 29.58 | 27.33 | 19.71 | 14.39 | 38.58 | 26.55 | 9.97 | 28.37 | 32.93 | 21.08 | 35.75 | 29.52 | 32.37 | 18.60 | 12.32 | 5.92 | 33.81 | 39.88 | 13.84 | 11.99 |
| 2xvc | 22.52 | 20.94 | 21.97 | 12.46 | 22.90 | 21.68 | 21.10 | 2.08 | 7.67 | 10.48 | 3.82 | 24.04 | 21.27 | 22.65 | 21.72 | 21.07 | 8.93 | 21.57 | 19.08 | 22.26 |
| 2zjd | 19.26 | 20.16 | 21.00 | 22.06 | 18.70 | 23.00 | 20.19 | 21.90 | 21.89 | 21.17 | 20.72 | 20.89 | 23.67 | 22.78 | 2.33 | 24.40 | 23.23 | 23.72 | 21.72 | 15.52 |
| 3asl | 19.90 | 19.28 | 18.32 | 22.36 | 17.96 | 8.52 | 17.58 | 22.56 | 22.94 | 11.61 | 18.14 | 18.07 | 18.86 | 20.85 | 15.07 | 16.64 | 18.84 | 24.21 | 10.71 | 16.96 |
| 3awr | 15.10 | 14.54 | 12.14 | 15.49 | 14.45 | 11.99 | 13.45 | 15.12 | 18.82 | 17.82 | 12.37 | 12.31 | 12.97 | 11.78 | 12.30 | 13.55 | 22.67 | 20.66 | 10.92 | 20.85 |
| 3ayu | 15.97 | 0.85 | 19.24 | 18.11 | 26.93 | 20.17 | 15.72 | 27.57 | 6.09 | 15.77 | 16.02 | 3.76 | 25.13 | 24.94 | 27.97 | 5.75 | 25.73 | 16.23 | 25.88 | 25.66 |
| 3bfq | 1.13 | 28.13 | 27.57 | 29.18 | 9.79 | 27.96 | 3.97 | 16.30 | 27.68 | 27.57 | 29.95 | 28.34 | 7.59 | 34.37 | 27.72 | 29.08 | 28.37 | 31.49 | 29.42 | 10.57 |
| 3c3r | 22.52 | 25.06 | 22.55 | 23.86 | 24.29 | 23.31 | 23.98 | 24.91 | 41.16 | 21.79 | 17.98 | 23.00 | 28.44 | 35.26 | 23.60 | 19.04 | 24.97 | 22.40 | 21.22 | 23.01 |
| 3d32 | 0.85 | 22.01 | 12.23 | 11.01 | 25.30 | 29.40 | 16.44 | 20.82 | 23.74 | 24.90 | 30.15 | 26.67 | 22.64 | 19.61 | 23.80 | 20.41 | 29.88 | 22.77 | 28.58 | 22.68 |
| 3ds4 | 1.17 | 7.65 | 10.43 | 7.92 | 9.62 | 6.08 | 10.70 | 12.68 | 11.97 | 7.77 | 11.48 | 12.40 | 8.40 | 9.18 | 10.95 | 6.03 | 11.67 | 10.44 | 9.39 | 11.19 |
| 3ery | 3.07 | 15.29 | 9.86 | 7.89 | 27.05 | 4.02 | 5.85 | 7.48 | 15.93 | 19.95 | 14.43 | 14.76 | 8.84 | 12.62 | 29.36 | 28.62 | 26.82 | 19.31 | 20.59 | 15.72 |
| 3fdo | 12.82 | 12.47 | 18.74 | 11.10 | 6.13 | 12.84 | 3.74 | 13.01 | 17.45 | 18.34 | 11.83 | 7.91 | 4.78 | 20.88 | 1.56 | 11.63 | 12.08 | 6.57 | 17.76 | 12.26 |
| 3g2s | 39.06 | 38.89 | 36.67 | 25.89 | 8.26 | 11.18 | 18.09 | 19.67 | 37.07 | 38.09 | 34.05 | 40.60 | 26.35 | 34.96 | 23.59 | 21.80 | 20.12 | 41.01 | 24.11 | 28.51 |
| 3gyt | 28.00 | 29.49 | 27.82 | 19.48 | 29.55 | 7.72 | 18.64 | 28.84 | 18.12 | 29.29 | 29.45 | 27.78 | 27.71 | 29.83 | 8.30 | 26.52 | 21.02 | 22.13 | 22.28 | 22.95 |
| 3h1z | 38.91 | 31.44 | 32.61 | 39.57 | 53.23 | 40.79 | 28.52 | 11.28 | 28.24 | 53.49 | 22.40 | 33.90 | 25.41 | 25.79 | 24.95 | 39.26 | 25.31 | 29.24 | 30.42 | 33.49 |
| 3i5r | 10.54 | 16.68 | 22.58 | 11.22 | 4.23 | 13.13 | 19.63 | 26.73 | 8.39 | 23.37 | 20.99 | 17.34 | 16.90 | 19.07 | 20.13 | 4.64 | 28.70 | 11.10 | 9.03 | 20.23 |
| 3ivv | 27.06 | 28.49 | 4.70 | 26.67 | 31.03 | 24.73 | 27.42 | 34.50 | 25.18 | 15.73 | 32.80 | 20.36 | 27.53 | 26.20 | 22.09 | 13.68 | 28.08 | 20.89 | 27.20 | 20.29 |
| 3kmr | 23.15 | 22.02 | 22.86 | 36.08 | 23.10 | 22.94 | 23.95 | 22.53 | 34.94 | 26.78 | 21.72 | 32.47 | 24.49 | 31.57 | 22.64 | 23.67 | 21.09 | 21.08 | 26.24 | 22.73 |
| 3kuj | 22.46 | 20.01 | 8.78 | 26.00 | 25.73 | 22.17 | 19.58 | 21.79 | 22.24 | 21.35 | 23.93 | 21.38 | 21.30 | 21.44 | 20.49 | 22.20 | 21.03 | 22.05 | 23.60 | 21.15 |
| 3kus | 23.99 | 22.37 | 17.36 | 22.75 | 15.96 | 16.29 | 8.95 | 15.47 | 8.16 | 20.68 | 19.40 | 13.16 | 19.65 | 20.60 | 15.54 | 17.77 | 17.68 | 18.70 | 9.27 | 4.53 |
| 3l0e | 32.05 | 20.60 | 34.86 | 21.62 | 7.42 | 33.37 | 30.10 | 12.52 | 18.19 | 33.68 | 20.49 | 31.59 | 34.29 | 35.17 | 38.49 | 33.47 | 33.02 | 12.25 | 21.48 | 11.86 |
| 3ll8 | 40.90 | 25.49 | 29.79 | 38.11 | 39.25 | 33.17 | 25.72 | 0.62 | 44.30 | 34.25 | 25.81 | 34.15 | 34.86 | 37.11 | 33.11 | 20.74 | 30.71 | 37.03 | 32.42 | 33.47 |
| 3llz | 30.59 | 25.83 | 23.74 | 18.34 | 26.71 | 25.97 | 32.24 | 21.03 | 25.80 | 23.09 | 25.03 | 21.23 | 29.22 | 22.23 | 27.64 | 31.90 | 30.68 | 26.15 | 23.08 | 20.77 |
| 3obq | 7.51 | 30.72 | 28.68 | 16.67 | 18.83 | 27.76 | 3.98 | 34.74 | 26.77 | 22.76 | 27.94 | 19.47 | 32.31 | 25.89 | 23.15 | 24.57 | 27.49 | 19.14 | 23.26 | 32.88 |
| 3olf | 34.26 | 34.48 | 32.67 | 36.08 | 31.84 | 22.53 | 22.20 | 29.88 | 32.94 | 4.90 | 41.51 | 36.17 | 20.78 | 30.40 | 36.19 | 31.60 | 41.49 | 40.69 | 36.55 | 42.46 |
| 3p72 | 5.50 | 6.92 | 7.58 | 8.00 | 7.54 | 9.81 | 9.23 | 7.33 | 9.48 | 8.86 | 8.89 | 9.15 | 9.38 | 8.71 | 10.09 | 6.76 | 9.83 | 0.95 | 8.15 | 6.87 |
| 3p8f | 1.77 | 12.77 | 12.54 | 2.71 | 15.78 | 8.02 | 13.13 | 13.56 | 12.07 | 5.36 | 11.03 | 11.19 | 9.54 | 10.19 | 11.43 | 12.16 | 4.33 | 13.84 | 13.84 | 13.70 |
| 3ptl | 12.18 | 17.19 | 23.50 | 28.84 | 4.54 | 26.32 | 12.47 | 13.64 | 29.64 | 27.58 | 33.48 | 17.78 | 9.12 | 30.83 | 28.60 | 33.29 | 30.33 | 21.83 | 34.28 | 37.72 |
| 3qis | 35.28 | 44.20 | 29.30 | 31.62 | 34.43 | 32.24 | 32.06 | 40.66 | 34.45 | 35.50 | 44.53 | 31.16 | 39.94 | 13.37 | 30.58 | 15.62 | 14.06 | 32.16 | 10.20 | 39.02 |
| 3rm1 | 17.14 | 16.72 | 6.90 | 3.48 | 9.09 | 19.82 | 10.26 | 17.67 | 10.72 | 19.27 | 4.21 | 9.57 | 5.25 | 11.11 | 20.88 | 19.74 | 16.47 | 17.67 | 3.99 | 5.09 |
| 3rqg | 27.43 | 30.16 | 29.69 | 35.00 | 5.10 | 11.28 | 7.65 | 5.86 | 32.27 | 27.98 | 28.71 | 30.08 | 7.36 | 11.84 | 2.00 | 31.34 | 5.55 | 12.93 | 26.02 | 35.23 |
| 3sfj | 15.46 | 21.41 | 14.14 | 10.71 | 25.79 | 24.18 | 15.46 | 1.65 | 15.46 | 19.37 | 22.27 | 15.69 | 16.35 | 15.75 | 8.58 | 7.05 | 27.25 | 15.25 | 28.61 | 23.37 |
| 3so6 | 0.99 | 22.39 | 9.73 | 19.93 | 3.89 | 22.52 | 30.24 | 19.50 | 32.47 | 31.71 | 30.79 | 3.80 | 30.22 | 31.61 | 26.10 | 31.46 | 34.73 | 33.08 | 19.85 | 34.49 |
| 3tjv | 2.90 | 8.20 | 16.54 | 18.23 | 16.38 | 15.80 | 25.66 | 3.86 | 32.80 | 32.97 | 28.10 | 15.09 | 18.52 | 17.08 | 3.05 | 29.96 | 15.60 | 22.27 | 26.93 | 24.86 |
| 3tzy | 21.75 | 21.12 | 19.87 | 18.85 | 23.65 | 19.19 | 22.46 | 20.39 | 17.09 | 16.69 | 19.61 | 25.68 | 20.26 | 17.71 | 20.02 | 18.81 | 21.70 | 23.94 | 23.38 | 21.39 |
| 3u9q | 26.94 | 20.60 | 8.24 | 25.11 | 3.15 | 34.93 | 7.88 | 24.81 | 25.44 | 30.63 | 4.42 | 34.41 | 28.59 | 23.70 | 21.64 | 25.47 | 27.12 | 21.43 | 25.61 | 21.27 |
| 3up3 | 30.58 | 22.22 | 24.54 | 7.39 | 15.16 | 10.27 | 16.44 | 20.01 | 36.67 | 21.80 | 32.60 | 2.71 | 35.62 | 35.67 | 26.18 | 18.30 | 13.21 | 6.10 | 22.41 | 22.99 |
| 3v2x | 36.70 | 15.40 | 14.39 | 16.08 | 30.34 | 12.07 | 27.10 | 27.92 | 24.88 | 32.93 | 36.75 | 30.70 | 8.10 | 19.84 | 29.77 | 23.13 | 5.95 | 30.21 | 32.59 | 28.23 |
| 3vtc | 20.52 | 20.75 | 19.41 | 19.84 | 11.91 | 17.74 | 12.26 | 32.24 | 32.74 | 16.20 | 18.81 | 9.25 | 19.44 | 15.23 | 26.35 | 1.36 | 34.56 | 33.72 | 37.04 | 13.54 |
| 3w1b | 65.47 | 69.26 | 67.90 | 69.20 | 66.04 | 66.76 | 64.82 | 65.25 | 67.44 | 71.82 | 69.45 | 45.15 | 70.68 | 74.74 | 66.75 | 68.05 | 69.10 | 66.59 | 82.35 | 45.58 |
| 3zqh | 38.24 | 37.54 | 20.07 | 36.12 | 34.25 | 18.49 | 31.23 | 39.27 | 31.50 | 37.34 | 25.51 | 32.76 | 22.90 | 35.02 | 16.11 | 34.99 | 19.64 | 31.26 | 34.77 | 37.95 |
| 4b4n | 11.57 | 32.29 | 31.41 | 10.06 | 13.60 | 10.47 | 9.54 | 32.45 | 29.85 | 30.89 | 32.68 | 32.88 | 35.41 | 32.49 | 25.64 | 9.23 | 34.07 | 32.10 | 32.90 | 10.32 |
| 4dcb | 10.88 | 9.87 | 10.99 | 8.41 | 11.18 | 12.20 | 7.78 | 10.86 | 5.17 | 9.47 | 8.63 | 9.49 | 10.74 | 6.60 | 8.08 | 60.98 | 10.82 | 1.43 | 61.39 | 25.04 |
| 4e34 | 14.34 | 16.93 | 1.94 | 17.95 | 17.11 | 16.69 | 15.19 | 18.08 | 11.53 | 15.61 | 28.39 | 16.18 | 20.92 | 26.86 | 17.12 | 15.62 | 14.03 | 26.28 | 23.80 | 15.05 |
| 4eik | 16.54 | 18.28 | 21.97 | 23.58 | 22.07 | 25.34 | 19.18 | 24.53 | 19.94 | 24.31 | 24.34 | 3.04 | 23.29 | 22.43 | 20.62 | 13.32 | 9.84 | 13.24 | 15.85 | 23.00 |
| 4ery | 28.78 | 32.37 | 31.62 | 33.37 | 10.41 | 31.95 | 31.90 | 31.62 | 8.51 | 35.78 | 37.05 | 32.81 | 6.69 | 11.72 | 31.70 | 33.39 | 6.30 | 31.31 | 30.52 | 33.24 |
| 4f14 | 15.46 | 20.08 | 20.54 | 18.08 | 25.48 | 20.05 | 20.93 | 20.48 | 21.77 | 27.36 | 13.26 | 18.41 | 18.69 | 23.25 | 15.87 | 23.38 | 20.51 | 12.48 | 19.52 | 20.77 |
| 4f1z | 3.00 | 2.62 | 26.20 | 28.83 | 16.63 | 24.84 | 27.38 | 13.65 | 27.08 | 30.43 | 20.52 | 33.21 | 4.38 | 25.29 | 30.32 | 32.90 | 9.03 | 26.87 | 28.92 | 33.56 |
| 4gq6 | 5.07 | 11.78 | 7.72 | 8.72 | 11.47 | 13.49 | 7.93 | 8.93 | 7.62 | 10.11 | 17.17 | 11.76 | 7.87 | 10.33 | 9.31 | 11.18 | 7.75 | 8.92 | 16.21 | 9.27 |
| 4gxl | 28.49 | 30.58 | 26.11 | 25.31 | 34.98 | 31.69 | 30.51 | 28.97 | 31.32 | 27.96 | 30.48 | 31.46 | 23.35 | 28.80 | 24.49 | 26.68 | 26.31 | 29.04 | 30.18 | 25.19 |
| 4gyw | 39.16 | 38.95 | 29.40 | 39.54 | 39.77 | 38.64 | 10.86 | 40.09 | 37.59 | 36.01 | 22.76 | 42.07 | 25.89 | 27.42 | 24.49 | 6.65 | 31.14 | 40.00 | 25.28 | 21.35 |
| 4h4f | 31.13 | 29.74 | 28.94 | 29.18 | 35.92 | 32.36 | 29.10 | 28.25 | 29.53 | 32.86 | 35.05 | 32.87 | 34.82 | 31.67 | 27.43 | 34.02 | 33.78 | 31.97 | 33.30 | 29.06 |
| 4hom | 1.41 | 18.11 | 29.78 | 30.94 | 26.36 | 21.62 | 24.79 | 22.53 | 20.31 | 24.19 | 18.21 | 29.65 | 14.39 | 27.73 | 24.45 | 24.71 | 21.28 | 22.05 | 28.92 | 22.42 |
| 4htp | 22.36 | 24.19 | 23.26 | 24.01 | 15.39 | 21.65 | 14.27 | 22.38 | 23.38 | 26.90 | 23.69 | 20.45 | 30.31 | 34.09 | 43.31 | 21.81 | 23.74 | 21.39 | 21.98 | 29.35 |
| 4iim | 13.84 | 28.78 | 24.62 | 16.74 | 27.21 | 14.18 | 0.75 | 20.39 | 7.75 | 13.23 | 23.49 | 17.25 | 19.88 | 24.28 | 22.43 | 10.73 | 17.07 | 20.81 | 20.12 | 21.01 |
| 4j8s | 8.87 | 11.93 | 32.37 | 12.23 | 17.67 | 29.10 | 12.59 | 30.18 | 32.09 | 14.30 | 11.10 | 19.40 | 12.97 | 12.23 | 10.26 | 17.83 | 31.99 | 11.51 | 19.40 | 13.55 |
| 4k0u | 16.24 | 1.78 | 13.77 | 4.52 | 6.73 | 14.52 | 19.14 | 18.18 | 14.50 | 5.64 | 10.65 | 16.07 | 13.89 | 8.03 | 10.01 | 5.96 | 17.16 | 14.17 | 4.41 | 12.88 |

**S4(e). L-RMSD values of all 20 poses obtained after blind docking by pepATTRACT on 133 protein-peptide complexes**

| **ID** | **Pose1** | **Pose2** | **Pose3** | **Pose4** | **Pose5** | **Pose6** | **Pose7** | **Pose8** | **Pose9** | **Pose10** | **Pose11** | **Pose12** | **Pose13** | **Pose14** | **Pose15** | **Pose16** | **Pose17** | **Pose18** | **Pose19** | **Pose20** |
| --- | --- | --- | --- | --- | --- | --- | --- | --- | --- | --- | --- | --- | --- | --- | --- | --- | --- | --- | --- | --- |
| 1cjr | 17.50 | 16.42 | 20.09 | 15.17 | 17.50 | 13.94 | 15.23 | 14.39 | 12.68 | 13.87 | 14.31 | 14.07 | 14.58 | 24.35 | 13.98 | 13.31 | 20.62 | 21.24 | 22.69 | 21.48 |
| 1cka | 2.04 | 1.76 | 1.77 | 14.25 | 1.53 | 1.58 | 4.37 | 1.50 | 5.37 | 6.14 | 11.18 | 3.80 | 4.74 | 4.83 | 4.84 | 4.57 | 14.61 | 15.26 | 4.33 | 2.59 |
| 1cvu | 45.72 | 42.76 | 41.88 | 41.96 | 44.03 | 37.37 | 42.50 | 43.67 | 41.93 | 43.02 | 43.53 | 43.93 | 43.50 | 54.65 | 42.75 | 44.58 | 40.41 | 45.57 | 39.97 | 46.30 |
| 1d4t | 27.03 | 27.74 | 20.85 | 27.20 | 27.87 | 27.87 | 28.76 | 30.10 | 30.04 | 26.03 | 27.78 | 28.89 | 29.51 | 29.19 | 29.21 | 28.13 | 29.10 | 29.78 | 27.68 | 27.42 |
| 1eg4 | 41.63 | 42.04 | 42.70 | 41.47 | 35.91 | 44.16 | 44.40 | 26.86 | 37.31 | 41.20 | 21.30 | 28.74 | 35.68 | 36.44 | 43.22 | 35.75 | 12.95 | 43.27 | 42.27 | 44.52 |
| 1h6w | 39.82 | 39.11 | 40.12 | 39.24 | 39.24 | 39.30 | 39.64 | 39.40 | 38.60 | 38.64 | 39.00 | 39.35 | 38.89 | 52.37 | 52.34 | 39.78 | 35.42 | 52.14 | 38.90 | 50.99 |
| 1hc9 | 15.35 | 18.04 | 18.92 | 13.60 | 17.25 | 11.93 | 15.30 | 13.60 | 13.57 | 13.54 | 19.52 | 15.85 | 13.51 | 20.15 | 13.45 | 24.67 | 15.23 | 14.28 | 15.69 | 13.43 |
| 1jbu | 22.92 | 22.88 | 21.70 | 21.99 | 22.83 | 11.04 | 10.81 | 10.23 | 24.78 | 10.45 | 45.14 | 11.00 | 22.85 | 25.76 | 22.73 | 13.69 | 15.98 | 22.97 | 40.61 | 11.14 |
| 1k5n | 22.02 | 21.74 | 22.61 | 21.64 | 21.58 | 21.67 | 22.23 | 8.56 | 21.96 | 21.83 | 21.47 | 20.70 | 22.61 | 20.81 | 22.34 | 21.45 | 22.28 | 22.53 | 21.48 | 21.91 |
| 1mfg | 16.25 | 16.61 | 13.54 | 16.87 | 14.51 | 14.34 | 16.98 | 14.25 | 24.88 | 14.90 | 20.18 | 11.86 | 15.52 | 14.23 | 16.16 | 16.63 | 13.39 | 17.55 | 16.45 | 22.24 |
| 1nln | 30.56 | 30.57 | 33.61 | 31.85 | 32.64 | 31.40 | 32.80 | 31.76 | 32.65 | 31.85 | 33.44 | 32.06 | 31.75 | 32.19 | 33.54 | 31.39 | 31.91 | 33.05 | 33.20 | 32.34 |
| 1nq7 | 32.64 | 38.41 | 32.95 | 38.86 | 32.02 | 35.81 | 33.41 | 32.13 | 24.01 | 35.42 | 34.91 | 32.62 | 35.84 | 32.84 | 33.88 | 34.77 | 23.74 | 33.58 | 34.56 | 32.82 |
| 1ntv | 15.67 | 19.73 | 15.73 | 13.90 | 30.50 | 14.04 | 15.84 | 14.18 | 15.32 | 31.96 | 22.24 | 15.69 | 16.82 | 16.93 | 16.67 | 30.59 | 16.48 | 16.28 | 26.70 | 16.72 |
| 1nx1 | 33.76 | 32.98 | 35.16 | 33.05 | 33.21 | 35.17 | 33.05 | 33.06 | 33.06 | 33.61 | 32.69 | 33.42 | 33.01 | 33.17 | 33.28 | 33.05 | 33.50 | 32.91 | 32.90 | 32.99 |
| 1oai | 7.96 | 8.01 | 8.17 | 9.92 | 7.71 | 7.67 | 8.22 | 8.37 | 9.75 | 7.37 | 9.69 | 7.63 | 8.32 | 9.61 | 8.41 | 7.83 | 10.51 | 10.06 | 9.69 | 7.94 |
| 1oj5 | 25.37 | 22.47 | 24.60 | 24.42 | 26.62 | 28.01 | 24.74 | 22.81 | 32.62 | 24.11 | 32.78 | 22.47 | 10.13 | 24.83 | 7.68 | 19.34 | 10.62 | 26.84 | 32.65 | 9.25 |
| 1ou8 | 12.77 | 12.72 | 13.31 | 14.40 | 15.88 | 14.19 | 15.10 | 15.51 | 27.96 | 25.21 | 14.83 | 9.43 | 15.10 | 14.10 | 12.04 | 13.68 | 14.22 | 31.24 | 19.62 | 18.20 |
| 1ow6 | 13.04 | 13.48 | 14.19 | 14.07 | 10.69 | 14.48 | 13.06 | 14.83 | 13.19 | 13.27 | 11.09 | 13.02 | 11.09 | 12.65 | 15.83 | 12.63 | 12.85 | 15.19 | 30.09 | 14.41 |
| 1pzl | 37.64 | 29.84 | 30.35 | 29.38 | 29.09 | 30.49 | 35.06 | 29.32 | 43.34 | 29.27 | 28.89 | 34.71 | 27.13 | 31.04 | 38.12 | 30.42 | 35.95 | 24.20 | 33.66 | 34.34 |
| 1qkz | 9.93 | 35.65 | 36.78 | 34.66 | 36.78 | 36.54 | 36.20 | 37.40 | 36.52 | 36.84 | 35.40 | 36.10 | 36.88 | 36.33 | 34.55 | 13.69 | 36.83 | 33.78 | 15.05 | 36.15 |
| 1rst | 8.19 | 8.00 | 8.38 | 7.84 | 8.20 | 6.74 | 7.64 | 6.62 | 7.42 | 7.72 | 6.90 | 13.03 | 7.10 | 6.79 | 7.89 | 9.76 | 10.28 | 7.36 | 6.39 | 8.02 |
| 1rxz | 20.02 | 19.98 | 22.57 | 20.60 | 23.42 | 20.68 | 23.43 | 23.23 | 38.49 | 22.12 | 20.28 | 24.61 | 22.10 | 22.09 | 21.51 | 22.08 | 38.37 | 23.88 | 39.02 | 23.71 |
| 1sfi | 20.25 | 20.20 | 20.20 | 20.19 | 17.94 | 21.25 | 20.21 | 20.53 | 18.66 | 21.23 | 19.62 | 19.95 | 20.89 | 20.30 | 19.68 | 15.81 | 21.07 | 17.83 | 18.10 | 13.27 |
| 1ssh | 2.77 | 2.13 | 4.79 | 1.41 | 2.00 | 2.20 | 1.81 | 6.55 | 2.71 | 2.63 | 5.52 | 2.03 | 2.36 | 5.42 | 3.82 | 6.21 | 2.64 | 4.26 | 2.37 | 1.73 |
| 1t08 | 18.70 | 20.74 | 21.19 | 34.22 | 20.89 | 20.66 | 21.36 | 22.36 | 21.16 | 19.82 | 18.76 | 26.83 | 26.01 | 26.06 | 20.60 | 20.80 | 19.59 | 26.08 | 31.41 | 20.43 |
| 1t4f | 5.60 | 5.71 | 23.14 | 5.82 | 13.69 | 23.60 | 7.57 | 14.62 | 7.54 | 17.56 | 5.79 | 7.09 | 6.28 | 6.63 | 5.90 | 18.10 | 13.39 | 21.24 | 22.13 | 21.48 |
| 1t7r | 34.76 | 31.86 | 11.82 | 11.68 | 36.33 | 11.59 | 15.52 | 38.58 | 14.24 | 11.82 | 10.62 | 11.60 | 39.53 | 37.16 | 11.02 | 11.82 | 11.73 | 13.18 | 30.56 | 38.16 |
| 1tfc | 10.80 | 11.86 | 11.22 | 10.37 | 12.72 | 8.32 | 12.27 | 11.12 | 10.60 | 13.63 | 10.67 | 9.54 | 12.29 | 11.66 | 10.48 | 11.15 | 10.90 | 10.59 | 10.01 | 11.47 |
| 1u00 | 19.06 | 20.66 | 20.51 | 15.24 | 19.35 | 14.53 | 19.49 | 15.96 | 19.08 | 15.19 | 14.60 | 14.47 | 14.00 | 13.76 | 14.74 | 19.54 | 15.68 | 15.99 | 16.23 | 14.81 |
| 1uj0 | 6.75 | 7.61 | 7.10 | 4.07 | 6.68 | 4.75 | 4.79 | 7.04 | 6.51 | 10.38 | 16.10 | 16.68 | 6.76 | 15.80 | 7.66 | 8.27 | 12.71 | 7.76 | 15.52 | 11.28 |
| 1x2r | 14.42 | 14.77 | 14.79 | 14.19 | 13.99 | 14.21 | 15.05 | 13.29 | 13.50 | 13.35 | 13.53 | 24.03 | 37.60 | 9.28 | 37.30 | 9.18 | 8.65 | 13.09 | 8.96 | 26.83 |
| 1xoc | 24.97 | 29.80 | 34.41 | 34.30 | 28.98 | 28.68 | 29.10 | 28.27 | 28.14 | 28.51 | 28.59 | 29.20 | 28.71 | 28.19 | 26.91 | 29.06 | 35.59 | 26.89 | 28.07 | 28.58 |
| 1ymt | 11.87 | 11.32 | 33.88 | 40.46 | 10.38 | 12.42 | 10.74 | 12.86 | 34.74 | 13.16 | 37.96 | 11.59 | 33.13 | 32.72 | 33.32 | 11.33 | 34.72 | 36.30 | 11.69 | 34.31 |
| 1yuc | 21.36 | 34.00 | 34.06 | 33.85 | 36.11 | 33.30 | 28.84 | 28.25 | 33.41 | 38.75 | 33.50 | 36.15 | 32.99 | 36.01 | 35.78 | 33.06 | 37.86 | 24.83 | 20.79 | 28.00 |
| 1ywo | 12.72 | 17.26 | 13.56 | 6.10 | 12.99 | 17.66 | 15.30 | 16.35 | 4.48 | 9.36 | 12.23 | 6.81 | 6.06 | 15.86 | 14.78 | 16.97 | 5.72 | 4.44 | 3.81 | 6.13 |
| 2a25 | 25.04 | 25.78 | 25.65 | 25.20 | 25.05 | 12.11 | 22.91 | 24.58 | 27.48 | 9.82 | 23.14 | 9.91 | 26.47 | 25.54 | 25.52 | 10.38 | 23.81 | 4.30 | 25.12 | 24.71 |
| 2a3i | 33.01 | 19.07 | 33.79 | 29.24 | 6.09 | 32.26 | 12.19 | 22.95 | 41.77 | 32.44 | 40.35 | 34.00 | 41.72 | 21.16 | 41.85 | 31.66 | 42.08 | 10.88 | 7.97 | 26.10 |
| 2aq9 | 30.10 | 29.13 | 31.38 | 31.20 | 35.73 | 37.78 | 32.45 | 32.01 | 30.76 | 37.63 | 29.44 | 38.55 | 29.73 | 33.93 | 30.99 | 37.71 | 37.28 | 29.83 | 31.71 | 39.68 |
| 2b9h | 5.05 | 6.61 | 14.72 | 42.39 | 42.27 | 43.23 | 16.50 | 16.16 | 16.10 | 29.08 | 14.15 | 15.95 | 6.68 | 16.08 | 15.66 | 9.85 | 29.27 | 29.51 | 33.12 | 32.11 |
| 2bba | 10.29 | 10.01 | 27.38 | 28.69 | 26.82 | 10.62 | 27.85 | 7.15 | 29.24 | 28.37 | 8.55 | 6.33 | 5.70 | 5.40 | 5.94 | 8.27 | 28.16 | 6.23 | 26.95 | 27.46 |
| 2cch | 33.37 | 34.02 | 36.62 | 33.06 | 36.70 | 36.72 | 36.60 | 36.43 | 9.16 | 36.17 | 36.62 | 36.18 | 36.70 | 36.06 | 32.56 | 35.98 | 38.77 | 36.54 | 36.16 | 37.68 |
| 2ce8 | 26.45 | 32.63 | 31.01 | 29.93 | 29.22 | 31.62 | 27.98 | 29.75 | 30.38 | 29.17 | 28.76 | 29.26 | 30.82 | 30.20 | 30.43 | 29.86 | 28.09 | 29.11 | 28.99 | 29.88 |
| 2d0n | 5.25 | 7.13 | 6.83 | 8.57 | 7.19 | 6.30 | 7.68 | 7.75 | 6.01 | 7.92 | 6.45 | 12.67 | 7.52 | 13.56 | 8.88 | 7.19 | 7.77 | 7.96 | 12.53 | 11.04 |
| 2drk | 17.61 | 17.73 | 17.89 | 17.69 | 17.84 | 17.62 | 17.37 | 9.39 | 11.01 | 11.08 | 10.96 | 17.65 | 17.86 | 11.49 | 2.37 | 10.39 | 9.46 | 18.31 | 18.18 | 17.60 |
| 2dyp | 25.45 | 23.83 | 24.30 | 22.40 | 20.17 | 23.44 | 24.44 | 20.37 | 24.06 | 24.77 | 20.03 | 24.60 | 20.13 | 19.53 | 23.88 | 23.87 | 19.56 | 20.04 | 23.10 | 24.02 |
| 2fff | 44.68 | 46.84 | 33.10 | 52.07 | 53.35 | 47.25 | 37.35 | 47.43 | 51.89 | 45.41 | 43.71 | 43.23 | 42.34 | 51.54 | 43.36 | 43.34 | 42.73 | 46.79 | 46.44 | 53.72 |
| 2ffu | 14.90 | 15.14 | 15.39 | 18.72 | 15.04 | 18.33 | 5.43 | 14.51 | 18.38 | 18.59 | 16.79 | 15.63 | 18.04 | 14.34 | 15.40 | 17.47 | 16.55 | 5.77 | 15.88 | 19.14 |
| 2fka | 4.77 | 5.70 | 6.35 | 5.92 | 5.22 | 4.78 | 5.62 | 5.09 | 5.73 | 6.06 | 5.63 | 6.61 | 4.77 | 6.50 | 5.54 | 5.28 | 5.54 | 5.67 | 6.44 | 4.86 |
| 2fmf | 5.75 | 9.60 | 6.99 | 10.43 | 9.62 | 9.78 | 10.16 | 9.63 | 11.78 | 10.12 | 12.11 | 12.43 | 9.79 | 10.26 | 12.26 | 12.25 | 10.12 | 10.60 | 13.11 | 10.18 |
| 2fts | 35.18 | 30.52 | 30.67 | 27.35 | 26.11 | 30.85 | 30.82 | 25.71 | 28.66 | 26.94 | 36.15 | 26.85 | 35.13 | 34.58 | 27.00 | 34.58 | 34.68 | 32.98 | 29.63 | 28.00 |
| 2fvj | 25.06 | 23.30 | 23.39 | 23.86 | 24.49 | 24.25 | 22.75 | 24.35 | 30.36 | 19.17 | 13.47 | 19.04 | 23.57 | 18.79 | 27.55 | 20.14 | 27.39 | 18.24 | 47.33 | 23.15 |
| 2ho2 | 8.52 | 18.52 | 8.51 | 19.20 | 18.44 | 5.20 | 19.22 | 5.69 | 18.82 | 18.43 | 15.60 | 18.08 | 18.00 | 5.39 | 16.10 | 18.94 | 18.45 | 18.42 | 18.01 | 17.43 |
| 2ht9 | 30.97 | 30.03 | 30.51 | 31.44 | 34.22 | 30.27 | 32.54 | 34.89 | 32.18 | 30.46 | 30.94 | 31.88 | 36.62 | 34.47 | 35.05 | 27.78 | 33.64 | 32.25 | 29.93 | 31.57 |
| 2o02 | 20.81 | 9.53 | 21.54 | 11.64 | 21.09 | 21.51 | 20.94 | 8.88 | 21.53 | 21.02 | 9.12 | 21.39 | 9.70 | 14.19 | 18.08 | 21.87 | 10.37 | 14.73 | 21.70 | 21.01 |
| 2o4j | 39.88 | 41.14 | 40.41 | 40.42 | 41.38 | 41.72 | 41.61 | 41.22 | 40.48 | 41.47 | 42.20 | 40.43 | 40.80 | 41.52 | 41.62 | 41.38 | 41.72 | 42.01 | 40.74 | 41.11 |
| 2o9v | 14.02 | 13.71 | 13.84 | 13.85 | 13.80 | 14.32 | 14.01 | 13.82 | 14.09 | 13.29 | 13.60 | 13.82 | 13.83 | 13.93 | 13.66 | 13.94 | 13.53 | 13.65 | 14.55 | 13.80 |
| 2oei | 4.80 | 17.64 | 19.29 | 12.15 | 18.86 | 18.69 | 4.96 | 19.97 | 4.84 | 4.40 | 12.93 | 21.10 | 20.82 | 11.15 | 21.73 | 21.82 | 18.55 | 20.32 | 19.52 | 6.26 |
| 2p0w | 22.37 | 21.86 | 22.38 | 22.35 | 22.69 | 23.45 | 22.09 | 24.57 | 23.11 | 22.22 | 25.90 | 25.87 | 23.58 | 34.04 | 32.03 | 23.31 | 34.72 | 34.96 | 21.24 | 33.22 |
| 2p1o | 32.55 | 30.24 | 30.73 | 32.34 | 35.99 | 32.97 | 33.55 | 31.47 | 27.46 | 28.42 | 31.79 | 36.23 | 20.40 | 35.96 | 20.99 | 32.43 | 19.83 | 20.57 | 43.23 | 20.66 |
| 2p1t | 33.38 | 31.41 | 33.86 | 30.23 | 32.12 | 31.83 | 31.77 | 32.43 | 33.19 | 27.63 | 13.99 | 17.36 | 33.60 | 16.20 | 31.25 | 30.20 | 18.09 | 17.69 | 27.90 | 17.53 |
| 2p54 | 44.70 | 45.12 | 45.00 | 44.95 | 44.95 | 43.91 | 43.64 | 21.77 | 43.69 | 44.38 | 44.43 | 45.62 | 45.34 | 44.53 | 45.40 | 45.49 | 45.33 | 44.62 | 44.67 | 18.75 |
| 2peh | 33.50 | 31.01 | 30.97 | 23.04 | 30.99 | 32.90 | 32.47 | 22.09 | 22.99 | 32.91 | 29.79 | 32.47 | 14.79 | 31.03 | 15.21 | 15.26 | 13.97 | 21.98 | 14.98 | 32.59 |
| 2pux | 15.57 | 15.74 | 15.64 | 14.61 | 15.80 | 16.79 | 15.73 | 14.70 | 16.87 | 15.93 | 15.74 | 16.09 | 16.43 | 16.76 | 15.53 | 15.53 | 16.99 | 16.20 | 16.10 | 36.99 |
| 2puy | 15.54 | 15.67 | 14.45 | 15.47 | 15.54 | 16.74 | 15.53 | 15.22 | 15.60 | 15.87 | 16.55 | 15.05 | 15.76 | 15.29 | 15.69 | 16.90 | 15.35 | 16.18 | 14.23 | 17.28 |
| 2qbx | 25.79 | 24.08 | 31.74 | 24.01 | 26.65 | 21.40 | 22.87 | 35.88 | 15.01 | 40.20 | 25.17 | 23.80 | 40.18 | 23.81 | 10.37 | 35.95 | 27.48 | 26.09 | 23.61 | 37.39 |
| 2qos | 23.68 | 26.49 | 26.05 | 23.79 | 26.46 | 26.47 | 7.84 | 14.39 | 23.67 | 11.76 | 13.59 | 14.35 | 23.87 | 8.51 | 24.54 | 24.42 | 11.28 | 7.80 | 7.49 | 8.26 |
| 2qse | 32.59 | 35.36 | 38.52 | 39.08 | 37.98 | 38.28 | 38.24 | 38.96 | 36.89 | 39.05 | 34.94 | 38.11 | 39.89 | 35.99 | 39.21 | 12.32 | 34.47 | 37.40 | 38.67 | 36.31 |
| 2r7g | 29.00 | 37.78 | 20.14 | 37.03 | 23.10 | 37.78 | 37.64 | 24.51 | 36.73 | 37.70 | 38.02 | 37.52 | 38.45 | 37.46 | 23.24 | 38.67 | 38.48 | 37.80 | 37.83 | 37.60 |
| 2r9q | 8.97 | 6.72 | 6.61 | 6.86 | 16.84 | 6.52 | 6.72 | 16.85 | 8.72 | 7.11 | 7.23 | 7.53 | 7.83 | 7.12 | 17.48 | 7.28 | 7.34 | 7.19 | 7.92 | 7.34 |
| 2v8y | 15.04 | 34.15 | 36.97 | 23.65 | 23.56 | 36.56 | 38.66 | 37.51 | 18.34 | 40.35 | 23.46 | 37.20 | 38.61 | 30.95 | 23.61 | 37.21 | 23.15 | 22.49 | 37.90 | 23.80 |
| 2vkn | 5.36 | 5.98 | 5.84 | 6.74 | 6.92 | 6.28 | 7.35 | 6.17 | 5.87 | 6.60 | 12.36 | 6.79 | 5.95 | 7.15 | 6.13 | 4.71 | 8.61 | 5.35 | 6.39 | 9.63 |
| 2vr3 | 27.24 | 26.58 | 25.52 | 26.80 | 24.50 | 32.81 | 33.69 | 27.34 | 26.22 | 30.01 | 33.27 | 24.17 | 28.05 | 29.63 | 29.09 | 32.73 | 16.10 | 19.16 | 19.10 | 28.77 |
| 2vwf | 14.22 | 15.68 | 31.35 | 11.54 | 14.95 | 25.81 | 13.39 | 16.10 | 23.46 | 13.17 | 22.89 | 19.84 | 21.51 | 10.34 | 19.74 | 25.45 | 8.61 | 21.67 | 8.62 | 22.74 |
| 2w2u | 9.88 | 8.13 | 7.84 | 8.70 | 8.24 | 7.75 | 9.95 | 9.40 | 8.68 | 21.40 | 20.08 | 21.81 | 7.84 | 6.80 | 23.72 | 20.17 | 8.46 | 8.26 | 10.06 | 7.47 |
| 2whx | 62.46 | 61.83 | 62.40 | 61.68 | 58.14 | 63.94 | 61.87 | 61.40 | 63.20 | 62.52 | 29.47 | 61.66 | 63.63 | 61.38 | 61.09 | 27.67 | 60.76 | 30.40 | 63.26 | 61.23 |
| 2xrw | 41.22 | 32.42 | 28.75 | 39.39 | 37.38 | 39.39 | 39.90 | 40.07 | 38.55 | 39.38 | 28.61 | 39.69 | 39.89 | 35.60 | 38.16 | 25.29 | 29.92 | 27.59 | 28.53 | 39.45 |
| 2xu7 | 5.52 | 6.26 | 6.23 | 40.40 | 41.08 | 9.42 | 6.35 | 42.58 | 43.75 | 6.50 | 11.83 | 40.13 | 12.07 | 43.55 | 42.56 | 6.40 | 43.32 | 41.87 | 9.81 | 42.55 |
| 2xvc | 21.71 | 22.34 | 24.10 | 21.02 | 24.53 | 23.00 | 20.86 | 19.31 | 23.25 | 22.69 | 22.08 | 22.68 | 24.79 | 22.77 | 25.79 | 13.35 | 28.39 | 24.53 | 19.69 | 22.35 |
| 2zjd | 4.53 | 5.35 | 6.48 | 5.04 | 5.26 | 5.00 | 6.10 | 11.54 | 5.90 | 5.05 | 5.55 | 5.59 | 30.88 | 8.09 | 22.10 | 15.39 | 9.07 | 7.34 | 15.41 | 25.83 |
| 3asl | 24.04 | 17.56 | 24.86 | 10.69 | 17.82 | 10.44 | 18.86 | 20.49 | 11.50 | 27.06 | 20.02 | 19.82 | 19.91 | 18.43 | 10.62 | 20.77 | 17.52 | 17.49 | 22.63 | 20.11 |
| 3awr | 19.27 | 19.39 | 19.30 | 19.46 | 19.28 | 17.13 | 20.80 | 9.57 | 18.46 | 19.26 | 18.87 | 7.23 | 7.66 | 8.13 | 7.65 | 7.44 | 18.72 | 19.83 | 6.55 | 17.81 |
| 3ayu | 17.69 | 17.55 | 7.09 | 7.56 | 8.41 | 8.31 | 8.46 | 24.63 | 8.81 | 8.24 | 8.94 | 8.86 | 9.46 | 9.08 | 17.63 | 17.75 | 8.83 | 6.70 | 7.41 | 6.66 |
| 3bfq | 31.59 | 31.35 | 31.46 | 31.44 | 32.17 | 8.89 | 12.51 | 32.71 | 7.39 | 12.39 | 6.79 | 11.97 | 6.20 | 31.55 | 35.77 | 28.24 | 31.49 | 20.44 | 30.04 | 10.94 |
| 3c3r | 39.41 | 38.87 | 39.18 | 39.64 | 30.20 | 40.09 | 39.01 | 38.05 | 39.71 | 20.01 | 40.88 | 37.62 | 39.54 | 39.75 | 39.53 | 36.72 | 39.89 | 40.87 | 39.75 | 38.40 |
| 3d32 | 23.30 | 23.62 | 17.90 | 27.54 | 21.96 | 24.45 | 24.26 | 23.30 | 27.93 | 18.23 | 8.67 | 18.93 | 23.90 | 29.73 | 20.41 | 27.19 | 18.27 | 21.84 | 22.09 | 18.99 |
| 3ds4 | 25.74 | 24.54 | 23.86 | 24.07 | 24.14 | 25.65 | 23.81 | 24.24 | 24.17 | 23.89 | 25.24 | 24.68 | 22.74 | 25.84 | 24.63 | 24.06 | 26.31 | 24.65 | 27.51 | 24.10 |
| 3ery | 7.33 | 4.73 | 4.87 | 4.38 | 5.32 | 5.12 | 4.64 | 4.69 | 8.12 | 3.70 | 7.60 | 3.65 | 4.41 | 4.94 | 3.57 | 6.14 | 15.66 | 16.40 | 6.42 | 4.38 |
| 3fdo | 21.57 | 19.51 | 18.15 | 18.01 | 19.29 | 8.41 | 10.35 | 18.70 | 11.54 | 10.73 | 9.09 | 12.52 | 7.56 | 8.25 | 9.67 | 8.05 | 10.40 | 10.75 | 7.67 | 8.46 |
| 3g2s | 11.41 | 25.22 | 19.46 | 23.81 | 13.52 | 15.02 | 11.60 | 9.37 | 14.59 | 15.67 | 14.85 | 21.37 | 26.00 | 8.70 | 33.07 | 12.88 | 10.49 | 13.60 | 33.54 | 12.48 |
| 3gyt | 31.19 | 43.59 | 31.05 | 30.65 | 42.28 | 29.07 | 31.10 | 42.37 | 30.70 | 42.48 | 33.09 | 10.49 | 43.16 | 30.45 | 31.30 | 31.02 | 40.60 | 25.08 | 30.79 | 31.09 |
| 3h1z | 32.80 | 25.36 | 28.92 | 28.37 | 27.92 | 55.85 | 28.18 | 27.44 | 28.72 | 28.79 | 55.09 | 28.40 | 56.53 | 28.33 | 31.36 | 55.34 | 28.30 | 36.05 | 29.02 | 29.61 |
| 3i5r | 15.10 | 12.32 | 15.00 | 16.27 | 16.07 | 16.35 | 14.63 | 15.68 | 9.72 | 16.58 | 16.37 | 15.59 | 16.27 | 16.02 | 11.40 | 13.89 | 16.53 | 16.65 | 15.09 | 3.53 |
| 3ivv | 16.71 | 15.95 | 16.42 | 15.82 | 16.99 | 16.59 | 16.60 | 16.03 | 16.14 | 18.02 | 16.77 | 16.23 | 15.70 | 16.01 | 15.63 | 27.93 | 15.23 | 16.77 | 16.64 | 16.59 |
| 3kmr | 34.16 | 32.30 | 33.29 | 33.23 | 33.68 | 33.10 | 33.13 | 33.31 | 34.00 | 33.51 | 34.31 | 33.06 | 32.26 | 32.30 | 33.90 | 33.01 | 33.93 | 32.70 | 32.77 | 33.15 |
| 3kuj | 22.56 | 22.09 | 21.62 | 20.20 | 23.68 | 22.12 | 13.76 | 22.50 | 24.02 | 23.37 | 24.05 | 24.07 | 24.36 | 21.88 | 31.04 | 21.50 | 31.14 | 31.41 | 23.52 | 21.85 |
| 3kus | 19.20 | 22.26 | 20.83 | 17.68 | 18.42 | 19.54 | 16.24 | 16.38 | 17.89 | 19.01 | 17.38 | 15.63 | 17.63 | 15.47 | 17.73 | 20.36 | 20.30 | 17.44 | 17.97 | 20.12 |
| 3l0e | 39.76 | 39.22 | 34.64 | 21.75 | 39.11 | 39.16 | 32.01 | 32.56 | 34.39 | 34.99 | 30.40 | 25.46 | 13.78 | 19.82 | 38.05 | 21.86 | 34.88 | 36.72 | 32.14 | 26.97 |
| 3ll8 | 35.26 | 34.84 | 35.96 | 35.80 | 34.90 | 35.71 | 35.12 | 35.56 | 35.61 | 35.28 | 36.80 | 35.77 | 34.61 | 35.95 | 35.02 | 36.81 | 34.44 | 35.64 | 35.20 | 39.17 |
| 3llz | 24.67 | 24.64 | 24.60 | 21.88 | 24.64 | 24.03 | 23.26 | 24.65 | 24.34 | 23.21 | 23.69 | 24.43 | 23.22 | 24.93 | 23.75 | 24.50 | 24.07 | 28.14 | 24.57 | 24.81 |
| 3obq | 16.93 | 16.85 | 16.60 | 17.04 | 6.32 | 6.59 | 25.14 | 24.39 | 4.71 | 26.19 | 24.36 | 24.39 | 17.00 | 24.52 | 23.73 | 24.32 | 25.33 | 6.01 | 16.82 | 29.10 |
| 3olf | 14.72 | 33.23 | 15.57 | 15.07 | 32.09 | 15.72 | 15.34 | 32.33 | 33.15 | 33.23 | 32.61 | 32.04 | 15.29 | 32.36 | 32.96 | 32.41 | 31.62 | 31.98 | 15.85 | 44.47 |
| 3p72 | 11.06 | 12.71 | 13.27 | 16.35 | 12.08 | 14.23 | 13.35 | 13.62 | 11.35 | 13.83 | 12.06 | 13.12 | 13.87 | 13.88 | 12.30 | 15.82 | 13.09 | 14.36 | 10.09 | 33.87 |
| 3p8f | 32.66 | 33.16 | 12.97 | 17.35 | 17.33 | 17.34 | 31.72 | 18.37 | 17.09 | 30.98 | 32.10 | 16.08 | 16.47 | 16.86 | 18.15 | 12.72 | 17.66 | 17.45 | 18.80 | 18.63 |
| 3ptl | 21.87 | 17.89 | 19.27 | 18.06 | 21.06 | 18.40 | 21.17 | 18.08 | 17.38 | 18.26 | 18.08 | 17.51 | 20.30 | 17.33 | 18.32 | 18.06 | 17.95 | 17.40 | 18.37 | 18.28 |
| 3qis | 31.32 | 30.09 | 10.26 | 9.70 | 6.04 | 32.65 | 8.12 | 8.98 | 9.09 | 7.93 | 6.72 | 9.04 | 9.52 | 10.75 | 8.49 | 6.66 | 7.04 | 34.08 | 32.15 | 9.82 |
| 3rm1 | 20.86 | 20.81 | 21.55 | 18.15 | 17.47 | 16.96 | 17.68 | 19.38 | 17.39 | 17.62 | 16.42 | 16.16 | 22.97 | 17.76 | 18.55 | 17.29 | 17.57 | 22.91 | 22.65 | 15.78 |
| 3rqg | 36.19 | 27.96 | 35.42 | 36.33 | 32.62 | 27.57 | 28.10 | 35.47 | 35.85 | 35.53 | 33.45 | 32.26 | 33.77 | 32.17 | 34.21 | 29.60 | 34.31 | 37.37 | 36.94 | 32.32 |
| 3sfj | 20.88 | 20.40 | 19.97 | 19.52 | 19.25 | 20.29 | 19.15 | 8.49 | 8.64 | 8.81 | 19.00 | 9.87 | 8.74 | 9.12 | 8.44 | 9.39 | 8.44 | 8.65 | 9.24 | 8.93 |
| 3so6 | 7.25 | 7.32 | 7.37 | 21.62 | 26.94 | 17.33 | 26.37 | 25.97 | 24.37 | 24.55 | 32.03 | 26.10 | 26.37 | 28.05 | 17.21 | 28.93 | 21.79 | 25.62 | 25.20 | 16.02 |
| 3tjv | 18.34 | 18.18 | 3.27 | 4.23 | 18.53 | 5.27 | 17.08 | 4.71 | 17.87 | 18.19 | 19.17 | 17.34 | 18.87 | 19.66 | 18.28 | 19.07 | 20.85 | 17.83 | 20.27 | 10.51 |
| 3tzy | 20.56 | 20.08 | 21.80 | 9.54 | 9.05 | 10.42 | 20.20 | 10.45 | 12.92 | 13.14 | 14.00 | 33.38 | 37.54 | 10.06 | 10.13 | 37.43 | 13.35 | 12.98 | 10.23 | 8.14 |
| 3u9q | 24.85 | 24.90 | 26.35 | 25.54 | 20.86 | 24.35 | 25.53 | 14.10 | 11.31 | 27.47 | 11.90 | 14.13 | 13.43 | 12.62 | 13.58 | 11.66 | 32.63 | 43.77 | 18.46 | 13.27 |
| 3up3 | 38.76 | 40.76 | 40.20 | 38.53 | 40.98 | 39.04 | 40.54 | 38.19 | 40.25 | 25.80 | 28.87 | 38.91 | 40.57 | 6.09 | 45.32 | 5.77 | 5.75 | 43.47 | 6.06 | 27.45 |
| 3v2x | 19.38 | 19.39 | 19.75 | 19.92 | 19.55 | 19.46 | 19.52 | 19.48 | 19.44 | 19.68 | 19.58 | 20.84 | 19.40 | 19.56 | 16.88 | 19.69 | 21.22 | 8.93 | 9.30 | 19.32 |
| 3vtc | 33.30 | 13.24 | 34.18 | 33.33 | 30.98 | 34.50 | 34.63 | 31.70 | 12.99 | 32.12 | 39.79 | 32.09 | 10.56 | 13.04 | 39.43 | 11.64 | 15.82 | 41.74 | 8.32 | 41.94 |
| 3w1b | 40.58 | 41.06 | 39.00 | 39.83 | 38.18 | 36.92 | 39.97 | 40.47 | 36.29 | 39.19 | 39.48 | 37.93 | 39.98 | 40.16 | 38.52 | 38.83 | 39.60 | 42.96 | 38.67 | 39.72 |
| 3zqh | 24.05 | 23.01 | 20.05 | 15.12 | 23.44 | 23.87 | 23.94 | 23.57 | 22.86 | 22.01 | 13.85 | 23.96 | 13.29 | 23.76 | 24.43 | 23.29 | 23.84 | 23.98 | 24.23 | 13.85 |
| 4b4n | 27.71 | 25.92 | 25.59 | 27.10 | 26.34 | 27.70 | 25.29 | 27.06 | 27.97 | 27.40 | 27.06 | 26.98 | 25.54 | 22.19 | 27.47 | 27.77 | 26.45 | 26.60 | 27.10 | 25.27 |
| 4dcb | 37.32 | 37.57 | 38.61 | 36.66 | 35.60 | 42.08 | 38.39 | 51.33 | 37.60 | 36.97 | 41.80 | 38.20 | 36.69 | 23.30 | 38.28 | 41.45 | 38.03 | 40.67 | 41.34 | 38.46 |
| 4e34 | 21.16 | 23.19 | 24.20 | 22.84 | 21.51 | 22.28 | 22.65 | 22.16 | 22.20 | 23.56 | 22.96 | 22.68 | 21.91 | 21.51 | 21.59 | 20.53 | 21.26 | 22.96 | 22.11 | 21.13 |
| 4eik | 3.84 | 5.09 | 6.98 | 14.61 | 14.41 | 15.78 | 19.68 | 5.01 | 13.09 | 22.30 | 3.17 | 4.50 | 6.38 | 18.83 | 3.50 | 19.87 | 5.72 | 22.25 | 6.44 | 4.49 |
| 4ery | 8.84 | 9.17 | 9.00 | 8.79 | 8.94 | 8.51 | 8.80 | 14.53 | 14.56 | 8.79 | 9.75 | 9.45 | 32.43 | 16.22 | 8.53 | 9.48 | 31.34 | 15.98 | 9.72 | 17.94 |
| 4f14 | 7.37 | 7.19 | 5.71 | 7.37 | 7.88 | 6.25 | 6.48 | 6.58 | 7.67 | 4.62 | 6.56 | 6.32 | 5.67 | 4.95 | 5.67 | 6.93 | 6.16 | 6.60 | 4.11 | 4.53 |
| 4f1z | 31.40 | 27.87 | 18.88 | 32.79 | 32.53 | 26.83 | 32.69 | 30.93 | 26.78 | 30.90 | 31.66 | 26.93 | 31.50 | 30.48 | 27.59 | 31.61 | 22.42 | 30.91 | 26.64 | 26.85 |
| 4gq6 | 12.34 | 30.19 | 13.91 | 13.39 | 13.03 | 12.77 | 12.30 | 12.99 | 13.31 | 12.95 | 13.09 | 12.78 | 13.38 | 13.31 | 12.93 | 12.03 | 12.64 | 12.90 | 12.79 | 12.26 |
| 4gxl | 35.50 | 33.39 | 35.36 | 32.27 | 11.76 | 34.56 | 32.38 | 34.26 | 33.08 | 32.69 | 32.40 | 14.01 | 19.40 | 32.90 | 32.51 | 11.38 | 16.08 | 14.87 | 34.24 | 13.20 |
| 4gyw | 13.96 | 39.93 | 12.12 | 13.18 | 17.95 | 20.64 | 32.37 | 33.00 | 36.67 | 34.67 | 32.08 | 28.09 | 32.83 | 14.88 | 36.65 | 33.42 | 32.27 | 26.52 | 33.47 | 15.68 |
| 4h4f | 33.73 | 20.22 | 33.32 | 20.63 | 16.95 | 17.95 | 33.48 | 19.77 | 16.32 | 16.80 | 20.56 | 17.60 | 19.84 | 18.00 | 17.17 | 16.88 | 16.78 | 17.79 | 18.11 | 12.45 |
| 4hom | 9.33 | 8.64 | 34.43 | 34.19 | 33.77 | 34.18 | 8.68 | 34.04 | 33.94 | 33.63 | 35.92 | 29.27 | 10.77 | 11.48 | 35.04 | 34.92 | 23.89 | 34.29 | 35.58 | 35.61 |
| 4htp | 21.75 | 18.90 | 21.41 | 20.43 | 20.91 | 20.50 | 20.71 | 20.85 | 19.90 | 18.63 | 20.84 | 20.61 | 20.91 | 21.83 | 21.07 | 22.75 | 22.03 | 34.39 | 20.40 | 22.14 |
| 4iim | 13.26 | 17.04 | 22.92 | 15.41 | 24.60 | 16.53 | 16.22 | 28.35 | 13.24 | 15.91 | 17.11 | 17.60 | 14.91 | 19.52 | 12.03 | 17.78 | 26.60 | 17.18 | 25.64 | 22.89 |
| 4j8s | 31.25 | 21.53 | 23.70 | 38.20 | 38.24 | 22.37 | 39.29 | 29.52 | 21.00 | 23.54 | 21.47 | 35.99 | 43.94 | 41.43 | 36.48 | 32.08 | 24.80 | 22.69 | 30.52 | 37.04 |
| 4k0u | 19.33 | 8.31 | 19.56 | 17.89 | 18.87 | 17.50 | 10.74 | 16.62 | 21.54 | 14.49 | 18.16 | 19.77 | 22.48 | 15.86 | 17.81 | 17.97 | 21.76 | 18.73 | 18.61 | 18.04 |

**S4(f). L-RMSD values of all 20 poses obtained after blind docking by FRODOCK on 133 protein-peptide complexes.**

| **ID** | **Pose1** | **Pose2** | **Pose3** | **Pose4** | **Pose5** | **Pose6** | **Pose7** | **Pose8** | **Pose9** | **Pose10** | **Pose11** | **Pose12** | **Pose13** | **Pose14** | **Pose15** | **Pose16** | **Pose17** | **Pose18** | **Pose19** | **Pose20** |
| --- | --- | --- | --- | --- | --- | --- | --- | --- | --- | --- | --- | --- | --- | --- | --- | --- | --- | --- | --- | --- |
| 1cjr | 3.22 | 13.50 | 3.84 | 5.34 | 20.25 | 24.53 | 27.56 | 18.88 | 27.08 | 17.63 | 16.73 | 26.21 | 26.44 | 19.12 | 10.02 | 30.03 | 14.75 | 11.87 | 24.12 | 8.50 |
| 1cka | 2.16 | 5.54 | 12.91 | 5.20 | 15.17 | 15.84 | 9.87 | 7.50 | 9.86 | 18.78 | 9.80 | 16.27 | 4.62 | 8.72 | 15.30 | 14.00 | 21.67 | 18.33 | 11.14 | 21.82 |
| 1cvu | 37.16 | 37.63 | 54.59 | 37.08 | 35.65 | 38.65 | 14.61 | 38.28 | 39.38 | 44.47 | 20.70 | 43.96 | 38.01 | 26.17 | 30.03 | 38.30 | 39.92 | 17.93 | 55.18 | 54.77 |
| 1d4t | 0.88 | 8.86 | 14.86 | 18.76 | 28.09 | 19.15 | 17.77 | 15.40 | 20.58 | 6.94 | 20.87 | 19.57 | 21.02 | 14.86 | 19.16 | 9.55 | 6.40 | 10.43 | 26.77 | 17.79 |
| 1eg4 | 42.05 | 5.27 | 17.52 | 42.72 | 17.64 | 14.13 | 1.21 | 36.95 | 18.93 | 43.10 | 20.85 | 16.00 | 43.66 | 42.39 | 44.95 | 11.62 | 21.05 | 18.05 | 44.64 | 33.54 |
| 1h6w | 1.22 | 20.87 | 18.68 | 16.90 | 12.18 | 22.93 | 16.19 | 19.29 | 18.00 | 11.65 | 19.70 | 19.34 | 17.48 | 13.89 | 9.28 | 14.48 | 64.75 | 23.04 | 21.59 | 11.88 |
| 1hc9 | 2.03 | 7.63 | 11.31 | 9.54 | 6.65 | 20.61 | 13.07 | 25.26 | 21.49 | 10.80 | 21.80 | 22.08 | 17.21 | 12.66 | 22.52 | 22.83 | 20.96 | 18.92 | 20.26 | 18.77 |
| 1jbu | 1.71 | 46.56 | 41.86 | 46.98 | 4.31 | 47.59 | 45.63 | 43.07 | 46.75 | 28.56 | 46.05 | 46.20 | 27.73 | 44.88 | 46.02 | 41.99 | 18.55 | 47.52 | 41.86 | 6.35 |
| 1k5n | 1.12 | 24.84 | 26.30 | 15.27 | 25.93 | 26.44 | 27.07 | 9.02 | 27.04 | 23.92 | 15.07 | 27.01 | 26.79 | 13.76 | 30.48 | 25.31 | 23.45 | 25.27 | 27.88 | 48.96 |
| 1mfg | 10.74 | 8.82 | 11.39 | 14.98 | 19.93 | 2.04 | 14.50 | 10.94 | 16.13 | 21.30 | 17.07 | 24.03 | 16.44 | 12.09 | 23.18 | 13.78 | 28.79 | 14.50 | 20.01 | 27.77 |
| 1nln | 29.67 | 1.53 | 6.48 | 32.94 | 20.22 | 30.53 | 36.35 | 29.96 | 27.71 | 33.76 | 21.35 | 30.19 | 31.69 | 32.09 | 27.53 | 41.53 | 24.37 | 15.64 | 33.02 | 25.55 |
| 1nq7 | 37.99 | 37.44 | 41.27 | 45.23 | 34.43 | 41.62 | 37.43 | 1.33 | 26.45 | 40.49 | 21.71 | 43.96 | 36.50 | 45.04 | 34.94 | 18.62 | 44.54 | 21.38 | 28.47 | 32.97 |
| 1ntv | 1.84 | 16.19 | 15.50 | 4.37 | 15.38 | 13.63 | 10.84 | 26.47 | 7.96 | 14.92 | 9.75 | 29.57 | 15.16 | 8.45 | 14.20 | 14.87 | 15.60 | 17.77 | 24.81 | 31.25 |
| 1nx1 | 1.73 | 33.82 | 35.99 | 34.36 | 35.38 | 38.68 | 6.67 | 5.98 | 36.02 | 36.85 | 35.39 | 34.88 | 33.93 | 35.45 | 11.69 | 43.98 | 34.97 | 35.71 | 7.11 | 10.20 |
| 1oai | 11.82 | 28.10 | 24.65 | 23.03 | 26.04 | 25.40 | 21.97 | 19.92 | 4.21 | 27.97 | 23.14 | 26.20 | 3.25 | 26.84 | 21.54 | 27.19 | 10.50 | 12.22 | 27.54 | 26.22 |
| 1oj5 | 4.77 | 3.51 | 15.25 | 14.19 | 9.32 | 14.73 | 20.03 | 14.50 | 33.76 | 25.05 | 8.34 | 24.53 | 12.82 | 15.22 | 15.59 | 18.38 | 16.24 | 13.94 | 18.32 | 23.89 |
| 1ou8 | 1.39 | 6.49 | 32.48 | 14.28 | 32.30 | 31.74 | 18.90 | 33.34 | 11.95 | 13.45 | 28.54 | 13.05 | 16.56 | 8.52 | 32.67 | 28.68 | 26.91 | 16.15 | 26.93 | 12.79 |
| 1ow6 | 10.55 | 28.64 | 15.07 | 13.37 | 13.38 | 29.48 | 12.14 | 14.83 | 16.69 | 15.54 | 11.60 | 11.49 | 10.19 | 31.09 | 13.89 | 28.93 | 31.45 | 12.37 | 38.11 | 28.80 |
| 1pzl | 41.83 | 39.54 | 42.34 | 38.22 | 39.08 | 3.76 | 8.62 | 41.60 | 28.26 | 33.12 | 19.37 | 40.87 | 46.06 | 38.60 | 27.74 | 35.32 | 46.35 | 42.37 | 42.88 | 30.41 |
| 1qkz | 10.45 | 11.21 | 7.90 | 11.06 | 12.71 | 9.33 | 10.95 | 10.26 | 15.22 | 10.04 | 12.89 | 13.45 | 9.16 | 9.66 | 9.50 | 10.36 | 48.27 | 11.10 | 9.43 | 8.49 |
| 1rst | 7.94 | 9.27 | 8.84 | 10.69 | 9.89 | 9.96 | 10.17 | 8.84 | 13.83 | 3.00 | 12.51 | 10.97 | 23.58 | 6.60 | 15.19 | 9.40 | 10.56 | 8.37 | 5.66 | 13.88 |
| 1rxz | 1.79 | 17.92 | 16.29 | 16.37 | 27.70 | 11.41 | 17.12 | 38.69 | 24.90 | 34.29 | 20.97 | 15.88 | 14.95 | 20.00 | 12.72 | 8.84 | 35.66 | 16.69 | 9.74 | 14.63 |
| 1sfi | 7.63 | 11.13 | 14.82 | 2.63 | 15.20 | 25.51 | 13.40 | 13.11 | 9.15 | 14.47 | 18.65 | 25.69 | 17.24 | 13.52 | 23.40 | 24.04 | 12.42 | 10.67 | 28.42 | 12.95 |
| 1ssh | 1.31 | 18.81 | 19.94 | 7.13 | 20.02 | 8.99 | 10.28 | 4.35 | 6.21 | 10.13 | 21.00 | 8.65 | 6.64 | 18.61 | 16.41 | 9.34 | 11.10 | 9.46 | 20.01 | 18.59 |
| 1t08 | 1.64 | 39.44 | 33.80 | 41.52 | 15.15 | 46.21 | 32.25 | 29.04 | 32.09 | 19.98 | 32.77 | 44.45 | 13.71 | 23.13 | 32.61 | 35.11 | 48.42 | 23.92 | 23.59 | 43.22 |
| 1t4f | 0.99 | 23.45 | 8.50 | 18.37 | 10.21 | 5.69 | 15.94 | 21.99 | 7.03 | 24.43 | 8.98 | 16.78 | 21.56 | 9.70 | 22.84 | 22.74 | 12.77 | 22.84 | 25.92 | 16.29 |
| 1t7r | 1.20 | 35.86 | 39.90 | 38.78 | 43.70 | 39.42 | 34.26 | 35.35 | 25.47 | 44.26 | 42.91 | 39.02 | 40.57 | 39.72 | 40.04 | 45.86 | 34.15 | 4.15 | 39.19 | 39.15 |
| 1tfc | 1.60 | 31.70 | 14.67 | 19.17 | 35.69 | 27.74 | 29.40 | 19.11 | 31.49 | 29.69 | 24.84 | 25.90 | 29.89 | 13.44 | 39.34 | 18.40 | 22.69 | 5.15 | 28.31 | 38.04 |
| 1u00 | 2.46 | 15.46 | 15.97 | 5.71 | 17.26 | 15.23 | 17.76 | 22.35 | 15.22 | 18.33 | 18.93 | 6.36 | 19.15 | 15.21 | 28.87 | 33.56 | 5.84 | 26.35 | 34.16 | 18.62 |
| 1uj0 | 1.35 | 9.53 | 17.94 | 15.22 | 14.36 | 18.81 | 4.00 | 15.28 | 21.49 | 7.01 | 8.95 | 18.43 | 15.47 | 15.14 | 17.01 | 14.25 | 4.79 | 11.94 | 8.90 | 21.01 |
| 1x2r | 3.84 | 8.27 | 7.85 | 6.42 | 11.07 | 34.65 | 5.82 | 33.42 | 7.92 | 8.18 | 11.28 | 31.85 | 8.69 | 14.72 | 16.19 | 8.29 | 8.15 | 9.97 | 18.09 | 35.16 |
| 1xoc | 1.12 | 24.13 | 28.13 | 26.79 | 27.82 | 24.27 | 27.34 | 13.85 | 27.41 | 26.22 | 25.93 | 25.22 | 23.81 | 24.64 | 28.53 | 31.08 | 27.33 | 24.40 | 23.52 | 20.57 |
| 1ymt | 1.48 | 10.21 | 47.39 | 44.09 | 26.73 | 38.87 | 35.53 | 40.77 | 21.53 | 23.80 | 22.73 | 37.92 | 36.11 | 34.77 | 21.04 | 39.58 | 29.44 | 4.97 | 37.78 | 39.05 |
| 1yuc | 1.17 | 20.13 | 24.13 | 32.44 | 14.47 | 29.45 | 21.28 | 40.69 | 16.61 | 29.96 | 19.38 | 31.69 | 35.73 | 23.94 | 33.85 | 34.43 | 35.49 | 13.06 | 29.66 | 5.58 |
| 1ywo | 1.88 | 17.41 | 13.62 | 20.31 | 17.18 | 4.36 | 17.11 | 21.75 | 17.71 | 13.88 | 15.51 | 7.65 | 19.24 | 6.51 | 23.17 | 16.54 | 16.41 | 16.38 | 18.05 | 14.00 |
| 2a25 | 2.25 | 20.08 | 18.23 | 16.31 | 23.14 | 24.84 | 16.98 | 19.93 | 26.33 | 30.78 | 27.73 | 7.49 | 5.61 | 27.09 | 16.21 | 16.82 | 7.00 | 13.12 | 22.33 | 16.38 |
| 2a3i | 41.93 | 42.55 | 37.25 | 44.46 | 40.33 | 0.80 | 43.29 | 43.42 | 44.16 | 43.71 | 32.02 | 43.00 | 42.32 | 44.99 | 40.07 | 43.57 | 39.39 | 40.51 | 38.40 | 31.13 |
| 2aq9 | 48.74 | 20.72 | 34.73 | 28.00 | 35.47 | 18.55 | 33.95 | 14.15 | 30.77 | 35.49 | 31.12 | 13.46 | 29.98 | 39.06 | 38.83 | 33.10 | 28.12 | 33.13 | 41.04 | 34.91 |
| 2b9h | 29.15 | 2.35 | 29.15 | 40.34 | 21.79 | 26.97 | 43.98 | 43.13 | 25.05 | 44.89 | 49.88 | 31.55 | 31.25 | 37.71 | 16.86 | 22.21 | 50.47 | 51.62 | 16.79 | 17.68 |
| 2bba | 3.42 | 39.21 | 46.18 | 3.45 | 27.50 | 49.21 | 47.44 | 30.13 | 7.15 | 50.04 | 32.74 | 44.71 | 44.40 | 42.66 | 51.33 | 34.18 | 46.41 | 6.68 | 51.91 | 8.49 |
| 2cch | 33.14 | 40.34 | 37.37 | 34.96 | 30.98 | 1.39 | 35.50 | 34.54 | 34.34 | 40.97 | 47.01 | 33.55 | 31.83 | 36.41 | 33.76 | 41.06 | 34.84 | 39.07 | 38.03 | 36.91 |
| 2ce8 | 32.45 | 0.92 | 40.04 | 31.13 | 41.25 | 31.80 | 41.38 | 40.87 | 41.29 | 41.00 | 41.65 | 37.98 | 41.01 | 40.39 | 41.38 | 41.67 | 44.62 | 35.98 | 43.24 | 38.38 |
| 2d0n | 1.47 | 5.61 | 7.82 | 14.10 | 4.94 | 10.23 | 26.94 | 15.50 | 15.42 | 21.20 | 5.91 | 14.14 | 8.73 | 18.50 | 5.97 | 15.21 | 13.02 | 12.29 | 23.64 | 14.23 |
| 2drk | 17.55 | 2.42 | 12.76 | 10.52 | 17.11 | 9.78 | 20.01 | 17.00 | 16.69 | 9.14 | 17.85 | 16.45 | 9.24 | 6.27 | 12.60 | 17.89 | 18.02 | 17.18 | 17.54 | 17.76 |
| 2dyp | 19.96 | 8.44 | 19.42 | 20.25 | 26.78 | 11.01 | 26.22 | 7.88 | 24.16 | 23.00 | 18.11 | 25.79 | 26.12 | 26.26 | 20.81 | 61.25 | 20.58 | 54.55 | 26.16 | 22.45 |
| 2fff | 2.70 | 53.37 | 53.55 | 49.96 | 52.94 | 49.24 | 8.85 | 12.94 | 57.64 | 12.15 | 54.28 | 53.96 | 13.25 | 58.82 | 51.96 | 9.67 | 51.86 | 56.36 | 12.10 | 56.26 |
| 2ffu | 1.64 | 14.15 | 4.49 | 14.87 | 15.84 | 15.77 | 16.37 | 19.54 | 21.37 | 16.74 | 14.88 | 11.02 | 6.33 | 13.75 | 24.14 | 14.65 | 15.90 | 5.90 | 18.28 | 14.71 |
| 2fka | 7.15 | 25.16 | 26.10 | 25.86 | 24.97 | 11.57 | 24.62 | 14.57 | 29.82 | 28.30 | 28.03 | 30.62 | 11.91 | 28.88 | 28.03 | 8.37 | 10.75 | 28.41 | 33.25 | 27.07 |
| 2fmf | 5.50 | 10.40 | 0.85 | 11.50 | 27.03 | 26.04 | 8.69 | 14.00 | 10.15 | 5.32 | 27.60 | 10.24 | 28.04 | 24.30 | 28.03 | 30.44 | 28.07 | 31.68 | 11.55 | 26.12 |
| 2fts | 27.70 | 1.30 | 21.97 | 18.54 | 48.28 | 5.89 | 25.93 | 34.55 | 18.16 | 19.78 | 6.24 | 33.82 | 15.03 | 35.91 | 14.83 | 19.94 | 5.06 | 15.94 | 10.92 | 29.82 |
| 2fvj | 42.15 | 1.45 | 34.61 | 46.47 | 42.14 | 22.57 | 46.75 | 41.99 | 46.43 | 44.68 | 46.00 | 24.57 | 44.08 | 43.69 | 50.28 | 22.69 | 46.50 | 46.49 | 43.12 | 45.29 |
| 2ho2 | 16.99 | 11.34 | 14.37 | 6.57 | 6.54 | 17.79 | 14.48 | 7.73 | 18.65 | 15.81 | 10.35 | 18.18 | 18.69 | 14.95 | 11.78 | 11.64 | 20.15 | 9.96 | 7.62 | 8.59 |
| 2ht9 | 30.45 | 22.53 | 25.76 | 21.71 | 28.04 | 24.63 | 23.85 | 19.11 | 12.96 | 21.92 | 22.19 | 21.10 | 4.09 | 28.45 | 7.67 | 28.96 | 25.61 | 27.88 | 22.13 | 23.53 |
| 2o02 | 20.50 | 28.30 | 21.78 | 2.47 | 16.11 | 20.41 | 23.71 | 27.36 | 32.38 | 23.56 | 34.02 | 25.32 | 22.30 | 23.95 | 26.85 | 23.56 | 7.50 | 21.43 | 34.81 | 22.96 |
| 2o4j | 30.10 | 29.62 | 37.90 | 22.06 | 24.78 | 23.53 | 19.62 | 40.62 | 36.09 | 33.96 | 35.36 | 32.98 | 40.08 | 30.86 | 33.84 | 31.77 | 29.13 | 20.85 | 33.81 | 38.29 |
| 2o9v | 1.95 | 16.62 | 15.07 | 9.69 | 5.85 | 17.54 | 20.86 | 31.35 | 18.09 | 16.43 | 19.56 | 26.11 | 11.55 | 32.88 | 18.28 | 9.82 | 17.37 | 17.32 | 18.26 | 5.76 |
| 2oei | 17.90 | 10.89 | 6.67 | 15.27 | 19.32 | 1.53 | 16.83 | 3.89 | 16.02 | 15.40 | 8.54 | 22.76 | 17.78 | 17.10 | 24.30 | 11.05 | 19.37 | 15.16 | 21.12 | 15.47 |
| 2p0w | 1.17 | 27.23 | 39.31 | 13.08 | 17.23 | 20.46 | 31.82 | 21.83 | 32.24 | 28.70 | 18.42 | 17.64 | 20.88 | 23.62 | 23.38 | 33.64 | 41.47 | 36.86 | 17.08 | 25.58 |
| 2p1o | 7.23 | 13.36 | 3.21 | 11.23 | 14.14 | 10.14 | 10.97 | 11.93 | 8.47 | 3.75 | 10.49 | 15.92 | 11.12 | 11.86 | 11.54 | 11.23 | 11.55 | 7.90 | 13.99 | 39.28 |
| 2p1t | 38.00 | 38.49 | 22.36 | 33.68 | 38.68 | 39.88 | 39.82 | 26.19 | 26.49 | 37.55 | 27.28 | 25.97 | 40.97 | 32.80 | 36.34 | 41.20 | 39.17 | 28.16 | 38.45 | 35.61 |
| 2p54 | 0.77 | 44.95 | 45.91 | 45.63 | 43.16 | 46.71 | 43.81 | 24.35 | 43.58 | 41.07 | 22.92 | 43.78 | 26.65 | 44.42 | 42.16 | 43.87 | 26.85 | 32.88 | 48.95 | 22.71 |
| 2peh | 0.98 | 5.49 | 33.45 | 32.40 | 5.56 | 10.23 | 30.71 | 37.71 | 26.90 | 13.18 | 28.25 | 29.89 | 9.94 | 18.45 | 23.11 | 10.25 | 26.89 | 36.04 | 30.96 | 28.51 |
| 2pux | 1.36 | 45.91 | 34.35 | 32.02 | 36.21 | 34.14 | 48.75 | 30.91 | 31.07 | 32.80 | 42.77 | 29.35 | 29.68 | 47.82 | 43.67 | 41.60 | 49.85 | 31.88 | 32.48 | 38.74 |
| 2puy | 2.38 | 29.57 | 22.88 | 21.54 | 20.94 | 18.84 | 20.22 | 20.39 | 21.41 | 24.11 | 18.21 | 28.55 | 27.97 | 12.01 | 12.98 | 16.19 | 15.28 | 28.05 | 21.81 | 21.61 |
| 2qbx | 15.72 | 2.88 | 37.93 | 18.61 | 36.70 | 15.95 | 8.81 | 44.97 | 8.77 | 24.03 | 37.79 | 39.62 | 22.12 | 36.04 | 44.44 | 39.78 | 42.77 | 40.40 | 39.08 | 6.20 |
| 2qos | 1.43 | 5.86 | 6.90 | 18.02 | 19.28 | 19.77 | 24.97 | 6.55 | 6.97 | 26.41 | 19.50 | 11.69 | 5.41 | 27.23 | 11.64 | 28.34 | 26.59 | 9.77 | 9.02 | 7.98 |
| 2qse | 1.37 | 31.95 | 29.38 | 31.87 | 31.28 | 35.66 | 28.19 | 17.98 | 31.89 | 31.57 | 32.98 | 31.31 | 34.60 | 35.13 | 29.87 | 11.16 | 33.70 | 34.74 | 31.53 | 34.86 |
| 2r7g | 1.07 | 19.96 | 21.65 | 27.14 | 18.48 | 37.30 | 28.04 | 16.17 | 31.41 | 33.48 | 48.75 | 33.28 | 39.87 | 39.62 | 40.19 | 30.59 | 46.63 | 46.20 | 8.30 | 46.92 |
| 2r9q | 4.50 | 10.70 | 8.30 | 5.49 | 7.83 | 10.19 | 29.67 | 11.56 | 29.08 | 8.24 | 11.53 | 11.28 | 34.99 | 9.49 | 8.51 | 5.42 | 6.51 | 30.56 | 28.34 | 8.92 |
| 2v8y | 2.38 | 9.27 | 16.34 | 16.78 | 14.71 | 14.83 | 17.26 | 5.93 | 3.86 | 37.85 | 13.78 | 15.28 | 17.18 | 15.05 | 15.61 | 8.82 | 8.62 | 39.02 | 12.21 | 6.32 |
| 2vkn | 3.01 | 21.52 | 9.67 | 15.45 | 21.47 | 21.27 | 17.66 | 16.48 | 17.07 | 22.39 | 21.66 | 25.58 | 5.61 | 19.76 | 14.58 | 25.96 | 15.44 | 22.60 | 24.07 | 10.39 |
| 2vr3 | 27.47 | 2.35 | 12.29 | 23.40 | 23.20 | 26.99 | 28.68 | 35.75 | 22.52 | 33.54 | 46.30 | 31.20 | 31.66 | 33.53 | 25.20 | 10.55 | 24.33 | 40.57 | 34.61 | 21.75 |
| 2vwf | 1.25 | 30.93 | 25.62 | 24.49 | 27.41 | 28.01 | 32.30 | 31.60 | 25.86 | 12.03 | 8.31 | 31.51 | 31.37 | 9.92 | 8.46 | 26.94 | 34.46 | 29.28 | 18.68 | 34.49 |
| 2w2u | 0.86 | 6.84 | 21.07 | 16.03 | 21.36 | 16.77 | 15.39 | 15.71 | 21.73 | 11.86 | 10.92 | 10.60 | 21.24 | 15.57 | 13.87 | 26.27 | 6.98 | 10.00 | 15.23 | 18.61 |
| 2whx | 1.57 | 25.27 | 25.56 | 59.07 | 25.92 | 26.23 | 26.99 | 25.92 | 29.45 | 25.02 | 30.06 | 43.88 | 18.42 | 25.53 | 27.09 | 6.60 | 54.03 | 30.69 | 28.50 | 25.55 |
| 2xrw | 1.09 | 47.69 | 44.15 | 36.21 | 43.67 | 38.20 | 41.54 | 18.81 | 22.31 | 44.27 | 31.52 | 5.78 | 50.06 | 40.27 | 24.33 | 29.63 | 22.29 | 32.68 | 17.75 | 48.45 |
| 2xu7 | 1.91 | 6.22 | 7.43 | 5.71 | 38.09 | 7.00 | 14.64 | 14.42 | 36.64 | 27.40 | 4.03 | 7.05 | 33.34 | 8.72 | 34.67 | 24.99 | 20.11 | 14.95 | 25.89 | 17.63 |
| 2xvc | 1.26 | 8.13 | 21.46 | 23.47 | 20.50 | 24.33 | 21.24 | 21.48 | 21.01 | 26.28 | 24.40 | 29.28 | 34.75 | 23.35 | 21.93 | 28.47 | 24.45 | 8.84 | 5.51 | 23.31 |
| 2zjd | 30.96 | 31.65 | 1.30 | 32.45 | 32.62 | 29.70 | 17.67 | 33.49 | 32.68 | 10.61 | 33.92 | 30.31 | 31.00 | 35.05 | 29.09 | 31.89 | 35.98 | 28.75 | 36.25 | 35.32 |
| 3asl | 24.70 | 21.78 | 0.75 | 23.83 | 26.24 | 25.13 | 25.83 | 21.64 | 23.83 | 25.12 | 26.66 | 14.60 | 21.84 | 25.75 | 26.94 | 21.01 | 25.20 | 25.15 | 27.76 | 27.01 |
| 3awr | 24.47 | 21.96 | 22.91 | 12.99 | 26.44 | 11.33 | 21.23 | 19.17 | 24.98 | 24.52 | 25.21 | 23.82 | 11.74 | 25.16 | 12.66 | 10.32 | 3.38 | 9.50 | 22.63 | 7.73 |
| 3ayu | 1.29 | 7.88 | 16.35 | 17.41 | 7.53 | 17.34 | 12.43 | 16.44 | 17.25 | 17.52 | 8.53 | 16.34 | 19.19 | 16.94 | 18.45 | 21.49 | 12.26 | 16.99 | 21.19 | 34.32 |
| 3bfq | 0.89 | 27.51 | 13.96 | 28.11 | 37.22 | 11.09 | 28.96 | 31.78 | 27.61 | 30.42 | 33.13 | 28.07 | 32.89 | 8.97 | 16.70 | 5.00 | 32.37 | 29.91 | 34.52 | 15.88 |
| 3c3r | 1.17 | 51.96 | 42.06 | 42.71 | 25.17 | 13.44 | 39.58 | 23.09 | 38.19 | 11.80 | 38.86 | 5.76 | 53.17 | 7.09 | 26.62 | 32.65 | 15.88 | 40.76 | 8.55 | 47.65 |
| 3d32 | 20.77 | 1.55 | 8.41 | 18.88 | 23.39 | 25.77 | 15.94 | 16.00 | 26.77 | 28.52 | 30.36 | 33.56 | 8.16 | 32.88 | 29.69 | 23.70 | 18.45 | 25.69 | 24.02 | 26.83 |
| 3ds4 | 1.54 | 11.38 | 24.80 | 11.04 | 26.04 | 22.27 | 26.49 | 23.12 | 22.36 | 24.28 | 31.21 | 24.48 | 25.27 | 27.86 | 11.11 | 29.44 | 12.23 | 24.25 | 7.13 | 24.39 |
| 3ery | 1.41 | 7.92 | 15.13 | 15.33 | 5.74 | 11.59 | 14.65 | 10.47 | 8.84 | 7.94 | 9.52 | 5.87 | 15.26 | 12.37 | 15.38 | 19.19 | 18.14 | 17.38 | 14.15 | 16.10 |
| 3fdo | 0.61 | 17.83 | 12.04 | 19.18 | 6.45 | 11.87 | 12.89 | 21.74 | 10.02 | 15.55 | 13.93 | 16.77 | 10.72 | 16.42 | 20.96 | 18.19 | 18.50 | 18.93 | 11.75 | 5.92 |
| 3g2s | 1.58 | 31.69 | 27.08 | 14.40 | 17.28 | 11.74 | 39.16 | 20.84 | 19.43 | 19.04 | 24.77 | 27.05 | 12.28 | 18.42 | 31.12 | 28.41 | 8.85 | 20.34 | 21.85 | 29.53 |
| 3gyt | 0.70 | 43.25 | 13.10 | 40.94 | 44.84 | 47.00 | 15.40 | 6.60 | 31.64 | 43.09 | 5.06 | 8.52 | 6.91 | 6.31 | 11.07 | 15.15 | 9.41 | 16.60 | 4.76 | 12.18 |
| 3h1z | 36.70 | 22.03 | 22.90 | 21.20 | 45.99 | 30.96 | 29.47 | 34.62 | 25.15 | 24.63 | 13.92 | 56.92 | 19.46 | 24.79 | 30.45 | 24.96 | 28.71 | 29.41 | 26.91 | 27.08 |
| 3i5r | 15.61 | 0.68 | 17.02 | 17.10 | 12.69 | 13.18 | 15.40 | 15.88 | 7.79 | 13.34 | 15.88 | 7.13 | 9.73 | 16.42 | 12.96 | 17.20 | 15.99 | 18.95 | 15.43 | 19.45 |
| 3ivv | 20.54 | 1.22 | 8.96 | 20.32 | 23.18 | 22.63 | 22.80 | 28.94 | 19.75 | 16.71 | 10.56 | 17.32 | 20.03 | 17.44 | 7.28 | 20.79 | 18.90 | 15.37 | 25.14 | 17.61 |
| 3kmr | 38.44 | 26.92 | 34.50 | 37.79 | 37.11 | 3.68 | 24.61 | 41.00 | 35.16 | 32.61 | 36.84 | 20.61 | 33.23 | 35.96 | 39.89 | 35.14 | 23.51 | 37.59 | 29.63 | 20.67 |
| 3kuj | 21.57 | 4.55 | 18.79 | 19.58 | 22.28 | 24.66 | 19.71 | 16.77 | 24.74 | 28.44 | 7.27 | 21.66 | 14.88 | 28.20 | 21.22 | 22.15 | 25.35 | 26.39 | 20.61 | 23.43 |
| 3kus | 2.06 | 23.77 | 19.42 | 20.25 | 24.67 | 24.86 | 24.23 | 4.65 | 26.51 | 21.08 | 17.38 | 22.67 | 18.58 | 23.79 | 18.05 | 24.39 | 19.81 | 24.03 | 24.31 | 27.08 |
| 3l0e | 19.59 | 39.90 | 26.30 | 29.55 | 31.40 | 24.27 | 22.13 | 41.18 | 33.57 | 26.71 | 25.42 | 26.54 | 33.46 | 38.04 | 22.37 | 38.24 | 25.95 | 39.76 | 23.96 | 26.31 |
| 3ll8 | 46.18 | 42.01 | 37.90 | 49.27 | 49.84 | 48.20 | 40.88 | 55.53 | 38.64 | 46.40 | 47.15 | 46.44 | 40.95 | 41.90 | 49.30 | 39.08 | 40.35 | 45.91 | 54.25 | 51.27 |
| 3llz | 2.52 | 21.21 | 24.98 | 9.62 | 7.61 | 25.51 | 30.13 | 9.92 | 31.25 | 23.80 | 23.61 | 16.63 | 37.43 | 23.01 | 19.77 | 36.29 | 18.92 | 23.94 | 28.18 | 30.28 |
| 3obq | 1.83 | 17.22 | 19.31 | 17.79 | 13.73 | 5.41 | 15.36 | 8.85 | 15.39 | 5.65 | 6.21 | 34.78 | 14.37 | 23.03 | 22.93 | 28.66 | 27.25 | 14.30 | 8.57 | 14.40 |
| 3olf | 37.89 | 34.95 | 38.14 | 34.11 | 15.16 | 35.75 | 36.44 | 22.29 | 23.63 | 23.74 | 26.96 | 34.70 | 36.60 | 44.82 | 23.49 | 35.97 | 24.08 | 28.11 | 35.92 | 26.80 |
| 3p72 | 6.38 | 6.63 | 36.59 | 34.79 | 34.15 | 9.10 | 35.91 | 34.09 | 38.15 | 35.85 | 34.23 | 33.71 | 36.54 | 33.40 | 9.81 | 38.36 | 36.03 | 35.24 | 32.65 | 33.97 |
| 3p8f | 1.15 | 13.95 | 7.02 | 13.96 | 7.07 | 9.48 | 14.48 | 7.63 | 11.74 | 23.91 | 14.13 | 16.11 | 14.51 | 11.18 | 13.62 | 8.10 | 14.78 | 20.93 | 15.78 | 14.14 |
| 3ptl | 22.41 | 37.72 | 20.84 | 17.74 | 20.96 | 22.88 | 23.32 | 45.62 | 17.21 | 34.17 | 20.22 | 20.34 | 26.13 | 21.33 | 36.39 | 22.70 | 19.46 | 17.51 | 15.13 | 33.35 |
| 3qis | 13.84 | 13.91 | 5.57 | 13.80 | 15.07 | 10.27 | 5.97 | 13.38 | 53.35 | 44.51 | 9.08 | 5.46 | 60.63 | 58.32 | 13.18 | 15.42 | 13.71 | 11.97 | 44.25 | 12.23 |
| 3rm1 | 1.24 | 19.59 | 29.48 | 19.31 | 18.90 | 18.89 | 32.42 | 19.78 | 24.42 | 9.34 | 17.33 | 29.08 | 30.41 | 18.63 | 21.79 | 31.50 | 29.74 | 19.71 | 20.62 | 11.96 |
| 3rqg | 37.95 | 32.35 | 11.72 | 35.12 | 46.69 | 32.23 | 35.05 | 31.79 | 1.07 | 34.60 | 31.89 | 12.15 | 38.63 | 42.15 | 12.27 | 42.59 | 43.85 | 44.03 | 36.64 | 30.53 |
| 3sfj | 14.96 | 3.44 | 24.50 | 26.67 | 27.63 | 22.78 | 28.17 | 15.81 | 32.28 | 23.16 | 25.36 | 14.22 | 14.37 | 29.35 | 2.88 | 24.43 | 26.57 | 23.98 | 8.85 | 27.56 |
| 3so6 | 1.78 | 21.61 | 15.55 | 21.60 | 8.37 | 8.68 | 15.07 | 11.07 | 6.13 | 13.37 | 19.38 | 18.03 | 30.87 | 21.63 | 17.15 | 15.66 | 13.40 | 18.05 | 19.44 | 26.20 |
| 3tjv | 1.15 | 15.31 | 12.29 | 18.34 | 9.10 | 17.44 | 22.43 | 20.38 | 28.32 | 31.90 | 11.87 | 5.20 | 17.81 | 26.90 | 15.36 | 11.68 | 25.70 | 28.81 | 17.79 | 13.25 |
| 3tzy | 4.16 | 8.39 | 9.65 | 9.21 | 9.99 | 10.19 | 9.28 | 14.98 | 38.11 | 7.82 | 46.00 | 26.40 | 8.38 | 33.50 | 26.15 | 16.28 | 26.18 | 29.23 | 45.36 | 40.93 |
| 3u9q | 0.92 | 21.21 | 26.07 | 5.32 | 23.90 | 5.07 | 8.55 | 43.29 | 7.13 | 26.17 | 27.21 | 42.36 | 27.02 | 21.62 | 25.52 | 7.29 | 46.92 | 14.15 | 17.83 | 13.78 |
| 3up3 | 0.98 | 20.47 | 5.59 | 45.73 | 6.31 | 8.13 | 11.18 | 31.97 | 20.43 | 8.22 | 18.17 | 6.90 | 21.66 | 6.83 | 34.38 | 7.93 | 19.94 | 40.94 | 29.68 | 35.18 |
| 3v2x | 18.08 | 1.23 | 6.60 | 8.92 | 17.20 | 17.74 | 27.72 | 23.25 | 25.04 | 10.66 | 15.46 | 12.31 | 26.97 | 28.59 | 19.19 | 31.77 | 15.54 | 27.38 | 27.88 | 32.62 |
| 3vtc | 31.61 | 34.00 | 22.83 | 41.34 | 3.45 | 33.02 | 30.57 | 10.49 | 30.98 | 38.25 | 33.35 | 32.44 | 41.61 | 21.78 | 30.39 | 36.64 | 30.14 | 42.27 | 40.57 | 25.15 |
| 3w1b | 1.51 | 91.09 | 69.82 | 87.03 | 87.11 | 27.71 | 11.22 | 83.76 | 36.77 | 68.28 | 83.79 | 72.03 | 45.30 | 80.12 | 72.03 | 87.22 | 85.49 | 71.28 | 39.42 | 47.06 |
| 3zqh | 34.86 | 37.26 | 28.51 | 25.18 | 32.03 | 28.79 | 36.43 | 28.60 | 28.14 | 35.98 | 30.64 | 39.12 | 36.10 | 38.70 | 32.69 | 14.53 | 38.18 | 39.81 | 36.56 | 24.50 |
| 4b4n | 28.96 | 30.45 | 28.69 | 29.59 | 29.23 | 29.87 | 28.10 | 32.52 | 34.54 | 29.47 | 30.64 | 30.60 | 30.09 | 29.00 | 30.25 | 27.05 | 28.24 | 26.34 | 27.56 | 32.02 |
| 4dcb | 51.03 | 55.96 | 1.19 | 54.58 | 53.35 | 50.59 | 12.89 | 10.86 | 49.84 | 57.14 | 54.52 | 52.41 | 49.94 | 51.11 | 49.69 | 53.10 | 12.81 | 43.22 | 58.60 | 43.28 |
| 4e34 | 14.36 | 1.80 | 16.46 | 15.98 | 8.18 | 15.07 | 16.15 | 18.73 | 16.35 | 24.04 | 14.82 | 17.60 | 24.14 | 17.19 | 20.30 | 27.64 | 8.53 | 22.90 | 29.61 | 12.33 |
| 4eik | 0.79 | 9.05 | 10.05 | 17.30 | 18.12 | 10.71 | 12.32 | 16.95 | 7.01 | 20.98 | 21.99 | 15.54 | 16.25 | 12.14 | 16.04 | 18.07 | 19.98 | 17.33 | 23.89 | 15.76 |
| 4ery | 2.33 | 10.99 | 14.39 | 9.64 | 12.57 | 13.82 | 13.35 | 12.01 | 9.72 | 12.43 | 11.37 | 13.80 | 5.42 | 14.76 | 12.36 | 10.91 | 8.60 | 12.52 | 14.60 | 8.24 |
| 4f14 | 10.36 | 2.27 | 19.81 | 21.04 | 23.12 | 25.20 | 20.10 | 14.31 | 6.77 | 18.59 | 5.68 | 18.74 | 14.77 | 9.30 | 23.91 | 18.54 | 6.94 | 10.25 | 3.58 | 9.53 |
| 4f1z | 29.00 | 29.89 | 26.10 | 32.05 | 34.84 | 33.18 | 16.24 | 39.03 | 31.35 | 30.05 | 33.55 | 42.32 | 22.37 | 11.74 | 26.20 | 38.25 | 26.22 | 27.23 | 45.80 | 30.58 |
| 4gq6 | 10.45 | 13.71 | 1.86 | 7.95 | 14.95 | 9.25 | 11.85 | 12.61 | 12.96 | 12.75 | 11.24 | 26.79 | 7.22 | 9.80 | 9.07 | 32.50 | 13.46 | 25.24 | 28.64 | 23.35 |
| 4gxl | 26.71 | 29.68 | 2.05 | 24.27 | 29.13 | 17.20 | 30.89 | 28.74 | 12.55 | 40.07 | 30.56 | 37.17 | 21.12 | 28.59 | 17.90 | 27.98 | 9.22 | 22.81 | 18.39 | 35.99 |
| 4gyw | 6.20 | 1.78 | 22.93 | 16.95 | 42.54 | 22.90 | 21.09 | 7.92 | 16.38 | 19.90 | 22.75 | 20.30 | 20.71 | 40.81 | 19.96 | 15.49 | 24.28 | 11.88 | 30.53 | 21.38 |
| 4h4f | 37.99 | 17.08 | 32.95 | 35.17 | 33.01 | 11.41 | 1.49 | 22.97 | 8.50 | 33.66 | 13.28 | 21.57 | 27.82 | 16.24 | 30.61 | 22.46 | 24.82 | 20.67 | 17.90 | 31.27 |
| 4hom | 8.30 | 38.07 | 22.36 | 43.24 | 11.48 | 12.56 | 16.69 | 16.17 | 37.54 | 28.30 | 34.14 | 17.60 | 32.16 | 13.12 | 13.45 | 13.83 | 36.27 | 21.13 | 34.18 | 16.91 |
| 4htp | 2.16 | 38.31 | 36.59 | 48.55 | 33.47 | 32.49 | 46.49 | 40.73 | 15.50 | 24.02 | 34.21 | 23.77 | 30.00 | 35.88 | 24.34 | 9.83 | 38.76 | 47.96 | 34.59 | 27.57 |
| 4iim | 2.88 | 19.76 | 11.58 | 23.95 | 23.75 | 6.73 | 24.89 | 23.34 | 18.21 | 26.50 | 15.47 | 23.60 | 19.31 | 22.89 | 18.18 | 28.84 | 24.09 | 21.45 | 21.98 | 9.99 |
| 4j8s | 34.58 | 2.43 | 30.20 | 31.40 | 28.63 | 38.77 | 28.41 | 12.23 | 35.79 | 27.46 | 39.68 | 29.18 | 26.42 | 35.10 | 27.85 | 33.40 | 27.45 | 31.41 | 32.24 | 45.25 |
| 4k0u | 2.00 | 9.96 | 13.61 | 8.01 | 15.25 | 15.85 | 15.43 | 29.15 | 24.30 | 13.81 | 15.72 | 10.87 | 11.73 | 24.07 | 12.28 | 26.48 | 15.32 | 11.13 | 28.72 | 19.86 |

**S5(a). I-RMSD values of all 20 poses obtained after blind docking by ATTRACT on 133 protein-peptide complexes.**

| **ID** | **Pose1** | **Pose2** | **Pose3** | **Pose4** | **Pose5** | **Pose6** | **Pose7** | **Pose8** | **Pose9** | **Pose10** | **Pose11** | **Pose12** | **Pose13** | **Pose14** | **Pose15** | **Pose16** | **Pose17** | **Pose18** | **Pose19** | **Pose20** |
| --- | --- | --- | --- | --- | --- | --- | --- | --- | --- | --- | --- | --- | --- | --- | --- | --- | --- | --- | --- | --- |
| 1cjr | 19.83 | 24.77 | 19.61 | 27.94 | 24.78 | 19.68 | 19.53 | 20.16 | 20.07 | 25.35 | 20.77 | 27.48 | 18.46 | 27.48 | 14.72 | 20.27 | 16.75 | 14.57 | 27.35 | 27.03 |
| 1cka | 3.35 | 3.35 | 3.49 | 3.24 | 3.40 | 3.54 | 3.70 | 3.47 | 1.43 | 1.34 | 1.23 | 3.54 | 1.43 | 3.83 | 0.82 | 13.11 | 2.69 | 7.64 | 13.31 | 12.96 |
| 1cvu | 53.94 | 33.60 | 33.47 | 53.56 | 53.78 | 42.82 | 35.38 | 33.35 | 29.37 | 55.01 | 33.32 | 35.57 | 48.49 | 39.96 | 53.84 | 56.91 | 49.86 | 54.06 | 46.25 | 53.52 |
| 1d4t | 0.63 | 31.10 | 23.63 | 19.32 | 24.40 | 19.71 | 26.60 | 31.03 | 22.36 | 20.01 | 22.23 | 17.94 | 18.85 | 21.53 | 25.25 | 22.13 | 23.99 | 21.87 | 27.33 | 23.73 |
| 1eg4 | 40.86 | 35.18 | 15.77 | 12.14 | 16.87 | 30.40 | 34.19 | 17.51 | 15.84 | 38.62 | 25.47 | 41.81 | 24.61 | 19.64 | 30.17 | 29.39 | 7.89 | 40.26 | 14.34 | 12.74 |
| 1h6w | 40.66 | 15.34 | 12.33 | 20.00 | 20.05 | 46.96 | 16.29 | 19.95 | 46.41 | 13.11 | 13.99 | 46.47 | 46.02 | 18.09 | 13.95 | 40.06 | 13.19 | 32.81 | 36.49 | 36.57 |
| 1hc9 | 11.93 | 11.48 | 9.38 | 7.79 | 18.60 | 10.34 | 11.78 | 10.09 | 20.09 | 9.95 | 19.09 | 19.48 | 18.11 | 20.23 | 22.79 | 26.11 | 18.09 | 6.52 | 21.07 | 18.15 |
| 1jbu | 1.02 | 1.03 | 4.70 | 10.68 | 4.71 | 10.61 | 10.40 | 44.46 | 4.82 | 44.45 | 43.24 | 45.05 | 17.32 | 44.52 | 17.47 | 45.66 | 45.16 | 16.93 | 11.72 | 16.40 |
| 1k5n | 10.76 | 13.46 | 21.57 | 24.39 | 13.99 | 23.39 | 23.22 | 12.51 | 24.81 | 15.15 | 60.42 | 60.45 | 13.89 | 20.83 | 60.53 | 14.04 | 25.40 | 13.72 | 13.97 | 14.79 |
| 1mfg | 13.32 | 12.38 | 12.51 | 20.46 | 11.23 | 12.84 | 18.86 | 11.26 | 20.58 | 14.00 | 19.12 | 18.81 | 19.29 | 19.06 | 11.74 | 14.57 | 19.01 | 19.39 | 19.38 | 18.75 |
| 1nln | 0.92 | 3.34 | 6.67 | 6.56 | 3.24 | 9.12 | 5.07 | 15.24 | 15.04 | 15.04 | 15.08 | 15.07 | 15.39 | 15.58 | 15.86 | 3.58 | 9.95 | 9.03 | 9.99 | 18.53 |
| 1nq7 | 34.00 | 33.80 | 34.07 | 33.18 | 33.15 | 33.99 | 33.83 | 33.76 | 33.25 | 32.77 | 32.74 | 35.36 | 33.26 | 26.36 | 26.52 | 34.89 | 21.80 | 26.64 | 1.41 | 38.98 |
| 1ntv | 13.44 | 13.51 | 21.23 | 30.26 | 28.80 | 1.49 | 19.31 | 31.93 | 31.89 | 31.86 | 25.72 | 13.52 | 26.83 | 15.07 | 29.12 | 16.97 | 31.73 | 19.84 | 20.42 | 20.14 |
| 1nx1 | 34.46 | 34.57 | 34.79 | 34.99 | 34.79 | 35.46 | 36.93 | 36.37 | 35.94 | 37.16 | 35.41 | 7.88 | 36.76 | 38.55 | 2.54 | 6.75 | 3.00 | 3.03 | 36.72 | 37.61 |
| 1oai | 22.37 | 22.05 | 23.49 | 23.41 | 6.02 | 6.14 | 23.52 | 9.72 | 10.41 | 11.71 | 10.62 | 11.60 | 2.83 | 5.82 | 14.98 | 5.05 | 19.86 | 5.18 | 18.01 | 19.93 |
| 1oj5 | 13.14 | 25.97 | 25.92 | 1.02 | 26.15 | 26.02 | 24.39 | 25.24 | 24.21 | 24.09 | 26.29 | 16.56 | 29.72 | 16.72 | 16.44 | 24.85 | 25.46 | 29.77 | 29.75 | 25.17 |
| 1ou8 | 11.21 | 15.52 | 10.46 | 27.11 | 13.33 | 12.33 | 13.25 | 24.68 | 13.11 | 13.11 | 24.72 | 11.46 | 22.16 | 12.63 | 19.03 | 12.65 | 22.14 | 28.58 | 9.92 | 6.31 |
| 1ow6 | 27.08 | 27.31 | 27.45 | 27.16 | 27.36 | 28.93 | 11.03 | 11.29 | 11.87 | 11.41 | 11.82 | 28.64 | 11.02 | 28.90 | 12.28 | 11.56 | 28.95 | 11.94 | 12.34 | 16.54 |
| 1pzl | 2.05 | 2.36 | 3.25 | 3.57 | 36.14 | 8.31 | 4.07 | 34.10 | 8.26 | 5.77 | 33.51 | 33.82 | 31.81 | 7.79 | 28.61 | 33.75 | 32.06 | 37.30 | 32.44 | 37.33 |
| 1qkz | 8.54 | 10.10 | 6.70 | 9.69 | 8.73 | 8.75 | 8.08 | 9.54 | 6.73 | 9.62 | 8.68 | 11.63 | 11.55 | 9.27 | 10.50 | 10.33 | 11.51 | 10.79 | 11.39 | 12.02 |
| 1rst | 8.20 | 7.55 | 7.17 | 7.36 | 36.46 | 36.45 | 36.76 | 8.26 | 8.28 | 35.59 | 35.66 | 35.59 | 4.78 | 2.65 | 7.58 | 35.09 | 3.34 | 10.16 | 2.64 | 7.37 |
| 1rxz | 1.25 | 5.31 | 16.56 | 4.66 | 6.07 | 18.04 | 16.61 | 17.20 | 6.24 | 6.56 | 16.04 | 6.28 | 7.93 | 10.37 | 9.07 | 24.28 | 10.38 | 10.14 | 16.45 | 8.91 |
| 1sfi | 1.76 | 21.00 | 18.06 | 18.22 | 15.29 | 13.13 | 12.56 | 33.50 | 13.16 | 21.16 | 25.78 | 12.64 | 14.70 | 25.81 | 7.40 | 33.86 | 14.53 | 19.14 | 24.66 | 37.56 |
| 1ssh | 1.23 | 1.36 | 1.10 | 1.61 | 1.12 | 1.11 | 1.90 | 1.39 | 3.09 | 5.92 | 5.60 | 3.87 | 5.06 | 20.35 | 5.21 | 6.51 | 4.08 | 6.31 | 4.87 | 6.65 |
| 1t08 | 35.98 | 27.75 | 27.82 | 23.41 | 23.42 | 37.00 | 34.31 | 48.72 | 36.90 | 58.78 | 28.83 | 45.82 | 48.72 | 58.72 | 33.96 | 36.60 | 38.47 | 43.16 | 58.58 | 41.38 |
| 1t4f | 1.01 | 1.66 | 1.03 | 1.23 | 1.64 | 0.79 | 8.10 | 1.92 | 2.65 | 8.24 | 4.99 | 5.78 | 11.81 | 3.19 | 5.16 | 3.82 | 4.25 | 4.14 | 9.41 | 6.80 |
| 1t7r | 1.75 | 1.64 | 2.50 | 3.04 | 29.20 | 29.20 | 29.26 | 10.05 | 26.97 | 6.77 | 33.77 | 32.28 | 21.24 | 40.96 | 38.92 | 3.77 | 38.98 | 5.77 | 40.80 | 33.21 |
| 1tfc | 32.73 | 34.77 | 33.42 | 32.20 | 32.26 | 32.45 | 36.33 | 32.34 | 32.02 | 32.00 | 34.59 | 34.60 | 32.08 | 35.94 | 31.85 | 35.81 | 34.18 | 4.24 | 2.31 | 4.21 |
| 1u00 | 18.93 | 16.01 | 25.07 | 19.87 | 25.20 | 16.97 | 20.69 | 17.14 | 25.31 | 29.18 | 14.66 | 25.05 | 29.38 | 29.07 | 29.25 | 29.02 | 29.14 | 28.93 | 28.99 | 28.22 |
| 1uj0 | 1.23 | 1.06 | 1.04 | 7.77 | 7.71 | 7.83 | 1.55 | 7.99 | 13.69 | 15.25 | 17.25 | 17.35 | 15.07 | 14.84 | 2.93 | 8.42 | 3.50 | 21.96 | 4.01 | 1.70 |
| 1x2r | 7.36 | 7.33 | 4.47 | 7.32 | 3.58 | 7.40 | 32.16 | 1.59 | 3.00 | 31.53 | 6.46 | 8.59 | 6.61 | 6.10 | 6.51 | 37.72 | 7.58 | 32.98 | 31.99 | 7.58 |
| 1xoc | 25.10 | 26.00 | 22.82 | 34.15 | 19.54 | 34.23 | 25.16 | 25.04 | 25.13 | 24.79 | 25.66 | 25.60 | 24.80 | 36.92 | 20.45 | 25.79 | 25.71 | 28.61 | 40.36 | 28.13 |
| 1ymt | 3.12 | 5.56 | 5.25 | 5.83 | 1.95 | 5.15 | 1.47 | 1.31 | 1.93 | 1.36 | 30.74 | 1.24 | 4.92 | 5.43 | 5.28 | 4.06 | 4.82 | 36.62 | 29.99 | 29.95 |
| 1yuc | 20.09 | 27.70 | 27.82 | 35.56 | 26.04 | 27.97 | 37.00 | 33.02 | 26.33 | 1.49 | 1.13 | 15.30 | 0.89 | 1.31 | 20.49 | 27.04 | 27.93 | 1.48 | 33.34 | 34.05 |
| 1ywo | 15.03 | 15.04 | 15.04 | 17.39 | 15.05 | 17.37 | 15.01 | 15.06 | 1.48 | 15.07 | 15.19 | 14.99 | 15.26 | 15.25 | 11.78 | 15.12 | 1.82 | 11.76 | 11.68 | 11.70 |
| 2a25 | 24.92 | 17.17 | 35.18 | 31.13 | 31.31 | 26.39 | 31.08 | 25.24 | 26.03 | 25.08 | 2.07 | 23.62 | 34.21 | 24.57 | 27.94 | 25.04 | 9.42 | 31.05 | 24.44 | 16.33 |
| 2a3i | 1.30 | 1.11 | 1.14 | 1.10 | 2.20 | 2.11 | 1.94 | 2.89 | 4.82 | 37.43 | 37.35 | 37.35 | 5.01 | 36.77 | 4.95 | 18.94 | 36.86 | 5.13 | 8.35 | 5.05 |
| 2aq9 | 31.32 | 31.15 | 31.20 | 31.23 | 31.02 | 30.43 | 31.40 | 31.49 | 31.07 | 30.53 | 28.17 | 31.51 | 30.38 | 30.19 | 31.78 | 30.80 | 31.16 | 31.15 | 31.82 | 31.82 |
| 2b9h | 1.68 | 1.74 | 1.54 | 1.35 | 44.63 | 40.12 | 37.56 | 44.58 | 49.95 | 33.92 | 52.00 | 43.24 | 52.22 | 49.77 | 19.41 | 19.25 | 19.18 | 25.07 | 18.89 | 19.07 |
| 2bba | 1.86 | 29.13 | 2.00 | 35.57 | 28.66 | 29.02 | 28.81 | 29.28 | 33.30 | 35.26 | 30.02 | 37.35 | 30.25 | 24.14 | 22.04 | 37.29 | 27.70 | 30.29 | 30.09 | 31.45 |
| 2cch | 1.26 | 25.33 | 1.58 | 25.28 | 6.75 | 19.26 | 32.00 | 32.19 | 39.49 | 6.83 | 41.79 | 19.22 | 44.58 | 37.04 | 44.31 | 41.65 | 36.15 | 2.28 | 36.14 | 33.33 |
| 2ce8 | 8.32 | 8.37 | 8.29 | 38.54 | 9.80 | 8.63 | 38.35 | 38.15 | 8.88 | 8.72 | 39.21 | 33.82 | 29.35 | 29.49 | 39.28 | 32.87 | 41.26 | 8.98 | 9.12 | 41.32 |
| 2d0n | 1.56 | 1.62 | 1.44 | 1.50 | 1.75 | 1.56 | 2.65 | 4.46 | 3.03 | 4.67 | 3.75 | 4.20 | 3.84 | 4.19 | 3.37 | 4.48 | 2.01 | 3.13 | 3.33 | 4.95 |
| 2drk | 17.59 | 17.63 | 1.75 | 17.57 | 3.55 | 17.59 | 1.59 | 17.62 | 1.30 | 17.58 | 17.55 | 17.59 | 1.42 | 1.32 | 17.59 | 1.49 | 17.62 | 2.46 | 17.54 | 17.79 |
| 2dyp | 24.63 | 58.23 | 16.71 | 58.35 | 58.04 | 58.15 | 24.95 | 58.23 | 58.30 | 24.90 | 15.44 | 16.14 | 21.01 | 21.33 | 23.74 | 5.08 | 41.15 | 47.69 | 33.74 | 58.73 |
| 2fff | 41.18 | 1.37 | 27.48 | 28.13 | 21.76 | 11.99 | 21.85 | 28.13 | 21.13 | 21.34 | 27.69 | 27.54 | 19.33 | 26.10 | 28.03 | 40.02 | 43.25 | 42.83 | 22.29 | 21.62 |
| 2ffu | 25.93 | 33.18 | 51.44 | 36.96 | 29.57 | 52.01 | 51.52 | 37.01 | 37.28 | 32.86 | 32.98 | 46.84 | 46.76 | 29.32 | 44.57 | 33.63 | 48.16 | 33.41 | 31.70 | 52.55 |
| 2fka | 4.32 | 4.28 | 4.32 | 3.35 | 3.67 | 3.35 | 4.61 | 7.01 | 4.69 | 9.30 | 8.47 | 8.52 | 8.50 | 8.53 | 6.15 | 5.95 | 3.82 | 8.36 | 6.23 | 8.50 |
| 2fmf | 11.65 | 6.48 | 6.62 | 6.86 | 6.65 | 6.49 | 6.68 | 6.46 | 6.60 | 6.64 | 6.48 | 6.36 | 6.38 | 6.37 | 11.24 | 11.29 | 6.17 | 8.57 | 6.13 | 8.79 |
| 2fts | 0.81 | 0.80 | 0.85 | 5.09 | 4.92 | 63.88 | 60.77 | 18.55 | 18.13 | 37.40 | 18.78 | 45.64 | 18.89 | 18.67 | 61.41 | 53.93 | 47.47 | 53.80 | 19.07 | 16.99 |
| 2fvj | 32.40 | 32.45 | 33.80 | 24.98 | 33.85 | 32.79 | 33.72 | 32.73 | 26.73 | 33.28 | 36.68 | 36.76 | 1.42 | 36.83 | 36.84 | 29.94 | 28.31 | 43.59 | 21.77 | 28.21 |
| 2ho2 | 13.03 | 12.90 | 12.91 | 6.08 | 8.76 | 12.74 | 14.19 | 5.80 | 3.25 | 6.57 | 5.66 | 3.89 | 12.26 | 12.28 | 12.22 | 6.02 | 3.25 | 1.69 | 12.17 | 9.30 |
| 2ht9 | 22.90 | 22.79 | 22.70 | 20.71 | 22.95 | 22.76 | 20.56 | 20.48 | 21.79 | 21.89 | 22.03 | 21.87 | 19.25 | 21.70 | 32.06 | 21.25 | 21.42 | 25.14 | 26.81 | 26.77 |
| 2o02 | 6.28 | 33.20 | 33.04 | 34.29 | 21.39 | 21.30 | 32.54 | 32.56 | 25.72 | 32.57 | 32.60 | 32.64 | 32.72 | 32.94 | 31.87 | 33.63 | 33.36 | 33.49 | 32.14 | 33.79 |
| 2o4j | 1.96 | 2.44 | 2.22 | 34.99 | 35.01 | 34.90 | 42.00 | 41.97 | 42.26 | 6.94 | 35.24 | 42.21 | 6.53 | 43.10 | 9.41 | 42.91 | 43.03 | 7.07 | 30.76 | 7.07 |
| 2o9v | 2.40 | 2.52 | 2.62 | 2.66 | 1.91 | 1.81 | 2.08 | 1.27 | 16.79 | 1.06 | 16.76 | 16.75 | 16.98 | 17.00 | 16.95 | 1.03 | 16.95 | 17.00 | 17.16 | 1.23 |
| 2oei | 14.73 | 14.78 | 14.69 | 14.68 | 14.78 | 15.07 | 15.26 | 15.00 | 1.46 | 1.52 | 0.99 | 14.17 | 1.67 | 1.39 | 10.03 | 10.06 | 16.02 | 15.91 | 15.76 | 15.81 |
| 2p0w | 18.26 | 31.58 | 24.87 | 21.24 | 17.33 | 26.63 | 25.77 | 25.42 | 24.66 | 36.11 | 20.58 | 26.20 | 25.34 | 1.41 | 14.17 | 22.70 | 28.30 | 30.24 | 26.37 | 13.72 |
| 2p1o | 34.31 | 32.99 | 12.42 | 33.58 | 32.51 | 53.12 | 37.91 | 37.74 | 22.70 | 22.53 | 22.55 | 38.71 | 2.74 | 29.57 | 1.62 | 10.67 | 35.35 | 35.79 | 34.42 | 38.95 |
| 2p1t | 33.09 | 33.56 | 33.19 | 36.07 | 34.33 | 35.46 | 36.07 | 36.14 | 35.43 | 31.84 | 36.01 | 31.65 | 32.08 | 35.46 | 36.14 | 34.25 | 4.25 | 37.64 | 35.53 | 33.71 |
| 2p54 | 26.36 | 26.41 | 26.90 | 23.97 | 25.94 | 44.53 | 27.17 | 27.48 | 25.69 | 25.84 | 26.77 | 26.63 | 31.05 | 26.89 | 26.86 | 32.29 | 27.90 | 31.98 | 42.94 | 27.79 |
| 2peh | 2.50 | 2.15 | 27.48 | 27.42 | 28.43 | 27.07 | 7.94 | 26.50 | 8.26 | 11.18 | 7.77 | 9.75 | 11.19 | 11.14 | 11.51 | 9.27 | 9.23 | 11.25 | 26.68 | 8.97 |
| 2pux | 1.46 | 1.57 | 1.94 | 3.81 | 1.91 | 3.72 | 2.22 | 4.26 | 7.91 | 43.77 | 38.42 | 8.87 | 38.35 | 37.80 | 35.45 | 40.87 | 10.29 | 40.42 | 10.85 | 40.96 |
| 2puy | 1.20 | 24.17 | 23.97 | 34.27 | 5.76 | 6.16 | 34.40 | 5.53 | 6.64 | 34.29 | 5.08 | 25.95 | 25.12 | 23.80 | 24.02 | 6.42 | 26.25 | 6.92 | 8.62 | 23.65 |
| 2qbx | 27.31 | 11.16 | 11.14 | 25.13 | 27.25 | 41.11 | 25.09 | 41.11 | 41.39 | 27.87 | 27.44 | 41.21 | 32.22 | 41.28 | 27.18 | 21.67 | 22.57 | 25.59 | 10.57 | 25.08 |
| 2qos | 22.14 | 22.11 | 2.26 | 24.49 | 22.17 | 28.68 | 22.68 | 23.49 | 26.62 | 28.71 | 16.89 | 28.77 | 25.09 | 25.73 | 17.04 | 13.58 | 26.10 | 26.58 | 25.02 | 22.72 |
| 2qse | 1.54 | 1.67 | 1.74 | 1.39 | 1.81 | 1.65 | 1.80 | 32.01 | 5.19 | 5.20 | 5.33 | 39.97 | 5.41 | 39.97 | 5.44 | 3.36 | 39.43 | 33.39 | 5.49 | 39.95 |
| 2r7g | 36.81 | 20.31 | 20.15 | 36.95 | 36.83 | 37.17 | 36.64 | 37.43 | 19.69 | 21.29 | 20.57 | 43.86 | 20.84 | 21.12 | 36.44 | 19.71 | 48.70 | 37.99 | 37.73 | 45.53 |
| 2r9q | 9.08 | 1.86 | 1.65 | 6.09 | 6.15 | 6.10 | 10.14 | 6.55 | 5.99 | 6.28 | 7.74 | 8.18 | 5.84 | 7.34 | 33.78 | 8.30 | 10.44 | 7.05 | 5.69 | 5.80 |
| 2v8y | 0.77 | 0.88 | 1.18 | 18.93 | 18.91 | 34.70 | 18.68 | 19.59 | 36.49 | 2.66 | 14.99 | 38.96 | 38.25 | 38.92 | 36.62 | 35.53 | 28.25 | 36.53 | 36.51 | 38.22 |
| 2vkn | 0.85 | 1.08 | 0.72 | 1.36 | 1.59 | 0.65 | 2.49 | 17.11 | 15.55 | 1.55 | 6.28 | 15.56 | 6.19 | 2.50 | 10.25 | 5.41 | 15.53 | 15.46 | 6.32 | 19.35 |
| 2vr3 | 23.80 | 25.53 | 38.24 | 13.78 | 33.64 | 26.71 | 16.24 | 37.25 | 13.53 | 32.94 | 28.85 | 20.48 | 20.47 | 24.33 | 37.45 | 35.11 | 20.76 | 36.52 | 37.77 | 36.70 |
| 2vwf | 22.18 | 22.17 | 0.63 | 0.62 | 6.84 | 0.66 | 7.30 | 1.58 | 30.83 | 26.12 | 26.08 | 26.12 | 30.73 | 3.28 | 5.44 | 15.09 | 12.13 | 12.32 | 11.86 | 3.70 |
| 2w2u | 26.38 | 24.01 | 23.98 | 24.28 | 24.08 | 24.13 | 25.02 | 25.03 | 25.10 | 25.37 | 25.26 | 23.87 | 11.19 | 21.72 | 21.82 | 23.86 | 25.70 | 24.32 | 23.86 | 1.27 |
| 2whx | 87.31 | 28.04 | 24.57 | 28.07 | 24.59 | 34.33 | 22.84 | 15.49 | 77.40 | 28.78 | 15.39 | 15.48 | 15.56 | 15.38 | 15.57 | 78.82 | 78.52 | 29.53 | 43.50 | 26.89 |
| 2xrw | 2.00 | 24.52 | 6.08 | 31.75 | 46.36 | 23.57 | 25.17 | 23.77 | 25.02 | 28.71 | 24.96 | 25.01 | 16.27 | 20.61 | 26.87 | 37.21 | 25.31 | 26.08 | 33.05 | 22.50 |
| 2xu7 | 1.12 | 0.90 | 1.04 | 4.46 | 0.92 | 1.17 | 3.01 | 2.57 | 14.19 | 4.24 | 3.95 | 3.51 | 2.03 | 4.67 | 5.61 | 44.13 | 43.53 | 42.32 | 43.69 | 41.21 |
| 2xvc | 25.82 | 22.93 | 22.72 | 26.58 | 27.84 | 26.74 | 26.79 | 26.71 | 33.26 | 33.22 | 33.26 | 33.04 | 26.59 | 28.02 | 23.54 | 27.92 | 32.84 | 25.95 | 28.08 | 26.89 |
| 2zjd | 0.84 | 1.33 | 1.71 | 30.78 | 30.78 | 30.21 | 30.22 | 28.91 | 8.15 | 29.36 | 7.70 | 18.35 | 7.99 | 18.63 | 33.12 | 28.77 | 8.23 | 19.30 | 19.19 | 8.22 |
| 3asl | 25.76 | 24.84 | 25.22 | 26.62 | 24.69 | 24.73 | 25.34 | 24.38 | 24.45 | 25.08 | 24.60 | 25.62 | 26.51 | 24.01 | 23.93 | 34.23 | 34.21 | 27.33 | 23.68 | 26.72 |
| 3awr | 20.70 | 11.44 | 12.47 | 12.49 | 22.97 | 23.14 | 23.50 | 22.93 | 22.89 | 19.32 | 23.00 | 24.17 | 11.09 | 19.64 | 19.53 | 24.28 | 23.14 | 23.86 | 19.93 | 23.07 |
| 3ayu | 0.73 | 1.08 | 17.69 | 4.76 | 24.25 | 15.81 | 17.26 | 25.07 | 16.07 | 8.56 | 2.45 | 20.68 | 18.86 | 31.88 | 32.03 | 31.76 | 31.74 | 31.52 | 31.76 | 31.69 |
| 3bfq | 0.36 | 23.94 | 33.46 | 33.43 | 33.84 | 33.82 | 33.97 | 29.51 | 33.13 | 33.26 | 33.05 | 11.75 | 13.46 | 31.52 | 33.94 | 31.52 | 23.70 | 32.63 | 12.85 | 23.85 |
| 3c3r | 12.09 | 2.55 | 12.06 | 12.06 | 39.50 | 2.38 | 39.70 | 5.90 | 5.93 | 36.74 | 6.04 | 5.92 | 5.71 | 40.60 | 5.71 | 40.70 | 5.57 | 40.57 | 5.61 | 40.53 |
| 3d32 | 0.64 | 18.52 | 19.52 | 19.50 | 19.33 | 11.28 | 11.19 | 15.67 | 18.16 | 22.02 | 15.77 | 11.24 | 8.33 | 18.00 | 24.95 | 15.64 | 15.49 | 14.68 | 11.88 | 16.48 |
| 3ds4 | 23.74 | 23.76 | 4.95 | 24.17 | 4.88 | 22.95 | 23.80 | 23.60 | 9.34 | 23.83 | 9.31 | 24.35 | 22.90 | 10.41 | 22.78 | 22.88 | 22.81 | 23.35 | 23.37 | 22.95 |
| 3ery | 1.00 | 2.55 | 5.93 | 5.45 | 7.78 | 7.75 | 8.77 | 7.28 | 9.97 | 8.07 | 6.61 | 15.39 | 16.45 | 4.34 | 12.71 | 10.60 | 12.20 | 5.40 | 10.48 | 12.52 |
| 3fdo | 1.78 | 2.14 | 17.14 | 2.48 | 11.86 | 11.55 | 25.97 | 25.87 | 17.76 | 25.91 | 25.79 | 25.99 | 17.99 | 19.43 | 8.19 | 24.57 | 15.97 | 11.64 | 17.96 | 11.57 |
| 3g2s | 13.44 | 1.95 | 1.76 | 23.52 | 23.58 | 23.67 | 2.00 | 27.69 | 24.45 | 19.59 | 8.71 | 24.47 | 24.39 | 24.34 | 28.91 | 8.55 | 8.18 | 17.67 | 8.12 | 24.63 |
| 3gyt | 1.96 | 38.36 | 38.40 | 37.95 | 38.62 | 37.71 | 41.38 | 41.15 | 43.38 | 38.26 | 41.02 | 41.18 | 39.52 | 45.94 | 41.29 | 39.50 | 41.33 | 39.46 | 41.52 | 39.42 |
| 3h1z | 1.47 | 10.16 | 9.55 | 21.21 | 21.21 | 31.30 | 26.66 | 21.15 | 21.23 | 25.37 | 24.18 | 26.44 | 19.60 | 4.58 | 30.15 | 37.81 | 57.82 | 37.76 | 31.40 | 31.10 |
| 3i5r | 1.23 | 1.11 | 1.33 | 1.07 | 1.46 | 15.58 | 15.49 | 15.56 | 15.62 | 15.53 | 10.46 | 10.24 | 10.13 | 15.46 | 10.28 | 15.49 | 11.55 | 16.66 | 11.21 | 6.33 |
| 3ivv | 15.75 | 11.78 | 30.92 | 30.90 | 32.45 | 32.37 | 32.36 | 27.21 | 24.69 | 17.71 | 26.03 | 18.88 | 18.54 | 32.37 | 17.36 | 16.43 | 31.89 | 28.15 | 26.87 | 26.88 |
| 3kmr | 32.80 | 34.79 | 34.91 | 34.99 | 2.04 | 35.49 | 1.13 | 35.41 | 35.88 | 35.53 | 35.34 | 34.47 | 35.45 | 34.37 | 35.57 | 34.75 | 34.01 | 33.92 | 33.90 | 33.93 |
| 3kuj | 1.03 | 19.46 | 20.99 | 19.69 | 15.11 | 17.80 | 18.73 | 17.86 | 21.80 | 15.28 | 20.96 | 20.85 | 25.27 | 21.85 | 21.10 | 21.16 | 24.37 | 21.95 | 25.85 | 21.91 |
| 3kus | 18.01 | 15.95 | 18.42 | 24.28 | 24.85 | 25.09 | 16.64 | 18.19 | 26.66 | 26.23 | 27.05 | 26.57 | 26.46 | 25.31 | 23.37 | 23.46 | 23.36 | 27.18 | 26.79 | 17.81 |
| 3l0e | 28.76 | 28.67 | 29.30 | 28.12 | 32.11 | 28.38 | 28.38 | 29.41 | 39.25 | 32.57 | 39.18 | 28.91 | 26.63 | 32.00 | 25.86 | 26.44 | 26.43 | 29.32 | 26.77 | 1.27 |
| 3ll8 | 41.91 | 0.84 | 3.38 | 1.66 | 24.87 | 24.84 | 24.87 | 24.99 | 27.94 | 12.81 | 25.03 | 12.36 | 22.09 | 10.27 | 22.21 | 20.19 | 20.42 | 48.65 | 22.26 | 22.35 |
| 3llz | 31.37 | 27.10 | 31.48 | 1.41 | 1.35 | 31.55 | 23.43 | 23.62 | 23.71 | 21.97 | 16.60 | 23.29 | 16.45 | 23.40 | 27.51 | 21.90 | 21.88 | 27.52 | 27.44 | 23.67 |
| 3obq | 17.80 | 17.87 | 17.65 | 25.58 | 23.55 | 7.64 | 26.30 | 7.60 | 7.63 | 7.80 | 26.77 | 32.77 | 7.47 | 32.84 | 26.63 | 27.44 | 26.00 | 32.84 | 42.51 | 28.15 |
| 3olf | 1.46 | 1.64 | 1.44 | 1.30 | 1.17 | 1.93 | 37.06 | 36.97 | 37.55 | 37.98 | 26.50 | 26.62 | 37.85 | 37.90 | 26.35 | 36.83 | 38.02 | 37.68 | 32.26 | 39.52 |
| 3p72 | 5.55 | 35.30 | 35.31 | 21.45 | 35.31 | 35.45 | 35.19 | 35.43 | 41.33 | 6.93 | 41.33 | 41.28 | 35.17 | 41.18 | 21.82 | 39.52 | 23.85 | 23.72 | 23.45 | 39.55 |
| 3p8f | 1.13 | 5.63 | 14.44 | 14.29 | 26.59 | 16.54 | 26.59 | 26.59 | 15.75 | 16.48 | 16.39 | 22.64 | 22.51 | 22.12 | 15.57 | 15.62 | 6.15 | 12.45 | 22.46 | 12.14 |
| 3ptl | 33.75 | 17.15 | 28.19 | 20.19 | 19.99 | 29.52 | 29.58 | 28.55 | 13.77 | 13.81 | 19.03 | 19.05 | 13.76 | 18.70 | 29.41 | 16.68 | 19.95 | 27.01 | 16.29 | 16.34 |
| 3qis | 0.90 | 0.81 | 0.77 | 0.69 | 13.58 | 30.68 | 30.57 | 41.47 | 34.57 | 41.44 | 2.59 | 34.13 | 2.02 | 12.81 | 31.01 | 2.77 | 34.61 | 31.70 | 31.47 | 28.71 |
| 3rm1 | 1.42 | 1.63 | 20.03 | 27.33 | 20.10 | 0.70 | 27.21 | 27.38 | 27.32 | 27.36 | 18.46 | 20.15 | 27.41 | 1.00 | 2.75 | 8.79 | 2.99 | 27.38 | 7.55 | 9.43 |
| 3rqg | 40.98 | 41.05 | 41.13 | 40.80 | 41.33 | 41.29 | 41.31 | 40.56 | 37.49 | 51.55 | 31.49 | 42.02 | 37.01 | 42.14 | 2.72 | 43.00 | 42.42 | 42.59 | 35.38 | 3.36 |
| 3sfj | 0.57 | 0.47 | 19.22 | 24.76 | 21.60 | 19.48 | 25.90 | 25.89 | 25.97 | 26.38 | 26.61 | 17.97 | 19.48 | 18.37 | 26.70 | 17.47 | 26.05 | 16.94 | 23.79 | 25.78 |
| 3so6 | 0.89 | 4.72 | 4.88 | 7.37 | 4.89 | 27.03 | 26.86 | 26.88 | 26.91 | 21.30 | 17.82 | 19.11 | 6.03 | 14.28 | 8.25 | 29.11 | 24.14 | 6.30 | 23.12 | 24.04 |
| 3tjv | 8.40 | 9.31 | 9.51 | 11.97 | 11.89 | 13.44 | 13.42 | 10.49 | 32.10 | 29.92 | 17.43 | 16.78 | 12.84 | 11.06 | 31.59 | 29.67 | 29.64 | 10.53 | 11.95 | 29.70 |
| 3tzy | 35.87 | 35.75 | 35.80 | 39.95 | 40.11 | 39.70 | 39.55 | 40.03 | 39.87 | 40.20 | 37.67 | 35.03 | 40.20 | 39.37 | 35.01 | 31.95 | 31.83 | 40.09 | 20.79 | 35.99 |
| 3u9q | 2.24 | 30.83 | 31.44 | 2.08 | 30.53 | 31.63 | 31.08 | 1.80 | 1.41 | 31.98 | 31.79 | 31.94 | 32.20 | 30.51 | 41.16 | 30.75 | 30.65 | 30.56 | 29.34 | 30.69 |
| 3up3 | 32.22 | 32.15 | 32.35 | 1.16 | 1.21 | 31.26 | 46.11 | 24.68 | 46.12 | 31.15 | 25.90 | 46.17 | 30.10 | 5.41 | 5.62 | 5.73 | 46.03 | 28.45 | 5.61 | 32.29 |
| 3v2x | 7.00 | 6.86 | 1.29 | 1.32 | 1.53 | 6.30 | 6.49 | 6.80 | 18.02 | 18.09 | 18.05 | 24.19 | 19.86 | 5.45 | 20.67 | 19.84 | 5.90 | 17.68 | 4.54 | 19.88 |
| 3vtc | 1.30 | 1.00 | 0.98 | 31.60 | 32.84 | 41.47 | 31.43 | 41.39 | 5.76 | 33.01 | 31.51 | 31.20 | 40.22 | 10.86 | 10.91 | 32.73 | 32.94 | 40.65 | 9.00 | 31.02 |
| 3w1b | 83.31 | 83.19 | 83.42 | 83.67 | 83.54 | 23.29 | 42.35 | 86.95 | 42.62 | 21.52 | 52.63 | 22.74 | 23.10 | 27.21 | 39.57 | 52.99 | 27.28 | 83.93 | 27.03 | 46.41 |
| 3zqh | 25.98 | 29.46 | 20.48 | 28.10 | 27.63 | 26.73 | 38.95 | 38.89 | 38.55 | 38.40 | 37.20 | 33.17 | 16.09 | 18.70 | 27.70 | 28.14 | 33.02 | 15.05 | 13.22 | 14.99 |
| 4b4n | 29.46 | 28.63 | 30.86 | 26.29 | 31.15 | 27.51 | 22.32 | 26.47 | 28.03 | 22.23 | 29.94 | 27.86 | 27.79 | 27.89 | 29.81 | 25.56 | 26.00 | 25.76 | 28.73 | 25.30 |
| 4dcb | 14.72 | 44.10 | 45.29 | 43.65 | 43.89 | 45.48 | 51.57 | 51.56 | 11.96 | 11.99 | 12.30 | 52.11 | 9.55 | 51.65 | 9.51 | 47.39 | 52.34 | 51.32 | 53.29 | 14.41 |
| 4e34 | 1.33 | 14.72 | 21.05 | 14.59 | 28.30 | 14.50 | 14.59 | 21.42 | 21.09 | 21.02 | 14.45 | 28.22 | 28.33 | 21.06 | 10.05 | 22.28 | 22.12 | 9.15 | 22.34 | 9.79 |
| 4eik | 0.67 | 0.66 | 0.95 | 1.13 | 0.71 | 23.00 | 23.05 | 24.97 | 24.93 | 24.93 | 1.83 | 11.89 | 11.78 | 10.52 | 26.23 | 26.35 | 26.43 | 27.02 | 26.95 | 26.97 |
| 4ery | 1.44 | 1.60 | 12.71 | 12.22 | 12.58 | 12.60 | 11.94 | 13.03 | 13.12 | 12.09 | 11.60 | 11.88 | 11.37 | 14.49 | 12.06 | 7.70 | 11.94 | 7.61 | 15.33 | 14.53 |
| 4f14 | 1.88 | 1.85 | 1.81 | 2.27 | 19.22 | 8.40 | 10.84 | 19.13 | 7.75 | 19.00 | 19.05 | 11.43 | 18.76 | 7.71 | 11.09 | 18.37 | 7.83 | 10.68 | 11.61 | 21.41 |
| 4f1z | 30.87 | 28.76 | 15.26 | 30.24 | 24.10 | 24.08 | 32.11 | 14.92 | 33.80 | 31.09 | 32.13 | 34.94 | 32.37 | 24.16 | 24.03 | 25.39 | 49.90 | 31.55 | 28.81 | 49.75 |
| 4gq6 | 9.27 | 10.04 | 10.08 | 9.58 | 33.17 | 33.26 | 32.71 | 33.31 | 9.77 | 10.30 | 32.90 | 34.72 | 10.35 | 12.03 | 39.61 | 13.42 | 31.90 | 30.95 | 25.81 | 34.70 |
| 4gxl | 11.10 | 9.98 | 10.04 | 11.31 | 8.88 | 8.77 | 9.31 | 8.91 | 23.74 | 29.58 | 10.20 | 10.31 | 27.75 | 12.58 | 12.11 | 32.64 | 27.66 | 10.72 | 12.07 | 14.49 |
| 4gyw | 15.33 | 15.91 | 39.49 | 25.76 | 26.04 | 45.45 | 38.96 | 27.91 | 47.85 | 38.15 | 38.07 | 42.04 | 47.71 | 40.62 | 41.03 | 24.90 | 49.55 | 38.70 | 25.67 | 38.59 |
| 4h4f | 1.52 | 14.44 | 1.04 | 1.31 | 14.52 | 14.83 | 15.77 | 2.14 | 14.90 | 34.41 | 15.15 | 14.91 | 15.09 | 34.46 | 9.82 | 27.83 | 36.41 | 33.77 | 9.50 | 10.21 |
| 4hom | 32.20 | 36.79 | 32.43 | 33.15 | 37.00 | 24.45 | 33.33 | 36.16 | 40.93 | 33.73 | 40.65 | 34.37 | 38.17 | 37.30 | 31.25 | 35.53 | 37.45 | 37.11 | 36.05 | 36.86 |
| 4htp | 38.72 | 38.36 | 38.83 | 44.65 | 34.99 | 35.12 | 38.92 | 33.29 | 33.56 | 34.86 | 38.87 | 34.43 | 38.83 | 26.83 | 26.49 | 27.86 | 46.31 | 30.36 | 25.34 | 34.85 |
| 4iim | 5.13 | 5.10 | 3.29 | 16.94 | 8.01 | 8.13 | 8.34 | 16.94 | 22.07 | 17.16 | 22.21 | 10.99 | 24.91 | 22.37 | 22.32 | 10.65 | 22.34 | 10.83 | 11.03 | 0.81 |
| 4j8s | 29.49 | 26.53 | 26.62 | 26.54 | 34.93 | 30.01 | 28.14 | 26.78 | 44.04 | 44.02 | 30.72 | 29.97 | 26.79 | 26.66 | 31.36 | 26.92 | 44.04 | 26.74 | 26.85 | 31.29 |
| 4k0u | 1.61 | 1.77 | 1.49 | 1.50 | 1.88 | 11.45 | 6.45 | 3.39 | 2.32 | 11.31 | 10.88 | 2.57 | 10.97 | 11.11 | 11.01 | 11.14 | 9.02 | 8.76 | 13.62 | 2.79 |

**S5(b). I-RMSD values of all 20 poses obtained after blind docking by HEX on 133 protein-peptide complexes.**

| **ID** | **Pose1** | **Pose2** | **Pose3** | **Pose4** | **Pose5** | **Pose6** | **Pose7** | **Pose8** | **Pose9** | **Pose10** | **Pose11** | **Pose12** | **Pose13** | **Pose14** | **Pose15** | **Pose16** | **Pose17** | **Pose18** | **Pose19** | **Pose20** |
| --- | --- | --- | --- | --- | --- | --- | --- | --- | --- | --- | --- | --- | --- | --- | --- | --- | --- | --- | --- | --- |
| 1cjr | 13.24 | 22.37 | 6.25 | 9.15 | 1.87 | 7.25 | 12.20 | 10.66 | 12.60 | 12.16 | 10.77 | 4.09 | 11.45 | 8.76 | 22.50 | 4.09 | 19.60 | 10.18 | 10.65 | 17.49 |
| 1cka | 182.04 | NA | NA | NA | NA | NA | NA | NA | NA | NA | NA | NA | NA | NA | NA | NA | NA | NA | NA | NA |
| 1cvu | 56.68 | 44.12 | 47.71 | 46.94 | 56.68 | 46.52 | 55.96 | 45.63 | 54.76 | 55.55 | 46.70 | 48.57 | 54.68 | 45.56 | 49.31 | 48.32 | 43.28 | 45.68 | 59.70 | 55.67 |
| 1d4t | 17.51 | 3.66 | 18.15 | 21.07 | 15.25 | 27.17 | 25.26 | 15.97 | 10.04 | 24.80 | 19.75 | 26.33 | 20.01 | 10.05 | 19.69 | 7.05 | 26.43 | 8.73 | 18.00 | 16.79 |
| 1eg4 | 34.72 | 64.62 | 64.71 | 62.89 | 65.90 | 41.86 | 35.49 | 54.63 | 32.03 | 63.61 | 28.81 | 33.40 | 40.31 | 37.06 | 24.79 | 42.36 | 34.49 | 53.54 | 61.56 | 63.57 |
| 1h6w | 143.01 | 142.70 | 142.73 | 142.80 | 141.95 | 143.34 | 141.57 | 142.98 | 142.78 | 142.78 | 142.89 | 142.14 | 142.17 | 142.73 | 142.91 | 141.91 | 143.03 | 142.54 | 142.71 | 141.84 |
| 1hc9 | 45.32 | 42.28 | 28.79 | 49.01 | 41.81 | 45.25 | 45.76 | 48.50 | 48.49 | 29.06 | 25.36 | 40.03 | 44.47 | 26.45 | 27.54 | 40.72 | 43.36 | 37.27 | 41.84 | 48.57 |
| 1jbu | 15.66 | 40.88 | 34.25 | 23.73 | 30.72 | 26.85 | 40.63 | 42.69 | 20.31 | 37.41 | 37.90 | 32.77 | 17.10 | 38.42 | 11.58 | 22.66 | 22.64 | 30.95 | 30.63 | 20.77 |
| 1k5n | 16.15 | 13.14 | 26.53 | 18.83 | 15.11 | 46.57 | 22.90 | 16.58 | 17.60 | 18.39 | 11.44 | 29.12 | 17.60 | 16.81 | 14.88 | 13.74 | 30.02 | 45.59 | 46.93 | 26.85 |
| 1mfg | 24.06 | 21.95 | 27.68 | 26.45 | 30.15 | 29.02 | 18.94 | 27.18 | 21.07 | 13.88 | 22.63 | 16.23 | 27.71 | 24.70 | 28.63 | 15.23 | 26.94 | 28.08 | 30.76 | 22.35 |
| 1nln | 51.50 | 51.11 | 51.12 | 54.34 | 54.96 | 55.95 | 52.66 | 51.71 | 53.24 | 56.55 | 53.85 | 55.54 | 49.86 | 48.45 | 54.86 | 54.34 | 48.34 | 52.59 | 57.95 | 51.36 |
| 1nq7 | 20.58 | 39.93 | 36.14 | 37.76 | 5.59 | 38.30 | 15.70 | 33.31 | 34.18 | 35.92 | 37.10 | 38.42 | 11.16 | 33.50 | 37.64 | 30.10 | 8.04 | 30.76 | 34.17 | 5.80 |
| 1ntv | 28.66 | 18.07 | 14.04 | 10.19 | 15.67 | 20.63 | 27.43 | 16.17 | 19.41 | 16.64 | 23.23 | 20.86 | 14.16 | 25.22 | 19.32 | 29.35 | 28.23 | 27.85 | 27.04 | 26.99 |
| 1nx1 | 73.84 | 74.07 | 72.98 | 74.99 | 72.35 | 74.20 | 72.77 | 75.99 | 73.11 | 73.93 | 74.05 | 71.64 | 71.72 | 74.61 | 73.44 | 74.16 | 75.33 | 50.11 | 77.00 | 78.25 |
| 1oai | 20.65 | 27.48 | 17.66 | 18.09 | 18.17 | 28.32 | 19.31 | 18.99 | 24.10 | 28.96 | 17.76 | 22.90 | 27.83 | 17.05 | 21.94 | 23.52 | 21.41 | 26.17 | 26.01 | 27.08 |
| 1oj5 | 30.39 | 21.82 | 25.34 | 28.22 | 30.83 | 31.83 | 30.74 | 28.49 | 34.05 | 33.72 | 29.09 | 27.89 | 33.11 | 28.42 | 32.02 | 33.70 | 27.03 | 34.28 | 31.58 | 32.37 |
| 1ou8 | 95.55 | 95.30 | 95.50 | 95.45 | 95.37 | 95.52 | 95.12 | 95.46 | 95.40 | 95.31 | 95.98 | 96.09 | 94.62 | 96.04 | 94.82 | 96.20 | 96.02 | 94.96 | 96.13 | 94.52 |
| 1ow6 | 53.38 | 54.27 | 35.97 | 44.93 | 48.45 | 37.26 | 43.18 | 51.10 | 41.08 | 42.80 | 53.02 | 43.61 | 37.68 | 49.84 | 49.97 | 49.91 | 36.34 | 36.49 | 34.21 | 51.98 |
| 1pzl | 90.21 | 90.30 | 89.41 | 90.51 | 89.47 | 89.34 | 89.85 | 88.78 | 89.31 | 93.25 | 89.48 | 89.48 | 89.42 | 90.67 | 89.04 | 89.89 | 89.43 | 89.62 | 93.63 | 90.40 |
| 1qkz | 32.74 | 34.96 | 34.23 | 33.92 | 34.38 | 36.97 | 34.49 | 32.93 | 33.77 | 34.99 | 33.42 | 36.80 | 33.33 | 33.27 | 35.16 | 29.27 | 37.11 | 37.56 | 34.08 | 36.70 |
| 1rst | 59.13 | 58.99 | 57.12 | 59.00 | 61.71 | 60.64 | 60.77 | 60.80 | 61.03 | 59.39 | 61.88 | 58.29 | 58.88 | 60.76 | 61.76 | 58.32 | 60.43 | 60.52 | 57.73 | 58.08 |
| 1rxz | 84.54 | 83.91 | 85.36 | 85.83 | 83.67 | 84.40 | 83.63 | 84.36 | 84.86 | 84.32 | 83.19 | 85.21 | 84.62 | 86.28 | 84.66 | 85.35 | 85.38 | 82.71 | 84.54 | 82.62 |
| 1sfi | 26.24 | 26.45 | 40.52 | 37.43 | 40.57 | 38.44 | 25.83 | 41.26 | 39.27 | 26.54 | 23.26 | 26.14 | 39.99 | 31.11 | 28.49 | 38.37 | 39.66 | 26.31 | 37.32 | 40.15 |
| 1ssh | 23.29 | 21.97 | 22.53 | 31.47 | 23.41 | 27.62 | 24.60 | 17.28 | 16.18 | 32.30 | 31.47 | 21.87 | 20.83 | 19.19 | 16.19 | 28.36 | 24.67 | 21.77 | 24.74 | 25.65 |
| 1t08 | 40.58 | 41.01 | 46.39 | 39.24 | 42.26 | 39.37 | 39.07 | 40.46 | 42.33 | 37.17 | 47.21 | 43.05 | 40.59 | 39.53 | 39.32 | 40.94 | 40.93 | 32.97 | 39.32 | 45.12 |
| 1t4f | 8.93 | 1.66 | 8.06 | 6.55 | 8.25 | 8.72 | 6.25 | 9.50 | 5.65 | 7.58 | 9.50 | 8.15 | 4.08 | 11.80 | 6.30 | 7.53 | 9.16 | 5.12 | 10.04 | 8.54 |
| 1t7r | 37.56 | 43.22 | 36.31 | 41.33 | 38.94 | 45.46 | 43.65 | 37.18 | 37.28 | 45.72 | 40.12 | 47.30 | 45.73 | 42.71 | 39.54 | 44.53 | 46.42 | 43.79 | 38.52 | 47.29 |
| 1tfc | 13.84 | 37.53 | 27.18 | 25.60 | 29.13 | 24.71 | 21.76 | 47.16 | 26.07 | 31.63 | 26.80 | 39.58 | 38.37 | 32.09 | 38.79 | 29.92 | 35.77 | 35.92 | 39.21 | 26.45 |
| 1u00 | 27.36 | 29.97 | 26.30 | 27.46 | 26.89 | 23.85 | 25.98 | 25.87 | 24.92 | 29.78 | 24.85 | 23.68 | 29.21 | 22.46 | 24.37 | 22.70 | 24.29 | 28.37 | 23.32 | 29.41 |
| 1uj0 | 29.48 | 28.08 | 32.78 | 19.21 | 30.92 | 31.81 | 32.95 | 30.83 | 29.31 | 30.73 | 25.12 | 28.09 | 25.09 | 26.78 | 19.50 | 24.65 | 18.61 | 30.05 | 16.26 | 28.41 |
| 1x2r | 63.37 | 61.21 | 66.38 | 67.55 | 64.71 | 63.44 | 65.63 | 55.17 | 62.93 | 63.82 | 64.85 | 60.43 | 60.33 | 65.01 | 65.86 | 65.29 | 63.62 | 63.55 | 61.82 | 63.34 |
| 1xoc | 41.38 | 45.40 | 44.47 | 43.10 | 43.07 | 42.48 | 43.95 | 43.28 | 42.43 | 40.32 | 43.52 | 41.20 | 41.46 | 41.79 | 45.56 | 41.96 | 42.06 | 44.81 | 44.47 | 43.08 |
| 1ymt | 84.37 | 84.75 | 83.95 | 78.94 | 80.73 | 90.92 | 79.95 | 80.50 | 84.20 | 78.78 | 78.24 | 79.48 | 71.28 | 79.87 | 77.00 | 84.33 | 78.50 | 78.18 | 80.53 | 83.85 |
| 1yuc | 77.23 | 72.82 | 53.44 | 75.93 | 52.42 | 53.17 | 76.35 | 56.80 | 55.01 | 55.46 | 56.00 | 50.11 | 50.87 | 59.91 | 54.09 | 76.82 | 56.58 | 54.56 | 75.83 | 50.70 |
| 1ywo | 17.00 | 17.68 | 23.04 | 22.38 | 26.05 | 25.79 | 18.53 | 23.34 | 24.71 | 22.67 | 21.40 | 20.53 | 19.32 | 24.92 | 28.57 | 24.19 | 27.13 | 23.66 | 23.28 | 30.60 |
| 2a25 | 33.90 | 33.65 | 32.91 | 34.96 | 29.97 | 31.00 | 29.86 | 33.09 | 33.72 | 30.90 | 34.53 | 29.73 | 34.86 | 30.28 | 29.20 | 31.70 | 29.88 | 30.78 | 32.58 | 30.78 |
| 2a3i | 64.87 | 66.76 | 67.37 | 65.92 | 64.51 | 67.41 | 63.10 | 66.52 | 63.11 | 65.14 | 70.21 | 68.43 | 70.21 | 76.71 | 66.39 | 67.37 | 61.69 | 64.67 | 62.95 | 68.69 |
| 2aq9 | 95.94 | 96.22 | 91.38 | 96.73 | 96.17 | 91.24 | 90.53 | 96.92 | 97.16 | 96.14 | 96.80 | 96.77 | 95.56 | 90.64 | 97.02 | 91.00 | 96.49 | 96.30 | 91.96 | 91.91 |
| 2b9h | 39.72 | 22.76 | 19.03 | 8.29 | 15.73 | 47.20 | 21.55 | 34.24 | 22.74 | 50.34 | 48.16 | 31.66 | 48.26 | 48.57 | 22.27 | 44.37 | 17.07 | 30.29 | 21.22 | 34.65 |
| 2bba | 66.59 | 67.01 | 67.46 | 64.04 | 65.84 | 67.57 | 69.16 | 66.05 | 66.75 | 66.69 | 46.40 | 65.18 | 33.06 | 49.50 | 65.21 | 64.66 | 48.24 | 67.74 | 42.24 | 48.47 |
| 2cch | 35.09 | 31.67 | 37.55 | 34.40 | 31.54 | 29.66 | 15.89 | 19.85 | 4.16 | 36.20 | 29.01 | 29.91 | 36.52 | 18.44 | 40.20 | 29.75 | 42.45 | 9.66 | 31.66 | 33.94 |
| 2ce8 | 49.07 | 33.93 | 46.75 | 47.00 | 46.44 | 34.62 | 35.56 | 40.62 | 36.67 | 36.47 | 41.84 | 38.91 | 42.63 | 44.78 | 41.20 | 37.70 | 41.28 | 40.24 | 38.33 | 40.84 |
| 2d0n | 23.35 | 22.77 | 21.26 | 20.88 | 17.33 | 18.07 | 26.24 | 15.90 | 22.48 | 30.15 | 21.50 | 19.55 | 28.86 | 21.97 | 31.72 | 17.13 | 17.96 | 19.82 | 29.17 | 21.28 |
| 2drk | 21.98 | 27.70 | 1.65 | 22.68 | 20.20 | 19.90 | 26.89 | 25.62 | 21.53 | 2.09 | 22.46 | 19.93 | 3.76 | 24.79 | 22.34 | 11.02 | 17.56 | 24.87 | 24.47 | 20.06 |
| 2dyp | 16.36 | 20.34 | 16.59 | 26.83 | 16.70 | 20.97 | 20.49 | 20.14 | 20.68 | 20.09 | 18.16 | 19.18 | 22.11 | 26.68 | 21.48 | 17.30 | 18.53 | 17.59 | 18.30 | 20.92 |
| 2fff | 29.23 | 28.52 | 53.72 | 40.34 | 53.07 | 34.99 | 45.53 | 29.79 | 45.06 | 28.41 | 40.97 | 40.94 | 43.87 | 28.90 | 31.38 | 26.39 | 32.73 | 44.23 | 23.90 | 30.86 |
| 2ffu | 15.66 | 22.02 | 21.57 | 15.48 | 15.56 | 19.67 | 15.42 | 15.66 | 14.60 | 16.11 | 12.52 | 14.86 | 14.20 | 21.01 | 14.74 | 19.07 | 14.33 | 14.14 | 22.40 | 14.14 |
| 2fka | 91.34 | 91.26 | 91.38 | 91.17 | 94.00 | 91.37 | 91.40 | 94.02 | 93.99 | 93.96 | 93.93 | 93.97 | 94.07 | 96.67 | 88.36 | 96.69 | 93.92 | 96.67 | 96.63 | 96.65 |
| 2fmf | 106.47 | 104.96 | 105.14 | 103.56 | 103.83 | 108.16 | 104.85 | 107.51 | 103.47 | 103.65 | 102.14 | 105.05 | 106.79 | 102.38 | 103.53 | 109.20 | 101.93 | 105.27 | 101.85 | 102.00 |
| 2fts | 47.79 | 34.16 | 37.11 | 27.38 | 45.67 | 46.28 | 28.54 | 46.52 | 13.36 | 33.11 | 45.18 | 45.51 | 23.56 | 45.09 | 44.14 | 46.95 | 39.75 | 44.15 | 16.95 | 31.74 |
| 2fvj | 8.72 | 30.76 | 40.89 | 41.24 | 35.81 | 32.87 | 30.65 | 17.76 | 33.22 | 42.13 | 9.10 | 38.60 | 4.54 | 40.32 | 42.24 | 16.51 | 32.24 | 42.83 | 42.32 | 36.17 |
| 2ho2 | 17.92 | 19.52 | 15.96 | 19.94 | 20.01 | 21.22 | 17.38 | 19.20 | 18.59 | 16.12 | 21.17 | 19.94 | 16.61 | 15.20 | 7.93 | 18.25 | 20.03 | 24.16 | 20.60 | 22.27 |
| 2ht9 | 48.10 | 48.64 | 40.76 | 47.30 | 47.50 | 48.75 | 49.26 | 47.55 | 42.97 | 49.97 | 47.18 | 50.31 | 37.67 | 46.93 | 47.88 | 46.71 | 48.97 | 48.04 | 46.74 | 42.88 |
| 2o02 | 1.04 | 14.33 | 14.82 | 14.71 | 2.72 | 29.37 | 14.08 | 15.58 | 11.63 | 5.90 | 7.47 | 23.60 | 7.54 | 2.92 | 14.44 | 7.59 | 16.37 | 8.81 | 14.88 | 14.22 |
| 2o4j | 47.82 | 48.40 | 54.37 | 40.61 | 37.78 | 52.47 | 41.92 | 38.25 | 52.17 | 41.37 | 53.22 | 44.10 | 50.76 | 50.39 | 29.85 | 43.55 | 53.07 | 59.40 | 38.57 | 36.37 |
| 2o9v | 28.68 | 28.06 | 29.06 | 26.00 | 28.09 | 21.19 | 15.11 | 23.88 | 25.78 | 29.91 | 19.29 | 22.69 | 24.41 | 27.93 | 20.08 | 26.28 | 25.75 | 26.91 | 22.53 | 26.37 |
| 2oei | 21.69 | 19.89 | 17.78 | 20.16 | 20.24 | 19.93 | 18.20 | 19.56 | 20.94 | 8.68 | 11.54 | 14.96 | 25.22 | 21.07 | 22.82 | 16.70 | 20.96 | 21.07 | 17.58 | 5.85 |
| 2p0w | 82.80 | 84.10 | 83.29 | 82.63 | 84.28 | 83.08 | 82.00 | 83.25 | 83.49 | 81.87 | 82.78 | 82.67 | 84.81 | 83.57 | 85.88 | 85.43 | 82.47 | 85.14 | 84.78 | 75.03 |
| 2p1o | 96.80 | 99.08 | 99.17 | 99.07 | 96.88 | 99.05 | 99.28 | 101.53 | 95.68 | 96.05 | 101.60 | 101.53 | 101.50 | 104.07 | 101.69 | 95.66 | 104.13 | 96.29 | 107.02 | 107.08 |
| 2p1t | 21.58 | 21.22 | 32.76 | 23.41 | 22.10 | 21.21 | 23.61 | 22.12 | 20.38 | 31.42 | 22.51 | 22.89 | 23.28 | 30.87 | 24.96 | 23.94 | 23.63 | 25.44 | 21.78 | 23.08 |
| 2p54 | 32.52 | 43.62 | 22.40 | 43.64 | 43.59 | 43.67 | 14.38 | 34.55 | 43.58 | 42.36 | 43.20 | 43.93 | 19.98 | 44.06 | 23.16 | 19.96 | 25.34 | 14.60 | 43.07 | 42.26 |
| 2peh | 4.78 | 30.21 | 22.85 | 22.48 | 25.78 | 30.90 | 25.61 | 23.25 | 24.05 | 29.71 | 24.53 | 22.79 | 20.77 | 21.92 | 23.81 | 35.25 | 19.74 | 22.02 | 23.97 | 19.50 |
| 2pux | 36.10 | 18.01 | 39.92 | 33.26 | 26.57 | 25.72 | 30.74 | 24.75 | 27.19 | 19.16 | 18.59 | 28.47 | 29.47 | 35.88 | 42.76 | 37.59 | 34.28 | 20.65 | 1.69 | 30.11 |
| 2puy | 69.97 | 70.77 | 68.87 | 70.70 | 68.98 | 67.57 | 67.32 | 67.37 | 68.71 | 70.27 | 68.99 | 68.32 | 69.01 | 70.29 | 67.72 | 68.25 | 68.38 | 70.06 | 70.13 | 69.51 |
| 2qbx | 1.32 | 6.63 | 31.91 | 4.25 | 16.59 | 16.72 | 24.79 | 30.53 | 13.21 | 30.77 | 24.17 | 6.77 | 25.58 | 36.95 | 16.54 | 28.10 | 2.52 | 25.36 | 25.45 | 25.97 |
| 2qos | 54.44 | 54.32 | 55.56 | 55.73 | 55.23 | 53.70 | 55.82 | 53.57 | 54.38 | 56.84 | 45.08 | 44.44 | 53.32 | 56.06 | 53.66 | 47.60 | 53.26 | 58.17 | 54.73 | 53.80 |
| 2qse | 30.94 | 29.83 | 32.09 | 32.37 | 30.50 | 29.07 | 29.11 | 26.98 | 37.37 | 33.64 | 37.86 | 29.57 | 31.20 | 26.83 | 20.42 | 29.64 | 29.13 | 25.23 | 33.85 | 36.63 |
| 2r7g | 16.87 | 16.12 | 15.57 | 18.74 | 16.85 | 21.75 | 11.89 | 16.40 | 15.64 | 11.02 | 19.83 | 32.42 | 21.90 | 18.60 | 16.81 | 21.80 | 20.29 | 18.86 | 28.32 | 32.13 |
| 2r9q | 77.20 | 81.25 | 82.27 | 78.71 | 81.65 | 77.05 | 81.72 | 78.59 | 77.40 | 75.43 | 75.97 | 54.61 | 82.13 | 75.81 | 56.14 | 81.53 | 56.31 | 79.14 | 76.91 | 80.11 |
| 2v8y | 27.24 | 14.87 | 26.60 | 26.56 | 37.74 | 38.46 | 23.63 | 30.27 | 36.70 | 28.38 | 40.34 | 19.55 | 37.52 | 39.70 | 40.33 | 27.33 | 31.81 | 38.17 | 24.46 | 17.43 |
| 2vkn | 30.05 | 27.06 | 28.27 | 24.47 | 27.21 | 24.93 | 25.81 | 11.84 | 15.36 | 20.71 | 27.19 | 25.01 | 23.41 | 24.11 | 25.67 | 25.26 | 32.45 | 34.26 | 20.49 | 18.29 |
| 2vr3 | 85.71 | 84.72 | 85.76 | 86.67 | 86.93 | 86.47 | 86.83 | 85.65 | 85.78 | 84.71 | 87.29 | 86.72 | 85.87 | 86.41 | 87.19 | 84.41 | 86.36 | 85.62 | 86.61 | 85.24 |
| 2vwf | 22.27 | 26.81 | 23.67 | 21.62 | 20.66 | 29.67 | 20.02 | 18.33 | 30.46 | 29.22 | 32.99 | 24.44 | 23.21 | 21.81 | 30.65 | 26.84 | 24.11 | 11.73 | 22.46 | 19.80 |
| 2w2u | 28.91 | 33.41 | 26.88 | 23.23 | 28.52 | 25.82 | 22.44 | 28.51 | 24.83 | 30.27 | 28.11 | 26.43 | 26.23 | 35.29 | 26.44 | 27.47 | 25.75 | 28.18 | 25.42 | 28.45 |
| 2whx | 42.78 | 23.14 | 27.87 | 28.13 | 30.54 | 37.66 | 30.50 | 38.87 | 55.34 | 32.79 | 34.78 | 31.82 | 55.70 | 42.29 | 34.84 | 40.38 | 32.44 | 30.25 | 22.81 | 25.36 |
| 2xrw | 27.85 | 32.48 | 37.31 | 31.39 | 33.08 | 30.38 | 33.85 | 35.80 | 24.37 | 35.39 | 39.50 | 37.18 | 37.57 | 39.02 | 25.46 | 27.24 | 25.25 | 25.71 | 39.68 | 21.90 |
| 2xu7 | 1.15 | 29.61 | 31.42 | 26.19 | 33.26 | 36.44 | 32.89 | 14.15 | 32.03 | 38.44 | 20.91 | 22.03 | 31.24 | 39.08 | 33.90 | 35.22 | 37.93 | 39.08 | 8.14 | 31.32 |
| 2xvc | 14.36 | 17.81 | 22.56 | 22.28 | 34.08 | 16.08 | 12.68 | 23.31 | 22.40 | 21.89 | 18.03 | 21.67 | 20.43 | 15.25 | 22.33 | 23.21 | 21.71 | 22.28 | 21.94 | 22.46 |
| 2zjd | 49.74 | 37.39 | 34.01 | 46.63 | 38.12 | 42.05 | 44.36 | 46.62 | 50.33 | 48.35 | 47.28 | 49.50 | 49.11 | 42.41 | 46.03 | 39.32 | 50.48 | 49.31 | 48.03 | 46.53 |
| 3asl | 19.04 | 21.03 | 23.09 | 21.26 | 24.97 | 12.54 | 23.08 | 25.88 | 24.59 | 19.75 | 18.53 | 21.63 | 8.23 | 10.93 | 21.04 | 15.29 | 10.34 | 20.91 | 18.22 | 21.00 |
| 3awr | 4.29 | 22.19 | 12.96 | 12.05 | 13.42 | 23.63 | 1.54 | 14.56 | 10.15 | 22.48 | 21.45 | 22.09 | 11.63 | 5.90 | 25.37 | 11.80 | 9.90 | 21.09 | 22.11 | 10.34 |
| 3ayu | 1.43 | 4.40 | 4.02 | 17.18 | 20.92 | 16.50 | 28.06 | 16.37 | 27.03 | 16.39 | 4.37 | 16.74 | 16.67 | 30.09 | 16.21 | 20.26 | 27.32 | 18.54 | 11.12 | 7.44 |
| 3bfq | 1.54 | 27.02 | 2.38 | 2.70 | 7.00 | 14.57 | 10.65 | 27.55 | 32.72 | 34.08 | 29.26 | 35.74 | 3.75 | 30.25 | 25.57 | 24.89 | 13.91 | 33.95 | 11.52 | 10.78 |
| 3c3r | 51.23 | 40.35 | 51.72 | 51.56 | 50.98 | 52.08 | 48.77 | 50.96 | 52.37 | 50.01 | 49.17 | 52.82 | 52.15 | 49.88 | 50.61 | 50.47 | 53.33 | 43.06 | 32.78 | 53.26 |
| 3d32 | 34.94 | 37.11 | 40.23 | 39.82 | 35.95 | 39.28 | 35.50 | 34.76 | 40.62 | 35.79 | 39.72 | 34.99 | 41.01 | 45.01 | 39.33 | 36.75 | 39.66 | 36.76 | 41.10 | 34.56 |
| 3ds4 | 1.61 | 5.71 | 11.97 | 4.88 | 9.92 | 9.20 | 11.25 | 7.94 | 10.49 | 9.19 | 10.72 | 11.20 | 7.47 | 8.84 | 10.91 | 11.99 | 6.62 | 10.83 | 2.69 | 10.53 |
| 3ery | 12.88 | 1.47 | 7.69 | 15.81 | 14.69 | 3.25 | 15.40 | 4.34 | 2.16 | 14.66 | 3.17 | 14.56 | 11.87 | 15.58 | 15.19 | 14.34 | 11.73 | 15.45 | 5.68 | 13.53 |
| 3fdo | 28.05 | 7.48 | 12.13 | 9.04 | 11.84 | 17.04 | 1.34 | 7.22 | 17.98 | 28.67 | 18.10 | 16.26 | 7.29 | 11.32 | 15.03 | 11.91 | 4.46 | 11.46 | 10.46 | 11.40 |
| 3g2s | 1.29 | 34.36 | 34.98 | 14.43 | 28.58 | 12.61 | 30.27 | 26.33 | 20.89 | 23.25 | 20.03 | 29.38 | 14.39 | 36.38 | 35.92 | 15.94 | 30.88 | 21.20 | 16.35 | 35.43 |
| 3gyt | 26.25 | 26.14 | 29.81 | 39.19 | 38.97 | 12.48 | 38.39 | 41.53 | 18.29 | 38.21 | 39.61 | 41.69 | 35.87 | 19.29 | 28.34 | 37.62 | 41.12 | 29.78 | 15.51 | 24.12 |
| 3h1z | 28.88 | 27.29 | 25.64 | 36.40 | 41.73 | 30.82 | 28.76 | 31.09 | 37.38 | 33.77 | 34.23 | 32.95 | 37.48 | 32.35 | 34.42 | 35.33 | 33.58 | 36.95 | 33.83 | 34.07 |
| 3i5r | 18.91 | 19.86 | 25.37 | 27.10 | 22.61 | 30.44 | 28.17 | 29.86 | 1.82 | 26.91 | 16.22 | 21.91 | 12.61 | 3.73 | 29.50 | 26.64 | 17.20 | 31.52 | 28.04 | 21.94 |
| 3ivv | 26.36 | 26.31 | 28.15 | 8.55 | 22.23 | 10.03 | 30.10 | 20.50 | 26.79 | 8.08 | 27.08 | 27.03 | 29.73 | 27.03 | 8.56 | 23.65 | 25.98 | 23.89 | 13.83 | 19.29 |
| 3kmr | 34.59 | 21.97 | 31.12 | 23.23 | 22.08 | 21.88 | 23.78 | 24.58 | 7.86 | 23.36 | 29.82 | 34.67 | 20.28 | 22.07 | 22.30 | 35.05 | 7.33 | 21.45 | 34.72 | 22.11 |
| 3kuj | 1.66 | 3.55 | 25.09 | 21.92 | 21.75 | 23.48 | 21.99 | 22.04 | 22.03 | 21.88 | 23.47 | 19.21 | 19.16 | 14.47 | 10.97 | 26.95 | 15.41 | 20.82 | 2.29 | 21.29 |
| 3kus | 25.83 | 24.20 | 15.90 | 22.41 | 23.53 | 16.24 | 11.49 | 13.40 | 11.28 | 10.81 | 15.27 | 17.04 | 19.65 | 22.58 | 22.07 | 7.03 | 19.04 | 8.64 | 11.88 | 25.08 |
| 3l0e | 23.65 | 34.43 | 34.13 | 33.66 | 26.18 | 21.32 | 20.71 | 21.64 | 28.05 | 26.97 | 29.98 | 35.30 | 38.09 | 27.94 | 30.96 | 33.79 | 22.84 | 40.97 | 23.49 | 34.40 |
| 3ll8 | 28.69 | 31.98 | 28.57 | 35.35 | 15.90 | 39.94 | 30.94 | 28.44 | 29.07 | 36.47 | 33.75 | 40.24 | 40.73 | 27.41 | 25.95 | 41.90 | 29.42 | 33.50 | 28.00 | 33.52 |
| 3llz | 1.67 | 21.59 | 28.80 | 29.99 | 29.29 | 18.44 | 25.29 | 31.29 | 27.02 | 30.04 | 27.88 | 3.23 | 31.52 | 31.43 | 20.44 | 5.18 | 26.47 | 20.69 | 28.37 | 30.45 |
| 3obq | 24.10 | 24.42 | 21.51 | 28.41 | 22.81 | 23.15 | 26.52 | 19.39 | 11.70 | 21.38 | 16.84 | 32.12 | 28.93 | 8.37 | 21.00 | 14.64 | 29.99 | 34.17 | 18.81 | 32.25 |
| 3olf | 21.77 | 20.71 | 14.41 | 1.37 | 35.23 | 20.23 | 32.47 | 24.29 | 36.35 | 33.20 | 17.97 | 9.15 | 22.32 | 23.61 | 35.04 | 42.85 | 32.88 | 38.59 | 13.95 | 24.11 |
| 3p72 | 35.91 | 28.23 | 34.00 | 35.92 | 26.65 | 34.08 | 35.60 | 24.88 | 26.70 | 34.83 | 27.27 | 35.56 | 35.02 | 26.94 | 27.33 | 26.91 | 28.90 | 34.77 | 27.47 | 38.14 |
| 3p8f | 19.05 | 15.77 | 7.49 | 37.39 | 12.05 | 27.72 | 17.23 | 32.16 | 38.42 | 36.37 | 15.33 | 17.77 | 18.68 | 36.20 | 37.38 | 34.96 | 18.68 | 13.11 | 14.87 | 6.73 |
| 3ptl | 27.01 | 24.77 | 26.38 | 26.82 | 25.15 | 18.47 | 30.16 | 34.99 | 34.23 | 18.38 | 19.59 | 29.46 | 33.90 | 28.94 | 25.10 | 37.42 | 24.83 | 39.42 | 41.36 | 30.52 |
| 3qis | 15.36 | 9.48 | 22.76 | 14.87 | 13.22 | 21.56 | 16.98 | 15.86 | 12.16 | 12.87 | 15.43 | 13.17 | 14.02 | 10.99 | 26.97 | 16.82 | 9.73 | 11.88 | 15.94 | 15.87 |
| 3rm1 | 1.44 | 16.97 | 18.83 | 5.90 | 10.40 | 19.78 | 21.09 | 8.86 | 8.19 | 18.86 | 18.94 | 8.15 | 8.31 | 18.89 | 19.11 | 18.61 | 19.16 | 17.06 | 11.09 | 19.07 |
| 3rqg | 40.31 | 43.14 | 40.88 | 41.05 | 41.49 | 38.89 | 41.26 | 43.51 | 43.42 | 42.22 | 30.79 | 43.37 | 41.25 | 48.92 | 44.61 | 43.52 | 30.73 | 43.47 | 43.98 | 39.46 |
| 3sfj | 30.62 | 32.38 | 32.50 | 24.01 | 25.62 | 26.12 | 24.91 | 28.78 | 30.01 | 23.64 | 22.75 | 25.05 | 32.52 | 31.60 | 17.32 | 31.28 | 35.35 | 27.83 | 30.42 | 29.87 |
| 3so6 | 51.18 | 52.52 | 51.30 | 50.11 | 52.35 | 51.16 | 53.41 | 53.43 | 54.71 | 52.18 | 52.27 | 52.83 | 52.74 | 51.73 | 51.35 | 52.23 | 54.46 | 53.12 | 54.89 | 54.16 |
| 3tjv | 1.18 | 32.14 | 30.31 | 32.02 | 29.42 | 21.99 | 16.60 | 10.70 | 24.63 | 10.84 | 31.06 | 16.41 | 17.61 | 3.82 | 19.33 | 31.37 | 30.25 | 13.27 | 29.97 | 17.54 |
| 3tzy | 45.84 | 46.14 | 46.78 | 45.71 | 47.98 | 42.06 | 44.86 | 35.33 | 47.00 | 48.86 | 49.47 | 45.05 | 48.09 | 48.26 | 47.00 | 48.26 | 49.50 | 48.81 | 47.77 | 46.60 |
| 3u9q | 0.88 | 25.82 | 3.26 | 29.34 | 28.77 | 34.05 | 4.84 | 27.39 | 8.76 | 5.97 | 27.51 | 37.89 | 34.12 | 28.81 | 6.10 | 40.21 | 33.94 | 22.18 | 26.06 | 34.79 |
| 3up3 | 21.94 | 29.60 | 31.20 | 28.76 | 20.63 | 2.64 | 27.99 | 28.17 | 21.30 | 24.82 | 16.17 | 34.49 | 29.77 | 2.11 | 28.42 | 25.79 | 30.85 | 28.43 | 29.43 | 21.50 |
| 3v2x | 7.71 | 12.52 | 20.78 | 24.80 | 1.54 | 25.18 | 23.62 | 9.73 | 26.22 | 7.60 | 8.51 | 16.12 | 7.51 | 28.94 | 7.43 | 26.99 | 14.36 | 23.79 | 8.86 | 13.32 |
| 3vtc | 22.20 | 18.38 | 36.38 | 37.63 | 36.53 | 33.79 | 26.08 | 23.88 | 33.62 | 25.01 | 40.60 | 20.98 | 34.36 | 24.44 | 39.44 | 44.32 | 27.92 | 19.60 | 41.69 | 34.55 |
| 3w1b | 65.15 | 65.89 | 65.95 | 65.12 | 64.93 | 65.17 | 65.55 | 36.67 | 66.61 | 66.99 | 67.30 | 65.51 | 37.14 | 66.08 | 66.28 | 65.98 | 66.46 | 64.91 | 66.76 | 65.69 |
| 3zqh | 21.89 | 33.39 | 29.25 | 29.12 | 16.20 | 25.76 | 30.76 | 21.62 | 26.57 | 14.02 | 14.34 | 29.64 | 22.11 | 29.56 | 30.37 | 29.94 | 17.49 | 12.22 | 9.36 | 28.82 |
| 4b4n | 26.05 | 33.49 | 11.18 | 30.69 | 13.29 | 30.99 | 30.48 | 10.77 | 12.35 | 31.56 | 12.84 | 12.38 | 26.33 | 31.65 | 2.21 | 18.43 | 32.08 | 31.94 | 10.60 | 18.24 |
| 4dcb | 49.74 | 23.92 | 23.11 | 27.83 | 48.35 | 48.92 | 15.91 | 14.32 | 50.46 | 49.00 | 27.82 | 47.79 | 14.33 | 27.34 | 50.90 | 25.78 | 48.01 | 50.19 | 22.86 | 50.30 |
| 4e34 | 16.99 | 38.06 | 23.91 | 35.47 | 24.99 | 20.39 | 27.11 | 28.64 | 37.58 | 21.03 | 25.64 | 14.63 | 26.59 | 25.23 | 27.21 | 24.91 | 28.13 | 35.31 | 17.38 | 25.91 |
| 4eik | 30.31 | 30.08 | 29.39 | 25.75 | 28.75 | 27.31 | 26.88 | 24.37 | 26.57 | 23.95 | 26.98 | 26.47 | 26.95 | 26.73 | 26.48 | 28.00 | 27.10 | 28.60 | 23.31 | 29.37 |
| 4ery | 28.47 | 32.90 | 31.03 | 32.35 | 29.77 | 31.98 | 32.69 | 32.44 | 29.58 | 33.57 | 34.83 | 11.55 | 34.19 | 35.13 | 32.59 | 33.58 | 16.11 | 32.85 | 3.65 | 37.72 |
| 4f14 | 19.69 | 18.67 | 18.95 | 22.76 | 21.15 | 22.53 | 15.77 | 18.58 | 15.81 | 8.51 | 22.75 | 20.23 | 21.47 | 20.74 | 30.00 | 22.64 | 19.06 | 24.03 | 20.14 | 20.52 |
| 4f1z | 20.97 | 26.46 | 9.31 | 20.74 | 19.94 | 27.64 | 25.45 | 25.42 | 24.54 | 26.32 | 24.81 | 23.82 | 18.81 | 20.83 | 22.86 | 25.50 | 17.12 | 31.78 | 29.76 | 28.26 |
| 4gq6 | 9.79 | 7.75 | 10.79 | 11.27 | 9.47 | 9.21 | 9.79 | 10.85 | 12.64 | 16.55 | 6.09 | 7.42 | 12.55 | 14.17 | 11.72 | 14.27 | 10.90 | 11.95 | 8.69 | 10.70 |
| 4gxl | 31.19 | 28.33 | 31.58 | 13.62 | 28.26 | 26.63 | 3.15 | 30.95 | 29.87 | 11.77 | 23.37 | 28.22 | 7.60 | 30.88 | 30.02 | 31.84 | 13.58 | 31.20 | 29.70 | 31.30 |
| 4gyw | 24.64 | 24.05 | 18.30 | 19.23 | 26.13 | 24.05 | 27.37 | 15.11 | 25.42 | 21.16 | 15.43 | 14.47 | 18.08 | 24.84 | 22.67 | 25.14 | 32.77 | 17.23 | 18.01 | 16.39 |
| 4h4f | 65.52 | 64.38 | 63.22 | 63.51 | 64.35 | 68.21 | 66.84 | 63.10 | 62.79 | 64.27 | 67.21 | 67.54 | 64.53 | 62.88 | 65.52 | 63.58 | 68.10 | 64.39 | 65.44 | 70.49 |
| 4hom | 48.43 | 48.21 | 50.12 | 48.46 | 48.10 | 47.88 | 48.29 | 47.22 | 49.07 | 49.10 | 54.53 | 49.95 | 49.94 | 47.95 | 50.09 | 49.01 | 48.40 | 55.19 | 47.96 | 47.67 |
| 4htp | 34.53 | 38.18 | 27.50 | 44.07 | 26.65 | 35.14 | 28.58 | 32.89 | 36.23 | 17.85 | 38.16 | 23.82 | 18.35 | 22.33 | 16.62 | 25.25 | 24.01 | 30.58 | 36.61 | 36.67 |
| 4iim | 1.49 | 19.28 | 14.49 | 6.38 | 20.50 | 2.98 | 24.83 | 22.18 | 23.97 | 11.28 | 22.17 | 20.56 | 23.74 | 14.16 | 24.63 | 24.57 | 28.49 | 10.30 | 23.76 | 4.36 |
| 4j8s | 45.68 | 49.99 | 48.54 | 36.75 | 49.56 | 49.49 | 50.78 | 50.23 | 47.22 | 47.61 | 41.95 | 38.97 | 47.28 | 48.64 | 49.71 | 39.49 | 41.49 | 40.94 | 44.01 | 49.58 |
| 4k0u | 1.39 | 7.57 | 4.83 | 4.55 | 13.68 | 14.23 | 14.41 | 14.61 | 5.62 | 13.76 | 4.31 | 11.03 | 4.09 | 5.93 | 9.34 | 13.07 | 12.30 | 13.83 | 13.90 | 14.24 |

**S5(c). I-RMSD values of all 20 poses obtained after blind docking by ZDOCK on 133 protein-peptide complexes.**

| **ID** | **Pose1** | **Pose2** | **Pose3** | **Pose4** | **Pose5** | **Pose6** | **Pose7** | **Pose8** | **Pose9** | **Pose10** | **Pose11** | **Pose12** | **Pose13** | **Pose14** | **Pose15** | **Pose16** | **Pose17** | **Pose18** | **Pose19** | **Pose20** |
| --- | --- | --- | --- | --- | --- | --- | --- | --- | --- | --- | --- | --- | --- | --- | --- | --- | --- | --- | --- | --- |
| 1cjr | 1.42 | 2.80 | 1.46 | 1.54 | 23.99 | 1.94 | 25.98 | 26.23 | 24.52 | 26.36 | 24.48 | 25.82 | 2.68 | 24.27 | 23.35 | 13.60 | 25.47 | 26.52 | 26.08 | 24.54 |
| 1cka | 0.60 | 1.88 | 2.28 | 15.55 | 15.48 | 1.73 | 15.78 | 2.87 | 17.13 | 16.95 | 17.23 | 15.33 | 4.55 | 2.39 | 15.35 | 4.10 | 8.54 | 8.93 | 5.27 | 2.51 |
| 1cvu | 37.16 | 39.67 | 37.00 | 38.19 | 37.28 | 38.49 | 37.27 | 37.43 | 36.91 | 38.77 | 37.38 | 38.03 | 38.72 | 37.41 | 37.36 | 62.31 | 38.80 | 36.61 | 37.44 | 37.22 |
| 1d4t | 2.42 | 1.71 | 12.72 | 4.01 | 9.66 | 18.56 | 6.38 | 11.83 | 12.41 | 8.77 | 20.72 | 11.09 | 4.15 | 7.20 | 7.60 | 2.81 | 6.23 | 9.51 | 14.50 | 11.14 |
| 1eg4 | 45.30 | 45.40 | 40.99 | 43.22 | 42.45 | 47.17 | 42.00 | 43.59 | 44.57 | 43.30 | 42.47 | 41.61 | 46.58 | 46.58 | 42.29 | 44.08 | 45.45 | 45.80 | 45.77 | 44.17 |
| 1h6w | 0.95 | 1.20 | 1.98 | 1.80 | 1.88 | 2.09 | 1.44 | 16.70 | 16.50 | 12.36 | 2.84 | 18.33 | 16.16 | 18.54 | 16.75 | 12.80 | 18.67 | 19.52 | 18.10 | 16.06 |
| 1hc9 | 21.66 | 0.93 | 20.04 | 2.94 | 19.25 | 21.24 | 17.24 | 16.99 | 18.55 | 16.64 | 19.05 | 20.60 | 21.69 | 17.00 | 18.00 | 21.29 | 16.07 | 21.97 | 21.37 | 19.63 |
| 1jbu | 1.90 | 1.88 | 41.37 | 45.38 | 2.51 | 45.73 | 46.02 | 45.84 | 46.00 | 47.19 | 45.26 | 44.62 | 46.10 | 45.48 | 45.57 | 45.70 | 45.14 | 46.65 | 46.50 | 46.32 |
| 1k5n | 1.77 | 2.28 | 25.65 | 23.16 | 2.96 | 22.29 | 21.35 | 23.10 | 22.42 | 22.52 | 13.90 | 23.51 | 22.96 | 5.60 | 22.87 | 21.82 | 24.95 | 26.24 | 23.28 | 23.43 |
| 1mfg | 27.27 | 26.61 | 23.80 | 26.43 | 28.53 | 23.82 | 26.24 | 22.72 | 25.03 | 26.56 | 26.96 | 24.67 | 28.34 | 22.96 | 27.48 | 29.33 | 26.16 | 23.11 | 1.90 | 29.00 |
| 1nln | 29.69 | 30.54 | 28.50 | 33.21 | 31.09 | 31.20 | 32.78 | 34.92 | 33.14 | 33.46 | 35.98 | 29.78 | 31.00 | 30.05 | 33.40 | 30.84 | 33.63 | 30.30 | 38.45 | 29.52 |
| 1nq7 | 36.80 | 38.01 | 36.42 | 39.52 | 22.17 | 37.35 | 21.14 | 22.67 | 37.97 | 35.39 | 44.04 | 37.76 | 36.41 | 36.95 | 21.01 | 36.17 | 43.43 | 36.69 | 43.51 | 43.88 |
| 1ntv | 3.55 | 16.28 | 15.13 | 9.91 | 10.29 | 5.75 | 1.04 | 14.78 | 15.06 | 14.63 | 15.05 | 9.44 | 11.65 | 15.20 | 3.58 | 16.11 | 14.69 | 15.81 | 1.73 | 16.67 |
| 1nx1 | 34.73 | 35.51 | 33.94 | 33.05 | 35.44 | 35.34 | 33.60 | 34.31 | 36.27 | 39.98 | 32.30 | 35.46 | 0.93 | 34.82 | 36.78 | 36.63 | 35.67 | 35.55 | 38.90 | 33.77 |
| 1oai | 23.75 | 24.39 | 3.96 | 1.56 | 20.46 | 24.40 | 23.90 | 21.79 | 2.87 | 19.78 | 23.48 | 24.01 | 23.40 | 24.05 | 18.66 | 24.04 | 23.50 | 13.67 | 23.23 | 23.67 |
| 1oj5 | 16.07 | 14.58 | 13.53 | 15.14 | 14.56 | 5.62 | 13.18 | 7.47 | 16.31 | 9.02 | 4.76 | 16.28 | 13.23 | 13.99 | 15.84 | 13.52 | 17.06 | 9.92 | 16.11 | 12.07 |
| 1ou8 | 7.09 | 8.51 | 8.66 | 8.57 | 8.73 | 13.68 | 9.43 | 8.04 | 10.36 | 14.86 | 7.25 | 9.54 | 9.81 | 14.76 | 28.50 | 13.65 | 15.14 | 10.16 | 15.28 | 12.15 |
| 1ow6 | 36.71 | 37.27 | 17.06 | 33.83 | 36.78 | 37.20 | 37.13 | 37.70 | 16.21 | 36.75 | 37.95 | 35.20 | 36.98 | 37.70 | 36.65 | 37.94 | 37.80 | 34.10 | 38.13 | 17.73 |
| 1pzl | 39.52 | 37.27 | 38.85 | 37.67 | 39.17 | 39.31 | 38.07 | 37.73 | 40.69 | 40.12 | 39.60 | 37.34 | 40.12 | 37.17 | 40.69 | 39.19 | 40.84 | 40.85 | 41.50 | 38.99 |
| 1qkz | 8.92 | 8.83 | 9.55 | 8.33 | 8.70 | 8.22 | 8.46 | 9.52 | 9.65 | 8.97 | 9.28 | 9.52 | 9.76 | 4.62 | 4.90 | 9.20 | 9.32 | 8.17 | 9.65 | 9.85 |
| 1rst | 7.41 | 3.09 | 10.02 | 2.19 | 7.51 | 10.31 | 9.46 | 9.96 | 8.33 | 2.54 | 7.71 | 3.12 | 6.73 | 2.69 | 3.38 | 7.06 | 9.41 | 6.30 | 3.76 | 7.20 |
| 1rxz | 13.24 | 12.30 | 1.89 | 3.94 | 13.63 | 41.13 | 4.46 | 1.55 | 6.39 | 13.14 | 3.44 | 38.97 | 13.14 | 20.50 | 2.48 | 11.96 | 17.36 | 9.42 | 41.79 | 31.43 |
| 1sfi | 15.18 | 11.86 | 14.31 | 15.21 | 21.12 | 17.06 | 18.15 | 17.11 | 21.84 | 23.87 | 13.77 | 22.30 | 23.99 | 15.58 | 20.81 | 12.31 | 14.89 | 19.23 | 17.57 | 11.11 |
| 1ssh | 1.85 | 1.85 | 1.48 | 18.78 | 4.93 | 18.76 | 9.08 | 18.60 | 20.47 | 19.76 | 2.51 | 3.35 | 5.48 | 5.28 | 6.69 | 1.30 | 3.67 | 4.00 | 3.06 | 18.58 |
| 1t08 | 24.50 | 25.95 | 37.35 | 34.85 | 25.85 | 24.78 | 19.84 | 23.89 | 24.95 | 23.26 | 15.46 | 30.71 | 26.74 | 25.42 | 28.87 | 20.12 | 39.75 | 25.90 | 24.76 | 24.81 |
| 1t4f | 1.05 | 1.69 | 1.87 | 0.72 | 2.04 | 8.55 | 9.10 | 10.94 | 2.94 | 1.37 | 21.19 | 19.01 | 5.59 | 4.48 | 1.66 | 12.58 | 12.12 | 19.29 | 6.79 | 12.11 |
| 1t7r | 29.38 | 40.37 | 2.94 | 1.68 | 35.77 | 38.28 | 38.17 | 5.50 | 42.99 | 37.72 | 1.22 | 2.61 | 38.86 | 38.60 | 39.41 | 39.01 | 27.76 | 39.20 | 27.61 | 40.36 |
| 1tfc | 1.90 | 2.59 | 27.88 | 24.35 | 25.27 | 25.97 | 3.16 | 24.77 | 26.60 | 25.07 | 27.87 | 19.49 | 26.50 | 36.13 | 3.54 | 24.49 | 1.65 | 1.18 | 27.25 | 24.88 |
| 1u00 | 2.92 | 1.46 | 2.81 | 1.46 | 3.01 | 7.46 | 2.70 | 2.76 | 6.55 | 2.21 | 3.66 | 3.08 | 6.05 | 18.92 | 5.91 | 4.82 | 2.63 | 33.23 | 7.35 | 33.20 |
| 1uj0 | 1.85 | 0.78 | 2.63 | 16.82 | 2.20 | 2.92 | 2.26 | 16.66 | 17.01 | 16.54 | 16.97 | 16.60 | 16.35 | 9.04 | 13.49 | 16.44 | 13.75 | 5.71 | 4.99 | 16.45 |
| 1x2r | 8.57 | 2.33 | 3.89 | 7.98 | 4.35 | 2.89 | 7.97 | 9.38 | 2.70 | 7.74 | 8.64 | 6.34 | 7.39 | 8.06 | 7.53 | 6.14 | 2.35 | 4.99 | 8.19 | 4.11 |
| 1xoc | 24.97 | 1.10 | 33.48 | 1.17 | 32.76 | 24.76 | 28.40 | 25.58 | 22.90 | 23.22 | 25.50 | 25.28 | 21.05 | 29.32 | 31.71 | 24.67 | 27.47 | 24.41 | 25.90 | 32.80 |
| 1ymt | 1.19 | 1.38 | 7.35 | 2.53 | 1.18 | 3.06 | 1.87 | 7.59 | 1.81 | 24.46 | 24.43 | 22.74 | 23.62 | 4.87 | 24.01 | 22.14 | 1.70 | 1.54 | 23.95 | 2.93 |
| 1yuc | 1.94 | 13.25 | 16.36 | 23.66 | 23.87 | 2.07 | 24.90 | 16.79 | 35.03 | 1.33 | 29.27 | 32.73 | 25.21 | 23.73 | 27.13 | 26.72 | 22.81 | 33.55 | 24.40 | 14.70 |
| 1ywo | 15.01 | 4.11 | 5.11 | 4.61 | 15.34 | 18.05 | 15.14 | 13.33 | 4.94 | 13.34 | 4.35 | 14.83 | 4.93 | 15.70 | 4.63 | 4.87 | 14.76 | 1.53 | 15.01 | 16.75 |
| 2a25 | 26.27 | 27.93 | 26.96 | 30.91 | 25.04 | 29.27 | 27.24 | 27.39 | 25.68 | 27.31 | 25.88 | 27.77 | 26.10 | 27.55 | 3.85 | 25.21 | 29.16 | 25.81 | 27.39 | 27.27 |
| 2a3i | 35.77 | 36.81 | 36.41 | 38.04 | 36.63 | 37.36 | 36.42 | 37.56 | 36.57 | 36.67 | 36.25 | 37.80 | 36.66 | 36.93 | 37.92 | 38.22 | 37.12 | 37.17 | 37.13 | 35.90 |
| 2aq9 | 16.57 | 19.80 | 14.17 | 20.04 | 32.49 | 12.70 | 29.01 | 14.78 | 19.81 | 16.65 | 18.53 | 13.02 | 14.21 | 20.16 | 19.88 | 29.43 | 32.58 | 32.51 | 31.29 | 35.33 |
| 2b9h | 30.73 | 1.18 | 3.03 | 1.97 | 43.28 | 29.01 | 43.47 | 41.09 | 46.98 | 39.85 | 26.42 | 40.40 | 29.85 | 32.24 | 49.51 | 34.79 | 46.98 | 34.34 | 27.83 | 43.54 |
| 2bba | 29.86 | 30.33 | 37.33 | 30.68 | 30.10 | 29.64 | 28.03 | 29.47 | 31.39 | 30.26 | 29.73 | 29.33 | 29.76 | 29.02 | 30.65 | 29.34 | 38.83 | 27.80 | 30.19 | 30.42 |
| 2cch | 0.85 | 26.69 | 37.13 | 36.37 | 35.83 | 26.82 | 35.13 | 20.31 | 26.30 | 37.96 | 36.57 | 27.84 | 26.82 | 37.59 | 35.06 | 41.62 | 38.70 | 35.72 | 28.78 | 36.88 |
| 2ce8 | 36.07 | 34.51 | 34.22 | 33.09 | 35.63 | 34.13 | 36.33 | 36.77 | 37.37 | 35.28 | 35.14 | 35.83 | 38.52 | 37.27 | 33.48 | 37.25 | 36.65 | 39.39 | 38.88 | 38.23 |
| 2d0n | 1.30 | 2.43 | 1.62 | 2.71 | 2.31 | 3.34 | 3.62 | 2.94 | 3.70 | 4.40 | 16.96 | 1.71 | 16.25 | 2.97 | 2.30 | 11.66 | 2.53 | 20.05 | 29.06 | 1.97 |
| 2drk | 1.54 | 2.23 | 3.55 | 1.18 | 17.53 | 9.83 | 1.89 | 17.46 | 17.51 | 17.25 | 20.16 | 17.63 | 17.18 | 18.05 | 13.76 | 1.58 | 19.25 | 17.83 | 17.21 | 16.98 |
| 2dyp | 2.19 | 1.86 | 1.40 | 1.40 | 19.71 | 23.71 | 23.70 | 2.73 | 1.87 | 19.99 | 1.90 | 20.37 | 21.00 | 22.54 | 61.90 | 21.10 | 18.92 | 20.24 | 20.80 | 61.58 |
| 2fff | 51.16 | 51.27 | 52.74 | 50.32 | 52.65 | 49.12 | 51.48 | 49.73 | 49.81 | 51.82 | 53.02 | 48.82 | 51.39 | 52.47 | 52.98 | 51.03 | 51.97 | 51.88 | 50.76 | 51.08 |
| 2ffu | 15.79 | 1.84 | 1.92 | 15.97 | 15.55 | 15.59 | 15.62 | 1.49 | 15.89 | 15.69 | 4.30 | 1.72 | 16.22 | 3.00 | 4.42 | 4.50 | 15.76 | 2.16 | 9.80 | 3.50 |
| 2fka | 29.19 | 7.10 | 29.43 | 5.46 | 27.28 | 25.28 | 25.32 | 26.26 | 25.67 | 26.82 | 6.98 | 25.04 | 26.35 | 28.59 | 4.80 | 25.81 | 0.86 | 26.01 | 29.74 | 7.00 |
| 2fmf | 26.52 | 30.66 | 7.75 | 31.98 | 4.34 | 31.26 | 30.03 | 31.74 | 4.66 | 30.13 | 30.93 | 29.85 | 28.87 | 28.09 | 27.91 | 26.73 | 28.34 | 32.39 | 28.92 | 30.07 |
| 2fts | 0.49 | 1.62 | 3.33 | 3.38 | 2.32 | 1.79 | 4.90 | 3.12 | 6.41 | 5.19 | 13.21 | 16.25 | 15.99 | 15.21 | 3.12 | 17.25 | 11.67 | 29.13 | 28.55 | 13.42 |
| 2fvj | 43.60 | 46.15 | 43.74 | 43.07 | 42.98 | 44.02 | 45.09 | 44.21 | 42.77 | 42.72 | 44.48 | 46.88 | 44.64 | 43.93 | 43.19 | 45.27 | 43.42 | 43.00 | 45.64 | 45.61 |
| 2ho2 | 5.73 | 6.51 | 5.64 | 3.98 | 12.78 | 12.60 | 6.33 | 5.40 | 11.97 | 13.28 | 13.19 | 5.47 | 12.37 | 17.03 | 12.64 | 12.06 | 12.57 | 8.02 | 4.98 | 8.31 |
| 2ht9 | 29.25 | 28.71 | 27.34 | 26.70 | 31.19 | 28.17 | 26.78 | 27.77 | 25.38 | 25.79 | 23.02 | 24.02 | 30.54 | 33.86 | 25.43 | 25.32 | 30.13 | 25.10 | 28.95 | 29.77 |
| 2o02 | 33.32 | 33.09 | 34.04 | 33.04 | 19.50 | 31.91 | 22.85 | 32.52 | 34.80 | 31.61 | 34.86 | 31.59 | 30.86 | 31.22 | 33.29 | 35.10 | 22.47 | 32.59 | 38.01 | 37.86 |
| 2o4j | 34.13 | 33.41 | 33.80 | 34.23 | 33.67 | 34.05 | 35.28 | 33.42 | 32.46 | 33.53 | 34.17 | 34.18 | 32.00 | 34.09 | 34.47 | 33.41 | 32.83 | 32.75 | 34.56 | 33.43 |
| 2o9v | 4.07 | 17.41 | 4.16 | 4.16 | 17.41 | 5.24 | 21.00 | 5.53 | 4.08 | 21.04 | 1.37 | 4.85 | 18.94 | 17.43 | 4.11 | 22.43 | 5.47 | 17.03 | 17.69 | 1.41 |
| 2oei | 8.83 | 13.59 | 3.22 | 9.08 | 9.08 | 15.04 | 9.05 | 3.39 | 6.23 | 13.27 | 3.10 | 3.31 | 15.00 | 13.61 | 13.29 | 15.63 | 15.99 | 4.33 | 3.26 | 2.39 |
| 2p0w | 37.19 | 26.07 | 23.10 | 32.69 | 39.01 | 22.32 | 25.97 | 22.30 | 31.96 | 33.37 | 30.00 | 27.40 | 40.50 | 32.34 | 33.82 | 28.43 | 32.43 | 31.21 | 29.95 | 25.88 |
| 2p1o | 11.63 | 11.42 | 1.20 | 9.74 | 2.80 | 10.03 | 1.86 | 12.16 | 11.10 | 9.44 | 8.71 | 12.43 | 5.19 | 11.99 | 11.64 | 9.30 | 10.01 | 9.11 | 11.37 | 8.71 |
| 2p1t | 0.95 | 7.29 | 2.38 | 35.22 | 35.98 | 6.67 | 35.44 | 5.36 | 7.25 | 34.95 | 25.34 | 35.25 | 1.98 | 35.57 | 5.94 | 35.61 | 35.05 | 35.26 | 10.68 | 35.23 |
| 2p54 | 43.18 | 44.20 | 42.14 | 39.94 | 40.28 | 41.04 | 44.74 | 43.77 | 27.39 | 31.54 | 41.87 | 41.46 | 31.46 | 43.83 | 44.08 | 45.11 | 44.16 | 26.53 | 45.21 | 27.90 |
| 2peh | 1.04 | 1.63 | 2.23 | 3.47 | 2.84 | 2.24 | 2.32 | 2.98 | 2.86 | 3.75 | 3.34 | 2.05 | 8.16 | 4.55 | 3.34 | 3.28 | 4.27 | 3.64 | 3.74 | 12.08 |
| 2pux | 32.43 | 31.65 | 33.30 | 30.50 | 35.49 | 33.29 | 37.77 | 40.76 | 35.61 | 32.61 | 32.18 | 34.16 | 34.02 | 1.20 | 32.48 | 46.17 | 33.11 | 32.64 | 32.51 | 34.30 |
| 2puy | 2.31 | 18.49 | 15.77 | 13.62 | 2.84 | 20.10 | 14.70 | 17.43 | 18.55 | 31.35 | 23.37 | 16.57 | 16.04 | 27.68 | 1.61 | 4.13 | 14.79 | 10.76 | 15.99 | 1.44 |
| 2qbx | 38.85 | 38.14 | 35.38 | 35.64 | 39.11 | 40.68 | 37.34 | 37.89 | 38.42 | 39.47 | 38.01 | 37.20 | 35.85 | 38.52 | 39.44 | 37.81 | 39.50 | 39.40 | 38.39 | 35.23 |
| 2qos | 11.10 | 1.96 | 10.90 | 10.67 | 2.06 | 4.89 | 1.97 | 10.51 | 1.59 | 4.48 | 7.37 | 5.63 | 11.55 | 11.23 | 8.77 | 11.86 | 9.83 | 10.65 | 11.02 | 10.63 |
| 2qse | 31.78 | 30.35 | 31.51 | 30.20 | 30.78 | 32.38 | 28.87 | 29.23 | 30.94 | 33.79 | 31.40 | 0.66 | 33.34 | 30.54 | 32.50 | 29.93 | 30.29 | 32.99 | 31.09 | 31.07 |
| 2r7g | 38.61 | 37.27 | 37.67 | 37.80 | 39.04 | 37.97 | 40.05 | 39.01 | 38.24 | 0.98 | 1.86 | 1.84 | 37.40 | 39.43 | 37.57 | 37.78 | 37.48 | 2.08 | 37.34 | 1.66 |
| 2r9q | 10.48 | 7.05 | 8.36 | 7.25 | 9.38 | 10.60 | 7.38 | 9.71 | 9.39 | 7.77 | 4.57 | 9.80 | 7.28 | 2.90 | 10.89 | 7.48 | 8.00 | 8.51 | 1.01 | 8.69 |
| 2v8y | 2.20 | 42.10 | 42.44 | 43.89 | 43.54 | 42.44 | 43.60 | 42.84 | 42.47 | 17.95 | 39.33 | 42.73 | 0.69 | 42.42 | 44.04 | 43.26 | 18.18 | 15.21 | 42.90 | 39.01 |
| 2vkn | 1.73 | 1.34 | 2.25 | 15.75 | 15.43 | 1.58 | 15.40 | 15.45 | 3.22 | 5.62 | 15.59 | 15.37 | 1.65 | 15.23 | 15.62 | 3.19 | 15.78 | 2.36 | 15.70 | 3.99 |
| 2vr3 | 1.47 | 28.63 | 24.56 | 27.72 | 1.86 | 36.72 | 23.26 | 32.63 | 23.35 | 28.63 | 32.04 | 28.36 | 36.35 | 35.06 | 36.43 | 29.91 | 31.82 | 26.41 | 31.21 | 36.66 |
| 2vwf | 0.93 | 1.93 | 3.26 | 21.85 | 2.68 | 22.67 | 20.10 | 21.75 | 17.13 | 22.06 | 21.57 | 22.52 | 11.34 | 24.67 | 20.57 | 11.24 | 2.23 | 21.43 | 4.90 | 21.61 |
| 2w2u | 27.18 | 9.98 | 27.86 | 26.74 | 27.21 | 26.32 | 27.54 | 23.27 | 23.29 | 28.36 | 26.78 | 22.13 | 25.44 | 26.84 | 15.16 | 27.68 | 15.22 | 21.26 | 22.22 | 27.41 |
| 2whx | 1.10 | 3.85 | 6.95 | 2.81 | 26.57 | 7.03 | 4.27 | 6.91 | 24.61 | 7.41 | 22.79 | 25.44 | 26.20 | 26.00 | 29.20 | 2.31 | 21.11 | 25.87 | 23.42 | 23.13 |
| 2xrw | 1.18 | 2.63 | 1.25 | 4.14 | 41.02 | 38.41 | 23.36 | 1.74 | 38.15 | 32.35 | 23.54 | 47.41 | 23.03 | 39.10 | 49.75 | 31.29 | 39.96 | 22.49 | 32.59 | 46.69 |
| 2xu7 | 1.18 | 2.73 | 6.46 | 37.64 | 2.72 | 4.30 | 4.87 | 7.65 | 5.26 | 6.49 | 16.68 | 19.25 | 38.80 | 4.73 | 38.09 | 16.61 | 29.35 | 6.31 | 11.63 | 5.13 |
| 2xvc | 3.75 | 2.37 | 21.17 | 25.18 | 20.86 | 18.85 | 24.09 | 21.43 | 6.63 | 24.74 | 23.83 | 24.61 | 19.85 | 28.26 | 28.79 | 21.00 | 34.49 | 23.10 | 28.98 | 21.19 |
| 2zjd | 0.63 | 27.56 | 28.90 | 27.92 | 31.18 | 28.71 | 31.62 | 33.23 | 27.21 | 32.67 | 32.69 | 30.79 | 28.42 | 32.04 | 29.24 | 33.49 | 2.32 | 30.02 | 34.16 | 32.49 |
| 3asl | 14.82 | 9.31 | 9.95 | 12.55 | 13.14 | 10.31 | 9.52 | 21.05 | 14.03 | 12.37 | 17.88 | 11.40 | 9.03 | 12.52 | 14.64 | 22.59 | 24.42 | 20.08 | 9.39 | 12.82 |
| 3awr | 15.25 | 15.73 | 15.60 | 14.85 | 25.11 | 24.23 | 15.45 | 18.24 | 24.68 | 11.17 | 14.99 | 25.68 | 14.27 | 24.46 | 15.76 | 15.79 | 15.47 | 14.82 | 15.76 | 23.88 |
| 3ayu | 1.20 | 1.38 | 1.09 | 2.54 | 4.38 | 3.61 | 17.41 | 23.34 | 2.44 | 8.70 | 8.43 | 2.76 | 8.26 | 1.78 | 6.78 | 10.43 | 6.38 | 1.66 | 4.12 | 20.99 |
| 3bfq | 0.88 | 0.95 | 27.98 | 15.79 | 15.89 | 14.24 | 9.26 | 0.88 | 15.08 | 27.54 | 5.74 | 15.28 | 27.97 | 5.24 | 40.28 | 33.00 | 27.61 | 17.69 | 5.19 | 20.17 |
| 3c3r | 38.63 | 37.66 | 37.56 | 0.80 | 42.64 | 38.11 | 38.29 | 38.96 | 38.18 | 39.49 | 39.85 | 39.84 | 39.74 | 38.38 | 36.69 | 36.48 | 38.14 | 38.54 | 37.69 | 40.09 |
| 3d32 | 1.19 | 2.71 | 16.97 | 17.98 | 19.17 | 25.58 | 28.65 | 1.68 | 16.43 | 18.70 | 18.70 | 23.40 | 15.74 | 21.68 | 17.43 | 24.61 | 21.67 | 16.03 | 32.33 | 16.66 |
| 3ds4 | 1.73 | 2.29 | 25.08 | 1.08 | 2.21 | 25.20 | 1.40 | 2.07 | 11.06 | 10.94 | 3.20 | 24.58 | 24.80 | 3.49 | 23.25 | 25.50 | 25.45 | 24.39 | 1.41 | 25.81 |
| 3ery | 2.36 | 2.85 | 1.26 | 1.15 | 2.38 | 2.63 | 3.36 | 5.72 | 3.33 | 3.96 | 6.87 | 5.20 | 2.39 | 8.56 | 5.78 | 9.00 | 15.73 | 6.85 | 1.82 | 11.84 |
| 3fdo | 0.72 | 1.90 | 10.27 | 14.99 | 15.39 | 10.40 | 12.99 | 1.67 | 9.97 | 2.01 | 10.97 | 11.45 | 6.92 | 10.56 | 11.30 | 12.25 | 11.52 | 4.36 | 4.62 | 6.06 |
| 3g2s | 0.31 | 1.51 | 1.78 | 12.31 | 12.90 | 24.29 | 1.57 | 1.20 | 12.88 | 1.29 | 1.48 | 4.72 | 2.93 | 14.17 | 2.02 | 1.72 | 1.85 | 12.88 | 14.49 | 25.53 |
| 3gyt | 1.23 | 1.32 | 1.20 | 1.52 | 9.30 | 45.80 | 44.88 | 45.18 | 1.18 | 4.46 | 45.36 | 43.68 | 44.34 | 42.34 | 45.66 | 44.78 | 45.14 | 43.34 | 1.53 | 45.63 |
| 3h1z | 1.40 | 19.12 | 21.73 | 18.43 | 7.28 | 23.27 | 21.68 | 27.45 | 18.75 | 3.57 | 21.92 | 6.22 | 10.53 | 10.73 | 22.99 | 21.93 | 56.59 | 38.13 | 56.62 | 22.91 |
| 3i5r | 0.56 | 2.19 | 16.24 | 15.69 | 2.64 | 15.69 | 16.14 | 16.46 | 2.55 | 3.69 | 15.51 | 15.72 | 1.46 | 15.67 | 10.85 | 15.63 | 16.19 | 10.49 | 9.94 | 15.48 |
| 3ivv | 13.84 | 15.35 | 1.10 | 14.90 | 12.20 | 14.76 | 15.91 | 14.43 | 14.61 | 11.96 | 12.52 | 14.38 | 12.48 | 10.35 | 14.37 | 2.30 | 15.19 | 14.11 | 16.71 | 13.96 |
| 3kmr | 0.97 | 34.89 | 33.27 | 34.60 | 1.94 | 1.59 | 36.27 | 36.24 | 35.45 | 36.63 | 1.92 | 36.34 | 37.11 | 34.82 | 28.63 | 35.37 | 35.42 | 1.59 | 35.36 | 35.59 |
| 3kuj | 22.81 | 22.34 | 20.18 | 22.60 | 17.43 | 19.26 | 21.29 | 18.56 | 20.46 | 19.79 | 19.93 | 23.66 | 22.55 | 16.99 | 17.86 | 19.00 | 21.95 | 17.71 | 28.23 | 23.69 |
| 3kus | 1.57 | 11.71 | 20.36 | 20.03 | 20.53 | 3.57 | 2.48 | 21.07 | 20.58 | 20.13 | 9.90 | 3.31 | 20.82 | 21.16 | 19.98 | 20.43 | 20.59 | 7.76 | 20.09 | 10.36 |
| 3l0e | 26.24 | 26.06 | 2.55 | 1.56 | 39.88 | 1.62 | 27.19 | 26.10 | 25.94 | 26.20 | 26.47 | 32.45 | 33.33 | 26.44 | 37.74 | 32.82 | 30.08 | 33.29 | 29.43 | 28.08 |
| 3ll8 | 1.28 | 1.67 | 3.96 | 39.63 | 40.90 | 39.27 | 48.15 | 34.12 | 38.14 | 36.57 | 34.47 | 38.37 | 38.28 | 48.25 | 35.51 | 34.74 | 39.74 | 40.74 | 35.52 | 13.22 |
| 3llz | 32.08 | 26.79 | 23.46 | 27.65 | 20.85 | 20.09 | 32.85 | 33.81 | 32.00 | 23.52 | 23.76 | 28.25 | 27.27 | 28.53 | 32.12 | 28.29 | 27.43 | 24.81 | 27.77 | 30.80 |
| 3obq | 16.31 | 21.73 | 21.85 | 22.03 | 1.03 | 6.41 | 17.26 | 21.88 | 15.27 | 20.89 | 21.91 | 19.07 | 17.81 | 17.18 | 22.48 | 17.38 | 21.34 | 21.53 | 17.30 | 2.62 |
| 3olf | 25.63 | 25.28 | 23.60 | 22.71 | 23.02 | 23.72 | 24.60 | 25.55 | 23.33 | 28.15 | 24.33 | 23.89 | 1.23 | 16.16 | 24.71 | 24.56 | 22.72 | 23.05 | 24.74 | 23.90 |
| 3p72 | 5.59 | 5.68 | 6.06 | 41.48 | 1.45 | 7.43 | 41.06 | 40.77 | 39.95 | 5.85 | 7.48 | 41.27 | 39.69 | 6.24 | 41.50 | 40.17 | 8.81 | 6.59 | 40.43 | 9.60 |
| 3p8f | 4.52 | 8.39 | 9.04 | 6.85 | 13.10 | 9.81 | 8.00 | 9.09 | 1.54 | 9.71 | 8.35 | 12.85 | 11.58 | 1.50 | 1.44 | 7.12 | 2.14 | 13.22 | 11.84 | 9.08 |
| 3ptl | 12.28 | 13.18 | 17.72 | 18.40 | 27.75 | 30.17 | 12.88 | 27.46 | 16.30 | 32.21 | 17.94 | 17.07 | 17.20 | 17.90 | 27.50 | 18.02 | 28.07 | 48.32 | 27.51 | 17.90 |
| 3qis | 0.94 | 1.61 | 1.53 | 13.39 | 1.55 | 13.72 | 5.58 | 13.45 | 4.30 | 14.11 | 12.83 | 13.96 | 13.57 | 13.44 | 2.16 | 1.03 | 13.02 | 5.28 | 13.70 | 14.16 |
| 3rm1 | 2.10 | 18.02 | 1.47 | 1.06 | 20.20 | 17.45 | 18.28 | 14.21 | 17.04 | 19.58 | 15.55 | 3.16 | 13.84 | 17.84 | 16.42 | 16.54 | 3.20 | 18.40 | 20.24 | 15.83 |
| 3rqg | 41.22 | 23.94 | 23.95 | 44.14 | 45.46 | 40.26 | 36.75 | 39.42 | 27.92 | 31.94 | 28.46 | 44.74 | 24.58 | 30.90 | 40.46 | 23.36 | 29.16 | 22.98 | 37.83 | 48.18 |
| 3sfj | 3.53 | 3.27 | 20.48 | 4.04 | 5.25 | 1.29 | 14.87 | 5.09 | 1.29 | 1.59 | 14.65 | 18.96 | 19.77 | 30.54 | 25.35 | 23.91 | 24.42 | 28.39 | 30.57 | 31.51 |
| 3so6 | 1.10 | 1.99 | 2.67 | 2.18 | 2.02 | 4.07 | 2.66 | 2.16 | 14.69 | 2.97 | 15.66 | 10.59 | 5.29 | 15.27 | 3.73 | 4.31 | 5.52 | 3.73 | 13.73 | 12.88 |
| 3tjv | 1.23 | 1.52 | 2.35 | 2.13 | 1.98 | 19.73 | 11.61 | 12.21 | 7.51 | 25.66 | 25.34 | 15.22 | 2.88 | 2.75 | 25.82 | 23.83 | 3.33 | 22.83 | 24.02 | 12.88 |
| 3tzy | 47.28 | 9.68 | 47.12 | 47.05 | 47.25 | 47.63 | 47.80 | 46.34 | 48.29 | 47.98 | 47.20 | 13.39 | 47.18 | 46.93 | 46.61 | 46.58 | 9.30 | 47.16 | 47.32 | 10.36 |
| 3u9q | 43.32 | 42.74 | 44.10 | 45.97 | 44.71 | 43.76 | 42.41 | 42.58 | 42.66 | 42.66 | 46.82 | 44.35 | 44.18 | 44.55 | 41.91 | 44.75 | 42.43 | 45.37 | 42.02 | 44.62 |
| 3up3 | 1.01 | 1.32 | 3.42 | 1.62 | 5.41 | 1.87 | 5.55 | 4.72 | 2.92 | 3.18 | 5.23 | 16.86 | 5.24 | 15.11 | 6.41 | 15.19 | 9.75 | 4.65 | 1.69 | 6.30 |
| 3v2x | 1.26 | 0.87 | 2.93 | 30.63 | 8.93 | 30.94 | 25.60 | 17.71 | 4.44 | 28.52 | 22.84 | 16.90 | 30.92 | 25.86 | 31.22 | 25.57 | 31.65 | 1.82 | 11.73 | 9.61 |
| 3vtc | 22.68 | 23.27 | 24.65 | 22.86 | 24.18 | 25.30 | 23.73 | 23.74 | 21.98 | 25.41 | 23.42 | 23.69 | 23.57 | 25.83 | 23.29 | 23.04 | 22.37 | 23.05 | 26.64 | 23.31 |
| 3w1b | 83.70 | 41.44 | 40.31 | 38.59 | 79.46 | 40.01 | 41.59 | 40.24 | 40.84 | 41.71 | 80.65 | 39.84 | 79.74 | 39.77 | 80.70 | 80.50 | 40.51 | 41.67 | 1.07 | 2.03 |
| 3zqh | 33.70 | 27.31 | 35.61 | 19.43 | 25.56 | 34.27 | 20.26 | 10.79 | 11.37 | 28.41 | 14.42 | 25.92 | 27.20 | 28.56 | 23.01 | 14.09 | 10.94 | 21.05 | 26.55 | 27.03 |
| 4b4n | 26.19 | 31.81 | 30.42 | 30.13 | 27.62 | 28.46 | 27.69 | 27.41 | 31.33 | 1.41 | 28.22 | 30.48 | 24.09 | 27.26 | 26.08 | 26.90 | 28.77 | 30.34 | 26.38 | 29.66 |
| 4dcb | 42.77 | 44.35 | 43.29 | 40.20 | 44.30 | 47.63 | 43.19 | 44.04 | 40.73 | 45.36 | 43.78 | 44.20 | 40.65 | 40.45 | 42.32 | 41.27 | 47.44 | 57.31 | 43.17 | 45.57 |
| 4e34 | 18.33 | 21.40 | 21.88 | 19.46 | 20.64 | 15.12 | 18.19 | 19.69 | 19.20 | 17.15 | 27.32 | 24.96 | 21.80 | 22.86 | 21.46 | 16.61 | 17.41 | 22.70 | 24.95 | 21.12 |
| 4eik | 1.12 | 2.05 | 16.39 | 17.22 | 16.37 | 16.78 | 10.45 | 16.34 | 16.36 | 1.52 | 16.64 | 16.64 | 16.48 | 16.33 | 17.08 | 3.93 | 16.45 | 3.78 | 16.40 | 2.43 |
| 4ery | 10.27 | 2.92 | 9.56 | 1.75 | 1.27 | 12.03 | 10.27 | 12.22 | 7.31 | 10.79 | 7.76 | 10.75 | 10.63 | 1.68 | 12.61 | 12.11 | 8.49 | 11.15 | 10.08 | 11.06 |
| 4f14 | 11.01 | 20.17 | 10.98 | 21.45 | 18.57 | 20.38 | 20.73 | 9.85 | 18.47 | 18.39 | 22.35 | 11.73 | 24.59 | 12.74 | 1.13 | 10.45 | 23.14 | 22.38 | 18.42 | 9.88 |
| 4f1z | 2.46 | 2.06 | 27.50 | 1.73 | 2.58 | 51.63 | 9.03 | 29.38 | 40.61 | 33.78 | 36.62 | 39.19 | 3.40 | 40.51 | 28.99 | 9.42 | 29.59 | 26.63 | 40.10 | 28.08 |
| 4gq6 | 13.42 | 13.95 | 5.99 | 13.62 | 13.45 | 9.66 | 13.36 | 13.12 | 2.80 | 4.82 | 12.47 | 13.22 | 8.99 | 5.98 | 12.29 | 4.57 | 8.84 | 8.30 | 10.99 | 4.42 |
| 4gxl | 27.92 | 29.40 | 28.96 | 29.44 | 27.99 | 28.37 | 29.26 | 29.51 | 13.88 | 15.22 | 27.32 | 29.90 | 29.03 | 28.49 | 28.27 | 27.67 | 28.93 | 29.45 | 25.49 | 27.56 |
| 4gyw | 22.40 | 47.81 | 44.26 | 22.77 | 22.31 | 44.20 | 22.19 | 5.52 | 45.32 | 31.26 | 43.59 | 21.37 | 42.14 | 42.18 | 42.46 | 43.69 | 42.59 | 51.50 | 44.99 | 21.56 |
| 4h4f | 16.55 | 12.31 | 11.99 | 12.16 | 12.55 | 8.78 | 8.84 | 33.26 | 9.84 | 12.53 | 8.90 | 14.19 | 30.37 | 12.86 | 13.30 | 13.56 | 12.54 | 30.46 | 10.67 | 13.24 |
| 4hom | 15.00 | 11.29 | 13.31 | 42.84 | 42.82 | 15.77 | 42.65 | 11.14 | 8.90 | 11.43 | 23.82 | 33.12 | 33.49 | 14.89 | 36.10 | 8.68 | 43.23 | 42.62 | 22.11 | 32.67 |
| 4htp | 36.43 | 37.55 | 36.97 | 36.99 | 36.00 | 0.88 | 34.59 | 33.61 | 37.31 | 1.65 | 35.02 | 34.69 | 40.32 | 33.76 | 35.67 | 38.46 | 34.73 | 36.54 | 34.77 | 1.92 |
| 4iim | 26.92 | 24.93 | 24.20 | 24.45 | 11.83 | 20.28 | 20.23 | 19.87 | 25.23 | 24.06 | 25.03 | 26.67 | 19.45 | 24.18 | 26.40 | 1.11 | 11.95 | 25.44 | 21.27 | 21.51 |
| 4j8s | 2.31 | 1.96 | 1.28 | 2.41 | 1.72 | 8.88 | 3.33 | 11.44 | 12.52 | 30.73 | 11.08 | 27.78 | 9.29 | 31.27 | 11.57 | 1.99 | 4.84 | 12.57 | 30.76 | 12.10 |
| 4k0u | 1.21 | 2.44 | 1.94 | 2.44 | 1.47 | 1.77 | 3.74 | 24.53 | 26.13 | 7.90 | 5.18 | 22.67 | 5.18 | 6.71 | 6.70 | 16.25 | 22.42 | 23.33 | 28.65 | 13.68 |

**S5(d). I-RMSD values of all 20 poses obtained after blind docking by PatchDock on 133 protein-peptide complexes.**

| **ID** | **Pose1** | **Pose2** | **Pose3** | **Pose4** | **Pose5** | **Pose6** | **Pose7** | **Pose8** | **Pose9** | **Pose10** | **Pose11** | **Pose12** | **Pose13** | **Pose14** | **Pose15** | **Pose16** | **Pose17** | **Pose18** | **Pose19** | **Pose20** |
| --- | --- | --- | --- | --- | --- | --- | --- | --- | --- | --- | --- | --- | --- | --- | --- | --- | --- | --- | --- | --- |
| 1cjr | 2.29 | 18.23 | 6.09 | 6.15 | 12.36 | 7.03 | 16.62 | 8.71 | 18.08 | 16.17 | 9.82 | 9.14 | 15.77 | 10.01 | 12.10 | 9.26 | 16.25 | 11.47 | 12.32 | 13.60 |
| 1cka | 14.43 | 10.71 | 22.12 | 23.35 | 17.60 | 16.33 | 10.52 | 18.23 | 19.53 | 19.39 | 20.85 | 19.20 | 24.66 | 14.07 | 12.10 | 21.63 | 24.36 | 21.97 | 22.95 | 13.25 |
| 1cvu | 36.37 | 35.87 | 35.84 | 37.20 | 35.89 | 35.69 | 34.47 | 48.64 | 46.78 | 37.12 | 48.45 | 50.40 | 48.75 | 35.67 | 35.23 | 36.14 | 35.19 | 50.00 | 45.31 | 48.76 |
| 1d4t | 1.52 | 6.15 | 4.63 | 8.49 | 18.24 | 20.81 | 19.24 | 5.98 | 18.89 | 8.71 | 22.14 | 10.88 | 25.98 | 23.72 | 8.75 | 12.95 | 8.36 | 16.36 | 17.30 | 4.09 |
| 1eg4 | 43.37 | 40.63 | 43.97 | 31.28 | 47.02 | 37.34 | 26.83 | 29.29 | 28.16 | 38.91 | 39.77 | 25.92 | 37.76 | 38.92 | 41.20 | 47.47 | 33.71 | 44.27 | 36.14 | 39.76 |
| 1h6w | 0.98 | 36.48 | 17.22 | 18.33 | 17.65 | 8.94 | 17.53 | 3.57 | 11.78 | 5.09 | 11.09 | 4.48 | 4.46 | 21.59 | 11.96 | 11.05 | 17.50 | 19.99 | 8.63 | 18.87 |
| 1hc9 | 1.58 | 4.23 | 20.47 | 6.34 | 22.02 | 11.11 | 11.29 | 19.21 | 5.05 | 9.71 | 10.83 | 8.63 | 10.78 | 6.44 | 10.51 | 22.27 | 11.37 | 11.40 | 10.34 | 20.60 |
| 1jbu | 34.78 | 16.24 | 21.54 | 39.86 | 23.53 | 34.40 | 38.28 | 34.39 | 15.74 | 32.87 | 20.86 | 18.52 | 24.66 | 40.30 | 41.45 | 32.71 | 40.91 | 31.02 | 41.98 | 17.68 |
| 1k5n | 14.92 | 6.58 | 24.57 | 26.46 | 14.65 | 4.79 | 14.73 | 27.04 | 15.98 | 28.45 | 44.12 | 16.08 | 21.50 | 7.06 | 25.18 | 29.85 | 5.06 | 14.23 | 17.41 | 30.75 |
| 1mfg | 15.78 | 17.18 | 9.17 | 14.75 | 6.14 | 6.41 | 20.57 | 16.52 | 6.62 | 12.88 | 16.46 | 15.50 | 22.53 | 19.87 | 24.28 | 16.72 | 21.43 | 15.39 | 16.18 | 11.39 |
| 1nln | 15.68 | 20.32 | 31.24 | 20.65 | 30.17 | 30.65 | 25.59 | 24.91 | 35.72 | 30.56 | 28.84 | 24.27 | 33.71 | 28.04 | 29.80 | 23.82 | 32.67 | 32.21 | 26.46 | 31.51 |
| 1nq7 | 35.88 | 21.04 | 35.25 | 21.18 | 35.01 | 21.52 | 35.81 | 22.28 | 21.53 | 35.61 | 20.81 | 37.32 | 35.29 | 35.62 | 34.06 | 21.20 | 35.64 | 20.71 | 34.41 | 19.16 |
| 1ntv | 27.61 | 1.78 | 32.19 | 29.29 | 28.07 | 13.82 | 27.94 | 32.91 | 26.88 | 25.09 | 28.92 | 30.23 | 28.73 | 25.31 | 25.81 | 28.28 | 16.19 | 19.40 | 27.47 | 32.32 |
| 1nx1 | 27.29 | 32.65 | 34.63 | 28.74 | 35.22 | 25.78 | 31.06 | 34.47 | 27.97 | 32.29 | 30.97 | 28.59 | 33.38 | 33.33 | 5.04 | 32.84 | 25.21 | 33.67 | 26.71 | 35.29 |
| 1oai | 17.31 | 21.71 | 20.96 | 18.32 | 17.50 | 14.06 | 24.55 | 23.76 | 22.62 | 10.53 | 22.98 | 21.12 | 25.09 | 10.92 | 15.39 | 21.41 | 10.59 | 24.08 | 24.89 | 10.76 |
| 1oj5 | 5.16 | 23.31 | 10.45 | 16.16 | 21.33 | 26.02 | 13.43 | 20.92 | 24.96 | 27.34 | 23.30 | 13.54 | 25.79 | 4.53 | 15.60 | 23.87 | 16.58 | 16.41 | 29.04 | 11.74 |
| 1ou8 | 13.13 | 11.29 | 9.16 | 29.90 | 0.99 | 7.11 | 30.77 | 26.46 | 11.54 | 33.76 | 23.51 | 31.07 | 29.59 | 23.76 | 30.10 | 24.42 | 27.66 | 30.75 | 10.07 | 30.87 |
| 1ow6 | 15.98 | 16.58 | 17.86 | 19.84 | 18.60 | 17.91 | 16.00 | 15.76 | 13.42 | 11.37 | 15.24 | 13.15 | 18.30 | 19.45 | 15.67 | 14.68 | 13.38 | 14.51 | 25.64 | 13.90 |
| 1pzl | 21.64 | 20.49 | 25.95 | 22.81 | 21.48 | 23.54 | 30.80 | 22.26 | 27.34 | 26.25 | 29.99 | 22.71 | 5.58 | 18.71 | 29.90 | 28.57 | 21.77 | 24.34 | 23.03 | 43.22 |
| 1qkz | 33.43 | 32.24 | 32.55 | 68.20 | 33.67 | 34.89 | 32.64 | 32.54 | 31.65 | 61.42 | 69.65 | 35.40 | 32.03 | 33.90 | 33.89 | 34.56 | 31.29 | 32.12 | 34.42 | 33.67 |
| 1rst | 8.35 | 8.07 | 8.97 | 6.76 | 7.10 | 8.03 | 8.08 | 3.70 | 35.35 | 10.03 | 8.53 | 6.00 | 7.98 | 7.17 | 5.72 | 7.95 | 5.04 | 35.51 | 7.21 | 5.82 |
| 1rxz | 19.43 | 28.39 | 23.17 | 14.54 | 22.08 | 4.66 | 19.36 | 31.60 | 23.46 | 21.95 | 31.80 | 25.39 | 24.92 | 26.26 | 25.48 | 26.74 | 6.53 | 23.44 | 1.60 | 21.44 |
| 1sfi | 2.52 | 17.12 | 12.56 | 12.26 | 5.73 | 34.61 | 14.31 | 15.06 | 6.54 | 11.40 | 13.97 | 13.30 | 23.07 | 14.85 | 6.78 | 32.41 | 10.39 | 4.25 | 11.64 | 10.33 |
| 1ssh | 19.70 | 8.60 | 25.82 | 11.62 | 17.79 | 25.92 | 23.45 | 18.46 | 28.05 | 16.61 | 13.02 | 12.92 | 18.29 | 20.10 | 11.50 | 25.91 | 8.91 | 15.72 | 17.82 | 12.26 |
| 1t08 | 31.86 | 24.65 | 11.79 | 20.76 | 23.26 | 29.11 | 31.21 | 18.97 | 13.16 | 30.02 | 24.02 | 13.24 | 16.06 | 38.32 | 51.91 | 25.54 | 31.33 | 10.07 | 23.73 | 25.75 |
| 1t4f | 8.23 | 7.75 | 3.07 | 9.31 | 9.67 | 15.90 | 4.39 | 18.83 | 5.82 | 8.13 | 9.14 | 5.66 | 7.69 | 11.30 | 10.53 | 16.90 | 9.10 | 16.00 | 8.94 | 9.33 |
| 1t7r | 27.16 | 38.75 | 35.52 | 34.34 | 27.09 | 38.65 | 8.98 | 25.54 | 15.71 | 29.51 | 15.15 | 39.26 | 18.48 | 1.20 | 28.42 | 37.50 | 35.54 | 28.31 | 19.47 | 35.55 |
| 1tfc | 24.02 | 23.68 | 23.21 | 10.82 | 24.47 | 27.31 | 24.15 | 27.81 | 29.80 | 27.13 | 24.23 | 25.96 | 27.08 | 26.27 | 26.56 | 24.11 | 11.33 | 24.52 | 31.08 | 23.84 |
| 1u00 | 28.10 | 29.81 | 17.16 | 14.46 | 16.23 | 14.73 | 21.70 | 15.32 | 33.17 | 18.59 | 23.32 | 14.00 | 31.75 | 13.66 | 27.88 | 15.12 | 16.52 | 6.04 | 25.89 | 29.46 |
| 1uj0 | 21.98 | 17.88 | 20.06 | 14.29 | 9.89 | 12.94 | 27.79 | 21.82 | 17.23 | 14.96 | 22.03 | 20.75 | 14.76 | 22.74 | 27.27 | 16.35 | 13.14 | 14.68 | 11.07 | 12.17 |
| 1x2r | 1.73 | 5.54 | 30.95 | 8.54 | 6.51 | 31.12 | 7.68 | 9.58 | 31.13 | 7.49 | 32.84 | 6.97 | 30.84 | 7.09 | 32.84 | 29.82 | 6.91 | 5.44 | 8.18 | 31.26 |
| 1xoc | 0.92 | 13.99 | 19.22 | 5.97 | 27.47 | 26.06 | 23.34 | 23.89 | 20.76 | 23.61 | 24.61 | 14.09 | 4.01 | 23.27 | 22.17 | 29.01 | 23.54 | 28.89 | 22.17 | 22.88 |
| 1ymt | 29.04 | 30.43 | 4.94 | 9.14 | 36.02 | 22.35 | 32.37 | 22.42 | 33.33 | 34.08 | 9.33 | 27.08 | 34.75 | 33.53 | 6.22 | 37.45 | 37.76 | 29.12 | 34.09 | 34.40 |
| 1yuc | 21.36 | 20.55 | 19.83 | 29.23 | 17.48 | 14.36 | 30.18 | 28.97 | 23.81 | 32.69 | 19.05 | 7.76 | 29.25 | 17.93 | 32.13 | 21.53 | 22.88 | 18.33 | 19.72 | 20.72 |
| 1ywo | 16.86 | 22.03 | 5.75 | 16.05 | 16.36 | 6.75 | 16.06 | 11.61 | 26.90 | 27.34 | 18.87 | 13.78 | 19.02 | 22.63 | 8.45 | 12.15 | 27.43 | 11.21 | 16.66 | 14.99 |
| 2a25 | 0.46 | 16.36 | 20.24 | 18.98 | 21.78 | 8.73 | 19.43 | 12.21 | 8.65 | 17.14 | 18.40 | 23.07 | 23.24 | 16.83 | 19.20 | 19.36 | 15.59 | 17.04 | 18.97 | 21.88 |
| 2a3i | 28.74 | 8.26 | 32.54 | 13.48 | 17.72 | 38.66 | 24.91 | 7.31 | 28.59 | 34.21 | 34.55 | 8.43 | 7.71 | 8.83 | 7.75 | 29.15 | 5.75 | 36.51 | 36.25 | 31.83 |
| 2aq9 | 20.41 | 31.94 | 29.04 | 20.08 | 26.13 | 31.72 | 29.67 | 34.17 | 35.25 | 28.31 | 31.92 | 20.40 | 34.99 | 19.06 | 35.21 | 35.10 | 37.67 | 34.10 | 19.12 | 12.68 |
| 2b9h | 33.56 | 31.30 | 21.34 | 21.70 | 31.13 | 28.60 | 36.69 | 28.43 | 31.58 | 20.57 | 25.96 | 36.74 | 24.88 | 18.90 | 42.15 | 41.79 | 19.73 | 46.04 | 43.68 | 34.30 |
| 2bba | 17.55 | 1.57 | 12.57 | 13.62 | 13.20 | 28.57 | 30.55 | 11.06 | 14.17 | 14.94 | 27.60 | 5.53 | 13.62 | 15.04 | 28.25 | 6.31 | 15.11 | 15.70 | 28.71 | 12.96 |
| 2cch | 30.68 | 35.87 | 27.56 | 31.81 | 30.60 | 25.93 | 25.50 | 33.96 | 21.91 | 28.03 | 25.97 | 39.68 | 29.46 | 31.49 | 27.02 | 25.17 | 42.80 | 33.62 | 28.68 | 24.52 |
| 2ce8 | 28.44 | 27.73 | 27.56 | 33.70 | 25.78 | 27.45 | 32.84 | 27.55 | 32.63 | 32.37 | 27.87 | 34.07 | 24.48 | 28.75 | 31.19 | 28.29 | 34.22 | 32.44 | 26.73 | 34.67 |
| 2d0n | 18.66 | 18.49 | 22.98 | 20.16 | 14.37 | 19.27 | 17.23 | 21.10 | 24.76 | 17.85 | 24.59 | 20.65 | 16.25 | 22.60 | 20.96 | 10.29 | 15.47 | 18.01 | 19.21 | 22.63 |
| 2drk | 22.72 | 18.31 | 27.62 | 11.83 | 15.94 | 12.37 | 19.12 | 25.98 | 25.62 | 19.50 | 15.87 | 20.73 | 23.26 | 19.53 | 18.35 | 12.23 | 26.19 | 16.56 | 21.94 | 21.34 |
| 2dyp | 19.61 | 14.46 | 30.38 | 15.26 | 14.88 | 19.49 | 30.33 | 3.06 | 7.64 | 10.66 | 29.25 | 23.11 | 16.63 | 16.77 | 6.28 | 23.00 | 21.95 | 34.26 | 27.65 | 29.30 |
| 2fff | 20.98 | 24.93 | 25.69 | 24.29 | 25.08 | 26.40 | 26.01 | 22.52 | 23.91 | 23.79 | 24.12 | 25.06 | 26.12 | 28.27 | 42.75 | 22.54 | 41.72 | 26.17 | 23.55 | 54.78 |
| 2ffu | 15.44 | 29.69 | 6.59 | 14.65 | 10.77 | 11.52 | 14.79 | 9.92 | 32.85 | 26.81 | 32.15 | 19.15 | 34.28 | 47.16 | 32.45 | 21.15 | 36.84 | 15.13 | 50.45 | 33.56 |
| 2fka | 25.06 | 5.17 | 8.62 | 10.00 | 29.25 | 6.47 | 8.33 | 24.11 | 7.61 | 6.94 | 27.37 | 7.99 | 9.76 | 7.54 | 8.39 | 28.94 | 28.04 | 9.77 | 7.50 | 5.33 |
| 2fmf | 11.11 | 7.75 | 12.50 | 29.31 | 19.73 | 20.77 | 6.68 | 6.26 | 29.58 | 11.73 | 25.23 | 27.56 | 12.56 | 31.35 | 22.55 | 24.64 | 10.78 | 13.16 | 13.00 | 20.23 |
| 2fts | 24.74 | 1.33 | 34.69 | 28.17 | 28.01 | 31.27 | 18.02 | 27.65 | 26.55 | 29.26 | 26.02 | 31.31 | 26.78 | 24.39 | 58.49 | 31.43 | 23.57 | 28.37 | 14.22 | 22.87 |
| 2fvj | 27.14 | 29.27 | 27.49 | 26.06 | 22.19 | 26.84 | 25.97 | 8.49 | 28.08 | 26.98 | 23.02 | 5.79 | 28.24 | 10.58 | 18.83 | 37.66 | 16.46 | 29.88 | 37.53 | 25.00 |
| 2ho2 | 24.74 | 20.17 | 24.45 | 21.99 | 18.47 | 20.69 | 16.81 | 27.20 | 22.13 | 11.47 | 14.41 | 18.77 | 19.83 | 18.89 | 16.99 | 11.34 | 17.35 | 14.51 | 21.29 | 10.77 |
| 2ht9 | 27.21 | 23.56 | 20.60 | 23.05 | 28.98 | 23.65 | 29.98 | 18.62 | 29.23 | 22.32 | 31.71 | 22.33 | 10.62 | 23.39 | 28.16 | 27.48 | 9.36 | 22.65 | 28.32 | 21.22 |
| 2o02 | 0.98 | 14.76 | 7.29 | 9.60 | 4.11 | 14.41 | 4.63 | 5.61 | 15.43 | 5.57 | 6.53 | 12.36 | 14.83 | 18.23 | 26.71 | 13.03 | 14.64 | 18.55 | 13.76 | 6.08 |
| 2o4j | 20.21 | 19.76 | 19.49 | 20.37 | 20.09 | 34.14 | 9.28 | 17.99 | 8.43 | 24.70 | 34.37 | 35.34 | 17.79 | 20.55 | 32.66 | 22.26 | 29.50 | 19.91 | 37.69 | 34.07 |
| 2o9v | 30.96 | 21.07 | 27.87 | 28.17 | 18.41 | 28.50 | 16.70 | 22.42 | 27.27 | 23.74 | 17.42 | 23.42 | 19.65 | 23.90 | 25.66 | 28.10 | 18.11 | 20.33 | 27.08 | 21.47 |
| 2oei | 21.54 | 19.55 | 17.47 | 24.31 | 20.87 | 10.30 | 23.07 | 24.15 | 25.24 | 15.67 | 26.14 | 21.06 | 20.19 | 25.68 | 19.89 | 19.33 | 12.86 | 18.04 | 13.48 | 18.66 |
| 2p0w | 17.80 | 15.01 | 24.83 | 25.75 | 17.92 | 19.93 | 14.73 | 7.96 | 13.02 | 33.67 | 21.07 | 16.25 | 14.04 | 12.87 | 18.34 | 31.06 | 34.51 | 15.52 | 32.48 | 22.64 |
| 2p1o | 39.18 | 11.57 | 7.28 | 18.71 | 35.59 | 36.31 | 31.77 | 9.49 | 11.49 | 29.98 | 12.02 | 12.77 | 3.11 | 13.24 | 35.89 | 8.46 | 9.95 | 22.98 | 13.57 | 16.69 |
| 2p1t | 20.05 | 22.09 | 20.70 | 23.08 | 23.07 | 10.08 | 21.32 | 21.64 | 18.90 | 19.09 | 27.75 | 22.42 | 8.75 | 18.78 | 12.64 | 30.01 | 20.42 | 22.06 | 22.49 | 22.85 |
| 2p54 | 22.10 | 26.13 | 1.83 | 23.90 | 24.92 | 23.16 | 24.12 | 25.38 | 31.95 | 25.69 | 21.83 | 29.97 | 42.20 | 21.94 | 22.54 | 21.15 | 37.51 | 21.26 | 11.56 | 23.55 |
| 2peh | 25.50 | 26.38 | 23.26 | 24.57 | 27.95 | 30.77 | 25.21 | 31.78 | 30.07 | 25.32 | 30.92 | 22.26 | 24.98 | 24.37 | 8.42 | 25.28 | 25.64 | 25.61 | 28.89 | 26.40 |
| 2pux | 44.32 | 33.58 | 21.91 | 20.21 | 28.75 | 29.66 | 25.72 | 29.62 | 24.87 | 21.53 | 14.43 | 33.50 | 27.95 | 28.43 | 24.91 | 28.05 | 31.25 | 39.46 | 39.80 | 30.19 |
| 2puy | 24.62 | 21.37 | 21.52 | 7.47 | 26.77 | 21.01 | 12.25 | 13.61 | 24.83 | 9.24 | 16.80 | 26.93 | 23.87 | 15.03 | 16.16 | 20.15 | 20.30 | 22.00 | 20.29 | 24.42 |
| 2qbx | 1.48 | 6.43 | 35.02 | 16.01 | 17.30 | 17.54 | 28.61 | 30.08 | 8.28 | 21.14 | 14.79 | 3.34 | 15.01 | 19.61 | 36.25 | 10.81 | 17.40 | 16.08 | 31.29 | 31.29 |
| 2qos | 9.84 | 9.16 | 5.53 | 6.99 | 1.41 | 10.38 | 4.12 | 8.34 | 6.97 | 8.49 | 10.44 | 5.92 | 9.35 | 3.88 | 5.10 | 7.03 | 9.32 | 11.24 | 9.47 | 11.42 |
| 2qse | 3.93 | 10.14 | 32.18 | 9.67 | 30.16 | 31.26 | 9.91 | 17.54 | 25.21 | 24.42 | 24.48 | 10.28 | 23.86 | 30.68 | 27.54 | 44.03 | 27.70 | 27.53 | 34.94 | 25.70 |
| 2r7g | 4.72 | 9.12 | 4.67 | 11.06 | 10.14 | 5.94 | 2.81 | 10.81 | 9.09 | 10.17 | 6.70 | 5.24 | 4.23 | 11.01 | 5.06 | 7.21 | 31.38 | 9.92 | 30.12 | 10.12 |
| 2r9q | 9.06 | 15.00 | 11.82 | 26.62 | 27.11 | 13.93 | 29.06 | 2.22 | 14.08 | 6.30 | 25.91 | 13.58 | 23.10 | 12.97 | 11.17 | 19.17 | 8.10 | 26.49 | 28.21 | 9.57 |
| 2v8y | 37.13 | 36.49 | 1.11 | 36.85 | 34.36 | 38.45 | 35.01 | 13.42 | 36.49 | 36.50 | 39.32 | 38.61 | 38.43 | 27.26 | 25.81 | 35.27 | 23.36 | 38.99 | 36.46 | 32.16 |
| 2vkn | 25.61 | 23.04 | 23.57 | 21.49 | 20.80 | 24.11 | 26.59 | 11.18 | 23.94 | 21.68 | 25.93 | 27.62 | 28.64 | 22.20 | 30.29 | 21.74 | 24.85 | 28.50 | 25.89 | 19.75 |
| 2vr3 | 0.82 | 23.30 | 26.38 | 27.06 | 27.05 | 27.75 | 26.95 | 23.42 | 33.10 | 18.84 | 23.02 | 26.30 | 10.46 | 17.83 | 14.68 | 23.51 | 12.82 | 17.34 | 30.45 | 22.40 |
| 2vwf | 14.67 | 26.70 | 19.11 | 24.46 | 16.69 | 15.94 | 14.84 | 18.51 | 25.16 | 26.55 | 26.97 | 24.80 | 20.70 | 28.93 | 17.86 | 18.00 | 26.21 | 10.64 | 23.66 | 26.92 |
| 2w2u | 17.87 | 15.45 | 14.93 | 16.97 | 6.22 | 23.09 | 8.11 | 7.21 | 16.63 | 22.14 | 21.96 | 9.71 | 15.26 | 8.03 | 21.73 | 8.80 | 25.66 | 9.45 | 16.35 | 7.01 |
| 2whx | 24.07 | 27.69 | 31.17 | 63.02 | 29.13 | 36.88 | 62.42 | 24.07 | 37.17 | 39.62 | 22.41 | 34.20 | 62.76 | 31.36 | 24.23 | 64.94 | 69.72 | 61.90 | 33.20 | 56.02 |
| 2xrw | 35.17 | 36.27 | 23.32 | 28.15 | 31.48 | 34.58 | 19.51 | 26.84 | 37.34 | 36.14 | 29.34 | 35.70 | 38.75 | 29.37 | 39.94 | 38.45 | 25.86 | 22.10 | 29.18 | 36.18 |
| 2xu7 | 30.07 | 27.60 | 20.39 | 14.02 | 37.98 | 27.15 | 10.10 | 27.89 | 32.69 | 20.27 | 34.97 | 29.54 | 32.34 | 18.46 | 12.75 | 5.90 | 34.35 | 39.67 | 13.24 | 12.31 |
| 2xvc | 22.52 | 20.94 | 21.97 | 12.46 | 22.90 | 21.68 | 21.10 | 2.07 | 7.67 | 10.48 | 3.82 | 24.04 | 21.27 | 22.65 | 21.72 | 21.07 | 8.93 | 21.57 | 19.08 | 22.26 |
| 2zjd | 18.88 | 20.12 | 21.34 | 22.93 | 18.61 | 23.21 | 20.39 | 21.65 | 22.07 | 21.26 | 20.99 | 21.67 | 23.97 | 22.84 | 2.28 | 24.02 | 23.27 | 24.56 | 22.48 | 15.23 |
| 3asl | 18.84 | 19.42 | 18.51 | 20.52 | 17.40 | 8.39 | 17.90 | 21.37 | 21.85 | 10.24 | 18.34 | 17.63 | 17.08 | 19.47 | 12.52 | 16.53 | 16.42 | 22.15 | 9.41 | 15.78 |
| 3awr | 15.10 | 14.54 | 12.14 | 15.49 | 14.45 | 11.99 | 13.45 | 15.12 | 18.82 | 17.82 | 12.37 | 12.31 | 12.97 | 11.78 | 12.30 | 13.55 | 22.67 | 20.66 | 10.92 | 20.85 |
| 3ayu | 15.97 | 0.85 | 19.24 | 18.11 | 26.93 | 20.17 | 15.72 | 27.57 | 6.09 | 15.77 | 16.02 | 3.76 | 25.13 | 24.94 | 27.97 | 5.75 | 25.73 | 16.23 | 25.88 | 25.66 |
| 3bfq | 1.12 | 28.13 | 27.57 | 29.18 | 9.79 | 27.96 | 3.97 | 16.30 | 27.68 | 27.57 | 29.95 | 28.34 | 7.59 | 34.37 | 27.72 | 29.08 | 28.37 | 31.49 | 29.42 | 10.57 |
| 3c3r | 22.52 | 25.06 | 22.55 | 23.86 | 24.29 | 23.31 | 23.98 | 24.91 | 41.16 | 21.79 | 17.98 | 23.00 | 28.43 | 35.26 | 23.60 | 19.04 | 24.97 | 22.40 | 21.22 | 23.01 |
| 3d32 | 0.83 | 21.62 | 11.77 | 10.70 | 25.18 | 29.23 | 15.87 | 20.44 | 23.43 | 24.73 | 30.14 | 26.42 | 22.37 | 19.23 | 23.44 | 20.31 | 29.74 | 22.60 | 28.40 | 22.57 |
| 3ds4 | 1.17 | 7.65 | 10.43 | 7.92 | 9.62 | 6.08 | 10.70 | 12.68 | 11.97 | 7.77 | 11.48 | 12.40 | 8.40 | 9.18 | 10.95 | 6.03 | 11.67 | 10.44 | 9.39 | 11.19 |
| 3ery | 3.07 | 15.29 | 9.86 | 7.89 | 27.05 | 4.02 | 5.85 | 7.47 | 15.93 | 19.95 | 14.43 | 14.76 | 8.84 | 12.62 | 29.36 | 28.62 | 26.82 | 19.31 | 20.59 | 15.72 |
| 3fdo | 12.48 | 12.17 | 18.93 | 10.74 | 5.94 | 12.39 | 3.69 | 12.63 | 17.71 | 18.35 | 11.45 | 7.92 | 4.67 | 20.88 | 1.50 | 11.30 | 11.74 | 6.45 | 17.99 | 11.90 |
| 3g2s | 35.06 | 36.20 | 33.60 | 25.58 | 6.95 | 8.19 | 18.46 | 12.98 | 32.47 | 35.79 | 29.12 | 34.37 | 19.02 | 35.91 | 20.56 | 24.79 | 14.38 | 40.11 | 21.68 | 26.52 |
| 3gyt | 28.00 | 29.49 | 27.82 | 19.48 | 29.55 | 7.72 | 18.64 | 28.84 | 18.12 | 29.29 | 29.45 | 27.78 | 27.71 | 29.83 | 8.30 | 26.52 | 21.02 | 22.13 | 22.28 | 22.95 |
| 3h1z | 40.19 | 32.93 | 33.23 | 40.85 | 53.65 | 42.17 | 30.88 | 10.99 | 28.48 | 54.28 | 23.36 | 33.64 | 26.80 | 26.66 | 25.20 | 40.56 | 24.57 | 30.64 | 32.86 | 34.75 |
| 3i5r | 10.54 | 16.68 | 22.58 | 11.22 | 4.22 | 13.13 | 19.63 | 26.73 | 8.39 | 23.37 | 20.99 | 17.34 | 16.90 | 19.07 | 20.13 | 4.64 | 28.70 | 11.10 | 9.03 | 20.23 |
| 3ivv | 26.42 | 27.72 | 4.30 | 26.21 | 30.58 | 24.51 | 26.35 | 33.41 | 25.74 | 14.34 | 32.03 | 20.63 | 27.02 | 25.73 | 21.85 | 13.60 | 27.18 | 21.32 | 26.87 | 21.16 |
| 3kmr | 22.30 | 21.77 | 21.76 | 35.96 | 22.68 | 22.26 | 24.20 | 22.12 | 35.09 | 25.39 | 21.78 | 33.46 | 23.32 | 32.62 | 21.88 | 23.13 | 21.09 | 20.98 | 25.00 | 22.09 |
| 3kuj | 22.60 | 19.40 | 8.64 | 26.18 | 26.52 | 21.57 | 19.10 | 22.00 | 22.25 | 21.46 | 24.03 | 22.09 | 22.15 | 21.74 | 20.81 | 21.85 | 21.17 | 21.53 | 23.73 | 21.23 |
| 3kus | 23.99 | 22.37 | 17.36 | 22.75 | 15.96 | 16.29 | 8.95 | 15.47 | 8.16 | 20.68 | 19.40 | 13.16 | 19.65 | 20.60 | 15.54 | 17.77 | 17.68 | 18.70 | 9.27 | 4.53 |
| 3l0e | 32.05 | 20.60 | 34.86 | 21.62 | 7.42 | 33.37 | 30.10 | 12.52 | 18.19 | 33.68 | 20.49 | 31.59 | 34.29 | 35.17 | 38.49 | 33.47 | 33.02 | 12.25 | 21.48 | 11.86 |
| 3ll8 | 40.90 | 25.48 | 29.79 | 38.11 | 39.25 | 33.17 | 25.72 | 0.62 | 44.30 | 34.25 | 25.81 | 34.15 | 34.86 | 37.11 | 33.11 | 20.74 | 30.71 | 37.03 | 32.42 | 33.47 |
| 3llz | 29.13 | 24.37 | 19.09 | 17.88 | 24.65 | 24.19 | 29.69 | 21.19 | 20.48 | 18.97 | 24.36 | 20.77 | 24.96 | 20.04 | 20.85 | 30.02 | 27.68 | 22.79 | 20.71 | 20.86 |
| 3obq | 7.51 | 30.72 | 28.68 | 16.67 | 18.83 | 27.76 | 3.98 | 34.74 | 26.77 | 22.76 | 27.94 | 19.47 | 32.31 | 25.88 | 23.15 | 24.57 | 27.49 | 19.14 | 23.26 | 32.88 |
| 3olf | 35.04 | 34.81 | 32.04 | 36.86 | 32.95 | 20.45 | 21.76 | 29.75 | 32.79 | 4.12 | 40.98 | 37.18 | 20.55 | 29.72 | 35.64 | 31.87 | 41.80 | 40.35 | 36.06 | 41.76 |
| 3p72 | 5.57 | 7.27 | 8.10 | 8.02 | 7.53 | 10.16 | 9.27 | 7.20 | 9.75 | 9.12 | 9.43 | 9.53 | 9.37 | 8.93 | 10.57 | 6.87 | 10.36 | 0.95 | 8.65 | 6.99 |
| 3p8f | 1.77 | 12.77 | 12.54 | 2.71 | 15.78 | 8.02 | 13.12 | 13.56 | 12.07 | 5.36 | 11.03 | 11.18 | 9.54 | 10.19 | 11.43 | 12.15 | 4.33 | 13.84 | 13.84 | 13.70 |
| 3ptl | 12.18 | 17.19 | 23.50 | 28.84 | 4.54 | 26.32 | 12.47 | 13.64 | 29.64 | 27.58 | 33.48 | 17.77 | 9.11 | 30.83 | 28.60 | 33.29 | 30.33 | 21.83 | 34.28 | 37.72 |
| 3qis | 35.04 | 44.01 | 29.15 | 31.52 | 34.25 | 31.86 | 31.97 | 40.48 | 34.36 | 35.36 | 44.32 | 30.86 | 39.72 | 13.38 | 30.33 | 15.38 | 13.80 | 32.02 | 9.98 | 38.84 |
| 3rm1 | 17.14 | 16.72 | 6.90 | 3.48 | 9.09 | 19.82 | 10.26 | 17.67 | 10.72 | 19.27 | 4.21 | 9.57 | 5.25 | 11.11 | 20.88 | 19.74 | 16.47 | 17.67 | 3.99 | 5.09 |
| 3rqg | 27.43 | 30.16 | 29.69 | 35.00 | 5.10 | 11.28 | 7.65 | 5.86 | 32.27 | 27.98 | 28.71 | 30.08 | 7.36 | 11.84 | 2.00 | 31.34 | 5.55 | 12.93 | 26.02 | 35.23 |
| 3sfj | 15.46 | 21.41 | 14.14 | 10.71 | 25.79 | 24.18 | 15.46 | 1.65 | 15.46 | 19.37 | 22.27 | 15.69 | 16.35 | 15.75 | 8.58 | 7.05 | 27.25 | 15.25 | 28.61 | 23.37 |
| 3so6 | 0.99 | 22.39 | 9.73 | 19.93 | 3.89 | 22.52 | 30.24 | 19.50 | 32.47 | 31.71 | 30.79 | 3.80 | 30.21 | 31.61 | 26.10 | 31.46 | 34.73 | 33.08 | 19.85 | 34.49 |
| 3tjv | 2.90 | 8.20 | 16.54 | 18.23 | 16.38 | 15.80 | 25.66 | 3.86 | 32.80 | 32.97 | 28.09 | 15.09 | 18.52 | 17.07 | 3.04 | 29.95 | 15.60 | 22.27 | 26.93 | 24.85 |
| 3tzy | 21.75 | 21.12 | 19.87 | 18.85 | 23.65 | 19.18 | 22.46 | 20.39 | 17.09 | 16.69 | 19.61 | 25.68 | 20.26 | 17.71 | 20.02 | 18.81 | 21.69 | 23.94 | 23.38 | 21.39 |
| 3u9q | 26.94 | 20.60 | 8.24 | 25.10 | 3.15 | 34.93 | 7.87 | 24.81 | 25.44 | 30.63 | 4.41 | 34.41 | 28.59 | 23.70 | 21.64 | 25.47 | 27.12 | 21.43 | 25.61 | 21.27 |
| 3up3 | 30.58 | 22.22 | 24.54 | 7.39 | 15.16 | 10.27 | 16.44 | 20.01 | 36.67 | 21.80 | 32.60 | 2.71 | 35.62 | 35.67 | 26.18 | 18.30 | 13.21 | 6.10 | 22.41 | 22.98 |
| 3v2x | 36.70 | 15.40 | 14.39 | 16.08 | 30.34 | 12.07 | 27.10 | 27.92 | 24.88 | 32.93 | 36.75 | 30.70 | 8.09 | 19.84 | 29.77 | 23.13 | 5.95 | 30.21 | 32.59 | 28.23 |
| 3vtc | 20.25 | 20.56 | 19.29 | 19.62 | 11.85 | 17.14 | 12.22 | 31.90 | 32.31 | 15.74 | 18.56 | 9.18 | 19.11 | 15.30 | 25.81 | 1.35 | 34.97 | 34.13 | 37.00 | 13.75 |
| 3w1b | 65.47 | 69.25 | 67.90 | 69.20 | 66.04 | 66.76 | 64.82 | 65.25 | 67.44 | 71.82 | 69.44 | 45.15 | 70.68 | 74.74 | 66.75 | 68.05 | 69.10 | 66.59 | 82.35 | 45.58 |
| 3zqh | 29.02 | 30.79 | 17.12 | 35.83 | 32.44 | 18.71 | 26.39 | 26.84 | 26.23 | 34.51 | 25.52 | 26.88 | 23.60 | 33.18 | 9.73 | 35.45 | 17.12 | 20.20 | 30.17 | 38.77 |
| 4b4n | 11.57 | 32.29 | 31.41 | 10.06 | 13.60 | 10.47 | 9.54 | 32.45 | 29.85 | 30.89 | 32.68 | 32.88 | 35.41 | 32.49 | 25.64 | 9.23 | 34.07 | 32.10 | 32.90 | 10.32 |
| 4dcb | 10.88 | 9.87 | 10.99 | 8.41 | 11.18 | 12.20 | 7.78 | 10.86 | 5.17 | 9.47 | 8.63 | 9.49 | 10.74 | 6.60 | 8.08 | 60.98 | 10.82 | 1.43 | 61.39 | 25.04 |
| 4e34 | 14.34 | 16.93 | 1.94 | 17.95 | 17.11 | 16.69 | 15.19 | 18.08 | 11.53 | 15.61 | 28.39 | 16.18 | 20.92 | 26.86 | 17.12 | 15.62 | 14.03 | 26.28 | 23.80 | 15.05 |
| 4eik | 16.54 | 18.28 | 21.97 | 23.58 | 22.07 | 25.34 | 19.18 | 24.53 | 19.94 | 24.31 | 24.34 | 3.04 | 23.29 | 22.43 | 20.62 | 13.32 | 9.84 | 13.24 | 15.85 | 23.00 |
| 4ery | 28.78 | 32.36 | 31.61 | 33.37 | 10.41 | 31.95 | 31.90 | 31.62 | 8.51 | 35.78 | 37.05 | 32.81 | 6.69 | 11.72 | 31.69 | 33.39 | 6.29 | 31.31 | 30.52 | 33.24 |
| 4f14 | 15.46 | 20.08 | 20.54 | 18.08 | 25.48 | 20.05 | 20.93 | 20.48 | 21.77 | 27.36 | 13.26 | 18.41 | 18.69 | 23.25 | 15.87 | 23.37 | 20.51 | 12.48 | 19.52 | 20.77 |
| 4f1z | 3.00 | 2.62 | 26.20 | 28.83 | 16.62 | 24.84 | 27.38 | 13.65 | 27.08 | 30.43 | 20.52 | 33.21 | 4.38 | 25.29 | 30.32 | 32.90 | 9.03 | 26.87 | 28.92 | 33.56 |
| 4gq6 | 5.07 | 11.78 | 7.72 | 8.72 | 11.47 | 13.49 | 7.93 | 8.93 | 7.62 | 10.11 | 17.17 | 11.76 | 7.87 | 10.33 | 9.31 | 11.18 | 7.74 | 8.92 | 16.21 | 9.27 |
| 4gxl | 28.49 | 30.58 | 26.11 | 25.31 | 34.98 | 31.69 | 30.51 | 28.97 | 31.32 | 27.96 | 30.48 | 31.46 | 23.35 | 28.80 | 24.49 | 26.68 | 26.31 | 29.04 | 30.18 | 25.18 |
| 4gyw | 39.16 | 38.95 | 29.40 | 39.54 | 39.77 | 38.63 | 10.86 | 40.09 | 37.59 | 36.01 | 22.76 | 42.07 | 25.89 | 27.42 | 24.49 | 6.65 | 31.14 | 40.00 | 25.28 | 21.35 |
| 4h4f | 31.13 | 29.74 | 28.94 | 29.18 | 35.92 | 32.36 | 29.10 | 28.25 | 29.53 | 32.86 | 35.05 | 32.87 | 34.82 | 31.67 | 27.43 | 34.02 | 33.78 | 31.97 | 33.30 | 29.06 |
| 4hom | 1.41 | 18.11 | 29.78 | 30.94 | 26.36 | 21.62 | 24.79 | 22.53 | 20.31 | 24.19 | 18.21 | 29.65 | 14.39 | 27.73 | 24.45 | 24.71 | 21.28 | 22.05 | 28.92 | 22.42 |
| 4htp | 22.36 | 24.19 | 23.26 | 24.01 | 15.39 | 21.65 | 14.27 | 22.38 | 23.38 | 26.90 | 23.69 | 20.45 | 30.31 | 34.09 | 43.31 | 21.81 | 23.74 | 21.39 | 21.98 | 29.35 |
| 4iim | 13.51 | 28.48 | 23.61 | 16.40 | 27.20 | 13.25 | 0.77 | 19.97 | 7.33 | 12.94 | 22.80 | 16.90 | 18.72 | 24.24 | 21.89 | 10.72 | 16.37 | 19.99 | 20.14 | 20.03 |
| 4j8s | 8.87 | 11.93 | 32.37 | 12.23 | 17.67 | 29.10 | 12.59 | 30.18 | 32.09 | 14.30 | 11.10 | 19.40 | 12.97 | 12.22 | 10.26 | 17.83 | 31.99 | 11.51 | 19.40 | 13.55 |
| 4k0u | 16.24 | 1.78 | 13.76 | 4.52 | 6.73 | 14.52 | 19.14 | 18.18 | 14.50 | 5.64 | 10.65 | 16.07 | 13.89 | 8.03 | 10.01 | 5.96 | 17.16 | 14.17 | 4.41 | 12.88 |

**S5(e) I-RMSD values of all 20 poses obtained after blind docking by pepATTRACT on 133 protein-peptide complexes.**

| **ID** | **Pose1** | **Pose2** | **Pose3** | **Pose4** | **Pose5** | **Pose6** | **Pose7** | **Pose8** | **Pose9** | **Pose10** | **Pose11** | **Pose12** | **Pose13** | **Pose14** | **Pose15** | **Pose16** | **Pose17** | **Pose18** | **Pose19** | **Pose20** |
| --- | --- | --- | --- | --- | --- | --- | --- | --- | --- | --- | --- | --- | --- | --- | --- | --- | --- | --- | --- | --- |
| 1cjr | 16.46 | 15.38 | 18.39 | 15.58 | 16.35 | 14.21 | 14.66 | 14.71 | 12.82 | 14.08 | 14.60 | 14.37 | 14.92 | 22.75 | 14.20 | 13.43 | 18.99 | 19.79 | 21.24 | 19.96 |
| 1cka | 2.04 | 1.76 | 1.77 | 14.25 | 1.53 | 1.58 | 4.37 | 1.50 | 5.37 | 6.14 | 11.18 | 3.79 | 4.74 | 4.83 | 4.84 | 4.57 | 14.61 | 15.26 | 4.33 | 2.59 |
| 1cvu | 45.72 | 42.76 | 41.88 | 41.96 | 44.03 | 37.37 | 42.50 | 43.67 | 41.93 | 43.02 | 43.53 | 43.92 | 43.50 | 54.65 | 42.75 | 44.58 | 40.41 | 45.57 | 39.97 | 46.30 |
| 1d4t | 27.03 | 27.74 | 20.85 | 27.20 | 27.87 | 27.87 | 28.76 | 30.10 | 30.04 | 26.03 | 27.78 | 28.89 | 29.51 | 29.19 | 29.21 | 28.13 | 29.10 | 29.78 | 27.68 | 27.42 |
| 1eg4 | 41.63 | 42.04 | 42.70 | 41.47 | 35.91 | 44.16 | 44.40 | 26.86 | 37.31 | 41.20 | 21.30 | 28.74 | 35.68 | 36.44 | 43.22 | 35.75 | 12.95 | 43.27 | 42.27 | 44.52 |
| 1h6w | 39.82 | 39.11 | 40.12 | 39.24 | 39.24 | 39.30 | 39.64 | 39.40 | 38.60 | 38.64 | 39.00 | 39.35 | 38.88 | 52.36 | 52.34 | 39.78 | 35.41 | 52.14 | 38.90 | 50.99 |
| 1hc9 | 15.66 | 18.20 | 18.76 | 13.66 | 17.81 | 11.25 | 15.78 | 13.63 | 13.54 | 13.65 | 19.77 | 15.22 | 13.62 | 18.95 | 13.36 | 23.10 | 14.79 | 14.55 | 15.10 | 13.29 |
| 1jbu | 22.92 | 22.88 | 21.70 | 21.99 | 22.83 | 11.04 | 10.81 | 10.23 | 24.78 | 10.45 | 45.14 | 11.00 | 22.85 | 25.76 | 22.73 | 13.69 | 15.97 | 22.97 | 40.61 | 11.14 |
| 1k5n | 22.02 | 21.74 | 22.60 | 21.64 | 21.58 | 21.67 | 22.23 | 8.56 | 21.96 | 21.83 | 21.47 | 20.70 | 22.61 | 20.81 | 22.34 | 21.45 | 22.28 | 22.53 | 21.47 | 21.91 |
| 1mfg | 16.25 | 16.61 | 13.54 | 16.87 | 14.51 | 14.34 | 16.98 | 14.25 | 24.88 | 14.90 | 20.18 | 11.86 | 15.52 | 14.23 | 16.16 | 16.63 | 13.39 | 17.55 | 16.45 | 22.24 |
| 1nln | 30.56 | 30.57 | 33.61 | 31.85 | 32.64 | 31.40 | 32.80 | 31.75 | 32.65 | 31.85 | 33.44 | 32.06 | 31.75 | 32.19 | 33.54 | 31.39 | 31.91 | 33.05 | 33.20 | 32.34 |
| 1nq7 | 32.64 | 38.41 | 32.94 | 38.86 | 32.02 | 35.81 | 33.41 | 32.13 | 24.01 | 35.42 | 34.91 | 32.62 | 35.84 | 32.84 | 33.88 | 34.76 | 23.74 | 33.58 | 34.56 | 32.82 |
| 1ntv | 15.67 | 19.73 | 15.73 | 13.90 | 30.50 | 14.04 | 15.84 | 14.18 | 15.32 | 31.96 | 22.24 | 15.69 | 16.82 | 16.93 | 16.67 | 30.59 | 16.47 | 16.28 | 26.70 | 16.72 |
| 1nx1 | 33.76 | 32.98 | 35.16 | 33.05 | 33.21 | 35.17 | 33.05 | 33.06 | 33.06 | 33.61 | 32.69 | 33.42 | 33.01 | 33.17 | 33.28 | 33.05 | 33.50 | 32.91 | 32.90 | 32.99 |
| 1oai | 8.36 | 8.23 | 8.49 | 9.99 | 7.70 | 8.01 | 8.08 | 8.30 | 10.48 | 7.56 | 9.80 | 7.22 | 8.25 | 9.88 | 8.95 | 8.01 | 11.53 | 11.14 | 10.34 | 8.48 |
| 1oj5 | 25.37 | 22.47 | 24.60 | 24.42 | 26.62 | 28.01 | 24.74 | 22.81 | 32.62 | 24.11 | 32.77 | 22.47 | 10.13 | 24.83 | 7.68 | 19.34 | 10.62 | 26.84 | 32.65 | 9.25 |
| 1ou8 | 11.48 | 11.28 | 11.70 | 12.49 | 14.21 | 12.77 | 13.37 | 13.72 | 26.24 | 23.25 | 13.00 | 7.51 | 13.32 | 12.78 | 11.03 | 11.90 | 12.26 | 32.04 | 18.15 | 16.24 |
| 1ow6 | 12.40 | 12.61 | 13.26 | 13.15 | 10.56 | 13.63 | 12.27 | 13.91 | 12.39 | 12.90 | 11.75 | 12.32 | 11.00 | 12.24 | 15.04 | 12.49 | 12.29 | 14.33 | 29.47 | 13.67 |
| 1pzl | 36.56 | 29.84 | 30.76 | 29.65 | 29.11 | 30.83 | 33.81 | 29.57 | 40.90 | 29.20 | 28.66 | 34.70 | 26.85 | 31.23 | 36.81 | 30.63 | 35.96 | 24.59 | 33.89 | 34.51 |
| 1qkz | 8.91 | 34.97 | 36.39 | 33.84 | 36.40 | 36.06 | 35.65 | 37.03 | 36.08 | 36.54 | 35.58 | 35.44 | 36.58 | 35.95 | 33.79 | 14.29 | 36.47 | 33.87 | 12.91 | 35.52 |
| 1rst | 8.19 | 8.00 | 8.38 | 7.84 | 8.20 | 6.74 | 7.63 | 6.62 | 7.42 | 7.72 | 6.90 | 13.03 | 7.10 | 6.79 | 7.89 | 9.76 | 10.28 | 7.36 | 6.39 | 8.02 |
| 1rxz | 20.02 | 19.98 | 22.57 | 20.59 | 23.42 | 20.68 | 23.43 | 23.23 | 38.48 | 22.12 | 20.28 | 24.61 | 22.10 | 22.09 | 21.51 | 22.08 | 38.37 | 23.88 | 39.02 | 23.71 |
| 1sfi | 20.25 | 20.20 | 20.20 | 20.19 | 17.94 | 21.25 | 20.21 | 20.53 | 18.66 | 21.23 | 19.62 | 19.95 | 20.89 | 20.30 | 19.67 | 15.81 | 21.07 | 17.83 | 18.10 | 13.27 |
| 1ssh | 2.77 | 2.13 | 4.79 | 1.41 | 2.00 | 2.20 | 1.81 | 6.55 | 2.71 | 2.63 | 5.52 | 2.03 | 2.36 | 5.42 | 3.82 | 6.21 | 2.64 | 4.26 | 2.37 | 1.73 |
| 1t08 | 18.86 | 20.86 | 21.60 | 33.43 | 21.18 | 20.95 | 21.47 | 22.92 | 21.42 | 20.05 | 18.75 | 26.06 | 25.11 | 25.19 | 20.77 | 20.93 | 19.88 | 25.15 | 29.84 | 20.61 |
| 1t4f | 5.60 | 5.71 | 23.14 | 5.82 | 13.69 | 23.60 | 7.57 | 14.62 | 7.54 | 17.56 | 5.79 | 7.09 | 6.28 | 6.63 | 5.89 | 18.10 | 13.39 | 21.24 | 22.13 | 21.48 |
| 1t7r | 34.76 | 31.86 | 11.82 | 11.68 | 36.33 | 11.59 | 15.52 | 38.58 | 14.24 | 11.82 | 10.61 | 11.60 | 39.52 | 37.16 | 11.02 | 11.82 | 11.73 | 13.18 | 30.56 | 38.16 |
| 1tfc | 10.80 | 11.86 | 11.22 | 10.37 | 12.72 | 8.32 | 12.27 | 11.12 | 10.60 | 13.63 | 10.67 | 9.54 | 12.29 | 11.66 | 10.48 | 11.15 | 10.90 | 10.59 | 10.01 | 11.47 |
| 1u00 | 19.06 | 20.66 | 20.51 | 15.24 | 19.35 | 14.53 | 19.49 | 15.96 | 19.08 | 15.18 | 14.60 | 14.47 | 14.00 | 13.76 | 14.74 | 19.54 | 15.67 | 15.99 | 16.23 | 14.81 |
| 1uj0 | 6.53 | 7.56 | 7.05 | 4.16 | 6.65 | 4.87 | 4.80 | 6.95 | 6.59 | 10.43 | 15.27 | 16.08 | 6.79 | 15.24 | 7.54 | 8.21 | 12.78 | 7.73 | 14.99 | 11.44 |
| 1x2r | 14.41 | 14.77 | 14.79 | 14.19 | 13.98 | 14.20 | 15.05 | 13.29 | 13.50 | 13.35 | 13.53 | 24.03 | 37.60 | 9.28 | 37.30 | 9.18 | 8.65 | 13.09 | 8.96 | 26.83 |
| 1xoc | 24.97 | 29.80 | 34.41 | 34.30 | 28.98 | 28.68 | 29.10 | 28.27 | 28.14 | 28.51 | 28.59 | 29.20 | 28.71 | 28.19 | 26.91 | 29.06 | 35.59 | 26.89 | 28.07 | 28.58 |
| 1ymt | 10.07 | 9.64 | 32.44 | 38.52 | 8.72 | 10.29 | 8.70 | 11.07 | 34.49 | 12.49 | 37.83 | 9.51 | 31.89 | 31.45 | 32.15 | 9.30 | 34.53 | 37.12 | 9.78 | 35.09 |
| 1yuc | 21.36 | 34.00 | 34.06 | 33.85 | 36.11 | 33.30 | 28.84 | 28.25 | 33.41 | 38.75 | 33.50 | 36.15 | 32.99 | 36.00 | 35.78 | 33.06 | 37.86 | 24.83 | 20.79 | 28.00 |
| 1ywo | 11.12 | 15.54 | 13.73 | 6.08 | 13.19 | 17.70 | 15.06 | 16.48 | 4.58 | 9.62 | 12.47 | 6.77 | 6.26 | 15.83 | 14.90 | 17.03 | 5.36 | 4.07 | 3.68 | 6.33 |
| 2a25 | 25.04 | 25.78 | 25.64 | 25.20 | 25.05 | 12.11 | 22.91 | 24.58 | 27.48 | 9.82 | 23.14 | 9.91 | 26.47 | 25.54 | 25.52 | 10.38 | 23.81 | 4.30 | 25.12 | 24.70 |
| 2a3i | 34.06 | 19.00 | 34.73 | 29.96 | 4.83 | 29.41 | 8.85 | 17.83 | 41.20 | 32.19 | 38.92 | 35.11 | 40.67 | 15.34 | 40.65 | 31.71 | 40.65 | 7.57 | 6.86 | 24.74 |
| 2aq9 | 29.27 | 27.93 | 30.39 | 30.05 | 34.30 | 37.02 | 31.30 | 30.70 | 29.68 | 36.50 | 28.90 | 39.14 | 28.93 | 32.37 | 29.26 | 36.96 | 36.19 | 28.30 | 30.92 | 40.32 |
| 2b9h | 5.05 | 6.61 | 14.72 | 42.39 | 42.27 | 43.23 | 16.50 | 16.16 | 16.09 | 29.08 | 14.15 | 15.95 | 6.68 | 16.08 | 15.66 | 9.85 | 29.27 | 29.51 | 33.12 | 32.10 |
| 2bba | 10.29 | 10.01 | 27.38 | 28.68 | 26.82 | 10.62 | 27.85 | 7.15 | 29.24 | 28.37 | 8.55 | 6.33 | 5.70 | 5.40 | 5.93 | 8.27 | 28.16 | 6.23 | 26.95 | 27.46 |
| 2cch | 33.37 | 34.02 | 36.62 | 33.06 | 36.70 | 36.72 | 36.60 | 36.43 | 9.16 | 36.17 | 36.62 | 36.18 | 36.70 | 36.06 | 32.56 | 35.98 | 38.77 | 36.54 | 36.16 | 37.68 |
| 2ce8 | 26.84 | 32.96 | 30.99 | 30.27 | 29.61 | 31.64 | 28.15 | 30.06 | 30.67 | 29.24 | 29.00 | 29.59 | 31.00 | 30.26 | 30.69 | 30.11 | 28.15 | 29.25 | 29.20 | 30.16 |
| 2d0n | 5.25 | 7.13 | 6.83 | 8.57 | 7.18 | 6.30 | 7.67 | 7.75 | 6.00 | 7.92 | 6.45 | 12.67 | 7.52 | 13.56 | 8.88 | 7.19 | 7.77 | 7.95 | 12.53 | 11.04 |
| 2drk | 17.61 | 17.73 | 17.89 | 17.69 | 17.84 | 17.62 | 17.37 | 9.39 | 11.01 | 11.08 | 10.96 | 17.65 | 17.86 | 11.49 | 2.37 | 10.39 | 9.46 | 18.30 | 18.18 | 17.60 |
| 2dyp | 25.45 | 23.83 | 24.30 | 22.40 | 20.17 | 23.43 | 24.44 | 20.37 | 24.06 | 24.77 | 20.03 | 24.60 | 20.13 | 19.53 | 23.88 | 23.87 | 19.56 | 20.04 | 23.10 | 24.02 |
| 2fff | 44.68 | 46.84 | 33.10 | 52.07 | 53.35 | 47.25 | 37.35 | 47.43 | 51.88 | 45.41 | 43.71 | 43.23 | 42.34 | 51.54 | 43.36 | 43.34 | 42.73 | 46.78 | 46.44 | 53.72 |
| 2ffu | 14.90 | 15.14 | 15.39 | 18.72 | 15.04 | 18.33 | 5.43 | 14.51 | 18.38 | 18.59 | 16.79 | 15.63 | 18.04 | 14.34 | 15.39 | 17.47 | 16.55 | 5.77 | 15.88 | 19.14 |
| 2fka | 4.77 | 5.70 | 6.35 | 5.92 | 5.22 | 4.78 | 5.62 | 5.09 | 5.73 | 6.06 | 5.63 | 6.61 | 4.77 | 6.50 | 5.54 | 5.28 | 5.54 | 5.67 | 6.44 | 4.86 |
| 2fmf | 5.75 | 9.60 | 6.99 | 10.43 | 9.62 | 9.78 | 10.16 | 9.63 | 11.78 | 10.12 | 12.10 | 12.43 | 9.78 | 10.26 | 12.26 | 12.25 | 10.12 | 10.60 | 13.11 | 10.18 |
| 2fts | 34.91 | 29.93 | 30.58 | 26.05 | 24.72 | 30.45 | 30.28 | 24.53 | 28.67 | 25.74 | 36.43 | 25.76 | 35.37 | 34.03 | 25.71 | 33.91 | 34.64 | 32.54 | 29.75 | 26.86 |
| 2fvj | 25.06 | 23.30 | 23.39 | 23.86 | 24.49 | 24.25 | 22.75 | 24.35 | 30.36 | 19.17 | 13.47 | 19.04 | 23.57 | 18.79 | 27.55 | 20.14 | 27.39 | 18.24 | 47.33 | 23.15 |
| 2ho2 | 7.52 | 14.56 | 7.43 | 13.40 | 13.74 | 3.53 | 13.99 | 3.72 | 14.02 | 15.19 | 18.09 | 13.79 | 13.39 | 3.47 | 16.93 | 14.00 | 16.36 | 14.67 | 13.10 | 19.44 |
| 2ht9 | 30.73 | 29.73 | 30.68 | 31.02 | 33.54 | 30.22 | 32.03 | 34.12 | 32.30 | 30.09 | 30.58 | 31.28 | 35.91 | 33.81 | 34.50 | 27.16 | 32.99 | 31.75 | 29.69 | 31.09 |
| 2o02 | 20.81 | 9.53 | 21.54 | 11.64 | 21.09 | 21.51 | 20.94 | 8.88 | 21.53 | 21.02 | 9.12 | 21.39 | 9.70 | 14.19 | 18.08 | 21.87 | 10.37 | 14.73 | 21.70 | 21.01 |
| 2o4j | 39.88 | 41.14 | 40.41 | 40.42 | 41.38 | 41.72 | 41.61 | 41.22 | 40.48 | 41.47 | 42.20 | 40.43 | 40.80 | 41.52 | 41.62 | 41.38 | 41.72 | 42.01 | 40.74 | 41.11 |
| 2o9v | 14.02 | 13.71 | 13.84 | 13.85 | 13.80 | 14.32 | 14.01 | 13.82 | 14.09 | 13.29 | 13.60 | 13.82 | 13.83 | 13.92 | 13.66 | 13.94 | 13.53 | 13.65 | 14.55 | 13.80 |
| 2oei | 4.57 | 14.96 | 16.11 | 11.35 | 15.75 | 15.62 | 4.73 | 18.33 | 4.63 | 4.23 | 12.31 | 19.20 | 18.96 | 10.25 | 19.83 | 19.91 | 15.53 | 18.58 | 16.36 | 6.09 |
| 2p0w | 22.37 | 21.86 | 22.38 | 22.35 | 22.69 | 23.45 | 22.09 | 24.57 | 23.11 | 22.22 | 25.90 | 25.87 | 23.58 | 34.04 | 32.03 | 23.31 | 34.72 | 34.96 | 21.24 | 33.22 |
| 2p1o | 32.55 | 30.24 | 30.73 | 32.34 | 35.99 | 32.97 | 33.55 | 31.47 | 27.45 | 28.42 | 31.79 | 36.23 | 20.40 | 35.96 | 20.99 | 32.43 | 19.83 | 20.56 | 43.23 | 20.66 |
| 2p1t | 33.38 | 31.41 | 33.86 | 30.23 | 32.12 | 31.83 | 31.77 | 32.43 | 33.19 | 27.63 | 13.99 | 17.36 | 33.60 | 16.20 | 31.25 | 30.20 | 18.09 | 17.69 | 27.90 | 17.53 |
| 2p54 | 44.70 | 45.12 | 45.00 | 44.95 | 44.95 | 43.91 | 43.64 | 21.77 | 43.69 | 44.38 | 44.43 | 45.62 | 45.34 | 44.53 | 45.40 | 45.49 | 45.33 | 44.62 | 44.67 | 18.75 |
| 2peh | 32.58 | 29.71 | 30.19 | 22.84 | 30.17 | 31.73 | 31.33 | 21.80 | 22.79 | 31.93 | 28.53 | 31.73 | 14.23 | 30.12 | 14.38 | 14.24 | 13.63 | 21.75 | 14.13 | 31.48 |
| 2pux | 15.57 | 15.74 | 15.64 | 14.61 | 15.80 | 16.79 | 15.73 | 14.70 | 16.87 | 15.93 | 15.73 | 16.09 | 16.43 | 16.76 | 15.53 | 15.53 | 16.99 | 16.19 | 16.10 | 36.99 |
| 2puy | 15.54 | 15.67 | 14.45 | 15.47 | 15.54 | 16.74 | 15.53 | 15.22 | 15.60 | 15.87 | 16.55 | 15.05 | 15.76 | 15.29 | 15.69 | 16.90 | 15.35 | 16.18 | 14.23 | 17.28 |
| 2qbx | 25.79 | 24.08 | 31.74 | 24.01 | 26.65 | 21.40 | 22.87 | 35.88 | 15.01 | 40.20 | 25.17 | 23.80 | 40.17 | 23.81 | 10.37 | 35.94 | 27.48 | 26.09 | 23.61 | 37.39 |
| 2qos | 23.68 | 26.49 | 26.05 | 23.79 | 26.46 | 26.47 | 7.84 | 14.39 | 23.67 | 11.76 | 13.59 | 14.35 | 23.87 | 8.51 | 24.54 | 24.42 | 11.28 | 7.80 | 7.49 | 8.26 |
| 2qse | 31.64 | 34.14 | 37.25 | 39.29 | 36.45 | 37.36 | 37.26 | 39.13 | 35.23 | 38.38 | 34.14 | 37.92 | 40.10 | 34.99 | 38.57 | 12.76 | 33.26 | 36.41 | 38.01 | 35.35 |
| 2r7g | 29.00 | 37.78 | 20.14 | 37.03 | 23.10 | 37.78 | 37.63 | 24.51 | 36.73 | 37.70 | 38.02 | 37.52 | 38.44 | 37.46 | 23.24 | 38.67 | 38.48 | 37.80 | 37.83 | 37.60 |
| 2r9q | 8.97 | 6.72 | 6.61 | 6.86 | 16.84 | 6.52 | 6.72 | 16.85 | 8.72 | 7.11 | 7.23 | 7.53 | 7.83 | 7.12 | 17.48 | 7.28 | 7.34 | 7.19 | 7.92 | 7.34 |
| 2v8y | 15.04 | 34.15 | 36.97 | 23.65 | 23.56 | 36.56 | 38.66 | 37.51 | 18.34 | 40.35 | 23.46 | 37.20 | 38.61 | 30.94 | 23.61 | 37.21 | 23.15 | 22.49 | 37.90 | 23.80 |
| 2vkn | 5.36 | 5.98 | 5.84 | 6.74 | 6.92 | 6.28 | 7.35 | 6.17 | 5.87 | 6.60 | 12.36 | 6.79 | 5.95 | 7.15 | 6.13 | 4.71 | 8.61 | 5.35 | 6.39 | 9.63 |
| 2vr3 | 27.24 | 26.58 | 25.52 | 26.80 | 24.50 | 32.81 | 33.69 | 27.34 | 26.22 | 30.01 | 33.27 | 24.17 | 28.05 | 29.63 | 29.09 | 32.73 | 16.10 | 19.16 | 19.10 | 28.77 |
| 2vwf | 14.22 | 15.68 | 31.35 | 11.54 | 14.95 | 25.81 | 13.39 | 16.09 | 23.46 | 13.17 | 22.89 | 19.84 | 21.50 | 10.34 | 19.74 | 25.45 | 8.61 | 21.67 | 8.62 | 22.74 |
| 2w2u | 9.88 | 8.13 | 7.84 | 8.70 | 8.24 | 7.75 | 9.95 | 9.39 | 8.67 | 21.40 | 20.08 | 21.81 | 7.84 | 6.79 | 23.72 | 20.17 | 8.46 | 8.26 | 10.06 | 7.46 |
| 2whx | 62.46 | 61.83 | 62.40 | 61.68 | 58.14 | 63.94 | 61.86 | 61.40 | 63.20 | 62.52 | 29.47 | 61.66 | 63.63 | 61.38 | 61.09 | 27.67 | 60.76 | 30.40 | 63.26 | 61.23 |
| 2xrw | 41.22 | 32.42 | 28.75 | 39.39 | 37.38 | 39.39 | 39.90 | 40.07 | 38.55 | 39.38 | 28.61 | 39.69 | 39.89 | 35.59 | 38.16 | 25.29 | 29.92 | 27.59 | 28.53 | 39.45 |
| 2xu7 | 5.40 | 6.23 | 6.19 | 40.64 | 41.43 | 9.08 | 6.29 | 42.79 | 43.57 | 6.52 | 11.25 | 40.15 | 11.60 | 43.39 | 42.78 | 6.39 | 43.15 | 41.81 | 9.08 | 42.49 |
| 2xvc | 21.71 | 22.34 | 24.10 | 21.02 | 24.53 | 23.00 | 20.86 | 19.31 | 23.25 | 22.69 | 22.08 | 22.68 | 24.79 | 22.77 | 25.79 | 13.35 | 28.39 | 24.53 | 19.69 | 22.35 |
| 2zjd | 4.53 | 5.34 | 6.48 | 5.03 | 5.26 | 5.00 | 6.10 | 11.54 | 5.90 | 5.05 | 5.55 | 5.59 | 30.88 | 8.09 | 22.10 | 15.39 | 9.07 | 7.34 | 15.41 | 25.83 |
| 3asl | 22.89 | 16.95 | 23.12 | 8.91 | 17.70 | 8.73 | 19.16 | 20.38 | 9.76 | 25.86 | 19.69 | 19.74 | 19.53 | 17.76 | 9.00 | 20.62 | 16.77 | 17.36 | 22.83 | 19.97 |
| 3awr | 19.27 | 19.39 | 19.30 | 19.46 | 19.28 | 17.12 | 20.80 | 9.57 | 18.46 | 19.26 | 18.87 | 7.23 | 7.66 | 8.13 | 7.65 | 7.44 | 18.72 | 19.83 | 6.55 | 17.81 |
| 3ayu | 17.69 | 17.55 | 7.09 | 7.56 | 8.41 | 8.30 | 8.46 | 24.63 | 8.81 | 8.24 | 8.94 | 8.86 | 9.46 | 9.08 | 17.63 | 17.75 | 8.83 | 6.70 | 7.41 | 6.66 |
| 3bfq | 31.59 | 31.35 | 31.46 | 31.44 | 32.17 | 8.89 | 12.51 | 32.71 | 7.39 | 12.39 | 6.79 | 11.97 | 6.20 | 31.55 | 35.77 | 28.24 | 31.49 | 20.44 | 30.04 | 10.94 |
| 3c3r | 39.41 | 38.87 | 39.18 | 39.64 | 30.20 | 40.09 | 39.01 | 38.04 | 39.71 | 20.01 | 40.88 | 37.62 | 39.54 | 39.75 | 39.52 | 36.72 | 39.89 | 40.87 | 39.75 | 38.40 |
| 3d32 | 23.30 | 23.62 | 17.90 | 27.54 | 21.96 | 24.45 | 24.26 | 23.30 | 27.93 | 18.23 | 8.67 | 18.93 | 23.90 | 29.73 | 20.41 | 27.18 | 18.27 | 21.84 | 22.09 | 18.99 |
| 3ds4 | 25.74 | 24.54 | 23.86 | 24.07 | 24.14 | 25.65 | 23.81 | 24.24 | 24.17 | 23.89 | 25.24 | 24.67 | 22.74 | 25.84 | 24.63 | 24.06 | 26.31 | 24.64 | 27.51 | 24.10 |
| 3ery | 7.33 | 4.73 | 4.87 | 4.38 | 5.32 | 5.12 | 4.64 | 4.69 | 8.12 | 3.70 | 7.60 | 3.64 | 4.41 | 4.94 | 3.57 | 6.14 | 15.66 | 16.39 | 6.42 | 4.38 |
| 3fdo | 21.57 | 19.51 | 18.15 | 18.01 | 19.29 | 8.41 | 10.35 | 18.70 | 11.54 | 10.73 | 9.09 | 12.52 | 7.56 | 8.25 | 9.67 | 8.04 | 10.40 | 10.75 | 7.67 | 8.46 |
| 3g2s | 12.70 | 17.82 | 19.47 | 16.41 | 14.41 | 15.53 | 12.33 | 7.78 | 14.13 | 11.65 | 15.03 | 17.63 | 21.96 | 7.26 | 31.28 | 13.16 | 11.54 | 12.53 | 28.56 | 12.36 |
| 3gyt | 31.19 | 43.59 | 31.05 | 30.65 | 42.28 | 29.07 | 31.10 | 42.37 | 30.70 | 42.48 | 33.08 | 10.49 | 43.16 | 30.45 | 31.30 | 31.02 | 40.60 | 25.08 | 30.79 | 31.09 |
| 3h1z | 32.80 | 25.36 | 28.92 | 28.37 | 27.92 | 55.85 | 28.18 | 27.44 | 28.72 | 28.79 | 55.09 | 28.39 | 56.52 | 28.33 | 31.36 | 55.34 | 28.30 | 36.05 | 29.02 | 29.61 |
| 3i5r | 15.10 | 12.32 | 15.00 | 16.27 | 16.07 | 16.35 | 14.63 | 15.68 | 9.72 | 16.58 | 16.37 | 15.59 | 16.27 | 16.02 | 11.40 | 13.89 | 16.53 | 16.65 | 15.09 | 3.53 |
| 3ivv | 15.19 | 14.54 | 14.94 | 14.55 | 15.39 | 15.36 | 15.13 | 14.63 | 14.62 | 16.48 | 15.46 | 14.73 | 14.19 | 14.66 | 13.95 | 26.43 | 14.26 | 15.28 | 15.29 | 15.32 |
| 3kmr | 34.16 | 32.30 | 33.29 | 33.23 | 33.68 | 33.10 | 33.13 | 33.31 | 34.00 | 33.51 | 34.31 | 33.06 | 32.26 | 32.30 | 33.90 | 33.01 | 33.93 | 32.70 | 32.77 | 33.15 |
| 3kuj | 22.56 | 22.09 | 21.62 | 20.20 | 23.67 | 22.12 | 13.76 | 22.50 | 24.02 | 23.37 | 24.05 | 24.07 | 24.36 | 21.88 | 31.04 | 21.49 | 31.14 | 31.41 | 23.52 | 21.85 |
| 3kus | 19.20 | 22.26 | 20.83 | 17.68 | 18.42 | 19.54 | 16.24 | 16.37 | 17.89 | 19.01 | 17.38 | 15.63 | 17.62 | 15.47 | 17.73 | 20.36 | 20.30 | 17.44 | 17.97 | 20.12 |
| 3l0e | 39.76 | 39.22 | 34.64 | 21.75 | 39.11 | 39.16 | 32.01 | 32.55 | 34.39 | 34.99 | 30.40 | 25.46 | 13.78 | 19.82 | 38.05 | 21.86 | 34.88 | 36.72 | 32.14 | 26.97 |
| 3ll8 | 35.26 | 34.84 | 35.96 | 35.80 | 34.90 | 35.71 | 35.12 | 35.56 | 35.61 | 35.28 | 36.79 | 35.77 | 34.61 | 35.95 | 35.02 | 36.81 | 34.44 | 35.64 | 35.19 | 39.17 |
| 3llz | 18.19 | 18.61 | 18.76 | 18.93 | 18.12 | 17.37 | 18.98 | 18.12 | 17.75 | 16.88 | 17.90 | 18.37 | 17.50 | 18.25 | 17.01 | 18.24 | 17.80 | 26.39 | 18.26 | 17.93 |
| 3obq | 16.93 | 16.85 | 16.60 | 17.04 | 6.32 | 6.58 | 25.14 | 24.39 | 4.71 | 26.19 | 24.36 | 24.39 | 17.00 | 24.52 | 23.73 | 24.32 | 25.33 | 6.01 | 16.82 | 29.10 |
| 3olf | 12.73 | 34.17 | 13.49 | 13.28 | 32.96 | 14.02 | 13.29 | 33.21 | 34.14 | 34.14 | 33.50 | 32.92 | 13.79 | 33.33 | 33.91 | 33.36 | 32.51 | 32.91 | 14.06 | 44.53 |
| 3p72 | 11.06 | 12.71 | 13.26 | 16.35 | 12.08 | 14.23 | 13.35 | 13.62 | 11.35 | 13.83 | 12.06 | 13.12 | 13.87 | 13.88 | 12.30 | 15.82 | 13.09 | 14.36 | 10.09 | 33.87 |
| 3p8f | 32.66 | 33.16 | 12.97 | 17.35 | 17.33 | 17.34 | 31.72 | 18.37 | 17.09 | 30.98 | 32.10 | 16.08 | 16.47 | 16.86 | 18.15 | 12.72 | 17.66 | 17.45 | 18.80 | 18.63 |
| 3ptl | 21.87 | 17.89 | 19.27 | 18.06 | 21.06 | 18.40 | 21.17 | 18.08 | 17.38 | 18.26 | 18.08 | 17.51 | 20.30 | 17.33 | 18.32 | 18.06 | 17.95 | 17.40 | 18.37 | 18.28 |
| 3qis | 31.32 | 30.09 | 10.26 | 9.70 | 6.04 | 32.65 | 8.12 | 8.98 | 9.09 | 7.93 | 6.72 | 9.04 | 9.52 | 10.75 | 8.49 | 6.66 | 7.03 | 34.08 | 32.15 | 9.82 |
| 3rm1 | 20.86 | 20.81 | 21.55 | 18.15 | 17.47 | 16.96 | 17.67 | 19.38 | 17.39 | 17.62 | 16.42 | 16.15 | 22.97 | 17.76 | 18.55 | 17.28 | 17.57 | 22.91 | 22.65 | 15.78 |
| 3rqg | 36.19 | 27.96 | 35.42 | 36.33 | 32.62 | 27.57 | 28.10 | 35.47 | 35.85 | 35.53 | 33.45 | 32.26 | 33.77 | 32.17 | 34.21 | 29.60 | 34.31 | 37.37 | 36.94 | 32.32 |
| 3sfj | 20.88 | 20.40 | 19.97 | 19.52 | 19.25 | 20.29 | 19.15 | 8.49 | 8.64 | 8.81 | 19.00 | 9.87 | 8.74 | 9.12 | 8.44 | 9.39 | 8.44 | 8.65 | 9.24 | 8.93 |
| 3so6 | 7.25 | 7.32 | 7.36 | 21.62 | 26.94 | 17.33 | 26.37 | 25.97 | 24.37 | 24.55 | 32.03 | 26.10 | 26.37 | 28.05 | 17.21 | 28.93 | 21.78 | 25.62 | 25.20 | 16.02 |
| 3tjv | 18.34 | 18.18 | 3.27 | 4.23 | 18.53 | 5.27 | 17.08 | 4.71 | 17.87 | 18.19 | 19.17 | 17.34 | 18.87 | 19.66 | 18.28 | 19.07 | 20.85 | 17.82 | 20.27 | 10.51 |
| 3tzy | 20.56 | 20.08 | 21.80 | 9.54 | 9.05 | 10.42 | 20.20 | 10.45 | 12.92 | 13.14 | 14.00 | 33.38 | 37.54 | 10.06 | 10.13 | 37.43 | 13.35 | 12.98 | 10.23 | 8.14 |
| 3u9q | 24.85 | 24.90 | 26.35 | 25.54 | 20.86 | 24.35 | 25.53 | 14.10 | 11.31 | 27.47 | 11.90 | 14.13 | 13.43 | 12.62 | 13.58 | 11.66 | 32.63 | 43.77 | 18.46 | 13.27 |
| 3up3 | 38.76 | 40.76 | 40.20 | 38.52 | 40.98 | 39.04 | 40.54 | 38.19 | 40.25 | 25.79 | 28.87 | 38.91 | 40.57 | 6.09 | 45.32 | 5.77 | 5.75 | 43.47 | 6.06 | 27.45 |
| 3v2x | 19.38 | 19.39 | 19.75 | 19.92 | 19.55 | 19.46 | 19.52 | 19.48 | 19.44 | 19.68 | 19.58 | 20.84 | 19.40 | 19.56 | 16.88 | 19.69 | 21.22 | 8.93 | 9.30 | 19.32 |
| 3vtc | 33.30 | 13.24 | 34.18 | 33.33 | 30.98 | 34.50 | 34.63 | 31.70 | 12.99 | 32.12 | 39.79 | 32.09 | 10.56 | 13.04 | 39.43 | 11.64 | 15.82 | 41.74 | 8.32 | 41.94 |
| 3w1b | 40.58 | 41.05 | 39.00 | 39.83 | 38.18 | 36.92 | 39.97 | 40.47 | 36.28 | 39.19 | 39.48 | 37.93 | 39.98 | 40.16 | 38.52 | 38.83 | 39.60 | 42.96 | 38.67 | 39.72 |
| 3zqh | 12.26 | 11.09 | 9.83 | 13.12 | 11.23 | 11.60 | 11.95 | 11.32 | 11.26 | 10.29 | 13.09 | 11.28 | 12.24 | 11.69 | 12.77 | 11.38 | 13.03 | 12.02 | 12.85 | 13.66 |
| 4b4n | 27.71 | 25.92 | 25.59 | 27.10 | 26.34 | 27.70 | 25.29 | 27.06 | 27.97 | 27.40 | 27.06 | 26.98 | 25.54 | 22.19 | 27.47 | 27.77 | 26.45 | 26.60 | 27.09 | 25.27 |
| 4dcb | 37.31 | 37.57 | 38.61 | 36.66 | 35.59 | 42.08 | 38.39 | 51.33 | 37.60 | 36.97 | 41.80 | 38.20 | 36.69 | 23.30 | 38.28 | 41.45 | 38.03 | 40.67 | 41.34 | 38.46 |
| 4e34 | 21.16 | 23.19 | 24.20 | 22.84 | 21.50 | 22.28 | 22.65 | 22.16 | 22.20 | 23.55 | 22.96 | 22.68 | 21.91 | 21.51 | 21.59 | 20.53 | 21.26 | 22.96 | 22.11 | 21.13 |
| 4eik | 3.84 | 5.09 | 6.98 | 14.61 | 14.41 | 15.78 | 19.68 | 5.01 | 13.09 | 22.30 | 3.17 | 4.50 | 6.38 | 18.83 | 3.49 | 19.87 | 5.72 | 22.24 | 6.44 | 4.48 |
| 4ery | 8.84 | 9.17 | 9.00 | 8.79 | 8.94 | 8.51 | 8.80 | 14.53 | 14.56 | 8.79 | 9.75 | 9.45 | 32.43 | 16.22 | 8.53 | 9.48 | 31.34 | 15.97 | 9.72 | 17.94 |
| 4f14 | 7.37 | 7.19 | 5.71 | 7.37 | 7.88 | 6.25 | 6.48 | 6.58 | 7.67 | 4.62 | 6.56 | 6.32 | 5.67 | 4.94 | 5.67 | 6.93 | 6.16 | 6.60 | 4.11 | 4.53 |
| 4f1z | 31.40 | 27.87 | 18.88 | 32.79 | 32.53 | 26.83 | 32.69 | 30.93 | 26.78 | 30.90 | 31.66 | 26.93 | 31.50 | 30.48 | 27.59 | 31.61 | 22.41 | 30.91 | 26.64 | 26.85 |
| 4gq6 | 12.34 | 30.19 | 13.91 | 13.39 | 13.03 | 12.77 | 12.30 | 12.99 | 13.31 | 12.95 | 13.09 | 12.78 | 13.38 | 13.31 | 12.93 | 12.03 | 12.64 | 12.90 | 12.79 | 12.26 |
| 4gxl | 35.50 | 33.39 | 35.36 | 32.27 | 11.76 | 34.56 | 32.38 | 34.26 | 33.08 | 32.69 | 32.40 | 14.01 | 19.40 | 32.89 | 32.51 | 11.38 | 16.08 | 14.87 | 34.24 | 13.20 |
| 4gyw | 13.96 | 39.93 | 12.12 | 13.18 | 17.95 | 20.64 | 32.37 | 33.00 | 36.67 | 34.67 | 32.08 | 28.09 | 32.83 | 14.88 | 36.64 | 33.42 | 32.27 | 26.52 | 33.47 | 15.68 |
| 4h4f | 33.73 | 20.22 | 33.32 | 20.63 | 16.95 | 17.95 | 33.48 | 19.77 | 16.32 | 16.80 | 20.56 | 17.60 | 19.84 | 18.00 | 17.17 | 16.88 | 16.77 | 17.79 | 18.11 | 12.45 |
| 4hom | 9.33 | 8.64 | 34.43 | 34.19 | 33.77 | 34.18 | 8.68 | 34.04 | 33.94 | 33.63 | 35.92 | 29.27 | 10.77 | 11.48 | 35.04 | 34.92 | 23.89 | 34.29 | 35.58 | 35.61 |
| 4htp | 21.75 | 18.90 | 21.41 | 20.43 | 20.91 | 20.50 | 20.71 | 20.85 | 19.90 | 18.63 | 20.84 | 20.61 | 20.91 | 21.83 | 21.07 | 22.75 | 22.03 | 34.39 | 20.40 | 22.14 |
| 4iim | 13.59 | 16.36 | 21.62 | 14.93 | 23.96 | 15.94 | 16.20 | 27.70 | 13.68 | 15.41 | 16.42 | 16.74 | 14.94 | 18.45 | 12.12 | 16.99 | 25.71 | 16.43 | 25.03 | 21.57 |
| 4j8s | 31.25 | 21.53 | 23.70 | 38.20 | 38.24 | 22.37 | 39.29 | 29.51 | 21.00 | 23.54 | 21.47 | 35.98 | 43.94 | 41.43 | 36.48 | 32.08 | 24.80 | 22.68 | 30.52 | 37.04 |
| 4k0u | 19.33 | 8.31 | 19.56 | 17.89 | 18.87 | 17.50 | 10.74 | 16.62 | 21.54 | 14.49 | 18.16 | 19.77 | 22.48 | 15.85 | 17.81 | 17.97 | 21.75 | 18.73 | 18.61 | 18.04 |

**S5(f). I-RMSD values of all 20 poses obtained after blind docking by FRODOCK on 133 protein-peptide complexes.**

| **ID** | **Pose1** | **Pose2** | **Pose3** | **Pose4** | **Pose5** | **Pose6** | **Pose7** | **Pose8** | **Pose9** | **Pose10** | **Pose11** | **Pose12** | **Pose13** | **Pose14** | **Pose15** | **Pose16** | **Pose17** | **Pose18** | **Pose19** | **Pose20** |
| --- | --- | --- | --- | --- | --- | --- | --- | --- | --- | --- | --- | --- | --- | --- | --- | --- | --- | --- | --- | --- |
| 1cjr | 2.59 | 12.92 | 3.42 | 5.10 | 17.93 | 24.94 | 27.02 | 19.25 | 26.65 | 18.05 | 15.88 | 26.51 | 26.51 | 19.23 | 9.50 | 28.12 | 14.79 | 11.21 | 22.45 | 8.16 |
| 1cka | 2.16 | 5.54 | 12.91 | 5.20 | 15.17 | 15.84 | 9.87 | 7.50 | 9.86 | 18.78 | 9.80 | 16.27 | 4.62 | 8.72 | 15.30 | 14.00 | 21.67 | 18.33 | 11.14 | 21.82 |
| 1cvu | 37.16 | 37.63 | 54.59 | 37.08 | 35.65 | 38.65 | 14.60 | 38.28 | 39.38 | 44.47 | 20.70 | 43.96 | 38.01 | 26.17 | 30.03 | 38.29 | 39.92 | 17.93 | 55.17 | 54.77 |
| 1d4t | 0.88 | 8.86 | 14.86 | 18.76 | 28.09 | 19.15 | 17.77 | 15.40 | 20.58 | 6.94 | 20.87 | 19.57 | 21.02 | 14.86 | 19.16 | 9.55 | 6.40 | 10.43 | 26.77 | 17.79 |
| 1eg4 | 42.05 | 5.27 | 17.52 | 42.72 | 17.64 | 14.13 | 1.21 | 36.95 | 18.93 | 43.10 | 20.85 | 16.00 | 43.65 | 42.39 | 44.95 | 11.62 | 21.05 | 18.05 | 44.64 | 33.54 |
| 1h6w | 1.21 | 20.87 | 18.68 | 16.90 | 12.17 | 22.93 | 16.19 | 19.29 | 18.00 | 11.65 | 19.70 | 19.34 | 17.47 | 13.89 | 9.28 | 14.48 | 64.75 | 23.04 | 21.59 | 11.88 |
| 1hc9 | 1.90 | 7.40 | 11.16 | 9.58 | 6.66 | 20.63 | 13.40 | 25.03 | 21.26 | 10.62 | 21.78 | 21.89 | 16.95 | 12.27 | 22.59 | 22.75 | 20.48 | 19.51 | 20.60 | 18.54 |
| 1jbu | 1.71 | 46.56 | 41.86 | 46.98 | 4.31 | 47.59 | 45.63 | 43.07 | 46.75 | 28.56 | 46.05 | 46.20 | 27.73 | 44.88 | 46.02 | 41.99 | 18.55 | 47.52 | 41.86 | 6.35 |
| 1k5n | 1.12 | 24.84 | 26.30 | 15.27 | 25.93 | 26.43 | 27.07 | 9.02 | 27.04 | 23.92 | 15.07 | 27.01 | 26.79 | 13.76 | 30.48 | 25.31 | 23.45 | 25.27 | 27.88 | 48.96 |
| 1mfg | 10.74 | 8.82 | 11.38 | 14.98 | 19.92 | 2.04 | 14.50 | 10.94 | 16.12 | 21.30 | 17.07 | 24.03 | 16.44 | 12.09 | 23.18 | 13.78 | 28.78 | 14.50 | 20.01 | 27.77 |
| 1nln | 29.67 | 1.53 | 6.48 | 32.94 | 20.22 | 30.53 | 36.35 | 29.96 | 27.71 | 33.76 | 21.35 | 30.19 | 31.69 | 32.09 | 27.53 | 41.53 | 24.37 | 15.63 | 33.02 | 25.55 |
| 1nq7 | 37.86 | 37.21 | 40.74 | 44.73 | 35.45 | 41.56 | 37.44 | 1.32 | 25.13 | 40.61 | 21.33 | 43.97 | 36.40 | 44.69 | 35.29 | 17.78 | 44.68 | 21.48 | 26.99 | 33.24 |
| 1ntv | 1.84 | 16.19 | 15.50 | 4.37 | 15.38 | 13.63 | 10.84 | 26.47 | 7.96 | 14.92 | 9.75 | 29.57 | 15.16 | 8.45 | 14.20 | 14.87 | 15.60 | 17.77 | 24.81 | 31.25 |
| 1nx1 | 1.73 | 33.81 | 35.99 | 34.36 | 35.38 | 38.68 | 6.67 | 5.98 | 36.02 | 36.85 | 35.39 | 34.88 | 33.93 | 35.45 | 11.69 | 43.98 | 34.97 | 35.71 | 7.11 | 10.20 |
| 1oai | 10.59 | 27.87 | 22.30 | 21.62 | 23.90 | 23.52 | 22.32 | 19.66 | 3.75 | 24.94 | 20.40 | 26.21 | 3.00 | 23.64 | 21.39 | 24.06 | 11.81 | 9.90 | 24.71 | 24.41 |
| 1oj5 | 4.77 | 3.51 | 15.25 | 14.19 | 9.32 | 14.73 | 20.03 | 14.50 | 33.76 | 25.05 | 8.34 | 24.53 | 12.82 | 15.22 | 15.59 | 18.38 | 16.24 | 13.94 | 18.32 | 23.89 |
| 1ou8 | 1.28 | 5.94 | 32.08 | 14.00 | 31.06 | 30.58 | 18.27 | 31.90 | 11.17 | 12.56 | 28.01 | 12.72 | 15.91 | 8.49 | 31.14 | 28.16 | 26.82 | 15.78 | 26.38 | 12.15 |
| 1ow6 | 10.60 | 28.02 | 14.49 | 13.49 | 13.48 | 28.91 | 11.52 | 14.65 | 16.49 | 15.84 | 9.77 | 11.98 | 10.10 | 30.98 | 12.26 | 28.50 | 33.21 | 13.41 | 37.32 | 30.30 |
| 1pzl | 39.39 | 37.63 | 38.79 | 35.77 | 37.26 | 3.41 | 6.80 | 38.60 | 29.24 | 30.14 | 21.40 | 38.59 | 44.14 | 36.49 | 30.65 | 34.38 | 42.34 | 40.95 | 40.02 | 31.76 |
| 1qkz | 10.60 | 10.84 | 7.91 | 10.87 | 12.35 | 8.94 | 11.04 | 10.42 | 14.75 | 9.78 | 13.00 | 13.32 | 9.28 | 10.02 | 9.53 | 10.33 | 47.47 | 11.20 | 9.60 | 8.60 |
| 1rst | 7.62 | 8.91 | 8.39 | 10.35 | 9.64 | 9.63 | 9.66 | 8.40 | 13.56 | 2.95 | 12.34 | 10.58 | 23.29 | 6.34 | 14.95 | 9.12 | 10.28 | 8.18 | 5.49 | 13.55 |
| 1rxz | 1.79 | 17.92 | 16.29 | 16.37 | 27.70 | 11.41 | 17.12 | 38.69 | 24.90 | 34.29 | 20.97 | 15.88 | 14.95 | 20.00 | 12.72 | 8.84 | 35.66 | 16.69 | 9.74 | 14.63 |
| 1sfi | 7.63 | 11.13 | 14.82 | 2.63 | 15.20 | 25.51 | 13.40 | 13.11 | 9.15 | 14.47 | 18.65 | 25.69 | 17.24 | 13.52 | 23.40 | 24.04 | 12.42 | 10.66 | 28.42 | 12.95 |
| 1ssh | 1.31 | 18.81 | 19.94 | 7.13 | 20.02 | 8.99 | 10.28 | 4.35 | 6.21 | 10.13 | 21.00 | 8.65 | 6.64 | 18.61 | 16.41 | 9.34 | 11.10 | 9.46 | 20.01 | 18.59 |
| 1t08 | 1.65 | 40.15 | 33.57 | 41.57 | 15.33 | 45.21 | 30.40 | 29.78 | 31.84 | 18.63 | 33.50 | 43.85 | 13.49 | 22.51 | 32.10 | 35.62 | 46.92 | 22.97 | 23.12 | 43.37 |
| 1t4f | 0.99 | 23.45 | 8.50 | 18.37 | 10.21 | 5.69 | 15.94 | 21.99 | 7.03 | 24.43 | 8.98 | 16.78 | 21.56 | 9.70 | 22.84 | 22.74 | 12.77 | 22.84 | 25.92 | 16.29 |
| 1t7r | 1.20 | 35.86 | 39.90 | 38.78 | 43.70 | 39.42 | 34.26 | 35.35 | 25.47 | 44.26 | 42.91 | 39.02 | 40.57 | 39.72 | 40.04 | 45.86 | 34.15 | 4.15 | 39.19 | 39.15 |
| 1tfc | 1.67 | 30.82 | 16.01 | 20.19 | 34.55 | 27.62 | 27.88 | 19.32 | 31.30 | 29.26 | 24.55 | 25.12 | 30.32 | 14.49 | 40.20 | 18.54 | 23.26 | 4.77 | 27.55 | 36.91 |
| 1u00 | 2.73 | 14.72 | 17.29 | 5.72 | 18.82 | 14.60 | 16.93 | 21.43 | 14.44 | 17.38 | 18.03 | 6.65 | 18.19 | 14.51 | 27.72 | 33.40 | 5.83 | 25.98 | 34.09 | 17.59 |
| 1uj0 | 1.37 | 9.42 | 16.69 | 14.86 | 13.73 | 17.56 | 3.86 | 14.49 | 19.97 | 7.36 | 8.79 | 17.26 | 14.56 | 14.32 | 16.69 | 14.02 | 4.87 | 11.32 | 8.51 | 19.71 |
| 1x2r | 3.84 | 8.27 | 7.85 | 6.42 | 11.07 | 34.65 | 5.82 | 33.42 | 7.92 | 8.18 | 11.28 | 31.85 | 8.69 | 14.72 | 16.19 | 8.29 | 8.15 | 9.97 | 18.09 | 35.16 |
| 1xoc | 1.12 | 24.13 | 28.13 | 26.79 | 27.82 | 24.27 | 27.34 | 13.84 | 27.41 | 26.22 | 25.93 | 25.21 | 23.81 | 24.64 | 28.53 | 31.08 | 27.33 | 24.40 | 23.52 | 20.57 |
| 1ymt | 1.30 | 8.55 | 46.65 | 43.74 | 26.80 | 37.30 | 36.06 | 40.49 | 21.72 | 24.09 | 23.18 | 37.58 | 34.83 | 34.36 | 21.63 | 40.09 | 30.17 | 4.55 | 37.42 | 38.59 |
| 1yuc | 1.17 | 20.13 | 24.13 | 32.43 | 14.47 | 29.45 | 21.28 | 40.69 | 16.61 | 29.96 | 19.38 | 31.69 | 35.73 | 23.94 | 33.85 | 34.43 | 35.49 | 13.06 | 29.66 | 5.58 |
| 1ywo | 1.90 | 15.19 | 12.00 | 17.21 | 16.28 | 3.79 | 15.05 | 18.64 | 15.33 | 12.12 | 13.43 | 6.75 | 17.87 | 5.93 | 19.94 | 16.69 | 15.82 | 16.36 | 16.15 | 14.41 |
| 2a25 | 2.25 | 20.08 | 18.23 | 16.31 | 23.14 | 24.84 | 16.98 | 19.93 | 26.32 | 30.78 | 27.73 | 7.49 | 5.61 | 27.09 | 16.21 | 16.82 | 7.00 | 13.12 | 22.33 | 16.37 |
| 2a3i | 40.39 | 43.67 | 36.05 | 42.77 | 39.48 | 0.71 | 41.63 | 44.09 | 44.55 | 42.03 | 33.64 | 41.98 | 39.94 | 45.60 | 38.60 | 40.93 | 38.19 | 38.11 | 37.13 | 29.81 |
| 2aq9 | 49.18 | 21.46 | 35.22 | 28.24 | 34.76 | 18.38 | 33.85 | 12.99 | 30.75 | 35.62 | 30.36 | 13.40 | 29.53 | 38.32 | 38.85 | 33.00 | 27.97 | 32.94 | 41.54 | 34.89 |
| 2b9h | 28.89 | 2.50 | 30.36 | 40.99 | 24.04 | 28.41 | 44.49 | 43.31 | 25.05 | 45.35 | 48.92 | 31.20 | 31.63 | 36.51 | 20.01 | 22.50 | 49.80 | 50.69 | 19.15 | 21.00 |
| 2bba | 3.42 | 39.21 | 46.18 | 3.45 | 27.50 | 49.21 | 47.44 | 30.13 | 7.15 | 50.04 | 32.74 | 44.71 | 44.40 | 42.66 | 51.33 | 34.18 | 46.41 | 6.68 | 51.91 | 8.49 |
| 2cch | 32.78 | 39.88 | 37.31 | 35.64 | 31.13 | 1.37 | 35.62 | 34.60 | 34.53 | 40.10 | 47.08 | 33.68 | 31.40 | 36.29 | 33.79 | 39.94 | 34.84 | 39.53 | 37.92 | 36.36 |
| 2ce8 | 32.47 | 0.94 | 39.88 | 30.99 | 41.11 | 32.04 | 41.13 | 40.71 | 41.34 | 40.90 | 41.57 | 38.20 | 40.83 | 40.51 | 41.25 | 41.46 | 44.47 | 35.80 | 43.09 | 38.43 |
| 2d0n | 1.47 | 5.61 | 7.82 | 14.10 | 4.94 | 10.23 | 26.94 | 15.50 | 15.42 | 21.20 | 5.91 | 14.14 | 8.73 | 18.50 | 5.97 | 15.21 | 13.02 | 12.29 | 23.64 | 14.23 |
| 2drk | 17.55 | 2.42 | 12.76 | 10.52 | 17.11 | 9.78 | 20.01 | 17.00 | 16.68 | 9.14 | 17.85 | 16.45 | 9.24 | 6.27 | 12.60 | 17.89 | 18.02 | 17.18 | 17.54 | 17.76 |
| 2dyp | 19.94 | 8.25 | 19.46 | 20.16 | 26.55 | 10.89 | 26.34 | 7.68 | 23.80 | 22.98 | 18.09 | 25.89 | 25.83 | 26.23 | 20.72 | 61.40 | 20.50 | 54.76 | 26.14 | 22.45 |
| 2fff | 2.70 | 53.37 | 53.55 | 49.96 | 52.94 | 49.24 | 8.85 | 12.94 | 57.64 | 12.15 | 54.28 | 53.96 | 13.25 | 58.82 | 51.96 | 9.67 | 51.86 | 56.36 | 12.10 | 56.26 |
| 2ffu | 1.64 | 14.15 | 4.49 | 14.87 | 15.84 | 15.77 | 16.37 | 19.54 | 21.37 | 16.74 | 14.88 | 11.02 | 6.33 | 13.75 | 24.14 | 14.65 | 15.90 | 5.90 | 18.28 | 14.71 |
| 2fka | 7.14 | 25.16 | 26.10 | 25.86 | 24.97 | 11.56 | 24.62 | 14.57 | 29.82 | 28.30 | 28.03 | 30.62 | 11.91 | 28.88 | 28.03 | 8.37 | 10.75 | 28.40 | 33.25 | 27.07 |
| 2fmf | 5.49 | 10.40 | 0.85 | 11.50 | 27.03 | 26.04 | 8.69 | 14.00 | 10.15 | 5.32 | 27.60 | 10.24 | 28.04 | 24.30 | 28.03 | 30.44 | 28.07 | 31.68 | 11.55 | 26.12 |
| 2fts | 28.12 | 1.19 | 22.01 | 17.72 | 47.24 | 5.42 | 25.54 | 33.95 | 18.69 | 18.95 | 6.00 | 33.40 | 15.10 | 35.73 | 14.28 | 19.39 | 4.96 | 16.01 | 11.02 | 29.65 |
| 2fvj | 42.22 | 1.39 | 34.51 | 46.39 | 42.28 | 22.90 | 46.42 | 42.24 | 46.06 | 44.75 | 45.86 | 25.21 | 44.03 | 43.72 | 49.70 | 23.02 | 46.04 | 46.13 | 43.16 | 44.96 |
| 2ho2 | 14.26 | 11.57 | 11.33 | 6.48 | 6.63 | 12.76 | 15.62 | 6.66 | 11.85 | 12.36 | 10.78 | 19.71 | 21.46 | 16.34 | 9.51 | 11.75 | 18.61 | 10.90 | 5.98 | 7.10 |
| 2ht9 | 29.65 | 22.03 | 25.93 | 22.11 | 27.52 | 24.83 | 23.99 | 18.92 | 12.57 | 21.71 | 21.35 | 20.22 | 4.16 | 27.28 | 7.42 | 28.71 | 24.86 | 26.72 | 22.26 | 22.51 |
| 2o02 | 20.05 | 28.61 | 21.53 | 2.59 | 16.82 | 20.21 | 23.87 | 28.43 | 33.05 | 23.07 | 34.44 | 24.79 | 22.07 | 23.40 | 26.85 | 23.49 | 7.38 | 21.49 | 35.47 | 24.15 |
| 2o4j | 30.11 | 30.10 | 37.62 | 22.72 | 24.27 | 23.19 | 19.27 | 40.46 | 36.55 | 33.45 | 35.47 | 33.02 | 39.82 | 31.21 | 34.33 | 31.95 | 28.57 | 20.46 | 33.44 | 38.02 |
| 2o9v | 1.95 | 16.62 | 15.07 | 9.69 | 5.85 | 17.54 | 20.86 | 31.35 | 18.09 | 16.43 | 19.56 | 26.11 | 11.55 | 32.88 | 18.28 | 9.82 | 17.37 | 17.32 | 18.26 | 5.76 |
| 2oei | 15.08 | 10.76 | 6.67 | 13.84 | 16.27 | 1.45 | 14.34 | 3.53 | 13.67 | 13.83 | 7.01 | 21.07 | 16.24 | 16.57 | 21.40 | 11.06 | 17.14 | 15.51 | 17.92 | 13.99 |
| 2p0w | 1.16 | 27.27 | 38.91 | 14.27 | 17.38 | 21.42 | 31.59 | 21.98 | 32.15 | 28.14 | 18.62 | 19.22 | 20.90 | 25.48 | 23.44 | 33.89 | 40.89 | 36.28 | 18.23 | 24.75 |
| 2p1o | 7.23 | 13.36 | 3.21 | 11.23 | 14.13 | 10.14 | 10.97 | 11.93 | 8.47 | 3.75 | 10.49 | 15.92 | 11.12 | 11.86 | 11.54 | 11.23 | 11.55 | 7.90 | 13.99 | 39.28 |
| 2p1t | 37.08 | 37.43 | 21.80 | 32.37 | 37.52 | 38.67 | 39.25 | 24.83 | 25.61 | 37.16 | 26.85 | 25.19 | 39.62 | 33.07 | 35.73 | 39.74 | 38.65 | 26.73 | 37.59 | 35.00 |
| 2p54 | 0.79 | 44.79 | 45.71 | 45.40 | 43.16 | 46.49 | 43.74 | 24.00 | 43.53 | 40.69 | 22.87 | 43.68 | 27.13 | 44.19 | 41.72 | 43.54 | 26.19 | 32.66 | 48.49 | 22.76 |
| 2peh | 1.02 | 5.41 | 33.42 | 31.30 | 5.09 | 9.71 | 31.08 | 37.35 | 27.71 | 11.67 | 27.72 | 29.60 | 9.87 | 18.33 | 23.45 | 10.18 | 26.67 | 36.31 | 31.81 | 28.93 |
| 2pux | 1.36 | 45.91 | 34.35 | 32.01 | 36.21 | 34.14 | 48.75 | 30.91 | 31.07 | 32.80 | 42.77 | 29.35 | 29.68 | 47.82 | 43.67 | 41.60 | 49.85 | 31.88 | 32.48 | 38.74 |
| 2puy | 2.38 | 29.57 | 22.88 | 21.54 | 20.94 | 18.84 | 20.22 | 20.39 | 21.41 | 24.11 | 18.21 | 28.54 | 27.97 | 12.01 | 12.98 | 16.19 | 15.28 | 28.05 | 21.81 | 21.61 |
| 2qbx | 15.72 | 2.88 | 37.93 | 18.61 | 36.70 | 15.95 | 8.81 | 44.97 | 8.77 | 24.03 | 37.79 | 39.62 | 22.12 | 36.04 | 44.44 | 39.78 | 42.77 | 40.40 | 39.08 | 6.20 |
| 2qos | 1.43 | 5.86 | 6.90 | 18.02 | 19.28 | 19.76 | 24.97 | 6.55 | 6.97 | 26.41 | 19.49 | 11.69 | 5.41 | 27.23 | 11.64 | 28.34 | 26.59 | 9.76 | 9.02 | 7.98 |
| 2qse | 1.34 | 31.49 | 28.82 | 31.65 | 30.53 | 35.63 | 27.76 | 17.62 | 31.50 | 30.76 | 32.58 | 30.80 | 33.69 | 34.76 | 29.81 | 9.90 | 33.09 | 34.54 | 30.99 | 34.37 |
| 2r7g | 1.06 | 20.11 | 21.56 | 27.06 | 18.76 | 37.25 | 27.96 | 16.27 | 31.63 | 33.69 | 48.89 | 33.53 | 39.86 | 39.48 | 40.18 | 30.70 | 46.99 | 46.24 | 8.25 | 47.19 |
| 2r9q | 4.50 | 10.70 | 8.30 | 5.49 | 7.83 | 10.19 | 29.67 | 11.55 | 29.08 | 8.24 | 11.53 | 11.28 | 34.99 | 9.49 | 8.50 | 5.42 | 6.51 | 30.55 | 28.34 | 8.92 |
| 2v8y | 2.38 | 9.27 | 16.34 | 16.78 | 14.71 | 14.83 | 17.26 | 5.93 | 3.86 | 37.85 | 13.78 | 15.28 | 17.18 | 15.05 | 15.61 | 8.82 | 8.62 | 39.02 | 12.21 | 6.32 |
| 2vkn | 3.01 | 21.52 | 9.67 | 15.45 | 21.47 | 21.27 | 17.66 | 16.48 | 17.07 | 22.39 | 21.66 | 25.58 | 5.61 | 19.76 | 14.58 | 25.96 | 15.44 | 22.60 | 24.07 | 10.39 |
| 2vr3 | 26.56 | 2.74 | 11.86 | 24.97 | 23.19 | 27.06 | 26.83 | 40.39 | 21.81 | 31.19 | 46.27 | 29.05 | 30.94 | 31.05 | 25.63 | 10.26 | 24.07 | 37.68 | 32.11 | 20.47 |
| 2vwf | 1.25 | 30.93 | 25.62 | 24.49 | 27.41 | 28.01 | 32.30 | 31.60 | 25.86 | 12.03 | 8.30 | 31.51 | 31.37 | 9.92 | 8.46 | 26.94 | 34.46 | 29.28 | 18.68 | 34.49 |
| 2w2u | 0.83 | 6.89 | 20.11 | 15.77 | 21.73 | 16.16 | 15.73 | 15.39 | 21.99 | 11.35 | 10.64 | 10.15 | 20.28 | 15.92 | 13.93 | 26.38 | 6.94 | 9.83 | 15.10 | 17.77 |
| 2whx | 1.57 | 25.27 | 25.56 | 59.07 | 25.92 | 26.23 | 26.99 | 25.92 | 29.45 | 25.01 | 30.06 | 43.88 | 18.42 | 25.53 | 27.09 | 6.60 | 54.03 | 30.69 | 28.50 | 25.55 |
| 2xrw | 1.08 | 47.80 | 44.20 | 36.22 | 43.82 | 38.17 | 41.38 | 18.20 | 22.33 | 44.43 | 31.36 | 5.65 | 50.37 | 40.39 | 24.53 | 29.67 | 21.59 | 32.99 | 17.17 | 48.52 |
| 2xu7 | 1.97 | 6.45 | 7.72 | 5.81 | 37.43 | 7.04 | 14.94 | 14.56 | 37.05 | 28.59 | 3.85 | 7.10 | 33.76 | 8.86 | 35.25 | 24.58 | 20.22 | 15.18 | 27.01 | 17.09 |
| 2xvc | 1.26 | 8.13 | 21.46 | 23.47 | 20.50 | 24.33 | 21.24 | 21.48 | 21.01 | 26.28 | 24.40 | 29.28 | 34.75 | 23.35 | 21.93 | 28.47 | 24.44 | 8.84 | 5.51 | 23.31 |
| 2zjd | 30.25 | 31.62 | 1.31 | 31.95 | 31.99 | 30.03 | 16.87 | 33.22 | 32.06 | 10.54 | 33.80 | 29.56 | 30.19 | 34.57 | 28.74 | 31.33 | 35.52 | 28.24 | 35.91 | 34.71 |
| 3asl | 22.94 | 21.12 | 0.75 | 22.21 | 24.80 | 24.07 | 24.32 | 20.64 | 22.63 | 22.80 | 24.97 | 12.24 | 21.39 | 24.37 | 25.95 | 20.58 | 24.85 | 23.95 | 26.09 | 24.78 |
| 3awr | 24.47 | 21.96 | 22.91 | 12.99 | 26.43 | 11.33 | 21.23 | 19.17 | 24.98 | 24.52 | 25.21 | 23.82 | 11.74 | 25.16 | 12.66 | 10.32 | 3.38 | 9.50 | 22.63 | 7.73 |
| 3ayu | 1.29 | 7.88 | 16.35 | 17.40 | 7.53 | 17.34 | 12.43 | 16.44 | 17.25 | 17.52 | 8.53 | 16.34 | 19.19 | 16.94 | 18.45 | 21.49 | 12.25 | 16.99 | 21.19 | 34.32 |
| 3bfq | 0.89 | 27.51 | 13.96 | 28.11 | 37.22 | 11.09 | 28.96 | 31.78 | 27.61 | 30.42 | 33.13 | 28.07 | 32.89 | 8.97 | 16.69 | 5.00 | 32.37 | 29.91 | 34.52 | 15.88 |
| 3c3r | 1.17 | 51.96 | 42.06 | 42.71 | 25.17 | 13.44 | 39.58 | 23.09 | 38.19 | 11.80 | 38.86 | 5.76 | 53.17 | 7.09 | 26.62 | 32.65 | 15.88 | 40.76 | 8.55 | 47.64 |
| 3d32 | 20.63 | 1.55 | 8.16 | 18.24 | 23.43 | 25.73 | 15.34 | 15.39 | 26.78 | 28.56 | 30.35 | 33.28 | 8.05 | 32.68 | 29.63 | 23.66 | 17.82 | 25.74 | 23.82 | 26.48 |
| 3ds4 | 1.54 | 11.38 | 24.80 | 11.04 | 26.04 | 22.27 | 26.49 | 23.12 | 22.36 | 24.28 | 31.21 | 24.48 | 25.27 | 27.86 | 11.11 | 29.44 | 12.23 | 24.25 | 7.13 | 24.39 |
| 3ery | 1.41 | 7.92 | 15.13 | 15.33 | 5.74 | 11.59 | 14.65 | 10.47 | 8.84 | 7.94 | 9.52 | 5.87 | 15.26 | 12.37 | 15.38 | 19.18 | 18.14 | 17.38 | 14.15 | 16.10 |
| 3fdo | 0.62 | 17.99 | 11.72 | 19.29 | 6.31 | 11.55 | 12.39 | 22.01 | 9.84 | 15.53 | 13.95 | 17.05 | 10.27 | 16.45 | 20.99 | 18.40 | 18.51 | 19.19 | 11.46 | 5.71 |
| 3g2s | 1.32 | 31.57 | 25.33 | 14.26 | 11.10 | 9.13 | 36.83 | 22.36 | 17.08 | 19.93 | 27.12 | 23.43 | 12.83 | 17.58 | 24.24 | 25.84 | 5.60 | 16.49 | 16.67 | 25.19 |
| 3gyt | 0.70 | 43.25 | 13.10 | 40.94 | 44.84 | 47.00 | 15.40 | 6.60 | 31.64 | 43.09 | 5.06 | 8.52 | 6.91 | 6.31 | 11.07 | 15.15 | 9.41 | 16.60 | 4.76 | 12.18 |
| 3h1z | 38.38 | 22.45 | 23.56 | 21.17 | 45.08 | 31.49 | 28.98 | 36.05 | 24.75 | 25.78 | 15.93 | 57.00 | 20.89 | 24.13 | 31.01 | 24.58 | 28.74 | 28.90 | 29.10 | 26.34 |
| 3i5r | 15.61 | 0.68 | 17.02 | 17.10 | 12.69 | 13.18 | 15.39 | 15.88 | 7.79 | 13.34 | 15.88 | 7.13 | 9.73 | 16.42 | 12.96 | 17.20 | 15.99 | 18.95 | 15.43 | 19.45 |
| 3ivv | 18.40 | 1.23 | 6.57 | 18.81 | 20.47 | 20.22 | 20.61 | 27.01 | 17.48 | 14.78 | 8.28 | 15.31 | 17.76 | 15.14 | 6.97 | 18.35 | 16.85 | 13.66 | 23.02 | 15.92 |
| 3kmr | 37.94 | 26.19 | 34.71 | 38.60 | 36.92 | 3.48 | 25.70 | 41.29 | 33.92 | 32.20 | 37.50 | 20.91 | 32.84 | 37.27 | 39.46 | 35.12 | 23.50 | 36.84 | 28.47 | 20.46 |
| 3kuj | 21.72 | 4.57 | 19.02 | 19.88 | 21.69 | 25.11 | 20.32 | 17.10 | 24.41 | 28.32 | 7.64 | 22.08 | 14.83 | 28.79 | 21.33 | 22.81 | 26.03 | 27.05 | 20.82 | 24.49 |
| 3kus | 2.06 | 23.76 | 19.42 | 20.25 | 24.67 | 24.86 | 24.23 | 4.65 | 26.51 | 21.08 | 17.38 | 22.67 | 18.58 | 23.79 | 18.05 | 24.39 | 19.81 | 24.03 | 24.31 | 27.07 |
| 3l0e | 19.58 | 39.90 | 26.29 | 29.55 | 31.40 | 24.27 | 22.13 | 41.18 | 33.57 | 26.71 | 25.42 | 26.54 | 33.46 | 38.04 | 22.37 | 38.24 | 25.94 | 39.76 | 23.96 | 26.31 |
| 3ll8 | 46.18 | 42.01 | 37.90 | 49.27 | 49.84 | 48.20 | 40.88 | 55.53 | 38.64 | 46.40 | 47.15 | 46.44 | 40.95 | 41.90 | 49.30 | 39.08 | 40.35 | 45.91 | 54.25 | 51.27 |
| 3llz | 1.61 | 19.83 | 19.89 | 7.58 | 4.54 | 21.68 | 23.48 | 7.31 | 23.83 | 18.91 | 19.63 | 14.77 | 31.92 | 20.20 | 19.25 | 32.52 | 15.69 | 19.77 | 21.49 | 24.00 |
| 3obq | 1.83 | 17.22 | 19.31 | 17.79 | 13.73 | 5.41 | 15.35 | 8.85 | 15.39 | 5.65 | 6.21 | 34.78 | 14.37 | 23.03 | 22.93 | 28.66 | 27.25 | 14.30 | 8.57 | 14.40 |
| 3olf | 35.86 | 34.21 | 38.09 | 32.70 | 14.57 | 34.89 | 35.25 | 20.83 | 23.01 | 21.37 | 26.21 | 33.97 | 37.62 | 44.52 | 24.76 | 35.13 | 22.34 | 26.31 | 34.79 | 26.97 |
| 3p72 | 6.59 | 6.45 | 36.57 | 34.79 | 34.12 | 9.50 | 36.19 | 34.18 | 37.91 | 36.00 | 33.75 | 33.39 | 36.80 | 33.46 | 9.65 | 38.31 | 36.01 | 35.16 | 32.62 | 33.47 |
| 3p8f | 1.11 | 13.14 | 6.96 | 13.36 | 7.02 | 9.45 | 13.64 | 7.43 | 11.53 | 23.57 | 13.51 | 15.15 | 13.77 | 11.06 | 12.94 | 7.78 | 14.01 | 20.53 | 15.63 | 13.39 |
[truncated: 440,514 more chars]
