# Supplementary figures and images for: Benchmarking of different molecular docking methods for protein-peptide docking
Source: BMC Bioinformatics. 2019 Feb 4;19(Suppl 13):426. doi: 10.1186/s12859-018-2449-y (PMC7394329; doi:10.1186/s12859-018-2449-y)

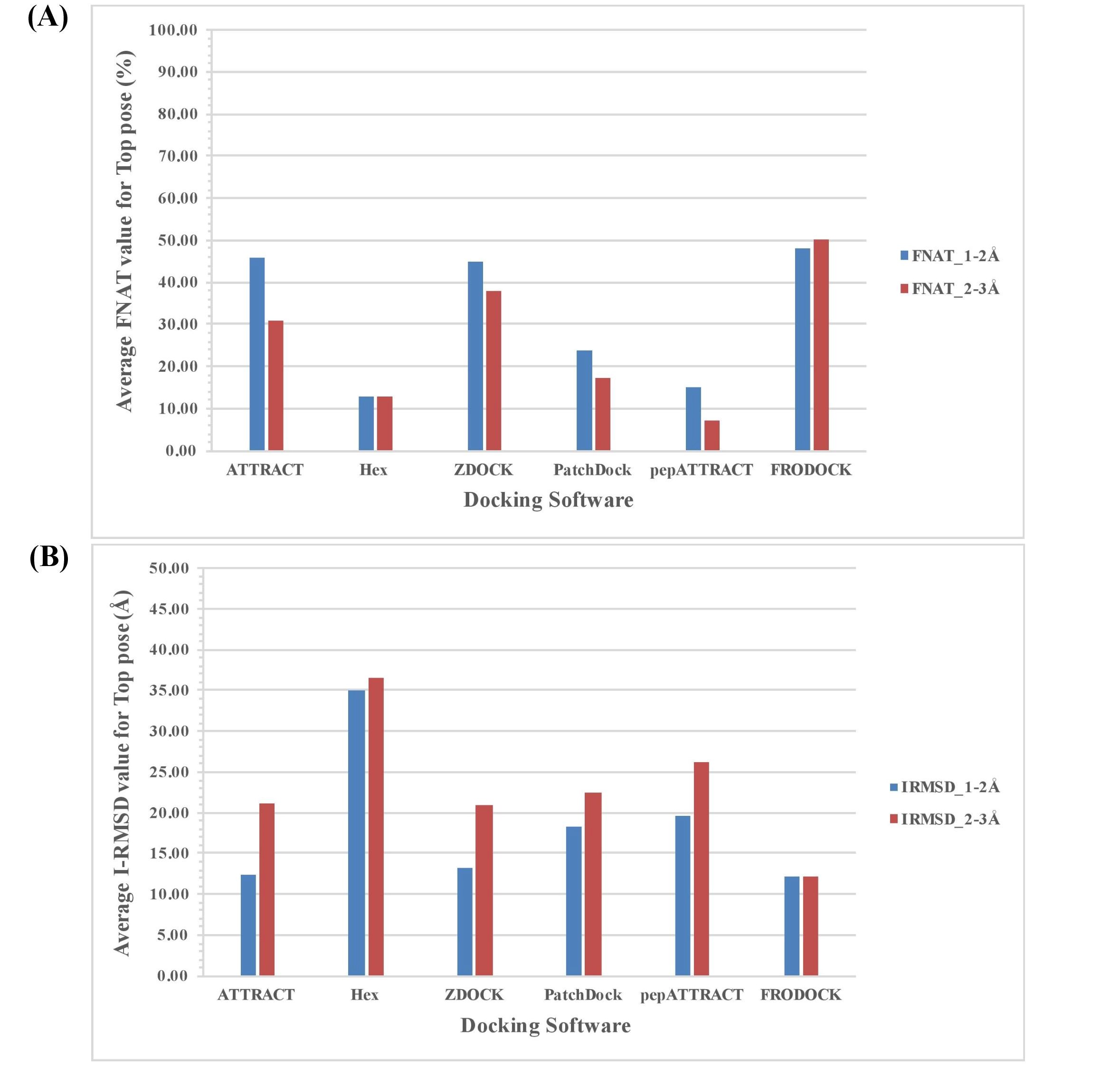

Supplement: Supplementary file 2 — Performance of different docking method on the PPDbench dataset with resolution 1–2 Å and 2–3 Å for top pose based on average (a) FNAT value and (b) I-RMSD respectively. (JPG 349 kb) [file 12859_2018_2449_MOESM2_ESM.jpg]

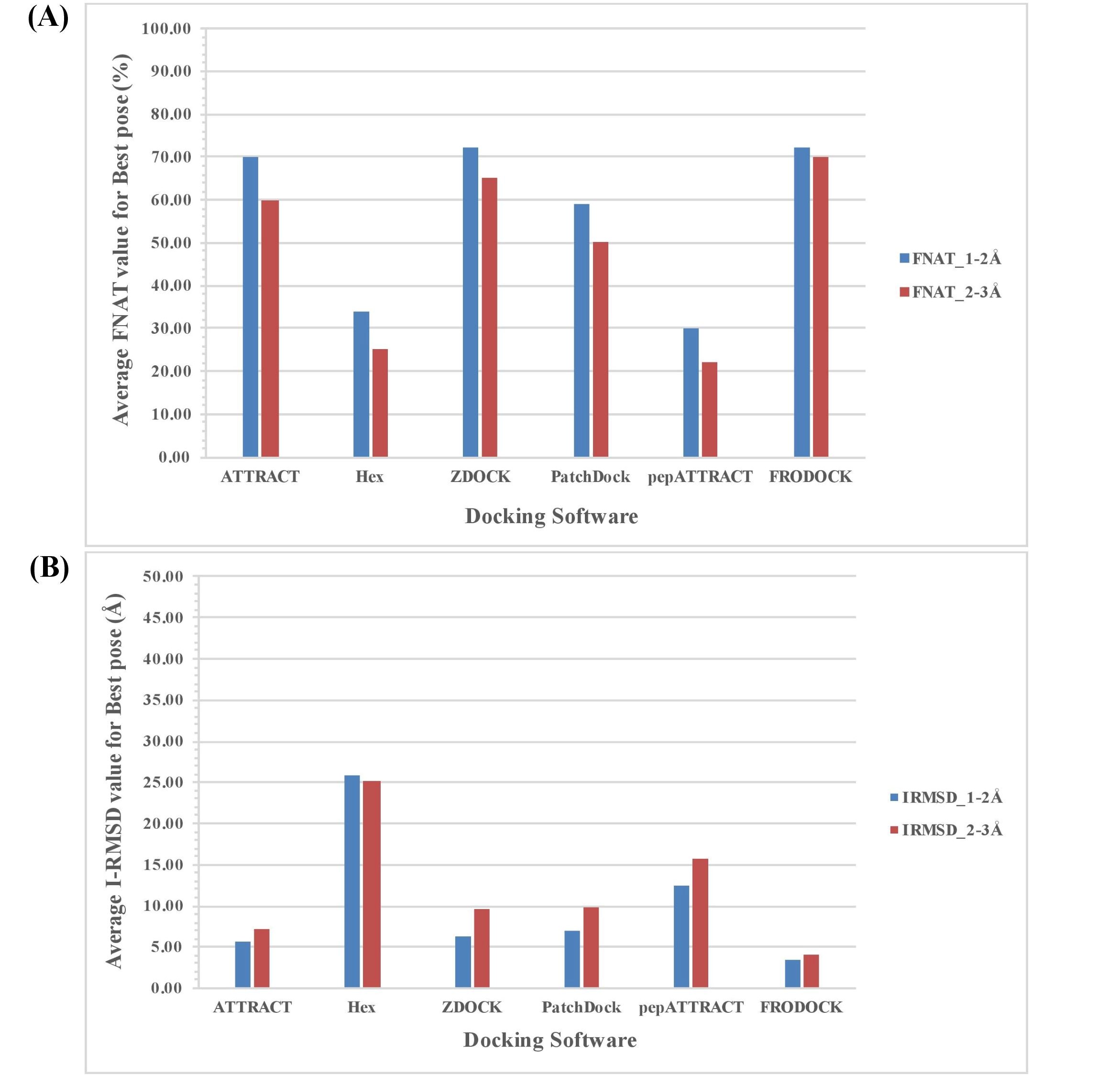

Supplement: Supplementary file 3 — Performance of different docking method on the PPDbench dataset with resolution 1–2 Å and 2–3 Å for best pose based on average (a) FNAT value and (b) I-RMSD respectively. (JPG 352 kb) [file 12859_2018_2449_MOESM3_ESM.jpg]

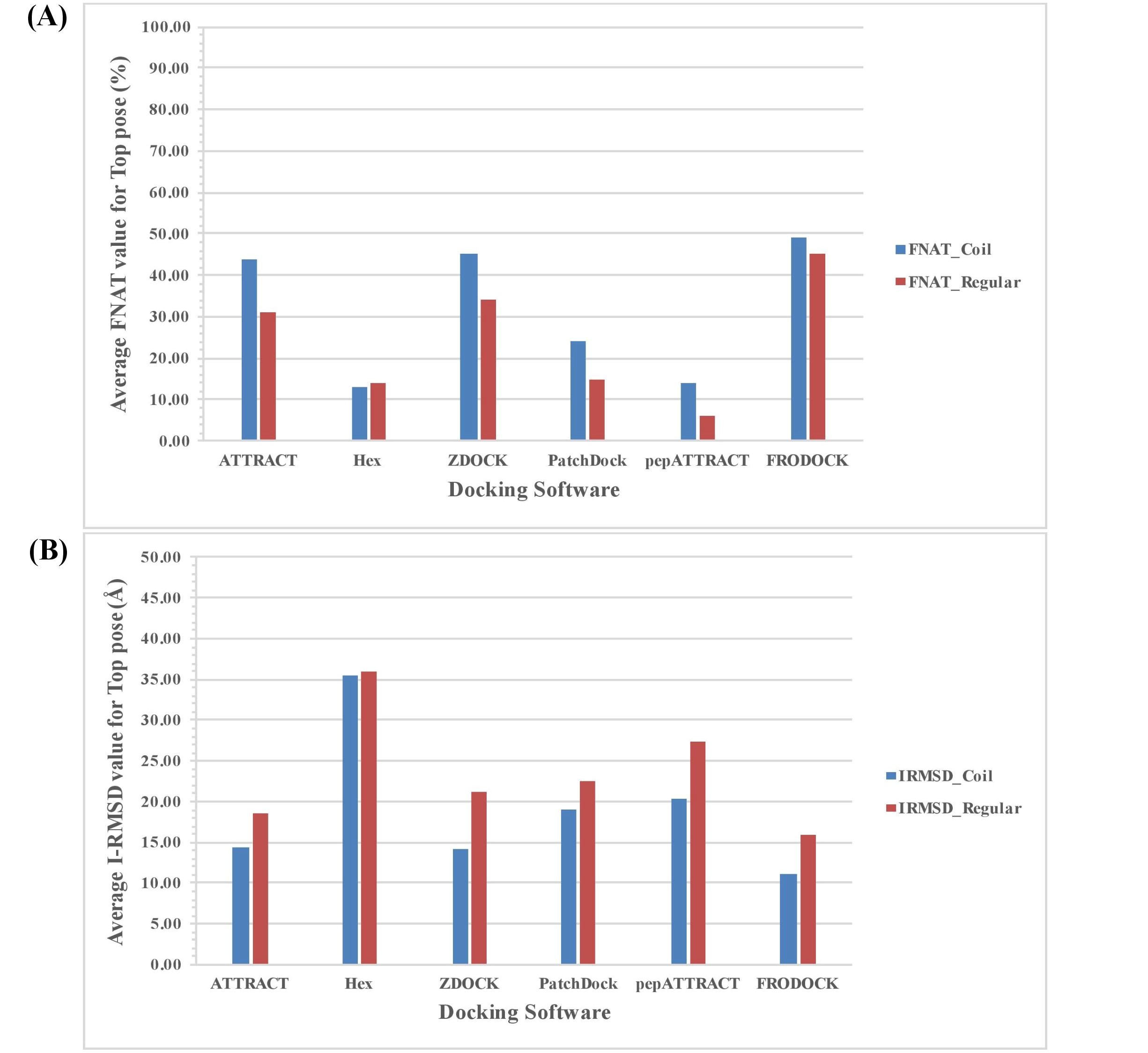

Supplement: Supplementary file 4 — Performance of different docking methods on the PPDbench dataset with the different secondary structure for top pose based on (a) FNAT and (b) I-RMSD value respectively. (JPG 362 kb) [file 12859_2018_2449_MOESM4_ESM.jpg]

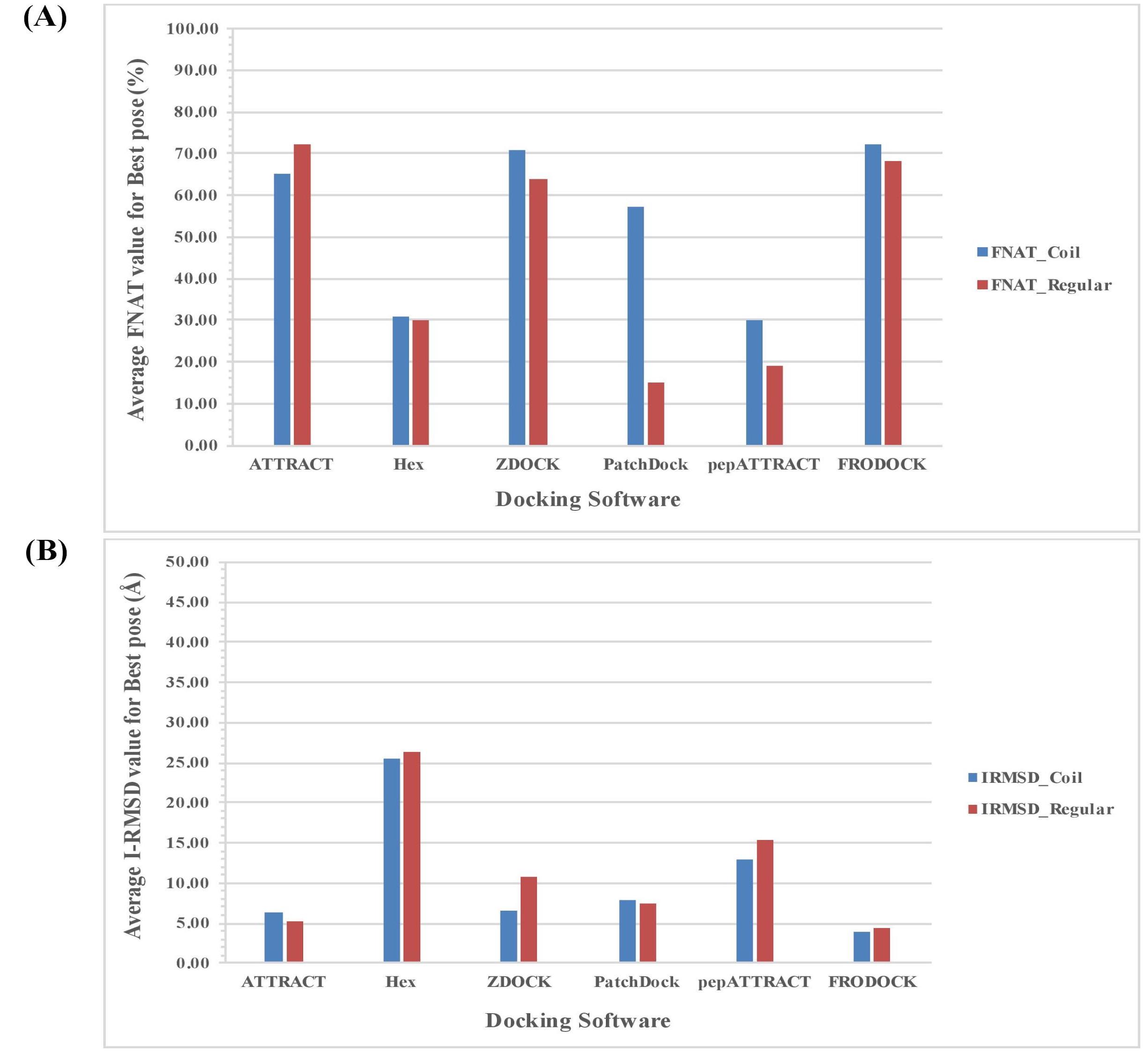

Supplement: Supplementary file 5 — Performance of different docking methods on the PPDbench dataset with the different secondary structure for best pose based on (a) FNAT and (b) I-RMSD value respectively. (JPG 274 kb) [file 12859_2018_2449_MOESM5_ESM.jpg]
